# Supplementary material for: Scalable total synthesis and comprehensive structure–activity relationship studies of the phytotoxin coronatine
Source: Nat Commun. 2018 Mar 16;9:1105. doi: 10.1038/s41467-018-03443-1 (PMC5856746; doi:10.1038/s41467-018-03443-1)
Supplement: Supplementary file 2 — Supplementary Information(PDF 56145 kb) [file 41467_2018_3443_MOESM2_ESM.pdf]

Scalable total synthesis and comprehensive structure–activity relationship studies of the phytotoxin coronatine

Mairi M. Littleson,[1] Christopher M. Baker,[2] Anne J. Dalencon,[2] Elizabeth C. Frye,[2] Craig Jamieson,[1] Alan R. Kennedy,[1] Kenneth B. Ling,[2] Matthew M. McLachlan,[2] Mark G. Montgomery,[2] Claire J. Russell,[2] and Allan J. B. Watson[3]*

[1]Department of Pure and Applied Chemistry, University of Strathclyde, 295 Cathedral Street, Glasgow, G1 1XL, UK. [2]Syngenta, Jealott's Hill International Research Centre, Bracknell, Berkshire, RG42 6EY, UK. [3]EaStCHEM, School of Chemistry, University of St. Andrews, North Haugh, St. Andrews, KY16 9ST, UK. *e-mail: aw260@st-andrews.ac.uk

Supplementary Information

Contents

General Techniques
General Experimental Procedures
Procedures and Characterization
Supplementary Figures 1–339 (Spectra)
Supplementary References

Supplementary Methods

General Techniques

All reagents and solvents were obtained from commercial suppliers and were used without further purification unless otherwise stated. Purification was carried out according to standard laboratory methods.[1]

Purification of Solvents

All solvents used for anhydrous reactions (THF, CH_2Cl_2, PhH, MeOH) were ether obtained from a PureSolv SPS-400-5 solvent purification system or dried over previously activated 3 Å molecular sieves. These solvents were transferred to and stored in a septum-sealed oven-dried flask over previously activated 3 Å molecular sieves and purged with and stored under nitrogen. CH_2Cl_2, Et_2O, EtOAc, MeOH, and petroleum ether 40-60 °C for purification purposes were used as obtained from suppliers without further purification.

Experimental Details

Air-sensitive reactions were carried out using conventional glassware. The glassware was oven-dried (150 °C) and purged with N_2 before use. Purging refers to a vacuum/nitrogen-refilling procedure. Reactions were carried out at −78 °C using dry ice/acetone baths. Reactions were carried out at 0 °C using ice/water baths. Room temperature was generally *ca*. 18 °C. Reactions were carried out at elevated temperatures using a temperature-regulated hotplate/stirrer. DIPEA for aldol additions was dried by heating to reflux over CaH_2 and distilling under vacuum before being purged with, and stored under N_2 in a septum-sealed oven-dried flask over previously activated 3 Å molecular sieves.

Purification of Products

Thin layer chromatography was carried out using Merck silica plates coated with fluorescent indicator UV254. These were analysed under 254 nm UV light and/or developed using potassium permanganate solution. Flash chromatography was carried out using ZEOprep 60 HYD 40-63 µm silica gel.

Analysis of Products

Fourier Transformed Infra-Red (FTIR) spectra were obtained on a Shimadzu IRAffinity-1 machine. ^1H and ^{13}C NMR spectra were obtained on a Bruker AV 400 spectrometer at 400 MHz and 125 MHz, respectively, or Bruker DRX 500 at 500 MHz and 126 MHz, respectively. ^{19}F NMR spectra were obtained on a Bruker AV 400 or Bruker DRX 500 spectrometer at 376 MHz and 471 MHz respectively. Chemical shifts are reported in ppm and coupling constants are reported in Hz with $CDCl_3$ referenced at 7.26 ppm (^1H) and 77.16 ppm (^{13}C), DMSO-d_6 referenced at 2.50 ppm (^1H) and 39.52 ppm (^{13}C), acetone-d_6 referenced at 2.05 ppm (^1H) and 29.84 ppm (^{13}C), D_2O referenced at 4.79 ppm (^1H), and MeOD referenced at 3.31 ppm (^1H) and 49.00 ppm (^{13}C). High-resolution mass spectra were obtained through analysis at the EPSRC UK National Mass Spectrometry Facility at Swansea University. Robot array compounds were purified by mass directed prep HPLC, using a mixed trigger of UV with ES+ on a Waters Fraction Lynx system comprising of a 2767 injector/collector with a 2545 gradient pump, two 515 isocratic pumps, SFO, 2998 photodiode array, 2424 ELSD, and 3100 mass spectrometers. A Waters XBridge dC18 5micron 19x10 mm guard column was used with an ACT ACE C18- AR, 5micron 30 x 100 mm prep column. The preparative HPLC was conducted using a 11.4 minute run time using a gradient method, eluting with MeCN (0.05% TFA)/H_2O (0.05% TFA) at a flow rate of 33 mL/min. Chiral HPLC purification was performed on a Waters FractionLynx Prep HPLC with PDA UV detection and chiral analysis was performed on a Waters Alliance HPLC with PDA UV detection.

Where compounds were obtained as 1:1 mixtures of two diastereoisomers ((±)-coronafacic acid or coronafacic acid analogue conjugates with enantiopure amino acids or (±)-coronafacic acid or coronafacic acid analogue conjugates with (±)-amino

acids, e.g., (±)-coronamic acid), ^{1}H NMR peaks corresponding to both diastereoisomers were integrated together and integration normalised to one. ^{13}C NMR signals are reported as observed.

Docking Studies

Docking studies were performed using protein data bank[2] (PDB) crystal structure 3OGK,[3] with the binding site occupied by ligand B selected as the target site for docking. The rotameric states of residues TRP519 and TRP467 in this binding site were reassigned to provide a better fit to the bound ligand before H atoms were added and protonation states assigned using the protein preparation wizard[4] from the 2017-01 release of the Schrodinger Suite. With a complete protein model in place, docking calculations were performed using the program Glide,[5,6] accessed via Maestro.[7] A Glide grid file was generated centred on the centroid of the bound coronatine molecule, with a cubic box of length 25 Å. All other options for grid generation were retained at their default values. The five molecules shown in Figure 28 were built in Maestro, and then docked using the standard precision mode of Glide: all options were assigned their default values, with only the highest scoring docking pose retained for each molecule.

HPLC Separation

Compound **45a** was separated by HPLC on a Chiralpak IC, 5µm, 250 x 21.2 mm id column with 20% EtOH in 2-methylpentane at 20 mL/min. Compound **43a** was separated by HPLC on a Regis (*S*,*S*) Whelk-O1, 5/100, 250 x 4.6 mm id column with 15% EtOH in 2-methylpentane at 20 mL/min.

Biological Testing

Supplementary Table 1. Herbicide Glasshouse screen (GH1):

The substances were dissolved in dimethyl sulfoxide for storage. Sub-samples to be tested were dried down and formulated into spray solution of acetone, water and Tween 20 for application. The compounds were tested for pre- and post-emergence activity against four weed species, with the compounds applied at 1000g/ha. The plants were then placed in the glasshouse for 12 days. The weeds tested were *Amaranthus retroflexus*, *Stellaria media*, *Lolium perenne* and *Digitaria sanguinalis*. Assessments were made of % phytotoxicity and converted to a banded score between 0 and 100, where complete control of the target is 100 and 0 is no control.

Test Species	Treatment Timing	Rate (g/ha)
*Amaranthus retroflexus (*Dicot)	Pre/post emergence	1000
Lolium perenne (Monocot)	Pre/post emergence	1000
Stellaria media (Dicot)	Pre/post emergence	1000
Digitaria sanguinalis (Monocot)	Pre/post emergence	1000

Positive controls: Acetochlor, Atrazine, Mesotrione, Pinoxaden and Glyphosate were used as positive controls for the test.

Supplementary Table 2. Herbicide Glasshouse screen (GH2):

The compounds were tested for pre- and post-emergence activity against eight weed species, with the compounds applied at 1000g/ha. The plants were then placed in the glasshouse for 12 days. The weeds tested are listed in the table below. Assessments were made of % phytotoxicity where complete control of the target is 100 and 0 is no control.

Test Species	Treatment Timing	Rate (g/ha)
Abutilon theophrasti (Dicot)	Pre/post emergence	1000
Bidens pilosa (Dicot)	Pre/post emergence	1000
Chenopodium album (Dicot)	Pre/post emergence	1000

Kochia scoparia (Dicot)	Pre/post emergence	1000
Echinochloa crus-galli (Monocot)	Pre/post emergence	1000
Setaria faberi (Monocot)	Pre/post emergence	1000
Eleusine indica (Monocot)	Pre/post emergence	1000
Sorghum halepense (Monocot)	Pre/post emergence	1000

Positive controls: Atrazine, Mesotrione, Pinoxaden and Glyphosate were used as positive controls for the test.

General Experimental Procedures

General Procedure A: Swern Oxidation.

For example, synthesis of aldehyde **7**.

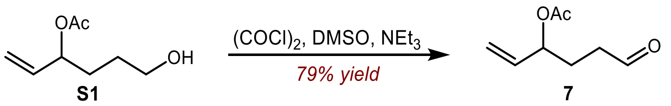

To a three-necked flask under an atmosphere of nitrogen was added oxalyl chloride (3.32 mL, 39.23 mmol, 1.5 equiv.) and anhydrous CH_2Cl_2 (90 mL). The reaction was cooled to −78 °C and DMSO (5.60 mL, 78.84 mmol, 3 equiv.) added dropwise. The reaction was stirred for 15 minutes at −78 °C before a solution of alcohol **S1** (4.15 g, 26.24 mmol, 1 equiv.) in CH_2Cl_2 (10 mL) was added dropwise. The reaction was stirred at −78 °C for a further 30 minutes before being quenched slowly with triethylamine (22 mL, 157.84 mmol, 5 equiv.). The reaction was allowed to warm to room temperature over 1 h. The pale orange suspension was then diluted with water (40 mL) and extracted with CH_2Cl_2 (3 x 30 mL). The organics were combined, washed with brine (20 mL), dried over Na_2SO_4, filtered, and evaporated to afford a pale orange liquid. The crude material was loaded directly in a solution of CH_2Cl_2 and purified by flash silica column chromatography, eluent 10-20% EtOAc/petroleum ether to afford the title compound as a pale yellow liquid (3.26 g, 79%).

General Procedure B: Aldol addition.

For example, synthesis of compound ***syn*-9**.

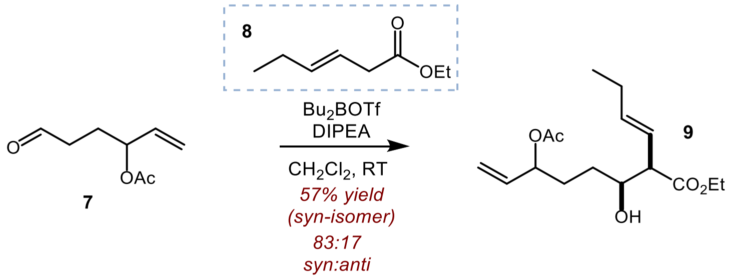

To a three-necked flask at room temperature under an atmosphere of nitrogen was added ester **8** (2.72 mL, 17.12 mmol, 1.3 equiv.) in anhydrous CH_2Cl_2 (50 mL) and DIPEA (3.44 mL, 19.75 mmol, 1.5 equiv.). Dibutylboryltrifluoromethanesulfonate solution (1 M in CH_2Cl_2) (17.1 mL, 17.1 mmol, 1.3 equiv.) was added dropwise and the resulting solution stirred at room temperature for 30 minutes. A solution of aldehyde **7** (2.06 g, 13.16 mmol, 1 equiv.) in CH_2Cl_2 (10 mL) was then added dropwise and the reaction stirred at room temperature for 1 h. The reaction was quenched with a potassium buffer solution (pH 7.4, 26 mL), MeOH (40 mL), and H_2O_2 (30% solution, 13 mL) which were added sequentially. A small exotherm was observed on H_2O_2 addition. The reaction was stirred vigorously at room temperature for 16 h, diluted with water (30 mL), and extracted with CH_2Cl_2 (3 x 40 mL). The organics were combined, washed with brine (30 mL), dried over Na_2SO_4, filtered, and evaporated to afford a pale yellow oil. The crude material loaded directly in a solution of CH_2Cl_2 and purified by flash silica column chromatography, eluent 20% EtOAc/petroleum ether to afford the title compound as a colourless liquid. 68% yield, 83:17 *syn:anti* by ^{1}H NMR; 57%, 2.81 g of the *syn*-isomer.

General Procedure C: Tandem dehydration/Diels-Alder followed by ester hydrolysis.

For example, synthesis of compound **11**.

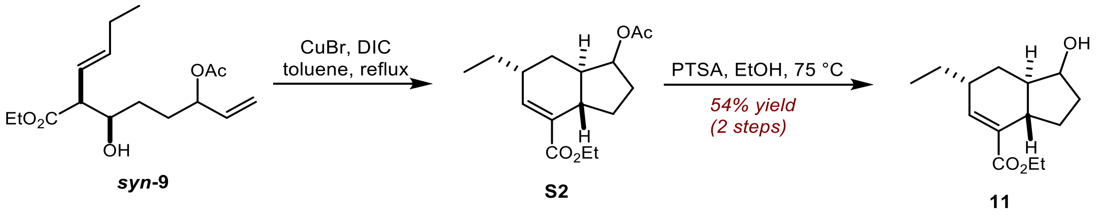

To a round bottom flask under an atmosphere of nitrogen was added compound ***syn*-9** (2.00 g, 6.71 mmol, 1 equiv. (79% purity)), CuBr (96 mg, 0.67 mmol, 10 mol%), and anhydrous toluene (1.3 mL). DIC (1.56 mL, 10.07 mmol, 1.5 equiv.) was added in one portion and the resulting solution was brought to 110 °C for 16 h. The reaction was allowed to cool to room temperature and the crude solution was filtered through celite, eluting with EtOAc (30 mL). The organics were washed with water (30 mL), followed by brine (30 mL), dried over Na_2SO_4, filtered, and evaporated to afford a pale brown oil. The crude material was directly loaded in a solution of 10% EtOAc/petroleum ether and purified by flash silica column chromatography, eluent 10% EtOAc/petroleum ether to afford a pale yellow oil (**S2**) (1.49 g, 5.32 mmol) which was not characterised.

To the pale yellow oil was added EtOH (50 mL) and PTSA (mono-hydrate) (1.52 g, 7.99 mmol, 1.5 equiv.) and the resulting solution was brought to 75 °C for 5 h. The reaction was allowed to cool to room temperature and the solvent evaporated to afford an orange oil. The crude material was directly loaded in a solution of 20% EtOAc/petroleum ether and minimal CH_2Cl_2 and purified by flash silica column chromatography, eluent 20% EtOAc/petroleum ether to afford the title compound as a colourless liquid (677 mg, 54% (2 steps)).

General Procedure D: DMP oxidation.

For examples, synthesis of compound **S3**.

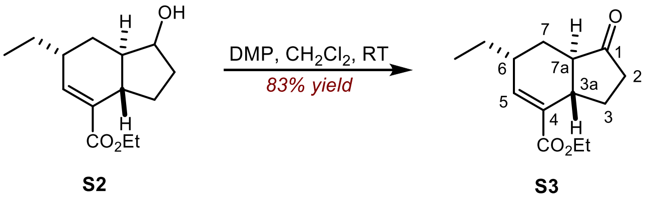

To a round bottom flask charged with compound **S2** (300 mg, 1.25 mmol, 1 equiv.) in anhydrous CH_2Cl_2 (12 mL) was added DMP (794 mg, 1.86 mmol, 1.5 equiv.) in one portion under an atmosphere of nitrogen. The reaction was stirred at room temperature for 16 h before 2 M NaOH (10 mL) was added and the layers stirred vigorously for 10 minutes. The layers were separated and the aqueous further extracted with CH_2Cl_2 (2 x 20 ml). The organics were combined, washed with brine (20 mL), dried over Na_2SO_4, filtered, and evaporated to afford a colourless oil. The crude material was loaded in a solution of 10% EtOAc/petroleum ether and purified by flash silica column chromatography, eluent 10% EtOAc/petroleum ether to afford the title compound as a colourless oil (245 mg, 83%).

General Procedure E: Acidic ester hydrolysis.

For example, see synthesis of (±)-coronafacic acid, **(±)-2**.

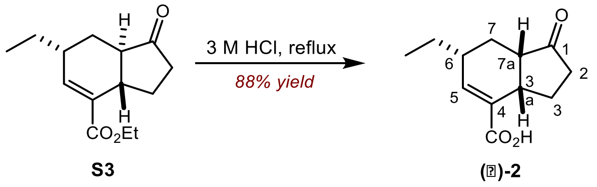

To a round bottom flask was added compound **S3** (1.10 g, 4.65 mmol) and 3 M HCl (150 mL). The reaction was brought to 100 °C and maintained at this temperature with stirring for 16 h. The reaction was allowed to cool to room temperature and extracted with EtOAc (3 x 30 mL). The organics were combined, washed with brine (30 mL), dried over Na_2SO_4, filtered, and evaporated to afford an orange oil. The crude material was loaded directly in a solution of 30% EtOAc/petroleum ether and purified by flash silica column chromatography, eluent 30-60% EtOAc/petroleum ether to afford the title compound as a white solid (850 mg, 88%).

General Procedure F: Synthesis of (±)-CFA-amino acid methyl ester analogues (Figure 3).

For example, synthesis of compound **S4**.

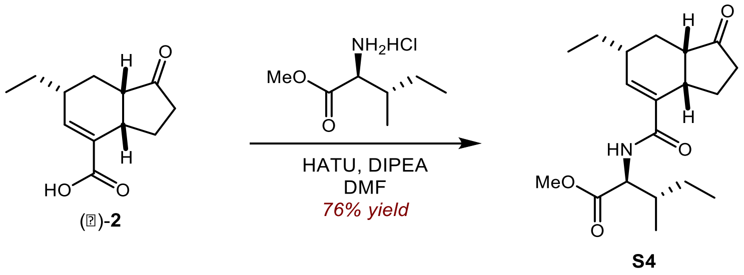

To a 2-dram vial was added (±)-CFA (**2**) (30 mg, 0.14 mmol, 1 equiv.) and HATU (66 mg, 0.17 mmol, 1.2 equiv.). DMF (0.7 mL) was added, followed by DIPEA (80 µL, 0.46 mmol, 3 equiv.) and the resulting solution stirred at room temperature for 5 minutes. Methyl *L*-isoleucinate hydrochloride (30 mg, 0.21 mmol, 1.5 equiv.) was then added in one portion and the vial capped with a screw top lid. The reaction was stirred for 16 h. The reaction was then diluted with H_2O (10 mL) and the organics extracted with EtOAc (3 x 5 mL). The organics were combined, washed with brine (10 mL), dried over Na_2SO_4, filtered, and evaporated to afford a pale yellow oil. The crude material was loaded directly in a solution of CH_2Cl_2 and purified by flash silica column chromatography, eluent 30% EtOAc/CH_2Cl_2 to afford the desired product as a colourless oil which solidified to a white solid on standing (35 mg, 76%).

General Procedure G: Pro-cide ester hydrolysis (Figure 3).

For example, synthesis of compound **16**.

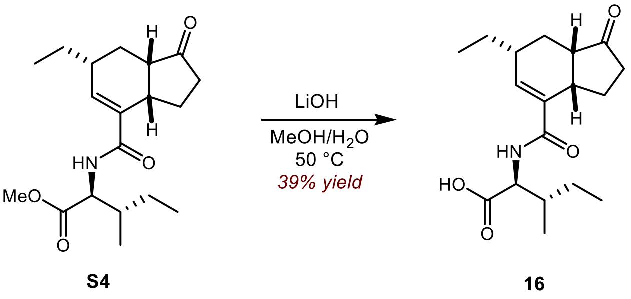

To a round bottom flask was added compound **S4** (24 mg, 0.07 mmol, 1 equiv.) and LiOH (5 mg, 0.20 mmol, 3 equiv.). The material was suspended in 1:1 MeOH:H_2O (3 mL) and the resulting suspension brought to 50 °C for 16 h. The reaction was

allowed to cool to room temperature, and extracted with EtOAc (1 x 5 mL), and the organics discarded. The aqueous phase was acidified with HCl (aq.), and extracted with EtOAc (3 x 10 mL). The organics were combined, dried over Na_2SO_4, filtered, and evaporated to afford a colourless oil. The crude material was taken up in a minimal volume of diethyl ether, and petroleum ether added until a white precipitate formed (where precipitation did not occur spontaneously the solvent was concentrated under a stream of compressed air until precipitation occurred). The solvent was removed using a Pasteur pipette and the precipitate dried under vacuum to afford the desired product as a white solid (9 mg, 39%).

General Procedure H: General procedure for coronalon core automated screen.

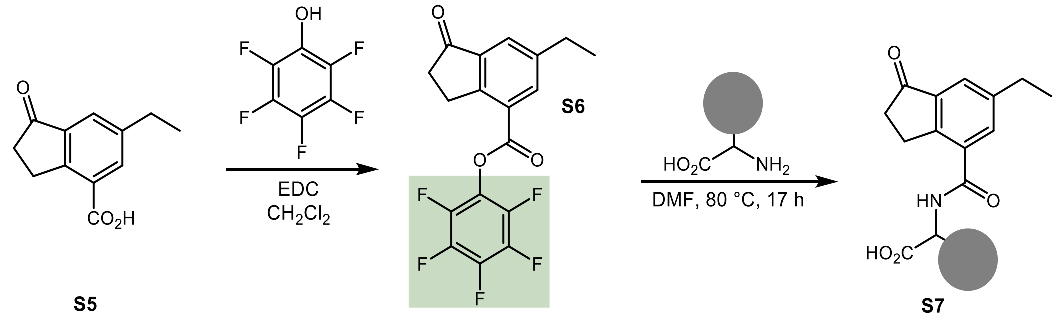

To a solution of amino acid (0.65 mmol, 1.2 equiv.) in DMF (2 mL) in a test tube was added **S5** (200 mg, 0.54 mmol, 1 equiv.) in one portion and the reaction agitated at 80 °C for 17 hours. The crude reaction was concentrated *in vacuo*, dissolved in 10% MeOH in DMSO (1 mL) with heating, filtered, and purified by mass-directed HPLC to give the title compound.

General Procedure I: General procedure for CMA automated screen.

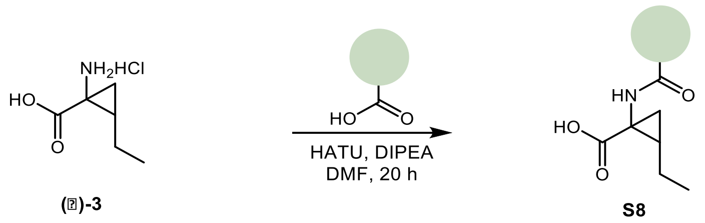

A test tube was charged with carboxylic acid (0.6 mmol, 1 equiv.) and a solution of HATU (266 mg, 0.7 mmol, 1.2 equiv.) in DMF (2 mL) added. The reaction mixture was agitated for 1 h before a solution of DIPEA (0.35 mL, 2 mmol, 3 equiv.) and compound **(±)-3** (100 mg, 0.6 mmol, 1 equiv.) in DMF (2 mL) was added and the reaction mixture agitated for 20 h. The crude reaction was concentrated *in vacuo*, dissolved in 10% MeOH in DMSO (1 mL) and purified by mass-directed HPLC to give the title compound.

Procedures and Characterization.

Synthesis of (±)-coronafacic acid (Figure 2).

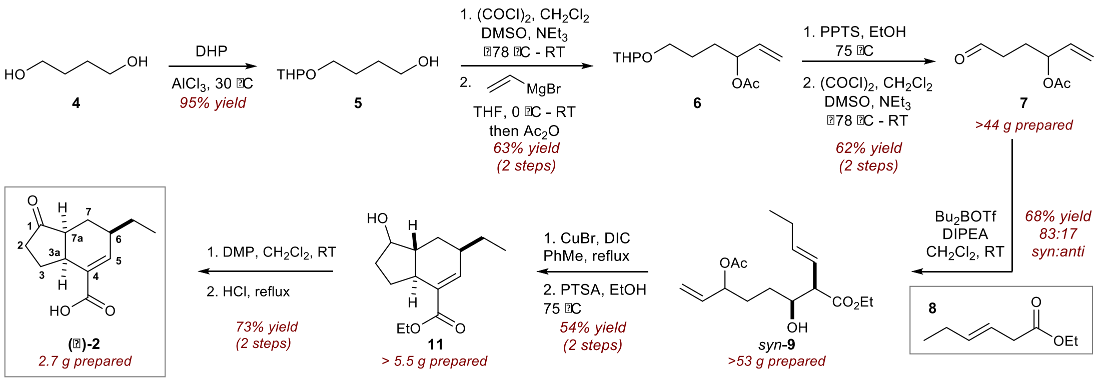

Compound 5.

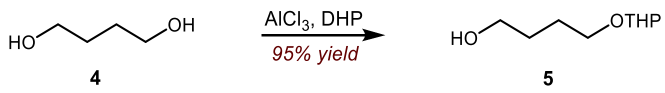

To a round bottom flask was added butane-1,4-diol (**4**) (27.3 g, 302.93 mmol, 5 equiv.) and anhydrous aluminium trichloride (79 mg, 0.59 mmol, 1 mol%). DHP (5.42 mL, 59.41 mmol, 1 equiv.) was added slowly and the resulting mixture was warmed to 30 °C for 30 minutes, before being allowed to cool to room temperature. The colourless, crude material was loaded directly in a solution of 40% EtOAc/petroleum ether and purified by flash silica column chromatography, eluent 30−60% EtOAc/petroleum ether to afford the title compound as a colourless liquid (9.86 g, 95%).

TLC (40% EtOAc/PE): R_f = 0.28 stained by $KMnO_4$.

υ_{max} (neat): 3389 (br.), 2937, 2867, 1442, 1353, 1203, 1121, 1022, 907, 870, 812 cm^{-1}.

^{1}H NMR (400 MHz, $CDCl_3$): δ 4.60 – 4.56 (m, 1H), 3.88 – 3.74 (m, 2H), 3.67 – 3.61 (m, 2H), 3.53 – 3.46 (m, 1H), 3.44 – 3.38 (m, 1H), 2.32 (br. s, 1H), 1.86 – 1.73 (m, 1H), 1.73 – 1.61 (m, 5H), 1.61 – 1.43 (m, 4H).

^{13}C NMR (126 MHz, $CDCl_3$): δ 99.1, 98.9, 67.7, 67.5, 62.9, 62.9, 62.5, 62.4, 30.9, 30.8, 30.3, 30.00, 26.7, 25.6, 25.5, 19.7, 19.7. 1:1 mixture of rotamers.

HRMS: exact mass calculated for $[M+H]^+$ ($C_9H_{19}O_4$) requires m/z 175.1329, found m/z 175.1328.

The spectral data were consistent with those previously reported in the literature.[8]

Compound 6.

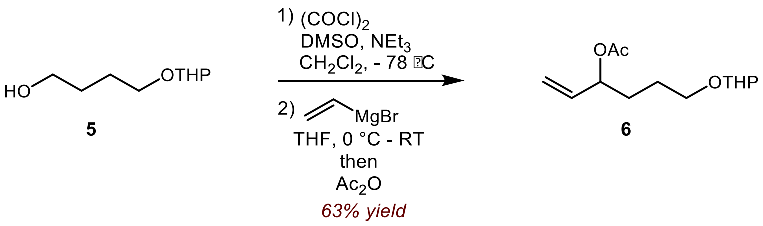

Swern oxidation carried out according to General Procedure A using oxalyl chloride (7.91 mL, 93.48 mmol, 1.5 equiv.), DMSO (13.26 mL, 186.69 mmol, 3 equiv.), compound **5** (9.81 g, 56.27 mmol, 1 equiv.), triethylamine (39.6 mL, 284.12 mmol, 5 equiv.), and CH_2Cl_2 (140 mL). The crude material was subjected to purification outlined in General Procedure A

(silica gel, 20% EtOAc/PE) to afford the corresponding aldehyde as a pale yellow liquid (7.78 g, 45.00 mmol) which was used immediately.

Vinylmagnesium bromide (1 M in THF, 45 mL, 45.00 mmol, 1 equiv.) was added dropwise to a stirring solution of the isolated material in anhydrous THF (100 mL) at 0 °C in a three-necked flask under an atmosphere of nitrogen. The resulting solution was allowed to rise to room temperature and stirred for 1.5 h. The reaction was quenched by dropwise addition of acetic anhydride (8.5 mL, 90.09 mmol, 2 equiv.) at room temperature and stirred for a further 1.5 h. The yellow reaction mixture was diluted with water (30 mL) and extracted with EtOAc (3 x 30 mL). The organics were combined, washed with brine (20 mL), dried over Na_2SO_4, filtered and evaporated to afford a pale orange oil. The crude material was loaded directly in a solution of CH_2Cl_2 and purified by flash silica column chromatography, eluent 20% EtOAc/petroleum ether to afford the title compound as a colourless liquid (8.65 g, 63%).

TLC (20% EtOAc/petroleum ether): $R_f = 0.50$ stained by $KMnO_4$ and faintly visible under UV (short wave).

υ_{max} (neat): 2941, 2870, 1736, 1371, 1233, 1200, 1121, 1076, 1020 cm^{-1}.

^{1}H NMR (400 MHz, $CDCl_3$): δ 5.78 (ddd, *J* = 17.0, 10.5, 6.4 Hz, 1H), 5.31 – 5.14 (m, 3H), 4.57 (t, *J* = 3.5 Hz, 1H), 3.89 – 3.82 (m, 1H), 3.78 – 3.70 (m, 1H), 3.53 – 3.46 (m, 1H), 3.43 – 3.36 (m, 1H), 2.06 (s, 3H), 1.86 – 1.77 (m, 1H), 1.77 – 1.46 (m, 9H).

^{13}C NMR (126 MHz, $CDCl_3$): δ 170.5, 136.6, 116.9, 99.0, 74.7, 74.7, 67.2, 62.5, 31.1, 30.9, 25.6, 25.6, 25.5, 21.4, 19.8.

HRMS: exact mass calculated for $[M+Na]^+$ ($C_{13}H_{22}O_4Na$) requires *m/z* 265.1410, found *m/z* 265.1410.

The spectral data were consistent with those previously reported in the literature.[9]

Compound S1.

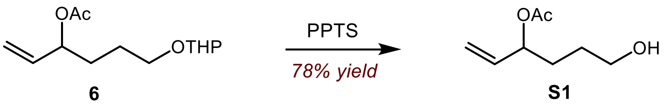

6 S1

To a round bottom flask was added compound **6** (11.51 g, 47.51 mmol, 1 equiv.) and EtOH (170 mL). PPTS (1.15 g, 4.58 mmol, 0.1 equiv.) was added portionwise and the resulting solution heated to 65 °C and maintained at this temperature for 3 h. The reaction was allowed to cool to room temperature and was then evaporated onto silica gel and purified by flash silica column chromatography, eluent 40% EtOAc/petroleum ether to afford the title compound as a colourless liquid (5.87 g, 78%).

TLC (40% EtOAc/petroleum ether): $R_f = 0.40$ stained by $KMnO_4$.

υ_{max} (neat): 3402 (br.), 2943, 2870, 1732, 1374, 1236, 1020, 968, 927 cm^{-1}.

^{1}H NMR (500 MHz, $CDCl_3$): δ 5.77 (ddd, *J* = 17.1, 10.5, 6.4 Hz, 1H), 5.29 – 5.14 (m, 3H), 3.65 (t, *J* = 6.4 Hz, 2H), 2.06 (s, 3H), 1.73 – 1.67 (m, 2H), 1.63 – 1.55 (m, 2H). O*H* not observed.

^{13}C NMR (126 MHz, $CDCl_3$): δ 170.6, 136.4, 117.0, 74.6, 62.5, 30.6, 28.3, 21.3.

HRMS: exact mass calculated for $[M+NH_4]^+$ ($C_8H_{18}O_3N$) requires *m/z* 176.1281, found *m/z* 176.1281.

The spectral data were consistent with those previously reported in the literature.[9]

Compound 7.

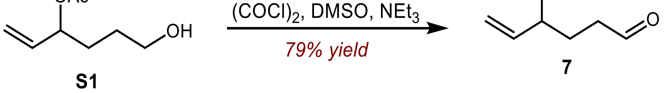

S1 7

Prepared according to General Procedure A using oxalyl chloride (3.32 mL, 39.23 mmol, 1.5 equiv.), DMSO (5.60 mL, 78.84 mmol, 3 equiv.), compound **S1** (4.15 g, 26.24 mmol, 1 equiv.), triethylamine (22 mL, 157.84 mmol, 5 equiv.), and CH_2Cl_2

(100 mL). The crude material was subjected to purification outlined in General Procedure A (silica gel, 10-20% EtOAc/petroleum ether) to afford the corresponding aldehyde as a pale yellow liquid (3.26 g, 79%).

TLC (20% EtOAc/petroleum ether): R_f = 0.37 stained by $KMnO_4$.

υ_{max} (neat): 2931, 2830, 1722, 1372, 1231, 1021, 930 cm^{-1}.

^{1}H NMR (400 MHz, $CDCl_3$): δ 9.77 (t, *J* = 1.3 Hz, 1H), 5.80 – 5.70 (m, 1H), 5.30 – 5.18 (m, 3H), 2.53 – 2.47 (m, 2H), 2.06 (s, 3H), 2.01 – 1.94 (m, 2H).

^{13}C NMR (126 MHz, $CDCl_3$): δ 201.2, 170.3, 135.7, 117.5, 73.7, 39.6, 26.5, 21.2.

HRMS: exact mass calculated for $[M+NH_4]^+$ ($C_8H_{16}O_3N$) requires *m/z* 174.1125, found *m/z* 174.1125.

The spectral data were consistent with those previously reported in the literature.[10]

Compound *syn*-9.

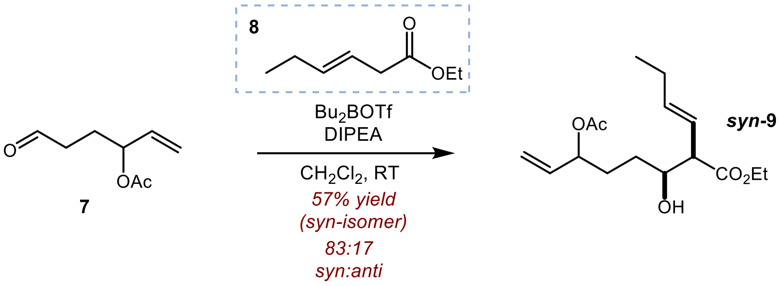

Prepared according to General Procedure B using ethyl (*E*)-hex-3-enoate (**8**) (2.72 mL, 17.12 mmol, 1.3 equiv.), DIPEA (3.44 mL, 19.75 mmol, 1.5 equiv.), dibutylboryltrifilate solution (1 M in CH_2Cl_2) (17.10 mL, 17.10 mmol, 1.3 equiv.), compound **7** (2.06 g, 13.16 mmol, 1 equiv.), CH_2Cl_2 (60 mL), potassium buffer solution (pH 7.4, 26 mL), MeOH (40 mL) and H_2O_2 (30 % solution, 13 mL). After 16 h the reaction was subjected to purification outlined in General Procedure B (silica gel, 20% EtOAc/petroleum ether) to afford the title compound as a colourless liquid (2.81 g, 57% *syn*-isomer).

Product contains 21% alkene isomerisation impurity. Data reported of products resulting from reaction carried out at – 78 °C to where isomerisation does not take place.[10]

TLC (20% EtOAc/petroleum ether): R_f = 0.31 stained by $KMnO_4$.

υ_{max} (neat): 3496 (br.), 2963, 2934, 2874, 1733, 1374, 1240, 1178, 1024, 975 cm^{-1}.

^{1}H NMR (400 MHz, $CDCl_3$): δ 5.81 – 5.66 (m, 2H), 5.51 (ddt, *J* = 15.4, 9.2, 1.5 Hz, 1H), 5.29 – 5.14 (m, 3H), 4.20 – 4.12 (m, 2H), 3.88 – 3.81 (m, 1H), 2.96 (dd, *J* = 9.2, 4.8 Hz, 1H), 2.67 (br. s, 1H), 2.13 – 2.02 (m, 5H), 1.89 – 1.78 (m, 1H), 1.72 – 1.60 (m, 1H), 1.55 – 1.35 (m, 2H), 1.26 (t, *J* = 7.1 Hz, 3H), 1.00 (td, *J* = 7.4, 0.6 Hz, 3H).

^{13}C NMR (101 MHz, $CDCl_3$): δ 173.9, 173.9, 170.5, 139.0, 136.5, 136.4, 122.1, 117.0, 116.9, 74.9, 74.5, 71.3, 71.1, 61.0, 55.0, 54.9, 30.4, 30.3, 29.7, 29.5, 25.8, 21.3, 21.3, 14.3, 13.6.

HRMS: exact mass calculated for $[M+H]^+$ ($C_{16}H_{27}O_5$) requires *m/z* 299.1853, found *m/z* 299.1856. Calculated for a mixture of the *syn*- and *anti*-diastereoisomers.

The spectral data were consistent with those previously reported in the literature.[10]

Compound ***anti*-9**.

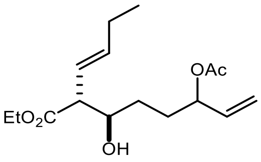

***anti*-9**

TLC (20% EtOAc/petroleum ether): R_f = 0.22 stained by $KMnO_4$.

υ_{max} (neat): 3478 (br.), 2963, 2934, 1732, 1371, 1236, 1020, 970, 930 cm^{-1}.

^{1}H NMR (400 MHz, $CDCl_3$): δ 5.82 – 5.64 (m, 2H), 5.44 – 5.35 (m, 1H), 5.29 – 5.14 (m, 3H), 4.21 – 4.13 (m, 2H), 3.83 – 3.76 (m, 1H), 3.04 – 2.97 (m, 1H), 2.55 (br. s, 1H), 2.10 – 2.01 (m, 5H), 1.91 – 1.80 (m, 1H), 1.74 – 1.52 (m, 2H), 1.43 – 1.30 (m, 1H), 1.26 (t, J = 7.1 Hz, 3H), 0.98 (td, J = 7.4, 0.7 Hz, 3H).

^{13}C NMR (101 MHz, $CDCl_3$): δ 173.8, 170.5, 170.4, 137.7, 136.5, 136.4, 123.4, 117.0, 116.8, 74.9, 74.5, 72.4, 72.2, 60.9, 56.0, 55.9, 30.3, 30.2, 30.1, 29.9, 25.7, 21.3, 21.3, 14.3, 13.5.

HRMS: exact mass calculated for $[M+H]^+$ ($C_{16}H_{27}O_5$) requires *m/z* 299.1853, found *m/z* 299.1856. Calculated for a mixture of the *syn*- and *anti*-diastereoisomers.

The spectral data were consistent with those previously reported in the literature.[10]

Compound 11.

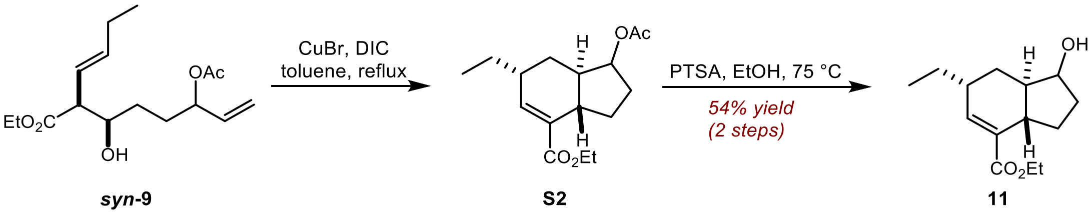

Compound **S2** was prepared according to General Procedure C using compound ***syn*-9** (2.00 g, 6.71 mmol, 1 equiv. (79% purity)), CuBr (96 mg, 0.67 mmol, 10 mol%), DIC (1.56 mL, 10.07 mmol, 1.5 equiv.) and toluene (1.3 mL). After 16 h the reaction was subjected to purification outlined in General Procedure C (silica gel, 10% EtOAc/petroleum ether) to afford a pale yellow oil (**S2**) (1.49 g, 5.32 mmol).

Compound **11** was prepared according to General Procedure C using compound **S2** (1.49 g, 5.32 mmol, 1 equiv.), PTSA (mono-hydrate) (1.52 g, 7.99 mmol, 1.5 equiv.), and EtOH (50 mL). After 5 h the reaction was subjected to purification outlined in General Procedure C (silica gel, 20% EtOAc/petroleum ether) to afford the title compound as a colourless liquid (677 mg, 54% (2 steps, based on 79% purity of starting material)).

TLC (20% EtOAc/petroleum ether): R_f = 0.16 stained by $KMnO_4$.

υ_{max} (neat): 3434 (br.), 2958, 2928, 2870, 1708, 1693, 1266, 1230, 1098, 1024 cm^{-1}.

^{1}H NMR (400 MHz, $CDCl_3$): δ 6.85 – 6.79 (m, 1H), 4.28 – 4.07 (m, 2.5H), 3.92 – 3.84 (m, 0.5H), 2.56 – 1.98 (m, 4.5H), 1.92 (d, J = 10.0 Hz, 0.5H), 1.74 – 1.32 (m, 6H), 1.32 – 1.25 (m, 3H), 1.23 – 1.12 (m, 1H), 1.02 – 0.95 (m, 3H). Mixture of isomers.

^{13}C NMR (101 MHz, $CDCl_3$): δ 167.7, 167.4, 144.0, 143.6, 143.0, 134.2, 133.4, 79.4, 76.1, 73.5, 60.4, 60.3, 48.0, 47.6, 46.0, 42.5, 40.8, 39.4, 38.8, 38.6, 38.2, 36.7, 35.1, 33.5, 33.3, 30.4, 29.3, 28.7, 28.5, 28.4, 28.3, 27.3, 26.0, 24.0, 14.5, 12.7, 11.4. Mixture of isomers, peaks reported as observed.

HRMS: exact mass calculated for $[M+H]^+$ ($C_{14}H_{23}O_3$) requires *m/z* 239.1642, found *m/z* 239.1641.

Compound S3.

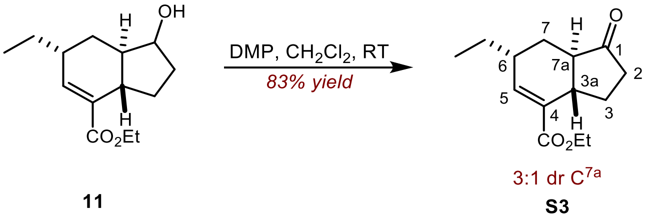

Compound **S3** was prepared according to General Procedure D using compound **11** (300 mg, 1.25 mmol, 1 equiv.), DMP (794 mg, 1.87 mmol, 1.5 equiv.) and CH_2Cl_2 (12 mL). After 16 h the reaction was subjected to purification outlined in

General Procedure D (silica gel, 10% EtOAc/petroleum ether) to afford the title compound as a colourless oil (245 mg, 83% (3:1 dr C^{7a})).

TLC (30% EtOAc/petroleum ether): R_f = 0.72 stained by $KMnO_4$.

υ_{max} (neat): 2960, 2928, 2872, 1742, 1705, 1258, 1232, 1216, 1095 cm^{-1}.

Major *anti*-isomer:

^{1}H NMR (400 MHz, $CDCl_3$): δ 6.92 – 6.87 (m, 1H), 4.30 – 4.14 (m, 2H), 2.75 – 2.66 (m, 1H), 2.51 – 2.33 (m, 3H), 2.29 – 2.17 (m, 1H), 2.05 – 1.86 (m, 2H), 1.64 – 1.44 (m, 4H), 1.31 (t, *J* = 7.1 Hz, 3H), 1.00 (t, *J* = 7.5 Hz, 3H).

Major *anti*-isomer:

^{13}C NMR (101 MHz, $CDCl_3$): δ 216.9, 166.7, 145.4, 133.0, 60.5, 51.1, 41.1, 38.5, 38.4, 28.3, 26.2, 24.8, 14.5, 12.5.

HRMS: exact mass calculated for $[M+H]^+$ ($C_{14}H_{21}O_3$) requires *m/z* 237.1485, found *m/z* 237.1487.

Compound S9.

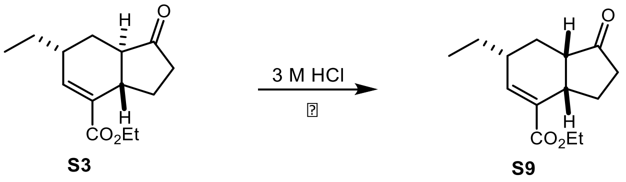

To a round bottom flask was added compound **S3** (245 mg, 1.04 mmol) and 3 M HCl (36 mL) and the resulting suspension brought to 60 °C for 16 h. The reaction was allowed to cool to room temperature and the organics extracted with EtOAc (3 x 10 mL). The organics were combined, washed with brine (10 mL), dried over Na_2SO_4, filtered, and evaporated to afford a colourless oil. The crude material was loaded directly in a solution of CH_2Cl_2 and purified by flash silica column chromatography, eluent 10% EtOAc/petroleum ether to afford the title compound as a colourless oil (186 mg, 76%).

TLC (30% EtOAc/petroleum ether): R_f = 0.72 stained by $KMnO_4$.

υ_{max} (film): 2961, 2932, 2876, 2859, 1742, 1706, 1244, 1097, 920, 753 cm^{-1}.

^{1}H NMR (400 MHz, $CDCl_3$): δ 6.90 (s, 1H), 4.29 – 4.14 (m, 2H), 3.11 – 3.02 (m, 1H), 2.59 – 2.51 (m, 1H), 2.43 – 2.22 (m, 3H), 2.22 – 2.12 (m, 1H), 1.84 (dt, *J* = 12.9, 4.8 Hz, 1H), 1.62 – 1.45 (m, 2H), 1.43 – 1.36 (m, 1H), 1.30 (t, *J* = 7.1 Hz, 3H), 1.11 – 1.01 (m, 1H), 0.97 (t, *J* = 7.5 Hz, 3H).

^{13}C NMR (101 MHz, $CDCl_3$): δ 166.9, 144.0, 131.7, 60.6, 46.8, 38.3, 37.8, 36.4, 28.3, 28.0, 26.0, 14.4, 11.3. Carbonyl *C*O not observed.

HRMS: exact mass calculated for $[M+H]^+$ ($C_{14}H_{21}O_3$) requires *m/z* 237.1485, found *m/z* 237.1487.

The spectral data were consistent with those previously reported in the literature.[10]

(±)-coronafacic acid, (±)-2.

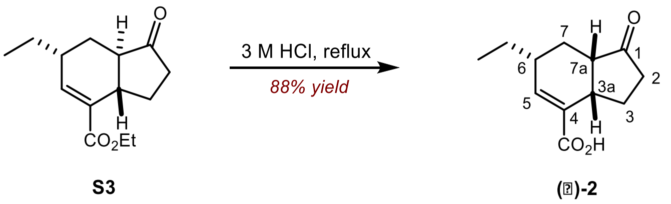

Prepared according to General Procedure E using compound **S3** (1.10 g, 4.65 mmol) and 3 M HCl (150 mL). After 16 h the reaction was subjected to purification outlined in General Procedure E (silica gel, 30-60% EtOAc/petroleum ether) to afford a white solid, which was washed with minimal petroleum ether to afford the title compound as a white solid (850 mg, 88%).

TLC (30% EtOAc/petroleum ether): R_f = 0.21 stained by $KMnO_4$.

υ_{max} (neat): 2954 (br.), 2930 (br.), 2855, 2629, 2525, 1732, 1673, 1625, 1428, 1270, 1139, 1069, 926, 727 cm^{-1}.

^{1}H NMR (400 MHz, $CDCl_3$): δ 7.08 (s, 1H, H^5), 3.13 – 3.04 (m, 1H, H^{3a}), 2.66 – 2.56 (m, 1H, H^3), 2.47 – 2.28 (m, 3H, H^{7a}, H^2), 2.28 – 2.19 (m, 1H, H^6), 1.89 (dt, J = 12.9, 4.8 Hz, 1H, H^7), 1.67 – 1.39 (m, 3H, $H^{3'}$, CH_3CH_2), 1.14 – 1.04 (m, 1H, $H^{7'}$), 0.99 (t, J = 7.4 Hz, 3H, CH_2CH_3).

^{13}C NMR (101 MHz, $CDCl_3$) δ 220.3 (C^1), 171.3 (CO_2H), 147.0 (C^5), 130.9 (C^4), 46.7 (C^{7a}), 38.3 ($C^{2/6}$), 38.0 ($C^{2/6}$), 36.2 (C^{3a}), 28.2 (CH_3CH_2), 27.9 (C^3), 25.9 (C^7), 11.3 (CH_2CH_3).

HRMS: exact mass calculated for $[M-H]^-$ ($C_{12}H_{15}O_3$) requires m/z 207.1027, found m/z 207.1030.

The spectral data were consistent with those previously reported in the literature.[10]

Synthesis of Compound S18.

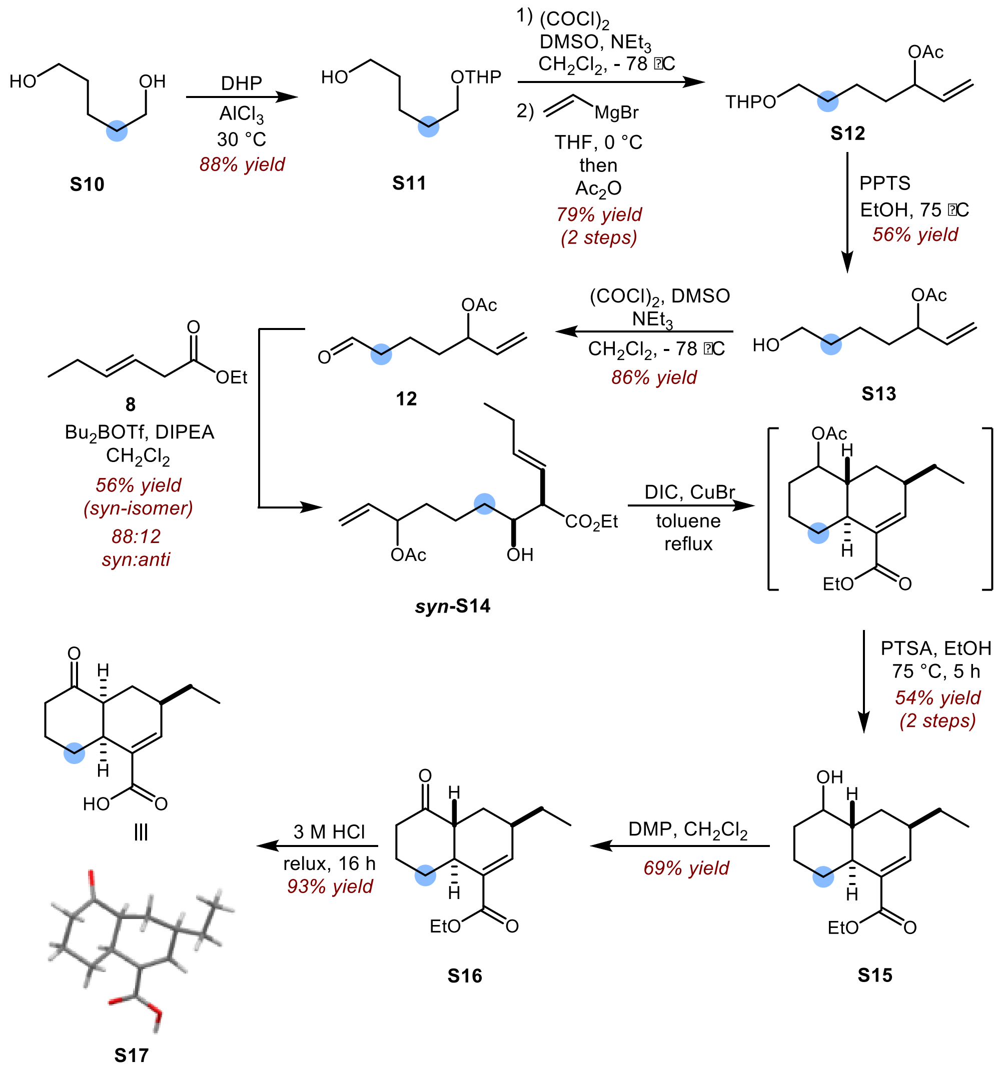

Compound S11.

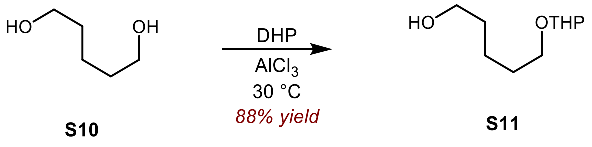

To a round bottom flask charged with 1,5-pentane diol (**S10**) (31 g, 295.86 mmol, 5 equiv.) was added anhydrous aluminium trichloride (79 mg, 0.59 mmol, 1 mol%) followed by dropwise addition of DHP (5.42 mL, 59.41 mmol, 1 equiv.). The

resulting mixture was warmed to 30 °C and maintained at this temperature for 1 h, before being allowed to cool to room temperature. The colourless, crude material was loaded directly in a solution of 30% EtOAc/petroleum ether and purified by flash silica column chromatography, eluent 30-60% EtOAc/petroleum ether to afford the title compound as a colourless liquid (9.90 g, 88%).

TLC (40% EtOAc/petroleum ether): R_f = 0.57 stained by $KMnO_4$

υ_{max} (neat): 3404 (br.), 2936, 2865, 1137, 1120, 1076, 1021 cm^{-1}.

^{1}H NMR (500 MHz, $CDCl_3$): δ 4.55 – 4.52 (m, 1H), 3.86 – 3.79 (m, 1H), 3.71 (dt, *J* = 9.6, 6.7 Hz, 1H), 3.59 (t, *J* = 6.6 Hz, 2H), 3.49 – 3.43 (m, 1H), 3.36 (dt, *J* = 9.6, 6.5 Hz, 1H), 2.05 (br. s, 1H), 1.83 – 1.74 (m, 1H), 1.71 – 1.63 (m, 1H), 1.63 – 1.45 (m, 8H), 1.44 – 1.37 (m, 2H).

^{13}C NMR (126 MHz, $CDCl_3$): δ 99.0, 67.6, 62.7, 62.4, 32.6, 30.8, 29.5, 25.5, 22.5, 19.7.

HRMS: exact mass calculated for $[M+Na]^+$ ($C_{10}H_{20}O_3Na$) requires *m/z* 211.1305, found *m/z* 211.1302.

The spectral data were consistent with those previously reported in the literature.[11]

Compound S12.

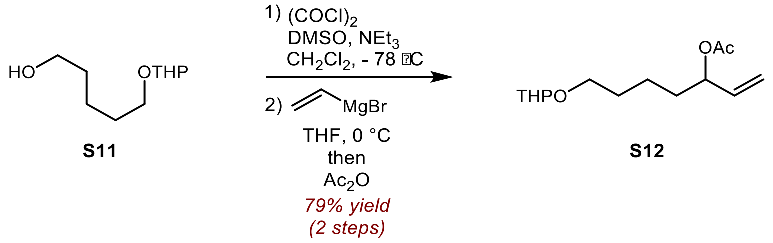

Compound **S12** was prepared according to General Procedure A using oxalyl chloride (6.68 mL, 78.95 mmol, 1.5 equiv.), DMSO (11.21 mL, 157.83 mmol, 3 equiv.), compound **S11** (9.90 g, 52.58 mmol, 1 equiv.), triethylamine (29 mL, 208.06 mmol, 4 equiv.), and CH_2Cl_2 (140 mL). After 2 h the reaction was subjected to purification outlined in General Procedure A (silica gel, 20% EtOAc/petroleum ether) to afford the corresponding aldehyde as a pale yellow liquid (9.55 g, 51.28 mmol) which was used immediately.

Vinylmagnesium bromide (1 M in THF, 56.4 mL, 56.40 mmol, 1.1 equiv.) was added dropwise to a stirring solution of the aldehyde (9.55 g, 51.28 mmol) in THF (100 mL) at 0 °C in a three-necked flask under an atmosphere of nitrogen. The resulting solution was allowed to warm to room temperature and stirred for 2 h. The reaction was quenched by dropwise addition of acetic anhydride (9.7 mL, 102.62 mmol, 2 equiv.) at room temperature and stirred for a further 16 h. The yellow reaction was diluted with water (30 mL) and extracted with EtOAc (3 x 30 mL). The organics were combined, washed with brine (20 mL), dried over Na_2SO_4, filtered, and evaporated to afford a pale orange oil. The crude material was loaded in a solution of CH_2Cl_2, and purified by flash silica column chromatography, eluent 10% EtOAc/petroleum ether to afford the title compound (10.66 g, 79% (2 steps)) as a colourless liquid.

TLC (20% EtOAc/petroleum ether): R_f = 0.74 stained by $KMnO_4$.

υ_{max} (neat): 2940, 2870, 1736, 1370, 1236, 1120 cm^{-1}.

^{1}H NMR (500 MHz, $CDCl_3$): δ 5.77 (ddd, *J* = 17.1, 10.5, 6.3 Hz, 1H), 5.26 – 5.14 (m, 3H), 4.58 – 4.54 (m, 1H), 3.89 – 3.82 (m, 1H), 3.73 (dtd, *J* = 9.4, 6.7, 1.0 Hz, 1H), 3.53 – 3.46 (m, 1H), 3.38 (dt, *J* = 9.5, 6.7 Hz, 1H), 2.06 (s, 3H), 1.86 – 1.76 (m, 1H), 1.74 – 1.48 (m, 9H), 1.46 – 1.35 (m, 2H).

^{13}C NMR (126 MHz, $CDCl_3$): δ 170.5, 136.6, 116.8, 99.0, 74.9, 67.4, 62.5, 34.1, 30.9, 29.6, 25.6, 22.0, 21.4, 19.8.

HRMS: exact mass calculated for $[M+Na]^+$ ($C_{14}H_{24}O_4Na$) requires *m/z* 279.1567, found *m/z* 279.1563.

Compound S13.

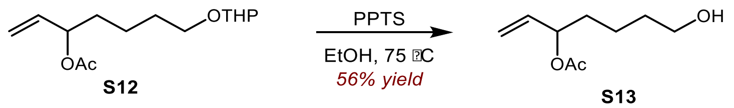

To a round bottom flask was added compound **S12** (10.66 g, 41.59 mmol, 1 equiv.) and EtOH (160 mL). PPTS (1.05 g, 4.18 mmol, 0.1 equiv.) was added portionwise and the resulting solution was brought to 60 °C for 4 h. The reaction was allowed to cool to room temperature and was then evaporated to afford a pale orange oil. The crude material was loaded directly in a solution of 30% EtOAc/petroleum ether and purified by flash silica column chromatography, eluent 30-50% EtOAc/petroleum ether to afford the title compound (4.02 g, 56%) as a colourless liquid.

TLC (30% EtOAc/petroleum ether): $R_f = 0.18$ stained by $KMnO_4$.

υ_{max} (neat): 3407 (br.), 2936, 2865, 1735, 1371, 1236, 1019 cm^{-1}.

^{1}H NMR (500 MHz, $CDCl_3$): δ 5.77 (ddd, J = 17.2, 10.5, 6.4 Hz, 1H), 5.26 – 5.15 (m, 3H), 3.64 (t, J = 6.5 Hz, 2H), 2.06 (s, 3H), 1.72 – 1.55 (m, 5H), 1.47 – 1.34 (m, 2H).

^{13}C NMR (126 MHz, $CDCl_3$): δ 170.6, 136.5, 116.9, 74.8, 62.9, 34.1, 32.5, 21.5, 21.4.

HRMS: exact mass calculated for $[M+Na]^+$ ($C_9H_{16}O_3Na$) requires *m/z* 195.0992, found *m/z* 195.0989.

Compound 12.

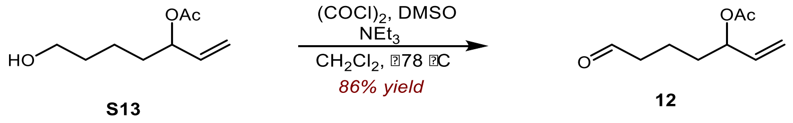

Prepared according to General Procedure A using oxalyl chloride (2.96 mL, 34.98 mmol, 1.5 equiv.), DMSO (4.97 mL, 69.97 mol, 3 equiv.), compound **S13** (4.02 g, 23.34 mmol, 1 equiv.), triethylamine (13 mL, 93.27 mmol, 4 equiv.) and CH_2Cl_2 (55 mL). After 2 h the reaction was subjected to purification outlined in General Procedure A (silica gel, 20% EtOAc/petroleum ether) to afford the title compound as a pale yellow liquid (3.40 g, 86%).

TLC (20% EtOAc/petroleum ether): $R_f = 0.41$ stained by $KMnO_4$.

υ_{max} (neat): 2935 (br.), 1734, 1372, 1238, 1022 cm^{-1}.

^{1}H NMR (500 MHz, $CDCl_3$): δ 9.76 (t, J = 1.5 Hz, 1H), 5.80 – 5.72 (m, 1H), 5.27 – 5.16 (m, 3H), 2.50 – 2.43 (m, 2H), 2.06 (s, 3H), 1.72 – 1.59 (m, 4H).

^{13}C NMR (126 MHz, $CDCl_3$): δ 202.0, 170.4, 136.2, 117.2, 74.3, 43.6, 33.6, 21.3, 17.7.

HRMS: exact mass calculated for $[M+NH_4]^+$ ($C_9H_{18}O_3N$) requires *m/z* 188.1281, found *m/z* 188.1277.

Compound 168.

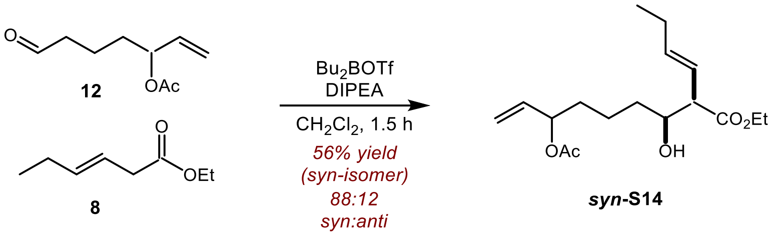

Prepared according to General Procedure B using ethyl (*E*)-hex-3-enoate (**8**) (1.38 ml, 8.70 mmol, 1.3 equiv.), DIPEA (1.73 mL, 9.93 mmol, 1.5 equiv.), Dibutylboryltrifluoromethanesulfonate solution (1 M in CH_2Cl_2) (8.74 mL, 8.74 mmol, 1.3 equiv.), compound **12** (1.15 g, 6.76 mmol, 1 equiv.), CH_2Cl_2 (35 mL), potassium buffer solution (pH 7.4, 15 mL), MeOH (25 mL), and H_2O_2 (30% solution, 8 mL). After 16 h the reaction was subjected to purification outlined in General Procedure B (silica gel, 20% EtOAc/petroleum ether) to afford the title compound as a colourless oil (1.49 g, 56% *syn*-isomer).

TLC (30% EtOAc/petroleum ether): $R_f = 0.65$ stained by $KMnO_4$.

υ_{max} (neat): 3517 (br.), 2937, 2873, 1730, 1370, 1237, 1174, 1020 cm^{-1}.

^{1}H NMR (400 MHz, $CDCl_3$): δ 5.82 – 5.65 (m, 2H), 5.55 – 5.45 (m, 1H), 5.26 – 5.13 (m, 3H), 4.20 – 4.11 (m, 2H), 3.92 – 3.80 (m, 1H), 3.35 (dd, J = 10.3, 4.3 Hz, 0.2H (minor)), 2.96 (dd, J = 9.2, 4.8 Hz, 0.8H (major)), 2.77 – 2.71 (m, 0.2H (minor)), 2.67 – 2.60 (m, 0.8H (major)), 2.15 – 2.02 (m, 5H), 1.71 – 1.31 (m, 6H), 1.29 – 1.23 (m, 3H), 1.02 – 0.97 (m, 3H).

^{13}C NMR (101 MHz, $CDCl_3$): δ 174.0, 174.0, 170.5, 138.9, 136.6, 122.2, 116.9, 116.8, 74.8, 71.4, 61.0, 55.1, 55.0, 34.2, 33.9, 25.8, 21.4, 14.3, 13.6. Major signals reported. One signal coincident.

HRMS: exact mass calculated for $[M+Na]^+$ ($C_{17}H_{28}O_5Na$) requires m/z 335.1829, found m/z 335.1827.

Compound S15.

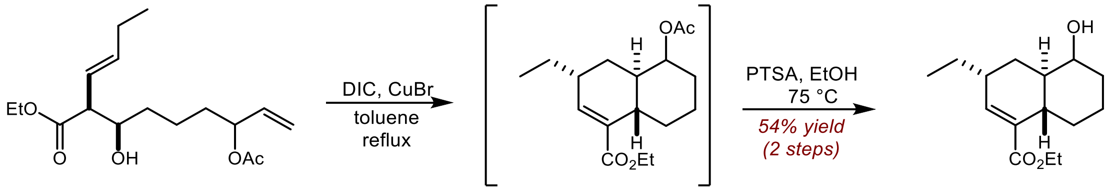

***syn*-S14** **S18** **S15**

Compound **S18** was prepared according to General Procedure C using compound ***syn*-S14** (1.49 g, 4.76 mmol, 1 equiv. (79% purity)), CuBr (74 mg, 0.52 mmol, 10 mol%), DIC (1.19 mL, 7.60 mmol, 1.5 equiv.) and toluene (0.9 mL). After 16 h the reaction was subjected to purification outlined in General Procedure C (silica gel, 5% EtOAc/petroleum ether) to afford a pale yellow oil (**S18**) (1.11 g, 3.77 mmol).

Compound **S15** was prepared according to General Procedure C using compound **S18** (1.11 g, 3.77 mmol, 1 equiv.), PTSA (mono-hydrate) (1.07 g, 5.63 mmol, 1.5 equiv.), and EtOH (35 mL). After 6 h the reaction was subjected to purification outlined in General Procedure C (silica gel, 20% EtOAc/petroleum ether) to afford the title compound as a colourless liquid (511 mg, 54% based on 79% purity of starting material (2 steps)). Isolated as a single diastereoisomer at C^1, the stereochemistry of which was not determined.

TLC (20% EtOAc/petroleum ether): $R_f = 0.19$ stained by $KMnO_4$.

υ_{max} (neat): 3377 (br.), 2972, 2932, 2662, 1711, 1447, 1245, 1045 cm^{-1}.

^{1}H NMR (400 MHz, $CDCl_3$): δ 6.72 – 6.67 (m, 1H), 4.22 – 4.08 (m, 2H), 3.36 – 3.28 (m, 1H), 2.25 – 2.12 (m, 2H), 2.11 – 1.95 (m, 3H), 1.85 – 1.70 (m, 1H), 1.59 – 1.11 (m, 10H), 0.98 (t, J = 7.4 Hz, 3H), 0.88 – 0.76 (m, 1H).

^{13}C NMR (101 MHz, $CDCl_3$): δ 168.3, 142.0, 134.3, 73.6, 60.3, 43.7, 40.2, 36.7, 36.2, 29.6, 27.5, 27.4, 24.3, 14.4, 12.6.

HRMS: exact mass calculated for $[M+H]^+$ ($C_{15}H_{25}O_3$) requires m/z 253.1798, found m/z 253.1799.

Compound S16.

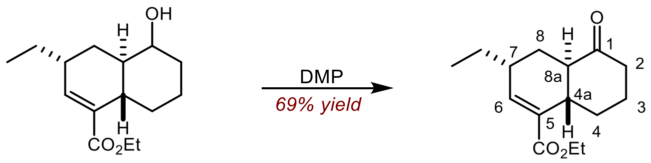

S15 **S16**

Compound **S16** was prepared according to General Procedure D using compound **S15** (511 mg, 2.02 mmol, 1 equiv.), DMP (1.29 g, 3.04 mmol, 1.5 equiv.) and CH_2Cl_2 (20 mL). After 16 h the reaction was subjected to purification outlined in General Procedure D (silica gel, 10% EtOAc/petroleum ether) to afford the title compound as a colourless oil (350 mg, 69%).

TLC (10% EtOAc/petroleum ether): R_f = 0.36 stained by $KMnO_4$.

υ_{max} (neat): 2958, 2928, 2863, 1706, 1260, 1234, 1082 cm^{-1}.

^{1}H NMR (500 MHz, $CDCl_3$): δ 6.81 (d, J = 5.2 Hz, 1H, H^6), 4.26 – 4.14 (m, 2H, $CO_2CH_2CH_3$), 2.53 (dd, J = 13.0, 3.0 Hz, 1H, H^4), 2.48 – 2.32 (m, 3H, H^2, H^{4a}), 2.30 – 2.20 (m, 2H, H^7, H^{8a}), 2.16 – 2.07 (m, 1H, H^3), 1.95 (d, J = 13.9 Hz, 1H, H^8), 1.84 – 1.72 (m, 1H, $H^{3'}$), 1.55 – 1.43 (m, 2H, $H^{8'}$, CH_3CH_2), 1.37 – 1.25 (m, 5H, H4', CH_3CH_2', $CO_2CH_2CH_3$), 0.99 (t, J = 7.4 Hz, 3H, CH_2CH_3).

^{13}C NMR (101 MHz, $CDCl_3$): δ 211.9 (C^1), 167.5 (CO_2Et), 142.9 (C^6), 133.7 (C^5), 60.5 ($CO_2CH_2CH_3$), 48.4 ($C^{7/8a}$), 42.8 (C^{4a}), 41.6 (C^2), 36.4 ($C^{7/8a}$), 29.7 (C^4), 27.6 (CH_2CH_3), 26.1 (C^3), 24.3 (C^8), 14.4 ($CO_2CH_2CH_3$), 12.5 (CH_2CH_3).

HRMS: exact mass calculated for $[M+H]^+$ ($C_{15}H_{23}O_3$) requires m/z 251.1642, found m/z 251.1647.

Compound 172.

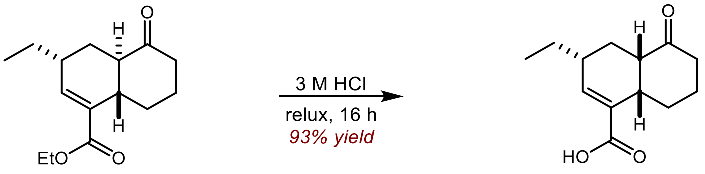

S16 **S17**

Prepared according to General Procedure E using compound **S16** (350 mg, 1.57 mmol) and 3 M HCl (44 mL). After 20 h the reaction was subjected to purification outlined in General Procedure E (silica gel, 30-50% EtOAc/petroleum ether) to afford the title compound as a pale orange solid (288 mg, 93%).

TLC (30% EtOAc/petroleum ether): R_f = 0.08 stained by $KMnO_4$.

m.p.: 114-116 °C. Crystallised by vapour diffusion (EtOAc/petroleum ether).

υ_{max} (neat): 2936, 2872, 2635, 2524, 1701, 1676, 1634, 1281cm^{-1}.

^{1}H NMR (400 MHz, $CDCl_3$): δ 11.51 (br. s, 1H), 7.05 (s, 1H), 2.91 – 2.80 (m, 1H), 2.57 – 2.46 (m, 1H), 2.45 – 2.24 (m, 3H), 2.20 – 2.08 (m, 1H), 2.06 – 1.94 (m, 1H), 1.81 – 1.61 (m, 2H), 1.61 – 1.36 (m, 4H), 0.97 (t, J = 7.4 Hz, 3H).

^{13}C NMR (101 MHz, $CDCl_3$): δ 214.4, 172.2, 146.6, 132.2, 50.1, 38.6, 38.5, 36.6, 27.9, 27.6, 27.5, 24.9, 11.3.

HRMS: exact mass calculated for $[M-H]^-$ ($C_{13}H_{17}O_3$) requires m/z 221.1183, found m/z 211.1185.

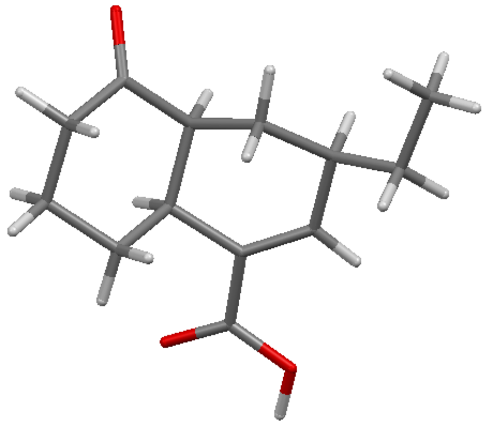

Supplementary Table 3. Crystal data and structure refinement for watson_ml66.

Identification code	watson_ml66	
Empirical formula	C13 H18 O3	
Formula weight	222.27	
Temperature	150(2) K	
Wavelength	0.71073 Å	
Crystal system	Triclinic	
Space group	P-1	
Unit cell dimensions	a = 6.8036(16) Å	α= 102.133(19)°.
	b = 9.467(2) Å	β= 106.551(19)°.
	c = 10.746(2) Å	γ = 110.11(2)°.
Volume	585.3(2) $Å^3$	
Z	2	
Density (calculated)	1.261 Mg/m^3	
Absorption coefficient	0.088 mm^{-1}	
F(000)	240	
Crystal size	0.33 x 0.18 x 0.05 mm^3	
Theta range for data collection	3.28 to 27.00°.	
Index ranges	-8<=h<=6, -12<=k<=11, -8<=l<=13	
Reflections collected	4443	

Independent reflections	2536 [R(int) = 0.0333]
Completeness to theta = 27.00°	99.6 %
Absorption correction	Semi-empirical from equivalents
Max. and min. transmission	1.00000 and 0.97703
Refinement method	Full-matrix least-squares on F^2
Data / restraints / parameters	2536 / 0 / 150
Goodness-of-fit on F^2	1.028
Final R indices [I>2sigma(I)]	R1 = 0.0538, wR2 = 0.1143
R indices (all data)	R1 = 0.0801, wR2 = 0.1362
Largest diff. peak and hole	0.412 and -0.194 e.Å^{-3}

Supplementary Table 4. Atomic coordinates (x 10^4) and equivalent isotropic displacement parameters (Å^{2}x 10^3) for watson_ml66. U(eq) is defined as one third of the trace of the orthogonalized U^{ij} tensor.

	x	y	z	U(eq)
O(1)	5021(3)	-6083(2)	3458(2)	39(1)
O(2)	538(2)	-1556(2)	4874(1)	30(1)
O(3)	1573(2)	280(2)	3898(1)	29(1)
C(1)	4024(3)	-3967(2)	3249(2)	28(1)
C(2)	3493(4)	-5705(2)	3057(2)	29(1)
C(3)	1051(4)	-6915(2)	2334(2)	31(1)
C(4)	-495(3)	-6347(2)	2865(2)	31(1)
C(5)	-189(3)	-4731(2)	2740(2)	27(1)
C(6)	2265(3)	-3471(2)	3598(2)	24(1)
C(7)	2616(3)	-1854(2)	3486(2)	22(1)
C(8)	3933(3)	-1125(2)	2883(2)	24(1)
C(9)	5201(3)	-1820(2)	2221(2)	26(1)
C(10)	4277(4)	-3619(2)	1964(2)	29(1)
C(11)	1490(3)	-1035(2)	4143(2)	23(1)
C(12)	5166(4)	-1423(3)	915(2)	33(1)
C(13)	6655(4)	-1904(3)	277(2)	41(1)

Synthesis of Compound 180.

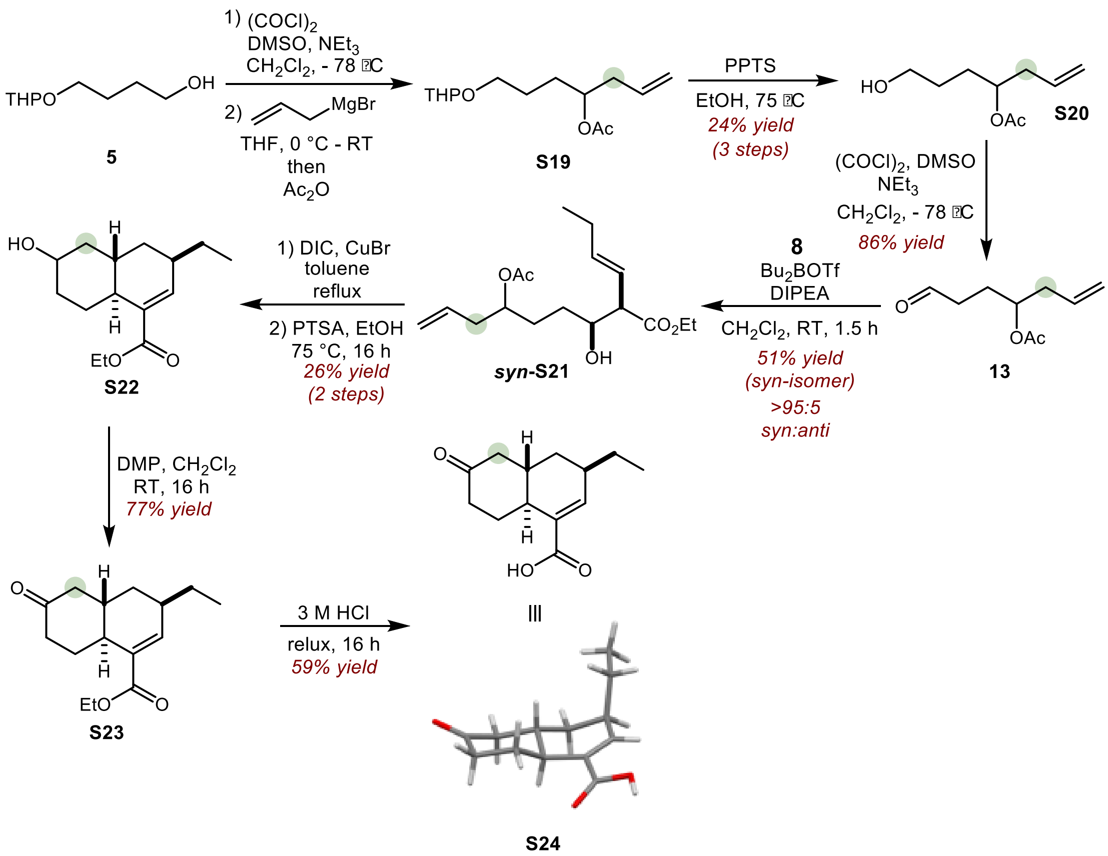

Compound S20.

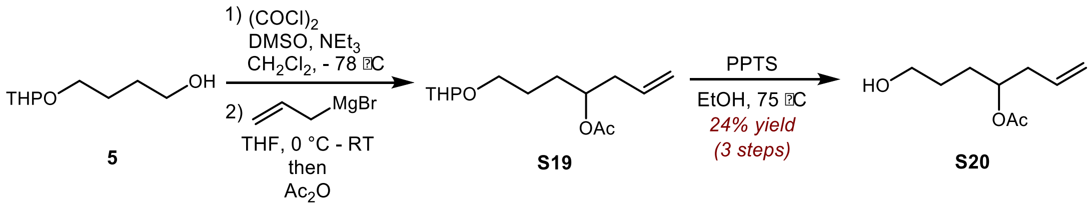

Compound **S20** was prepared according to General Procedure A using DMSO (5.82 mL, 81.94 mmol, 3 equiv.), oxalyl chloride (3.51 mL, 40.93 mmol, 1.5 equiv.), compound **5** (4.76 g, 27.32 mmol, 1 equiv.), triethylamine (15.24 mL, 109.34 mmol, 4 equiv.), and CH_2Cl_2 (55 mL). After 2 h the reaction was subjected to purification outlined in General Procedure A (silica gel, 10-30% EtOAc/petroleum ether) to afford a pale yellow oil which was dissolved in THF (50 mL) under an atmosphere of nitrogen and allylmagnesium bromide (1 M in Et_2O) (30.0 mL, 30.00 mmol, 1.1 equiv.) added dropwise at 0 °C over 5 minutes. The resulting solution was allowed to rise to room temperature and stirred for 16 h. The reaction was slowly quenched with water (70 mL) and stirred vigorously for 10 minutes. The organics were extracted with EtOAc (3 x 30 mL), washed with brine (30 mL) and dried over Na_2SO_4, filtered, and evaporated to afford a yellow oil. The crude material was loaded in a solution of CH_2Cl_2 and purified by flash silica column chromatography, eluent 40% EtOAc/petroleum ether to afford compound **S19** as a colourless oil (3.86 g, 15.06 mmol) which was used without further purification.

To a round bottom flask was added compound **S19** (3.86 g, 15.06 mmol, 1 equiv.) and EtOH (30 mL). PPTS (379 mg, 1.51 mmol, 0.1 equiv.) was added portionwise and the resulting solution was brought to 60 °C for 4 h. The reaction was allowed

to cool to room temperature and was then evaporated to afford a pale orange oil. The crude material was loaded directly in a solution of 30% EtOAc/petroleum ether and purified by flash silica column chromatography, eluent 30-50% EtOAc/petroleum ether to afford the title compound (1.13 g, 24% (3 steps)) as a colourless liquid.

TLC (60% EtOAc/petroleum ether): R_f = 0.12 stained by $KMnO_4$.

υ_{max} (neat): 3434 (br.), 2945, 2870, 1732, 1716, 1376, 1238, 1024 cm^{-1}.

^{1}**H NMR** (500 MHz, $CDCl_3$): δ 5.75 (ddt, *J* = 17.2, 10.2, 7.1 Hz, 1H), 5.11 – 5.03 (m, 2H), 4.98 – 4.90 (m, 1H), 3.65 (t, *J* = 6.2 Hz, 2H), 2.37 – 2.26 (m, 2H), 2.03 (s, 3H), 1.71 – 1.53 (m, 4H). O*H* not observed.

^{13}**C NMR** (101 MHz, $CDCl_3$): δ 171.0, 133.7, 117.9, 73.2, 62.7, 38.8, 30.1, 28.6, 21.3.

HRMS: exact mass calculated for $[M+Na]^+$ ($C_9H_{16}O_3Na$) requires *m/z* 195.0992, found *m/z* 195.0991.

Compound 13.

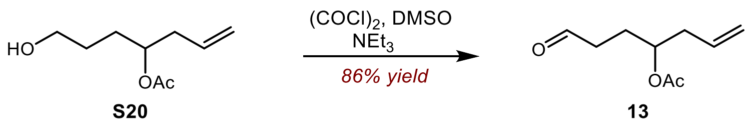

Prepared according to General Procedure A using oxalyl chloride (0.24 mL, 2.84 mmol, 1.5 equiv.), DMSO (0.40 mL, 5.63 mmol, 3 equiv.), compound **S20** (324 mg, 1.88 mmol, 1 equiv.), triethylamine (1.05 mL, 7.53 mmol, 4 equiv.) and CH_2Cl_2 (5 mL). After 2 h the reaction was subjected to purification outlined in General Procedure A (silica gel, 40% EtOAc/petroleum ether) to afford the title compound as a pale yellow oil (275 mg, 86%).

TLC (40% Et_2O/petroleum ether): R_f = 0.50 stained by $KMnO_4$.

υ_{max} (neat): 3366, 2963, 1727, 1374, 1234, 1020, 916 cm^{-1}.

^{1}**H NMR** (400 MHz, $CDCl_3$): δ 9.75 (t, *J* = 1.3 Hz, 1H), 5.73 (ddt, *J* = 17.3, 10.3, 7.1 Hz, 1H), 5.13 – 5.05 (m, 2H), 4.96 – 4.88 (m, 1H), 2.48 (td, *J* = 7.4, 1.3 Hz, 2H), 2.35 – 2.29 (m, 2H), 2.03 (s, 3H), 2.00 – 1.90 (m, 1H), 1.90 – 1.79 (m, 1H).

^{13}**C NMR** (101 MHz, $CDCl_3$): δ 201.5, 170.8, 133.2, 118.4, 72.5, 40.1, 38.8, 26.0, 21.2.

HRMS: exact mass calculated for $[M+NH_4]^+$ ($C_9H_{18}O_3N$) requires *m/z* 188.1281, found *m/z* 188.1281.

Compound *syn*-S21.

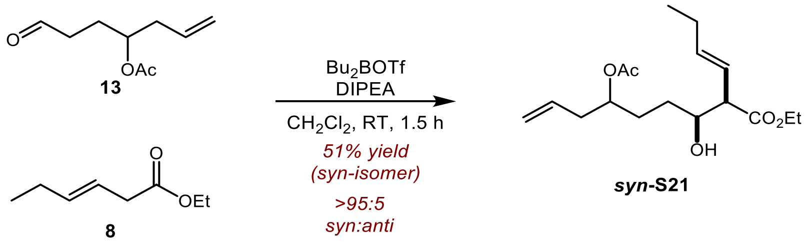

Prepared according to General Procedure B using ethyl (*E*)-hex-3-enoate (**8**) (1.21 mL, 7.62 mmol, 1.3 equiv.), DIPEA (1.53 mL, 8.78 mmol, 1.5 equiv.), dibutylboryltrifilate solution (1 M in CH_2Cl_2) (7.64 mL, 7.64 mmol, 1.3 equiv.), and compound **13** (1.00 g, 5.88 mmol, 1 equiv.), CH_2Cl_2 (30 mL), potassium buffer solution (pH 7.4, 13 mL), MeOH (20 mL), and H_2O_2 (30 % solution, 6.9 mL). After 16 h the reaction was subjected to purification outlined in General Procedure B (silica gel, 15-20% EtOAc/petroleum ether) to afford the title compound as a colourless oil (1.21 g, 51% *syn*-isomer).

TLC (20% EtOAc/petroleum ether): R_f = 0.42 stained by $KMnO_4$.

υ_{max} (neat): 3502, 2958, 2930, 2870, 1727, 1372, 1236, 1022 cm^{-1}.

^{1}**H NMR** (500 MHz, $CDCl_3$): δ 5.77 – 5.64 (m, 2H), 5.53 – 5.44 (m, 1H), 5.09 – 5.02 (m, 2H), 4.95 – 4.86 (m, 1H), 4.19 – 4.10 (m, 2H), 3.91 – 3.78 (m, 1H), 3.37 – 3.31 (m, 0.2H (minor)), 2.98 – 2.91 (m, 0.8H (major)), 2.83 (br. s, 0.2H (minor)),

2.74 (br. s, 0.8H (major)), 2.34 – 2.24 (m, 2H), 2.12 – 2.04 (m, 2H), 2.04 – 1.98 (m, 3H), 1.83 – 1.67 (m, 1H), 1.65 – 1.31 (m, 3H), 1.30 – 1.20 (m, 3H), 1.01 – 0.95 (m, 3H).

^{13}C NMR (101 MHz, $CDCl_3$): δ 173.91, 170.88, 138.93, 137.66 (minor), 133.70, 133.66, 122.03, 122.00, 121.63 (minor), 117.88, 73.46, 72.95, 71.64 (minor), 71.52, 71.15 (minor), 71.03, 61.03 (minor), 60.99, 54.89, 54.86, 49.5 (minor), 49.39 (minor), 38.83 (minor), 38.78, 38.74, 30.06 (minor), 30.02 (minor), 29.93, 29.76 (minor), 29.70 (minor), 29.66, 29.62, 25.79, 21.29, 14.25, 14.12 (minor), 13.61.

HRMS: exact mass calculated for $[M+H]^+$ ($C_{17}H_{29}O_5$) requires *m/z* 313.2010, found *m/z* 313.2009.

Compound S22.

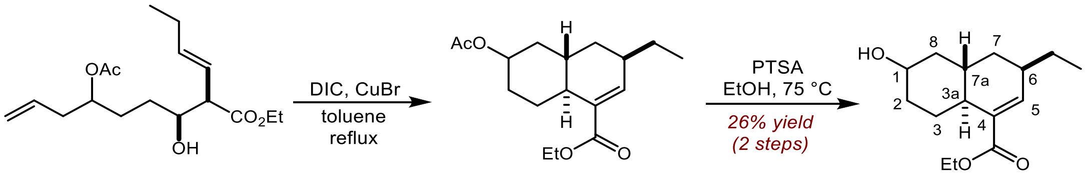

S22 was prepared according to General Procedure C using compound ***syn*-S21** (103 mg, 0.33 mmol, 1 equiv. 78% purity), CuBr (5 mg, 0.03 mmol, 10 mol%), DIC (80 µL, 0.51 mmol, 1.5 equiv.) and toluene (0.1 mL). After 16 h the reaction was subjected to purification outlined in General Procedure C (silica gel, 10% EtOAc/petroleum ether) to afford compound **S25** as a colourless oil which was not characterised.

S22 was prepared according to General Procedure C using **S25**, PTSA (mono-hydrate) (43 mg, 0.23 mmol, 1.2 equiv.), and EtOH (2 mL). After 5 h the reaction was subjected to purification outlined in General Procedure C (silica gel, 30% EtOAc/petroleum ether) to afford the title compound as a colourless oil and as two separable diastereoisomers at C^1 (17 mg, 26% (combined yield)), the relative stereochemistry of which were not confirmed.

Isomer 1:

TLC (30% EtOAc/petroleum ether): R_f = 0.28 stained by $KMnO_4$.

υ_{max} (neat): 3456 (br.), 2960, 2922, 2871, 1708, 1447, 1371, 1262, 1234, 1079, 1026 cm^{-1}.

^{1}H NMR (500 MHz, $CDCl_3$): δ 6.74 – 6.68 (m, 1H), 4.25 – 4.12 (m, 3H), 2.17 – 2.09 (m, 2H), 2.00 – 1.92 (m, 1H), 1.87 – 1.80 (m, 1H), 1.78 – 1.62 (m, 3H), 1.59 – 1.49 (m, 1H), 1.45 – 1.32 (m, 5H), 1.32 – 1.25 (m, *J* = 7.1 Hz, 4H), 0.97 (t, *J* = 7.4 Hz, 3H).

^{13}C NMR (126 MHz, $CDCl_3$): δ 168.2, 142.2, 134.4, 66.8, 60.2, 42.5, 40.3, 37.1, 33.7, 32.8, 29.6, 27.8, 24.1, 14.5, 12.7.

HRMS: exact mass calculated for $[M+H]^+$ ($C_{15}H_{25}O_3$) requires *m/z* 253.1798, found *m/z* 253.1801.

Calculated for a mixture of isomer 1 and 2.

Isomer 2:

TLC (30% EtOAc/petroleum ether): R_f = 0.17 stained by $KMnO_4$.

υ_{max} (neat): 3359 (br.), 2968, 2929, 2865, 1708, 1449, 1370, 1247, 1075, 1024 cm^{-1}.

^{1}H NMR (500 MHz, $CDCl_3$): δ 6.70 (dd, *J* = 4.8, 1.8 Hz, 1H), 4.26 – 4.09 (m, 2H), 3.73 – 3.64 (m, 1H), 2.39 –2.32 (m, 1H), 2.19 – 2.12 (m, 1H), 2.09 – 2.02 (m, 1H), 1.97 – 1.89 (m, 2H), 1.58 – 1.43 (m, 5H), 1.43 – 1.32 (m, 2H), 1.32 – 1.24 (m, 3H), 1.23 – 1.11 (m, 1H), 0.98 (t, *J* = 7.4 Hz, 3H), 0.96 – 0.85 (m, 1H).

^{13}C NMR (126 MHz, $CDCl_3$): δ 168.2, 142.3, 134.1, 70.9, 60.3, 42.6, 41.7, 37.1, 36.1, 34.5, 33.0, 28.1, 27.8, 14.5, 12.6.

HRMS: exact mass calculated for $[M+H]^+$ ($C_{15}H_{25}O_3$) requires *m/z* 253.1798, found *m/z* 253.1801.

Calculated for a mixture of isomer 1 and 2.

Compound S23.

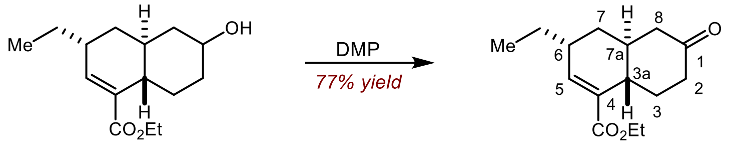

Compound **S23** was prepared according to General Procedure D using compound **S22** (17 mg, 0.07 mmol, 1 equiv.), DMP (43 mg, 0.10 mmol, 1.5 equiv.) and CH_2Cl_2 (0.7 mL). After 16 h the reaction was subjected to purification outlined in General Procedure D (silica gel, 10% EtOAc/petroleum ether) to afford the title compound as a colourless oil (13 mg, 77%).

TLC (10% EtOAc/petroleum ether): R_f = 0.20 stained by $KMnO_4$.

υ_{max} (neat): 3385 (br.), 2972, 2931, 2874, 1707, 1460, 1446, 1265 cm^{-1}.

^{1}H NMR (500 MHz, $CDCl_3$): δ 6.84 (dd, J = 5.2, 2.2 Hz, 1H, H^5), 4.26 – 4.14 (m, 2H, $CO_2CH_2CH_3$), 2.69 – 2.63 (m, 1H, H^3), 2.50 – 2.33 (m, 4H, H^2, H^{3a}, H^7), 2.27 – 2.18 (m, 2H, H^6, $H^{7'}$), 1.75 – 2.18 (m, 1H, H^{7a}), 1.57 – 1.48 (m, 3H, H^8, CH_3CH_2), 1.38 – 1.21 (m, 5H, $H^{3'}$, CH_3CH_2', $CO_2CH_2CH_3$), 0.98 (t, J = 7.4 Hz, 3H, CH_2CH_3).

^{13}C NMR (101 MHz, $CDCl_3$): δ 210.8 (CO), 167.5 (CO_2Et), 143.0 (C^5), 132.8 (C^4), 60.4 ($CO_2CH_2CH_3$), 48.2 (C^7), 41.5 (C^2), 40.9 (C^{3a}), 36.9 ($C^{6/7a}$), 36.6 ($C^{6/7a}$), 32.9 (C^8), 29.5 (C^3), 27.5 (CH_2CH_3), 14.3 ($CO_2CH_2CH_3$), 12.4 (CH_2CH_3).

HRMS: exact mass calculated for $[M+H]^+$ ($C_{15}H_{23}O_3$) requires m/z 251.1642, found m/z 251.1645.

Compound S24.

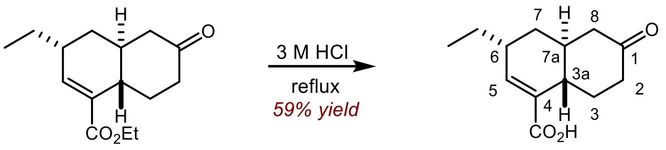

Prepared according to General Procedure E using compound **S23** (103 mg, 0.41 mmol) and 3 M HCl (12 mL). After 16 h the reaction was subjected to purification outlined in General Procedure E (silica gel, 30% EtOAc/petroleum ether) to afford the desired product as a colourless oil which solidified to a white solid on standing (54 mg, 59%).

TLC (30% EtOAc/petroleum ether): R_f = 0.19 stained by $KMnO_4$.

υ_{max} (neat): 2916 (br.), 2871, 1706, 1676, 1429, 1278 cm^{-1}.

m.p.: 114-116 °C. Crystallised by vapour diffusion (EtOAc/petroleum ether).

^{1}H NMR (500 MHz, $CDCl_3$): δ 11.44 (br. s, 1H, CO_2H), 7.07 (dd, J = 5.2, 2.0 Hz, 1H, H^5), 2.81 – 2.74 (m, 1H, H^3), 2.52 – 2.35 (m, 4H, H^2, H^{3a}, H^7), 2.31 – 2.20 (m, 2H, H^6, $H^{7'}$), 1.78 – 1.66 (m, 1H, H^{7a}), 1.59 – 1.49 (m, 3H, H^8, CH_3CH_2), 1.41 – 1.24 (m, 2H, $H^{3'}$, CH_3CH_2'), 0.99 (t, J = 7.4 Hz, 3H, CH_2CH_3).

^{13}C NMR (101 MHz, $CDCl_3$): δ 210.8 (C^1), 172.5 (CO_2H), 146.6 (C^5), 131.8 (C^4), 48.3 (C^7), 41.6 (C^2), 40.7 (C^{3a}), 37.2 (C^6), 36.7 (C^{7a}), 32.9 (C^8), 29.6 (C^3), 27.5 (CH_2CH_3), 12.5 (CH_2CH_3).

HRMS: exact mass calculated for $[M+H]^+$ ($C_{13}H_{19}O_3$) requires m/z 223.1334, found m/z 223.1336.

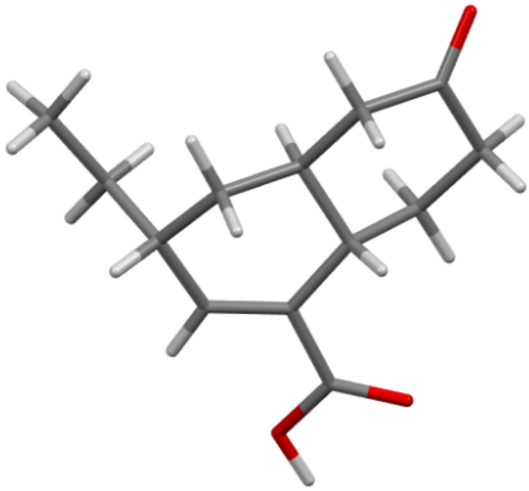

Supplementary Table 5. Crystal data and structure refinement for watson_mllb04s401monop.

Identification code	shelx	
Empirical formula	C13 H18 O3	
Formula weight	222.27	
Temperature	163(2) K	
Wavelength	0.71073 Å	
Crystal system	Monoclinic	
Space group	P 2_1/n	
Unit cell dimensions	a = 5.4512(2) Å	α= 90°.
	b = 9.6960(3) Å	β= 96.733(3)°.
	c = 23.0078(8) Å	γ = 90°.
Volume	1207.69(7) $Å^3$	
Z	4	
Density (calculated)	1.222 Mg/m^3	
Absorption coefficient	0.086 mm^{-1}	
F(000)	480	
Crystal size	0.35 x 0.28 x 0.10 mm^3	
Theta range for data collection	3.401 to 28.979°.	
Index ranges	-7<=h<=6, -9<=k<=12, -31<=l<=24	
Reflections collected	6091	
Independent reflections	3029 [R(int) = 0.0162]	
Completeness to theta = 26.000°	99.8 %	
Absorption correction	Semi-empirical from equivalents	
Max. and min. transmission	1.00000 and 0.93827	
Refinement method	Full-matrix least-squares on F^2	

Data / restraints / parameters	3029 / 0 / 150
Goodness-of-fit on F^2	1.045
Final R indices [I>2sigma(I)]	R1 = 0.0430, wR2 = 0.0997
R indices (all data)	R1 = 0.0635, wR2 = 0.1132
Extinction coefficient	n/a
Largest diff. peak and hole	0.231 and -0.173 e.$Å^{-3}$

Supplementary Table 6. Atomic coordinates (x 10^4) and equivalent isotropic displacement parameters ($Å^2$x 10^3)
for watson_mllb04s401monop. U(eq) is defined as one third of the trace of the orthogonalized U^{ij} tensor.

	x	y	z	U(eq)
O(1)	-3282(2)	3140(1)	2518(1)	55(1)
O(2)	3908(2)	4715(1)	608(1)	37(1)
O(3)	4469(2)	3135(1)	-69(1)	43(1)
C(1)	3028(2)	2384(1)	812(1)	29(1)
C(2)	3637(2)	1086(1)	699(1)	36(1)
C(3)	3252(3)	-128(1)	1081(1)	38(1)
C(4)	2830(3)	360(1)	1694(1)	36(1)
C(5)	959(2)	1525(1)	1660(1)	31(1)
C(6)	1842(2)	2793(1)	1346(1)	28(1)
C(7)	-358(2)	3771(1)	1221(1)	33(1)
C(8)	-1237(3)	4262(2)	1793(1)	40(1)
C(9)	-1587(3)	3117(2)	2219(1)	39(1)
C(10)	266(3)	1960(1)	2261(1)	39(1)
C(11)	3805(2)	3517(1)	441(1)	31(1)
C(12)	1183(3)	-1044(2)	779(1)	43(1)
C(13)	820(4)	-2387(2)	1103(1)	55(1)
H(13B)	-2971	863	830	Uiso
H	-2183	1476	830	Uiso

Synthesis of Compound S28.

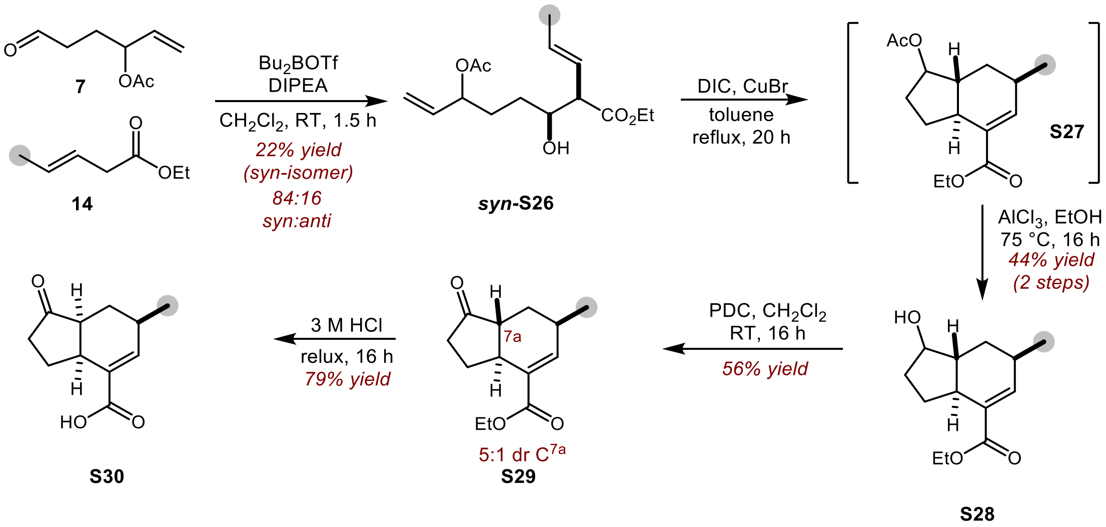

Compound *syn*-S26.

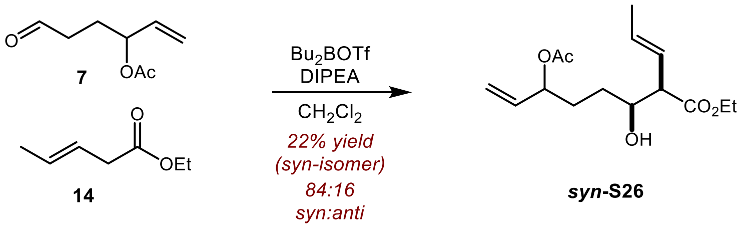

Prepared according to General Procedure B using ethyl (*E*)-pent-3-enoate (**14**) (265 mg, 2.07 mmol, 1.3 equiv.), DIPEA (0.4 mL, 2.30 mmol, 1.5 equiv.), dibutylboryltrifilate solution (1 M in CH_2Cl_2) (2.10 mL, 2.10 mmol, 1.3 equiv.), compound **7** (250 mg, 1.60 mmol, 1 equiv.), CH_2Cl_2 (7 mL), potassium buffer solution (pH 7.4, 3 mL), MeOH (5 mL) and H_2O_2 (30 % solution, 1.5 mL). After 16 h the reaction was subjected to purification outlined in General Procedure B (silica gel, 15-20% EtOAc/petroleum ether) to afford the title compound as a colourless oil (101 mg, 22% *syn*-isomer).

Product contains 35% alkene isomerisation impurity. Data reported of products resulting from reaction carried out at – 78 °C to where isomerisation does not take place.[10]

TLC (30% EtOAc/petroleum ether): R_f = 0.64 stained by $KMnO_4$.

υ_{max} (neat): 3522 (br.), 2954, 1730, 1370, 1235, 1176, 1021, 969 cm^{-1}.

^{1}H NMR (500 MHz, $CDCl_3$): δ 5.81 – 5.72 (m, 1H), 5.72 – 5.62 (m, 1H), 5.58 – 5.50 (m, 1H), 5.29 – 5.14 (m, 3H), 4.21 – 4.10 (m, 2H), 3.87 – 3.81 (m, 1H), 2.97 (dd, *J* = 9.2, 4.8 Hz, 1H), 2.67 (br. s, 1H), 2.05 (s, 3H), 1.89 – 1.79 (m, 1H), 1.74 (d, *J* = 6.4 Hz, 3H), 1.71 – 1.61 (m, 1H), 1.54 – 1.37 (m, 2H), 1.30 – 1.22 (m, 3H).

^{13}C NMR (101 MHz, $CDCl_3$): δ 173.8, 170.5, 136.5, 136.4, 132.0, 124.3, 117.0, 116.9, 74.9, 74.5, 71.4, 71.1, 61.0, 55.0, 55.0, 30.5, 30.3, 29.7, 29.5, 21.4, 21.3, 18.3, 14.3.

HRMS: exact mass calculated for $[M+Na]^+$ ($C_{15}H_{24}O_5Na$) requires *m/z* 307.1516, found *m/z* 307.1513.

Compound S29.

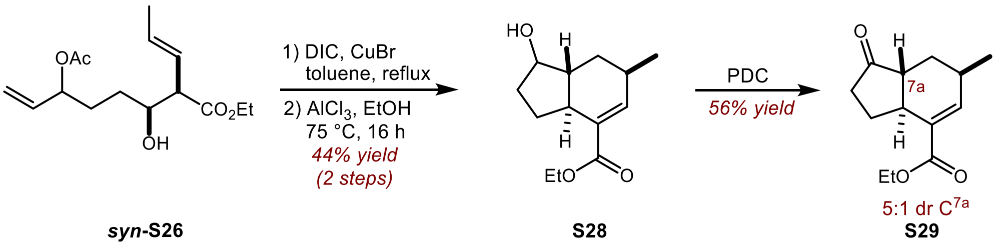

Intermediate **S28** prepared according to General Procedure C using compound ***syn*-S26** (880 mg, 3.09 mmol, 1 equiv. (65% purity)), CuBr (44 mg, 0.31 mmol, 0.1 equiv.), DIC (0.73 mL, 4.66 mmol, 1.5 equiv.), and toluene (25 mL). After 20 h the reaction was subjected to purification outlined in General Procedure C (silica gel, 10% EtOAc/petroleum ether) to afford a colourless oil (**S27**) (838 mg, 3.15 mmol) which was further reacted according to General Procedure C using $AlCl_3$ (420 mg, 3.15 mmol, 1 equiv.) and EtOH (60 ml). After 16 h the reaction was subjected to purification outlined in General Procedure C (silica gel, 20% EtOAc/petroleum ether) to afford compound **S28** as a colourless oil and a mixture of diastereoisomers which was not characterised. (199 mg, 44% based on 65% purity of starting material).

To a round bottom flask was added compound **S28** (171 mg, 0.76 mmol, 1 equiv.) and anhydrous CH_2Cl_2 (5 mL). PDC (430 mg, 1.14 mmol, 1.5 equiv.) was added in one portion and the reaction was stirred at room temperature for 16 h. The crude reaction mixture was concentrated onto silica gel and purified by flash silica column chromatography, eluent 10% EtOAc/petroleum ether to afford the title compound as a colourless oil (95 mg, 56%) (5:1 dr C^{7a}).

TLC (10% EtOAc/petroleum ether): R_f = 0.26 stained by $KMnO_4$.

υ_{max} (neat): 3375, 2957, 2874, 1736, 1707, 1240, 1211, 1091, 754 cm^{-1}.

^{1}H NMR (400 MHz, $CDCl_3$): δ 6.84 – 6.80 (m, 1H), 4.31 – 4.13 (m, 2H), 2.76 – 2.63 (m, 2H), 2.51 – 2.30 (m, 2H), 2.29 – 2.17 (m, 1H), 2.01 – 1.85 (m, 2H), 1.64 – 1.51 (m, 2H), 1.31 (t, J = 7.1 Hz, 3H), 1.10 (d, J = 7.3 Hz, 3H).

Major *trans*-isomer:

^{13}C NMR (101 MHz, $CDCl_3$): δ 217.0, 166.7, 146.3, 132.6, 60.5, 50.6, 40.9, 38.4, 31.5, 27.5, 26.2, 21.0, 14.5.

Minor *cis*-isomer:

^{13}C NMR (101 MHz, $CDCl_3$): δ 145.2, 60.6, 47.0, 38.3, 36.1, 31.3, 28.6, 28.3, 20.6.

HRMS: exact mass calculated for $[M+H]^+$ ($C_{13}H_{19}O_3$) requires m/z 223.1334 found m/z 223.1345.

Compound S30.

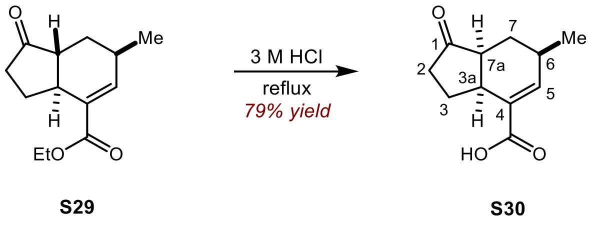

Prepared according to General Procedure E using compound **S29** (83 mg, 0.37 mmol, 1 equiv.) and 3 M HCl (12 mL). After 16 h the reaction was subjected to purification outlined in General Procedure E (silica gel, 40-70% EtOAc/petroleum ether) to afford the tile compound as a white solid (57 mg, 79%).

TLC (30% EtOAc/petroleum ether): R_f = 0.15 stained by $KMnO_4$.

υ_{max} (neat): 2947 (br.), 2641 (br.), 2521 (br.), 1730, 1674, 1626, 1269, 1136, 1057 cm^{-1}.

^{1}H NMR (400 MHz, $CDCl_3$): δ 7.02 (s, 1H, H^5), 3.12 – 3.03 (m, 1H, H^{3a}), 2.61 (dt, J = 12.9, 7.7 Hz, 1H, H^3), 2.47 – 2.24 (m, 4H, H^2, H^6, H^{7a}), 1.87 (dt, J = 12.9, 4.8 Hz, 1H, H^7), 1.68 – 1.54 (m, 1H, $H^{3'}$), 1.14 (d, J = 7.2 Hz, 3H, CH_3), 1.12 – 1.02 (m, 1H, $H^{7'}$). CO_2H not observed.

^{13}C NMR (101 MHz, $CDCl_3$): δ 220.2 (C^1), 172.0 (CO_2H), 148.2 (C^5), 130.7 (C^4), 46.9 (*C*H), 38.3 (C^2), 35.8 (*C*H), 31.5 (C^6), 28.4 ($C^{3/7}$), 28.2 ($C^{3/7}$), 20.5 (CH_3).

HRMS: exact mass calculated for $[M-H]^-$ ($C_{11}H_{13}O_3$) requires *m/z* 193.0870 found *m/z* 193.0872.

The spectral data were consistent with those previously reported in the literature.[12]

Synthesis of Compound S35.

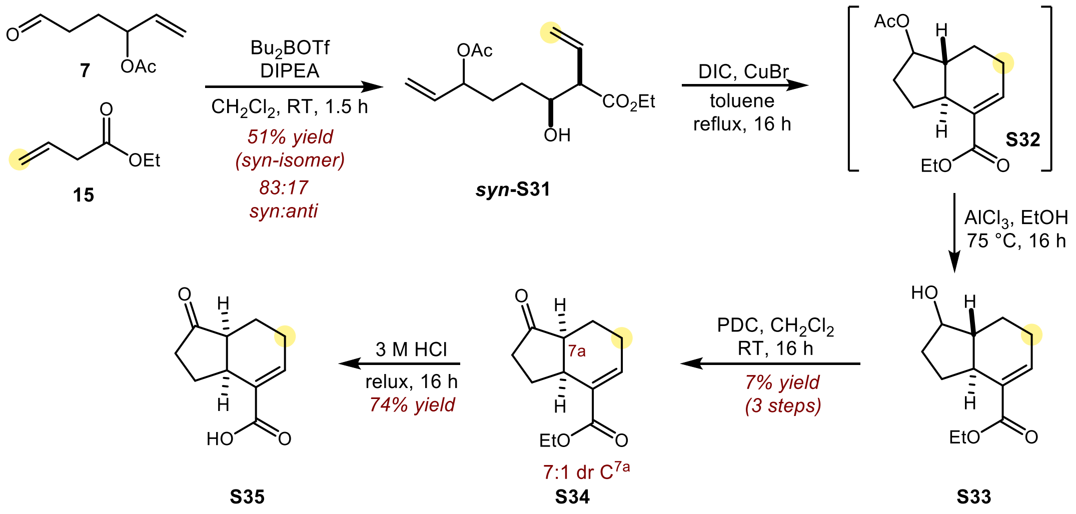

Compound *syn*-S31.

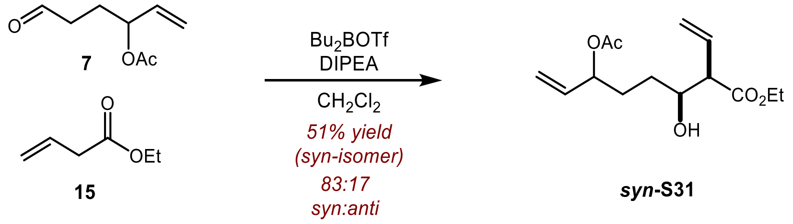

Prepared according to General Procedure B using ethyl but-3-enoate (**15**) (1.50 g, 13.14 mmol, 1.3 equiv.), DIPEA (2.3 mL, 13.20 mmol, 1.5 equiv.), dibutylboryltrifilate solution (1 M in CH_2Cl_2) (13.14 mL, 13.14 mmol, 1.3 equiv.), compound **7** (1.37 g, 8.77 mmol, 1 equiv.), CH_2Cl_2 (50 mL), potassium buffer solution (pH 7.4, 17 mL), MeOH (25 mL) and H_2O_2 (30 % solution, 9 mL). After 16 h the reaction was subjected to purification outlined in General Procedure B (silica gel, 15-40% EtOAc/petroleum ether) to afford the title compound as a colourless oil (1.46 g, 51% (^{1}H NMR yield), 83:17 *syn/anti* as inseparable *syn/anti* diastereoisomers).

TLC (30% EtOAc/petroleum ether): R_f = 0.44 stained by $KMnO_4$.

υ_{max} (neat): 3525 (br.), 2978, 2935, 2867, 1729, 1238 cm^{-1}.

^{1}H NMR (400 MHz, $CDCl_3$): δ 5.98 – 5.86 (m, 1H), 5.81 – 5.71 (m, 1H), 5.34 – 5.14 (m, 5H), 4.22 – 4.13 (m, 2H), 3.94 – 3.88 (m, 1H), 3.03 (dd, J = 9.2, 4.5 Hz, 1H), 2.05 (s, 3H), 1.90 – 1.80 (m, 1H), 1.73 – 1.60 (m, 1H), 1.59 – 1.35 (m, 2H), 1.27 (t, J = 7.1 Hz, 3H). CO_2H not observed.

^{13}C NMR (101 MHz, $CDCl_3$): δ 173.4, 173.3, 170.5, 136.4, 136.4, 131.7, 120.8, 117.1, 117.0, 74.8, 74.4, 71.2, 70.9, 61.2, 56.0, 55.9, 30.5, 30.3, 29.7, 29.5, 21.3, 14.3.

HRMS: exact mass calculated for $[M+H]^+$ ($C_{14}H_{23}O_5$) requires *m/z* 271.1540, found *m/z* 271.1541.

Compound S34.

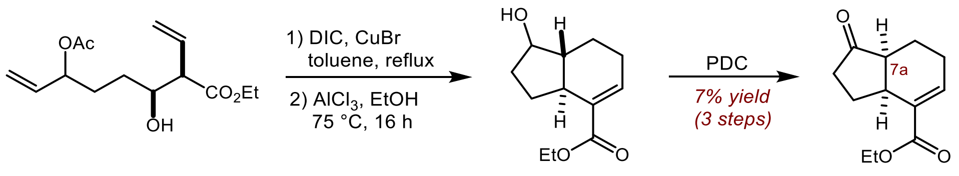

syn-**S31** **S33** **S34**

Intermediate **S33** prepared according to General Procedure C using compound ***syn*-S31** (1.45 g, 5.42 mmol, 1 equiv.), CuBr (78 mg, 0.54 mmol, 0.1 equiv.), DIC (1.27 mL, 8.11 mmol, 1.5 equiv.) and toluene (40 mL). After 16 h the reaction was subjected to purification outlined in General Procedure C (silica gel, 10% EtOAc/petroleum ether) to afford a colourless oil (**S32**) (1.11 g, 4.16 mmol) which was further reacted according to General Procedure C using $AlCl_3$ (555 mg, 4.16 mmol, 1 equiv.) and EtOH (65 ml). After 16 h the reaction was subjected to purification outlined in General Procedure C (silica gel, 30% EtOAc/petroleum ether) to afford a colourless oil (**S33**) as two separable diastereoisomers (163 g, 0.78 mmol) which were not characterized.

To a round bottom flask was added compound **S33** (163 mg, 0.78 mmol, 1 equiv.) and anhydrous CH_2Cl_2 (5 mL). PDC (437 mg, 1.16 mmol, 1.5 equiv.) was added in one portion and the reaction was stirred at room temperature for 16 h. The crude reaction mixture was concentrated onto silica gel and purified by flash silica column chromatography, eluent 10% EtOAc/petroleum ether to afford the title compound as a colourless oil (83 mg, 7% (3 steps) 7:1 dr C^{7a}).

TLC (10% EtOAc/petroleum ether): R_f = 0.16 stained by $KMnO_4$.

υ_{max} (neat): 3362 (br.), 2978, 2938, 1736, 1705, 1248, 1092, 1057 cm^{-1}.

^{1}H NMR (500 MHz, $CDCl_3$): δ 7.02 (dd, J = 3.9, 3.3 Hz, 1H), 4.26 – 4.10 (m, 2H), 3.20 – 3.13 (m, 1H), 2.45 – 2.29 (m, 2H), 2.25 – 2.05 (m, 4H), 1.83 – 1.74 (m, 1H), 1.70 – 1.61 (m, 2H), 1.27 (t, J = 7.1 Hz, 3H).

Major *cis*-isomer:

^{13}C NMR (101 MHz, $CDCl_3$): δ 216.0, 166.8, 140.23, 131.9, 60.4, 46.7, 37.2, 35.8, 27.5, 24.0, 19.6, 14.3.

Minor *trans*-isomer:

^{13}C NMR (101 MHz, $CDCl_3$): δ 166.4, 141.2, 133.4, 60.4, 53.9, 40.3, 37.9, 27.1, 26.2, 20.5.

HRMS: exact mass calculated for $[M+H]^+$ ($C_{12}H_{17}O_3$) requires m/z 209.1178, found m/z 209.1176.

Compound S35.

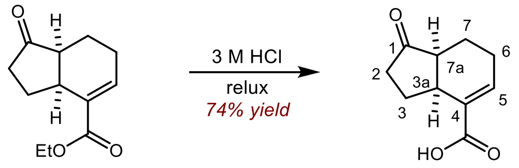

S34 **S35**

Prepared according to General Procedure E using compound **S34** (159 mg, 0.88 mmol) and 3 M HCl (20 mL). After 16 h the reaction was subjected to purification outlined in General Procedure E (silica gel, 30-70% EtOAc/petroleum ether) to afford a white solid, which was washed with minimal petroleum ether to afford the title compound as a white solid (102 mg, 74%).

TLC (30% EtOAc/petroleum ether): R_f = 0.20 stained by $KMnO_4$.

υ_{max} (neat): 2938, 2895, 2627, 2532, 1736, 1661, 1632, 1427, 1283, 930 cm^{-1}.

^{1}H NMR (400 MHz, $CDCl_3$): δ 11.75 (br. s, 1H, CO_2H), 7.24 (td, J = 4.1, 1.1 Hz, 1H, H^5), 3.25 – 3.15 (m, 1H, H^{3a}), 2.50 – 2.36 (m, 2H, H^3, H^{7a}), 2.34 – 2.19 (m, 4H, H^2, H^6), 1.92 – 1.82 (m, 1H, $H^{3'}$), 1.78 – 1.66 (m, 2H, H^7).

^{13}C NMR (101 MHz, $CDCl_3$): δ 220.6 (C^1), 172.3 (CO_2H), 143.6 (C^5), 131.3 (C^4), 46.7 (C^{7a}), 37.3 (CH_2), 35.7 (C^{3a}), 27.5 (C^3), 24.4 (CH_2), 19.6 (C^7).

HRMS: exact mass calculated for $[M-H]^-$ ($C_{10}H_{11}O_3$) requires *m/z* 179.0714, found *m/z* 179.0716.

The spectral data were consistent with those previously reported in the literature.[12]

***N*-coronafacoyl Analogue Procedures and Characterization (Figure 3).**

Compound S4.

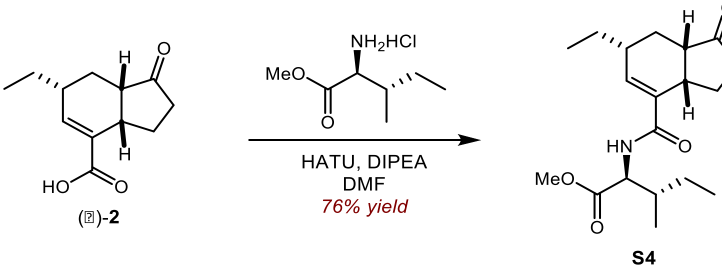

Prepared according to General Procedure F using (±)-CFA (**2**) (30 mg, 0.14 mmol, 1 equiv.), HATU (66 mg, 0.17 mmol, 1.2 equiv.), methyl *L*-isoleucinate hydrochloride (30 mg, 0.21 mmol, 1.5 equiv.), DIPEA (80 µL, 0.46 mmol, 3 equiv.), and DMF (0.7 mL). After 16 h the reaction was subjected to purification outlined in General Procedure F (silica gel, 30% EtOAc/CH_2Cl_2) to afford the title compound as a colourless oil which solidified to a white solid on standing (35 mg, 76%).

TLC (30% EtOAc/petroleum ether): R_f = 0.19 stained by $KMnO_4$.

υ_{max} (film): 3323 (br.), 2963, 2938, 2877, 1735, 1658, 1621, 1518, 1203, 1147 cm^{-1}.

^{1}H NMR (400 MHz, $CDCl_3$): δ 6.42 – 6.34 (m, 1H), 6.31 – 6.23 (m, 1H), 4.73 – 4.65 (m, 1H), 3.76 (s, 3H), 3.23 – 3.09 (m, 1H), 2.54 – 2.23 (m, 4H), 2.21 – 2.11 (m, 1H), 1.98 – 1.86 (m, 2H), 1.68 – 1.35 (m, 4H), 1.28 – 1.16 (m, 1H), 1.13 – 1.01 (m, 1H), 1.01 – 0.91 (m, 9H).

^{13}C NMR (101 MHz, $CDCl_3$): δ 220.4, 220.3, 173.0, 167.9, 167.8, 137.1, 135.8, 135.6, 56.5, 56.5, 52.3, 46.6, 46.6, 38.3, 38.3, 38.2, 37.5, 37.4, 36.4, 36.4, 28.3, 28.2, 28.0, 28.0, 26.2, 26.2, 25.5, 25.4, 15.7, 15.6, 11.7, 11.7, 11.5, 11.4.

HRMS: exact mass calculated for $[M+H]^+$ ($C_{19}H_{30}NO_4$) requires *m/z* 336.2169, found *m/z* 336.2173.

Compound 16.

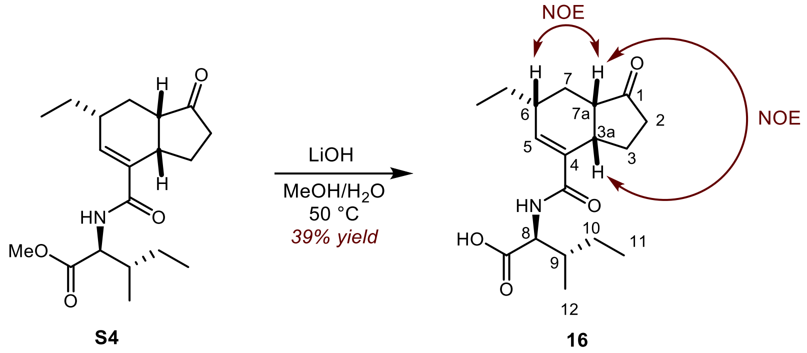

Prepared according to General Procedure G using compound **S4** (24 mg, 0.07 mmol, 1 equiv.), LiOH (5 mg, 0.20 mmol, 3 equiv.), and 1:1 MeOH:H_2O (3 mL). After 16 h at 50 °C the reaction mixture was subjected to purification outlined in General Procedure G to afford the title compound as a white solid (9 mg, 39%).

υ_{max} (film): 2967, 2926, 2862, 1728, 1655, 1610, 1516, 1457, 1142 cm^{-1}.

^{1}H NMR (500 MHz, $CDCl_3$): δ 6.43 – 6.38 (m, 1H, H^5), 6.29 – 6.25 (m, 1H, N*H*), 4.73 – 4.66 (m, 1H, H^8), 3.22 – 3.10 (m, 1H, H^{3a}), 2.53 – 2.25 (m, 4H, H^3, H^{7a}, H^2), 2.21 – 2.12 (m, 1H, H^6), 2.07 – 1.97 (m, 1H, H^9), 1.94 – 1.86 (m, 1H, H^7), 1.67 –

1.48 (m, 3H, H3', H10, CH_3CH_2), 1.44 – 1.35 (m, 1H, CH_3CH_2'), 1.31 – 1.19 (m, 1H, H10'), 1.13 – 1.02 (m, 1H, H7'), 1.02 – 0.92 (m, 9H, H12, H11, CH_3CH_2). CO_2H not observed.

^{13}C NMR (126 MHz, $CDCl_3$): δ 175.5, 175.5, 168.5, 168.4, 137.8, 137.7, 135.5, 135.4, 56.8, 56.6, 46.6, 46.6, 38.3, 37.9, 37.8, 37.5, 37.4, 36.4, 36.4, 28.2, 28.2, 28.0, 27.9, 26.1, 26.1, 25.4, 25.3, 15.8, 15.7, 11.7, 11.7, 11.5, 11.4.

HRMS: exact mass calculated for $[M-H]^-$ ($C_{18}H_{26}NO_4$) requires *m/z* 320.1867, found *m/z* 320.1865.

The spectral data were consistent with those previously reported in the literature.[13]

Compound S36.

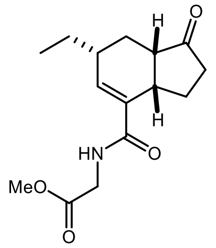

S36

Prepared according to General Procedure F using (±)-CFA (**2**) (30 mg, 0.12 mmol, 1 equiv.), HATU (66 mg, 0.17 mmol, 1.2 equiv.), glycine methyl ester hydrochloride (36 mg, 0.21 mmol, 1.5 equiv.), DIPEA (80 µL, 0.46 mmol, 3 equiv.) and DMF (0.7 mL). After 16 h the reaction was subjected to purification outlined in General Procedure F (silica gel, 70% EtOAc/petroleum ether) to afford the title compound as a colourless oil (18 mg, 45%).

TLC (50% EtOAc/petroleum ether): R_f= 0.15 stained by $KMnO_4$.

υ_{max} (film): 3344 (br.), 2956, 2937, 2875, 2858, 1735, 1655, 1624, 1204, 1181, 1151 cm^{-1}.

^{1}H NMR (500 MHz, $CDCl_3$): δ 6.40 (s, 1H), 6.30 (br. s, 1H), 4.12 (d, *J* = 5.2 Hz, 2H), 3.78 (s, 3H), 3.21 – 3.13 (m, 1H), 2.51 – 2.43 (m, 1H), 2.43 – 2.23 (m, 3H), 2.21 – 2.12 (m, 1H), 1.91 – 1.85 (m, 1H), 1.65 – 1.55 (m, 1H), 1.54 – 1.46 (m, 1H), 1.43 – 1.35 (m, 1H), 1.11 – 1.02 (m, 1H), 0.97 (t, *J* = 7.4 Hz, 3H).

^{13}C NMR (101 MHz, $CDCl_3$): δ 220.5, 170.8, 168.2, 137.4, 135.4, 52.6, 46.5, 41.5, 38.3, 37.4, 36.3, 28.2, 28.0, 26.1, 11.4.

HRMS: exact mass calculated for $[M+H]^+$ ($C_{15}H_{22}NO_4$) requires *m/z* 280.1543, found *m/z* 280.1543.

Compound 17.

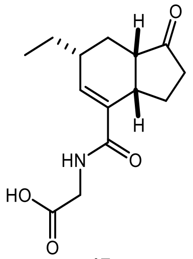

17

Prepared according to General Procedure G using compound **S36** (20 mg, 0.05 mmol, 1 equiv.), LiOH (5 mg, 0.21 mmol, 3 equiv.), and 1:1 MeOH:H_2O (4 mL). After 16 h the reaction was allowed to cool to room temperature, acidified with AcOH and the organics extracted with EtOAc (3 x 10 mL). The organics were combined, dried over Na_2SO_4, filtered, and evaporated to afford a pale yellow oil. The crude material was loaded in a solution of CH_2Cl_2 and purified by flash silica column chromatography, eluent 1% AcOH, 30-70% EtOAc/CH_2Cl_2 to afford a colourless oil. The solid material was washed with petroleum ether to afford the title compound as a colourless oil (13 mg, 68%).

TLC (1% AcOH, 30% EtOAc/CH_2Cl_2): R_f= 0.09 stained by $KMnO_4$.

υ_{max} (film): 3351 (br.), 2962, 2925, 2856, 1735, 1654, 1613, 1523, 1214 cm^{-1}.

^{1}H NMR (500 MHz, $CDCl_3$): δ 6.51 (s, 1H), 3.88 (br. s, 2H), 3.13 – 3.04 (br. s, 1H), 2.47 – 2.18 (m, 4H), 2.12 (br. s, 1H), 1.87 – 1.79 (m, 1H), 1.61 – 1.43 (m, 2H), 1.38 – 1.30 (m, 1H), 1.09 – 0.99 (m, 1H), 0.94 (t, J = 7.3 Hz, 3H). CO_2H and N*H* not observed.

^{13}C NMR (101 MHz, $CDCl_3$): δ 220.0, 169.1, 156.2, 138.7, 134.6, 46.8, 43.0, 38.1, 37.5, 36.1, 28.2, 28.2, 25.9, 11.4.

HRMS: exact mass calculated for $[M+H]^+$ ($C_{14}H_{20}NO_4$) requires *m/z* 266.1392, found *m/z* 266.1396.

Compound S37.

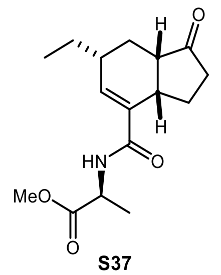

S37

Prepared according to General Procedure F using (±)-CFA (**2**) (30 mg, 0.14 mmol, 1 equiv.), HATU (66 mg, 0.17 mmol, 1.2 equiv.), *L*-alanine methyl ester hydrochloride (30 mg, 0.21 mmol, 1.5 equiv.), DIPEA (80 µL, 0.46 mmol, 3 equiv.), and DMF (0.7 mL). After 16 h the reaction was subjected to purification outlined in General Procedure F (silica gel, 20-35% EtOAc/petroleum ether) to afford the desired product as a colourless oil (35 mg, 83%).

TLC (40% EtOAc/petroleum ether): R_f= 0.17 stained by $KMnO_4$.

υ_{max} (film): 3312 (br.), 2934, 2958, 2878, 2857, 1738, 1658, 1621, 1521, 1455, 1210, 1158, 1072 cm^{-1}.

^{1}H NMR (500 MHz, $CDCl_3$): δ 6.43 – 6.30 (m, 2H), 4.71 – 4.62 (m, 1H), 3.77 (s, 3H), 3.21 – 3.11 (m, 1H), 2.53 – 2.24 (m, 4H), 2.19 – 2.12 (m, 1H), 1.92 – 1.84 (m, 1H), 1.64 – 1.55 (m, 1H), 1.55 – 1.47 (m, 1H), 1.45 (d, J = 7.1 Hz, 3H), 1.42 – 1.35 (m, 1H), 1.11 – 1.01 (m, 1H), 0.98 (t, J = 7.4 Hz, 3H).

^{13}C NMR (126 MHz, $CDCl_3$): δ 173.9, 167.8, 167.5, 137.4, 136.9, 135.7, 135.3, 52.7, 48.2, 48.2, 46.6, 46.5, 38.2, 37.4, 36.3, 36.3, 28.2, 28.2, 28.0, 27.9, 26.2, 26.1, 18.6, 18.6, 11.4, 11.4.

HRMS: exact mass calculated for $[M+H]^+$ ($C_{16}H_{24}NO_4$) requires *m/z* 294.1700, found *m/z* 294.1703.

Compound 18.

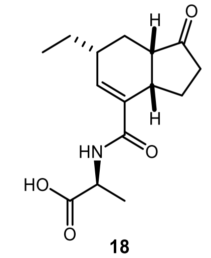

18

Prepared according to General Procedure G using compound **S37** (18 mg, 0.06 mmol, 1 equiv.), LiOH (5 mg, 0.21 mmol, 3 equiv.), and 1:1 MeOH:H_2O (2 mL). After 6 h the reaction was allowed to cool to room temperature, acidified with AcOH and the organics extracted with EtOAc (3 x 10 mL). The organics were combined, dried over Na_2SO_4, filtered, and evaporated to afford a pale yellow oil. The crude material was loaded in a solution of CH_2Cl_2 and was purified by flash silica column chromatography, eluent 1% AcOH, 30% EtOAc/CH_2Cl_2 to afford the title compound as a colourless oil which solidified to a white solid on standing (16 mg, 94%).

TLC (1% AcOH, 30% EtOAc/CH_2Cl_2): R_f= 0.14 stained by $KMnO_4$.

υ_{max} (film): 3323 (br.), 2954, 2924, 2855, 1735, 1654, 1617, 1526, 1453, 1147 cm^{-1}.

^{1}**H NMR** (500 MHz, $CDCl_3$): δ 7.58 (br. s, 1H), 6.55 (s, 1H), 6.45 – 6.39 (m, 1H), 4.60 – 4.49 (m, 1H), 3.19 – 3.07 (m, 1H), 2.51 – 2.23 (m, 4H), 2.19 – 2.10 (m, 1H), 1.91 – 1.83 (m, 1H), 1.63 – 1.42 (m, 5H), 1.41 – 1.33 (m, 1H), 1.11 – 1.01 (m, 1H), 1.00 – 0.94 (m, 3H).

^{13}**C NMR** (101 MHz, $CDCl_3$): δ 220.3, 168.6, 168.4, 138.1, 138.0, 135.1, 135.0, 49.2, 49.1, 46.6, 46.6, 38.2, 37.5, 37.5, 36.2, 36.2, 28.2, 28.0, 27.9, 26.1, 26.0, 18.1, 18.0, 11.5, 11.4.

HRMS: exact mass calculated for $[M\text{-}H]^-$ ($C_{15}H_{20}NO_4$) requires *m/z* 278.1398, found *m/z* 278.1400.

The spectral data were consistent with those previously reported in the literature.[14]

Compound S38.

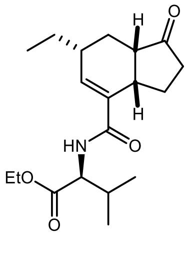

S38

Prepared according to General Procedure F using (±)-CFA (**2**) (27 mg, 0.13 mmol, 1 equiv.), HATU (66 mg, 0.17 mmol, 1.2 equiv.), *L*-valine ethyl ester hydrochloride (39 mg, 0.21 mmol, 1.5 equiv.), DIPEA (80 µL, 0.46 mmol, 3 equiv.), and DMF (0.7 mL). After 16 h the reaction was subjected to purification outlined in General Procedure F (silica gel, 30% EtOAc/petroleum ether) to afford the title compound as a colourless oil (35 mg, 85%).

TLC (30% EtOAc/petroleum ether): R_f= 0.26 stained by $KMnO_4$.

υ_{max} (film): 3341 (br.), 2962, 2930, 2875, 1735, 1659, 1624, 1513, 1192, 1148, 1025 cm^{-1}.

^{1}**H NMR** (500 MHz, $CDCl_3$): δ 6.43 – 6.35 (m, 1H), 6.30 – 6.22 (m, 1H), 4.66 – 4.59 (m, 1H), 4.28 – 4.15 (m, 2H), 3.22 – 3.11 (m, 1H), 2.53 – 2.11 (m, 6H), 1.92 – 1.85 (m, 1H), 1.66 – 1.57 (m, 1H), 1.57 – 1.46 (m, 1H), 1.42 – 1.35 (m, 1H), 1.30 (t, *J* = 7.1 Hz, 3H), 1.11 – 1.01 (m, 1H), 1.00 – 0.91 (m, 9H).

^{13}**C NMR** (126 MHz, $CDCl_3$): δ 172.5, 168.1, 168.0, 137.0, 137.0, 135.9, 135.8, 61.5, 57.2, 57.1, 46.7, 46.6, 38.3, 37.5, 37.4, 36.4, 31.7, 31.7, 28.3, 28.3, 28.0, 28.0, 26.2, 26.2, 19.2, 19.2, 18.1, 18.0, 14.4, 11.5, 11.4.

HRMS: exact mass calculated for $[M+H]^+$ ($C_{19}H_{30}NO_4$) requires *m/z* 336.2169, found *m/z* 336.2171.

Compound 19.

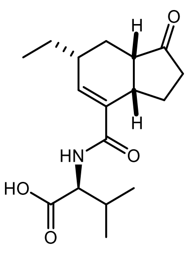

19

Prepared according to General Procedure G using compound **S38** (23 mg, 0.07 mmol, 1 equiv.), NaOH (8 mg, 0.20 mmol, 3 equiv.), and 1:1 $MeOH:H_2O$ (1 mL). After 16 h a further portion of NaOH (4 mg, 0.10 mmol, 1.5 equiv.) was added and the reaction stirred at 50 °C for a further 1.5 h. The reaction was subjected to purification outlined in General Procedure G to afford the title compound as a white solid (16 mg, 76%).

υ_{max} (film): 3323 (br.), 2961, 2924, 2874, 1730, 1651, 1607, 1518, 1204, 1146 cm^{-1}.

^{1}**H NMR** (500 MHz, $CDCl_3$): δ 6.44 – 6.39 (m, 1H), 6.29 – 6.23 (m, 1H), 4.69 – 4.62 (m, 1H), 3.22 – 3.11 (m, 1H), 2.53 – 2.36 (m, 3H), 2.36 – 2.25 (m, 2H), 2.21 – 2.13 (m, 1H), 1.93 – 1.86 (m, 1H), 1.67 –1.58 (m, 1H), 1.58 – 1.49 (m, 1H), 1.45 – 1.35 (m, 1H), 1.12 – 1.04 (m, 1H), 1.04 – 0.96 (m, 9H). CO_2*H* not observed.

^{13}**C NMR** (126 MHz, $CDCl_3$): δ 175.6, 168.6, 168.5, 137.7, 137.6, 135.6, 135.5, 57.4, 57.3, 46.6, 46.6, 38.3, 37.5, 37.4, 36.4, 31.3, 31.2, 28.3, 28.2, 28.0, 27.9, 26.2, 26.1, 19.3, 19.2, 18.0, 18.0, 11.5, 11.4.

HRMS: exact mass calculated for $[M-H]^-$ ($C_{17}H_{24}NO_4$) requires *m/z* 306.1711, found *m/z* 306.1709.

The spectral data were consistent with those previously reported in the literature.[14]

Compound S39.

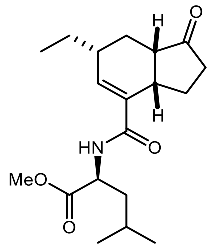

S39

Prepared according to General Procedure F using (±)-CFA (**2**) (50 mg, 0.24 mmol, 1 equiv.), HATU (110 mg, 0.30 mmol, 1.2 equiv.), DIPEA (0.13 mL, 0.72 mmol, 3 equiv.), *L*-leucine methyl ester hydrochloride (48 mg, 0.26 mmol, 1.1 equiv.), and DMF (1.2 mL). After 16 h the reaction was subjected to purification outlined in General Procedure F (silica gel, 30% EtOAC/petroleum ether) to afford the title compound colourless oil (74 mg, 91%).

TLC (30% EtOAc/petroleum ether): R_f = 0.31 stained by $KMnO_4$ and visible under UV (short wave).

$\boldsymbol{\upsilon_{max}}$ (film): 3315 (br.), 2958, 2874, 1744, 1657, 1627, 1524, 1206, 1156 cm^{-1}.

^{1}**H NMR** (500 MHz, $CDCl_3$): δ 6.39 – 6.31 (m, 1H), 6.22 – 6.16 (m, 1H), 4.74 – 4.64 (m, 1H), 3.72 (s, 3H), 3.19 – 3.09 (m, 1H), 2.50 – 2.20 (m, 4H), 2.16 – 2.08 (m, 1H), 1.88 – 1.82 (m, 1H), 1.71 – 1.61 (m, 2H), 1.61 – 1.53 (m, 2H), 1.53 – 1.44 (m, 1H), 1.41 – 1.32 (m, 1H), 1.08 – 0.99 (m, 1H), 0.98 – 0.90 (m, 9H).

^{13}**C NMR** (126 MHz, $CDCl_3$): δ 173.9, 168.0, 167.8, 137.1, 137.0, 135.7, 135.5, 52.4, 50.9, 50.8, 46.5, 46.5, 41.8, 38.2, 37.4, 37.3, 36.3, 36.3, 28.2, 28.2, 27.9, 27.9, 26.1, 26.1, 25.1, 25.1, 22.9, 22.9, 22.1, 11.4, 11.4.

HRMS: exact mass calculated for $[M+Na]^+$ ($C_{19}H_{29}NO_4Na$) requires *m/z* 358.1989, found *m/z* 358.1989.

Compound 20.

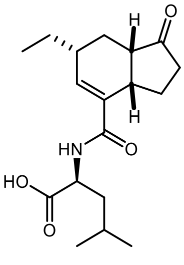

20

To a round bottom flask was added compound **S39 (**63 mg, 0.19 mmol). The material was suspended in 3 M HCl (1.5 mL) and the resulting suspension brought to 80 °C for 3 h. The reaction was then allowed to cool to room temperature and diluted with EtOAc (5 mL). The layers were separated and the aqueous phase washed twice more with EtOAc (2 x 5 mL). The organics were combined, dried over Na_2SO_4, filtered and evaporated to afford a white gum. The crude material was loaded in

a solution of EtOAc and purified by flash silica column chromatography, eluent 1% AcOH/EtOAc to afford the title compound as a colourless oil (32 mg, 53%).

TLC (1% AcOH/EtOAc): R_f= 0.48 stained by $KMnO_4$.

υ_{max} (film): 3319 (br.), 2958, 2926, 2874, 1733, 1653, 1615, 1526, 1195, 1150 cm^{-1}.

^{1}H NMR (500 MHz, $CDCl_3$): δ 9.22 (br. s, 1H), 6.42 – 6.35 (m, 1H), 6.30 – 6.22 (m, 1H), 4.74 – 4.64 (m, 1H), 3.21 – 3.11 (m, 1H), 2.51 – 2.24 (m, 4H), 2.19 – 2.11 (m, 1H), 1.91 – 1.84 (m, 1H), 1.80 – 1.68 (m, 2H), 1.68 – 1.55 (m, 2H), 1.55 – 1.47 (m, 1H), 1.43 – 1.34 (m, 1H), 1.11 – 1.01 (m, 1H), 1.01 – 0.94 (m, 9H).

^{13}C NMR (126 MHz, $CDCl_3$): δ 176.9, 176.8, 168.7, 168.5, 137.8, 137.7, 135.4, 135.3, 51.2, 51.1, 46.6, 46.5, 41.4, 41.3, 38.2, 37.5, 37.4, 36.3, 28.2, 28.2, 27.9, 27.8, 26.1, 26.1, 25.2, 25.2, 23.0, 23.0, 22.1, 22.0, 11.5, 11.4.

HRMS: exact mass calculated for $[M+H]^+$ ($C_{18}H_{28}NO_4$) requires *m/z* 322.2013, found *m/z* 322.2016.

The spectral data were consistent with those previously reported in the literature.[14]

Compound S40.

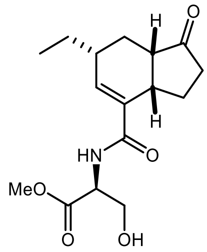

S40

To a 2-dram vial was added (±)-CFA (**2**) (30 mg, 0.13 mmol, 1 equiv.) and COMU (123 mg, 0.17 mmol, 1.2 equiv.). DMF (0.7 mL) was added, followed by DIPEA (80 µL, 0.46 mmol, 3 equiv.) and the resulting solution stirred at room temperature under air for 5 minutes. *L*-serine methyl ester hydrochloride (34 mg, 0.21 mmol, 1.5 equiv.) was then added portionwise and the reaction stirred for 16 h. The yellow solution was diluted with H_2O (15 mL) and extracted with EtOAc (3 x 10 mL). The organics were combined, washed with brine (10 mL), dried over Na_2SO_4, filtered, and evaporated to afford a red oil. The crude material was loaded in a solution of EtOAc and purified by flash silica column chromatography, eluent 1% MeOH/EtOAc to afford a red oil. The material was taken up in Et_2O, and petroleum ether added until a solid precipitated. The solvent was removed by Pasteur pipette and the solid dried under vacuum to afford the title compound as a pale red solid (22 mg, 49%).

TLC (1% MeOH/EtOAc): R_f= 0.45 stained by $KMnO_4$ and faintly visible by UV (short wave).

υ_{max} (film): 3387 (br.), 2955, 1736, 1655, 1618, 1522, 1209 cm^{-1}.

^{1}H NMR (500 MHz, $CDCl_3$): δ 6.77 – 6.69 (m, 1H), 6.52 – 6.42 (m, 1H), 4.78 – 4.71 (m, 1H), 4.06 – 3.94 (m, 2H), 3.81 (s, 3H), 3.21 – 3.12 (m, 1H), 2.78 – 2.54 (m, 1H), 2.54 – 2.24 (m, 4H), 2.21 – 2.12 (m, 1H), 1.92 – 1.85 (m, 1H), 1.66 – 1.57 (m, 1H), 1.56 – 1.47 (m, 1H), 1.44 – 1.34 (m, 1H), 1.11 – 1.02 (m, 1H), 0.98 (t, *J* = 7.4 Hz, 3H).

^{13}C NMR (101 MHz, $CDCl_3$): δ 220.3, 220.2, 171.3, 168.5, 168.3, 138.4, 137.8, 135.3, 134.9, 63.8, 63.6, 55.0, 55.0, 53.0, 46.7, 46.6, 38.2, 37.5, 36.3, 28.2, 28.0, 26.1, 26.1, 11.4.

HRMS: exact mass calculated for $[M+H]^+$ ($C_{16}H_{24}NO_5$) requires *m/z* 310.1649, found *m/z* 310.1651.

Compound 21.

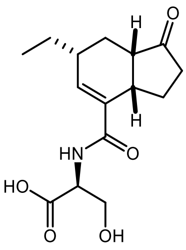

21

Prepared according to General Procedure G using compound **S40** (26 mg, 0.08 mmol, 1 equiv.), LiOH (4 mg, 0.17 mmol, 2 equiv.), and 1:1 MeOH:H_2O (2 mL). After 16 h at 50 °C the reaction mixture was subjected to purification outlined in General Procedure G to afford the title compound as a white solid (4 mg, 18%).

υ_{max} (film): 3356 (br.), 2953, 2924, 2855, 1734, 1709, 1228, 1057 cm^{-1}.

^{1}H NMR (500 MHz, MeOD): δ 6.61 – 6.54 (m, 1H), 4.60 – 4.52 (m, 1H), 4.00 – 3.94 (m, 1H), 3.92 – 3.86 (m, 1H), 3.25 – 3.17 (m, 1H), 2.49 – 2.30 (m, 4H), 2.24 – 2.15 (m, 1H), 1.85 – 1.78 (m, 1H), 1.69 – 1.59 (m, 1H), 1.59 – 1.50 (m, 1H), 1.48 – 1.40 (m, 1H), 1.20 – 1.10 (m, 1H), 1.02 (t, *J* = 7.4 Hz, 3H). CO_2H, O*H*, and N*H* not observed.

^{13}C NMR (101 MHz, MeOD): δ 222.9, 222.8, 170.9, 170.5, 139.0, 138.6, 136.5, 136.2, 63.0, 63.0, 47.9, 47.9, 38.8, 38.7, 37.4, 37.3, 29.1, 28.8, 28.8, 27.1, 11.6, 11.6.

HRMS: exact mass calculated for $[M-H]^-$ ($C_{15}H_{20}NO_5$) requires *m/z* 294.1347, found *m/z* 294.1343.

Compound S41.

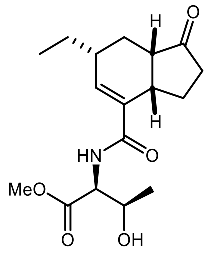

S41

To a round bottom flask was added (±)-CFA (**2**) (30 mg, 0.14 mmol, 1 equiv.) and COMU (80 mg, 0.19 mmol, 1.2 equiv.). DMF (0.7 mL) was added, followed by DIPEA (80 µL, 0.46 mmol, 3 equiv.) and the resulting solution stirred at room temperature under air for 5 minutes. *L*-threonine methyl ester hydrochloride (37 mg, 0.22 mmol, 1.5 equiv.) was then added and the reaction stirred for 16 h. The red solution was diluted with H_2O (15 mL) and extracted with EtOAc (3 x 10 mL). The organics were combined, washed with brine (10 mL), dried over Na_2SO_4, filtered, and evaporated to afford a red oil. The crude material was loaded in a solution of 40% EtOAc/CH_2Cl_2 and purified by flash silica column chromatography, eluent 40-50% EtOAc/CH_2Cl_2 to afford a pale yellow oil which was taken up in Et_2O and petroleum ether added until a precipitate formed. The solvent was removed by Pasteur pipette and the residue dried under vacuum to afford the title compound as a white solid (21 mg, 64%).

TLC (40% EtOAc/CH_2Cl_2): R_f = 0.17 stained by $KMnO_4$.

υ_{max} (film): 3376 (br.), 2956, 2930, 2872, 2855, 1736, 1654, 1619, 1513, 1210, 1151 cm^{-1}.

^{1}H NMR (500 MHz, $CDCl_3$): δ 6.56 – 6.51 (m, 1H), 6.48 – 6.44 (m, 1H), 4.72 – 4.65 (m, 1H), 4.44 – 4.37 (m, 1H), 3.80 – 3.76 (m, 3H), 3.23 – 3.14 (m, 1H), 2.53 – 2.25 (m, 5H), 2.20 – 2.13 (m, 1H), 1.92 – 1.86 (m, 1H), 1.68 – 1.57 (m, 1H), 1.57 – 1.47 (m, 1H), 1.43 – 1.34 (m, 1H), 1.27 – 1.22 (m, 3H), 1.12 – 1.02 (m, 1H), 0.98 (t, *J* = 7.4 Hz, 3H).

^{13}**C NMR** (101 MHz, $CDCl_3$): δ 220.4, 220.3, 171.8, 171.8, 168.7, 168.5, 137.8, 137.7, 135.4, 135.3, 68.2, 57.3, 52.8, 46.6, 38.2, 37.5, 37.5, 36.4, 36.3, 28.2, 28.0, 27.9, 26.1, 20.3, 20.2, 11.5.
HRMS: exact mass calculated for $[M+H]^+$ ($C_{17}H_{26}NO_5$) requires *m/z* 324.1805, found *m/z* 324.1807.

Compound 22.

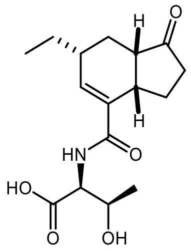

22

Prepared according to General Procedure G using compound **S41** (20 mg, 0.06 mmol, 1 equiv.), LiOH (5 mg, 0.21 mmol, 3 equiv.) and 1:1 MeOH:H_2O (4 mL). After 16 h the reaction was subjected to purification outlined in General Procedure G to afford the title compound as a white solid (12 mg, 63%).
υ_{max} (film): 3374 (br.), 2959, 2920, 2853, 1730, 1651, 1607, 1524 cm^{-1}.
^{1}**H NMR** (500 MHz, $CDCl_3$): δ 6.80 (s, 1H), 6.53 (s, 1H), 5.39 – 4.32 (m, 3H), 3.23 – 3.08 (m, 1H), 2.54 – 2.25 (m, 4H), 2.17 (br. s, 1H), 1.95 – 1.84 (m, 1H), 1.67 – 1.47 (m, 2H), 1.44 – 1.34 (m, 1H), 1.26 (br. s, 3H), 1.13 – 1.02 (m, 1H), 0.98 (t, *J* = 6.9 Hz, 3H). O*H* not observed.
^{13}**C NMR** (101 MHz, $CDCl_3$): δ 220.2, 220.1, 169.7, 169.4, 139.4, 139.0, 134.7, 67.5, 67.2, 46.7, 46.6, 38.2, 37.6, 37.5, 36.3, 36.2, 28.2, 28.1, 27.9, 26.0, 19.5, 11.5, 11.4.
HRMS: exact mass calculated for $[M-H]^-$ ($C_{16}H_{22}NO_5$) requires *m/z* 308.1503, found *m/z* 308.1496.

Compound S42.

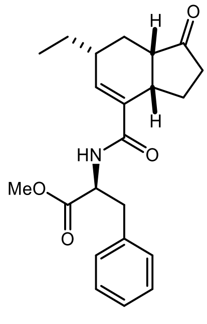

S42

Prepared according to General Procedure F using (±)-CFA (**2**) (30 mg, 0.14 mmol, 1 equiv.), HATU (66 mg, 0.17 mmol, 1.2 equiv.), *L*-phenylalanine methyl ester hydrochloride (47 mg, 0.22 mmol, 1.5 equiv.), DIPEA (80 µL, 0.46 mmol, 3 equiv.) and DMF (0.7 mL). After 16 h the reaction was subjected to purification outlined in General Procedure F (silica gel, 40% EtOAc/petroleum ether to afford the title compound as a white gum (42 mg, 79%).
TLC (60% EtOAc/petroleum ether): R_f= 0.48 stained by $KMnO_4$.
υ_{max} (film): 3317 (br.), 2960, 2932, 2876, 2859, 1740, 1658, 1625, 1526, 1215, 705 cm^{-1}.
^{1}**H NMR** (500 MHz, $CDCl_3$): δ 7.33 – 7.23 (m, 3H), 7.14 – 7.08 (m, 2H), 6.33 – 6.23 (m, 1H), 6.19 – 6.11 (m, 1H), 4.98 – 4.92 (m, 1H), 3.79 – 3.75 (m, 3H), 3.26 – 3.02 (m, 3H), 2.44 – 2.20 (m, 4H), 2.16 – 2.07 (m, 1H), 1.90 – 1.83 (m, 1H), 1.64 – 1.31 (m, 3H), 1.10 – 0.98 (m, 1H), 0.96 (t, *J* = 7.4 Hz, 3H).

^{13}C NMR (126 MHz, $CDCl_3$): δ 172.3, 167.6, 167.5, 137.3, 137.2, 136.0, 135.6, 135.3, 129.4, 129.4, 128.7, 128.7, 127.4, 127.3, 53.2, 52.5, 52.5, 46.6, 46.5, 38.2, 37.9, 37.8, 37.4, 37.3, 36.3, 36.2, 28.1, 28.0, 27.7, 26.2, 26.1, 11.4, 11.3.
HRMS: exact mass calculated for $[M+H]^+$ ($C_{22}H_{28}NO_4$) requires *m/z* 370.2013, found *m/z* 370.2013.

Compound 23.

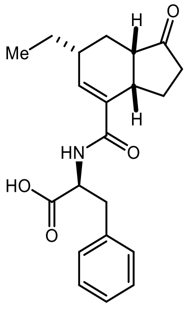

23

Prepared according to General Procedure G using compound **S42** (36 mg, 0.10 mmol, 1 equiv.), LiOH (8 mg, 0.33 mmol, 3 equiv.), and 1:1 MeOH:H_2O (5 mL) and the resulting suspension brought to 40 °C for 16 h. The reaction was allowed to cool to room temperature, acidified with AcOH and the organics extracted with EtOAc (3 x 10 mL). The organics were combined, dried over Na_2SO_4, filtered, and evaporated to afford a pale yellow oil. The crude material was loaded in a solution of CH_2Cl_2 and was purified by flash silica column chromatography, eluent 1% AcOH, 30% EtOAc/CH_2Cl_2 to afford the title compound as a colourless oil which solidified to a white solid on standing (11 mg, 32%).
TLC (1% AcOH, 30% EtOAc/CH_2Cl_2): $R_f = 0.15$ stained by $KMnO_4$.
υ_{max} (film): 3312 (br.), 2957, 2924, 2855, 1726, 1719, 1653, 1611, 1522, 1211 cm^{-1}.
^{1}H NMR (400 MHz, $CDCl_3$): δ 7.33 – 7.23 (m, 3H), 7.22 – 7.14 (m, 2H), 6.34 – 6.20 (m, 2H), 5.31 – 4.45 (m, 2H), 3.36 – 3.24 (m, 1H), 3.22 – 3.09 (m, 1H), 3.09 – 2.95 (m, 1H), 2.39 – 2.02 (m, 5H), 1.88 – 1.77 (m, 1H), 1.55 – 1.20 (m, 3H), 1.07 – 0.95 (m, 1H), 0.92 (t, $J = 7.3$ Hz, 3H).
^{13}C NMR (101 MHz, $CDCl_3$): δ 220.3, 220.2, 170.7, 168.4, 138.5, 138.0, 136.2, 135.0, 135.0, 129.5, 128.9, 128.8, 127.5, 127.4, 46.6, 46.5, 38.2, 37.5, 37.3, 36.3, 36.1, 28.1, 28.0, 27.6, 26.1, 26.0, 11.4, 11.3.
HRMS: exact mass calculated for $[M-H]^-$ ($C_{21}H_{24}NO_4$) requires *m/z* 354.1711, found *m/z* 354.1706.
The spectral data were consistent with those previously reported in the literature.[14]

Compound S43.

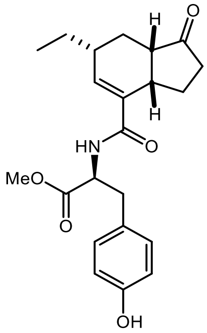

S43

Prepared according to General Procedure F using (±)-CFA (**2**) (30 mg, 0.14 mmol, 1 equiv.), HATU (66 mg, 0.17 mmol, 1.2 equiv.), *L*-tyrosine methyl ester (42 mg, 0.22 mmol, 1.5 equiv.), DIPEA (50 µL, 0.29 mmol, 2 equiv.), and DMF (0.7 mL).

After 16 h the reaction was subjected to purification outlined in General Procedure F (silica gel, 60-70% EtOAc/petroleum ether) to afford the title compound as a pale yellow gum (47 mg, 85%).

TLC (70% EtOAc/petroleum ether): $R_f = 0.43$ stained by $KMnO_4$.

υ_{max} (film): 3312 (br.), 2959, 2923, 2857, 1732, 1654, 1614, 1515, 1213 cm^{-1}.

^{1}H NMR (500 MHz, $CDCl_3$): δ 6.97 – 6.91 (m, 2H), 6.76 – 6.71 (m, 2H), 6.46 (br. s, 1H), 6.38 – 6.27 (m, 1H), 6.27 – 6.20 (m, 1H), 4.93 – 4.87 (m, 1H), 3.78 – 3.74 (m, 3H), 3.17 – 3.02 (m, 3H), 2.41 – 2.19 (m, 4H), 2.16 – 2.07 (m, 1H), 1.88 – 1.82 (m, 1H), 1.59 – 1.40 (m, 2H), 1.40 – 1.32 (m, 1H), 1.09 – 0.98 (m, 1H), 0.95 (t, J = 7.4 Hz, 3H).

^{13}C NMR (126 MHz, $CDCl_3$): δ 172.6, 168.0, 167.8, 155.6, 155.6, 137.9, 137.7, 135.4, 135.1, 130.5, 127.3, 115.7, 115.7, 53.5, 52.7, 52.6, 46.7, 46.5, 38.2, 37.4, 37.4, 37.2, 37.2, 36.3, 36.2, 28.1, 28.0, 27.7, 26.2, 26.0, 11.4, 11.3.

HRMS: exact mass calculated for $[M+H]^+$ ($C_{22}H_{28}NO_5$) requires m/z 386.1962, found m/z 386.1961.

Compound 24.

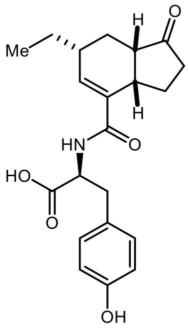

12d

Prepared according to General Procedure G using compound **S43** (30 mg, 0.07 mmol, 1 equiv.), LiOH (10 mg, 0.42 mmol, 3 equiv.), and 1:1 MeOH:H_2O (5 mL). After 16 h the reaction was allowed to cool to room temperature, acidified with AcOH and the organics extracted with EtOAc (3 x 10 mL). The organics were combined, dried over Na_2SO_4, filtered and evaporated to afford a pale yellow oil. The crude material was loaded in a solution of CH_2Cl_2 and purified by flash silica column chromatography, eluent 1% AcOH, 30-50% EtOAc/CH_2Cl_2 to afford the title compound as a colourless oil which solidified to a white solid on standing (15 mg, 52%).

TLC (1% AcOH, 30% EtOAc/CH_2Cl_2): $R_f = 0.13$ stained by $KMnO_4$.

υ_{max} (film): 3289 (br.), 2961, 2924, 2855, 1719, 1653, 1611, 1514 cm^{-1}.

^{1}H NMR (500 MHz, MeOD): δ 7.10 – 6.99 (m, 2H), 6.72 – 6.64 (m, 2H), 6.41 – 6.28 (m, 1H), 4.74 – 4.57 (m, 1H), 3.24 – 3.14 (m, 1H), 3.13 – 3.02 (m, 1H), 3.02 – 2.92 (m, 1H), 2.38 – 2.07 (m, 5H), 1.80 – 1.72 (m, 1H), 1.54 – 1.35 (m, 3H), 1.15 – 1.04 (m, 1H), 0.99 (t, J = 7.4 Hz, 3H). CO_2*H*, O*H*, and N*H* not observed.

^{13}C NMR (101 MHz, MeOD): δ 222.9, 222.9, 157.3, 157.2, 138.3, 137.9, 136.6, 131.4, 129.6, 116.1, 116.1, 47.8, 38.7, 38.6, 38.5, 37.7, 37.6, 37.3, 37.3, 29.1, 28.7, 28.5, 27.2, 27.0, 11.6, 11.6.

HRMS: exact mass calculated for $[M-H]^-$ ($C_{21}H_{24}NO_5$) requires m/z 370.1660, found m/z 370.1655.

The spectral data were consistent with those previously reported in the literature.[14]

Compound S44.

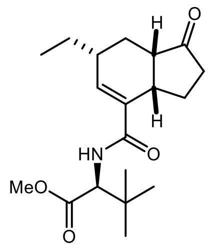

S44

Prepared according to General Procedure F using (±)-CFA (**2**) (20 mg, 0.10 mmol, 1 equiv.), HATU (44 mg, 0.12 mmol, 1.2 equiv.), methyl (*S*)-2-amino-3,3-dimethylbutanoate hydrochloride (26 mg, 0.14 mmol, 1.5 equiv.), DIPEA (50 µL, 0.30 mmol, 3 equiv), and DMF (0.5 mL). After 16 h the reaction was subjected to purification outlined in General Procedure F (silica gel, 10% EtOAc/CH_2Cl_2) to afford the title compound as a pale orange oil (29 mg, 90%).

TLC (10% EtOAc/CH_2Cl_2): R_f = 0.14 stained by $KMnO_4$.

$\boldsymbol{\upsilon}_{max}$ (film): 3359 (br.), 2960, 2874, 1738, 1662, 1627, 1509, 1216, 1165 cm^{-1}.

^{1}H NMR (400 MHz, $CDCl_3$): δ 6.36 (s, 1H), 6.32 – 6.22 (m, 1H), 4.59 – 4.52 (m, 1H), 3.74 (s, 3H), 3.22 – 3.09 (m, 1H), 2.52 – 2.22 (m, 4H), 2.20 – 2.09 (m, 1H), 1.92 – 1.84 (m, 1H), 1.66 – 1.47 (m, 2H), 1.43 – 1.34 (m, 1H), 1.12 – 1.01 (m, 1H), 1.01 – 0.93 (m, 12H).

^{13}C NMR (101 MHz, $CDCl_3$): δ 220.2, 172.6, 172.5, 167.9, 167.8, 137.1, 136.9, 135.9, 135.8, 60.0, 59.9, 52.0, 46.6, 38.2, 37.5, 37.4, 36.4, 35.3, 35.1, 28.3, 28.2, 28.0, 27.9, 26.8, 26.8, 26.2, 26.1, 11.5, 11.4.

HRMS: exact mass calculated for $[M+H]^+$ ($C_{19}H_{30}NO_4$) requires *m/z* 336.2169, found *m/z* 336.2169.

Compound 25.

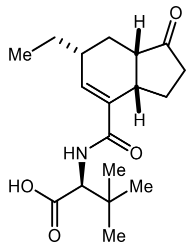

25

Prepared according to General Procedure G using compound **S44** (24 mg, 0.07 mmol, 1 equiv.), NaOH (9 mg, 0.23 mmol, 3 equiv.), and 1:1 MeOH:H_2O (3 mL). After 16 h the reaction was subjected to purification outlined in General Procedure G to afford the title compound as a pale orange solid (18 mg, 78%).

$\boldsymbol{\upsilon}_{max}$ (film): 3343 (br.), 2963, 2876, 1733, 1658, 1616, 1515, 1213 cm^{-1}.

^{1}H NMR (500 MHz, $CDCl_3$): δ 6.43 – 6.37 (m, 1H), 6.37 – 6.29 (m, 1H), 4.59 – 4.50 (m, 1H), 3.22 – 3.10 (m, 1H), 2.51 – 2.24 (m, 4H), 2.15 (br. s, 1H), 1.93 – 1.85 (m, 1H), 1.67 – 1.48 (m, 2H), 1.44 – 1.34 (m, 1H), 1.11 – 1.02 (m, 10H), 1.01 – 0.94 (m, 3H). CO_2H not observed.

^{13}C NMR (101 MHz, $CDCl_3$): δ 220.5, 175.3, 168.4, 168.3, 137.7, 137.4, 135.6, 60.4, 46.6, 38.2, 37.5, 37.4, 36.4, 35.0, 34.9, 28.2, 28.2, 28.0, 27.9, 26.9, 26.8, 26.1, 26.1, 11.5, 11.4.

HRMS: exact mass calculated for $[M-H]^-$ ($C_{18}H_{26}NO_4$) requires *m/z* 320.1867, found *m/z* 320.1860.

Compound S45.

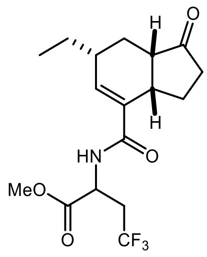

S45

Prepared according to General Procedure F using (±)-CFA (**2**) (20 mg, 0.10 mmol, 1 equiv.), HATU (44 mg, 0.12 mmol, 1.2 equiv.), methyl 2-amino-4,4,4-trifluorobutanoate hydrochloride (30 mg, 0.14 mmol, 1.5 equiv.), DIPEA (50 µL, 0.30 mmol, 3 equiv), and DMF (0.5 mL). After 16 h the reaction was subjected to purification outlined in General Procedure F (silica gel, 20% EtOAc/CH_2Cl_2) to afford the title compound as a colourless oil (24 mg, 69%).

TLC (20% EtOAc/CH_2Cl_2): R_f= 0.61 stained by $KMnO_4$.

υ_{max} (film): 3802 (br.), 2963, 2870, 1705, 1532, 1364, 1165 cm^{-1}.

^{1}H NMR (400 MHz, $CDCl_3$): δ 6.52 – 6.47 (m, 1H), 6.44 – 6.37 (m, 1H), 4.93 – 4.83 (m, 1H), 3.82 (s, 3H), 3.23 – 3.08 (m, 1H), 2.96 – 2.84 (m, 1H), 2.81 – 2.67 (m, 1H), 2.52 – 2.22 (m, 4H), 2.22 – 2.11 (m, 1H), 1.94 – 1.85 (m, 1H), 1.64 – 1.46 (m, 2H), 1.46 – 1.33 (m, 1H), 1.13 – 1.01 (m, 1H), 0.98 (t, J = 7.4 Hz, 3H).

^{13}C NMR (101 MHz, $CDCl_3$): δ 220.1, 220.1, 170.6, 167.8, 167.8, 138.1, 138.0, 135.2, 125.8 (q, $^1J_{C\text{-}F}$ = 277.8 Hz), 53.3, 47.6, 46.6, 46.5, 38.2, 37.5, 37.4, 36.3, 36.2, 35.2 (q, $^2J_{C\text{-}F}$ = 28.2 Hz), 28.2, 27.9, 27.8, 26.1, 26.1, 11.4, 11.4.

^{19}F NMR (376 MHz, $CDCl_3$): δ −62.85 (t, J = 10.4 Hz), −62.96 (t, J = 10.4 Hz) (1:1 diastereosiomers).

HRMS: exact mass calculated for $[M+H]^+$ ($C_{17}H_{23}F_3NO_4$) requires m/z 362.1574, found m/z 362.1574.

Compound 26.

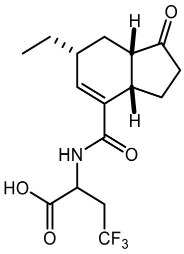

26

Prepared according to General Procedure G using compound **S45** (24 mg, 0.07 mmol, 1 equiv.), NaOH (10 mg, 0.25 mmol, 3 equiv.), and 1:1 MeOH:H_2O (3 mL). After 16 h the reaction was subjected to purification outlined in General Procedure G to afford the title compound as a pale orange solid (18 mg, 74%).

υ_{max} (film): 3339 (br.), 2967, 2930, 2880, 1740, 1718, 1653, 1617, 1523, 1247, 1133 cm^{-1}.

^{1}H NMR (500 MHz, $CDCl_3$): δ 7.22 (br. s, 1H), 6.67 (s, 1H), 6.45 (s, 1H), 4.83 (br. s, 1H), 3.20 – 3.08 (m, 1H), 3.00 – 2.84 (m, 1H), 2.78 (br. s, 1H), 2.49 – 2.24 (m, 4H), 2.17 (br. s, 1H), 1.93 – 1.84 (m, 1H), 1.66 – 1.47 (m, 2H), 1.43 – 1.34 (m, 1H), 1.13 – 1.01 (m, 1H), 0.98 (t, J = 6.7 Hz, 3H).

^{13}C NMR (101 MHz, $CDCl_3$): δ 220.6, 173.2 – 172.6 (m), 168.7, 168.6, 139.2, 138.7, 134.8, 134.8, 125.9 (q, $^1J_{C\text{-}F}$ = 277.8 Hz), 48.3 – 47.7 (m), 46.6, 46.5, 38.2, 37.5, 37.5, 36.2, 36.1, 35.4 – 34.3 (m), 28.4, 28.1, 27.9, 27.7, 26.0, 26.0, 11.4, 11.3.

^{19}F NMR (376 MHz, $CDCl_3$): δ −62.90 (t, J = 10.0 Hz), −63.00 (t, J = 10.1 Hz). (1:1 mixture of diastereoisomers).

HRMS: exact mass calculated for $[M-H]^-$ ($C_{16}H_{19}F_3NO_4$) requires m/z 346.1272, found m/z 346.1264.

Compound 27.

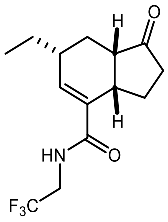

27

Prepared according to General Procedure F using (±)-CFA (**2**) (20 mg, 0.1 mmol, 1 equiv.), HATU (44 mg, 0.12 mmol, 1.2 equiv.), methyl 2-amino-3,3,3-trifluoropropanoate hydrochloride (50 mg, 0.26 mmol, 2.6 equiv.), DIPEA (50 µL, 0.30 mmol, 3 equiv), and DMF (0.5 mL). After 16 h the reaction was subjected to purification outlined in General Procedure F (silica gel, 20% EtOAc/CH_2Cl_2) to afford the title compound as a colourless oil (7 mg, 25%).

TLC (20% EtOAc/CH_2Cl_2): R_f = 0.54 stained by $KMnO_4$.

υ_{max} (film): 3330 (br.), 2963, 2926, 2880, 2861, 1740, 1662, 1632, 1532, 1256 cm^{-1}.

^{1}H NMR (400 MHz, $CDCl_3$): δ 6.36 (s, 1H), 6.00 (br. s, 1H), 4.12 – 3.88 (m, 2H), 3.24 – 3.14 (m, 1H), 2.51 – 2.24 (m, 4H), 2.23 – 2.13 (m, 1H), 1.91 (dt, *J* = 13.0, 4.8 Hz, 1H), 1.64 – 1.46 (m, 2H), 1.45 – 1.34 (m, 1H), 1.13 – 1.02 (m, 1H), 0.99 (t, *J* = 7.4 Hz, 3H).

^{13}C NMR (101 MHz, $CDCl_3$): δ 220.0, 168.1, 137.8, 135.6, 124.3 (q, $^1J_{C\text{-}F}$ = 278.6 Hz), 46.44, 40.9 (q, $^2J_{C\text{-}F}$ = 34.5 Hz), 38.3, 37.5, 36.4, 28.2, 27.9, 26.1, 11.4.

^{19}F NMR (376 MHz, $CDCl_3$): δ -72.45 (t, *J* = 9.1 Hz).

HRMS: exact mass calculated for $[M+H]^+$ ($C_{14}H_{19}F_3NO_2$) requires *m/z* 290.1362, found *m/z* 290.1365.

Compound S46.

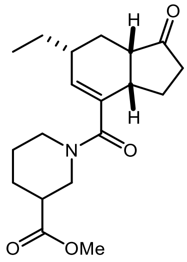

S46

Prepared according to General Procedure F using (±)-CFA (**2**) (20 mg, 0.10 mmol, 1 equiv.), HATU (44 mg, 0.12 mmol, 1.2 equiv.), methyl piperidine-3-carboxylate hydrochloride (26 mg, 0.14 mmol, 1.5 equiv.), DIPEA (50 µL, 0.30 mmol, 3 equiv), and DMF (0.5 mL). After 16 h the reaction was subjected to purification outlined in General Procedure F (silica gel, 30-50% EtOAc/CH_2Cl_2) to afford the title compound as a colourless oil (17 mg, 53%).

TLC (20% EtOAc/CH_2Cl_2): R_f = 0.12 stained by $KMnO_4$.

υ_{max} (film): 3445 (br.), 2956, 2939, 2922, 2902, 2872, 2855, 1738, 1619, 1435, 1245 cm^{-1}.

^{1}H NMR (500 MHz, $CDCl_3$): δ 5.74 – 5.67 (m, 1H), 4.36 (br. s, 2H), 3.71 – 3.66 (m, 3H), 3.24 – 2.77 (m, 3H), 2.53 – 2.31 (m, 3H), 2.31 – 2.19 (m, 2H), 2.17 – 2.07 (m, 2H), 1.91 – 1.84 (m, 1H), 1.83 – 1.62 (m, 3H), 1.53 – 1.34 (m, 3H), 1.19 – 1.09 (m, 1H), 0.94 (t, *J* = 7.4 Hz, 3H).

^{13}C NMR (101 MHz, $CDCl_3$): δ 220.2, 173.5, 170.8, 170.7, 134.6, 133.0, 52.0, 52.0, 46.3, 46.3, 42.0 (br.), 38.4, 38.3, 37.6, 37.4, 36.7, 36.6, 28.3, 27.6, 27.3, 27.2, 26.2, 26.1, 25.0 (br.), 11.2.

HRMS: exact mass calculated for $[M+H]^+$ ($C_{19}H_{28}NO_4$) requires *m/z* 334.2013, found *m/z* 334.2014.

Compound 28.

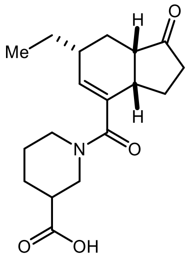

28

Prepared according to General Procedure G using compound **S46** (15 mg, 0.04 mmol, 1 equiv.), NaOH (5 mg, 0.13 mmol, 3 equiv.), and 1:1 MeOH:H_2O (2 mL). After 16 h the reaction was subjected to purification outlined in General Procedure G to afford the title compound as a white solid (9 mg, 63%).

υ_{max} (film): 2934 (br.), 2861 (br.), 1733, 1584, 1444, 1266, 1186, 917 cm^{-1}.

^{1}H NMR (500 MHz, $CDCl_3$): δ 5.75 – 5.68 (m, 1H), 4.51 – 3.71 (m, 2H), 3.41 – 3.01 (m, 3H), 2.59 – 2.31 (m, 3H), 2.30 – 2.18 (m, 2H), 2.12 (br. s, 2H), 1.92 – 1.84 (m, 1H), 1.84 – 1.61 (m, 3H), 1.58 – 1.33 (m, 3H), 1.20 – 1.07 (m, 1H), 0.94 (t, *J* = 7.4 Hz, 3H). CO_2H not observed.

^{13}C NMR (101 MHz, $CDCl_3$): δ 220.3, 177.3, 171.2, 171.0, 134.4, 133.3, 133.0, 46.3, 46.3, 38.4, 37.6, 37.4, 36.7, 36.7, 28.3, 27.4, 27.3, 27.2, 26.2, 26.1, 11.3.

HRMS: exact mass calculated for $[M-H]^-$ ($C_{18}H_{24}NO_4$) requires *m/z* 318.1711, found *m/z* 318.1706.

Compound S47.

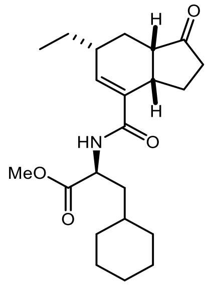

S47

Prepared according to General Procedure F using (±)-CFA (**2**) (20 mg, 0.10 mmol, 1 equiv.), HATU (44 mg, 0.12 mmol, 1.2 equiv.), methyl (*S*)-2-amino-3-cyclohexylpropanoate hydrochloride (32 mg, 0.14 mmol, 1.5 equiv.), DIPEA (50 µL, 0.30 mmol, 3 equiv), and DMF (0.5 mL). After 16 h the reaction was subjected to purification outlined in General Procedure F (silica gel, 20% EtOAc/CH_2Cl_2) to afford the title compound as a colourless oil (33 mg, 91%).

TLC (30% EtOAc/CH_2Cl_2): R_f = 0.49 stained by $KMnO_4$.

υ_{max} (film): 3322 (br.), 2919, 2850, 1738, 1656, 1619, 1524, 1203, 1152 cm^{-1}.

^{1}H NMR (500 MHz, $CDCl_3$): δ 6.41 – 6.30 (m, 1H), 6.16 – 6.07 (m, 1H), 4.80 – 4.68 (m, 1H), 3.75 (s, 3H), 3.21 – 3.12 (m, 1H), 2.51 – 2.23 (m, 4H), 2.19 – 2.11 (m, 1H), 1.92 – 1.85 (m, 1H), 1.85 – 1.76 (m, 1H), 1.76 – 1.47 (m, 8H), 1.41 – 1.28 (m, 2H), 1.23 – 1.11 (m, 3H), 1.11 – 1.01 (m, 1H), 1.01 – 0.89 (m, 5H).

^{13}C NMR (101 MHz, $CDCl_3$): δ 220.4, 220.3, 174.1, 168.0, 167.8, 137.0, 136.9, 135.8, 135.7, 52.5, 50.3, 50.2, 46.6, 40.4, 40.4, 38.3, 37.5, 37.4, 36.4, 36.3, 34.5, 34.4, 33.6, 32.8, 32.7, 28.3, 28.2, 27.9, 27.9, 26.5, 26.3, 26.3, 26.2, 11.5, 11.4.

HRMS: exact mass calculated for $[M+H]^+$ ($C_{22}H_{34}NO_4$) requires *m/z* 376.2482, found *m/z* 376.2476.

Compound 29.

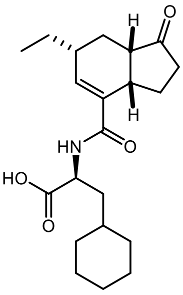

29

Prepared according to General Procedure G using compound **S47** (27 mg, 0.07 mmol, 1 equiv.), NaOH (9 mg, 0.23 mmol, 3 equiv.), and 1:1 MeOH:H_2O (4 mL). After 16 h the reaction was subjected to purification outlined in General Procedure G to afford the title compound as a white solid (18 mg, 69%).

υ_{max} (film): 3325 (br.), 2922, 2854, 1733, 1653, 1616, 1526, 1195, 1150 cm^{-1}.

^{1}H NMR (500 MHz, $CDCl_3$): δ 6.97 (br. s, 1H), 6.43 – 6.33 (m, 1H), 6.25 – 6.14 (m, 1H), 4.78 – 4.63 (m, 1H), 3.22 – 3.12 (m, 1H), 2.51 – 2.24 (m, 4H), 2.16 (br. s, 1H), 1.93 – 1.86 (m, 1H), 1.86 – 1.77 (m, 2H), 1.77 – 1.46 (m, 7H), 1.44 – 1.34 (m, 2H), 1.26 – 1.12 (m, 3H), 1.12 – 1.02 (m, 1H), 1.01 – 0.88 (m, 5H).

^{13}C NMR (126 MHz, $CDCl_3$): δ 176.9, 176.8, 168.7, 168.4, 137.7, 137.6, 135.4, 135.4, 50.6, 50.5, 46.5, 39.9, 39.8, 38.3, 37.5, 37.4, 36.3, 36.3, 34.5, 34.4, 33.6, 32.7, 32.6, 26.4, 26.3, 26.3, 26.2, 26.1, 11.5, 11.4.

HRMS: exact mass calculated for $[M+H]^+$ ($C_{21}H_{32}NO_4$) requires *m/z* 362.2326, found *m/z* 362.2327.

Compound S48.

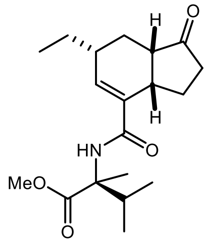

S48

Prepared according to General Procedure F using (±)-CFA (**2**) (20 mg, 0.10 mmol, 1 equiv.), HATU (44 mg, 0.12 mmol, 1.2 equiv.), methyl (*S*)-2-amino-2,3-dimethylbutanoate hydrochloride (26 mg, 0.14 mmol, 1.5 equiv.), DIPEA (50 µL, 0.30 mmol, 3 equiv.), and DMF (0.5 mL). After 16 h the reaction was subjected to purification outlined in General Procedure F (silica gel, 10% EtOAc/CH_2Cl_2) to afford the title compound as a colourless oil (26 mg, 81%).

TLC (10% EtOAc/CH_2Cl_2): R_f = 0.15 stained by $KMnO_4$.

υ_{max} (film): 3460 (br.), 2960, 2874, 2855, 1736, 1660, 1513, 1463, 1260, 1147 cm^{-1}.

^{1}H NMR (400 MHz, $CDCl_3$): δ 6.42 – 6.29 (m, 2H), 3.78 – 3.74 (m, 3H), 3.20 – 3.10 (m, 1H), 2.52 – 2.23 (m, 5H), 2.20 – 2.10 (m, 1H), 1.92 – 1.84 (m, 1H), 1.67 – 1.56 (m, 4H), 1.56 – 1.45 (m, 1H), 1.43 – 1.33 (m, 1H), 1.12 – 0.95 (m, 7H), 0.94 – 0.90 (m, 3H).

^{13}C NMR (101 MHz, $CDCl_3$): δ 220.4, 174.5, 174.4, 167.7, 167.6, 136.5, 136.5, 136.3, 136.3, 63.5, 63.4, 52.5, 52.5, 46.6, 46.6, 38.3, 37.4, 36.5, 36.4, 34.9, 28.3, 28.0, 27.9, 26.2, 18.9, 18.4, 17.8, 17.7, 17.7, 17.6, 11.4.

HRMS: exact mass calculated for $[M+H]^+$ ($C_{19}H_{30}NO_4$) requires *m/z* 336.2169, found *m/z* 336.2170.

Compound 30.

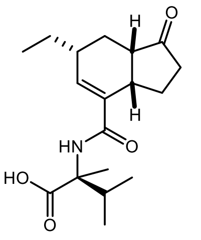

30

Prepared according to General Procedure G using compound **S48** (26 mg, 0.08 mmol, 1 equiv.), NaOH (9 mg, 0.23 mmol, 3 equiv.), and 1:1 MeOH:H_2O (4 mL). After 20 h the reaction was subjected to purification outlined in General Procedure G to afford the title compound as a white solid (17 mg, 68%).

υ_{max} (film): 3414 (br.), 2965, 2939, 2878, 1733, 1662, 1623, 1513, 1448, 1150 cm^{-1}.

^{1}H NMR (400 MHz, $CDCl_3$): δ 6.38 – 6.33 (m, 1H), 6.22 – 6.16 (m, 1H), 3.20 – 3.09 (m, 1H), 2.65 – 2.24 (m, 5H), 2.22 – 2.12 (m, 1H), 1.94 – 1.85 (m, 1H), 1.66 – 1.47 (m, 5H), 1.44 – 1.35 (m, 1H), 1.12 – 0.92 (m, 10H). CO_2H not observed.

^{13}C NMR (101 MHz, $CDCl_3$): δ 220.2, 220.1, 176.4, 175.9, 169.4, 169.1, 138.1, 137.5, 136.0, 135.9, 64.7, 64.2, 46.6, 46.5, 38.2, 37.5, 37.4, 36.4, 36.3, 33.3, 32.6, 28.2, 28.0, 27.9, 26.1, 26.1, 18.5, 18.3, 17.5, 17.2, 17.1, 11.4.

HRMS: exact mass calculated for $[M-H]^-$ ($C_{18}H_{26}NO_4$) requires *m/z* 320.1867, found *m/z* 320.1858.

Compound 31.

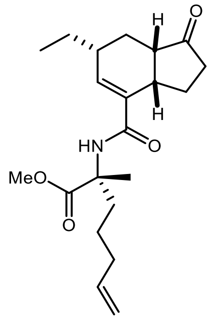

31

Prepared according to General Procedure F using (±)-CFA (**2**) (15 mg, 0.07 mmol, 1 equiv.), HATU (33 mg, 0.09 mmol, 1.2 equiv.), methyl (*R*)-2-amino-2-methylhept-6-enoate hydrochloride (25 mg, 0.12 mmol, 1.7 equiv.), DIPEA (40 µL, 0.23 mmol, 3 equiv.), and DMF (0.5 mL). After 16 h the reaction was subjected to purification outlined in General Procedure F (silica gel, 30% EtOAc/CH_2Cl_2) to afford the title compound as a white solid (5 mg, 20%).

TLC (30% EtOAc/CH_2Cl_2): R_f= 0.66 stained by $KMnO_4$.

υ_{max} (film): 3304, 3057, 2922, 2857, 1736, 1659, 1624, 1516, 1462, 1202, 1076 cm^{-1}.

^{1}H NMR (500 MHz, $CDCl_3$): δ 6.63 (s, 1H), 6.37 – 6.32 (m, 1H), 5.80 – 5.70 (m, 1H), 5.02 – 4.93 (m, 2H), 3.78 (s, 3H), 3.19 – 3.10 (m, 1H), 2.49 – 2.24 (m, 5H), 2.19 – 2.11 (m, 1H), 2.08 – 1.99 (m, 2H), 1.91 – 1.79 (m, 2H), 1.64 (s, 3H), 1.63 – 1.60 (m, 1H), 1.54 – 1.47 (m, 1H), 1.41 – 1.31 (m, 2H), 1.20 – 1.11 (m, 1H), 1.10 – 1.01(m, 1H), 0.98 (t, *J* = 7.4 Hz, 3H).

^{13}C NMR (101 MHz, $CDCl_3$): δ 175.5, 167.3, 167.2, 138.3, 138.2, 136.6, 136.4, 136.4, 136.3, 115.1, 60.7, 53.0, 46.7, 46.6, 38.3, 37.4, 36.4, 36.3, 36.0, 35.8, 33.5, 28.3, 27.9, 26.2, 23.9, 23.3, 23.3, 11.4.

HRMS: exact mass calculated for $[M+H]^+$ ($C_{21}H_{32}NO_4$) requires *m/z* 362.2326, found *m/z* 362.2328.

Compound 32.

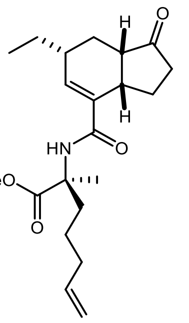

32

Prepared according to General Procedure F using (±)-CFA (**2**) (7 mg, 0.03 mmol, 1 equiv.), HATU (15 mg, 0.04 mmol, 1.2 equiv.), methyl (*S*)-2-amino-2-methylhept-6-enoate hydrochloride (10 mg, 0.05 mmol, 1.5 equiv.), DIPEA (20 µL, 0.10 mmol, 3 equiv.), and DMF (0.15 mL). After 16 h the reaction was subjected to purification outlined in General Procedure F (silica gel, 10% EtOAc/CH_2Cl_2) to afford the title compound as a white solid (4 mg, 29%). Compound tested as the methyl ester due to paucity of available material.

TLC (30% EtOAc/CH_2Cl_2): R_f = 0.68 stained by $KMnO_4$ and visible under UV (short wave).

υ_{max} (film): 3341 (br.), 2926, 2857, 1736, 1659, 1624, 1514, 1204, 1146, 1123 cm^{-1}.

^{1}H NMR (500 MHz, $CDCl_3$): δ 6.63 (s, 1H), 6.38 – 6.32 (m, 1H), 5.81 – 5.70 (m, 1H), 5.02 – 4.93 (m, 2H), 3.79 (s, 3H), 3.19 – 3.11 (m, 1H), 2.48 – 2.25 (m, 5H), 2.20 – 2.11 (m, 1H), 2.08 – 2.00 (m, 2H), 1.91 – 1.79 (m, 2H), 1.64 (s, 3H), 1.63 – 1.60 (m, 1H), 1.54 – 1.49 (m, 1H), 1.42 – 1.31 (m, 2H), 1.19 – 1.12 (m, 1H), 1.10 – 1.02 (m, 1H), 0.98 (t, *J* = 7.4 Hz, 3H).

^{13}C NMR (101 MHz, $CDCl_3$): δ 175.5, 138.3, 138.2, 136.6, 136.5, 136.4, 136.3, 115.1, 115.1, 60.7, 53.0, 46.7, 46.6, 38.3, 37.4, 36.4, 36.4, 36.0, 35.8, 33.5, 28.3, 27.9, 26.2, 23.9, 23.3, 23.3, 11.5.

HRMS: exact mass calculated for $[M+H]^+$ ($C_{21}H_{32}NO_4$) requires *m/z* 362.2326, found *m/z* 362.2328.

Compound S49

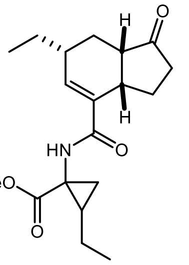

S49

Prepared according to General Procedure F using (±)-CFA (**2**) (22 mg, 0.1 mmol, 1 equiv.), HATU (44 mg, 0.12 mmol, 1.2 equiv.), compound **S62** (38 mg, 0.2 mmol, 2 equiv.), DIPEA (50 µL, 0.30 mmol, 3 equiv), and DMF (0.5 mL). After 16 h the reaction was subjected to purification outlined in General Procedure F (silica gel, 30% EtOAc/CH_2Cl_2) to afford the title compound as a colourless oil (7 mg, 20%).

TLC (30% EtOAc/CH_2Cl_2): R_f = 0.33 stained by $KMnO_4$.

υ_{max} (film): 3312 (br.), 2961, 2928, 2874, 1734, 1655, 1624, 1510, 1337 cm^{-1}.

^{1}H NMR (500 MHz, $CDCl_3$): δ 6.30 – 6.25 (m, 2H), 3.73 – 3.69 (m, 3H), 3.23 – 3.14 (m, 1H), 2.52 – 2.23 (m, 4H), 2.19 – 2.10 (m, 1H), 1.92 – 1.85 (m, 1H), 1.67 – 1.55 (m, 4H), 1.53 – 1.45 (m, 2H), 1.41 – 1.34 (m, 1H), 1.34 – 1.26 (m, 1H), 1.11 – 1.02 (m, 1H), 1.02 – 0.95 (m, 6H).

^{13}**C NMR** (101 MHz, $CDCl_3$): δ 220.4, 171.8, 169.4, 169.2, 136.6, 136.6, 136.2, 136.1, 52.5, 52.5, 46.5, 46.5, 38.4, 38.4, 38.3, 37.4, 36.4, 36.4, 33.3, 33.1, 28.3, 27.9, 27.8, 26.2, 26.2, 23.3, 23.1, 20.6, 13.6, 11.5.
HRMS: exact mass calculated for $[M+H]^+$ ($C_{19}H_{28}NO_4$) requires *m/z* 334.2013, found *m/z* 334.2016.

(±)-Coronatine (33).

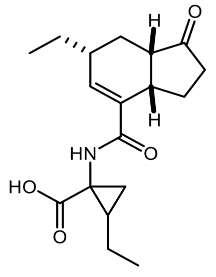

33

Prepared according to General Procedure G compound **S49** (20 mg, 0.10 mmol, 1 equiv.), LiOH (8 mg, 0.33 mmol, 3 equiv.), and 1:1 MeOH:H_2O (5 mL). After 16 h the reaction was allowed to cool to room temperature, acidified with AcOH, and the organics extracted with EtOAc (3 x 5 mL). The organics were combined, dried over Na_2SO_4, filtered, and evaporated to afford a colourless oil. The crude material was loaded in a solution of CH_2Cl_2 and purified by flash silica column chromatography, eluent 1% AcOH, 30% EtOAc/CH_2Cl_2 to afford a colourless oil. The crude material was dissolved in a minimal volume of diethyl ether and petroleum ether added until a white precipitate formed. The solvent was removed by Pasteur pipette and the residue dried under vacuum to afford the desired product as a white solid (9 mg, 47%).
TLC (1% AcOH, 30% EtOAc/CH_2Cl_2): R_f = 0.38 stained by $KMnO_4$.
υ_{max} (film): 3314 (br.), 2961, 2928, 2872, 1719, 1655, 1618, 1508, 1167 cm^{-1}.
^{1}**H NMR** (500 MHz, $CDCl_3$): δ 6.52 – 6.40 (m, 1H), 6.39 – 6.32 (m, 1H), 3.21 – 3.12 (m, 1H), 2.51 – 2.23 (m, 4H), 2.21 – 2.10 (m, 1H), 1.92 – 1.85 (m, 1H), 1.68 – 1.53 (m, 4H), 1.53 – 1.44 (m, 2H), 1.44 – 1.34 (m, 1H), 1.31 – 1.26 (m, 1H), 1.11 – 1.00 (m, 4H), 0.98 (t, *J* = 7.4 Hz, 3H). CO_2H not observed.
^{13}**C NMR** (101 MHz, $CDCl_3$): δ 220.2, 220.1, 174.9, 174.2, 170.9, 170.5, 138.7, 137.9, 135.4, 135.3, 46.5, 46.4, 39.3, 38.9, 38.3, 37.6, 37.5, 36.4, 36.3, 33.9, 33.8, 28.2, 28.2, 28.0, 27.9, 26.1, 26.0, 22.6, 22.1, 21.0, 20.9, 13.6, 13.5, 11.5.
HRMS: exact mass calculated for $[M+H]^+$ ($C_{18}H_{26}NO_4$) requires *m/z* 320.1862, found *m/z* 320.1865.
The spectral data were consistent with those previously reported in the literature.[15]

Compound S50.

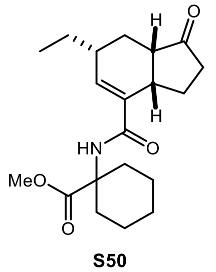

S50

Prepared according to General Procedure F using (±)-CFA (**2**) (20 mg, 0.10 mmol, 1 equiv.), HATU (44 mg, 0.12 mmol, 1.2 equiv.), methyl 1-aminocyclohexane-1-carboxylate hydrochloride (28 mg, 0.14 mmol, 1.5 equiv.), DIPEA (50 µL, 0.30 mmol, 3 equiv.), and DMF (0.5 mL). After 16 h the reaction was subjected to purification outlined in General Procedure F (silica gel, 20% EtOAc/CH_2Cl_2) to afford the title compound as a colourless oil (26 mg, 78%).
TLC (20% EtOAc/CH_2Cl_2): R_f = 0.34 stained by $KMnO_4$.

υ_{max} (film): 3357 (br.), 2932, 2855, 1738, 1660, 1625, 1517, 1277, 1238 cm^{-1}.
^{1}H NMR (500 MHz, $CDCl_3$): δ 6.31 (s, 1H), 5.86 (s, 1H), 3.71 (s, 3H), 3.15 (br. s, 1H), 2.46 – 2.22 (m, 4H), 2.19 – 2.10 (m, 2H), 2.06 – 1.97 (m, 1H), 1.95 – 1.82 (m, 3H), 1.75 – 1.48 (m, 5H), 1.47 – 1.29 (m, 4H), 1.12 – 1.01 (m, 1H), 0.98 (t, J = 7.1 Hz, 3H).
^{13}C NMR (101 MHz, $CDCl_3$): δ 220.3, 174.7, 168.0, 136.4, 136.3, 58.9, 52.4, 46.5, 38.3, 37.3, 36.4, 33.1, 32.0, 28.3, 27.8, 26.2, 25.3, 21.8, 21.8, 11.5.
HRMS: exact mass calculated for $[M+H]^+$ ($C_{20}H_{30}NO_4$) requires *m/z* 348.2169, found *m/z* 348.2167.

Compound 34.

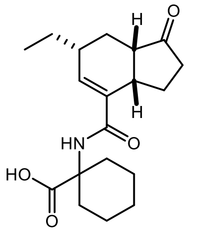

34

Prepared according to General Procedure G using compound **S50** (24 mg, 0.07 mmol, 1 equiv.), NaOH (8 mg, 0.20 mmol, 3 equiv.), and 1:1 MeOH:H_2O (4 mL). After 16 h the reaction was subjected to purification outlined in General Procedure G to afford the title compound as a white solid (22 mg, 96%).
υ_{max} (film): 3323 (br.), 2928, 2859, 1733, 1617, 1526, 1146 cm^{-1}.
^{1}H NMR (500 MHz, $CDCl_3$): δ 6.38 (s, 1H), 5.96 (s, 1H), 3.19 – 3.12 (m, 1H), 2.48 – 2.24 (m, 4H), 2.24 – 2.12 (m, 2H), 2.10 – 2.02 (m, 1H), 1.97 – 1.84 (m, 3H), 1.78 – 1.48 (m, 5H), 1.47 – 1.29 (m, 4H), 1.11 – 1.01 (m, 1H), 0.98 (t, J = 7.4 Hz, 3H). CO_2H not observed.
^{13}C NMR (126 MHz, $CDCl_3$): δ 177.1, 169.4, 137.8, 135.9, 59.6, 46.5, 38.3, 37.5, 36.3, 32.7, 31.7, 28.2, 27.9, 26.1, 25.2, 21.7, 21.6, 11.5. Carbonyl *C*O not observed.
HRMS: exact mass calculated for $[M-H]^-$ ($C_{19}H_{26}NO_4$) requires *m/z* 332.1867, found *m/z* 332.1860.

Compound S51.

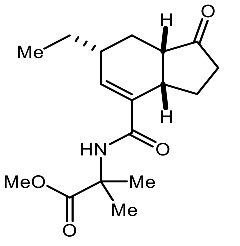

S51

Prepared according to General Procedure F using (±)-CFA (**2**) (20 mg, 0.10 mmol, 1 equiv.), HATU (44 mg, 0.12 mmol, 1.2 equiv.), methyl 2-amino-2-methylpropanoate hydrochloride (17 mg, 0.15 mmol, 1.5 equiv.), DIPEA (50 µL, 0.29 mmol, 3 equiv.), and DMF (0.2 mL). After 16 h the reaction mixture was subjected to the purification outlined in General Procedure F (silica gel, 30% EtOAc/CH_2Cl_2) to afford the title compound as a colourless oil (24 mg, 81%).
TLC (30% EtOAc/CH_2Cl_2): R_f = 0.32 stained by $KMnO_4$.
υ_{max} (film): 3304, 2938, 1734, 1649, 1607, 1522, 1267, 1148 cm^{-1}.

^{1}**H NMR** (500 MHz, $CDCl_3$): δ 6.35 (br. s, 1H), 6.31 (s, 1H), 3.75 (s, 3H), 3.18 – 3.11 (m, 1H), 2.47 – 2.22 (m, 4H), 2.17 – 2.09 (m, 1H), 1.87 (dt, *J* = 12.8, 4.8 Hz, 1H), 1.64 – 1.55 (m, 7H), 1.53 – 1.45 (m, 1H), 1.43 – 1.33 (m, 1H), 1.09 – 1.00 (m, 1H), 0.97 (t, *J* = 7.4 Hz, 3H).
^{13}**C NMR** (126 MHz, $CDCl_3$): δ 175.4, 167.7, 136.4, 136.2, 56.7, 52.8, 46.6, 38.3, 37.4, 36.4, 28.3, 27.9, 26.2, 25.0, 24.8, 11.5. Carbonyl *C*O not observed.
HRMS: exact mass calculated for $[M+H]^+$ ($C_{17}H_{26}NO_4$) requires *m/z* 308.1856, found *m/z* 308.1858.

Compound 35.

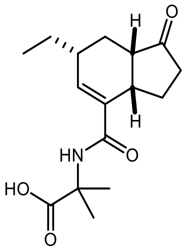

35

Prepared according to General Procedure G using compound **S51** (24 mg, 0.08 mmol, 1 equiv.), LiOH (6 mg, 0.25 mmol, 3 equiv.), and 1:1 MeOH:H_2O (2 mL). After 16 h the reaction was purified by flash silica column chromatography, eluent 1% AcOH, 30% EtOAc/CH_2Cl_2 to afford the title compound as a colourless oil (9 mg, 39%).
TLC (1% AcOH, 30% EtOAc/CH_2Cl_2): R_f = 0.40 stained by $KMnO_4$ and visible under UV (short wave).
υ_{max} (film): 3271, 2922, 2862, 1734, 1719, 1701, 1616, 1528 cm^{-1}.
^{1}**H NMR** (500 MHz, $CDCl_3$): δ 6.34 (s, 1H), 6.31 (s, 1H), 3.18 – 3.11 (m, 1H), 2.48 – 2.24 (m, 4H), 2.19 – 2.11 (m, 1H), 1.88 (dt, *J* = 12.7, 4.7 Hz, 1H), 1.66 – 1.54 (m, 7H), 1.54 – 1.47 (m, 1H), 1.42 – 1.32 (m, 1H), 1.10 – 1.01 (m, 1H), 0.98 (t, *J* = 7.4 Hz, 3H). CO_2*H* not observed.
^{13}**C NMR** (101 MHz, $CDCl_3$): δ 220.4, 178.3, 168.6, 137.4, 135.8, 57.0, 46.5, 38.3, 37.5, 36.3, 28.2, 27.9, 26.1, 25.1, 24.9, 11.5.
HRMS: exact mass calculated for $[M+H]^+$ ($C_{16}H_{24}NO_4$) requires *m/z* 294.1705, found *m/z* 294.1704.

Compound S52.

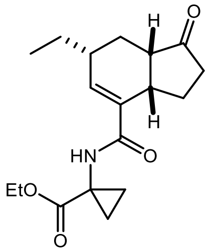

S52

Prepared according to General Procedure F using (±)-CFA (**2**) (30 mg, 0.14 mmol, 1 equiv.), HATU (66 mg, 0.18 mmol, 1.2 equiv.), ethyl 1-aminocyclopropane-1-carboxylate hydrochloride (36 mg, 0.22 mmol, 1.5 equiv.), DIPEA (80 µL, 0.46 mmol, 3 equiv.), and DMF (0.8 mL). After 16 h the reaction was subjected to purification outlined in General Procedure F (silica gel, 30% EtOAc/CH_2Cl_2) to afford the title compound as a pale orange oil (30 mg, 65%).
TLC (30% EtOAc/CH_2Cl_2): R_f = 0.24 stained by $KMnO_4$ and faintly visible by UV (short wave).
υ_{max} (film): 3320 (br.), 2958, 2930, 2872, 2854, 1729, 1658, 1625, 1513, 1333, 1180, 1156 cm^{-1}.

^{1}H NMR (500 MHz, $CDCl_3$): δ 6.31 (s, 1H), 6.29 (s, 1H), 4.20 – 4.08 (m, 2H), 3.23 – 3.15 (m, 1H), 2.52 – 2.43 (m, 1H), 2.41 – 2.22 (m, 3H), 2.18 – 2.09 (m, 1H), 1.87 (dt, J = 11.7, 4.5 Hz, 1H), 1.65 – 1.53 (m, 3H), 1.52 – 1.43 (m, 1H), 1.42 – 1.31 (m, 1H), 1.29 – 1.16 (m, 5H), 1.11 – 1.01 (m, 1H), 0.97 (t, J = 7.4 Hz, 3H).
^{13}C NMR (101 MHz, $CDCl_3$): δ 220.4, 172.4, 169.5, 136.5, 136.2, 61.6, 46.5, 38.3, 37.3, 36.4, 34.0, 28.3, 27.9, 26.2, 17.5, 14.3, 11.4. One signal equivalent.
HRMS: exact mass calculated for $[M+H]^+$ ($C_{18}H_{26}NO_4$) requires *m/z* 320.1856, found *m/z* 320.1855.

Compound 36.

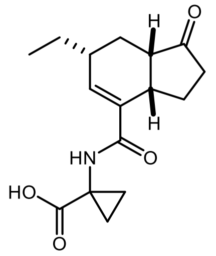

36

Prepared according to General Procedure G using compound **S52** (30 mg, 0.09 mmol, 1 equiv.), LiOH (8 mg, 0.33 mmol, 3 equiv.), and 1:1 MeOH:H_2O (4 mL). After 16 h the reaction was subjected to purification outlined in General Procedure G to afford the title compound as a white solid (4 mg, 15%).
υ_{max} (film): 3327 (br.), 2965, 2934, 2874, 1736, 1655, 1624, 1508, 1273, 1196, 1146 cm^{-1}.
^{1}H NMR (500 MHz, $CDCl_3$): δ 6.72 (s, 1H), 6.36 (s, 1H), 5.43 (br. s, 1H), 3.20 – 3.09 (m, 1H), 2.51 – 2.21 (m, 4H), 2.18 – 2.07 (m, 1H), 1.91 – 1.82 (m, 1H), 1.57 (br. s, 3H), 1.52 – 1.44 (m, 1H), 1.39 – 1.33 (m, 1H), 1.16 (br. s, 2H), 1.10 – 1.00 (m, 1H), 0.96 (t, J = 7.4 Hz, 3H).
^{13}C NMR (101 MHz, $CDCl_3$): δ 220.5, 170.0, 137.5, 135.6, 46.6, 38.3, 37.4, 36.3, 28.3, 28.0, 26.1, 17.9, 17.8, 11.5. One signal not observed, one signal equivalent.
HRMS: exact mass calculated for $[M-H]^-$ ($C_{16}H_{20}NO_4$) requires *m/z* 290.1398, found *m/z* 290.1393.

Compound S53.

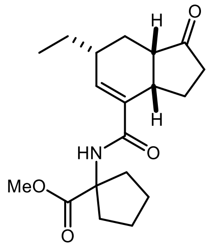

S53

Prepared according to General Procedure F using (±)-CFA (**2**) (20 mg, 0.1 mmol, 1 equiv.), HATU (44 mg, 0.15 mmol, 1.2 equiv.), methyl 1-aminocyclopropane-1-carboxylate (26 mg, 0.15 mmol, 1.5 equiv.), DIPEA (50 µL, 0.29 mmol, 3 equiv.), and DMF (0.2 mL). After 16 h the reaction mixture was subjected to the purification outlined in General Procedure F (silica gel, 30% EtOAc/CH_2Cl_2) to afford the title compound as a white solid (26 mg, 81%).
TLC (30% EtOAc/CH_2Cl_2): R_f = 0.62 stained by $KMnO_4$.
υ_{max} (film): 3329 (br.), 2957, 2874, 1736, 1655, 1618, 1516, 1449, 1267, 1194 cm^{-1}.

^{1}H NMR (500 MHz, $CDCl_3$): δ 6.29 (s, 1H), 6.18 (s, 1H), 3.73 (s, 3H), 3.19 – 3.12 (m, 1H), 2.47 – 2.20 (m, 6H), 2.17 – 2.09 (m, 1H), 2.06 – 1.93 (m, 2H), 1.87 (dt, *J* = 12.9, 4.8 Hz, 1H), 1.84 – 1.77 (m, 4H), 1.64 – 1.45 (m, 2H), 1.42 – 1.33 (m, 1H), 1.10 – 1.00 (m, 1H), 0.97 (t, *J* = 7.4 Hz, 3H).
^{13}C NMR (101 MHz, $CDCl_3$): δ 220.4, 175.1, 168.2, 136.4, 136.2, 66.1, 52.7, 46.5, 38.3, 37.8, 37.4, 37.3, 36.4, 28.3, 27.9, 26.2, 25.1, 11.5. One signal equivalent.
HRMS: exact mass calculated for $[M+H]^+$ ($C_{19}H_{28}NO_4$) requires *m/z* 334.2013, found *m/z* 334.2015.

Compound 37.

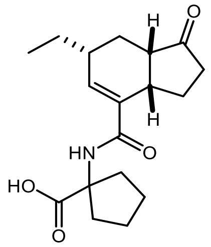

37

Prepared according to General Procedure G using compound **S53** (26 mg, 0.08 mmol, 1 equiv.), LiOH (6 mg, 0.25 mmol, 3 equiv.), and 1:1 MeOH:H_2O (3 mL). After 16 h the reaction was allowed to cool to room temperature, acidified with AcOH, and the organics extracted with EtOAc (3 x 10 mL). The organics were combined, dried over Na_2SO_4, filtered, and evaporated to afford a pale yellow oil. The crude material was loaded in a solution of CH_2Cl_2 and purified by flash silica column chromatography, eluent 1% AcOH, 30% EtOAc/CH_2Cl_2 to afford the title compound as a colourless oil (18 mg, 72%).
TLC (1% AcOH, 30% EtOAc/CH_2Cl_2): R_f = 0.59 stained by $KMnO_4$.
υ_{max} (film): 3281, 2959, 2934, 2862, 1734, 1719, 1695, 1612, 1528 cm^{-1}.
^{1}H NMR (500 MHz, $CDCl_3$): δ 7.91 (br. s, 1H), 6.34 (s, 1H), 6.29 (s, 1H), 3.18 – 3.11 (m, 1H), 2.47 – 2.23 (m, 6H), 2.18 – 2.10 (m, 1H), 2.07 – 1.92 (m, 2H), 1.88 (dt, *J* = 12.9, 4.8 Hz, 1H), 1.85 – 1.73 (m, 4H), 1.63 – 1.45 (m, 2H), 1.41 – 1.34 (m, 1H), 1.10 – 1.00 (m, 1H), 0.97 (t, *J* = 7.4 Hz, 3H).
^{13}C NMR (101 MHz, $CDCl_3$): δ 220.4, 178.0, 169.4, 137.6, 135.7, 66.7, 46.5, 38.3, 37.6, 37.5, 37.1, 36.3, 28.2, 27.9, 26.1, 24.8, 24.8, 11.5.
HRMS: exact mass calculated for $[M+H]^+$ ($C_{18}H_{26}NO_4$) requires *m/z* 320.1862, found *m/z* 320.1864.

Synthesis of (±)-CMA (3).

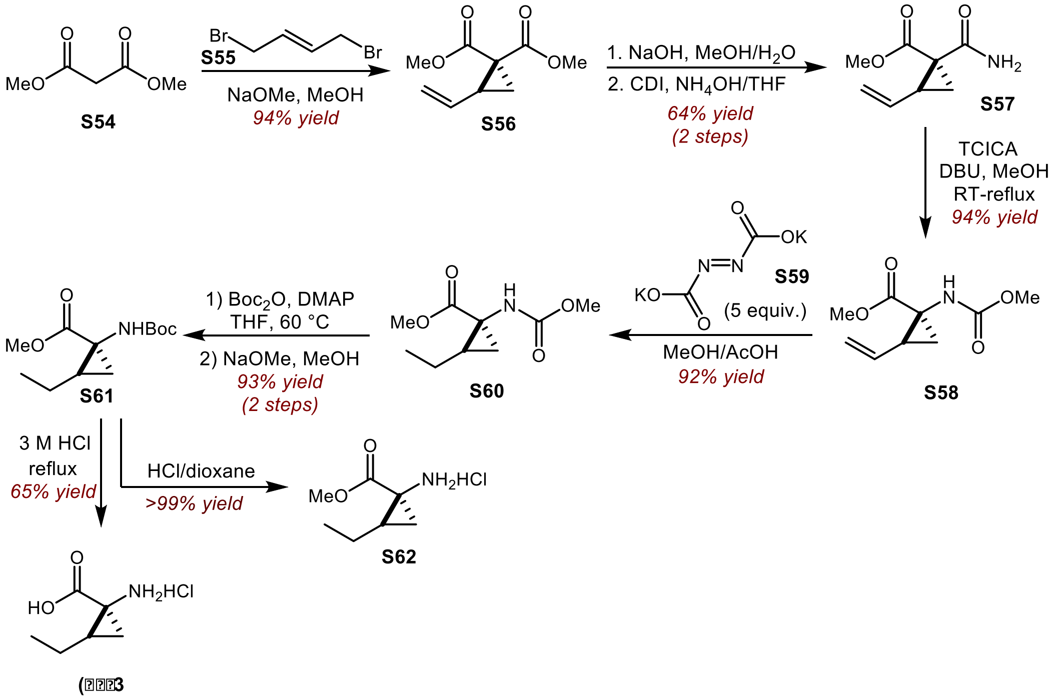

Compound S56.

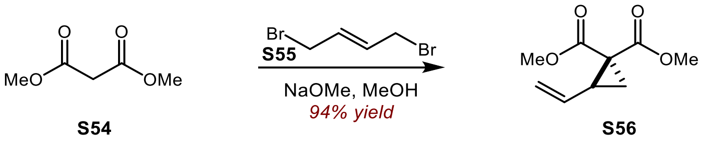

To a round bottom flask was added anhydrous MeOH (15 mL) followed by portionwise addition of Na metal (1.18 g, 51.30 mmol, 2.2 equiv.) at room temperature. The resulting solution was then added dropwise under nitrogen to a stirring solution of (*E*)-1,4-dibromobut-2-ene (**S55**) (5.00 g, 23.38 mmol, 1 equiv.) and dimethyl malonate (**S54**) (2.94 mL, 25.73 mmol, 1.1 equiv.) in anhydrous MeOH (10 mL) at room temperature. The resulting beige suspension was stirred at room temperature for 16 h. The reaction was diluted with water (20 mL) and extracted with EtOAc (3 x 20 mL). The organics were combined, washed with brine (20 mL), dried over Na_2SO_4, filtered, and evaporated to afford a colourless oil. The crude material was loaded in a solution of 10% EtOAc/petroleum ether and purified by flash silica column chromatography, eluent 10% EtOAc/petroleum ether to afford the title compound as a colourless oil (4.07 g, 94%).

TLC (10% EtOAc/petroleum ether): R_f = 0.18 stained by $KMnO_4$.

υ_{max} (neat): 2951, 1722, 1437, 1329, 1272, 1209, 1127 cm^{-1}.

^{1}H NMR (500 MHz, $CDCl_3$): δ 5.47 – 5.38 (m, 1H), 5.32 – 5.27 (m, 1H), 5.16 – 5.12 (m, 1H), 3.74 (s, 6H), 2.61 – 2.55 (m, 1H), 1.72 (dd, *J* = 7.6, 4.9 Hz, 1H), 1.61 – 1.55 (m, 1H).

^{13}C NMR (101 MHz, $CDCl_3$): δ 170.2, 167.9, 133.1, 118.8, 52.9, 52.7, 35.9, 31.6, 20.8.

HRMS: exact mass calculated for $[M+H]^+$ ($C_9H_{13}O_4$) requires *m/z* 185.0814, found *m/z* 185.0450.

The spectral data were consistent with those previously reported in the literature.[16]

Compound S57.

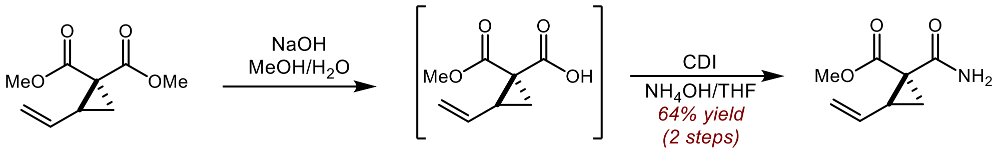

S56 **S63** **S57**

To a round bottom flask was added compound **S56** (11.36 g, 61.68 mmol, 1 equiv.) and MeOH/H_2O (1:1, 90 mL). NaOH (2.71 g, 67.75 mmol, 1.1 equiv.) was added in one portion and the resulting solution stirred at room temperature for 16 h. The reaction brought to pH 1 with HCl and extracted with EtOAc (3 x 50 ml). The organics were combined, washed with brine (50 mL), dried over Na_2SO_4, filtered, and evaporated to afford a colourless oil (**S63**) (10.04 g, 59.00 mmol).

To the colourless oil in a round bottom flask was added THF (240 mL), followed by CDI (10.53 g, 64.92 mmol, 1.1 equiv.) at room temperature. The reaction was stirred for 3 h, and NH_4OH (aq.) (200 mL) added slowly. The reaction was stirred for a further 16 h. The reaction was diluted with water (100 mL) and extracted with EtOAc (3 x 100 mL). The organics were combined, washed with brine (100 mL), dried over Na_2SO_4, filtered, and evaporated to afford a colourless oil which solidified to a white solid on standing. The crude material was dry loaded onto silica gel and purified by flash silica column chromatography, eluent 30-50% EtOAc/petroleum ether to afford the title compound as a white solid (6.67 g, 64%).

TLC (40% EtOAc/petroleum ether): R_f = 0.26 stained by $KMnO_4$.

υ_{max} (neat): 3411, 3169 (br.), 1709, 1679, 1400, 1329, 1134, 1108 cm^{-1}.

^{1}**H NMR** (500 MHz, $CDCl_3$): δ 8.20 (br. s, 1H), 5.68 – 5.57 (m, 2H), 5.35 (dd, *J* = 17.0, 0.6 Hz, 1H), 5.18 (dd, *J* = 10.2, 1.2 Hz, 1H), 3.73 (s, 3H), 2.58 (q, *J* = 8.7 Hz, 1H), 2.07 (dd, *J* = 9.2, 4.4 Hz, 1H), 1.90 (dd, *J* = 8.0, 4.4 Hz, 1H).

^{13}**C NMR** (101 MHz, $CDCl_3$): δ 171.6, 170.4, 133.2, 120.0, 52.3, 37.6, 34.6, 21.9.

HRMS: exact mass calculated for $[M+H]^+$ ($C_8H_{12}NO_3$) requires *m/z* 170.0812, found *m/z* 170.0812.

The spectral data were consistent with those previously reported in the literature.[16]

Compound S58.

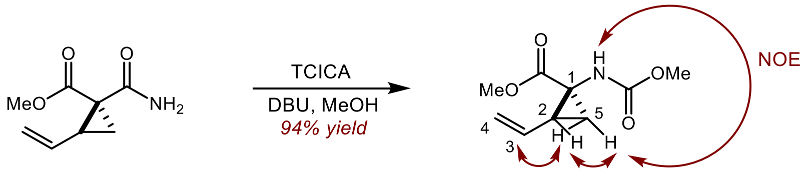

S57 **S58**

To a round bottom flask was added compound **S57** (1.49 g, 8.81 mmol, 1 equiv.) and MeOH (15 mL) under an atmosphere of nitrogen. DBU (2.96 mL, 19.79 mmol, 2.25 equiv.) was added, followed by portionwise addition of TCICA (778 mg, 3.35 mmol, 0.38 equiv.) and the resulting solution brought to 65 °C for 16 h. The solvent was removed *in vacuo* to afford an orange oil which solidified to an orange solid on standing. The material was dry loaded and purified by flash silica column chromatography, eluent 30% EtOAc/petroleum ether to afford the title compound colourless oil (1.65 g, 94%).

TLC (30% EtOAc/petroleum ether): R_f = 0.37 stained by $KMnO_4$.

υ_{max} (neat): 3321 (br.), 2951, 1705, 1514, 1324, 1248, 1164 cm^{-1}.

^{1}**H NMR** (400 MHz, $CDCl_3$): δ 5.73 (ddd, *J* = 17.2, 10.2, 8.9 Hz, 1H, H^3), 5.41 (br. s, 1H, N*H*), 5.28 (d, *J* = 17.0 Hz, 1H, H^4), 5.11 (dd, *J* = 10.3, 1.4 Hz, 1H, $H^{4'}$), 3.70 (s, 3H, C*H*$_3$), 3.68 (s, 3H, C*H*$_3$), 2.16 (q, *J* = 8.8 Hz, 1H, H^2), 1.85 – 1.78 (m, 1H, H^5), 1.53 (br. s, 1H, $H^{5'}$).

^{13}**C NMR** (101 MHz, $CDCl_3$): δ 171.2 (*C*O), 133.6 (C^3), 118.1 (C^4), 52.6 (*C*H_3 x 2), 41.0 (C^1), 34.7 (C^2), 23.5 (C^5). One carbonyl *C*O not observed, one peak equivalent.

HRMS: exact mass calculated for $[M+H]^+$ ($C_9H_{14}NO_4$) requires *m/z* 200.0917, found *m/z* 200.0915.

The spectral data were consistent with those previously reported in the literature.[16]

Compound S60.

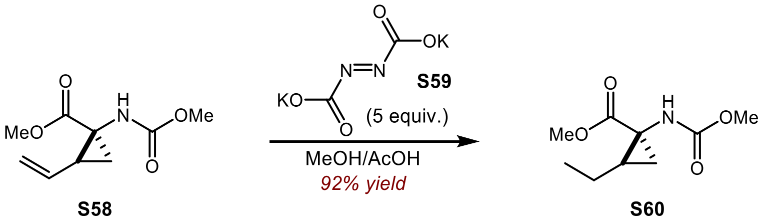

To a round bottom flask was added compound **S58** (6.28 g, 34.24 mmol, 1 equiv.), dipotassium azo-1,2-dicarboxylate[17] (**S59**) (33.00 g, 169.90 mmol, 5 equiv.), and MeOH (55 mL). AcOH was added dropwise at 0 °C and the resulting suspension allowed to rise to room temperature and stir for 16 h. The reaction concentrated *in vacuo*, diluted with water (30 mL) and extracted with EtOAc (3 x 40 mL). The organics were combined, washed with brine (40 mL), dried over Na_2SO_4, filtered, and evaporated to afford a colourless oil. The crude material was loaded in a solution of 30% EtOAc/petroleum ether and purified by flash silica column chromatography, eluent 30% EtOAc/petroleum ether to afford the title compound as a colourless oil (6.36 g, 92%).

TLC (30% EtOAc/petroleum ether): R_f = 0.37 stained by $KMnO_4$.

υ_{max} (neat): 2921 (br.), 2572 (br.), 1666, 1588, 1311, 1283, 1216 cm^{-1}.

^{1}H NMR (400 MHz, $CDCl_3$): δ 5.33 (br. s, 1H), 3.70 (s, 3H), 3.67 (s, 3H), 1.65 – 1.42 (m, 4H), 1.33 – 1.22 (m, 1H), 0.94 (t, *J* = 7.2 Hz, 3H).

^{13}C NMR (101 MHz, $CDCl_3$): δ 172.2, 52.5, 38.9, 33.9, 23.4, 20.5, 13.5. One carbonyl *C*O not observed, one peak coincident.

HRMS: exact mass calculated for $[M+H]^+$ ($C_9H_{16}NO_4$) requires *m/z* 202.1074, found *m/z* 202.1070.

Compound S61.

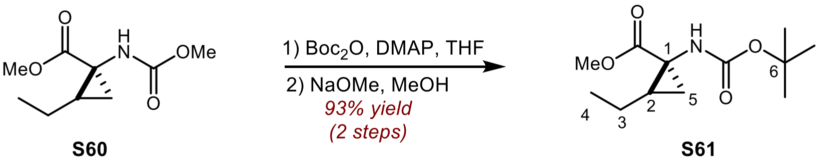

To a round bottom flask charged with compound **S60** (6.36 g, 31.61 mmol, 1 equiv.) and Boc_2O (8.97 g, 41.10 mmol, 1.3 equiv.) in a solution of THF (50 mL) was added DMAP (777 mg, 6.33 mmol, 0.2 equiv.) at room temperature under an atmosphere of nitrogen. The reaction was brought to 70 °C for 3 h. The reaction was then allowed to cool to room temperature and diluted with anhydrous MeOH (30 mL). To a separate round bottom flask charged with anhydrous MeOH (35 mL) was added Na metal (223 mg, 9.70 mmol, 0.3 equiv.) portionwise under an atmosphere of nitrogen. The resulting solution was then added dropwise to the reaction flask at 0 °C in an ice bath. The reaction was allowed to rise to room temperature and stirred for 1.5 h. The reaction was diluted with water (100 mL) and extracted with EtOAc (3 x 100 mL). The organics were combined, washed with brine (150 mL), dried over Na_2SO_4, filtered, and evaporated to afford a pale orange oil. The crude material was loaded in a solution of 20% EtOAc/petroleum ether and purified by flash silica column chromatography, eluent 20% EtOAc/petroleum ether to afford the title compound as a pale yellow oil (7.18 g, 93%).

TLC (30% EtOAc/petroleum ether): R_f = 0.60 stained by $KMnO_4$.

υ_{max} (neat): 3261 (br.), 3131, 2961, 2926, 2868, 1705, 1377, 1364, 1335, 1165, 1022 cm^{-1}.

^{1}H NMR (500 MHz, $CDCl_3$): δ 5.13 (br. s, 1H, N*H*), 3.74 (s, 3H, OC*H*$_3$), 1.63 – 1.54 (m, 2H, H^3), 1.49 – 1.43 (m, 11H, H^2, H^5, 3C*H*$_3$), 1.29 (br. s, 1H, $H^{5'}$), 0.97 (t, J = 7.4 Hz, 3H, H^4).

^{13}C NMR (126 MHz, $CDCl_3$): δ 172.5 (*C*O), 156.1 (*C*O), 80.0 (br., C^6), 52.3 (*C*H$_3$), 39.0 (br., C^1), 33.5 (br., C^2), 28.5 (*C*H$_3$), 28.5 (*C*H$_3$), 28.4 (*C*H$_3$), 23.3 (br., C^5), 20.5 (C^3), 13.6 (C^4).

HRMS: exact mass calculated for $[M+H]^+$ ($C_{12}H_{22}NO_4$) requires *m/z* 244.1543, found *m/z* 244.1544.

Compound S62.

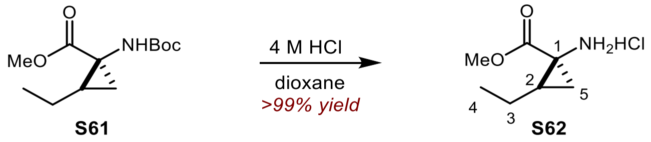

To a round bottomed flask charged with compound **S61** (150 mg, 0.62 mmol, 1 equiv.) and dioxane (1.2 mL) was added 6 M HCl (1.5 mL) dropwise at room temperature and the resulting solution stirred for 5 h. The solvent was removed *in vacuo* to afford a colourless oil, which was dissolved in acetone (2 mL) and the solvent removed *in vacuo* to afford the title compound as a colourless oil which solidified to a white solid on standing (113 mg, > 99%).

υ_{max} (neat): 2872 (br.), 1745, 1526, 1444, 1370, 1201, 1167 cm^{-1}.

^{1}H NMR (400 MHz, $CDCl_3$): δ 9.01 (br. s, 2H, N*H*$_2$), 3.84 (s, 3H, OC*H*$_3$), 2.05 – 1.94 (m, 1H, H^2), 1.90 – 1.82 (m, 1H, H^5), 1.72 – 1.54 (m, 2H, H^3), 1.51 – 1.44 (m, 1H, $H^{5'}$), 0.99 (t, J = 7.4 Hz, 3H, H^4).

^{13}C NMR (101 MHz, $CDCl_3$): δ 168.6 (*C*O), 53.3 (O*C*H$_3$), 38.5 (C^1), 30.5 (C^2), 20.1 ($C^{3/5}$), 20.0 ($C^{3/5}$), 13.5 (C^4).

HRMS: exact mass calculated for $[M+H]^+$ ($C_7H_{14}NO_2$) requires *m/z* 144.1019, found *m/z* 144.1016.

The spectral data were consistent with those previously reported in the literature.[18]

(±)-CMA (3).

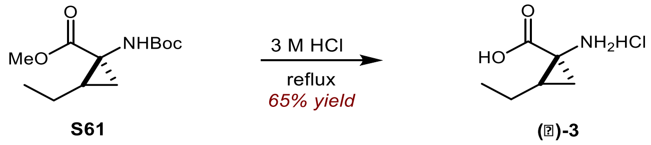

To a round bottomed flask was added compound **S61** (1.35 g, 4.11 mmol) and 3 M HCl (60 mL). The reaction was brought to 100 °C for 16 h. The reaction was allowed to cool to room temperature and concentrated *in vacuo* to afford a pale orange solid. The solid material was washed sparingly with acetone to afford the title compound as a beige solid (596 mg, 65%).

υ_{max} (neat): 2956 (br.), 1714, 1500, 1253, 1165 cm^{-1}.

^{1}H NMR (400 MHz, D_2O): δ 1.80 – 1.68 (m, 2H), 1.61 – 1.51 (m, 3H), 0.98 (t, J = 7.0 Hz, 3H). N*H*$_2$ and CO_2*H* not observed.

^{13}C NMR (101 MHz, DMSO-d_6): δ 170.1, 37.2, 28.2, 19.4, 18.2, 13.3.

HRMS: exact mass calculated for $[M-H]^-$ ($C_6H_{10}NO_2$) requires *m/z* 128.0717, found *m/z* 128.0721.

The spectral data were consistent with those previously reported in the literature.[19]

Compound S64.

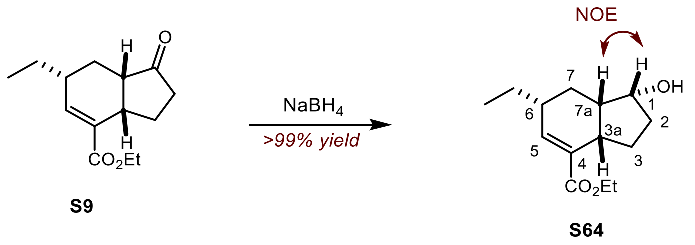

To a round bottom flask charged with compound **S9** (50 mg, 0.21 mmol, 1 equiv.) in a solution was EtOH (1 mL) was added $NaBH_4$ (9 mg, 0.24 mmol, 1.2 equiv.) in one portion at room temperature under an atmosphere of nitrogen. The reaction was stirred for 30 minutes, quenched with water (5 mL) and extracted with EtOAc (3 x 10 mL). The organics were combined, washed with brine (10 mL), dried over Na_2SO_4, filtered, and evaporated to afford the title compound as a colourless oil (50 mg, >99%).

TLC (20% EtOAc/petroleum ether): R_f = 0.21 stained by $KMnO_4$.

υ_{max} (neat): 3406 (br.), 2956, 2919, 2870, 2855, 1708, 1640, 1463, 1242, 1100 cm^{-1}.

^{1}H NMR (400 MHz, $CDCl_3$): δ 6.82 (s, 1H, H^5), 4.37 (td, J = 8.4, 6.5 Hz, 1H, H^1), 4.24 – 4.09 (m, 2H, $CO_2CH_2CH_3$), 2.76 – 2.67 (m, 1H, H^{3a}), 2.16 – 1.97 (m, 4H, H^2, H^3, H^{7a}, H^6), 1.87 – 1.79 (m, 1H, H^7), 1.68 – 1.32 (m, 4H, $H^{2'}$, $H^{3'}$, CH_2CH_3), 1.27 (t, J = 7.1 Hz, 3H, $CO_2CH_2CH_3$), 0.98 (t, J = 7.4 Hz, 3H, CH_2CH_3), 0.95 – 0.85 (m, 1H, $H^{7'}$). O*H* not observed.

^{13}C NMR (101 MHz, $CDCl_3$): δ 167.6 (CO_2Et), 143.5 (C^5), 133.5 (C^4), 75.2 (C^1), 60.3 ($CO_2CH_2CH_3$), 42.5 (CH), 38.0 (CH), 36.4 (C^{3a}), 31.1 (CH_2), 28.5 (CH_2), 28.4 (CH_2), 23.9 (C^7), 14.4 ($CO_2CH_2CH_3$), 11.5 (CH_2CH_3).

HRMS: exact mass calculated for $[M+NH_4]^+$ ($C_{14}H_{26}NO_3$) requires *m/z* 256.1909, found *m/z* 256.1884.

Compound S66.

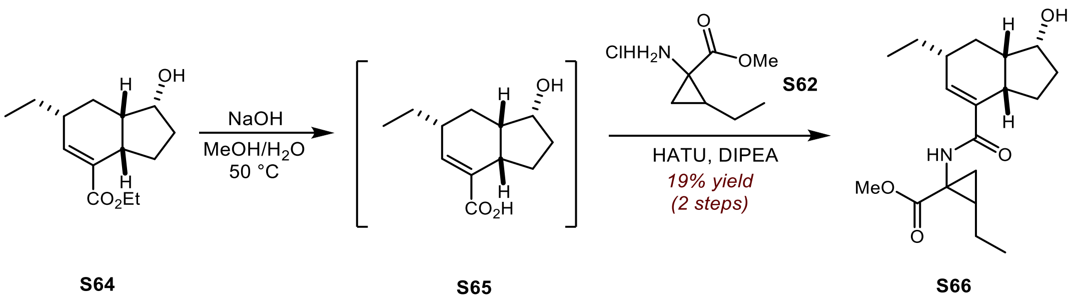

To a round bottom flask charged with compound **S64** (31 mg, 0.13 mmol, 1 equiv.) in a solution of 1:1 MeOH/H_2O (9 mL) was added NaOH (22 mg, 0.55 mmol, 4.4 equiv.) in one portion. The resulting solution was brought to 50 °C for 21 h. The reaction was allowed to cool to room temperature, extracted with EtOAc (5 mL) and the aqueous brought to pH 1 with HCl (aq.). The aqueous was extracted with EtOAc (3 x 10 mL), and the organics combined, washed with brine (10 mL), dried over Na_2SO_4, filtered and evaporated to afford compound **S65** as a colourless oil (11 mg, 0.05 mmol).

The oil was transferred to a 2-dram vial and HATU (26 mg, 0.07 mmol, 1.2 equiv.) added, followed by DMF (0.3 mL) and DIPEA (30 µL, 0.17 mmol, 3 equiv.). The reaction was stirred at room temperature for 5 minutes before compound **S62** (14 mg, 0.08 mmol, 1.5 equiv.) was added. The resulting solution was stirred at room temperature for 6 h. The reaction was diluted with water (10 mL) and extracted with EtOAc (3 x 5 mL). The organics were combined, washed with brine (10 mL), dried over Na_2SO_4, filtered, and evaporated to afford a pale orange oil. The crude material was loaded in a solution of CH_2Cl_2 and purified by flash silica column chromatography, eluent 40-50% EtOAc/CH_2Cl_2 to afford the title compound as a white solid (6 mg, 19% (2 steps)).

TLC (30% EtOAc/CH_2Cl_2): R_f = 0.16 stained by $KMnO_4$ and faintly visible by UV (short wave).

υ_{max} (film): 3359 (br.), 2956, 2926, 2894, 1734, 1508, 1459, 1253, 1193 cm^{-1}.

^{1}H NMR (500 MHz, $CDCl_3$): δ 6.37 – 6.32 (m, 1H), 6.19 (s, 1H), 4.42 – 4.36 (m, 1H), 3.69 (s, 3H), 2.80 – 2.72 (m, 1H), 2.21 – 2.12 (m, 1H), 2.09 – 1.97 (m, 3H), 1.89 – 1.83 (m, 1H), 1.68 – 1.36 (m, 8H), 1.29 – 1.23 (m, 1H), 1.02 – 0.95 (m, 6H), 0.94 – 0.84 (m, 1H). O*H* not observed.

^{13}C NMR (101 MHz, $CDCl_3$): δ 171.8, 169.5, 169.4, 137.3, 137.2, 137.0, 75.1, 52.5, 52.4, 42.4, 38.4, 38.3, 37.6, 37.6, 36.4, 33.3, 33.2, 31.4, 28.7, 28.1, 28.0, 24.3, 24.2, 23.3, 23.2, 20.6, 13.6, 11.6.

HRMS: exact mass calculated for $[M+H]^+$ ($C_{19}H_{30}NO_4$) requires *m/z* 336.2169, found *m/z* 336.2173.

Compound 38a.

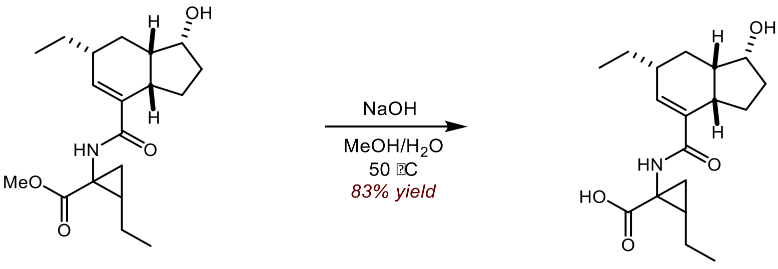

S66 **38a**

Prepared according to General Procedure G using compound **S66** (5 mg, 0.01 mmol, 1 equiv.), NaOH (2 mg, 0.05 mmol, 5 equiv.), and 1:1 MeOH/H_2O (1 mL). After 7 h the reaction was subjected to purification outlined in General Procedure G to afford the title compound as a white solid (4 mg, 83 %).

υ_{max} (film): 3317 (br.), 2956, 2921, 2870, 1697, 1654, 1619, 1509, 1275, 1182 cm^{-1}.

^{1}H NMR (400 MHz, $CDCl_3$): δ 6.49 (s, 1H), 6.38 – 6.28 (m, 1H), 4.44 – 4.35 (m, 1H), 2.77 – 2.63 (m, 1H), 2.23 – 2.13 (m, 1H), 2.12 – 1.92 (m, 3H), 1.92 – 1.83 (m, 1H), 1.73 – 1.32 (m, 8H), 1.22 – 1.12 (m, 1H), 1.06 – 0.97 (m, 6H), 0.94 – 0.86 (m, 1H). CO_2*H* and O*H* not observed.

^{13}C NMR (101 MHz, $CDCl_3$): δ 172.8, 172.3, 172.0, 171.6, 140.5, 139.9, 135.6, 74.9, 42.5, 42.4, 40.1, 39.8, 37.8, 37.8, 36.3, 36.2, 33.6, 33.5, 31.3, 28.5, 28.5, 28.3, 28.0, 24.0, 21.3, 21.3, 21.2, 20.8, 13.5, 13.4, 11.5.

HRMS: exact mass calculated for $[M+H]^+$ ($C_{18}H_{28}NO_4$) requires *m/z* 322.2013, found *m/z* 322.2015.

Compound 38b.

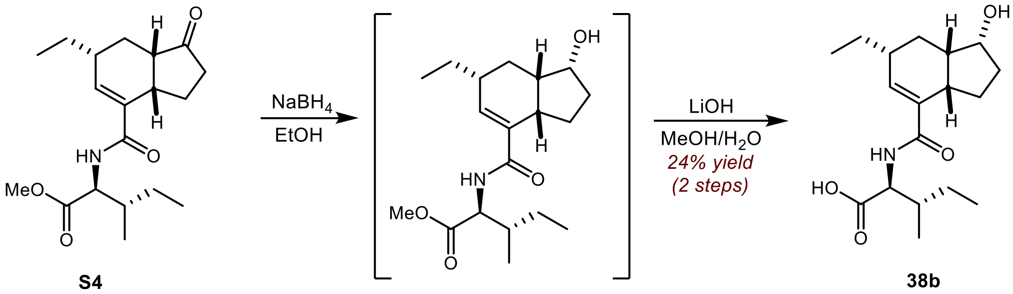

S4 **38b**

To a round bottom flask charged with compound **S4** (34 mg, 0.10 mmol, 1 equiv.) in a solution of EtOH (3 mL) was added $NaBH_4$ (6 mg, 0.16 mmol, 1.5 equiv.) in one portion under an atmosphere of nitrogen. The reaction was stirred at room temperature for 16 h, before being quenched with water (5 mL). The organics were extracted with EtOAc (3 x 10 mL) and the layers combined, washed with brine (10 mL), dried over Na_2SO_4, filtered, and evaporated to afford a colourless oil. The residue was suspended in 1:1 MeOH:H_2O (5 mL) and LiOH (7 mg, 0.29 mmol, 3 equiv.) added. The resulting suspension was brought to 50 °C and maintained at this temperature for 16 h. The reaction was allowed to cool to room temperature,

acidified with AcOH, and the organics extracted with EtOAc (3 x 10 mL). The organics were combined, dried over Na_2SO_4, filtered, and evaporated to afford a pale yellow oil. The crude material was taken up in diethyl ether and petroleum ether added until a white precipitate formed. The solvent was removed with a Pasteur pipette, and the residue dried under vacuum to afford the title compound as a white solid (8 mg, 24%).

ν_{max} (film): 3412 (br.), 3174, (br.) 1709, 1679, 1400, 1331, 1136, 1108 cm $^{-1}$.

^{1}H NMR (500 MHz, $CDCl_3$): δ 6.54 – 6.46 (m, 1H), 6.39 – 6.26 (m, 1H), 4.52 (br. s, 1H), 4.42 – 4.34 (m, 1H), 4.19 (br. s, 1H), 2.77 – 2.66 (m, 1H), 2.21 – 2.12 (m, 1H), 2.11 – 1.91 (m, 4H), 1.92 – 1.83 (m, 1H), 1.72 – 1.61 (m, 1H), 1.59 – 1.36 (m, 4H), 1.23 – 1.12 (m, 1H), 1.02 – 0.81 (m, 10H). One signal not observed.

^{13}C NMR (101 MHz, $CDCl_3$): δ 168.6, 168.5, 139.2, 138.5, 136.1, 135.8, 75.0, 75.0, 42.6, 42.5, 37.7, 37.6, 37.6, 36.3, 36.1, 31.1, 31.0, 28.6, 28.2, 28.2, 25.4, 25.3, 24.1, 15.8, 15.7, 11.7, 11.6, 11.5.

HRMS: exact mass calculated for $[M-H]^-$ ($C_{18}H_{28}NO_4$) requires *m/z* 322.2024, found *m/z* 322.2024.

Compound S67.

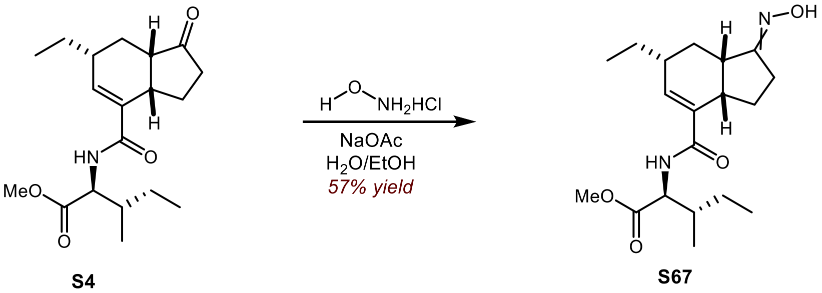

To a round bottom flask charged with hydroxylamine hydrochloride (10 mg, 0.14 mmol, 1.5 equiv.) and NaOAc (10 mg, 0.12 mmol, 1.2 equiv.) in a solution of H_2O (0.5 mL) was added compound **S4** (32 mg, 0.10 mmol, 1 equiv.) in EtOH (0.2 mL) at room temperature. The reaction was stirred for 16 h before being diluted with H_2O (5 mL) and extracted with EtOAc (3 x 5 mL). The organics were combined, washed with brine (5 mL), dried over Na_2SO_4, filtered, and evaporated to afford a colourless oil. The crude material was taken up in diethyl ether and petroleum ether added until a precipitate formed. The solvent was removed with a Pasteur pipette and the residue dried under vacuum to afford the title compound as a colourless oil (19 mg, 57%). 7:3 oxime isomers.

TLC (30% EtOAc/CH_2Cl_2): R_f = 0.29 stained by $KMnO_4$ and faintly visible by UV (short wave).

ν_{max} (film): 3316 (br.), 2957, 2922, 2876, 2855, 1744, 1649, 1612, 1518 cm^{-1}.

^{1}H NMR (500 MHz, $CDCl_3$): δ 7.83 (br. s, 1H), 6.46 – 6.38 (m, 1H), 6.31 – 6.25 (m, 1H), 4.72 – 4.65 (m, 1H), 3.75 (s, 3H), 3.22 – 3.14 (m, 0.3H (minor)), 3.00 – 2.68 (m, 2.7H), 2.59 – 2.14 (m, 4H), 1.99 – 1.89 (m, 1.3H), 1.86 – 1.80 (m, 0.7H (major)), 1.56 – 1.33 (m, 3H), 1.27 – 1.09 (m, 2H), 1.02 – 0.89 (m, 9H). Major/minor isomers reported where separation of signals observed.

^{13}C NMR (101 MHz, $CDCl_3$): δ 173.1, 173.0, 168.0, 167.9, 137.7, 137.7, 137.5, 135.6, 135.4, 56.5, 56.5, 52.3, 41.3, 41.2, 38.3, 38.3, 38.1, 37.7, 37.6, 37.5, 29.9, 29.9, 29.6, 29.5, 29.4, 29.3, 28.3, 26.4, 25.5, 25.4, 15.7, 15.6, 11.7, 11.7, 11.5, 11.4.

HRMS: exact mass calculated for $[M+H]^+$ ($C_{19}H_{31}N_2O_4$) requires *m/z* 351.2278, found *m/z* 351.2281.

Compound 39b.

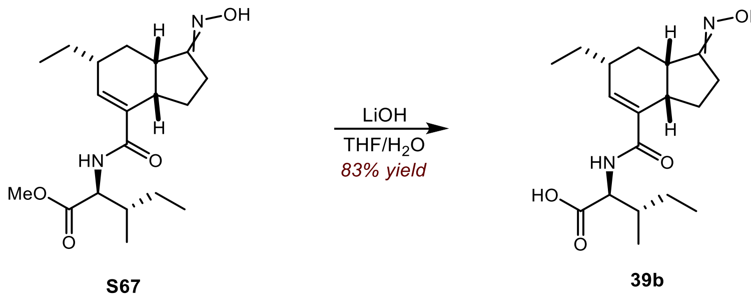

To a round bottom flask was added compound **S67** (10 mg, 0.03 mmol, 1 equiv.) and LiOH (3 mg, 0.13 mmol, 4 equiv.). The material was suspended in 1:1 THF:H_2O (1 mL) and the resulting suspension brought to 40 °C for 16 h. The reaction was allowed to cool to room temperature, extracted once with EtOAc (10 mL), the aqueous acidified with AcOH and the organics extracted with EtOAc (3 x 5 mL). The organics were combined, washed with brine (5 mL) dried over Na_2SO_4, filtered, and evaporated to afford a colourless oil. The crude material was dissolved in a minimal volume of diethyl ether and petroleum ether added until a white precipitate formed. The solvent was removed by Pasteur pipette and the residue dried under vacuum to afford the title compound as a white solid (8 mg, 83%). 7:3 oxime isomers.

υ_{max} (film): 3323 (br.), 2963, 2928, 2874, 1719, 1655, 1612, 1508, 1202 cm^{-1}.

^{1}H NMR (500 MHz, $CDCl_3$): δ 6.47 – 6.41 (m, 1H), 6.34 – 6.28 (m, 1H), 5.81 (br. s, 1H), 4.72 – 4.67 (m, 1H), 3.24 – 3.15 (m, 0.3H (minor)), 3.03 – 2.71 (m, 2.7 H), 2.62 – 2.27 (m, 3H), 2.21 (br. s, 1H), 2.06 – 1.95 (m, 1H), 1.84 – 1.75 (m, 0.7H (major)), 1.61 – 1.35 (m, 4.3H), 1.33 – 1.09 (m, 1H), 1.03 – 0.90 (m, 9H). One signal not observed. Major/minor isomers reported where separation of signals observed.

^{13}C NMR (126 MHz, $CDCl_3$): δ 176.0, 168.2, 168.1, 137.7, 137.5, 135.3, 56.8, 56.7, 56.7, 41.2, 41.2, 38.2, 38.2, 38.2, 38.0, 38.0, 37.6, 37.5, 29.8, 29.8, 29.6, 29.5, 29.3, 29.2, 28.2, 28.2, 26.8, 25.4, 25.4, 15.7, 15.7, 11.8, 11.8, 11.5, 11.4, 11.4.

HRMS: exact mass calculated for $[M-H]^-$ ($C_{18}H_{27}N_2O_4$) requires *m/z* 335.1976, found *m/z* 335.1973.

Compound S68.

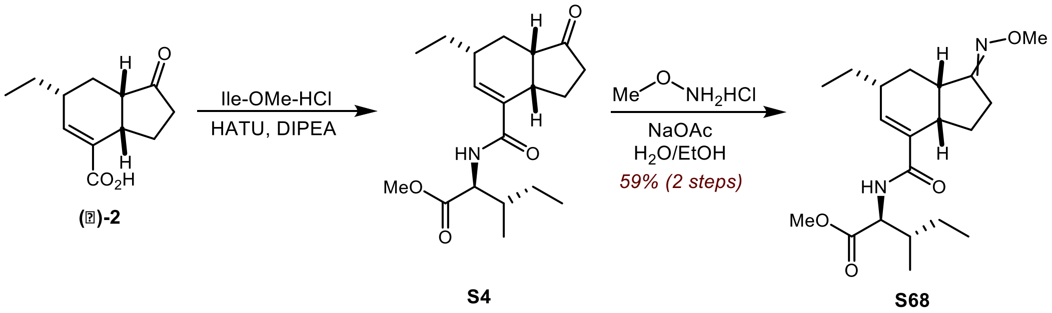

To a 2-dram vial was added (±)-CFA (**2**) (20 mg, 0.10 mmol, 1 equiv.) and HATU (44 mg, 0.12 mmol, 1.2 equiv.). DMF (0.5 mL) was added, followed by DIPEA (50 µL, 0.29 mmol, 3 equiv.) and the resulting solution stirred at room temperature for 5 minutes. Methyl *L*-isoleucinate hydrochloride (26 mg, 0.14 mmol, 1.5 equiv.) was then added in one portion and the vial capped with a screw top lid. The reaction was stirred for 16 h under air. The reaction was then diluted with H_2O (10 mL) and the organics extracted with EtOAc (3 x 5 mL). The organics were combined, washed with brine (10 mL), dried over Na_2SO_4, filtered, and evaporated to afford a pale yellow oil. The crude material was loaded in a solution of CH_2Cl_2 and purified by flash silica column chromatography, eluent 30% EtOAc/CH_2Cl_2 to afford compound **S4** as a colourless oil. The residue which was taken up in EtOH (0.18 mL) and added to a stirring solution of *O*-methylhydroxylamine hydrochloride (13 mg, 0.16 mmol, 1.5 equiv.) and NaOAc (11 mg, 0.13 mmol, 1.25 equiv.) in H_2O (0.55 mL). The reaction was stirred for 16 h

before being diluted with H_2O (5 mL) and extracted with EtOAc (3 x 5 mL). The organics were combined, washed with brine (5 mL), dried over Na_2SO_4, filtered, and evaporated to afford a colourless oil. The crude material was loaded in a solution of CH_2Cl_2 and purified by flash silica column chromatography, eluent 10-20% EtOAc/CH_2Cl_2 to afford the title compound as a colourless oil (23 mg, 59% (2 steps)). 7:3 oxime isomers.

TLC (10% EtOAc/CH_2Cl_2): R_f = 0.14 and 0.08 stained by $KMnO_4$ and faintly visible by UV (short wave). Separation of isomers visible.

υ_{max} (film): 3315 (br.), 2958, 2934, 2874, 2857, 1742, 1656, 1619, 1519, 1050 cm^{-1}.

^{1}H NMR (400 MHz, $CDCl_3$): δ 6.43 – 6.35 (m, 1H), 6.27 – 6.20 (m, 1H), 4.70 – 4.63 (m, 1H), 3.87 – 3.80 (m, 3H), 3.74 (s, 3H), 3.12 – 3.02 (m, 0.3H (minor)), 2.98 – 2.79 (m, 1H), 2.76 – 2.60 (m, 1.3H (minor)), 2.59 – 2.10 (m, 3.7H (major)), 1.97 – 1.87 (m, 1H), 1.87 – 1.80 (m, 0.7H (major)), 1.56 – 1.30 (m, 4H), 1.22 – 1.07 (m, 2H), 1.01 – 0.88 (m, 9H). Major/minor isomers reported where separation of signals observed.

^{13}C NMR (101 MHz, $CDCl_3$): δ 173.0, 172.9, 168.0, 168.0, 168.0, 167.9, 167.5, 167.5, 167.0, 137.6, 137.5, 135.6, 135.6, 135.5, 135.4, 61.6, 56.5, 56.4, 52.3, 41.4, 41.3, 39.4, 39.4, 38.3, 38.2, 38.1, 37.7, 37.6, 37.5, 37.4, 30.1, 30.1, 29.8, 29.7, 29.5, 29.4, 28.3, 28.2, 26.8, 25.8, 25.7, 25.5, 25.4, 15.7, 15.6, 11.7, 11.7, 11.5, 11.4, 11.4.

HRMS: exact mass calculated for $[M+H]^+$ ($C_{20}H_{33}N_2O_4$) requires *m/z* 365.2435, found *m/z* 365.2431.

Compound 40b.

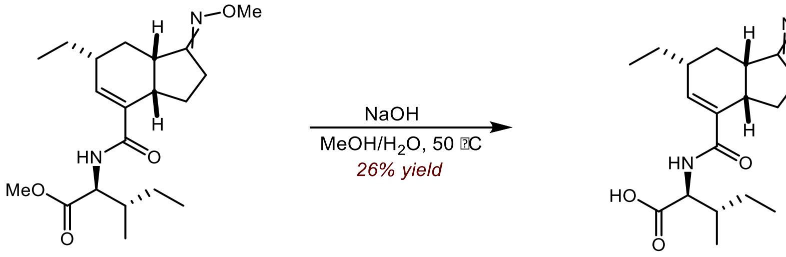

S68 40b

Prepared according to General Procedure G using compound **S68** (20 mg, 0.05 mmol, 1 equiv.) NaOH (5 mg, 0.13 mmol, 2 equiv.), and 1:1 MeOH:H_2O (4 mL). After 5 h the reaction was subjected to purification outlined in General Procedure G to afford the title compound as a white solid (5 mg, 26%). 7:3 oxime isomers.

υ_{max} (film): 3323 (br.), 2963, 2937, 2878, 1727, 1659, 1616, 1521, 1052 cm^{-1}.

^{1}H NMR (500 MHz, $CDCl_3$): δ 6.49 – 6.38 (m, 1H), 6.31 – 6.25 (m, 1H), 4.69 – 4.63 (m, 1H), 3.93 – 3.76 (m, 3H), 3.15 – 3.03 (m, 0.3H (minor)), 2.97 – 2.79 (m, 1H), 2.77 – 2.62 (m, 1.3H), 2.59 – 2.13 (m, 3.7H), 2.00 (br. s, 1H), 1.89 – 1.81 (m, 0.7H (major)), 1.58 – 1.32 (m, 4H), 1.32 – 1.05 (m, 2H), 1.01 – 0.91 (m, 9H). CO_2H not observed. Major/minor isomers reported where separation of signals observed.

^{13}C NMR (101 MHz, $CDCl_3$) δ 175.5, 168.5, 168.5, 168.4, 167.7, 167.7, 167.6, 167.1, 138.2, 138.1, 138.1, 138.0, 135.4, 135.3, 135.2, 61.6, 56.8, 56.8, 45.7, 41.4, 41.3, 39.5, 39.4, 38.3, 38.1, 37.9, 37.8, 37.8, 37.7, 37.6, 37.5, 30.1, 30.0, 29.6, 29.5, 29.4, 28.3, 26.9, 25.7, 25.7, 25.4, 25.4, 15.8, 15.7, 11.7, 11.7, 11.5, 11.4, 11.4.

HRMS: exact mass calculated for $[M-H]^-$ ($C_{19}H_{29}N_2O_4$) requires *m/z* 349.2133, found *m/z* 349.2123.

Compound S69.

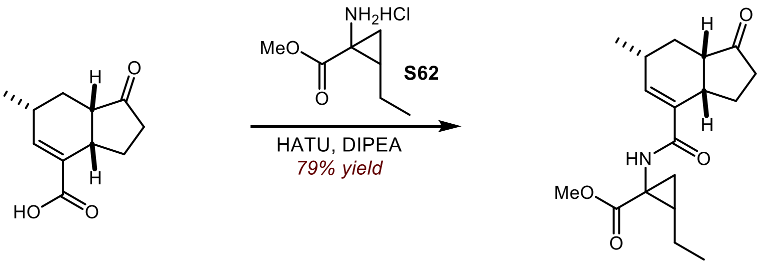

Prepared according to General Procedure F using compound **S30** (10 mg, 0.05 mmol, 1 equiv.), HATU (23 mg, 0.06 mmol, 1.2 equiv.), compound **S62** (14 mg, 0.08 mmol, 1.5 equiv.), DIPEA (30 µL, 0.17 mmol, 3 equiv.), and DMF (0.3 mL). After 16 h the reaction was subjected to purification outlined in General Procedure F (silica gel, 30% EtOAc/CH_2Cl_2) afford the title compound as a colourless oil (13 mg, 79%).

TLC (30% EtOAc/CH_2Cl_2): R_f = 0.34 stained by $KMnO_4$ and faintly visible by UV (short wave).

υ_{max} (film): 3320 (br.), 2956, 2922, 2870, 2852, 1732, 1658, 1625, 1515, 1162 cm^{-1}.

^{1}H NMR (400 MHz, $CDCl_3$): δ 6.30 (s, 1H), 6.24 – 6.18 (m, 1H), 3.72 – 3.68 (m, 3H), 3.23 – 3.13 (m, 1H), 2.52 – 2.21 (m, 5H), 1.88 – 1.80 (m, 1H), 1.67 – 1.55 (m, 4H), 1.53 – 1.44 (m, 1H), 1.32 – 1.26 (m, 1H), 1.12 – 0.96 (m, 7H).

^{13}C NMR (101 MHz, $CDCl_3$): δ 220.2, 220.2, 171.7, 169.3, 169.2, 137.7, 137.7, 136.0, 135.9, 52.5, 52.5, 46.6, 46.6, 38.4, 38.3, 36.0, 36.0, 33.3, 33.1, 30.8, 30.8, 28.7, 27.9, 27.8, 23.3, 23.1, 20.9, 20.6, 13.6.

HRMS: exact mass calculated for $[M+H]^+$ ($C_{18}H_{26}NO_4$) requires *m/z* 320.1856, found *m/z* 320.1857.

Compound 41a.

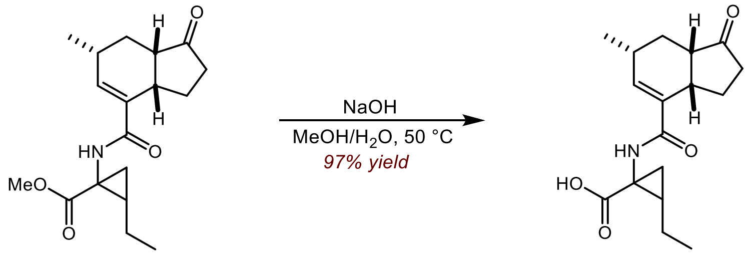

Prepared according to General Procedure G using compound **S69** (13 mg, 0.04 mmol, 1 equiv.), NaOH (5 mg, 0.13 mmol, 3 equiv.), and 1:1 MeOH/H_2O (2 mL). After 16 h the reaction was subjected to purification outlined in General Procedure G to afford the title compound as a white solid (12 mg, 97%).

υ_{max} (film): 3339 (br.), 2950, 2935, 2870, 1731, 1656, 1625, 1519, 1178 cm^{-1}.

^{1}H NMR (500 MHz, $CDCl_3$): δ 6.60 – 6.48 (m, 1H), 6.33 – 6.24 (m, 1H), 4.68 (br. s, 1H), 3.22 – 3.09 (m, 1H), 2.51 – 2.42 (m, 1H), 2.42 – 2.23 (m, 4H), 1.92 – 1.80 (m, 1H), 1.69 – 1.53 (m, 4H), 1.53 – 1.40 (m, 1H), 1.32 – 1.26 (m, 1H), 1.13 – 0.96 (m, 7H).

^{13}C NMR (101 MHz, $CDCl_3$): δ 220.3, 220.1, 175.1, 174.5, 170.7, 170.3, 139.6, 138.9, 135.3, 135.1, 46.6, 46.6, 39.0, 38.7, 38.3, 36.0, 35.9, 33.9, 33.8, 30.9, 30.9, 28.6, 28.6, 27.9, 27.8, 22.7, 22.3, 20.9, 20.8, 20.8, 13.6, 13.5.

HRMS: exact mass calculated for $[M-H]^-$ ($C_{17}H_{22}NO_4$) requires *m/z* 304.1554, found *m/z* 304.1551.

Compound S70.

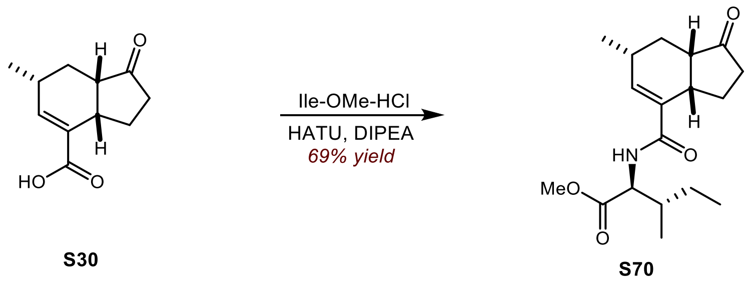

Prepared according to General Procedure F using compound **S30** (20 mg, 0.10 mmol, 1 equiv.), HATU (51 mg, 0.12 mmol, 1.2 equiv.), *L*-isoleucine methyl ester hydrochloride (28 mg, 0.15 mmol, 1.5 equiv.), DIPEA (60 µL, 0.34 mmol, 3 equiv.), and DMF (0.5 mL). After 16 h the reaction was subjected to the purification outlined in General Procedure F (silica gel, 30% EtOAc/CH_2Cl_2) to afford the title compound as a pale yellow oil (23 mg, 69%).

TLC (30% EtOAc/CH_2Cl_2): R_f = 0.56 stained by $KMnO_4$ and faintly visible by UV (short wave).

υ_{max} (film): 3346 (br.), 2961, 2933, 2874, 1735, 1659, 1622, 1513, 1199, 1147 cm^{-1}.

^{1}H NMR (400 MHz, $CDCl_3$): δ 6.34 – 6.23 (m, 2H), 4.71 – 4.66 (m, 1H), 3.77 – 3.73 (m, 3H), 3.22 – 3.09 (m, 1H), 2.53 – 2.22 (m, 5H), 1.98 – 1.89(m, 1H), 1.88 – 1.81 (m, 1H), 1.65 – 1.52 (m, 1H), 1.52 – 1.39 (m, 1H), 1.28 – 1.14 (m, 1H), 1.13 – 1.00 (m, 4H), 0.97 – 0.89 (m, 6H).

^{13}C NMR (101 MHz, $CDCl_3$): δ 220.1, 220.1, 172.9, 167.9, 167.8, 138.1, 138.0, 135.7, 135.6, 56.5, 56.5, 52.3, 46.7, 38.3, 38.3, 38.2, 36.0, 36.0, 30.9, 30.8, 28.7, 28.0, 27.9, 25.5, 25.4, 20.9, 15.7, 15.6, 11.7, 11.7.

HRMS: exact mass calculated for $[M+H]^+$ ($C_{18}H_{28}NO_4$) requires *m/z* 322.2013, found *m/z* 322.2012.

Compound 41b.

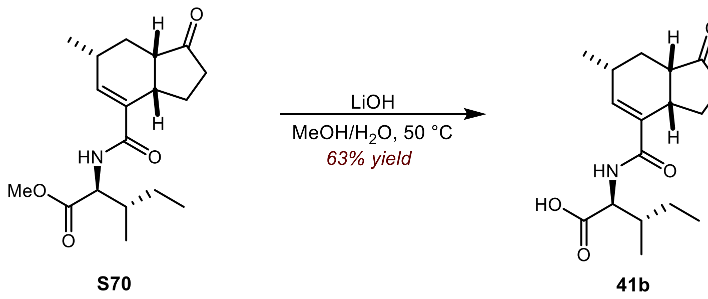

Prepared according to General Procedure G using compound **S70** (20 mg, 0.06 mmol, 1 equiv.), LiOH (5 mg, 0.21 mmol, 3 equiv.), and 1:1 MeOH:H_2O (5 mL) and the resulting suspension brought to 50 °C for 16 h. The reaction was allowed to cool to room temperature, acidified with AcOH and the organics extracted with EtOAc (3 x 5 mL). The organics were combined, dried over Na_2SO_4, filtered, and evaporated to afford a colourless oil. The crude material was loaded in a solution of CH_2Cl_2 and was purified by flash silica column chromatography, eluent 1% AcOH, 30% EtOAc/CH_2Cl_2 to afford a colourless oil. The material was washed with petroleum ether to afford the title compound as a colourless oil (12 mg, 63%).

TLC (1% AcOH, 30% EtOAc/CH_2Cl_2): R_f = 0.27 stained by $KMnO_4$ and faintly visible by UV (short wave).

υ_{max} (film): 3337 (br.), 2958, 2921, 2870, 2850, 1731, 1654, 1613, 1519, 1195, 1149 cm^{-1}.

^{1}H NMR (500 MHz, $CDCl_3$): δ 6.35 – 6.30 (m, 1H), 6.29 – 6.23 (m, 1H), 4.72 – 4.67 (m, 1H), 3.22 – 3.10 (m, 1H), 2.53 – 2.25 (m, 5H), 2.07 – 1.98 (m, 1H), 1.89 – 1.83 (m, 1H), 1.67 – 1.49 (m, 2H), 1.30 – 1.19 (m, 1H), 1.15 – 1.03 (m, 4H), 1.02 – 0.94 (m, 6H). CO_2H not observed.

^{13}C NMR (101 MHz, $CDCl_3$): δ 220.2, 220.1, 175.3, 175.3, 168.4, 168.3, 138.6, 138.5, 135.5, 135.4, 56.6, 46.7, 38.3, 37.9, 37.8, 36.0, 30.9, 30.9, 28.7, 28.0, 27.9, 25.4, 25.3, 20.9, 15.8, 15.7, 11.7, 11.7.

HRMS: exact mass calculated for [M-H]⁻ ($C_{17}H_{24}NO_4$) requires *m/z* 306.1711, found *m/z* 306.1706.

Synthesis of Compound S73.

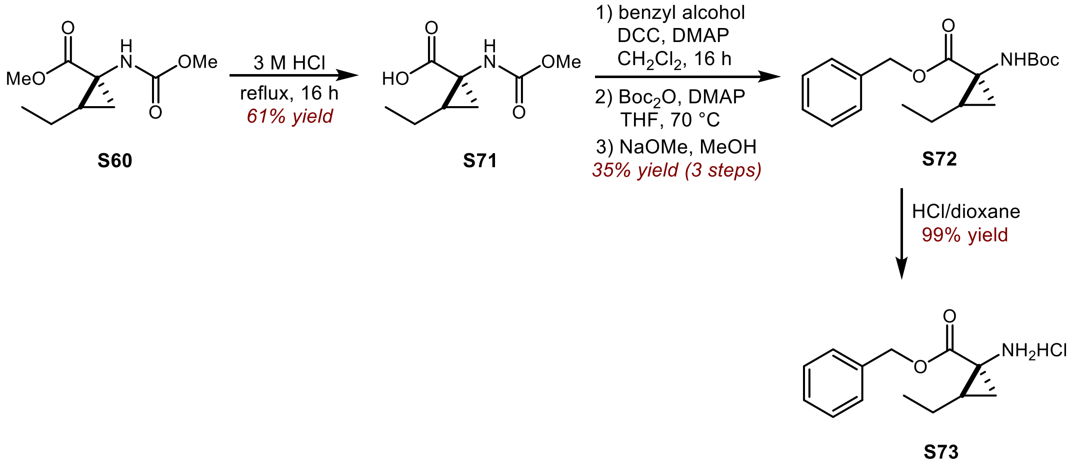

Compound S71.

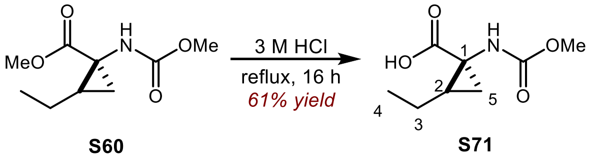

To a round bottom flask was added compound **S60** (894 mg, 4.44 mmol) and 3 M HCl (16 mL). The reaction was brought to 100 °C for 16 h. The reaction was allowed to cool to room temperature and extracted with EtOAc (3 x 20 mL). The organics were combined, washed with brine (20 mL), dried over Na_2SO_4, filtered, and evaporated to afford a pale yellow oil. The crude material was loaded in a solution of 50% EtOAc/petroleum ether and purified by flash silica column chromatography, eluent 50-60% EtOAc/petroleum ether to afford the title compound as a colourless oil which solidified to a white solid on standing (508 mg, 61%).

TLC (50% EtOAc/petroleum ether): R_f = 0.14 stained by $KMnO_4$.

υ_{max} (neat): 3331, 2958 (br.), 2874, 1703, 1686, 1526, 1268, 1191 cm^{-1}.

^{1}H NMR (400 MHz, DMSO-d$_6$): δ 12.33 (s, 1H, N*H*/O*H*), 7.73 (s, 1H, N*H*/O*H*), 3.50 (s, 3H, OC*H*$_3$), 1.59 – 1.40 (m, 2H, H^3), 1.40 – 1.30 (m, 1H, H^2), 1.26 – 1.18 (m, 1H, H^5), 1.08 – 0.98 (m, 1H, H$^{5'}$), 0.91 (t, *J* = 7.3 Hz, 3H, H^4).

^{13}C NMR (101 MHz, DMSO-d$_6$): δ 173.2 (*C*O), 156.7 (*C*O), 51.1 (O*C*H$_3$), 37.9 (C^1), 31.4 (C^2), 22.1 (C^5), 20.0 (C^3), 13.4 (C^4).

HRMS: exact mass calculated for [M-H]⁻ ($C_8H_{12}NO_4$) requires *m/z* 186.0772, found *m/z* 186.0776.

Compound S72.

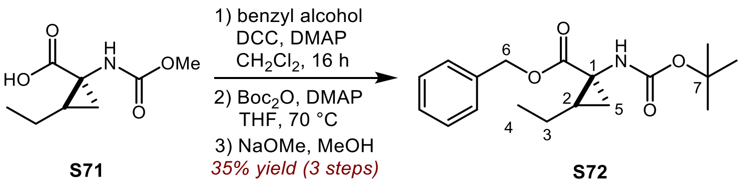

To a round bottom flask charged with **S71** (300 mg, 1.60 mmol, 1 equiv.) and CH_2Cl_2/DMF (3:1, 8 mL) was added DMAP (20 mg, 0.16 mmol, 0.1 equiv.), DCC (364 mg, 1.76 mmol, 1.1 equiv.), and benzyl alcohol (0.18 mL, 1.76 mmol, 1.1 equiv.).

The reaction was stirred at room temperature for 16 h before being diluted with H_2O (20 mL) and extracted with EtOAc (3 x 20 mL). The organics were combined, washed with brine (20 mL), dried over Na_2SO_4, filtered, and evaporated to afford a colourless residue. The crude material was loaded in a solution of 20% EtOAc/petroleum ether and purified by flash silica column chromatography, eluent 20% EtOAc/petroleum ether to afford a colourless oil which was not characterised. The colourless oil was dissolved in THF (3 mL) in a round bottom flask. Boc_2O (454 mg, 2.08 mmol, 1.3 equiv.) and DMAP (39 mg, 0.32 mmol, 0.2 equiv.) were added and the reaction was brought to 70 °C for 16 h under an atmosphere of nitrogen. The reaction was then allowed to cool to room temperature and diluted with anhydrous MeOH (2 mL). To a separate round bottom flask charged with anhydrous MeOH (2 mL) was added Na metal (11 mg, 0.48 mmol, 0.3 equiv.) under an atmosphere of nitrogen. The resulting solution was then added dropwise to the reaction flask at 0 °C. The reaction was allowed to rise to room temperature and stirred for 1.5 h. The reaction was diluted with water (20 mL) and extracted with EtOAc (3 x 20 mL). The organics were combined, washed with brine (20 mL), dried over Na_2SO_4, filtered, and evaporated to afford a pale orange oil. The crude material was loaded in a solution of 15% EtOAc/petroleum ether and purified by flash silica column chromatography, eluent 15-20% EtOAc/petroleum ether to afford the title compound as a pale yellow oil (177 mg, 35% over three steps).

TLC (20% EtOAc/petroleum ether): $R_f = 0.46$ stained by $KMnO_4$.

υ_{max} (neat): 3398 (br.), 3363 (br.), 2974, 1734, 1560, 1498, 1389, 1366, 1164 cm^{-1}.

^{1}H NMR (500 MHz, $CDCl_3$): δ 7.38 – 7.27 (m, 5H, Ar*H*), 5.22 – 5.08 (m, 2H, H^6), 1.62 – 1.49 (m, 3H, H^3, H^5), 1.48 – 1.42 (m, 1H, H^2), 1.39 (s, 9H, *t*Bu), 1.32 – 1.24 (m, 1H, $H^{5'}$), 0.91 (t, *J* = 7.4 Hz, 3H, H^4). N*H* not observed.

^{13}C NMR (101 MHz, $CDCl_3$): δ 171.7 (*C*O), 155.9 (*C*O), 136.0 (Ar), 128.5 (Ar), 128.1 (Ar), 79.8 (br., C^7), 66.9 (C^6), 38.8 (br., C^1), 33.6 (br., C^2), 28.3 (CH_3), 23.4 (br., C^5), 20.4 (C^3), 13.6 (C^4). Five signals not observed.

HRMS: exact mass calculated for $[M+H]^+$ ($C_{18}H_{26}NO_4$) requires *m/z* 320.1856, found *m/z* 320.1859.

The spectral data were consistent with those previously reported in the literature.[19]

Compound S73.

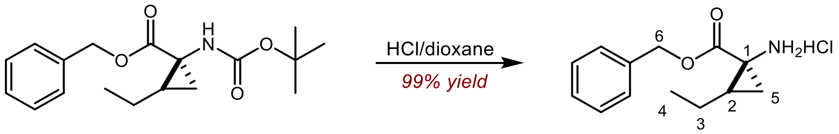

To a round bottom flask charged with compound **S72** (167 mg, 0.52 mmol) was added dioxane (1 mL), followed by dropwise addition of 6 M HCl (1 mL). The reaction was stirred at room temperature for 3 h, before the addition of further 6 M HCl (1 mL). The reaction was stirred at room temperature for a further 1 h, before being concentrated *in vacuo* to afford the title compound as a pale brown solid (132 mg, 99%).

υ_{max} (neat): 3411 (br.), 2961 (br.), 2874 (br.), 2683 (br.), 1727, 1455, 1355, 1262, 1190, 1169 cm^{-1}.

^{1}H NMR (400 MHz, $CDCl_3$): δ 9.03 (br. s, 2H), 7.41 – 7.36 (m, 2H, Ar*H*), 7.36 – 7.27 (m, 3H, Ar*H*), 5.22 (s, 2H, H^6), 2.02 – 1.92 (m, 1H, H^2), 1.85 – 1.76 (m, 1H, H^5), 1.64 – 1.44 (m, 2H, H^3), 1.44 – 1.36 (m, 1H, $H^{5'}$), 0.87 (t, *J* = 7.4 Hz, 3H, H^4).

^{13}C NMR (101 MHz, $CDCl_3$): δ 168.2 (*C*O), 134.8 (Ar), 128.7 (Ar), 128.7 (Ar), 128.6 (Ar), 68.2 (C^6), 38.5 (C^1), 30.5 (C^2), 20.0 (C^5, C^3), 13.3 (C^4). Two signals equivalent, one signal coincident.

HRMS: exact mass calculated for $[M+H]^+$ ($C_{13}H_{18}NO_2$) requires *m/z* 220.1332, found *m/z* 220.1331.

Compound S74.

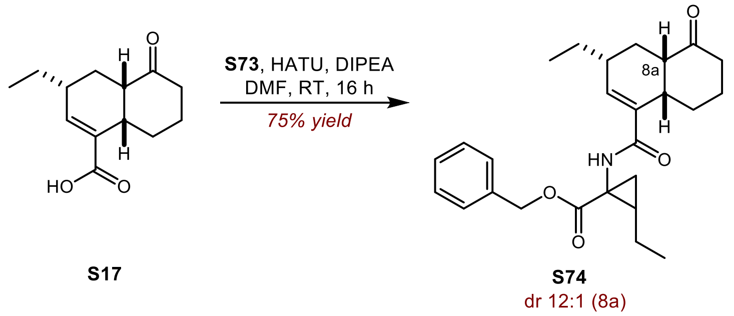

Prepared according to General Procedure F using compound **S17** (50 mg, 0.22 mmol, 1 equiv.), HATU (111 mg, 0.29 mmol, 1.3 equiv.), compound **S73** (86 mg, 0.34 mmol, 1.5 equiv.), DIPEA (0.12 mL, 0.69 mmol, 3 equiv.), and DMF (1.7 mL). After 16 h the reaction was subjected to purification outlined in General Procedure F (silica gel, 10% EtOAc/CH_2Cl_2) to afford the title compound as an orange oil (71 mg, 75%), (dr 12:1 C^{8a}).

TLC (10% EtOAc/CH_2Cl_2): R_f= 0.28 stained by $KMnO_4$ and faintly visible by UV (short wave).

υ_{max} (film): 3307 (br.), 2958, 2928, 2870, 1723, 1703, 1658, 1627, 1500, 1455, 1327, 1264, 1158 cm^{-1}.

^{1}**H NMR** (400 MHz, $CDCl_3$): δ 7.36 – 7.27 (m, 5H), 6.36 – 6.31 (m, 1H), 6.17 – 6.10 (m, 1H), 5.22 – 5.03 (m, 2H), 2.96 – 2.85 (m, 1H), 2.51 – 2.41 (m, 1H), 2.35 – 2.24 (m, 2H), 2.18 – 2.06 (m, 1H), 1.93 – 1.77 (m, 2H), 1.76 – 1.66 (m, 1H), 1.66 – 1.53 (m, 4H), 1.53 – 1.19 (m, 6H), 1.00 – 0.83 (m, 6H).

^{13}**C NMR** (101 MHz, $CDCl_3$): δ 214.3, 214.3, 171.1, 171.1, 169.5, 169.4, 137.7, 137.6, 135.8, 135.7, 128.5, 128.4, 128.4, 128.3, 67.3, 67.2, 49.9, 49.8, 38.6, 38.4, 38.3, 38.0, 36.8, 36.7, 33.5, 33.2, 28.3, 28.0, 28.0, 27.2, 27.2, 24.8, 23.4, 23.2, 20.6, 20.5, 13.6, 11.4, 11.3.

HRMS: exact mass calculated for $[M+H]^+$ ($C_{26}H_{34}NO_4$) requires *m/z* 424.2482, found *m/z* 424.2481.

Compound 43a.

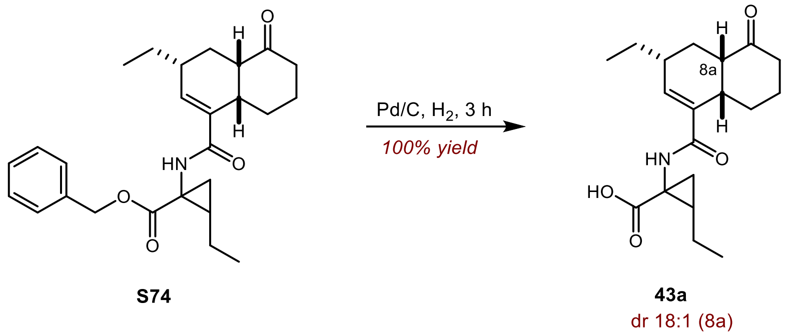

To a round bottom flask charged with compound **S74** (66 mg, 0.16 mmol, 1 equiv.) was added 10% Pd/C (30 mg, 0.03 mmol, 20 mol%) and EtOAc (3 mL). The reaction was sparged with H_2 (balloon) for 1 minute, and stirred under an atmosphere of H_2 (balloon) for 3 h. The reaction was filtered through celite, eluting with EtOAc. The organics were concentrated *in vacuo* to afford a colourless oil, which was taken up in a minimal volume of diethyl ether, and petroleum ether added until a white precipitate formed. The solvent was removed using a Pasteur pipette and the precipitate dried under vacuum to afford the desired product as a white solid (52 mg, 100%), (dr 18:1 C^{8a}).

υ_{max} (film): 3296 (br.), 2958, 2924, 2870, 1693, 1656, 1625, 1513, 1169 cm^{-1}.

¹H NMR (400 MHz, Acetone-d$_6$): δ 8.04 – 7.90 (m, 1H), 6.47 – 6.34 (m, 1H), 3.00 – 2.91 (m, 1H), 2.54 – 2.29 (m, 2H), 2.25 – 2.13 (m, 2H), 2.00 – 1.90 (m, 2H), 1.69 – 1.24 (m, 10H), 1.21 – 1.06 (m, 1H), 1.01 – 0.91 (m, 6H). One signal not observed.

¹³C NMR (101 MHz, Acetone-d$_6$): δ 213.1, 213.0, 172.9, 172.7, 170.2, 169.9, 138.1, 137.9, 136.6, 136.1, 50.9, 38.9, 38.9, 38.8, 37.5, 37.5, 32.7, 32.5, 28.9, 28.9, 28.6, 28.5, 28.0, 25.6, 22.6, 22.4, 21.4, 21.3, 13.7, 11.4.

HRMS: exact mass calculated for $[M+H]^+$ ($C_{19}H_{28}NO_4$) requires *m/z* 334.2013, found *m/z* 334.2013.

Compound S75.

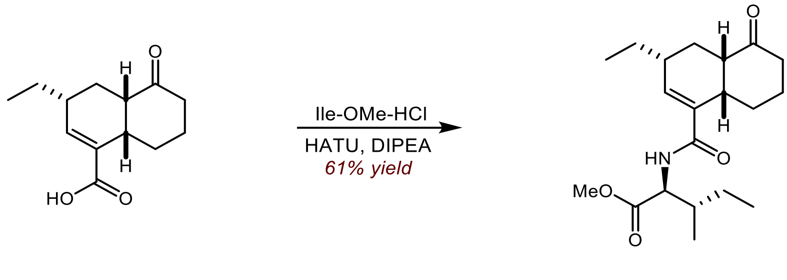

Prepared according to General Procedure F using compound **S17** (20 mg, 0.09 mmol, 1 equiv.), HATU (41 mg, 0.12 mmol, 1.2 equiv.), *L*-isoleucine methyl ester hydrochloride (25 mg, 0.14 mmol, 1.5 equiv.), DIPEA (50 µL, 0.30 mmol, 3 equiv.) and DMF (0.4 mL). After 16 h the reaction was subjected to purification outlined in General Procedure F (silica gel, 30% EtOAc/CH_2Cl_2) to afford the title compound as a pale yellow oil (19 mg, 61%).

TLC (30% EtOAc/CH_2Cl_2): R_f= 0.63 stained by $KMnO_4$ and faintly visible by UV (short wave).

υ_{max} (film): 3321 (br.), 2959, 2930, 2874, 1740, 1703, 1657, 1624, 1518, 1200, 1150 cm^{-1}.

¹H NMR (400 MHz, $CDCl_3$): δ 6.34 – 6.25 (m, 1H), 6.25 – 6.20 (m, 1H), 4.70 – 4.61 (m, 1H), 3.78 – 3.73 (m, 3H), 3.01 – 2.90 (m, 1H), 2.56 – 2.49 (m, 1H), 2.43 – 2.31 (m, 2H), 2.27 – 2.15 (m, 1H), 2.06 – 1.87 (m, 3H), 1.81 – 1.62 (m, 2H), 1.57 – 1.35 (m, 5H), 1.27 – 1.13 (m, 1H), 1.01 – 0.89 (m, 9H).

¹³C NMR (101 MHz, $CDCl_3$): δ 213.7, 213.6, 172.3, 172.3, 167.6, 167.3, 136.9, 136.7, 135.5, 135.4, 56.0, 55.9, 51.7, 49.3, 49.3, 38.0, 37.6, 37.5, 37.4, 36.3, 27.8, 27.8, 27.5, 27.4, 26.9, 26.8, 24.9, 24.8, 24.2, 24.2, 15.1, 15.0, 11.1, 10.8, 10.8.

HRMS: exact mass calculated for $[M+H]^+$ ($C_{20}H_{32}NO_4$) requires *m/z* 350.2326, found *m/z* 350.2326.

Compound 43b.

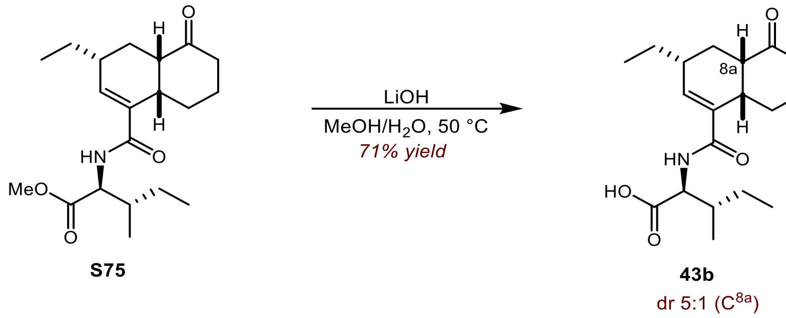

Prepared according to General Procedure G using compound **S75** (19 mg, 0.06 mmol, 1 equiv.), LiOH (5 mg, 0.21 mmol, 3 equiv.), and 1:1 MeOH:H_2O (5 mL) and the resulting suspension brought to 50 °C for 16 h. The reaction was allowed to cool to room temperature, acidified with AcOH and the organics extracted with EtOAc (3 x 5 mL). The organics were combined, dried over Na_2SO_4, filtered, and evaporated to afford a colourless oil. The crude material was loaded in a solution of CH_2Cl_2

and was purified by flash silica column chromatography, eluent 1% AcOH, 30% EtOAc/CH_2Cl_2 to afford a colourless oil. The material was washed with petroleum ether to afford the title compound as a colourless oil (13 mg, 71%). dr 5:1 C^{8a}.

TLC (1% AcOH, 30% EtOAc/CH_2Cl_2): R_f = 0.63 stained by $KMnO_4$ and faintly visible by UV (short wave).

υ_{max} (film): 3325 (br.), 2959, 2924, 2872, 1701, 1655, 1616, 1522, 1231, 1152 cm^{-1}.

^{1}H NMR (500 MHz, $CDCl_3$): δ 6.35 – 6.28 (m, 1H), 6.26 – 6.20 (m, 1H), 4.69 – 4.63 (m, 1H), 3.01 – 2.90 (m, 1H), 2.57 – 2.51 (m, 1H), 2.41 – 2.33 (m, 2H), 2.27 – 2.19 (m, 1H), 2.06 – 1.93 (m, 3H), 1.81 – 1.76 (m, 1H), 1.75 – 1.64 (m, 1H), 1.59 – 1.36 (m, 4H), 1.35 – 1.27 (m, 2H), 1.02 – 0.94 (m, 9H). CO_2H not observed. Minor isomerisation to *trans*-ring junction observed.

^{13}C NMR (126 MHz, $CDCl_3$): δ 214.4, 214.4, 175.4, 168.6, 168.4, 137.3, 137.1, 136.6, 56.8, 56.7, 49.9, 38.6, 38.2, 38.1, 37.8, 37.7, 36.9, 28.4, 28.4, 28.1, 28.0, 27.5, 27.4, 25.4, 25.3, 24.8, 24.8, 15.8, 15.7, 11.7, 11.5, 11.4.

HRMS: exact mass calculated for $[M+H]^+$ ($C_{19}H_{30}NO_4$) requires *m/z* 336.2175, found *m/z* 336.2177.

Compound 44a.

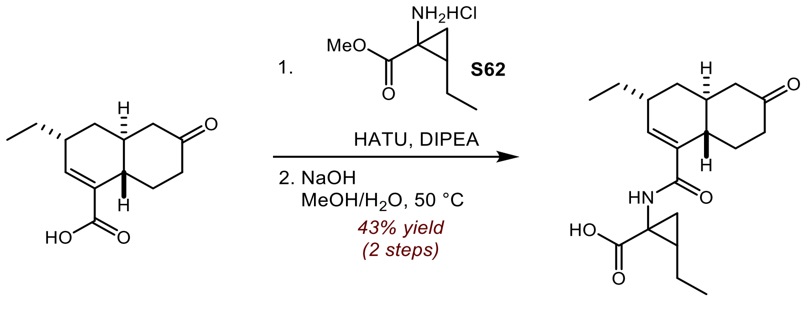

To a 2-dram vial was added compound **S24** (10 mg, 0.04 mmol, 1 equiv.) and HATU (21 mg, 0.06 mmol, 1.2 equiv.). DMF (0.2 mL) was added, followed by DIPEA (20 µL, 0.11 mmol, 3 equiv.) and the resulting solution stirred at room temperature for 5 minutes. Compound **S62** (12 mg, 0.07 mmol, 1.5 equiv.) was then added in one portion and the vial capped with a screw top lid. The reaction was stirred for 16 h under air. The reaction was then diluted with H_2O (10 mL) and the organics extracted with EtOAc (3 x 5 mL). The organics were combined, washed with brine (10 mL), dried over Na_2SO_4, filtered, and evaporated to afford a pale yellow oil. The crude material was loaded in a solution of CH_2Cl_2 and purified by flash silica column chromatography, eluent 30% EtOAc/CH_2Cl_2 to afford a pale yellow oil which was taken up in 1:1 MeOH/H_2O (1.5 mL) and NaOH (5 mg, 0.13 mmol, 3 equiv.) added. The reaction was brought to 50 °C for 16 h. The reaction was then subjected to purification outlined in General Procedure G to afford the title compound as an orange solid (7 mg, 43% (2 steps)).

υ_{max} (film): 3289 (br.), 2958, 2930, 1697, 1625, 1509, 1400, 1307 cm^{-1}.

^{1}H NMR (500 MHz, $CDCl_3$): δ 6.38 – 6.31 (m, 1H), 6.27 – 6.17 (m, 1H), 2.55 – 2.47 (m, 1H), 2.47 – 2.38 (m, 3H), 2.38 – 2.31 (m, 1H), 2.26 – 2.14 (m, 2H), 1.70 – 1.51 (m, 6H), 1.51 – 1.40 (m, 2H), 1.36 – 1.20 (m, 3H), 1.06 – 1.00 (m, 3H), 0.96 (t, J = 7.4 Hz, 3H). CO_2H not observed.

^{13}C NMR (101 MHz, $CDCl_3$): δ 211.1, 210.8, 174.8, 173.8, 172.6, 172.0, 137.5, 137.2, 137.2, 135.9, 48.2, 48.2, 41.6, 41.5, 40.9, 40.9, 39.1, 38.5, 36.5, 36.4, 36.3, 34.0, 33.9, 33.0, 32.9, 29.1, 28.1, 28.0, 22.7, 22.1, 21.0, 20.8, 13.6, 13.5, 12.5.

HRMS: exact mass calculated for $[M-H]^-$ ($C_{19}H_{26}NO_4$) requires *m/z* 332.1867, found *m/z* 332.1863.

Compound S76.

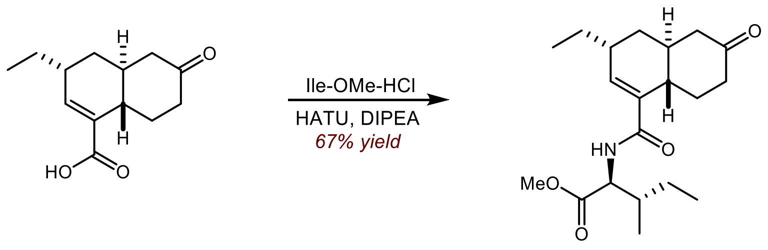

S24 S76

Prepared according to General Procedure F using compound **S24** (20 mg, 0.09 mmol, 1 equiv.), HATU (41 mg, 0.12 mmol, 1.2 equiv.), *L*-isoleucine methyl ester hydrochloride (25 mg, 0.14 mmol, 1.5 equiv.), DIPEA (50 µL, 0.30 mmol, 3 equiv.), and DMF (0.4 mL). After 16 h the reaction was subjected to purification outlined in General Procedure F (silica gel, 30% EtOAc/CH_2Cl_2) to afford the title compound as a pale yellow oil (21 mg, 67%).

TLC (30% EtOAc/CH_2Cl_2): R_f = 0.42 stained by $KMnO_4$ and faintly visible by UV (short wave).

υ_{max} (film): 3314 (br.), 2959, 2924, 2874, 1742, 1713, 1657, 1624, 1518 cm^{-1}.

^{1}H NMR (500 MHz, $CDCl_3$): δ 6.25 – 6.19 (m, 1H), 6.13 – 6.08 (m, 1H), 4.68 – 4.61 (m, 1H), 3.76 – 3.73 (m, 3H), 2.55 – 2.46 (m, 1H), 2.47 – 2.33 (m, 4H), 2.25 – 2.15 (m, 2H), 1.97 – 1.89 (m, 1H), 1.71 – 1.62 (m, 1H), 1.57 – 1.52 (m, 2H), 1.52 – 1.39 (m, 2H), 1.37 – 1.12 (m, 3H), 1.00 – 0.89 (m, 9H).

^{13}C NMR (101 MHz, $CDCl_3$): δ 210.8, 172.8, 172.8, 169.7, 169.4, 138.1, 138.1, 135.2, 134.9, 56.4, 56.2, 52.3, 48.3, 41.6, 40.9, 40.8, 38.2, 38.0, 36.4, 36.3, 36.3, 33.0, 33.0, 29.2, 29.1, 28.0, 25.4, 25.2, 15.8, 15.7, 12.5, 11.7, 11.6.

HRMS: exact mass calculated for $[M+H]^+$ ($C_{20}H_{32}NO_4$) requires *m/z* 350.2326, found *m/z* 350.2326.

Compound 44b.

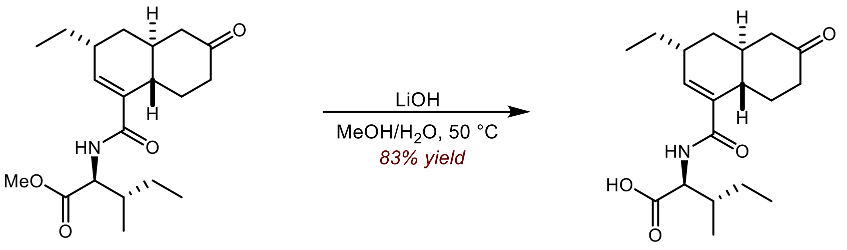

S76 44b

Prepared according to General Procedure G using compound **S76** (20 mg, 0.06 mmol, 1 equiv.), LiOH (5 mg, 0.21 mmol, 3 equiv.), and 1:1 MeOH:H_2O (3 mL). After 16 h the reaction was allowed to cool to room temperature, acidified with AcOH, and the organics extracted with EtOAc (3 x 5 mL). The organics were combined, dried over Na_2SO_4, filtered and evaporated to afford a colourless oil. The crude material was loaded in a solution of CH_2Cl_2 and was purified by flash silica column chromatography, eluent 1% AcOH, 30% EtOAc/CH_2Cl_2 to afford a colourless oil. The material was washed with petroleum ether to afford the title compound as a colourless oil (16 mg, 83%).

TLC (1% AcOH, 30% EtOAc/CH_2Cl_2): R_f = 0.76 and 0.66 stained by $KMnO_4$ and faintly visible by UV (short wave). Separation of isomers observed.

υ_{max} (film): 3310 (br.), 2961, 2922, 2872, 1711, 1655, 1611, 1522, 1202 cm^{-1}.

^{1}H NMR (500 MHz, $CDCl_3$): δ 6.29 – 6.17 (m, 2H), 4.68 – 4.55 (m, 1H), 2.54 – 2.46 (m, 1H), 2.47 – 2.32 (m, 4H), 2.26 – 2.15 (m, 2H), 1.99 (br. s, 1H), 1.72 – 1.61 (m, 1H), 1.60 – 1.43 (m, 4H), 1.38 – 1.15 (m, 3H), 1.02 – 0.90 (m, 9H). $CO_2\mathit{H}$ not observed.

^{13}**C NMR** (101 MHz, $CDCl_3$): δ 211.3, 211.1, 175.6, 170.5, 170.0, 137.8, 135.7, 135.4, 56.8, 56.7, 48.2, 41.5, 40.8, 40.7, 37.7, 37.6, 36.4, 36.4, 36.3, 32.9, 29.1, 28.0, 25.4, 25.2, 15.8, 15.7, 12.5, 11.7, 11.7.

HRMS: exact mass calculated for $[M-H]^-$ ($C_{19}H_{28}NO_4$) requires *m/z* 334.2024, found *m/z* 334.2018.

Synthesis of compound S81.

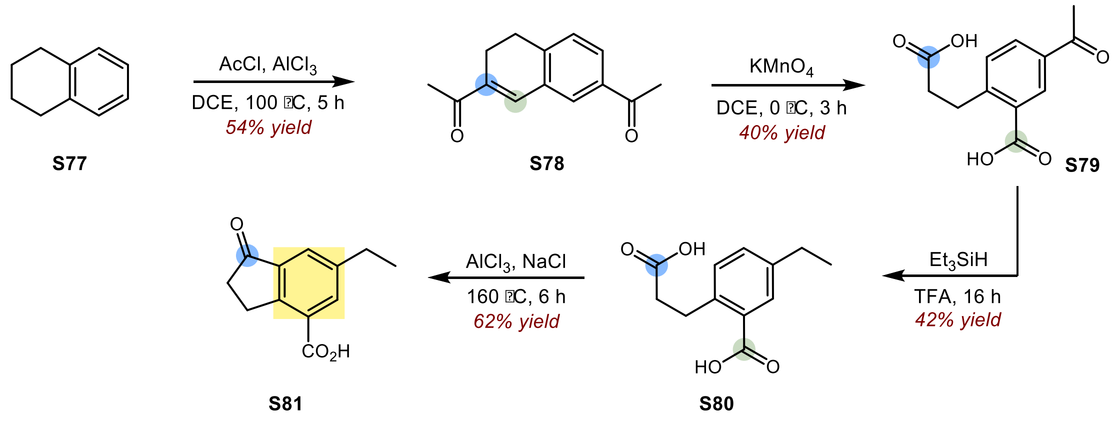

Compound S78.

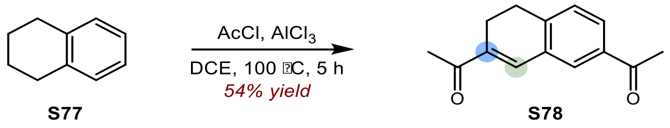

To a round bottom flask fitted was added $AlCl_3$ (20 g, 0.15 mol, 4 equiv.) and DCE (12.5 mL). AcCl (8.05 mL, 0.11 mol, 3 equiv.) was added dropwise to the stirring suspension at room temperature. A small exotherm was observed. A solution of 1,2,3,4-tetrahydronapthalene (**S77**) (5.15 mL, 0.04 mol, 1 equiv.) in DCE (6 mL) was then added dropwise. A second exotherm was observed. The reaction was stirred for 5 minutes and the solvent removed *in vacuo* to afford a viscous residue. The residue was then heated to 100 °C for 5 h. The reaction was cooled to 0 °C in an ice bath before being quenched slowly with water (100 mL) and $NaHCO_3$ (aq.) (100 mL). On quenching a dark brown precipitate was formed. The precipitate was extracted into EtOAc (3 x 100 mL). The organics were combined, washed with brine (30 mL), dried over Na_2SO_4, filtered, and evaporated to afford a viscous dark red/brown oil. The crude material was loaded in a solution of 30% EtOAc/petroleum ether and purified by flash silica column chromatography, eluent 30% EtOAc/petroleum ether to afford an orange oil which solidified to an orange solid on standing. The solid was triturated with diethyl ether to afford the title compound as a beige solid (4.41 g, 54 %).

TLC (20% EtOAc/petroleum ether): $R_f = 0.32$ stained by $KMnO_4$ and visible by UV (short wave).

υ_{max} (neat): 2939, 2893, 1679, 1656, 1623, 1355, 1281, 1271, 1203 cm^{-1}.

^{1}**H NMR** (400 MHz, $CDCl_3$): δ 7.87 – 7.83 (m, 2H), 7.44 (s, 1H), 7.28 (d, *J* = 7.7 Hz, 1H), 2.89 (t, *J* = 8.3 Hz, 2H), 2.64 – 2.59 (m, 5H), 2.46 (s, 3H).

^{13}**C NMR** (101 MHz, $CDCl_3$): δ 198.4, 197.4, 143.1, 139.2, 136.3, 136.2, 133.1, 129.9, 128.3, 128.2, 27.8, 26.7, 25.5, 20.8.

HRMS: exact mass calculated for $[M+H]^+$ ($C_{14}H_{15}O_2$) requires *m/z* 215.1067, found *m/z* 215.1067.

The spectral data were consistent with those previously reported in the literature.[20]

Compound S79.

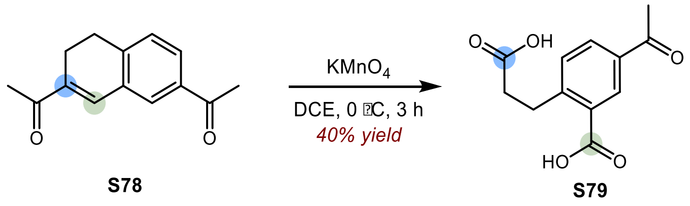

To a solution of $KMnO_4$ (4.74 g, 29.99 mmol, 1 equiv.) in water (125 mL) in a round bottom flask at 0 °C was added compound **S78** (2.57 g, 11.99 mmol, 2.5 equiv.) in a solution of DCE (5 mL) over the course of 5 minutes. The reaction was stirred at ~ 3 °C for 3 h. Powdered NaOH (~ 2.3 g) was added and the solution filtered. The solution was brought to pH 1 with HCl (aq.) and the aqueous was extracted with EtOAc (3 x 50 mL). The organics were combined, dried over Na_2SO_4, filtered, and evaporated to afford a brown solid, which was then triturated with acetone to afford the title compound as an orange solid (1.12 g, 40%).

υ_{max} (neat): 3049 (br.), 2930, 1716, 1693, 1651, 1195 cm^{-1}.

^{1}H NMR (400 MHz, DMSO-d_6): δ 12.63 (br. s, 2H), 8.32 (d, J = 2.0 Hz, 1H), 8.03 (dd, J = 8.0, 2.0 Hz, 1H), 7.50 (d, J = 8.1 Hz, 1H), 3.20 (t, J = 7.7 Hz, 2H), 2.59 (s, 3H), 2.54 (t, J = 7.7 Hz, 2H).

^{13}C NMR (101 MHz, DMSO-d_6): δ 197.0, 173.5, 147.0, 134.9, 131.3, 131.1, 129.9, 34.9, 29.0, 26.7. Two signals not observed.

HRMS: exact mass calculated for $[M-H]^-$ ($C_{12}H_{11}O_5$) requires m/z 235.0612, found m/z 235.0612.

The spectral data were consistent with those previously reported in the literature.[20]

Compound S80.

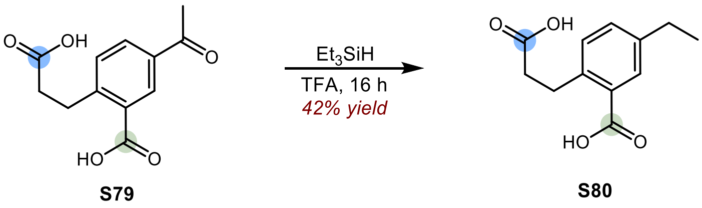

To a round bottom flask charged with compound **S79** (444 mg, 1.88 mmol, 1 equiv.) in TFA (6.5 mL) was added triethylsilane (0.85 mL, 5.32 mmol, 2.5 equiv.) dropwise and the resulting orange suspension was stirred at room temperature for 16 h under air. The solvent was removed in *vacuo* to afford a brown oil. The crude material was dry loaded onto silica gel and purified by flash silica column chromatography, eluent 2% AcOH, 30% EtOAc/petroleum ether to afford title compound as a white solid (176 mg, 42%).

TLC (2% AcOH, 30% EtOAc/PE): R_f = 0.22 stained by $KMnO_4$ and visible by UV (short wave).

υ_{max} (neat): 2963 (br.), 2932, 2872, 2634, 1682, 1403, 1277, 1210, 907 cm^{-1}.

^{1}H NMR (400 MHz, Acetone-d_6): δ 10.84 (br. s, 1H), 7.80 (d, J = 1.7 Hz, 1H), 7.37 – 7.29 (m, 2H), 3.27 – 3.21 (m, 2H), 2.70 – 2.60 (m, 4H), 1.23 (t, J = 7.6 Hz, 3H). CO_2H not observed.

^{13}C NMR (101 MHz, DMSO-d_6): δ 173.8, 168.8, 141.7, 139.1, 131.2, 130.8, 130.3, 129.5, 35.4, 28.7, 27.5, 15.4.

HRMS: exact mass calculated for $[M-H]^-$ ($C_{12}H_{13}O_4$) requires m/z 221.0819, found m/z 221.0819.

The spectral data were consistent with those previously reported in the literature.[20]

Compound S81.

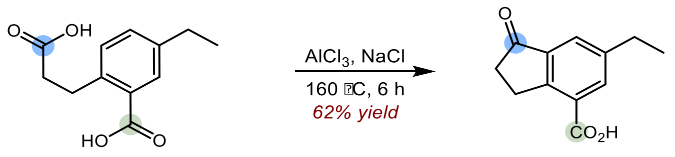

S80 **S81**

A round bottom flask charged with compound **S80** (177 mg, 0.80 mmol, 1 equiv.), $AlCl_3$ (743 mg, 5.57 mmol, 7 equiv.), NaCl (116 mg, 1.98 mmol, 2.5 equiv.) was brought to 160 °C under air and stirred for 6 h. The reaction was allowed to cool to room temperature and water (3 mL) added, followed by HCl (aq.) (0.5 mL), and the resulting suspension stirred at room temperature for 20 h. The reaction was diluted with EtOAc (10 mL) and filtered. Water (10 mL) was added and the layers separated. The organics were dried over Na_2SO_4, filtered, and evaporated to afford a beige solid. The material was dry loaded onto silica gel and purified by flash silica column chromatography, eluent 2% AcOH, 30% EtOAc/petroleum ether to afford the title compound as a white solid (101 mg, 62%).

TLC (2% AcOH, 30% EtOAc/petroleum ether): R_f = 0.35 stained by $KMnO_4$ and visible by UV (short wave).

υ_{max} (neat): 2961, 2924, 2868, 2668, 1708, 1673, 1580, 1435, 1299, 1242 cm^{-1}.

^{1}H NMR (400 MHz, $CDCl_3$) δ 8.23 (d, *J* = 1.8 Hz, 1H), 7.85 (d, *J* = 1.6 Hz, 1H), 3.54 – 3.47 (m, 2H), 2.83 – 2.72 (m, 4H), 1.30 (t, *J* = 7.6 Hz, 3H). CO_2H not observed.

^{13}C NMR (101 MHz, $CDCl_3$): δ 206.9, 171.5, 155.2, 144.5, 138.9, 137.4, 128.2, 127.3, 36.6, 28.4, 27.2, 15.5.

HRMS: exact mass calculated for $[M-H]^-$ ($C_{12}H_{11}O_3$) requires *m/z* 203.0714, found *m/z* 203.0714.

The spectral data were consistent with those previously reported in the literature.[20]

Compound S82.

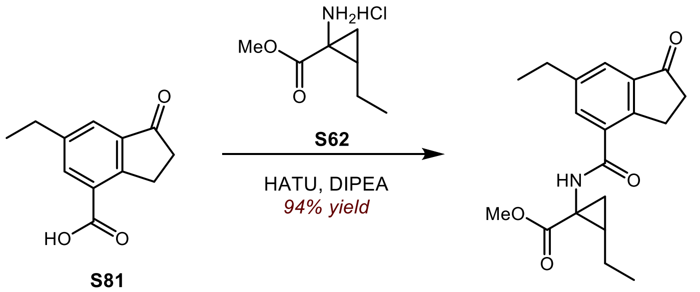

S81 **S82**

Prepared according to General Procedure F using compound **S81** (10 mg, 0.05 mmol, 1 equiv.), HATU (22 mg, 0.06 mmol, 1.2 equiv.), compound **S62** (10 mg, 0.06 mmol, 1.1 equiv.), DIPEA (30 µL, 0.17 mmol, 3 equiv.), and DMF (0.3 mL). After 16 h the reaction was subjected to purification outlined in General Procedure F (silica gel, 30% EtOAc/CH_2Cl_2) to afford the title compound as a colourless oil (15 mg, 94%).

TLC (30% EtOAc/CH_2Cl_2): R_f = 0.55 stained by $KMnO_4$ and faintly visible by UV (short wave).

υ_{max} (film): 3305 (br.), 2960, 2922, 2872, 2852, 1714, 1651, 1519, 1336, 1162 cm^{-1}.

^{1}H NMR (500 MHz, $CDCl_3$): δ 7.67 (s, 1H), 7.62 (s, 1H), 6.66 (s, 1H), 3.74 (s, 3H), 3.37 – 3.32 (m, 2H), 2.75 – 2.65 (m, 4H), 1.71 – 1.54 (m, 4H), 1.42 – 1.38 (m, 1H), 1.25 (t, *J* = 7.6 Hz, 3H), 1.01 (t, *J* = 7.3 Hz, 3H).

^{13}C NMR (101 MHz, $CDCl_3$): δ 206.8, 171.5, 168.2, 152.1, 144.4, 138.7, 132.8, 132.8, 125.4, 52.6, 38.7, 36.6, 33.3, 28.5, 25.9, 23.3, 20.6, 15.6, 13.6.

HRMS: exact mass calculated for $[M+H]^+$ ($C_{19}H_{24}NO_4$) requires *m/z* 330.1700, found *m/z* 330.1702.

Compound 45a.

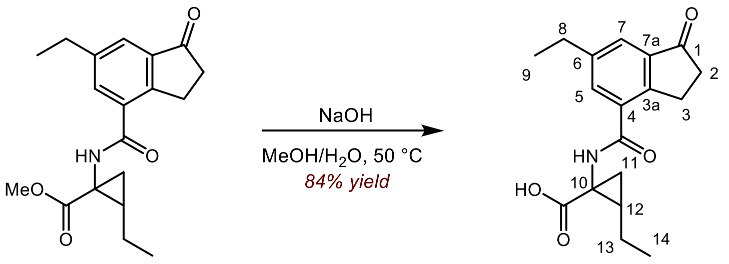

S82 **45a**

Prepared according to General Procedure G using compound **S82** (15 mg, 0.05 mmol, 1 equiv.), NaOH (7 mg, 0.18 mmol, 3 equiv.), and 1:1 MeOH/H_2O (2.5 mL). After 6 h the reaction was subjected to purification outlined in General Procedure G to afford the title compound as a white solid (12 mg, 84 %).

$\mathbf{\upsilon_{max}}$ (film): 3272 (br.), 2963, 2934, 2872, 1654, 1586, 1396, 1305 cm^{-1}.

^{1}H NMR (400 MHz, $CDCl_3$): δ 7.69 (s, 1H, $H^{5/7}$), 7.63 (s, 1H, $H^{5/7}$), 6.83 (s, 1H, N*H*), 3.40 – 3.30 (m, 2H, CH_2), 2.76 – 2.63 (m, 4H, H^8,CH_2), 1.74 – 1.55 (m, 4H, H^{13}, H^{12}, H^{11}), 1.46 – 1.38 (m, 1H, $H^{11'}$), 1.25 (t, *J* = 7.6 Hz, 3H, H^9), 1.04 (t, *J* = 7.0 Hz, 3H, H^{14}).

^{13}C NMR (101 MHz, $CDCl_3$): δ 206.8 (*C*O), 175.6 (*C*O), 169.1 (*C*O), 152.2 (Ar), 144.4 (Ar), 138.7 (Ar), 133.0 ($C^{5/7}$), 132.2 (Ar), 125.7 ($C^{5/7}$), 38.8 (C^{10}), 36.6 (*C*H_2), 34.1 (C^{12}), 28.5 (*C*H_2), 25.9 (*C*H_2), 23.2 (C^{11}), 20.7 (C^{13}), 15.5 (C^9), 13.6 (C^{14}).

HRMS: exact mass calculated for $[M-H]^-$ ($C_{18}H_{20}NO_4$) requires *m/z* 314.1398, found *m/z* 314.1394.

Coronalon Aromatic Core Amino Acid Analogues.

Reactions carried out according to General Procedure H.

Compound S83.

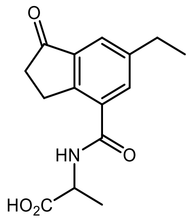

S83

^{1}H NMR (400 MHz, $CDCl_3$): δ 7.72 (d, *J* = 1.5 Hz, 1H), 7.69 (s, 1H), 6.89 (d, *J* = 6.9 Hz, 1H), 4.80 – 4.71 (m, 1H), 3.38 – 3.33 (m, 2H), 2.76 – 2.68 (m, 4H), 1.56 (d, *J* = 7.1 Hz, 3H), 1.25 (t, *J* = 7.6 Hz, 3H). CO_2*H* not observed.

^{13}C NMR (101 MHz, $CDCl_3$): δ 207.0, 175.2, 166.8, 151.7, 144.4, 138.5, 133.4, 132.7, 125.4, 48.7, 36.6, 28.5, 25.9, 18.6, 15.5.

HRMS: exact mass calculated for $[M+H]^+$ ($C_{15}H_{18}NO_4$) requires *m/z* 276.1230, found *m/z* 276.1228.

Compound S84.

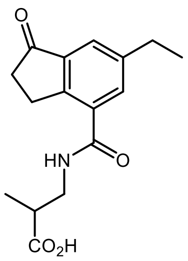

S84

^{1}H NMR (400 MHz, $CDCl_3$): δ 7.66 (app. s, 2H), 6.87 (t, *J* = 5.5 Hz, 1H), 3.77 – 3.69 (m, 1H), 3.54 – 3.45 (m, 1H), 3.35 – 3.26 (m, 2H), 2.86 – 2.76 (m, 1H), 2.75 – 2.66 (m, 4H), 1.29 – 1.18 (m, 6H). CO_2H not observed.

^{13}C NMR (101 MHz, $CDCl_3$): δ 207.0, 178.5, 167.5, 151.5, 144.4, 138.5, 133.2, 125.2, 42.0, 39.4, 36.6, 28.4, 25.8, 15.5, 15.1. One signal not observed.

HRMS: exact mass calculated for $[M+H]^+$ ($C_{16}H_{20}NO_4$) requires *m/z* 290.1387, found *m/z* 290.1384.

Compound S85.

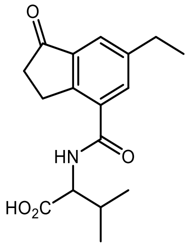

S85

^{1}H NMR (400 MHz, $CDCl_3$): δ 7.74 – 7.68 (m, 2H), 6.67 (d, *J* = 8.4 Hz, 1H), 4.78 (dd, *J* = 8.4, 4.4 Hz, 1H), 3.38 – 3.33 (m, 2H), 2.78 – 2.68 (m, 4H), 2.41 – 2.32 (m, 1H), 1.26 (t, *J* = 7.6 Hz, 3H), 1.08 – 0.98 (m, 6H). CO_2H not observed.

^{13}C NMR (101 MHz, $CDCl_3$): δ 206.9, 174.3, 167.2, 151.4, 144.5, 138.5, 133.4, 133.1, 125.4, 57.5, 36.6, 31.5, 28.5, 25.9, 19.3, 18.0, 15.5.

HRMS: exact mass calculated for $[M+H]^+$ ($C_{17}H_{22}NO_4$) requires *m/z* 304.1543, found *m/z* 304.1541.

Compound 45b.

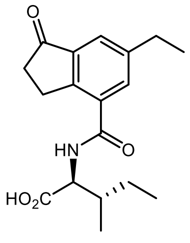

45b

^{1}H NMR (400 MHz, $CDCl_3$): δ 7.72 (d, *J* = 1.5 Hz, 1H), 7.68 (s, 1H), 6.72 (d, *J* = 8.2 Hz, 1H), 4.79 (dd, *J* = 8.2, 4.4 Hz, 1H), 3.37 – 3.31 (m, 2H), 2.77 – 2.68 (m, 4H), 2.12 – 2.02 (m, 1H), 1.63 – 1.51 (m, 1H), 1.35 – 1.20 (m, 4H), 1.03 – 0.91 (m, 6H). CO_2H not observed.

^{13}C NMR (101 MHz, $CDCl_3$): δ 206.9, 174.1, 167.0, 151.4, 144.4, 138.5, 133.4, 133.0, 125.3, 57.0, 38.1, 36.6, 28.4, 25.9, 25.4, 15.7, 15.5, 11.9.

HRMS: exact mass calculated for $[M+H]^+$ ($C_{18}H_{24}NO_4$) requires *m/z* 318.1700, found *m/z* 318.1696.

Compound S86.

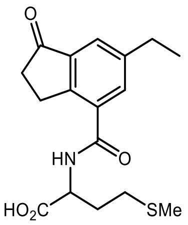

S86

^{1}H NMR (400 MHz, $CDCl_3$): δ 7.75 (d, *J* = 1.5 Hz, 1H), 7.72 (s, 1H), 7.09 (d, *J* = 7.5 Hz, 1H), 5.44 (br. s, 1H), 4.95 (td, *J* = 7.3, 5.1 Hz, 1H), 3.37 (dd, *J* = 10.8, 4.6 Hz, 2H), 2.80 – 2.63 (m, 6H), 2.41 – 2.29 (m, 1H), 2.26 – 2.15 (m, 1H), 2.13 (s, 3H), 1.26 (t, *J* = 7.6 Hz, 3H).

^{13}C NMR (101 MHz, $CDCl_3$): δ 207.1, 175.4, 167.5, 152.0, 144.6, 138.6, 133.4, 132.2, 125.9, 52.4, 36.6, 31.1, 30.4, 28.5, 26.0, 15.7, 15.5.

HRMS: exact mass calculated for $[M+H]^+$ ($C_{17}H_{22}NO_4S$) requires *m/z* 336.1264, found *m/z* 336.1261.

Compound S87.

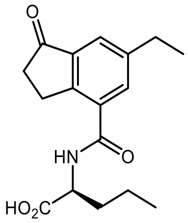

S87

^{1}H NMR (400 MHz, $CDCl_3$): δ 8.82 (br. s, 1H), 7.72 (d, *J* = 1.6 Hz, 1H), 7.68 (s, 1H), 6.79 (d, *J* = 7.5 Hz, 1H), 4.79 (td, *J* = 7.3, 5.3 Hz, 1H), 3.38 – 3.32 (m, 2H), 2.76 – 2.67 (m, 4H), 2.05 – 1.93 (m, 1H), 1.86 – 1.75 (m, 1H), 1.53 – 1.37 (m, 2H), 1.24 (t, *J* = 7.6 Hz, 3H), 0.95 (t, *J* = 7.3 Hz, 3H).

^{13}C NMR (101 MHz, $CDCl_3$): δ 207.0, 175.0, 167.0, 151.6, 144.4, 138.5, 133.4, 132.8, 125.4, 52.6, 36.6, 34.6, 28.4, 25.9, 18.7, 15.5, 13.9.

HRMS: exact mass calculated for $[M+H]^+$ ($C_{17}H_{22}NO_4$) requires *m/z* 304.1543, found *m/z* 304.1540.

Compound S88.

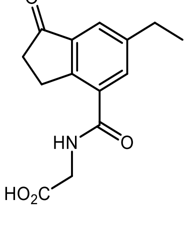

S88

^{1}H NMR (400 MHz, $CDCl_3$): δ 7.72 (d, *J* = 1.5 Hz, 1H), 7.67 (s, 1H), 4.16 (s, 2H), 3.36 – 3.31 (m, 2H), 2.78 – 2.63 (m, 4H), 1.23 (t, *J* = 7.6 Hz, 3H). N*H* and CO_2*H* not observed.

^{13}C NMR (101 MHz, $CDCl_3$): δ 207.4, 171.7, 167.6, 152.0, 144.4, 138.4, 133.4, 132.4, 125.4, 41.6, 36.6, 28.4, 25.8, 15.4.

HRMS: exact mass calculated for $[M+H]^+$ ($C_{14}H_{16}NO_4$) requires *m/z* 262.1074, found *m/z* 262.1073.

Compound S89.

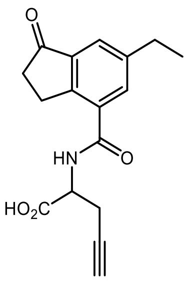

S89

^{1}H NMR (400 MHz, $CDCl_3$): δ 7.79 (d, *J* = 1.6 Hz, 1H), 7.72 (s, 1H), 7.00 (d, *J* = 7.2 Hz, 1H), 4.90 (dt, *J* = 7.3, 4.6 Hz, 1H), 3.46 – 3.32 (m, 2H), 3.06 – 2.87 (m, 2H), 2.79 – 2.69 (m, 4H), 2.06 (t, *J* = 2.6 Hz, 1H), 1.26 (t, *J* = 7.6 Hz, 3H). CO_2H not observed.
^{13}C NMR (101 MHz, $CDCl_3$): δ 206.8, 172.3, 166.8, 151.5, 144.5, 138.6, 133.7, 132.5, 125.7, 79.0, 71.78, 51.2, 36.6, 28.5, 26.0, 22.5, 15.5.
HRMS: exact mass calculated for $[M+H]^+$ ($C_{17}H_{18}NO_4$) requires *m/z* 300.1230, found *m/z* 300.1228.

Compound S90.

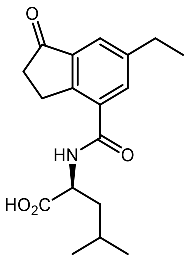

S90

^{1}H NMR (400 MHz, $CDCl_3$): δ 8.02 (br. s, 1H), 7.70 (d, *J* = 1.6 Hz, 1H), 7.66 (s, 1H), 6.72 (d, *J* = 8.0 Hz, 1H), 4.79 (td, *J* = 8.4, 4.9 Hz, 1H), 3.41 – 3.25 (m, 2H), 2.75 – 2.65 (m, 4H), 1.86 – 1.63 (m, 3H), 1.23 (t, *J* = 7.6 Hz, 3H), 1.02 – 0.92 (m, 6H).
^{13}C NMR (101 MHz, $CDCl_3$): δ 207.0, 175.3, 167.2, 151.7, 144.3, 138.4, 133.3, 132.9, 125.3, 51.3, 41.7, 36.6, 28.4, 25.82, 25.15, 22.97, 22.13, 15.47.
HRMS: exact mass calculated for $[M+H]^+$ ($C_{18}H_{24}NO_4$) requires *m/z* 318.1700, found *m/z* 318.1696.

Compound S91.

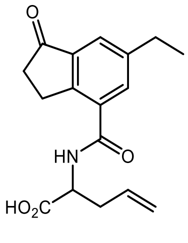

S91

^{1}H NMR (400 MHz, $CDCl_3$): δ 7.75 – 7.68 (m, 2H), 7.18 (br. s, 1H), 6.72 (d, *J* = 7.3 Hz, 1H), 5.80 (ddt, *J* = 17.3, 10.1, 7.2 Hz, 1H), 5.23 – 5.13 (m, 2H), 4.87 (dt, *J* = 7.2, 5.6 Hz, 1H), 3.38 – 3.31 (m, 2H), 2.85 – 2.69 (m, 6H), 1.25 (t, *J* = 7.6 Hz, 3H).

^{13}**C NMR** (101 MHz, $CDCl_3$): δ 206.9, 174.0, 166.9, 151.5, 144.5, 138.6, 133.4, 132.7, 132.5, 125.5, 119.6, 52.2, 36.6, 36.4, 28.5, 25.9, 15.5.

HRMS: exact mass calculated for $[M+H]^+$ ($C_{17}H_{20}NO_4$) requires *m/z* 302.1387, found *m/z* 302.1383.

Compound S92.

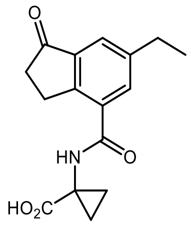

S92

^{1}**H NMR** (400 MHz, $CDCl_3$): δ 7.65 (s, 2H), 7.05 (s, 1H), 6.71 (br. s, 1H), 3.36 – 3.30 (m, 2H), 2.73 – 2.63 (m, 4H), 1.70 – 1.64 (m, 2H), 1.34 – 1.27 (m, 2H), 1.22 (t, *J* = 7.6 Hz, 3H).

^{13}**C NMR** (101 MHz, $CDCl_3$): δ 207.1, 176.0, 168.7, 152.3, 144.2, 138.5, 133.0, 132.7, 125.3, 36.6, 33.9, 28.4, 25.8, 17.8, 15.5. One signal not observed.

HRMS: exact mass calculated for $[M+H]^+$ ($C_{16}H_{18}NO_4$) requires *m/z* 288.1230, found *m/z* 288.1228.

Compound S93.

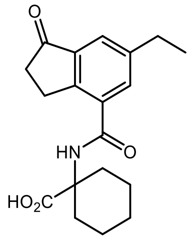

S93

^{1}**H NMR** (400 MHz, $CDCl_3$): δ 7.68 (s, 2H), 6.30 (s, 1H), 3.36 – 3.28 (m, 2H), 2.78 – 2.66 (m, 4H), 2.27 – 2.16 (m, 2H), 2.01 – 1.92 (m, 2H), 1.81 – 1.64 (m, 3H), 1.57 – 1.44 (m, 2H), 1.43 – 1.34 (m, 1H), 1.26 (t, *J* = 7.6 Hz, 3H). CO_2H not observed.

^{13}**C NMR** (101 MHz, $CDCl_3$): δ 206.7, 176.8, 167.7, 151.4, 144.4, 138.5, 133.3, 133.2, 125.3, 59.7, 36.6, 32.3, 28.5, 25.9, 25.3, 21.8, 15.5. Two signals equivalent.

HRMS: exact mass calculated for $[M+H]^+$ ($C_{19}H_{24}NO_4$) requires *m/z* 330.1700, found *m/z* 300.1696.

Compound S94.

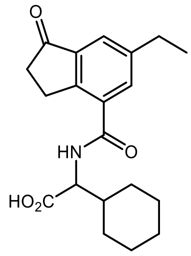

S94

^{1}H NMR (400 MHz, $CDCl_3$): δ 7.77 – 7.68 (m, 2H), 6.66 (d, J = 8.4 Hz, 1H), 4.77 (dd, J = 8.4, 4.7 Hz, 1H), 3.40 – 3.33 (m, 2H), 2.78 – 2.70 (m, 4H), 2.05 – 1.93 (m, 1H), 1.89 – 1.61 (m, 5H), 1.36 – 1.05 (m, 8H). CO_2H not observed.
^{13}C NMR (101 MHz, $CDCl_3$): δ 207.0, 174.3, 167.2, 151.5, 144.5, 138.6, 133.5, 132.9, 125.5, 57.3, 41.2, 36.6, 29.8, 28.5, 28.4, 26.2, 26.2, 26.1, 26.0, 15.5.
HRMS: exact mass calculated for $[M+H]^+$ ($C_{20}H_{26}NO_4$) requires *m/z* 344.1856, found *m/z* 344.1853.

Compound S95.

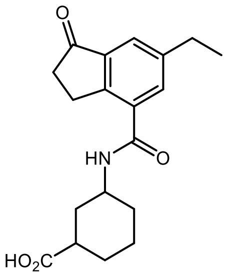

S95

^{1}H NMR (400 MHz, $CDCl_3$): δ 7.68 (s, 1H), 7.62 (d, J = 1.5 Hz, 1H), 6.21 (d, J = 7.9 Hz, 1H), 4.12 – 4.00 (m, 1H), 3.38 – 3.31 (m, 2H), 2.78 – 2.67 (m, 4H), 2.59 – 2.50 (m, 1H), 2.41 – 2.31 (m, 1H), 2.14 – 1.99 (m, 2H), 1.95 – 1.87 (m, 1H), 1.56 – 1.36 (m, 3H), 1.31 – 1.19 (m, 4H). CO_2H not observed.
^{13}C NMR (101 MHz, $CDCl_3$): δ 206.8, 178.7, 166.6, 151.5, 144.2, 138.4, 133.4, 132.7, 124.9, 47.9, 41.7, 36.5, 34.7, 32.4, 28.4, 28.2, 25.7, 23.8, 15.4.
HRMS: exact mass calculated for $[M+H]^+$ ($C_{19}H_{24}NO_4$) requires *m/z* 330.1700, found *m/z* 330.1696.

Compound S96.

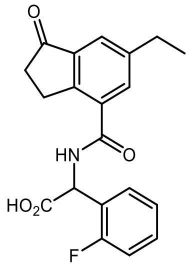

S96

^{1}H NMR (400 MHz, $CDCl_3$): δ 7.76 (d, J = 1.6 Hz, 1H), 7.71 (s, 1H), 7.51 (td, J = 7.5, 1.7 Hz, 1H), 7.36 – 7.27 (m, 2H), 7.17 (td, J = 7.5, 1.1 Hz, 1H), 7.12 – 7.06 (m, 1H), 5.93 (d, J = 7.0 Hz, 1H), 3.46 – 3.29 (m, 2H), 2.78 – 2.68 (m, 4H), 1.25 (t, J = 7.6 Hz, 3H). CO_2H not observed.
^{13}C NMR (101 MHz, $CDCl_3$): δ 206.9, 172.1, 166.4, 160.9 (d, $^1J_{C\text{-}F}$ = 247.6 Hz), 151.7, 144.5, 138.6, 133.7, 132.3, 130.6 (d, $^3J_{C\text{-}F}$ = 3.7 Hz), 130.5 (d, $^3J_{C\text{-}F}$ = 8.4 Hz), 125.7, 124.9 – 124.5 (m), 116.1 (d, $^2J_{C\text{-}F}$ = 21.2 Hz), 52.4, 36.6, 28.5, 26.0, 15.5.
HRMS: exact mass calculated for $[M+H]^+$ ($C_{20}H_{19}FNO_4$) requires *m/z* 356.1293, found *m/z* 356.1291.

Compound S97.

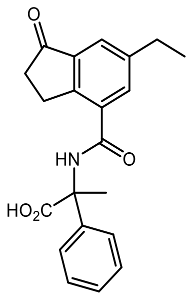

S97

^{1}H NMR (400 MHz, $CDCl_3$): δ 7.78 (d, J = 1.6 Hz, 1H), 7.71 (s, 1H), 7.66 (s, 1H), 7.55 – 7.50 (m, 2H), 7.40 – 7.34 (m, 2H), 7.33 – 7.28 (m, 1H), 3.44 – 3.28 (m, 2H), 2.79 – 2.67 (m, 4H), 2.19 (s, 3H), 1.27 (t, J = 7.6 Hz, 3H). CO_2H not observed.

^{13}C NMR (101 MHz, $CDCl_3$): δ 206.9, 175.3, 166.2, 151.5, 144.5, 140.0, 138.6, 133.5, 133.1, 128.8, 128.1, 125.9, 125.5, 62.8, 36.6, 28.5, 26.1, 22.7, 15.5. Two peaks equivalent.

HRMS: exact mass calculated for $[M+H]^+$ ($C_{21}H_{22}NO_4$) requires *m/z* 352.1543, found *m/z* 352.1543.

Compound S98.

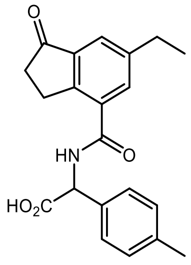

S98

^{1}H NMR (400 MHz, $CDCl_3$): δ 7.74 (d, J = 1.4 Hz, 1H), 7.71 (s, 1H), 7.37 (d, J = 8.1 Hz, 2H), 7.19 (d, J = 7.9 Hz, 2H), 7.14 (d, J = 6.5 Hz, 1H), 5.71 (d, J = 6.7 Hz, 1H), 3.38 – 3.32 (m, 2H), 2.77 – 2.68 (m, 4H), 2.34 (s, 3H), 1.25 (t, J = 7.6 Hz, 3H). CO_2H not observed.

^{13}C NMR (101 MHz, $CDCl_3$): δ 207.0, 166.5, 151.8, 144.5, 138.8, 138.6, 133.5, 132.3, 129.9, 127.4, 125.7, 56.8, 36.6, 28.5, 26.0, 21.3, 15.5. Four signals not observed.

HRMS: exact mass calculated for $[M+H]^+$ ($C_{21}H_{22}NO_4$) requires *m/z* 352.1543, found *m/z* 352.1542.

Compound S99.

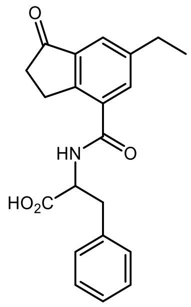

S99

^{1}H NMR (400 MHz, $CDCl_3$): δ 7.68 (s, 1H), 7.60 (d, J = 1.4 Hz, 1H), 7.32 – 7.19 (m, 5H), 6.60 (d, J = 7.2 Hz, 1H), 5.10 – 5.03 (m, 1H), 3.47 – 3.09 (m, 4H), 2.76 – 2.63 (m, 4H), 1.24 (t, J = 7.6 Hz, 3H). CO_2H not observed.

^{13}C NMR (101 MHz, $CDCl_3$): δ 206.9, 173.6, 166.8, 151.6, 144.4, 138.5, 136.2, 133.4, 132.6, 129.7, 128.7, 127.3, 125.5, 53.6, 37.4, 36.6, 28.4, 25.7, 15.4. Two signals equivalent.

HRMS: exact mass calculated for $[M+H]^+$ ($C_{21}H_{22}NO_4$) requires *m/z* 352.1543, found *m/z* 352.1542.

Compound S100.

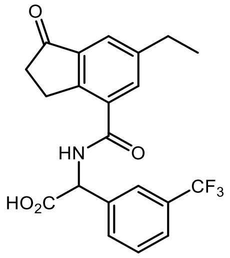

S100

^{1}H NMR (400 MHz, $CDCl_3$): δ 7.83 – 7.67 (m, 4H), 7.61 – 7.56 (m, 1H), 7.54 – 7.40 (m, 2H), 5.77 (d, J = 6.3 Hz, 1H), 3.40 – 3.32 (m, 2H), 2.79 – 2.69 (m, 4H), 1.26 (t, J = 7.6 Hz, 3H). CO_2H not observed.

^{13}C NMR (101 MHz, MeOD): δ 209.0, 172.8, 169.6, 153.5, 145.7, 139.7, 139.2, 135.0, 134.3, 132.9, 132.0 (app. d, $^2J_{C\text{-}F}$ = 32.4 Hz), 130.7, 126.1 (q, $^3J_{C\text{-}F}$ = 3.8 Hz), 125.8 (app. d, $^3J_{C\text{-}F}$ = 4.1 Hz), 125.7, 58.0, 37.3, 29.3, 26.4, 15.8. F bearing carbon not observed.

HRMS: exact mass calculated for $[M+H]^+$ ($C_{21}H_{19}F_3NO_4$) requires *m/z* 406.1261, found *m/z* 406.1256.

Compound S101.

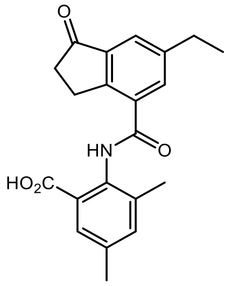

S101

^{1}H NMR (400 MHz, $CDCl_3$): δ 9.86 (s, 1H), 7.96 (s, 1H), 7.75 (s, 1H), 7.73 (d, J = 1.5 Hz, 1H), 7.33 (s, 1H), 3.48 – 3.41 (m, 2H), 2.83 – 2.69 (m, 4H), 2.35 (s, 3H), 2.33 (s, 3H), 1.30 (t, J = 7.6 Hz, 3H). One signal not observed.

^{13}C NMR (101 MHz, $CDCl_3$): δ 207.3, 170.6, 152.7, 144.5, 138.7, 137.2, 135.6, 135.6, 135.1, 133.8, 133.1, 129.7, 125.7, 122.6, 36.7, 28.6, 26.2, 20.9, 19.6, 15.5. One signal not observed.

HRMS: exact mass calculated for $[M+H]^+$ ($C_{21}H_{22}NO_4$) requires *m/z* 352.1543, found *m/z* 352.1542.

Compound S102.

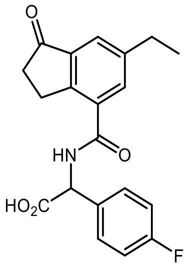

S102

^{1}H NMR (400 MHz, $CDCl_3$): δ 7.75 (d, J = 1.4 Hz, 1H), 7.73 (s, 1H), 7.50 – 7.44 (m, 2H), 7.19 (d, J = 6.4 Hz, 1H), 7.11 – 7.04 (m, 2H), 5.73 (d, J = 6.4 Hz, 1H), 3.40 – 3.33 (m, 2H), 2.78 – 2.69 (m, 4H), 1.27 (t, J = 7.6 Hz, 3H). CO_2H not observed.

^{13}C NMR (101 MHz, $CDCl_3$): δ 166.4, 151.8, 144.6, 138.7, 133.4, 129.3 (d, $^{3}J_{C\text{-}F}$ = 8.4 Hz), 125.8, 116.2 (d, $^{2}J_{C\text{-}F}$ = 21.8 Hz), 36.6, 28.5, 26.1, 15.6. Two signals equivalent, F bearing carbon not observed. Five signals not observed.

HRMS: exact mass calculated for $[M+H]^+$ ($C_{20}H_{19}FNO_4$) requires m/z 356.1293, found m/z 356.1290.

Compound S103.

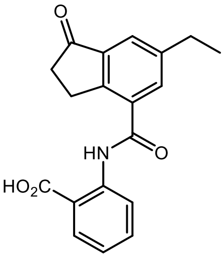

S103

^{1}H NMR (400 MHz, $CDCl_3$): δ 8.74 (dd, J = 8.5, 0.8 Hz, 1H), 8.08 (dd, J = 8.0, 1.6 Hz, 1H), 7.86 (d, J = 1.5 Hz, 1H), 7.67 (s, 1H), 7.54 (ddd, J = 8.7, 7.4, 1.7 Hz, 1H), 7.13 – 7.06 (m, 1H), 3.46 – 3.38 (m, 2H), 2.77 – 2.63 (m, 4H), 1.23 (t, J = 7.6 Hz, 3H). NH and CO_2H not observed.

^{13}C NMR (101 MHz, $CDCl_3$): δ 207.8, 170.6, 165.8, 152.8, 144.5, 141.3, 138.4, 134.5, 133.6, 133.2, 131.7, 125.5, 123.0, 120.1, 115.9, 36.5, 28.3, 25.8, 15.2.

HRMS: exact mass calculated for $[M+H]^+$ ($C_{19}H_{18}NO_4$) requires m/z 324.1230, found m/z 324.1228.

Compound S104.

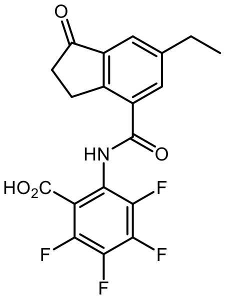

S104

^{1}H NMR (400 MHz, $CDCl_3$): δ 9.61 (s, 1H), 7.90 (d, J = 1.4 Hz, 1H), 7.78 (s, 1H), 3.47 – 3.39 (m, 2H), 2.81 – 2.71 (m, 4H), 1.28 (t, J = 7.6 Hz, 3H). One signal not observed.

^{13}C NMR (101 MHz, $CDCl_3$): δ 206.9, 165.3, 164.7, 153.1, 144.7, 138.9, 133.9, 131.3, 126.5, 36.6, 28.5, 26.2, 15.5. Six signals not observed.

HRMS: exact mass calculated for $[M+H]^+$ ($C_{19}H_{14}F_4NO_4$) requires *m/z* 396.0853, found *m/z* 396.0853.

Compound S105.

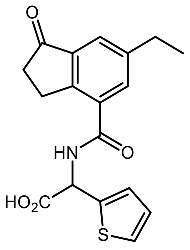

S105

^{1}H NMR (400 MHz, $CDCl_3$): δ 7.76 (d, J = 1.4 Hz, 1H), 7.72 (s, 1H), 7.27 (d, J = 5.1 Hz, 1H), 7.21 – 7.15 (m, 2H), 6.99 (dd, J = 4.9, 3.7 Hz, 1H), 6.05 (d, J = 7.1 Hz, 1H), 3.40 – 3.35 (m, 2H), 2.78 – 2.68 (m, 4H), 1.26 (t, J = 7.6 Hz, 3H). CO_2H not observed.

^{13}C NMR (101 MHz, $CDCl_3$): δ 206.9, 171.8, 166.5, 151.8, 144.5, 139.1, 138.7, 133.5, 132.2, 127.3, 126.7, 125.9, 125.8, 52.5, 36.6, 28.5, 26.0, 15.5.

HRMS: exact mass calculated for $[M+H]^+$ ($C_{18}H_{18}SNO_4$) requires *m/z* 344.0951, found *m/z* 344.0949.

Compound S106.

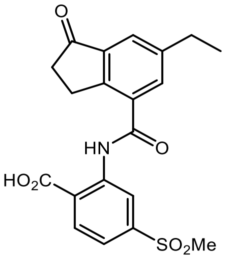

S106

^{1}H NMR (400 MHz, DMSO-d_6): δ 12.14 (s, 1H), 9.17 (d, J = 1.8 Hz, 1H), 8.26 (d, J = 8.3 Hz, 1H), 8.03 (d, J = 1.4 Hz, 1H), 7.76 (dd, J = 8.3, 1.9 Hz, 1H), 7.72 (s, 1H), 3.56 – 3.31 (br. m, 3H), 2.78 (q, J = 7.6 Hz, 2H), 2.71 – 2.65 (m, 2H), 2.54 (s, 3H), 1.26 (t, J = 7.6 Hz, 3H).

^{13}C NMR (101 MHz, DMSO-d_6): δ 205.9, 168.9, 165.1, 152.7, 145.0, 144.1, 140.9, 138.4, 132.7, 132.6, 132.4, 125.2, 121.7, 121.3, 118.3, 43.3, 36.1, 27.7, 25.6, 15.6.

HRMS: exact mass calculated for $[M+H]^+$ ($C_{20}H_{20}SNO_6$) requires *m/z* 402.1006, found *m/z* 402.1004.

Compound S107.

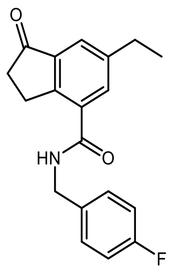

S107

^{1}H NMR (400 MHz, $CDCl_3$): δ 7.68 (s, 1H), 7.64 (d, J = 1.5 Hz, 1H), 7.38 – 7.30 (m, 2H), 7.08 – 6.99 (m, 2H), 6.42 (br. s, 1H), 4.62 (d, J = 5.8 Hz, 2H), 3.39 – 3.31 (m, 2H), 2.77 – 2.65 (m, 4H), 1.25 (t, J = 7.6 Hz, 3H).

^{13}C NMR (101 MHz, $CDCl_3$): δ 206.8, 167.2, 162.4 (d, $^1J_{C\text{-}F}$ = 246.3 Hz), 151.9, 144.4, 138.7, 133.9 (d, $J_{C\text{-}F}$ = 3.3 Hz), 133.0, 132.7, 129.8 (d, $^3J_{C\text{-}F}$ = 8.1 Hz), 125.3, 115.9 (d, $^2J_{C\text{-}F}$ = 21.5 Hz), 43.4, 36.6, 28.5, 25.9, 15.6. Two signals equivalent.

HRMS: exact mass calculated for $[M+H]^+$ ($C_{19}H_{19}FNO_2$) requires m/z 312.1394, found m/z 312.1391.

Compound S108.

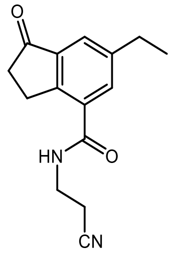

S108

^{1}H NMR (400 MHz, $CDCl_3$): δ 7.67 (s, 2H), 3.71 – 3.64 (m, 2H), 3.37 – 3.28 (m, 2H), 2.80 – 2.64 (m, 6H), 1.24 (t, J = 7.6 Hz, 3H). NH not observed.

^{13}C NMR (101 MHz, $CDCl_3$): δ 207.1, 168.0, 152.1, 144.4, 138.6, 133.1, 132.3, 125.5, 118.5, 36.6, 36.0, 28.4, 25.8, 18.5, 15.4.

HRMS: exact mass calculated for $[M+H]^+$ ($C_{15}H_{17}N_2O_2$) requires m/z 257.1285, found m/z 257.1283.

Compound S109.

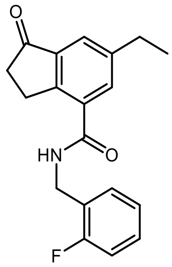

S109

^{1}H NMR (400 MHz, $CDCl_3$): δ 7.68 (s, 1H), 7.66 (d, J = 1.5 Hz, 1H), 7.44 (td, J = 7.6, 1.6 Hz, 1H), 7.34 – 7.27 (m, 1H), 7.15 (td, J = 7.5, 1.0 Hz, 1H), 7.12 – 7.04 (m, 1H), 6.53 (br. s, 1H), 4.70 (d, J = 5.9 Hz, 2H), 3.39 – 3.30 (m, 2H), 2.77 – 2.65 (m, 4H), 1.25 (t, J = 7.6 Hz, 3H).

^{13}**C NMR** (101 MHz, $CDCl_3$): δ 206.8, 167.2, 161.3 (d, $^1J_{c\text{-}f}$= 246.0 Hz), 151.8, 144.4, 138.7, 133.0, 132.9, 130.7 (d, $^3J_{c\text{-}f}$= 4.2 Hz), 129.8 (d, $^3J_{c\text{-}f}$= 8.2 Hz), 125.3, 125.1 (d, $^2J_{c\text{-}f}$= 14.7 Hz), 124.6 (d, $J_{c\text{-}f}$= 3.6 Hz), 115.7 (d, $^2J_{c\text{-}f}$= 21.2 Hz), 38.3 (d, $^3J_{c\text{-}f}$= 3.6 Hz), 36.6, 28.5, 25.9, 15.5.

HRMS: exact mass calculated for $[M+H]^+$ ($C_{19}H_{19}FNO_2$) requires *m/z* 312.1394, found *m/z* 312.1391.

Compound S110.

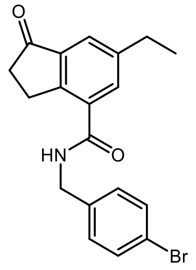

S110

^{1}**H NMR** (400 MHz, $CDCl_3$): δ 7.67 (s, 1H), 7.64 (d, J = 1.4 Hz, 1H), 7.49 – 7.45 (m, 2H), 7.23 (d, J = 8.4 Hz, 2H), 6.55 (br. s, 1H), 4.59 (d, J = 5.9 Hz, 2H), 3.38 – 3.29 (m, 2H), 2.74 – 2.65 (m, 4H), 1.24 (t, J = 7.6 Hz, 3H).

^{13}**C NMR** (101 MHz, $CDCl_3$): δ 206.8, 167.3, 151.9, 144.4, 138.7, 137.2, 132.9, 132.0, 129.7, 125.3, 121.7, 43.5, 36.6, 28.5, 25.9, 15.5. Three signals not observed.

HRMS: exact mass calculated for $[M+H]^+$ ($C_{19}H_{19}BrNO_2$) requires *m/z* 372.0594, found *m/z* 372.0599.

Compound S111.

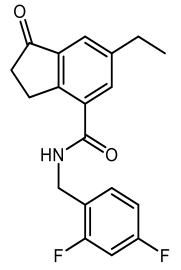

S111

^{1}**H NMR** (400 MHz, $CDCl_3$): δ 7.69 (s, 1H), 7.64 (d, J = 1.4 Hz, 1H), 7.48 – 7.39 (m, 1H), 6.92 – 6.81 (m, 2H), 6.44 (br. s, 1H), 4.66 (d, J = 5.9 Hz, 2H), 3.38 – 3.29 (m, 2H), 2.77 – 2.66 (m, 4H), 1.25 (t, J = 7.6 Hz, 3H).

^{13}**C NMR** (101 MHz, $CDCl_3$): δ 206.7, 167.2, 151.8, 144.4, 138.7, 132.8, 132.8, 131.7 – 131.5 (m), 125.4, 121.2 (dd, $^2J_{C\text{-}F}$ = 15.0 Hz, $J_{C\text{-}F}$ = 4.0 Hz), 111.7 (dd, $^2J_{C\text{-}F}$ = 21.1 Hz, $J_{C\text{-}F}$ = 3.7 Hz), 104.2 (t, $^2J_{C\text{-}F}$ = 25.4 Hz), 37.8 (d, $^3J_{C\text{-}F}$ = 3.1 Hz), 36.6, 28.5, 25.9, 15.6. F bearing carbons not observed.

HRMS: exact mass calculated for $[M+H]^+$ ($C_{19}H_{18}F_2NO_2$) requires *m/z* 330.1300, found *m/z* 330.1296.

CMA Core Analogues.

Reactions carried out according to General Procedure I.

Compound S112.

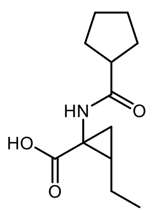

S112

^{1}H NMR (500 MHz, MeOD): δ 2.64 – 2.56 (m, 1H), 1.90 – 1.67 (m, 6H), 1.67 – 1.53 (m, 4H), 1.47 – 1.39 (m, 2H), 1.14 – 1.06 (m, 1H), 1.00 (t, J = 7.4 Hz, 3H). N*H* and CO_2*H* not observed.

^{13}C NMR (126 MHz, MeOD): δ 180.2, 174.9, 46.0, 39.0, 33.2, 31.2, 31.1, 27.1, 27.0, 23.3, 21.6, 13.8.

HRMS: exact mass calculated for $[M+H]^+$ ($C_{12}H_{20}NO_3$) requires *m/z* 226.1443, found *m/z* 226.1441.

Compound S113.

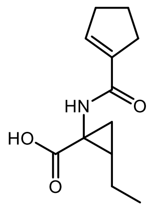

S113

^{1}H NMR (500 MHz, MeOD): δ 6.58 – 6.56 (m, 1H), 2.58 – 2.52 (m, 2H), 2.52 – 2.46 (m, 2H), 2.00 – 1.92 (m, 2H), 1.67 – 1.59 (m, 2H), 1.55 – 1.43 (m, 2H), 1.17 – 1.12 (m, 1H), 1.02 (t, J = 7.4 Hz, 3H). N*H* and CO_2*H* not observed.

^{13}C NMR (126 MHz, MeOD): δ 175.0, 169.2, 140.5, 139.8, 39.1, 34.1, 33.2, 32.4, 24.3, 23.3, 21.7, 13.7.

HRMS: exact mass calculated for $[M+H]^+$ ($C_{12}H_{18}NO_3$) requires *m/z* 224.1282, found *m/z* 224.1287.

Compound S114.

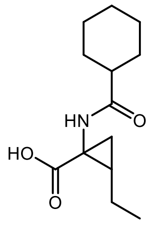

S114

^{1}H NMR (500 MHz, MeOD): δ 2.18 – 2.10 (m, 1H), 1.94 – 1.53 (m, 8H), 1.48 – 1.36 (m, 2H), 1.35 – 1.18 (m, 4H), 1.12 – 1.05 (m, 1H), 1.00 (t, J = 7.4 Hz, 3H). N*H* and CO_2*H* not observed.

^{13}C NMR (126 MHz, MeOD): δ 180.2, 174.9, 46.0, 44.3, 38.8, 33.2, 30.4, 26.9, 26.8, 26.8, 23.3, 21.6, 13.7.

HRMS: exact mass calculated for $[M+H]^+$ ($C_{13}H_{22}NO_3$) requires *m/z* 240.1600, found *m/z* 240.1595.

Compound S115.

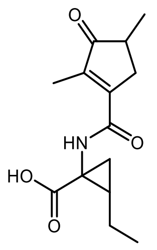

S115

^{1}H NMR (500 MHz, MeOD): δ 3.05 – 2.97 (m, 1H), 2.51 – 2.44 (m, 1H), 2.39 – 2.31 (m, 1H), 1.87 (t, J = 2.2 Hz, 3H), 1.72 – 1.61 (m, 2H), 1.60 – 1.49 (m, 2H), 1.26 – 1.22 (m, 1H), 1.17 (d, J = 7.5 Hz, 3H), 1.03 (t, J = 7.4 Hz, 3H). N*H* and CO_2H not observed.

^{13}C NMR (126 MHz, MeOD): δ 214.3, 174.2, 169.9, 160.9, 141.0, 40.7, 38.8, 36.8, 33.4, 23.4, 21.6, 16.3, 13.7, 9.3.

HRMS: exact mass calculated for $[M+H]^+$ ($C_{14}H_{20}NO_4$) requires *m/z* 266.1392, found *m/z* 266.1389.

Compound S116.

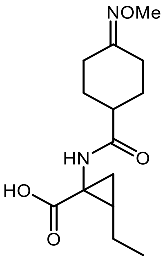

S116

^{1}H NMR (500 MHz, MeOD): δ 3.77 (s, 3H), 3.22 – 3.14 (m, 1H), 2.46 – 2.36 (m, 2H), 2.19 – 2.09 (m, 1H), 2.06 – 1.84 (m, 3H), 1.69 – 1.51 (m, 4H), 1.47 – 1.39 (m, 2H), 1.14 – 1.07 (m, 1H), 1.00 (t, J = 7.4 Hz, 3H). 1:1 mixture of oxime isomers. N*H* and CO_2H not observed.

^{13}C NMR (126 MHz, MeOD): δ 178.6, 174.8, 160.2, 61.3, 44.4, 38.9, 33.2, 31.4, 31.3, 30.5, 30.4, 29.2, 29.1, 24.5, 24.4, 23.3, 21.6, 13.8. Oxime isomer peaks observed.

HRMS: exact mass calculated for $[M+H]^+$ ($C_{14}H_{23}N_4O_2$) requires *m/z* 283.1658, found *m/z* 283.1653.

Compound S117.

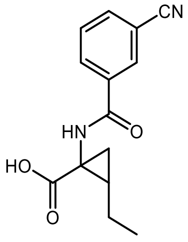

S117

^{1}H NMR (500 MHz, MeOD): δ 8.18 (t, J = 1.4 Hz, 1H), 8.14 – 8.10 (m, 1H), 7.91 – 7.88 (m, 1H), 7.66 (t, J = 7.8 Hz, 1H), 1.73 – 1.59 (m, 3H), 1.57 – 1.53 (m, 1H), 1.31 – 1.25 (m, 1H), 1.06 (t, J = 7.3 Hz, 3H). N*H* and CO_2H not observed.

^{13}C NMR (126 MHz, MeOD): δ 174.6, 168.8, 136.8, 136.0, 133.0, 132.2, 130.8, 119.1, 113.8, 39.4, 33.4, 23.4, 21.6, 13.8.

HRMS: exact mass calculated for $[M+H]^+$ ($C_{14}H_{15}N_2O_3$) requires *m/z* 259.1083, found *m/z* 259.1077.

Compound S118.

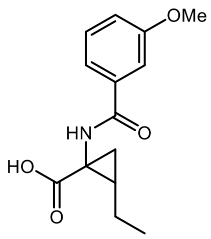

S118

^{1}H NMR (500 MHz, MeOD): δ 7.42 – 7.38 (m, 2H), 7.37 – 7.32 (m, 1H), 7.11 – 7.06 (m, 1H), 3.84 (s, 3H), 1.74 – 1.64 (m, 2H), 1.64 – 1.57 (m, 1H), 1.55 – 1.51 (m, 1H), 1.26 – 1.22 (m, 1H), 1.06 (t, J = 7.3 Hz, 3H). N*H* and CO_2*H* not observed.
^{13}C NMR (126 MHz, MeOD): δ 175.0, 171.0, 161.2, 136.9, 130.5, 120.6, 118.7, 113.6, 55.9, 39.6, 33.2, 23.3, 21.7, 13.8.
HRMS: exact mass calculated for $[M+H]^+$ ($C_{14}H_{18}NO_4$) requires *m/z* 264.1236, found *m/z* 264.1232.

Compound S119.

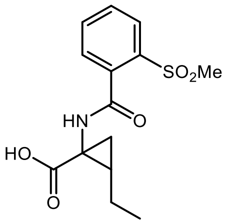

S119

^{1}H NMR (500 MHz, MeOD): δ 8.05 – 8.01 (m, 1H), 7.80 – 7.75 (m, 1H), 7.71 – 7.66 (m, 2H), 3.29 (s, 3H), 1.72 – 1.59 (m, 3H), 1.54 – 1.42 (m, 2H), 1.03 (t, J = 7.1 Hz, 3H). N*H* and CO_2*H* not observed.
^{13}C NMR (126 MHz, MeOD): δ 174.7, 171.1, 139.1, 138.6, 135.0, 131.3, 130.5, 129.9, 45.6, 39.0, 33.5, 23.1, 21.6, 13.8.
HRMS: exact mass calculated for $[M+H]^+$ ($C_{14}H_{18}NO_5S$) requires *m/z* 312.0906, found *m/z* 312.0901.

Compound S120.

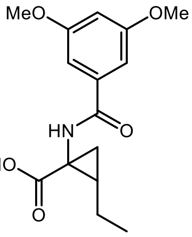

S120

^{1}H NMR (500 MHz, MeOD): δ 7.00 (d, J = 2.3 Hz, 2H), 6.63 (t, J = 2.3 Hz, 1H), 3.81 (s, 6H), 1.71 – 1.58 (m, 3H), 1.55 – 1.51 (m, 1H), 1.26 – 1.22 (m, 1H), 1.06 (t, J = 7.3 Hz, 3H). N*H* and CO_2*H* not observed.
^{13}C NMR (126 MHz, MeOD): δ 174.9, 170.9, 162.3, 137.4, 106.3, 104.8, 56.0, 39.5, 33.3, 23.4, 21.7, 13.8. Three signals not observed.
HRMS: exact mass calculated for $[M+H]^+$ ($C_{15}H_{20}NO_5$) requires *m/z* 294.1341, found *m/z* 294.1337.

Compound S121.

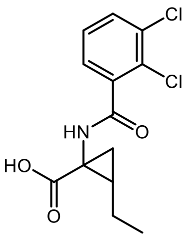

S121

^{1}H NMR (500 MHz, MeOD): δ 7.60 (dd, J = 7.9, 1.6 Hz, 1H), 7.42 (dd, J = 7.6, 1.6 Hz, 1H), 7.36 (t, J = 7.8 Hz, 1H), 1.72 – 1.52 (m, 4H), 1.35 – 1.28 (m, 1H), 1.03 (t, J = 7.3 Hz, 3H). N*H* and CO_2H not observed.

^{13}C NMR (126 MHz, MeOD): δ 174.5, 170.0, 139.8, 134.4, 132.6, 130.4, 129.1, 128.2, 39.1, 33.4, 23.3, 21.6, 13.7.

HRMS: exact mass calculated for $[M+H]^+$ ($C_{13}H_{14}NO_3Cl_2$) requires *m/z* 302.0351, found *m/z* 302.0347.

Compound S122.

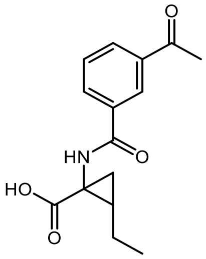

S122

^{1}H NMR (500 MHz, MeOD): δ 8.46 (t, J = 1.6 Hz, 1H), 8.16 – 8.13 (m, 1H), 8.08 – 8.05 (m, 1H), 7.60 (t, J = 7.8 Hz, 1H), 2.65 (s, 3H), 1.73 – 1.66 (m, 2H), 1.66 – 1.58 (m, 1H), 1.58 – 1.53 (m, 1H), 1.29 – 1.24 (m, 1H), 1.07 (t, J = 7.3 Hz, 3H). N*H* and CO_2H not observed.

^{13}C NMR (126 MHz, MeOD): δ 199.6, 175.1, 170.1, 138.5, 136.2, 133.1, 132.2, 130.0, 128.5, 39.8, 33.1, 26.8, 23.2, 21.7, 13.8.

HRMS: exact mass calculated for $[M+H]^+$ ($C_{15}H_{18}NO_4$) requires *m/z* 276.1236, found *m/z* 276.1234.

Compound S123.

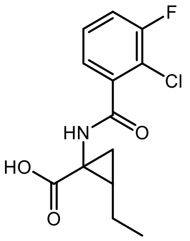

S123

^{1}H NMR (500 MHz, MeOD): δ 7.42 – 7.37 (m, 1H), 7.37 – 7.29 (m, 2H), 1.74 – 1.52 (m, 4H), 1.34 – 1.29 (m, 1H), 1.03 (t, J = 7.3 Hz, 3H). N*H* and CO_2H not observed.

^{13}C NMR (126 MHz, MeOD): δ 174.4, 169.5 (d, J_{C-F} = 2.7 Hz), 159.5 (d, $^1J_{C-F}$ = 248.5 Hz), 139.6, 129.6 (d, $^3J_{C-F}$ = 7.8 Hz), 125.3 (d, $^3J_{C-F}$ = 3.7 Hz), 119.5 (d, $^2J_{C-F}$ = 19.1 Hz), 118.7 (d, $^2J_{C-F}$ = 21.7 Hz), 39.1, 33.5, 23.3, 21.6, 13.7.

HRMS: exact mass calculated for $[M+H]^+$ ($C_{13}H_{14}NO_3ClF$) requires *m/z* 286.0646, found *m/z* 286.0645.

Compound S124.

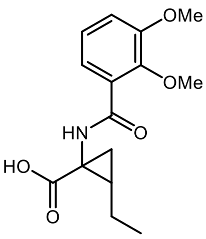

S124

^{1}H NMR (500 MHz, MeOD): δ 7.30 (dd, J = 7.4, 2.1 Hz, 1H), 7.19 – 7.11 (m, 2H), 3.88 (s, 3H), 3.87 (s, 3H), 1.71 – 1.63 (m, 2H), 1.62 – 1.52 (m, 2H), 1.32 – 1.28 (m, 1H), 1.05 (t, J = 7.4 Hz, 3H). NH and CO_2H not observed.

^{13}C NMR (126 MHz, MeOD): δ 174.6, 169.6, 154.3, 148.8, 129.4, 125.5, 122.3, 116.6, 62.0, 56.6, 39.3, 33.7, 23.5, 21.6, 13.7.

HRMS: exact mass calculated for $[M+H]^+$ ($C_{15}H_{20}NO_5$) requires m/z 294.1341, found m/z 294.1339.

Compound S125.

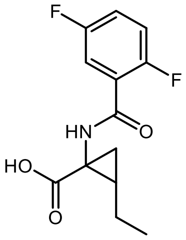

S125

^{1}H NMR (500 MHz, MeOD): δ 7.43 – 7.38 (m, 1H), 7.30 – 7.19 (m, 2H), 1.71 – 1.56 (m, 3H), 1.55 – 1.51 (m, 1H), 1.31 – 1.26 (m, 1H), 1.04 (t, J = 7.3 Hz, 3H). NH and CO_2H not observed.

^{13}C NMR (126 MHz, MeOD): δ 174.4, 166.6, 159.9 (dd, $^{1}J_{C\text{-}F}$ = 242.6 Hz, $J_{C\text{-}F}$ = 2.0 Hz), 157.4 (dd, $^{1}J_{C\text{-}F}$ = 246.3 Hz, $J_{C\text{-}F}$ = 2.2 Hz), 125.8 (dd, $^{2}J_{C\text{-}F}$ = 16.9 Hz, $^{3}J_{C\text{-}F}$ = 7.3 Hz), 120.4 (dd, $^{2}J_{C\text{-}F}$ = 24.5 Hz, $^{3}J_{C\text{-}F}$ = 9.1 Hz), 119.0 (dd, $^{2}J_{C\text{-}F}$ = 26.1 Hz, $^{3}J_{C\text{-}F}$ = 8.4 Hz), 117.5 (dd, $^{2}J_{C\text{-}F}$ = 25.9 Hz, $^{3}J_{C\text{-}F}$ = 3.1 Hz), 39.4, 33.6, 23.5, 21.6, 13.7.

HRMS: exact mass calculated for $[M+H]^+$ ($C_{13}H_{14}NO_3F_2$) requires m/z 270.0942, found m/z 270.0938.

Compound S126.

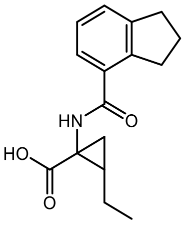

S126

^{1}H NMR (500 MHz, MeOD): δ 7.35 – 7.30 (m, 2H), 7.17 (t, J = 7.6 Hz, 1H), 3.10 (t, J = 7.4 Hz, 2H), 2.92 (t, J = 7.4 Hz, 2H), 2.06 (p, J = 7.4 Hz, 2H), 1.71 – 1.63 (m, 2H), 1.63 – 1.55 (m, 1H), 1.55 – 1.51 (m, 1H), 1.28 – 1.24 (m, 1H), 1.04 (t, J = 7.3 Hz, 3H). NH and CO_2H not observed.

^{13}C NMR (126 MHz, MeOD): δ 174.8, 173.1, 146.7, 144.3, 133.1, 127.7, 127.3, 125.9, 39.3, 33.6, 33.3, 33.3, 26.4, 23.5, 21.6, 13.8.

HRMS: exact mass calculated for $[M+H]^+$ ($C_{16}H_{20}NO_3$) requires m/z 274.1443, found m/z 274.1437.

Compound S127.

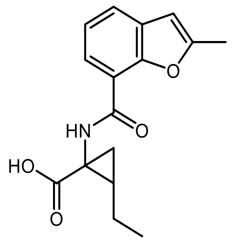

S127

^{1}H NMR (500 MHz, MeOD): δ 7.72 (dd, J = 7.6, 1.0 Hz, 1H), 7.65 (dd, J = 7.7, 1.0 Hz, 1H), 7.26 (t, J = 7.7 Hz, 1H), 6.56 (d, J = 1.0 Hz, 1H), 2.52 (app. d, J = 0.8 Hz, 3H), 1.77 – 1.65 (m, 3H), 1.61 – 1.57 (m, 1H), 1.39 – 1.35 (m, 1H), 1.08 (t, J = 7.2 Hz, 3H). NH and CO_2H not observed.

^{13}C NMR (126 MHz, MeOD): δ 174.7, 168.1, 157.8, 153.1, 131.7, 125.1, 125.0, 123.7, 118.6, 103.9, 39.5, 33.9, 23.7, 21.7, 13.8, 13.8.

HRMS: exact mass calculated for $[M+H]^+$ ($C_{16}H_{18}NO_4$) requires m/z 288.1236, found m/z 288.1233.

Compound S128.

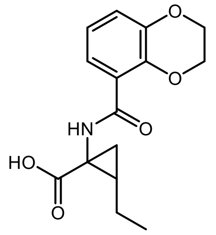

S128

^{1}H NMR (500 MHz, MeOD): δ 7.38 (dd, J = 7.8, 1.6 Hz, 1H), 6.99 (dd, J = 8.0, 1.6 Hz, 1H), 6.89 (t, J = 7.9 Hz, 1H), 4.41 – 4.37 (m, 2H), 4.31 – 4.27 (m, 2H), 1.72 – 1.63 (m, 2H), 1.63 – 1.55 (m, 1H), 1.56 – 1.51 (m, 1H), 1.29 – 1.24 (m, 1H), 1.05 (t, J = 7.3 Hz, 3H). NH and CO_2H not observed.

^{13}C NMR (126 MHz, MeOD): δ 174.7, 168.9, 145.4, 143.7, 123.8, 123.7, 121.9, 121.6, 66.2, 65.1, 39.4, 33.7, 23.6, 21.6, 13.7.

HRMS: exact mass calculated for $[M+H]^+$ ($C_{15}H_{18}NO_5$) requires m/z 292.1185, found m/z 292.1183.

Compound S129.

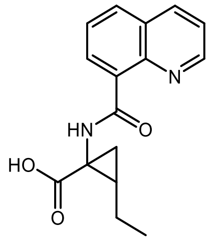

S129

^{1}H NMR (500 MHz, MeOD): δ 9.11 (dd, J = 4.7, 1.5 Hz, 1H), 8.77 (d, J = 8.2 Hz, 1H), 8.65 (dd, J = 7.4, 1.3 Hz, 1H), 8.29 (d, J = 8.1 Hz, 1H), 7.88 – 7.81 (m, 2H), 1.78 – 1.68 (m, 3H), 1.65 – 1.60 (m, 1H), 1.43 – 1.37 (m, 1H), 1.10 (t, J = 7.2 Hz, 3H). NH and CO_2H not observed.

^{13}C NMR (126 MHz, MeOD): δ 174.7, 169.1, 149.7, 143.1 (br), 134.8, 134.3, 130.5, 128.6, 123.1, 39.5, 33.6, 23.5, 21.7, 13.8. Two signals not observed.
HRMS: exact mass calculated for $[M+H]^+$ ($C_{16}H_{17}N_2O_3$) requires *m/z* 285.1239, found *m/z* 285.1235.

Compound S130.

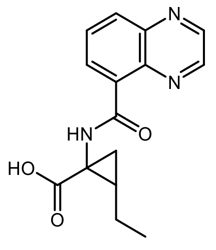

S130

^{1}H NMR (500 MHz, MeOD): δ 9.02 – 8.98 (m, 2H), 8.64 (dd, *J* = 7.4, 1.4 Hz, 1H), 8.28 (dd, *J* = 8.4, 1.4 Hz, 1H), 7.99 – 7.94 (m, 1H), 1.77 – 1.67 (m, 3H), 1.64 – 1.60 (m, 1H), 1.43 – 1.37 (m, 1H), 1.09 (t, *J* = 7.2 Hz, 3H). N*H* and CO_2*H* not observed.
^{13}C NMR (126 MHz, MeOD): δ 174.6, 168.1, 146.8, 145.9, 144.0, 141.5, 134.6, 134.3, 131.1, 131.0, 39.5, 33.8, 23.6, 21.7, 13.8.
HRMS: exact mass calculated for $[M+H]^+$ ($C_{15}H_{16}N_3O_3$) requires *m/z* 286.1192, found *m/z* 286.1187.

Single enantiomer data.
Compound 45a.

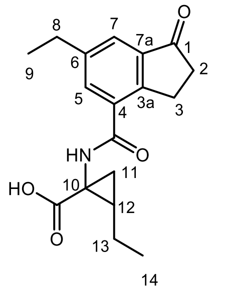

45a

Isomer 1.
υ_{max} (film): 3348 (br.), 2969, 2928, 2874, 1701, 1654, 1522, 1273 cm^{-1}.
^{1}H NMR (400 MHz, $CDCl_3$): δ 7.72 (s, 1H, $H^{5/7}$), 7.63 (s, 1H, $H^{5/7}$), 6.66 (s, 1H, N*H*), 3.40 – 3.34 (m, 2H, CH_2), 2.79 – 2.67 (m, 4H, H^8, CH_2), 1.74 – 1.51 (m, 4H, H^{11}, H^{12}, H^{13}), 1.45 – 1.38 (m, 1H, $H^{11'}$), 1.27 (t, *J* = 7.6 Hz, 3H, H^9), 1.07 (t, *J* = 7.2 Hz, 3H, H^{14}). CO_2*H* not observed.
^{13}C NMR (101 MHz, $CDCl_3$): δ 206.7 (C^1), 152.3 (Ar), 144.5 (Ar), 138.8 (Ar), 132.9 ($C^{5/7}$), 125.9 ($C^{5/7}$), 39.0 (C^{10}), 36.6 (CH_2), 34.2 (C^{12}), 28.5 (CH_2), 26.0 (CH_2), 22.9 (C^{11}), 20.8 (C^{13}), 15.6 (C^9), 13.6 (C^{14}). Three signals not observed.

Isomer 2.
υ_{max} (film): 3279 (br.), 2963, 2928, 2872, 1697, 1651, 1524, 1270, 1184 cm^{-1}.
^{1}H NMR (400 MHz, $CDCl_3$): δ 7.69 (s, 1H, $H^{5/7}$), 7.63 (s, 1H, $H^{5/7}$), 6.79 (s, 1H, N*H*), 3.42 – 3.29 (m, 2H, CH_2), 2.78 – 2.64 (m, 4H, H^8, CH_2), 1.77 – 1.48 (m, 4H, H^{11}, H^{12}, H^{13}), 1.46 – 1.38 (m, 1H, $H^{11'}$), 1.25 (t, *J* = 7.6 Hz, 3H, H^9), 1.05 (t, *J* = 7.2 Hz, 3H, H^{14}). CO_2*H* not observed.

^{13}**C NMR** (101 MHz, $CDCl_3$): δ 206.9 (C^1), 175.4 (*C*O), 169.1 (*C*O), 152.3 (Ar), 144.4 (Ar), 138.7 (Ar), 133.0 ($C^{5/7}$), 132.2 (Ar), 125.8 ($C^{5/7}$), 38.8 (C^{10}), 36.6 (CH_2), 34.1 (C^{12}), 28.5 (CH_2), 25.9 (CH_2), 23.2 (C^{11}), 20.7 (C^{13}), 15.5 (C^9), 13.6 (C^{14}).

Compound 43a.

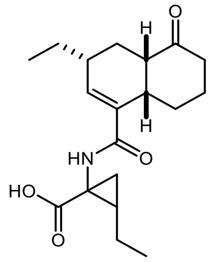

43a

Isomer 1.

υ_{max} (film): 3302 (br.), 2963, 2924, 2867, 1703, 1654, 1625, 1509, 1459 cm^{-1}.

^{1}**H NMR** (400 MHz, Acetone-d_6): δ 7.97 (s, 1H), 6.43 (s, 1H), 3.19 – 2.55 (br. m, 2H), 2.52 – 2.35 (m, 2H), 2.24 – 2.13 (m, 2H), 2.00 – 1.91 (m, 2H), 1.69 – 1.32 (m, 10H), 1.20 – 1.14 (m, 1H), 1.01 – 0.92 (m, 6H).

^{13}**C NMR** (101 MHz, Acetone-d_6): δ 212.9, 138.0, 136.5, 51.0, 38.9, 37.5, 32.6, 28.9, 28.5, 28.0, 25.6, 22.3, 21.4, 13.8, 11.4. Four signals not observed.

Isomer 2.

υ_{max} (film): 3320 (br), 2961, 2934, 2876, 1703, 1656, 1630, 1519, 1459, 1190 cm^{-1}.

^{1}**H NMR** (500 MHz, Acetone-d_6): δ 7.92 (s, 1H), 6.38 (s, 1H), 3.00 – 2.92 (m, 1H), 2.92 – 2.63 (br. s, 1H), 2.51 – 2.34 (m, 2H), 2.24 – 2.10 (m, 2H), 1.99 – 1.91 (m, 2H), 1.67 – 1.35 (m, 10H), 1.13 – 1.07 (m, 1H), 0.96 (t, *J* = 7.5 Hz, 6H).

^{13}**C NMR** (101 MHz, Acetone-d_6): δ 213.0, 172.8, 138.1, 136.0, 51.0, 38.9, 38.9, 37.6, 32.5, 29.0, 28.6, 28.0, 25.6, 22.6, 21.3, 13.7, 11.4. Two signals not observed.

Isomer 3.

υ_{max} (film): 3302 (br), 2960, 2934, 2870, 1697, 1656, 1625, 1519, 1191 cm^{-1}.

^{1}**H NMR** (600 MHz, Acetone-d_6): δ 7.96 (s, 1H), 6.43 (s, 1H), 2.98 – 2.93 (m, 1H), 2.92 – 2.54 (br. s, 1H), 2.46 (td, *J* = 14.3, 6.0 Hz, 1H), 2.41 – 2.35 (m, 1H), 2.24 – 2.13 (m, 2H), 2.01 – 1.92 (m, 2H), 1.67 – 1.34 (m, 10H), 1.17 (dd, *J* = 9.2, 4.8 Hz, 1H), 1.01 – 0.93 (m, 6H).

^{13}**C NMR** (101 MHz, Acetone-d_6): δ 213.0, 138.0, 136.5, 51.0, 38.9, 37.5, 32.6, 28.9, 28.5, 28.0, 25.6, 22.3, 21.4, 13.7, 11.4. Four signals not observed.

Isomer 4.

υ_{max} (film): 3307 (br), 2961, 2935, 2870, 1701, 1654, 1638, 1522, 1459, 1186 cm^{-1}.

^{1}**H NMR** (400 MHz, Acetone-d_6): δ 7.94 (s, 1H), 6.38 (s, 1H), 3.00 – 2.90 (m, 1H), 2.51 – 2.33 (m, 2H), 2.23 – 2.13 (m, 2H), 2.00 – 1.90 (m, 2H), 1.69 – 1.34 (m, 10H), 1.11 – 1.06 (m, 1H), 0.99 – 0.91 (m, 6H). CO_2H not observed.

^{13}**C NMR** (101 MHz, Acetone-d_6): δ 213.0, 169.8, 138.2, 135.9, 51.0, 38.9, 38.9, 37.6, 32.3, 29.0, 28.6, 28.0, 25.6, 22.3, 21.3, 13.8, 11.5. Two signals not observed.

Biological Results.

Supplementary Table 7.

	Post-emergence					Pre-emergence				
Compound	AMARE	LOLPE	STEME	DIGSA	Symptom	AMARE	LOLPE	STEME	DIGSA	Symptom
(+)-1	90	60	NT	90	ST/DS	80	90	NT	100	ST/DS
(±)-2	0	0	0	0	-	0	0	0	0	-
(±)-3	0	0	0	0	-	0	0	0	0	-
S4	0	0	0	0	-	0	0	0	0	-
16	0	0	0	0	ST	0	0	50	0	ST
S36	0	0	0	0	ST	0	0	0	50	ST
17	0	0	0	0	-	0	0	0	0	-
S37	0	0	0	0	-	0	0	0	0	-
18	0	0	0	0	-	0	0	0	0	-
S38	10	10	30	60	NC/ST	20	0	20	20	NC/ST
19	NT	NT	NT	NT	-	NT	NT	NT	NT	-
20	0	0	0	0	-	0	0	0	0	-
21	0	0	70	20	NC/ST	0	0	0	0	NC/ST
22	0	0	0	0	-	0	0	0	0	-
S42	0	0	0	0	-	0	0	0	0	-
23	0	0	0	0	-	0	0	0	0	-
S43	0	0	0	0	-	0	0	0	0	-
24	0	0	0	0	-	0	0	0	0	-
25	0	0	0	0	-	0	0	0	0	-
26	0	0	0	0	-	0	0	0	0	-
27	0	0	0	0	-	0	0	0	0	-
28	0	0	0	0	-	0	0	0	0	-
29	0	0	0	0	-	0	0	0	0	-
30	0	0	0	0	-	0	0	0	0	-
31	0	0	0	0	-	0	0	0	0	-
32	0	0	0	0	ST	50	0	80	0	ST
33	40	0	50	60	NC/ST	70	40	70	80	NC/ST
34	0	0	0	0	-	0	0	0	0	-
35	0	0	0	0	ST	20	0	50	50	ST
36	50	40	0	60	ST	40	30	50	50	ST
37	0	0	0	0	-	0	0	0	0	-
38a	70	70	70	80	NC/ST	80	60	80	80	NC/ST
38b	0	0	0	0	-	0	0	0	0	-
39b	20	0	40	0	ST	30	40	70	0	ST
40b	0	0	0	0	-	0	0	0	0	-
41a	30	20	30	60	GI/ST	0	20	80	0	GI/ST
41b	0	0	0	0	-	0	0	0	0	-
42a	0	0	0	0	-	0	0	0	0	-

42b	0	0	0	0	-	0	0	0	0	-
43a	30	10	0	50	GI/ST	30	60	40	80	GI/ST
43b	0	0	0	0	-	0	0	0	0	-
44a	0	0	0	0	-	0	0	0	0	-
44b	NT	NT	NT	NT	-	NT	NT	NT	NT	-
45a	30	20	10	100	NC/ST	20	20	20	40	NC/ST
S83	0	0	0	0	-	0	0	0	0	-
S84	0	0	0	0	-	0	0	0	0	-
S85	0	0	0	0	-	0	0	0	0	-
45b	0	0	0	0	-	0	0	0	0	-
S86	0	0	0	0	-	0	0	0	0	-
S87	0	0	0	0	-	0	0	0	0	-
S88	0	0	0	0	-	0	0	0	0	-
S89	0	0	0	0	-	0	0	0	0	-
S90	0	0	0	0	-	0	0	0	0	-
S91	0	0	0	0	-	0	0	0	0	-
S92	0	0	0	0	-	0	0	0	0	-
S93	0	0	0	10	NC	0	0	0	0	-
S94	0	0	0	0	-	0	0	0	0	-
S95	0	0	0	0	-	0	0	0	0	-
S96	0	0	0	0	-	0	0	0	0	-
S97	10	0	0	0	NC	0	0	0	0	NC
S98	10	0	0	10	NC	0	0	0	0	NC
S99	0	0	0	0	-	0	0	0	0	-
S100	0	0	0	0	-	0	0	0	0	-
S101	0	0	0	0	-	0	0	0	0	-
S102	10	0	10	0	NC/BL	0	0	0	0	NC/BL
S103	0	0	0	0	-	0	0	0	0	-
S104	0	0	0	0	-	0	0	0	0	-
S105	0	0	0	0	-	0	0	0	0	-
S106	0	0	0	0	-	0	0	0	0	-
S107	20	10	10	10	NC	0	0	0	0	NC
S108	0	0	0	0	-	0	0	0	0	-
S109	10	0	10	0	BL/NC	0	0	0	0	BL/NC
S110	60	10	80	10	ST/NC	0	0	0	0	ST/NC
S111	20	0	100	20	NC/MR	0	0	0	0	MC/MR
S112	0	0	0	0	-	0	0	0	0	-
S113	0	0	0	0	-	0	0	0	0	-
S114	0	0	0	0	-	0	0	0	0	-
S115	0	0	0	0	-	0	0	0	0	-

S116	0	0	0	0	-	0	0	0	0	-
S117	0	0	0	0	-	0	0	0	0	-
S118	0	0	0	0	-	0	0	0	0	-
S119	0	0	0	0	-	0	0	0	0	-
S120	0	0	0	0	-	0	0	0	0	-
S121	0	0	0	0	-	0	0	0	0	-
S122	0	0	0	0	-	0	0	0	0	-
S123	0	0	0	0	-	0	0	0	0	-
S124	0	0	0	0	-	0	0	0	0	-
S125	0	0	10	0	BL	0	0	0	0	BL
S126	0	0	0	0	-	0	0	0	0	-
S127	0	0	0	0	-	0	0	0	0	-
S128	0	0	0	0	-	0	0	0	0	-
S129	0	0	70	0	BL/NC	0	0	0	0	BL/NC
S130	0	0	0	0	-	0	0	0	0	-

Supplementary Table 8.

	Post-emergence						
Compound	AMARE	SOLNI	SETFA	LOLPE	ECHCG	IPOHE	Symptom
45a (isomer 1)	0	0	NT	0	0	0	-
45a (isomer 2)	0	50	NT	40	40	30	ST
43a (isomer 1)	0	0	0	0	0	0	-
43a (isomer 2)	10	10	0	0	0	10	ST/CL
43a (isomer 3)	10	70	0	50	20	50	ST/CL
43a (isomer 4)	10	80	70	50	50	80	

Supplementary Table 9.

	Pre-emergence						
Compound	AMARE	SOLNI	SETFA	LOLPE	ECHCG	IPOHE	Symptom
45a (isomer 1)	0	0	0	0	0	0	-
45a (isomer	20	60	20	40	0	0	ST

2)							
43a (isomer 1)	0	0	0	0	0	0	-
43a (isomer 2)	0	0	0	0	0	0	-
43a (isomer 3)	20	70	50	60	10	10	ST
43a (isomer 4)	50	80	60	70	0	80	

Supplementary Figures 1-339: Spectra.

Supplementary Figure 1: ^{1}H NMR 5.

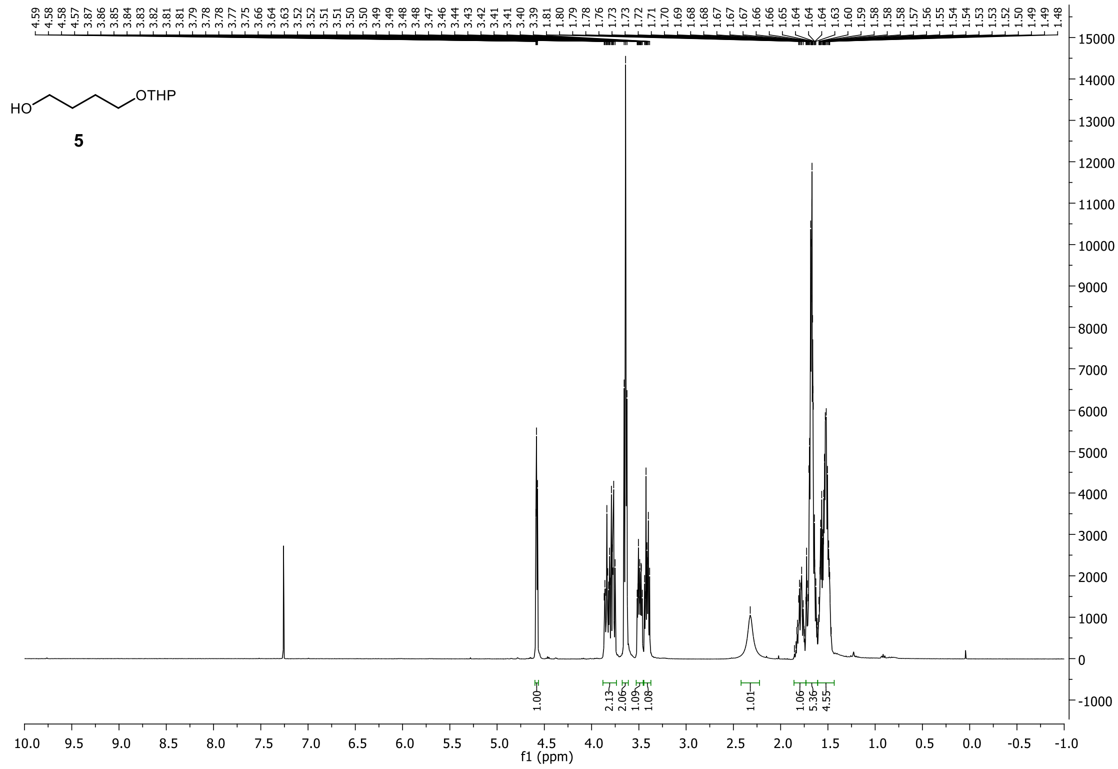

Supplementary Figure 2: ^{13}C NMR 5.

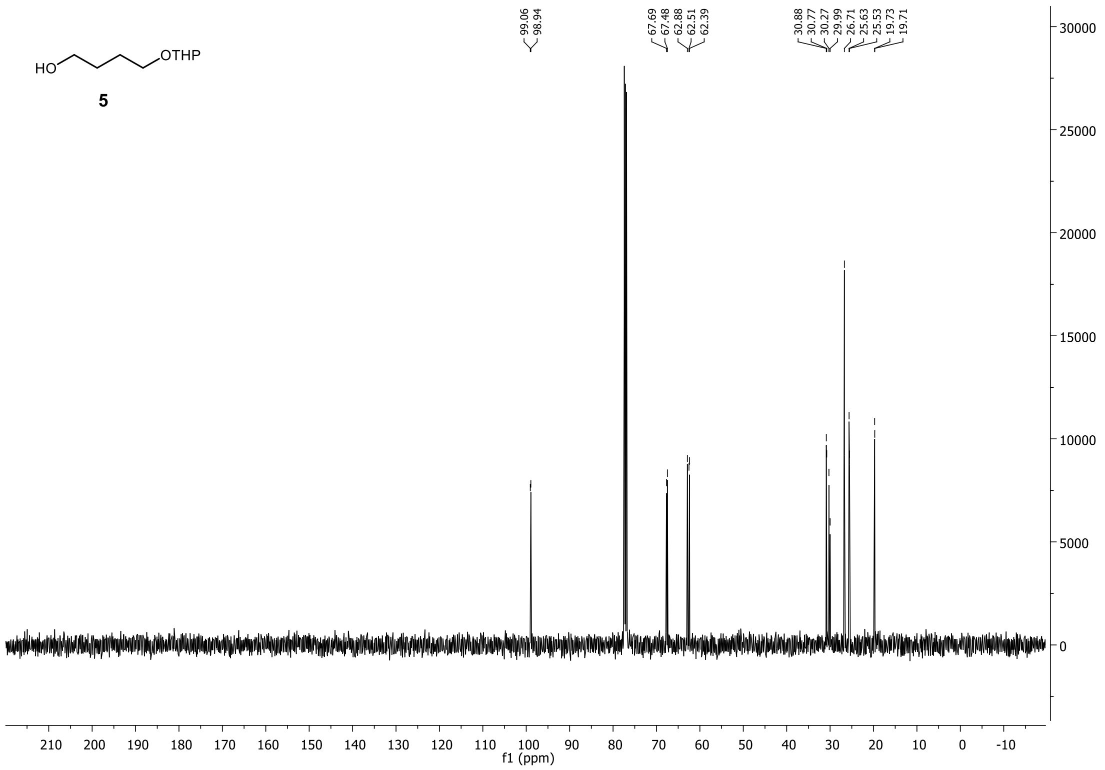

Supplementary Figure 3: ^{1}H NMR 6.

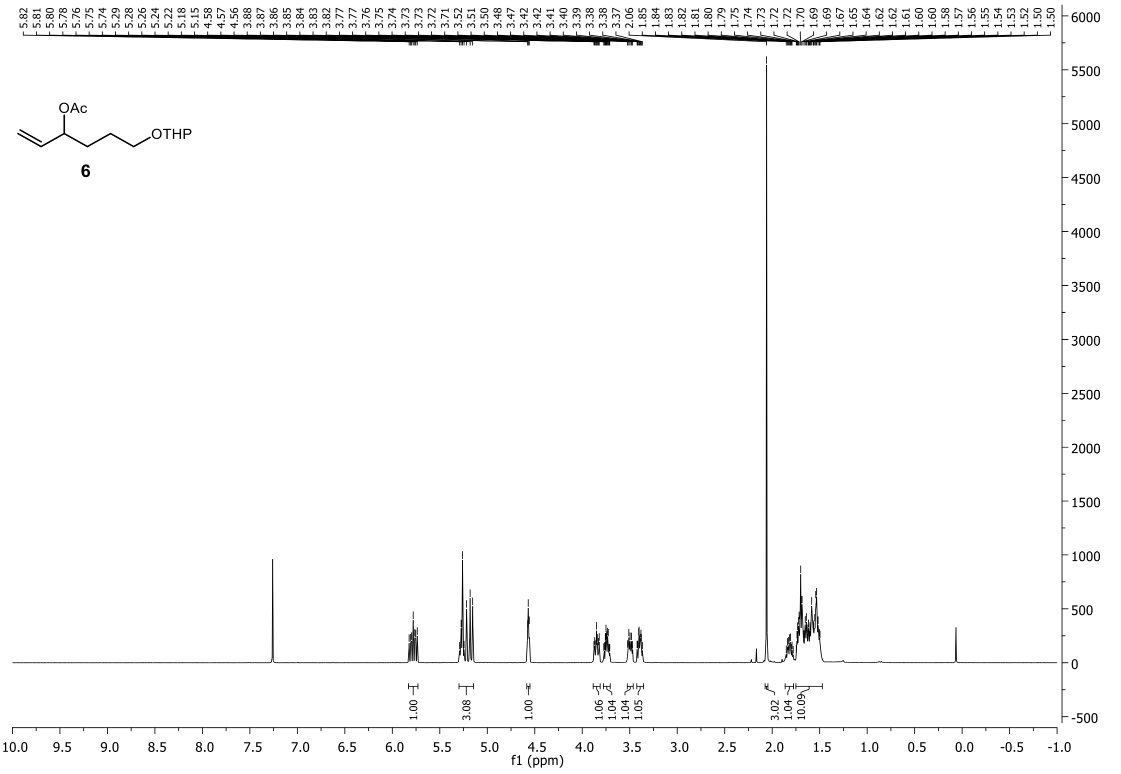

Supplementary Figure 4: ^{13}C NMR 6.

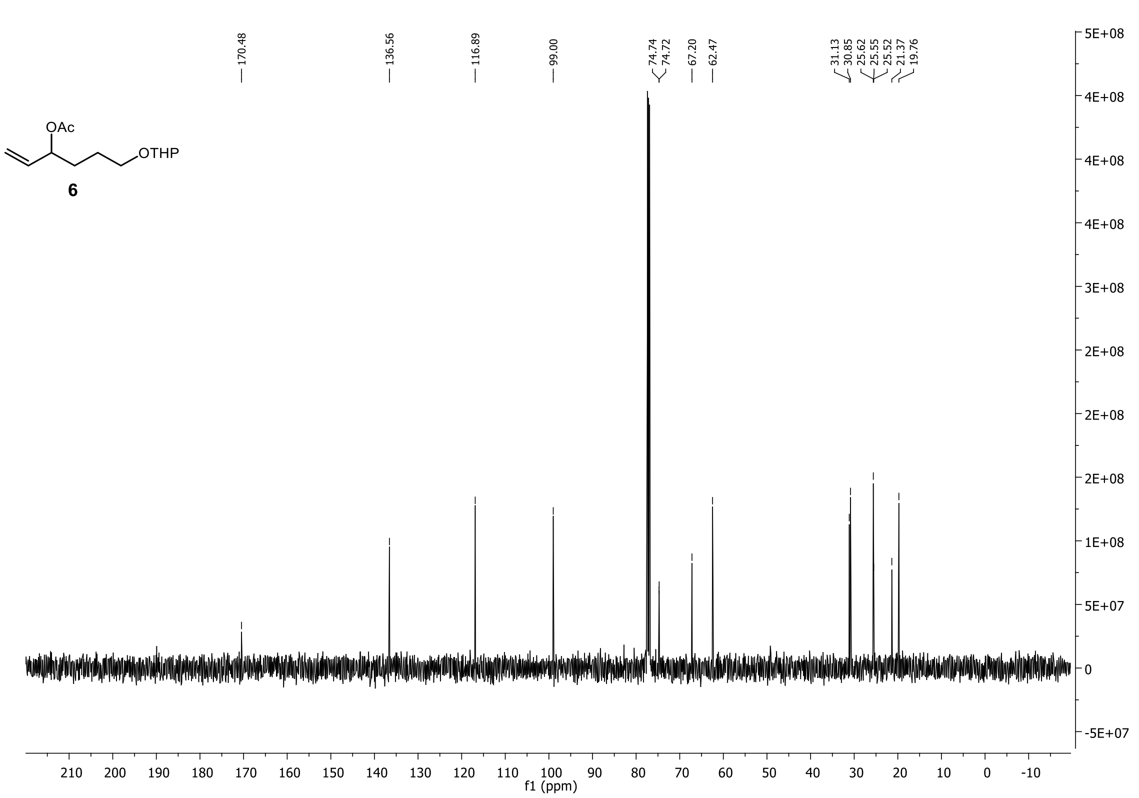

Supplementary Figure 5: ^{1}H NMR S1.

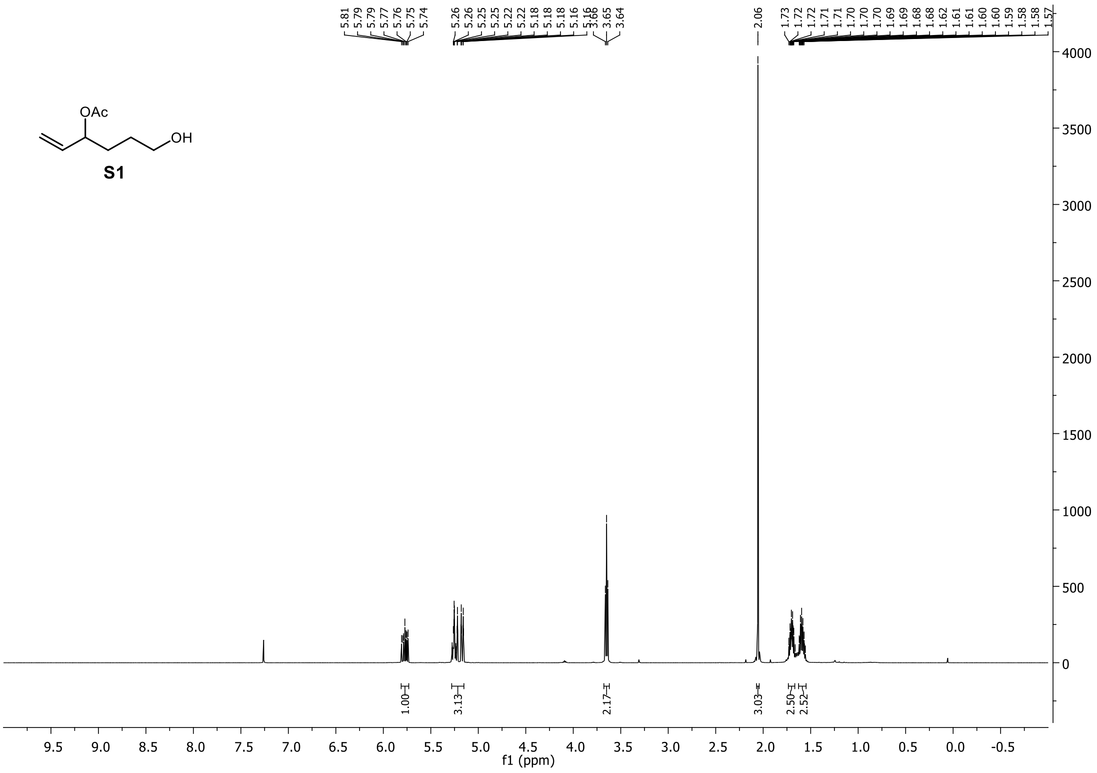

Supplementary Figure 6: ^{13}C NMR S1.

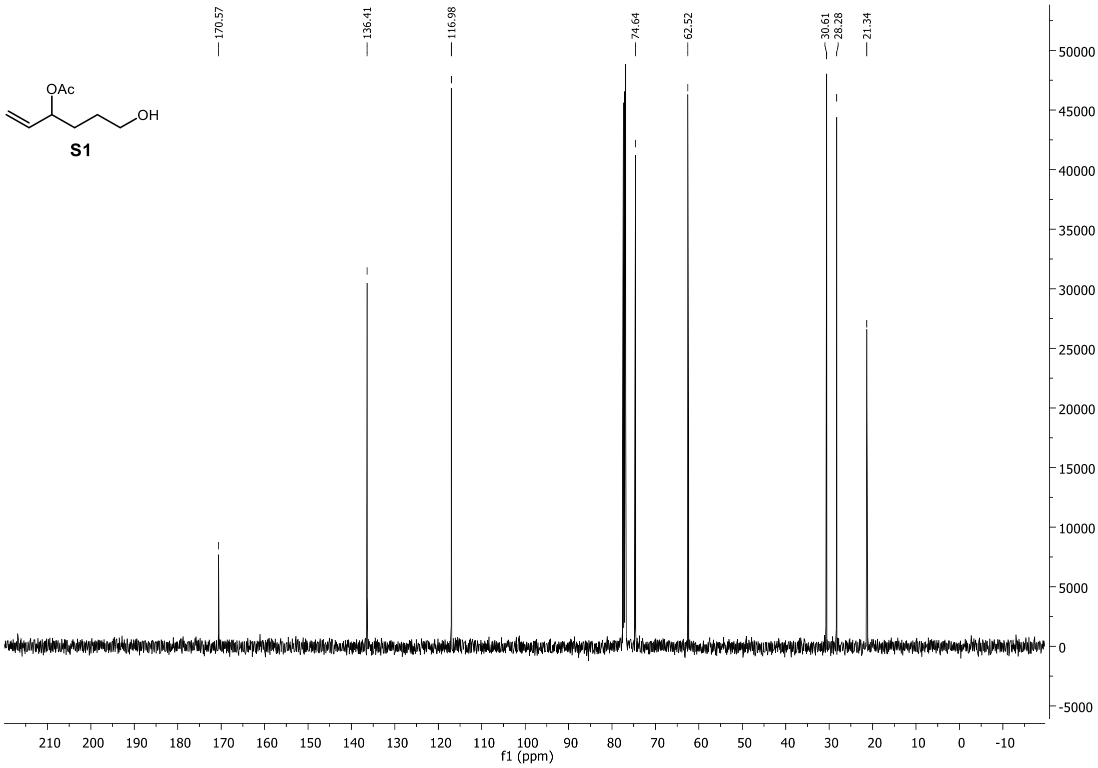

Supplementary Figure 7: ^{1}H NMR 7.

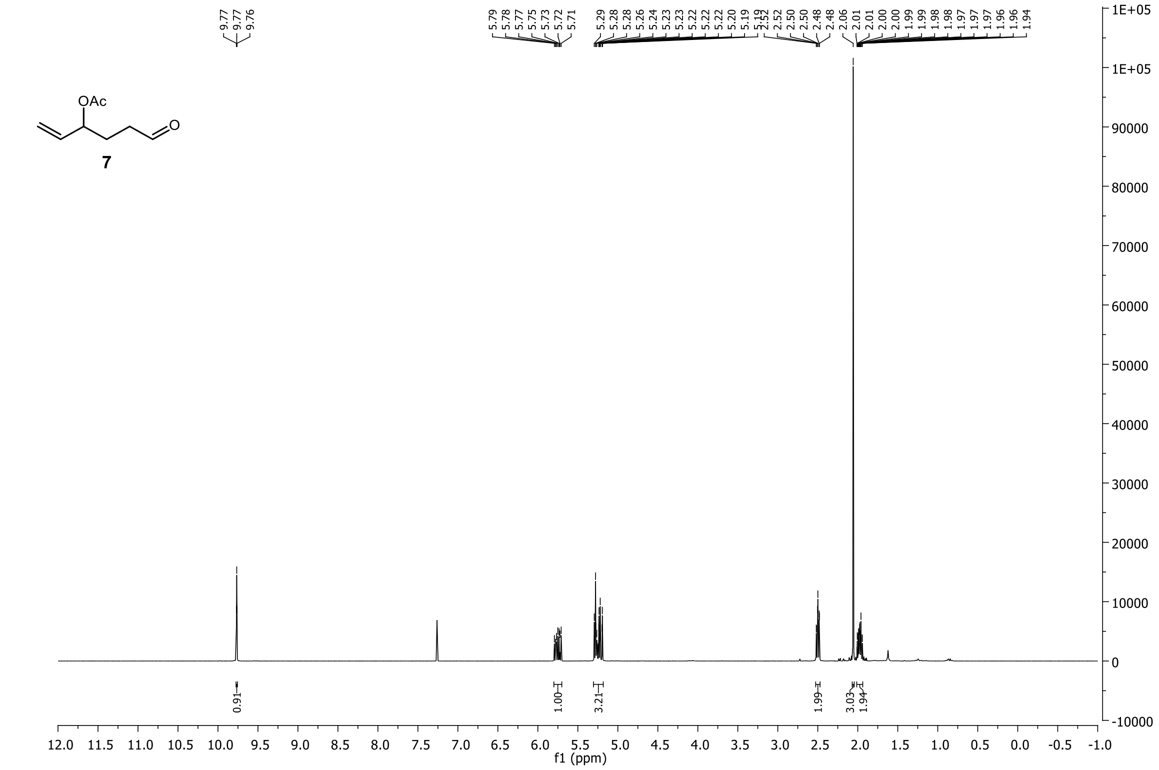

Supplementary Figure 8: ^{13}C NMR 7.

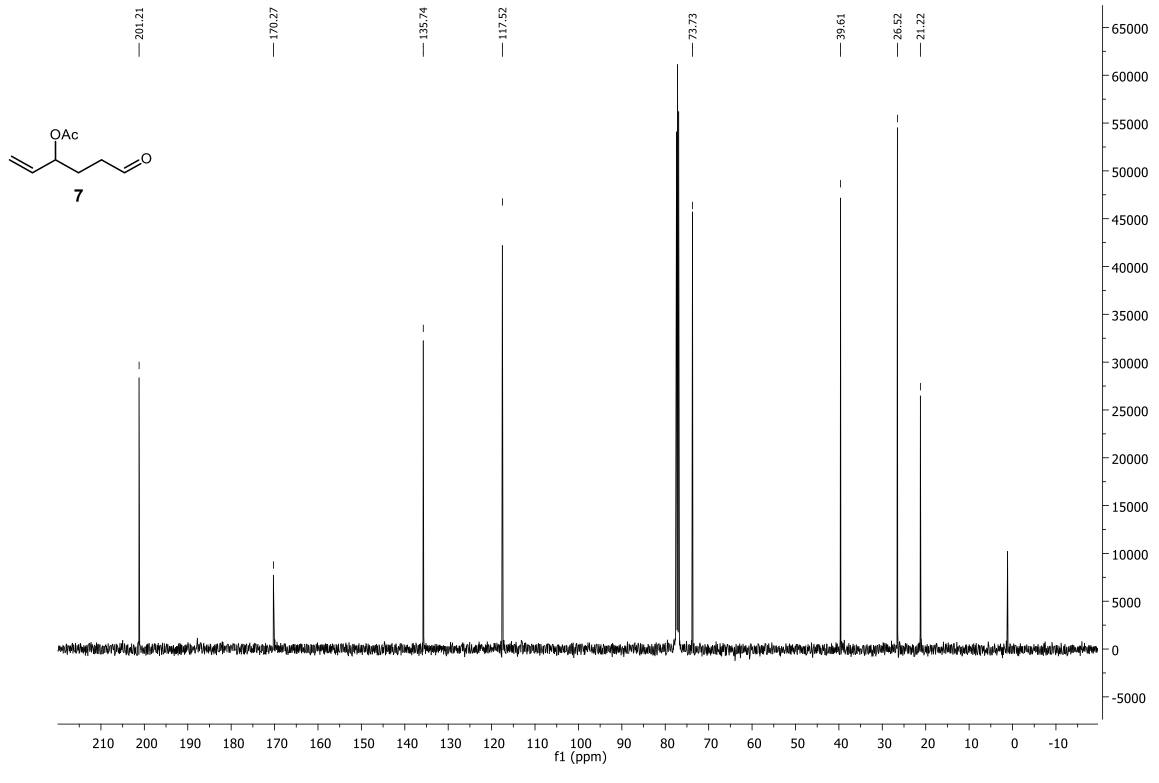

Supplementary Figure 9: ^{1}H NMR *syn*-9.

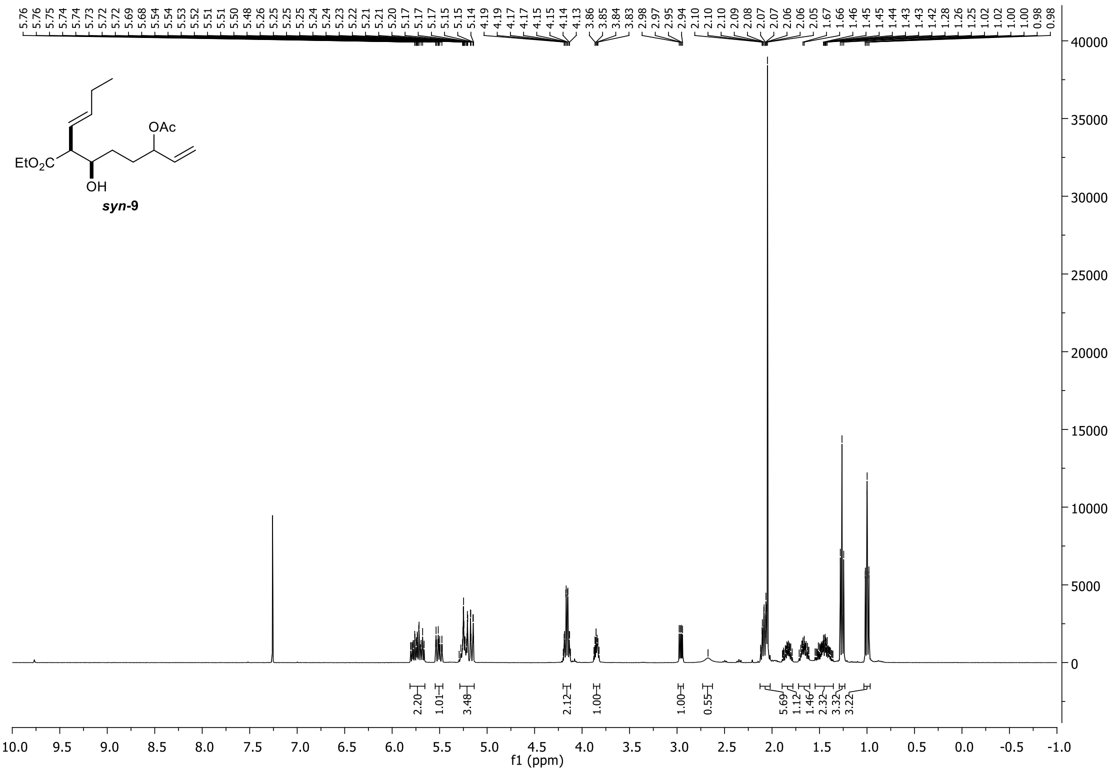

Supplementary Figure 10: ^{1}H NMR *syn*-9 showing alkene isomerization.

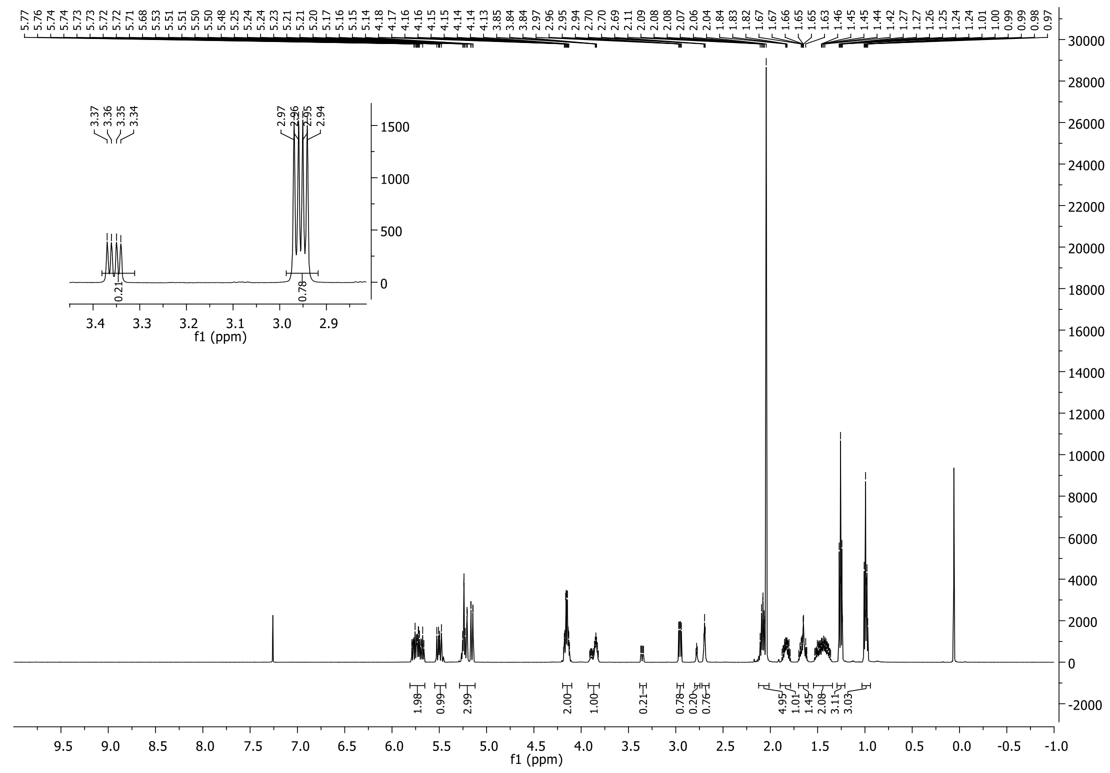

Supplementary Figure 11: ^13^C NMR *syn*-9.

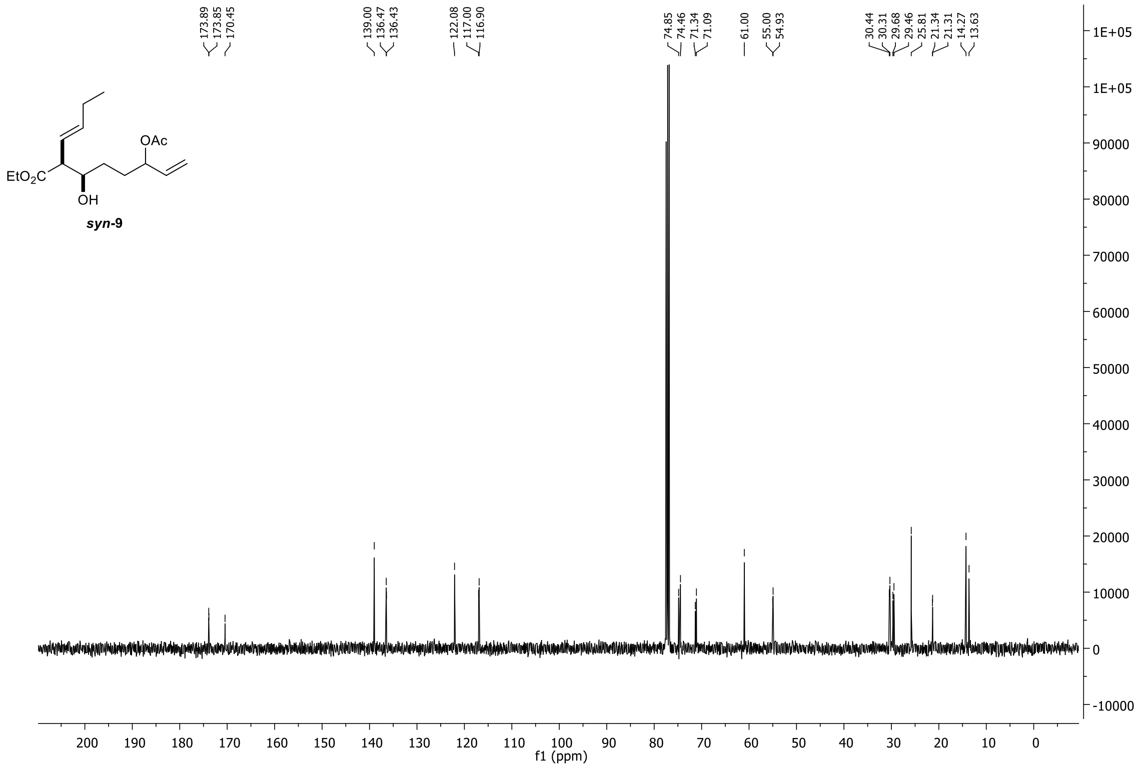

Supplementary Figure 12: ^1^H NMR *anti*-9.

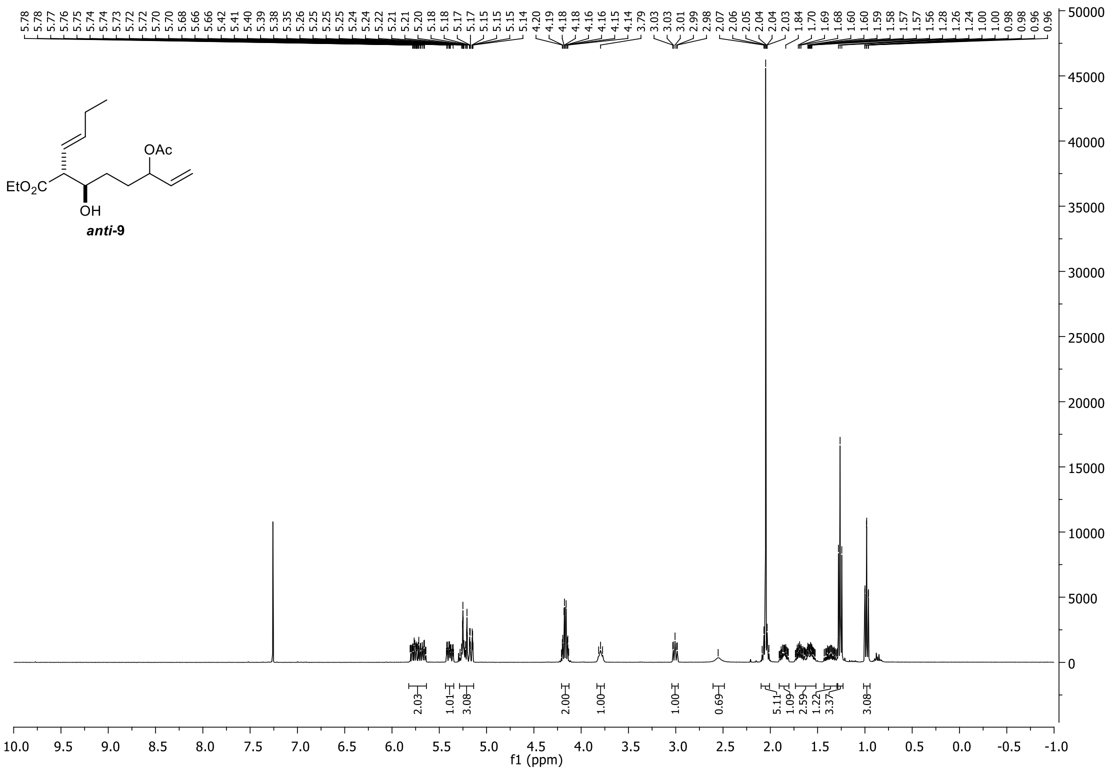

Supplementary Figure 13: ^{13}C NMR *anti*-9.

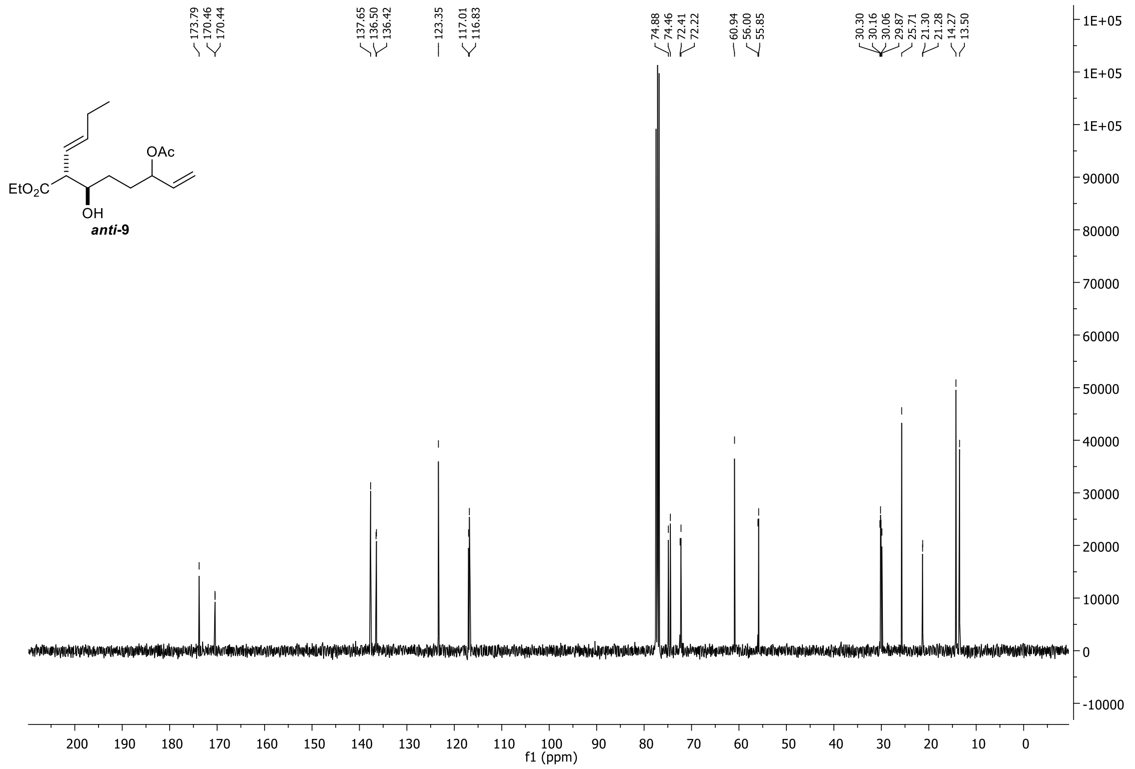

Supplementary Figure 14: ^{1}H NMR 11.

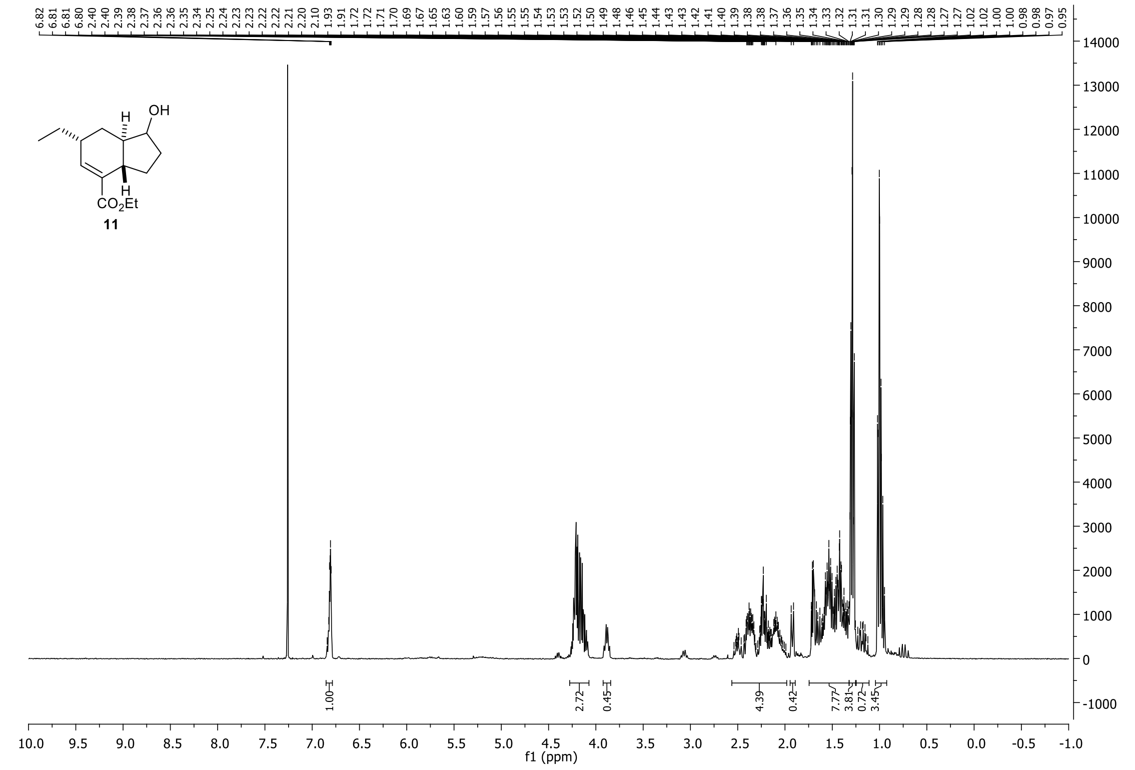

Supplementary Figure 15: ^{13}C NMR of 11.

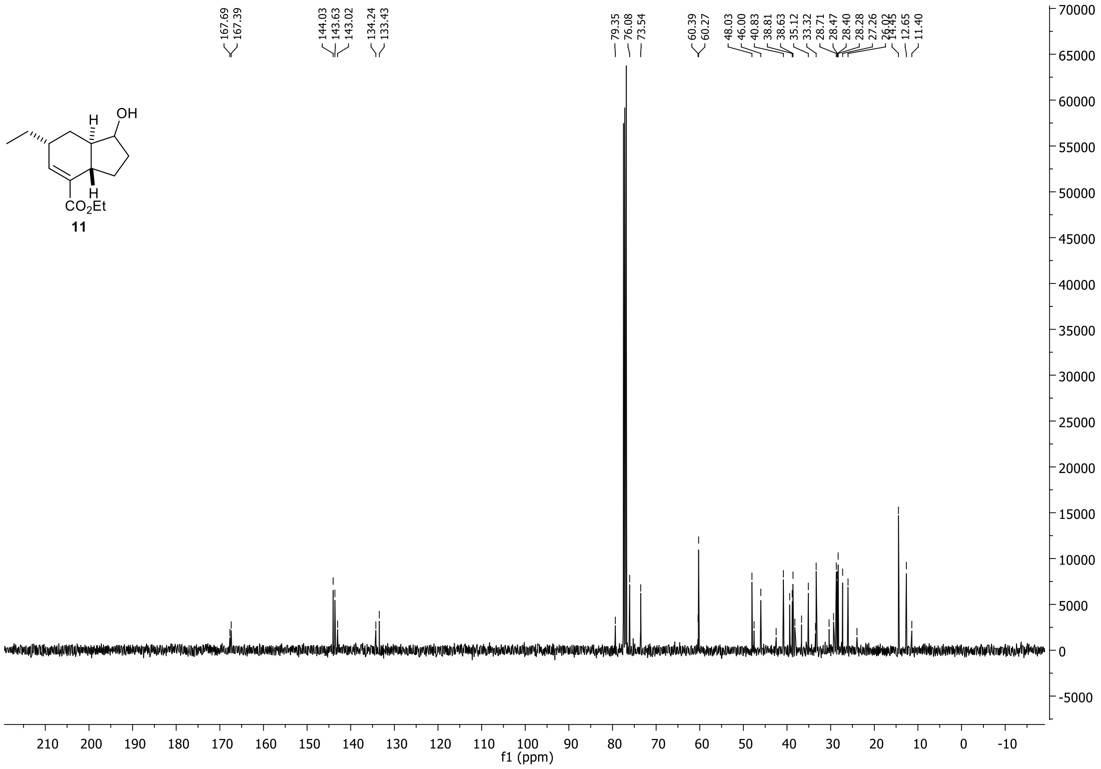

Supplementary Figure 16: ^{1}H NMR of S3.

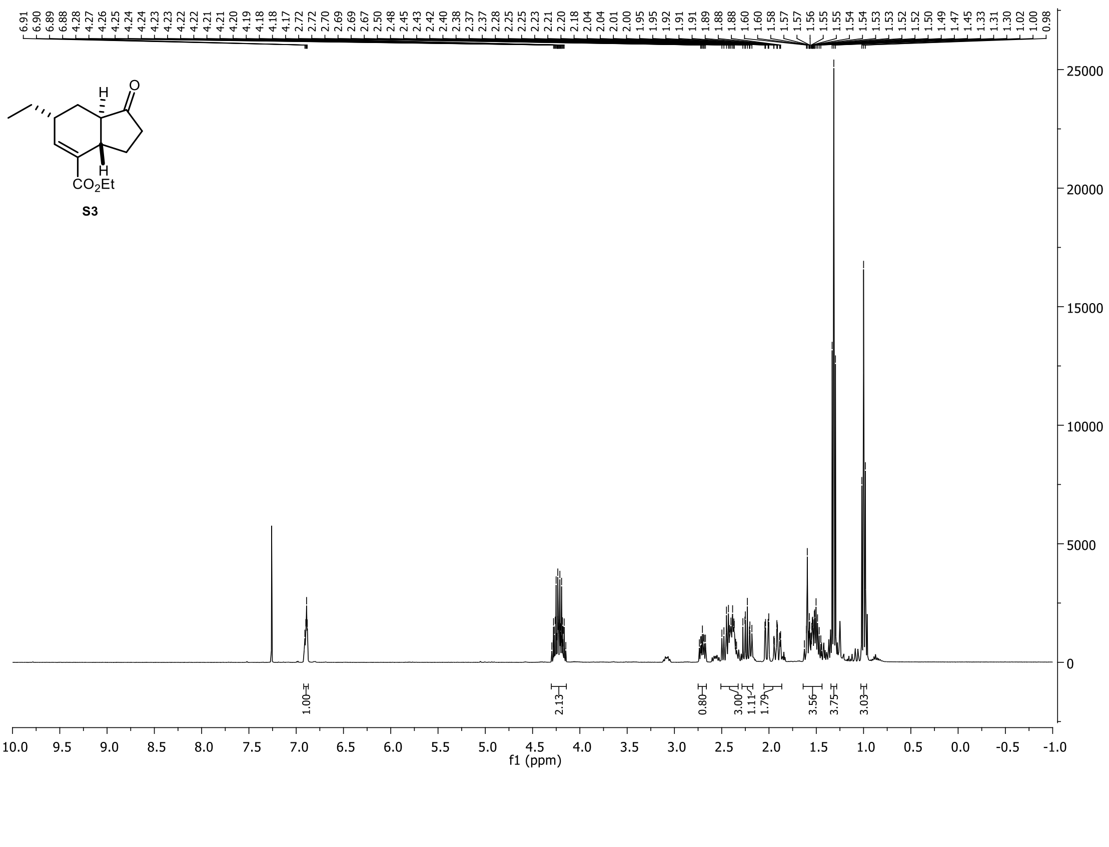

Supplementary Figure 17: ^{13}C NMR S3.

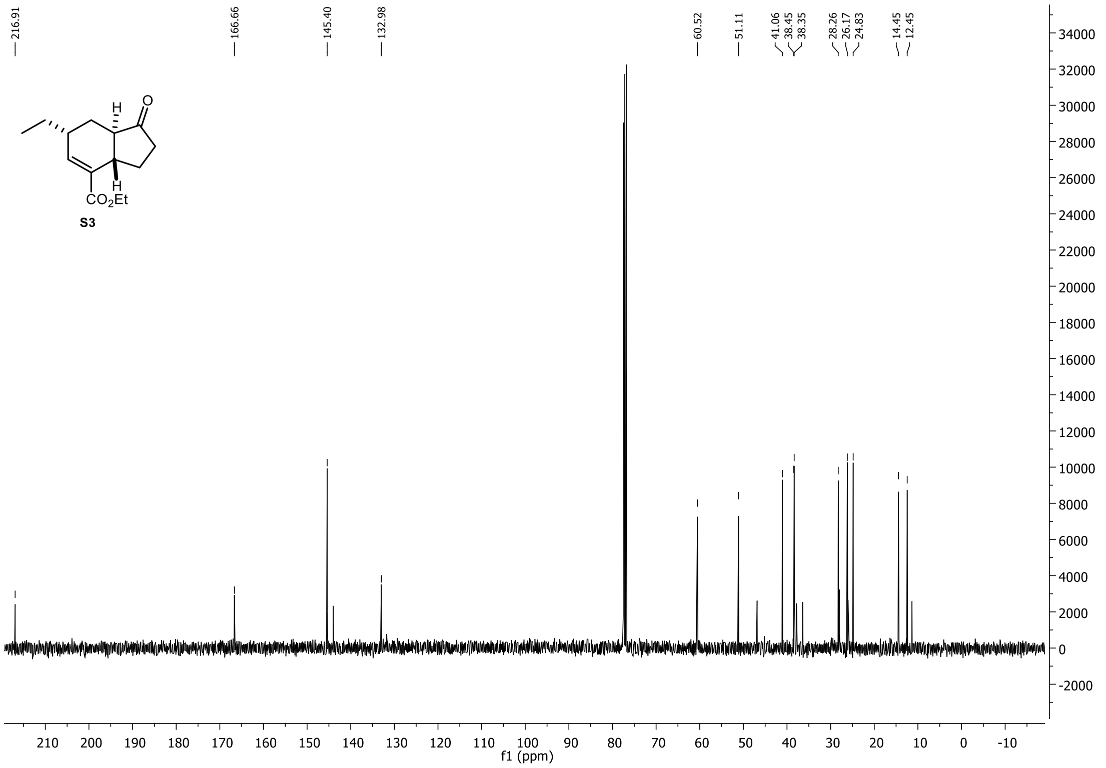

Supplementary Figure 18: ^{1}H NMR S9.

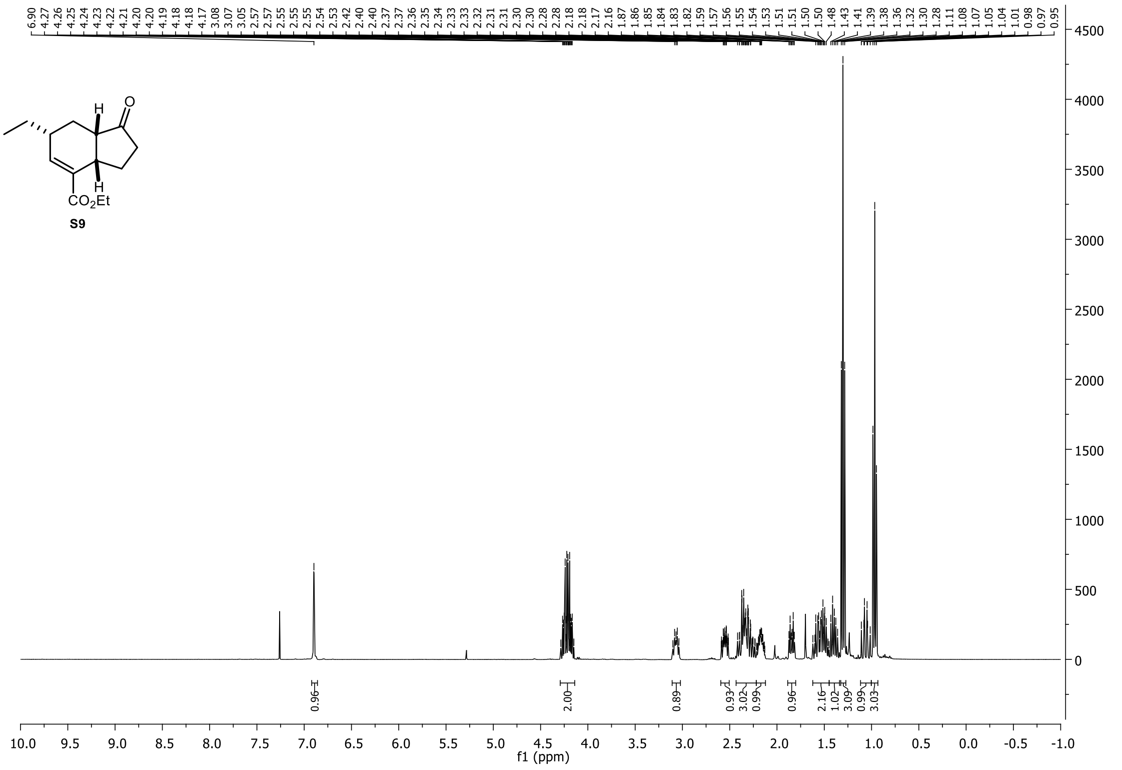

Supplementary Figure 19: ^{13}C NMR S9.

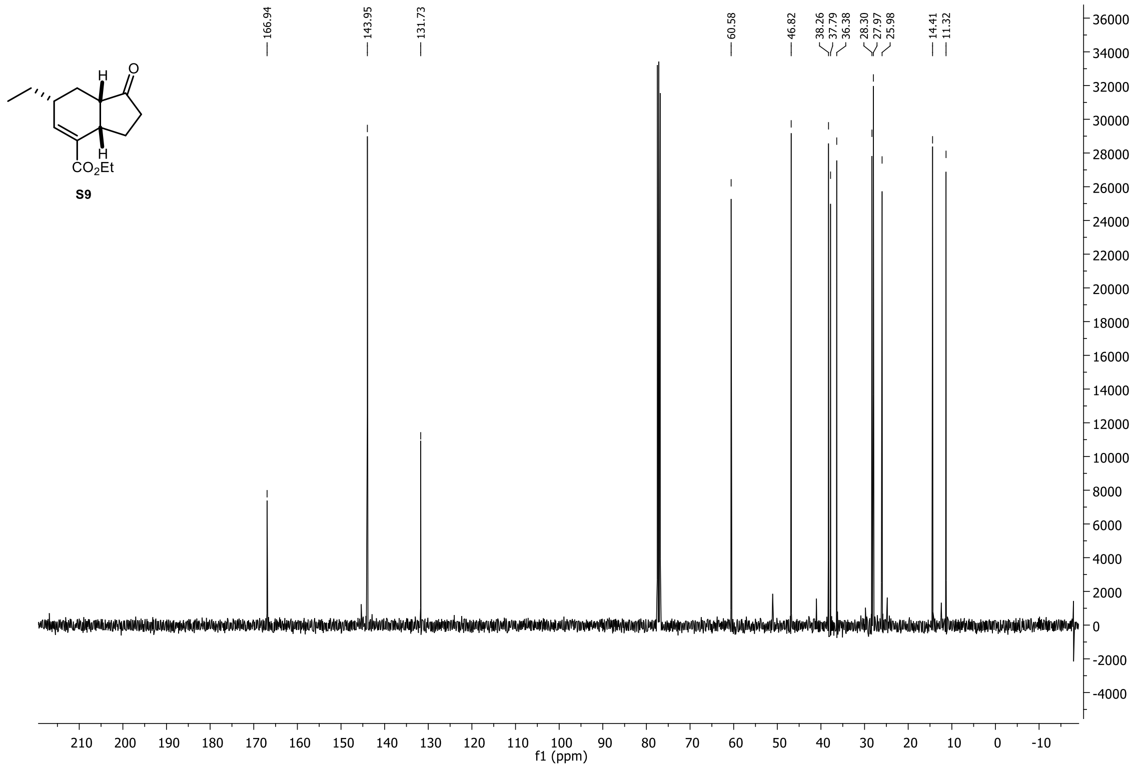

Supplementary Figure 20: ^{1}H NMR (±)-2.

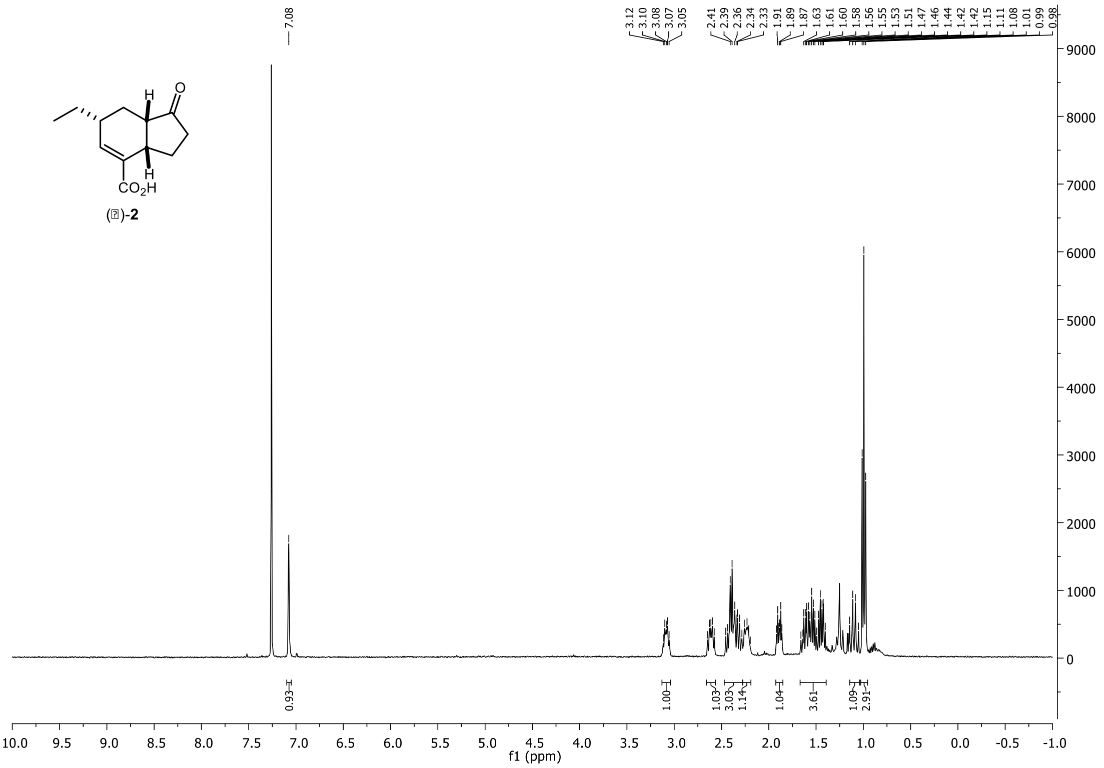

Supplementary Figure 21: ^{13}C NMR (±)-2.

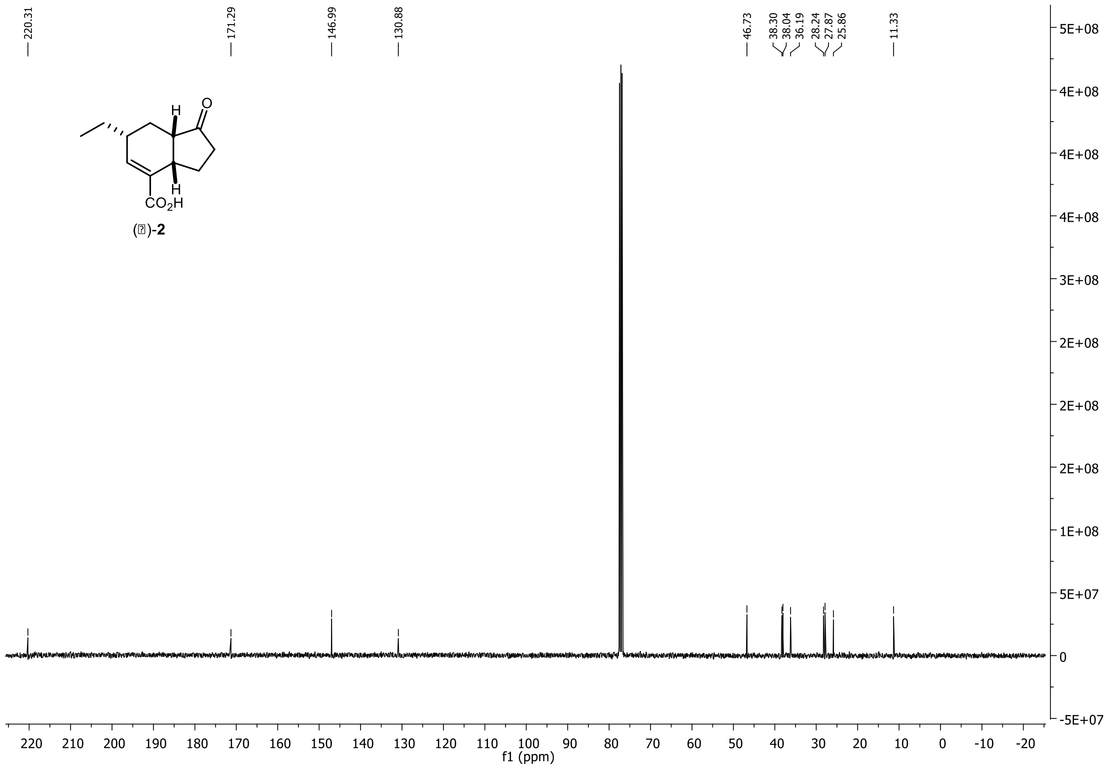

Supplementary Figure 22: ^{1}H NMR S10.

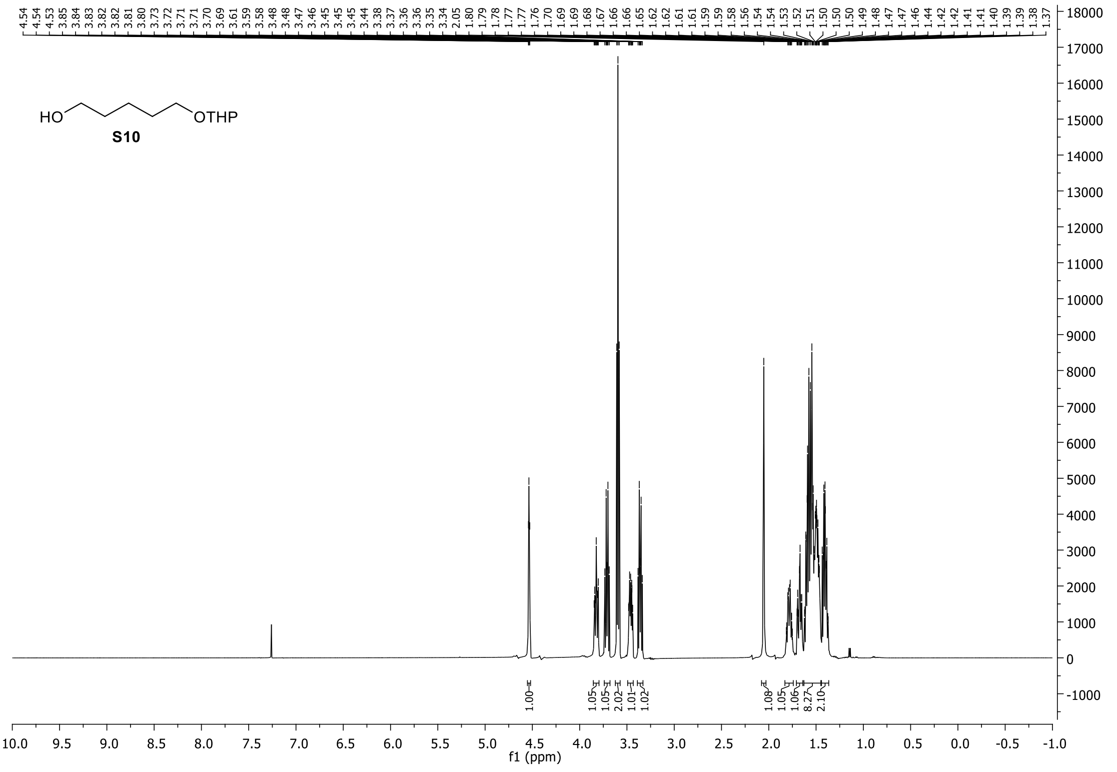

Supplementary Figure 23: ^{13}C NMR S10.

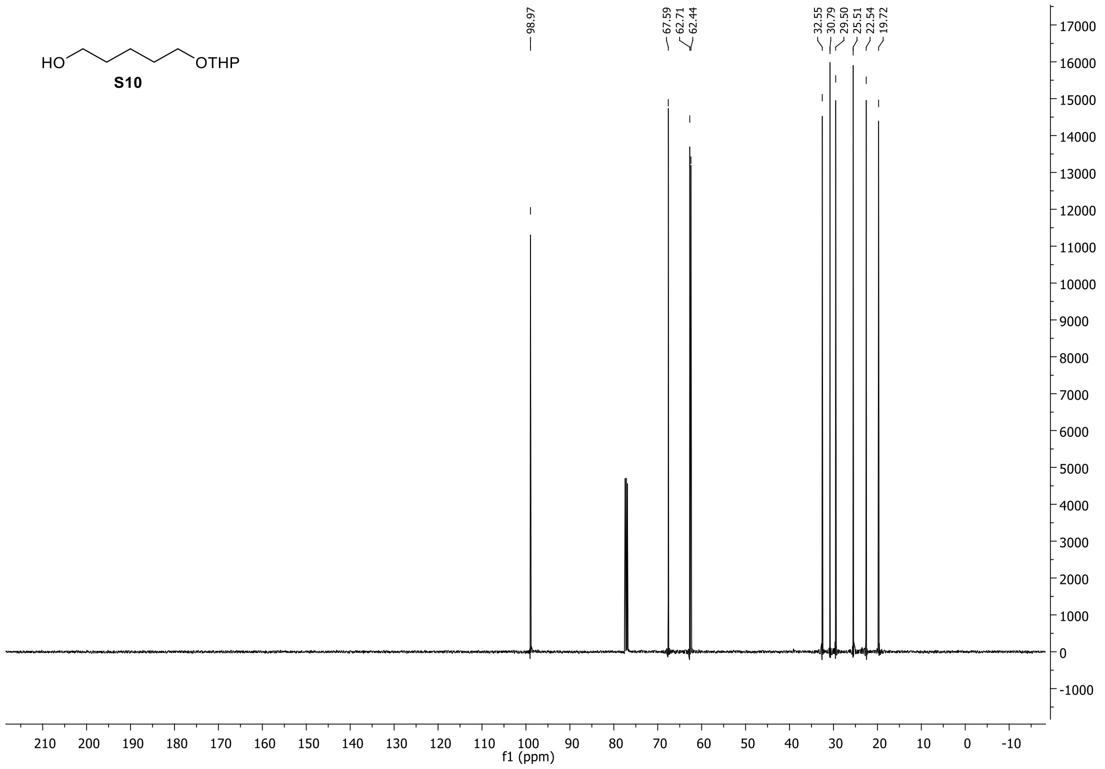

Supplementary Figure 24: ^{1}H NMR S12.

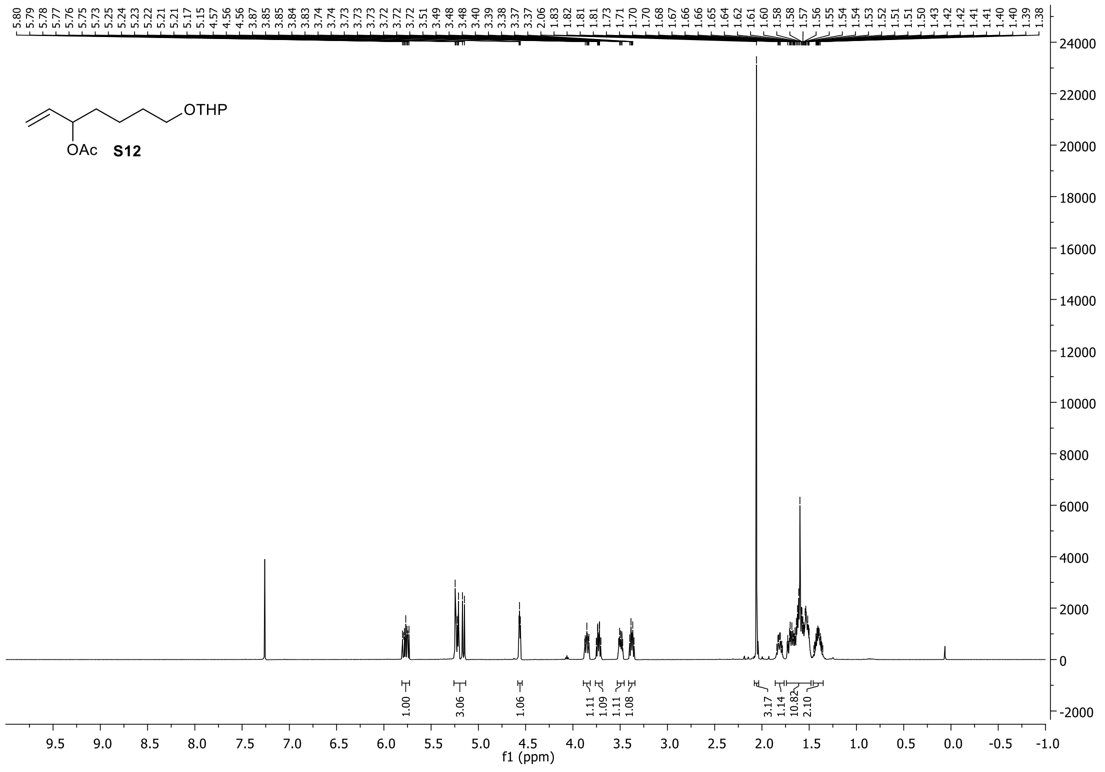

Supplementary Figure 25: ^{13}C NMR S12.

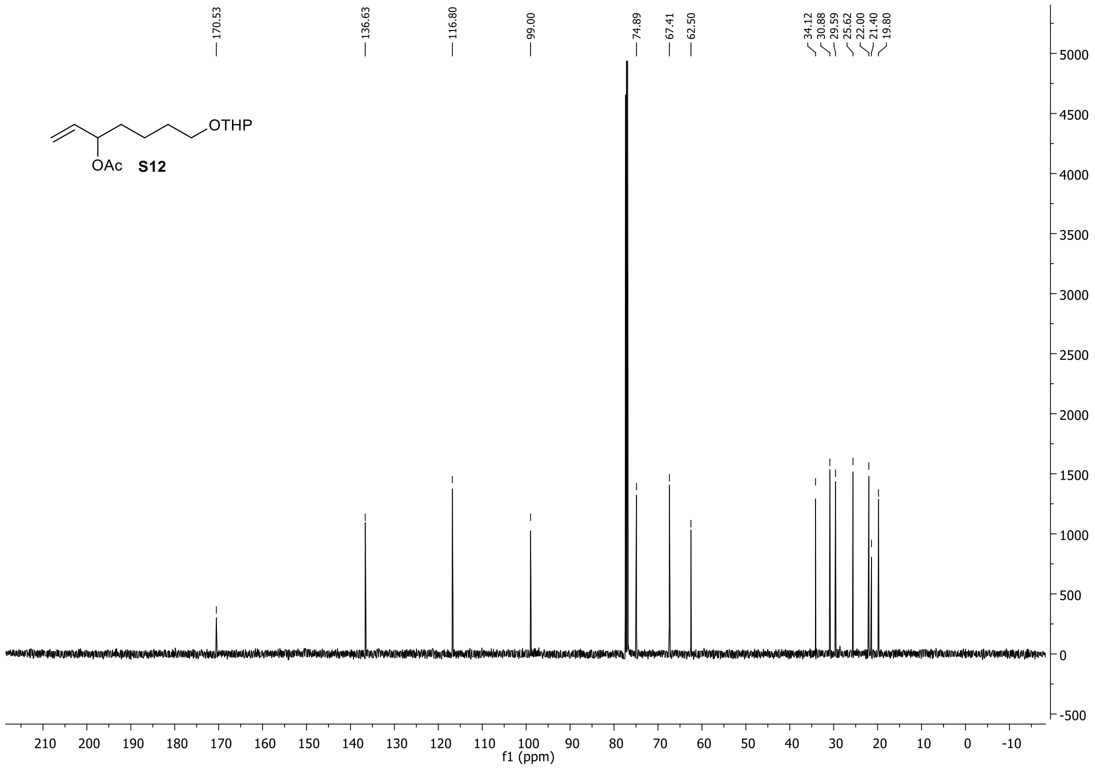

Supplementary Figure 26: ^{1}H NMR S13.

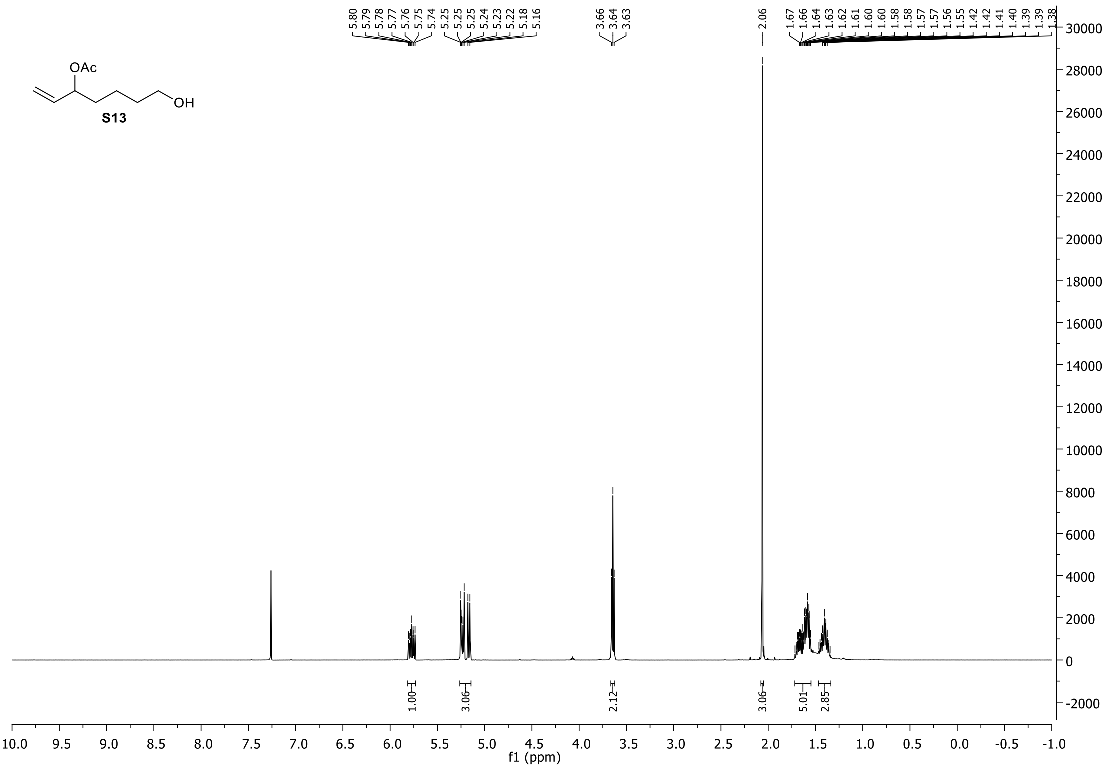

Supplementary Figure 27: ^{13}C NMR S13.

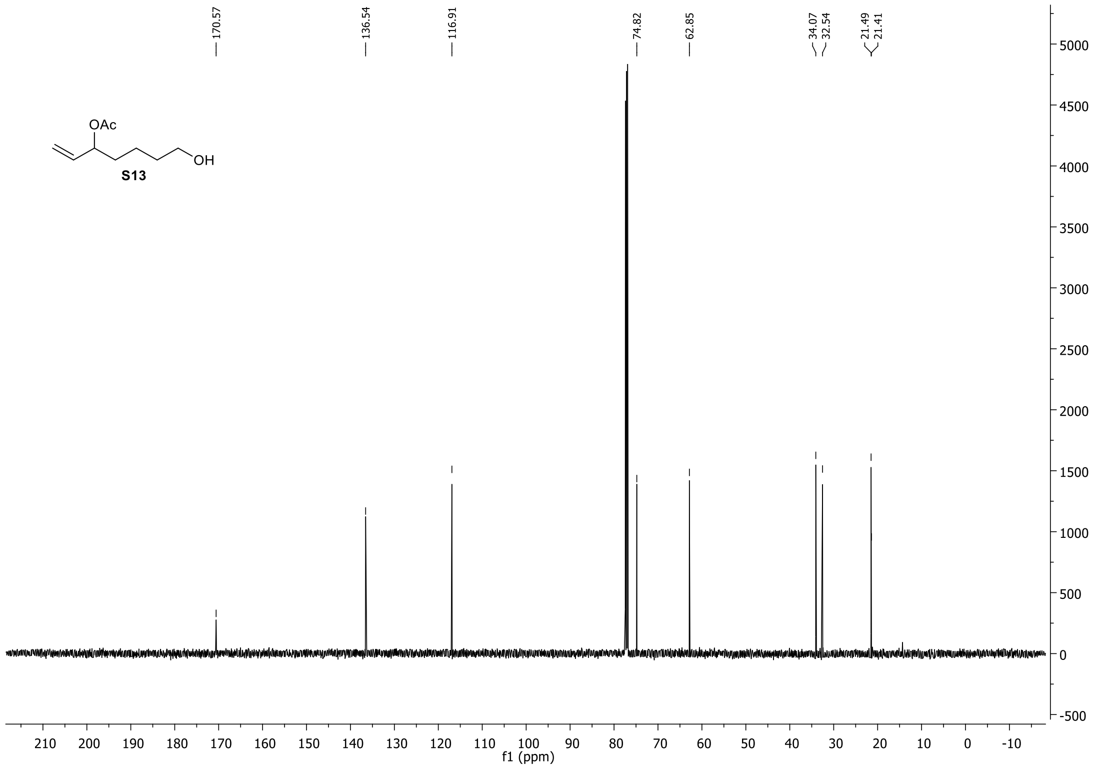

Supplementary Figure 28: ^{1}H NMR 12.

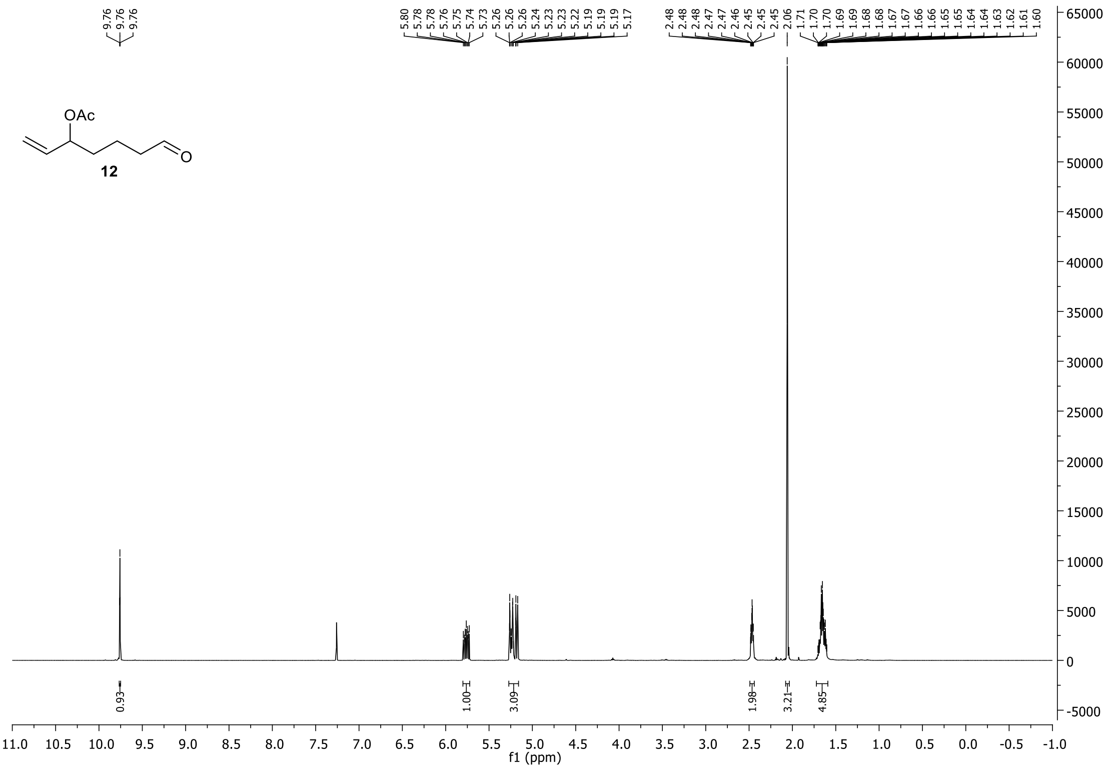

Supplementary Figure 29: ^{13}C NMR 12.

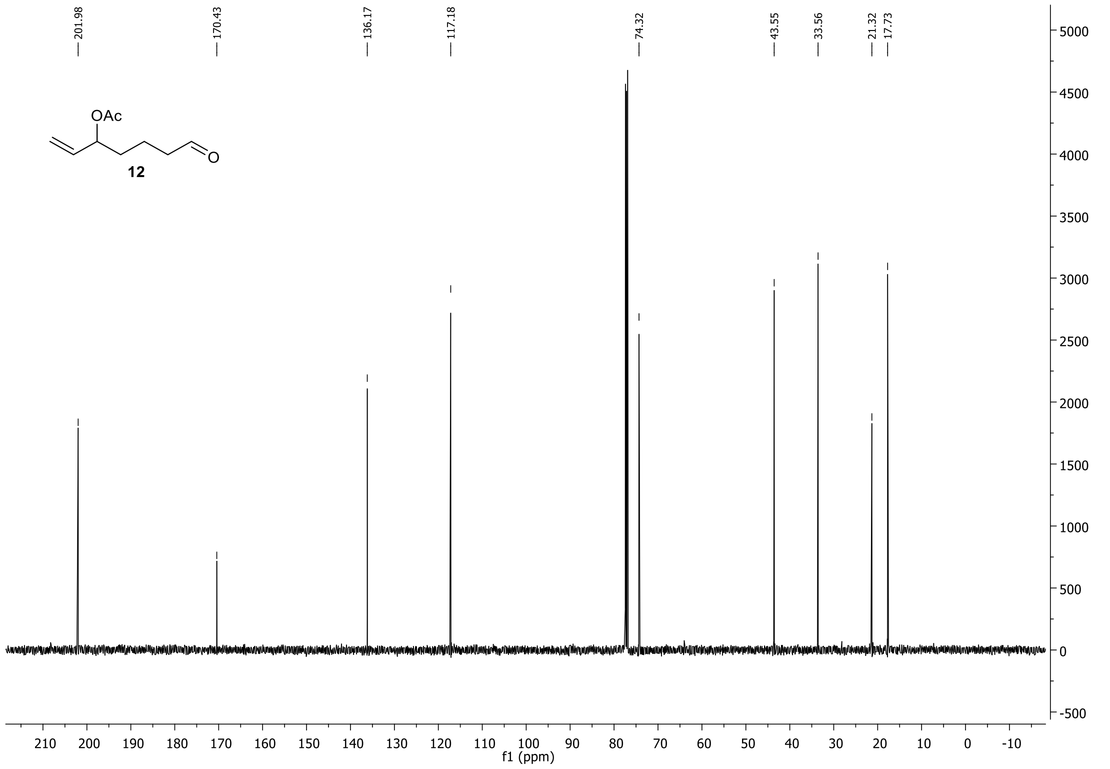

Supplementary Figure 30: ^{1}H NMR *syn*-S14.

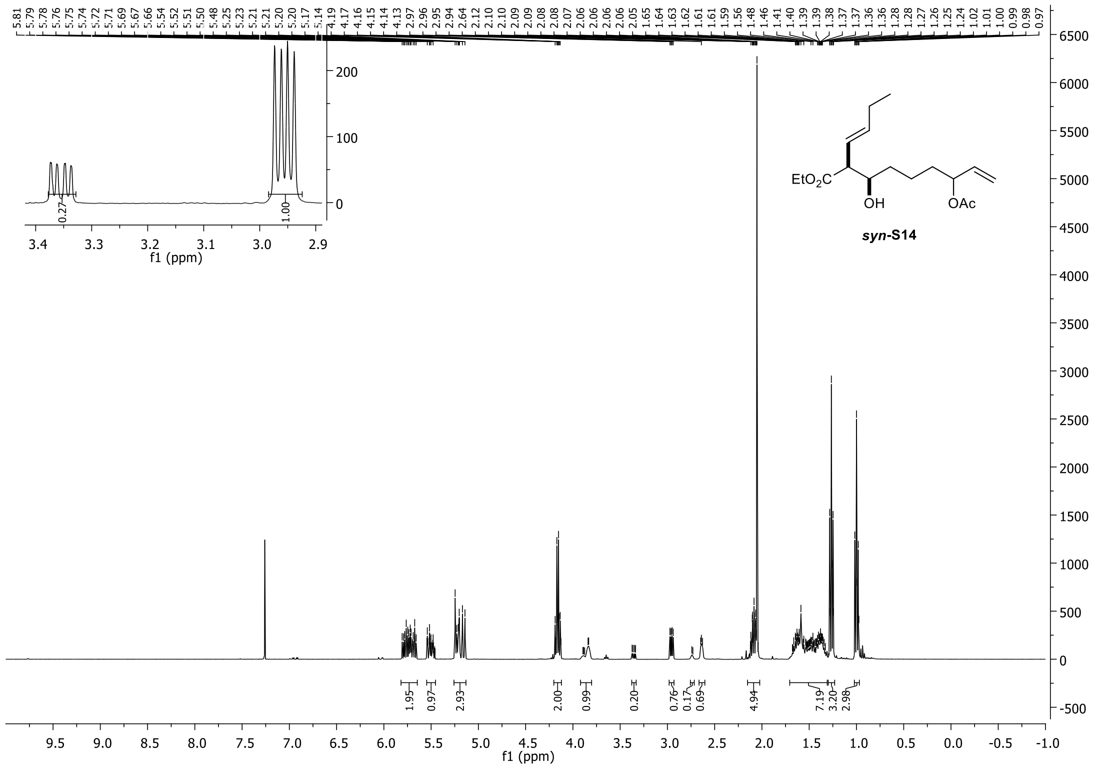

Supplementary Figure 31 ^{13}C NMR *syn*-S14.

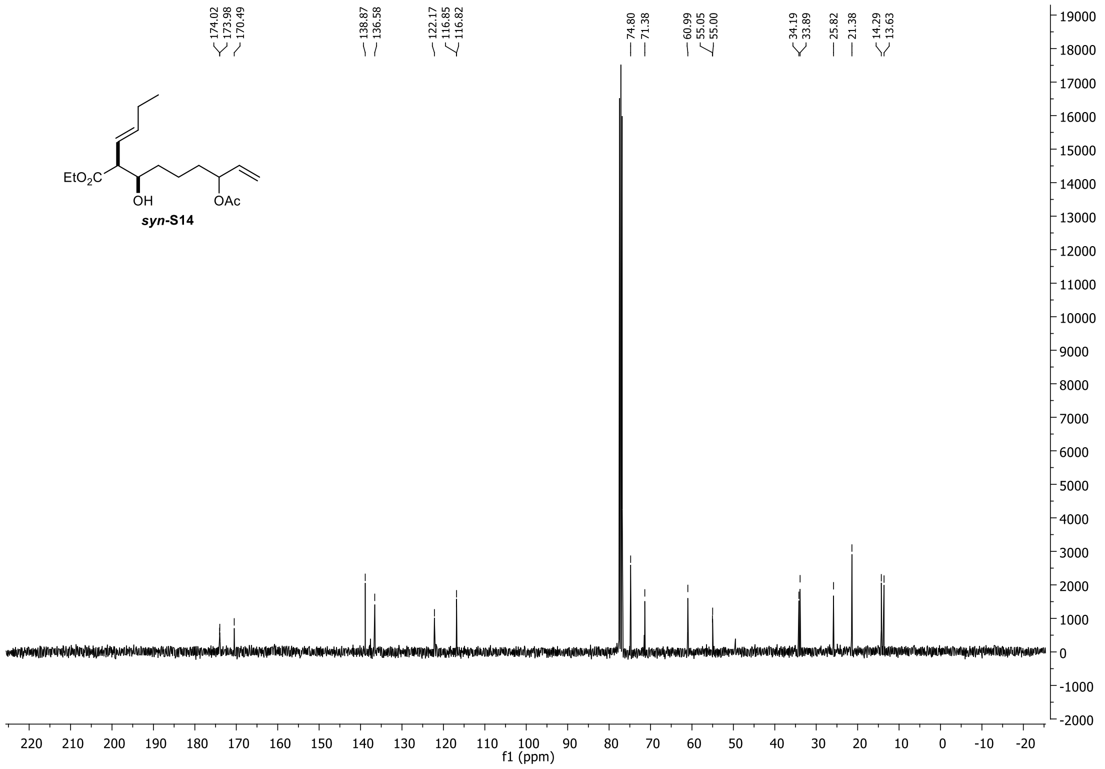

Supplementary Figure 32: ^{1}H NMR S15.

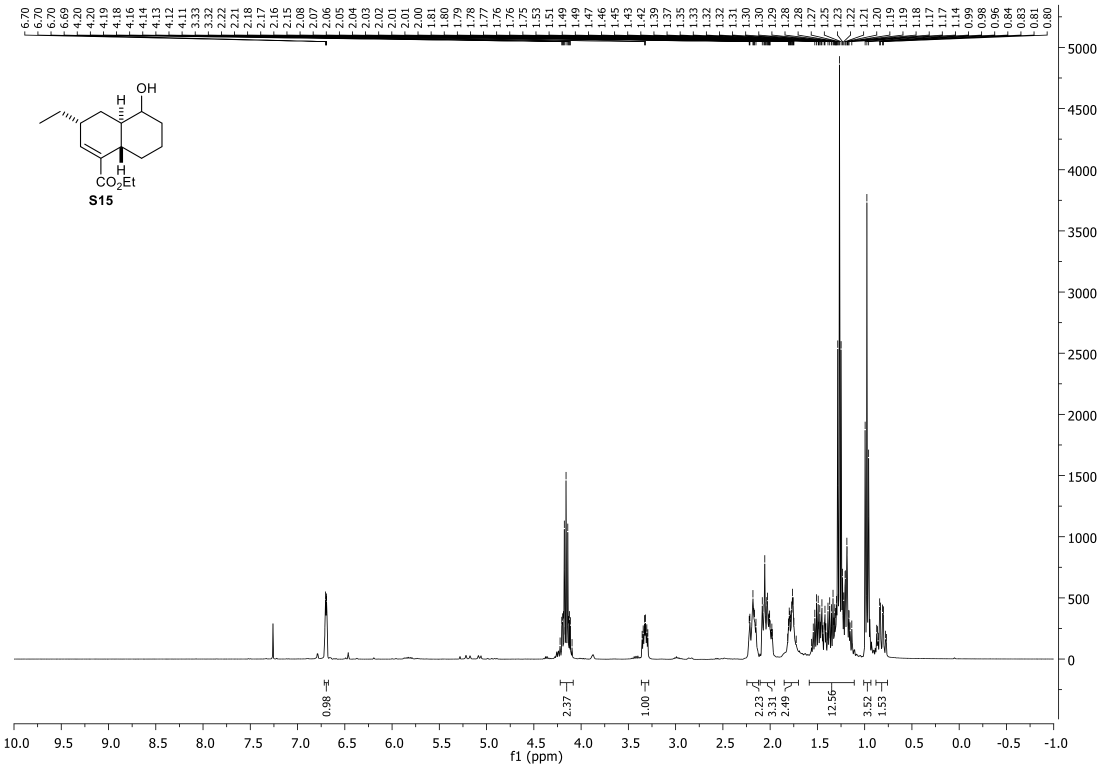

Supplementary Figure 33: ^{13}C NMR S15.

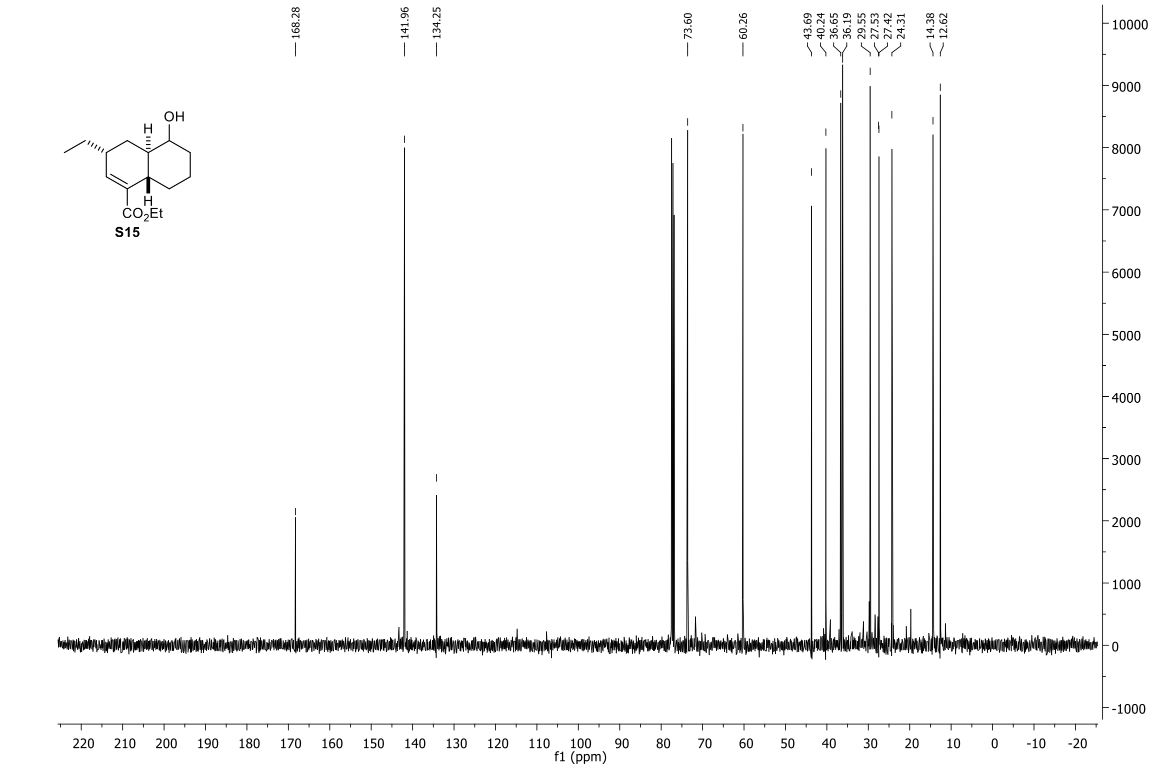

Supplementary Figure 34: ^{1}H NMR S16.

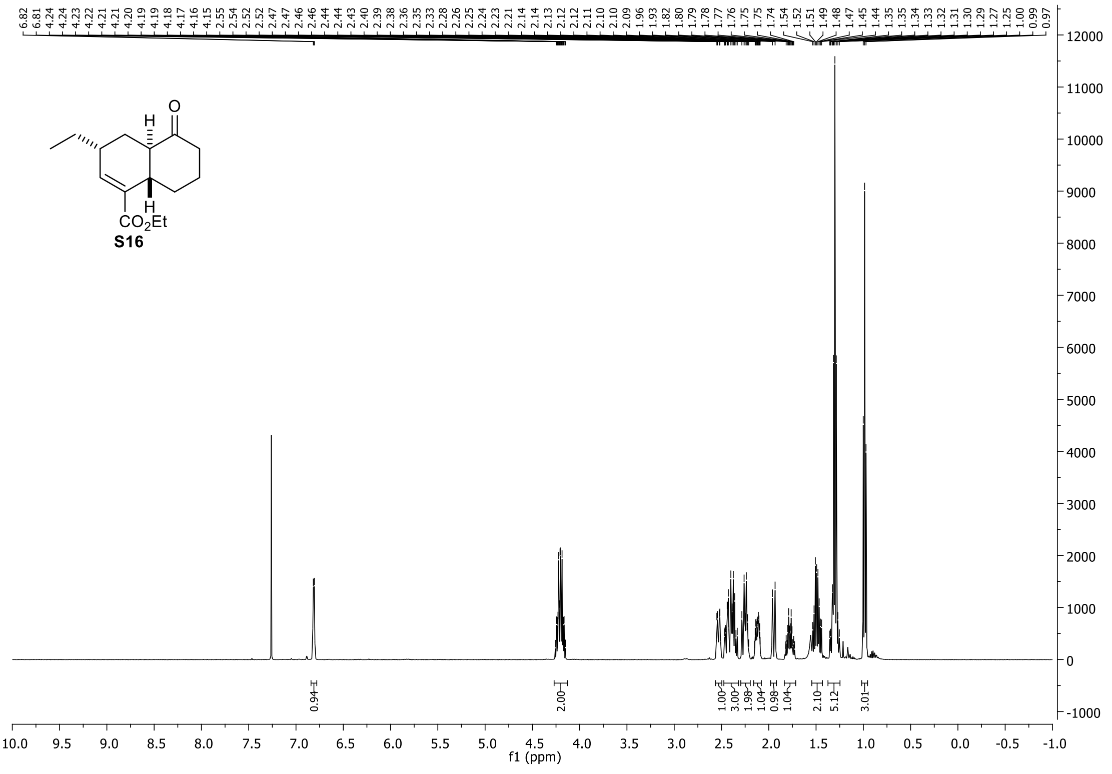

Supplementary Figure 35: ^{13}C NMR S16.

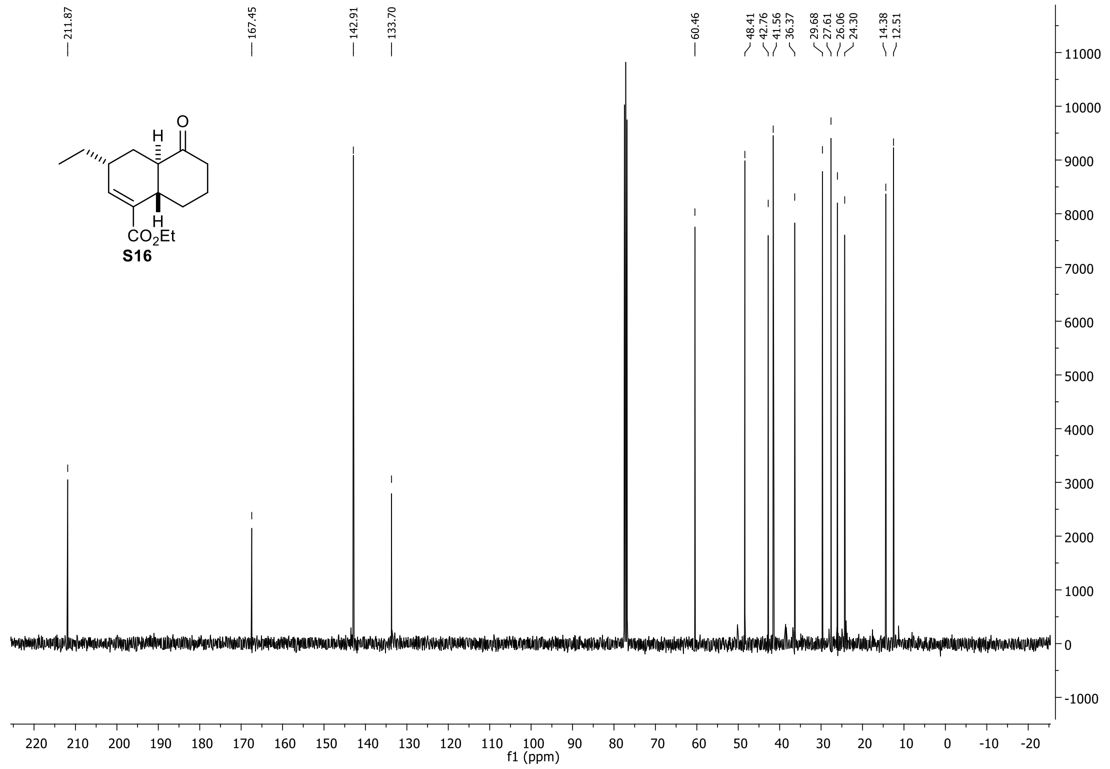

Supplementary Figure 36: ^{1}H NMR S17.

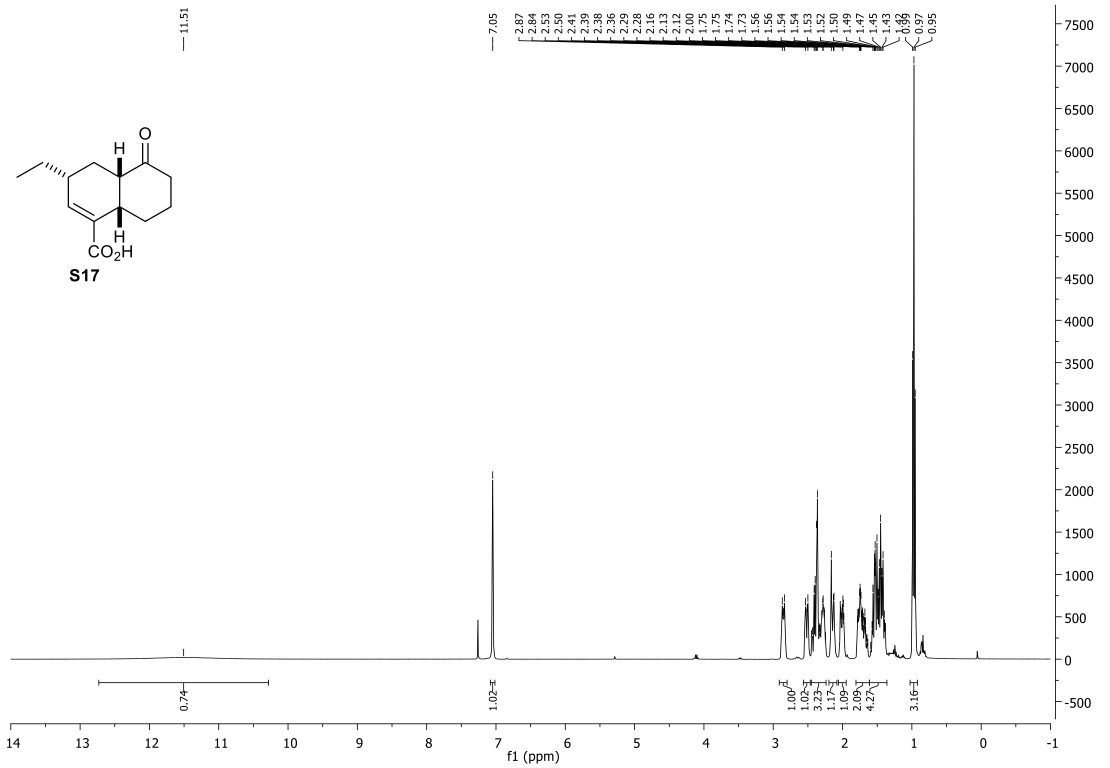

Supplementary Figure 37: ^{13}C NMR S17.

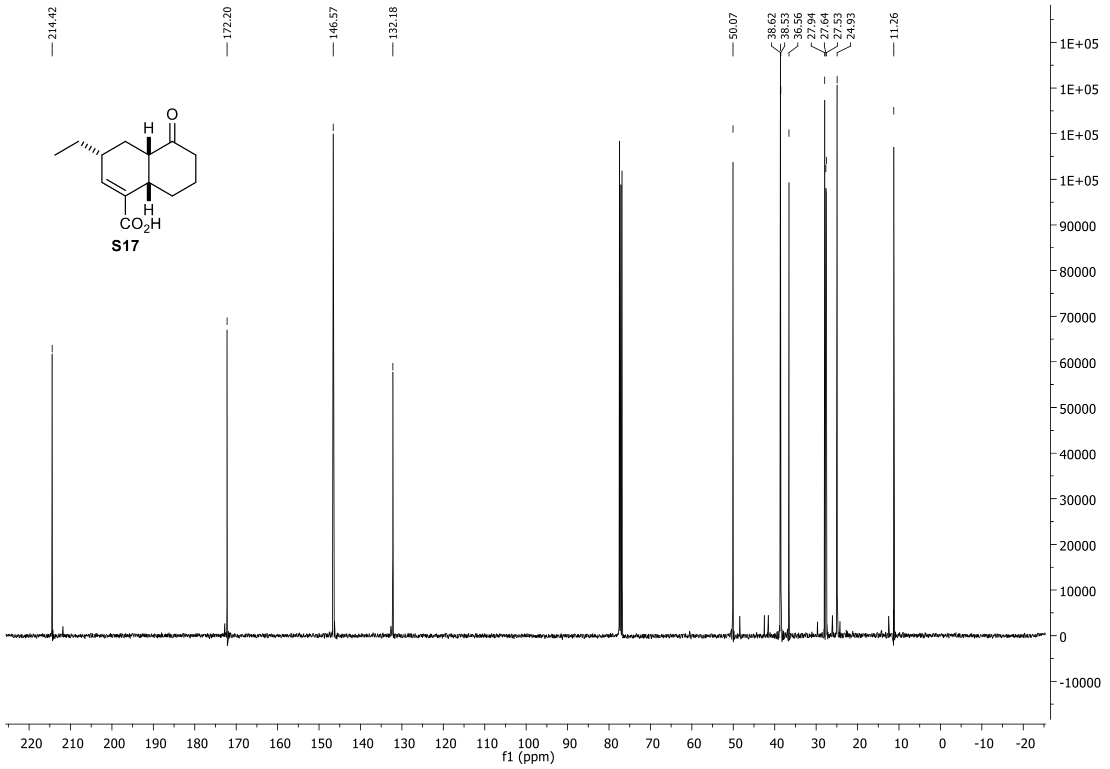

Supplementary Figure 38: ^{1}H NMR S19.

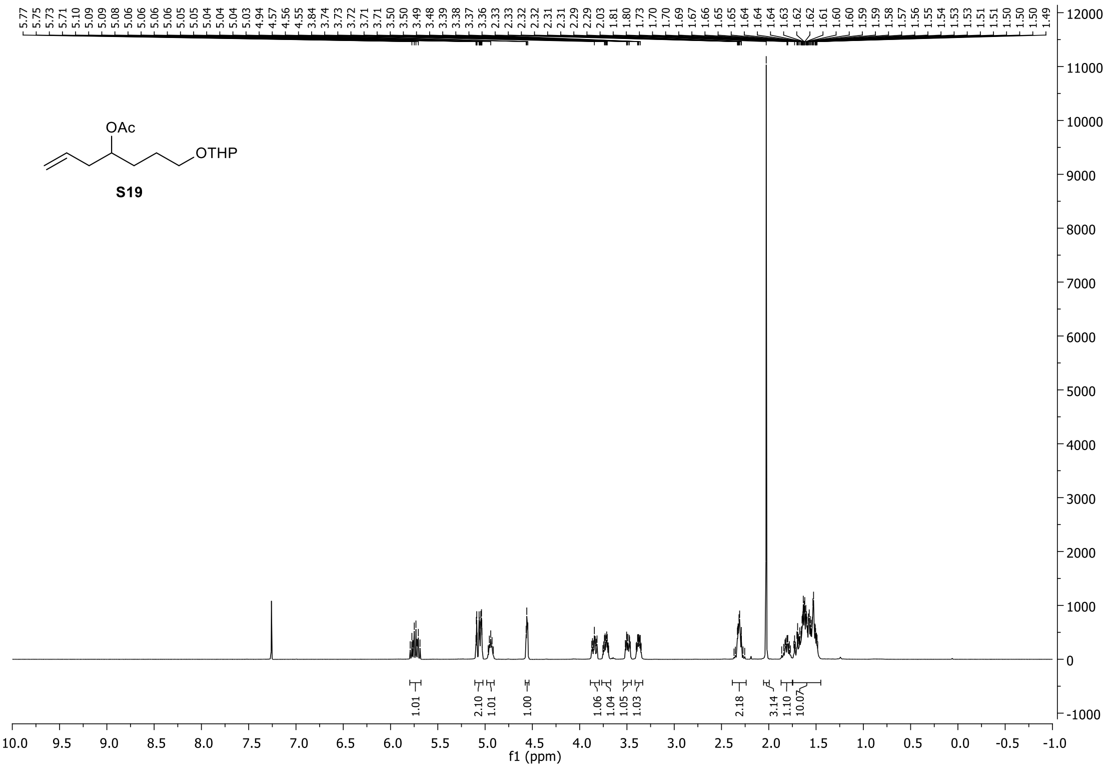

Supplementary Figure 39: ^{13}C NMR S19.

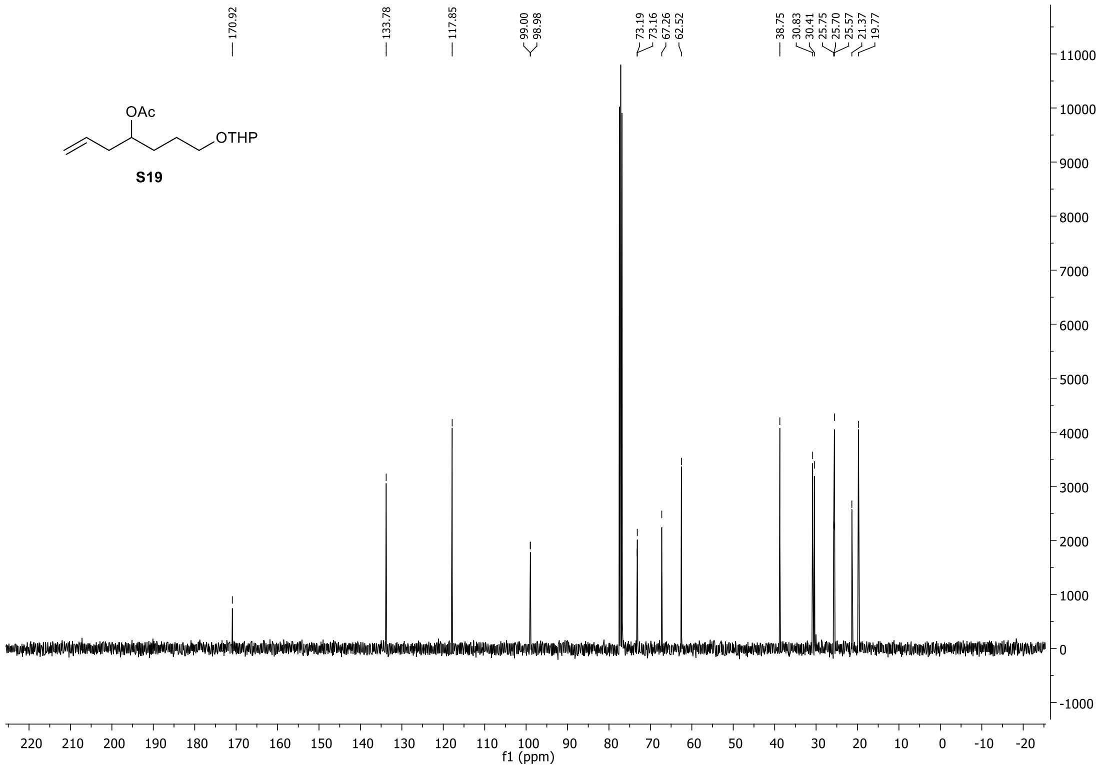

Supplementary Figure 40: ^{1}H NMR S20.

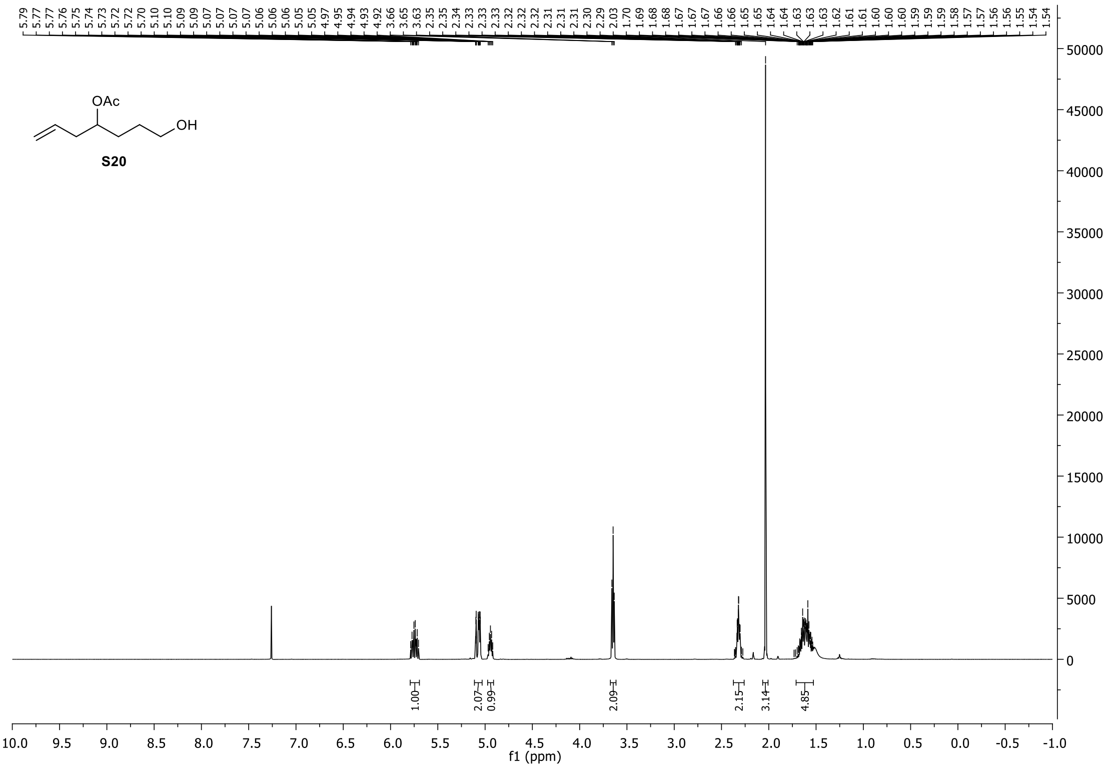

Supplementary Figure 41: ^{13}C NMR S20.

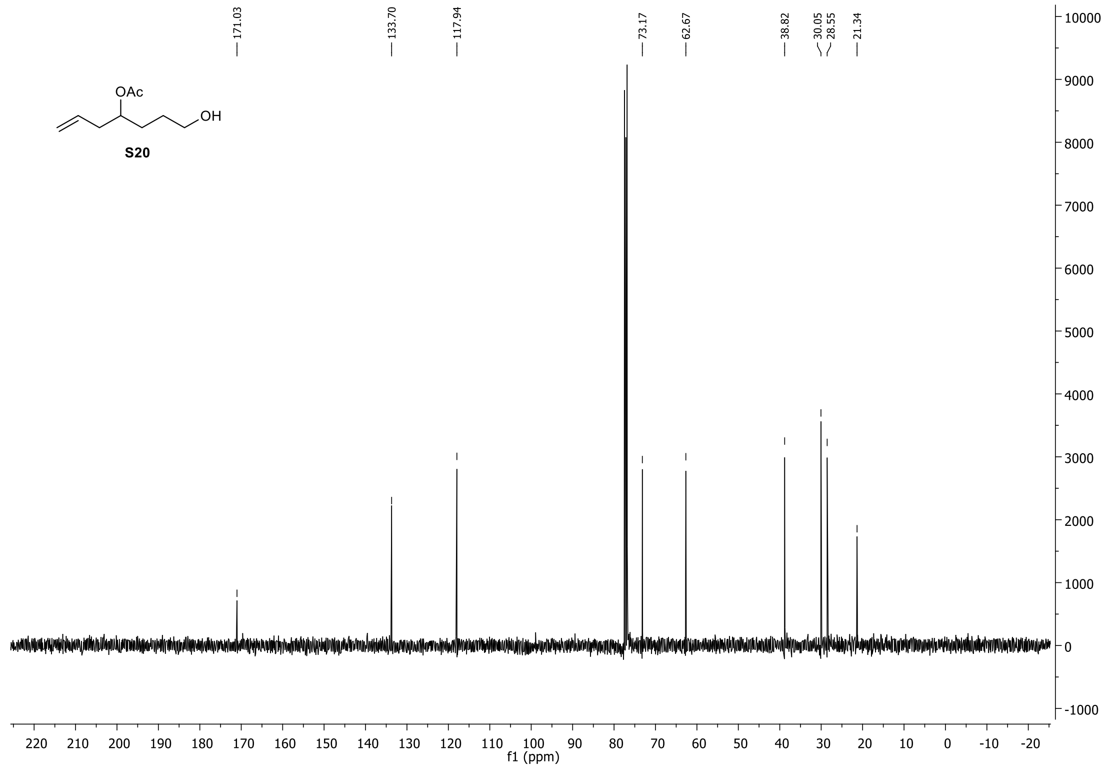

Supplementary Figure 42: ^{1}H NMR 13.

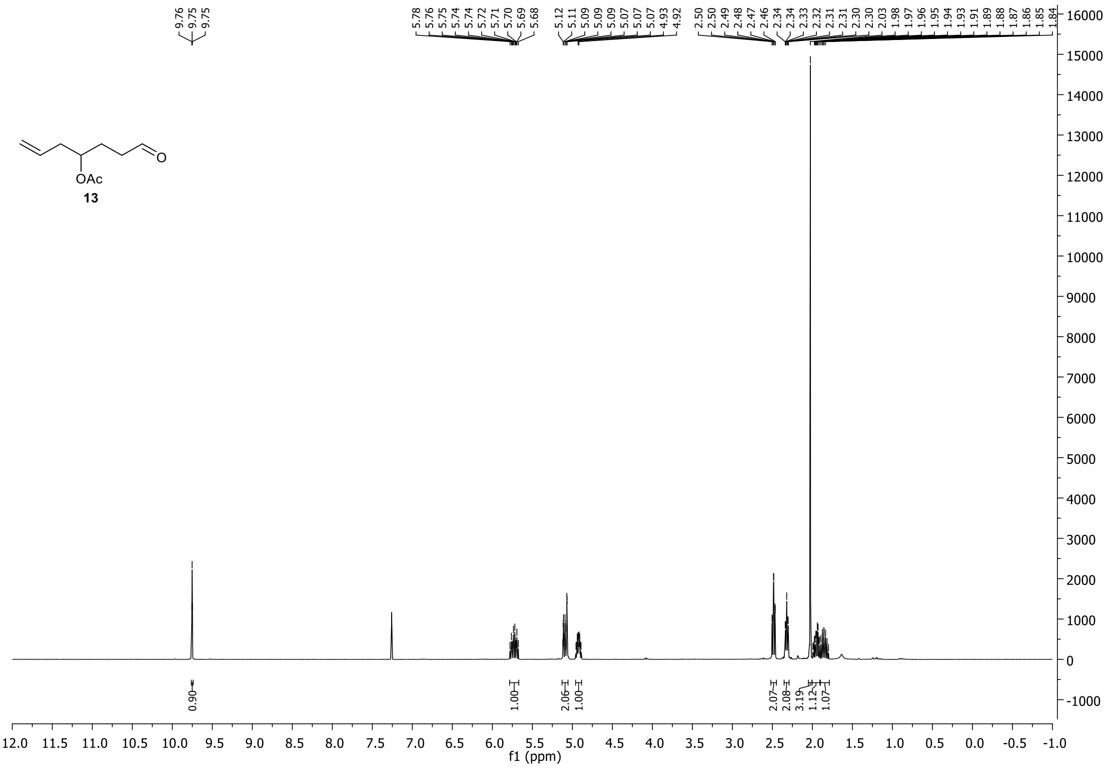

Supplementary Figure 43: ^{13}C NMR 13.

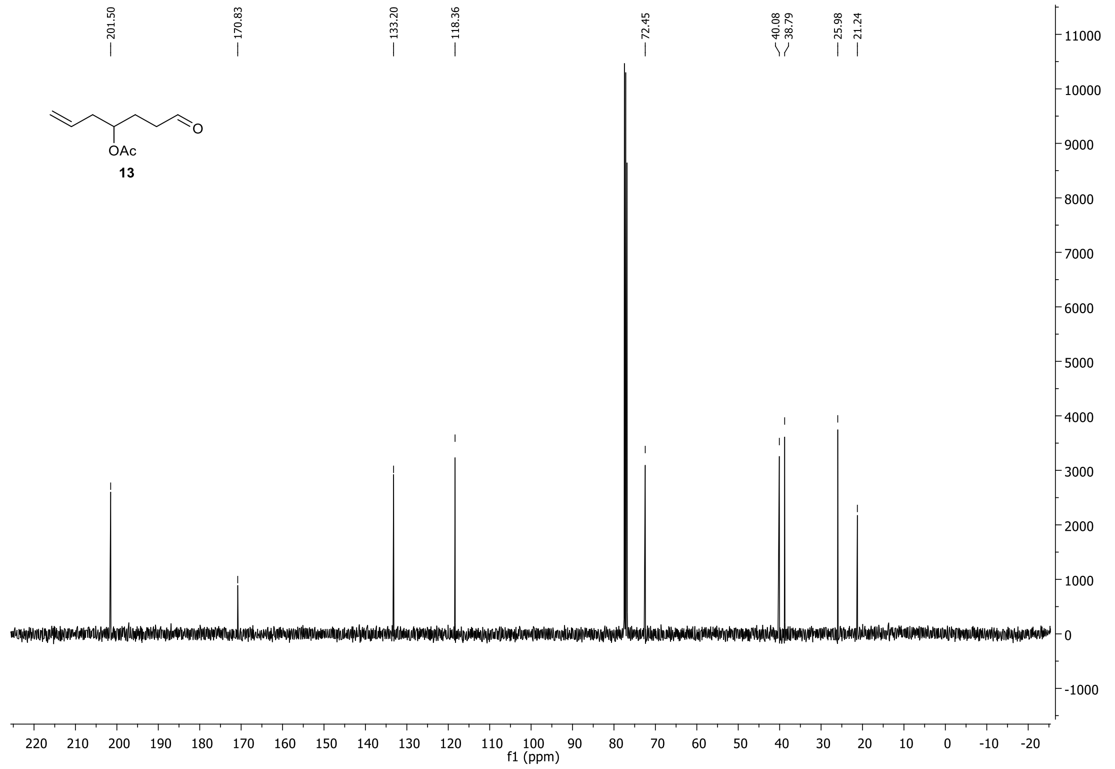

Supplementary Figure 44: ^{1}H NMR *syn*-S21.

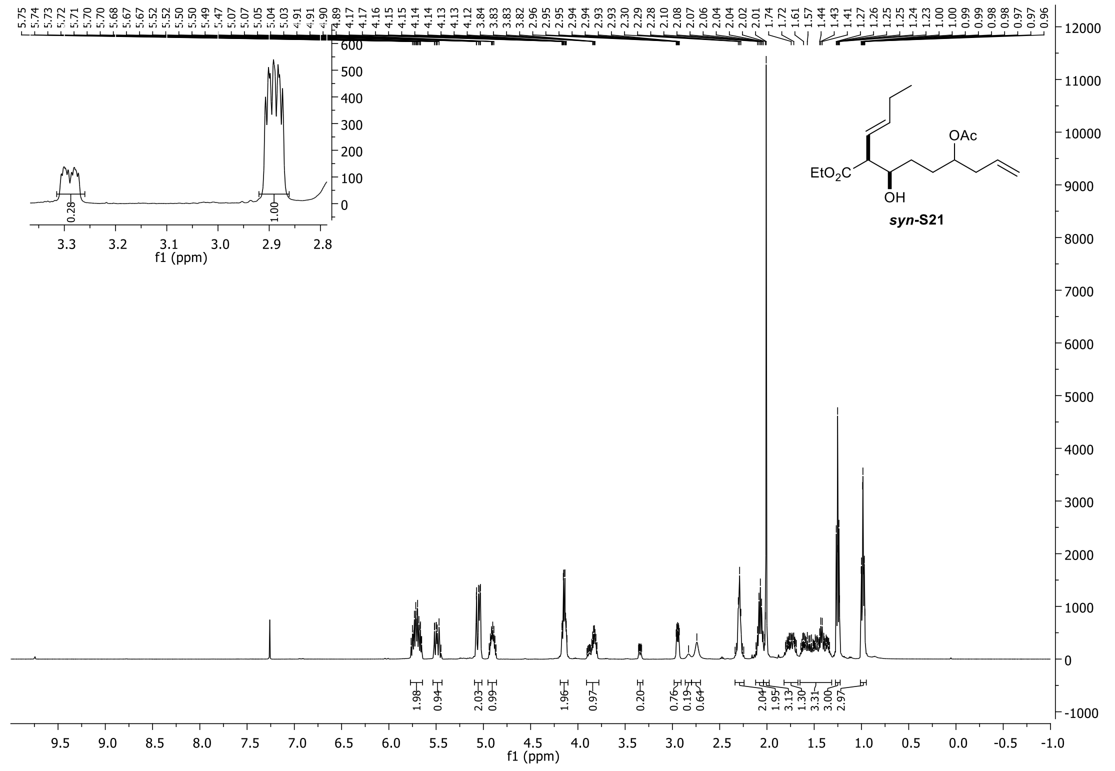

Supplementary Figure 45: ^{13}C NMR *syn*-S21.

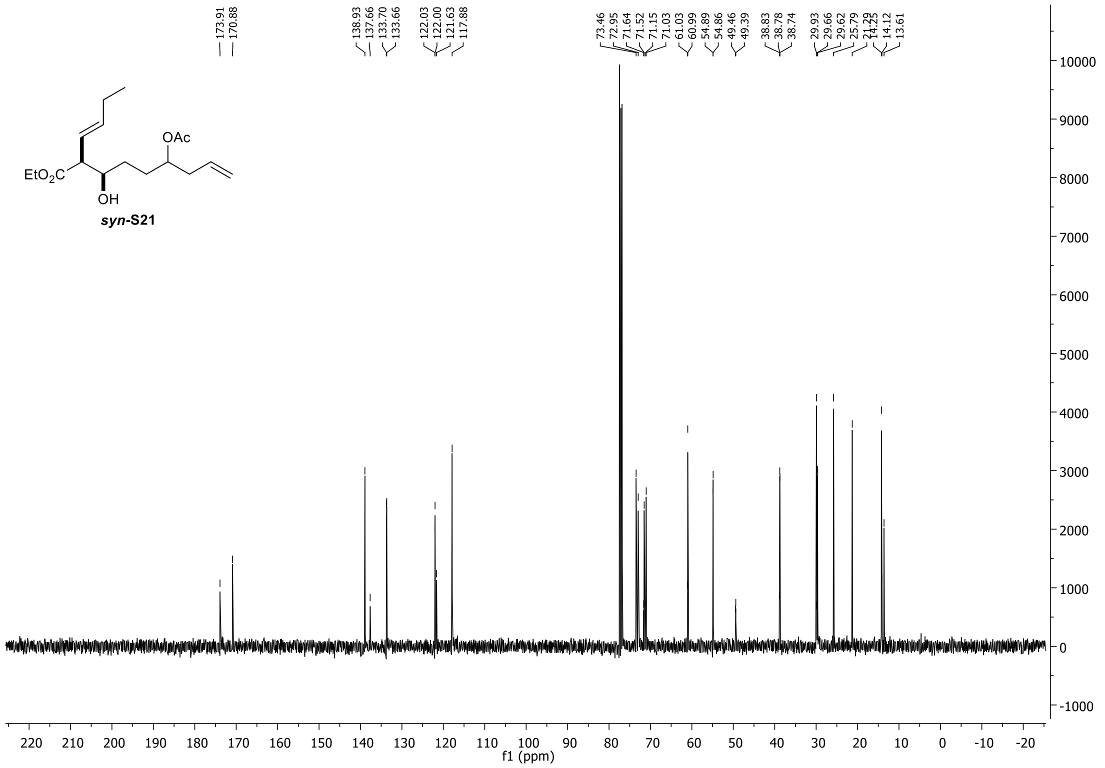

^{1}H NMR S22.
Supplementary Figure 46: Isomer 1:

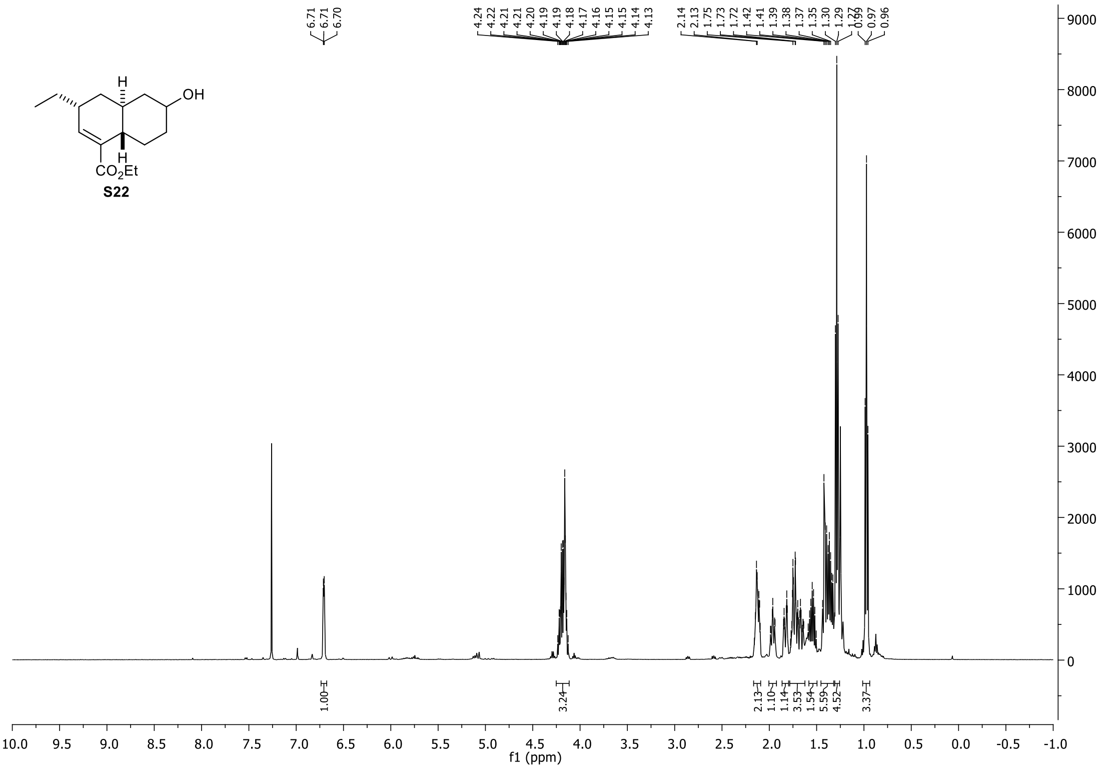

Supplementary Figure 47: Isomer 2:

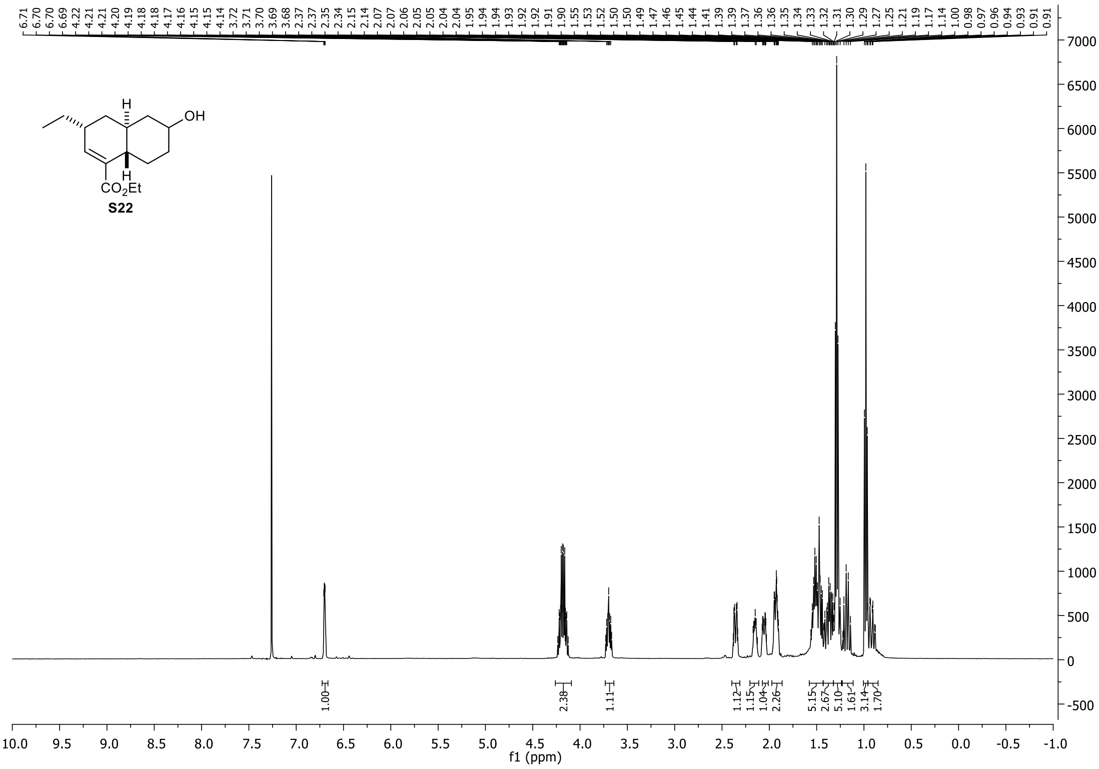

^{13}C NMR S22.
Supplementary Figure 48: Isomer 1:

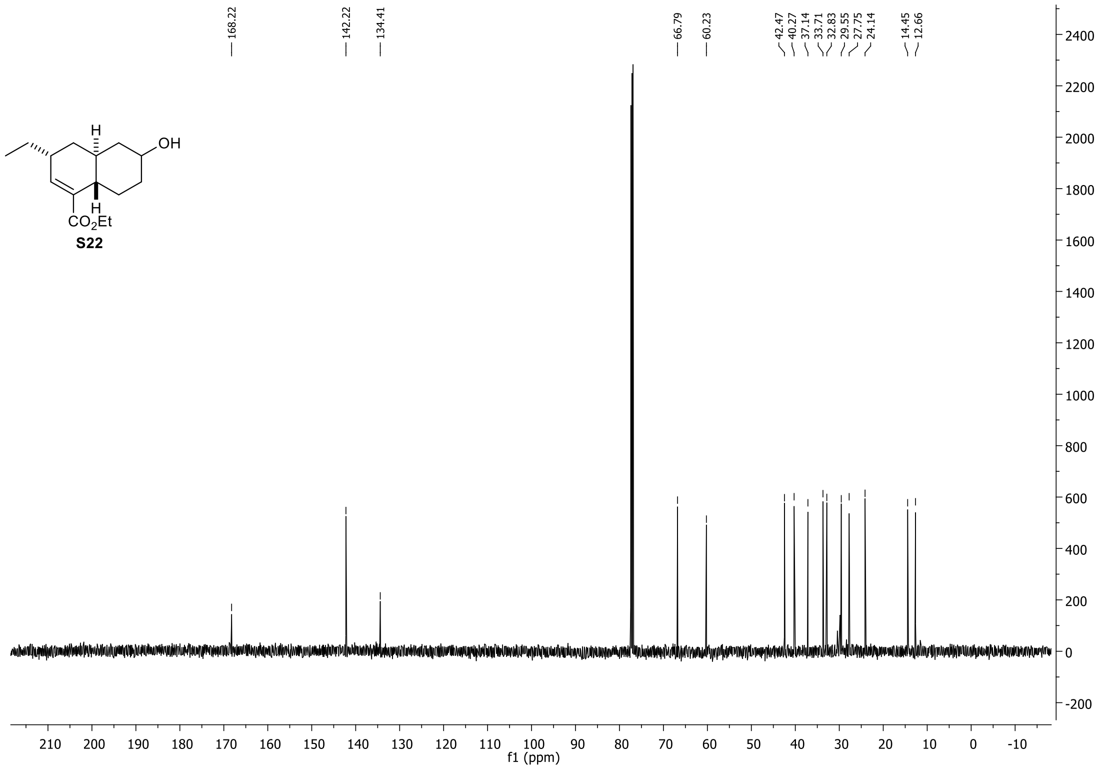

Supplementary Figure 49: Isomer 2:

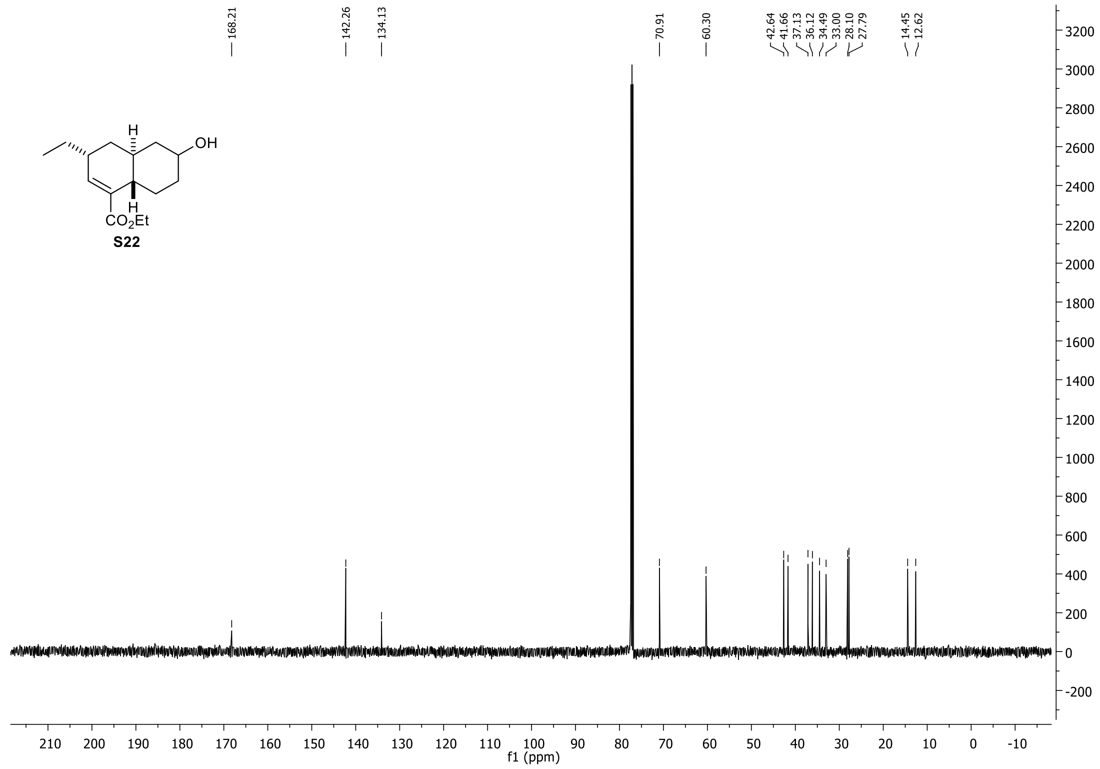

Supplementary Figure 50: ^{1}H NMR S23.

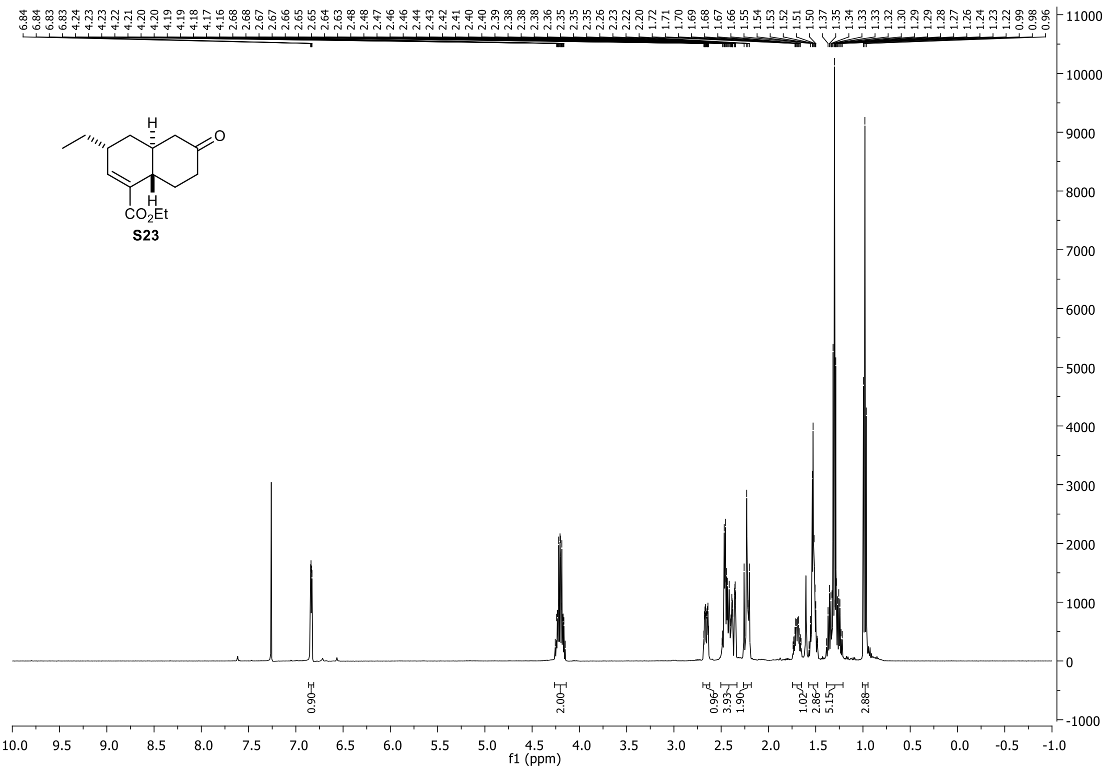

Supplementary Figure 51: ^{13}C NMR S23.

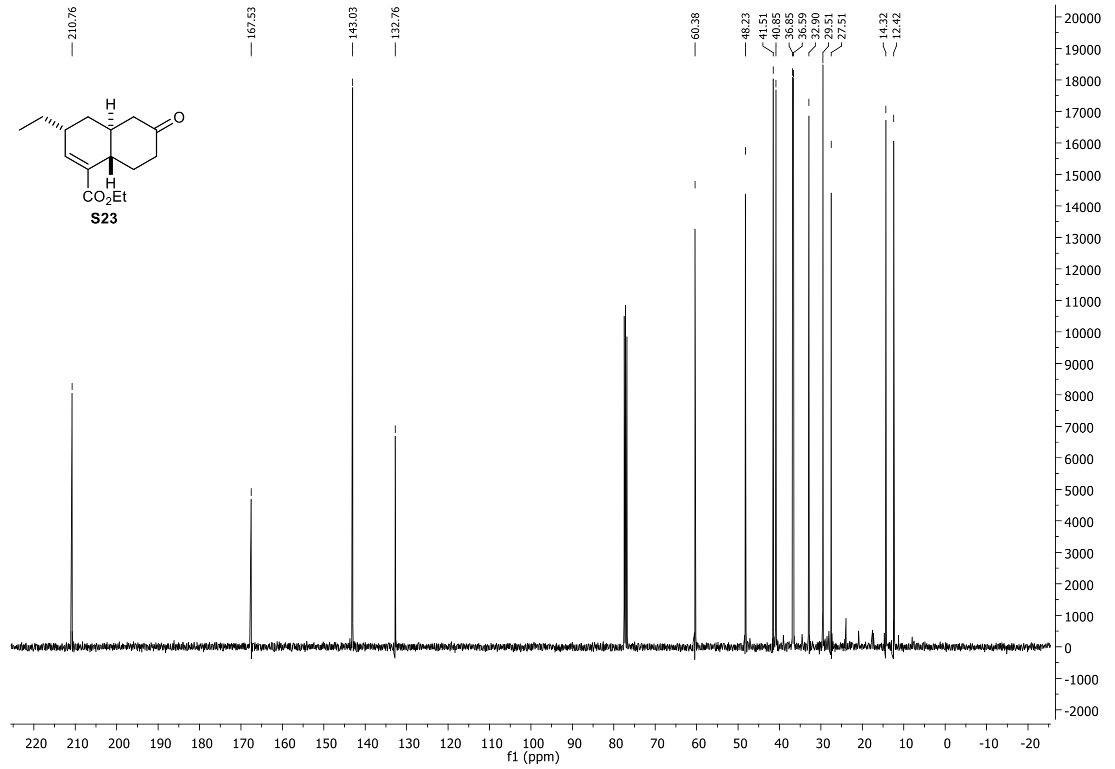

Supplementary Figure 52: ^{1}H NMR S24.

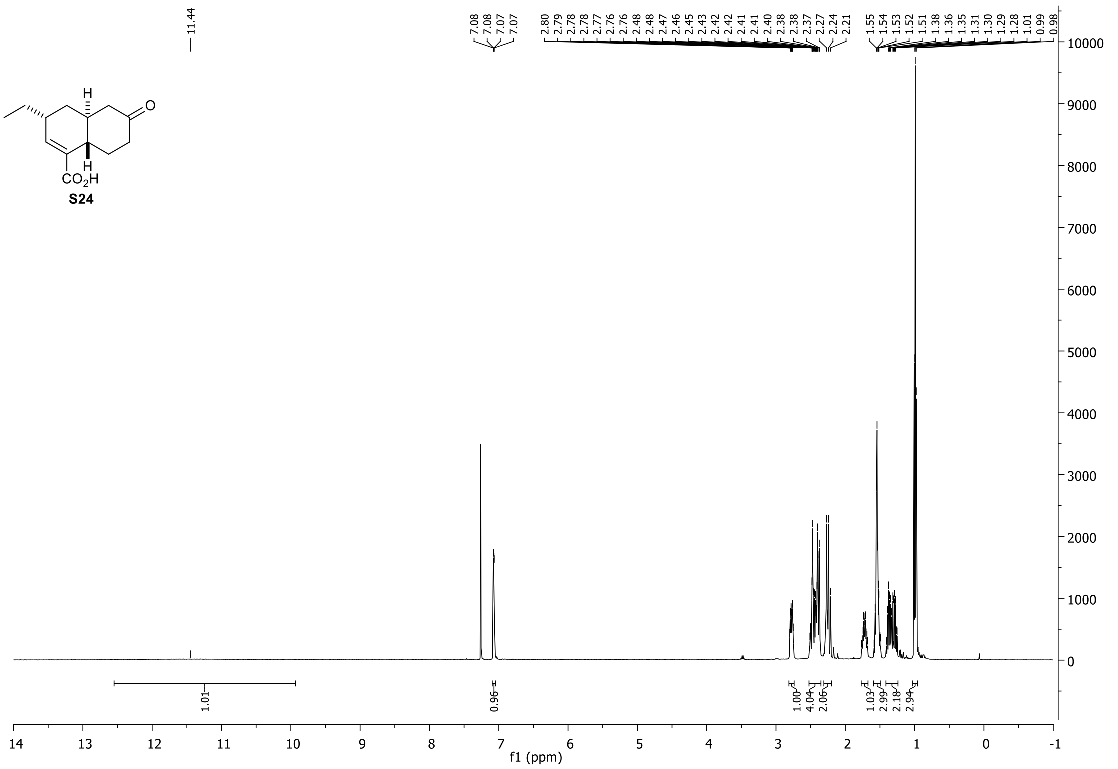

Supplementary Figure 53: ^{13}C NMR S24.

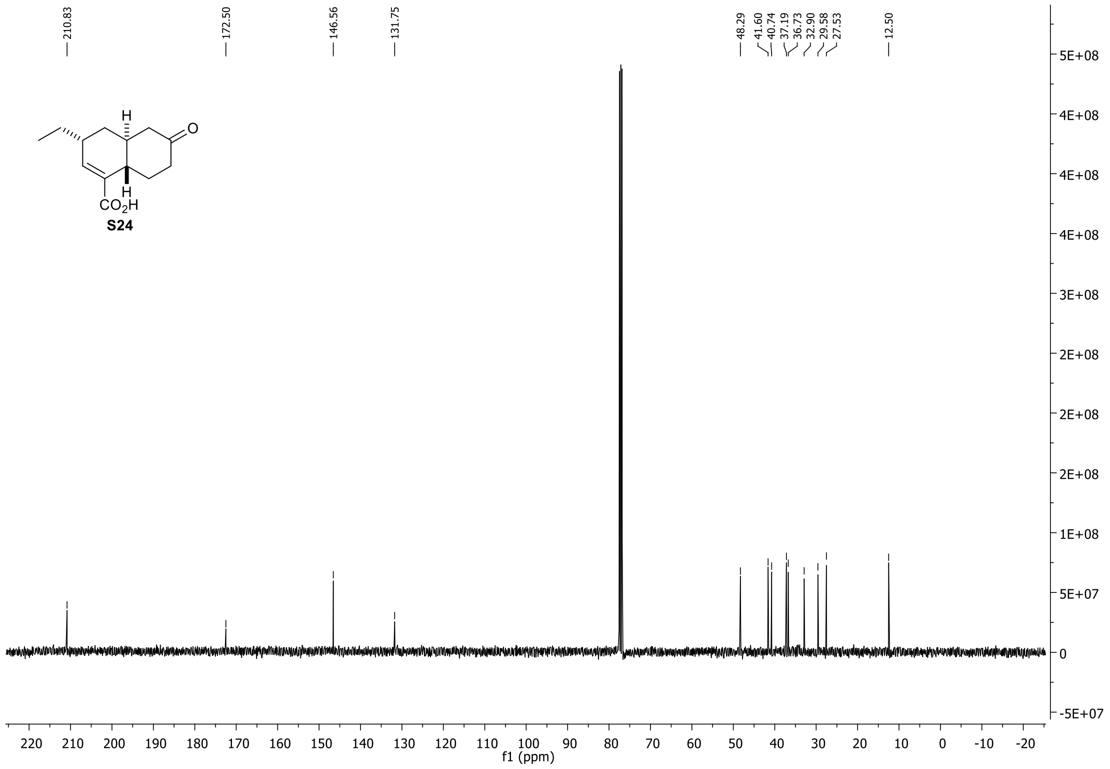

Supplementary Figure 54: ^{1}H NMR *syn*-S26.

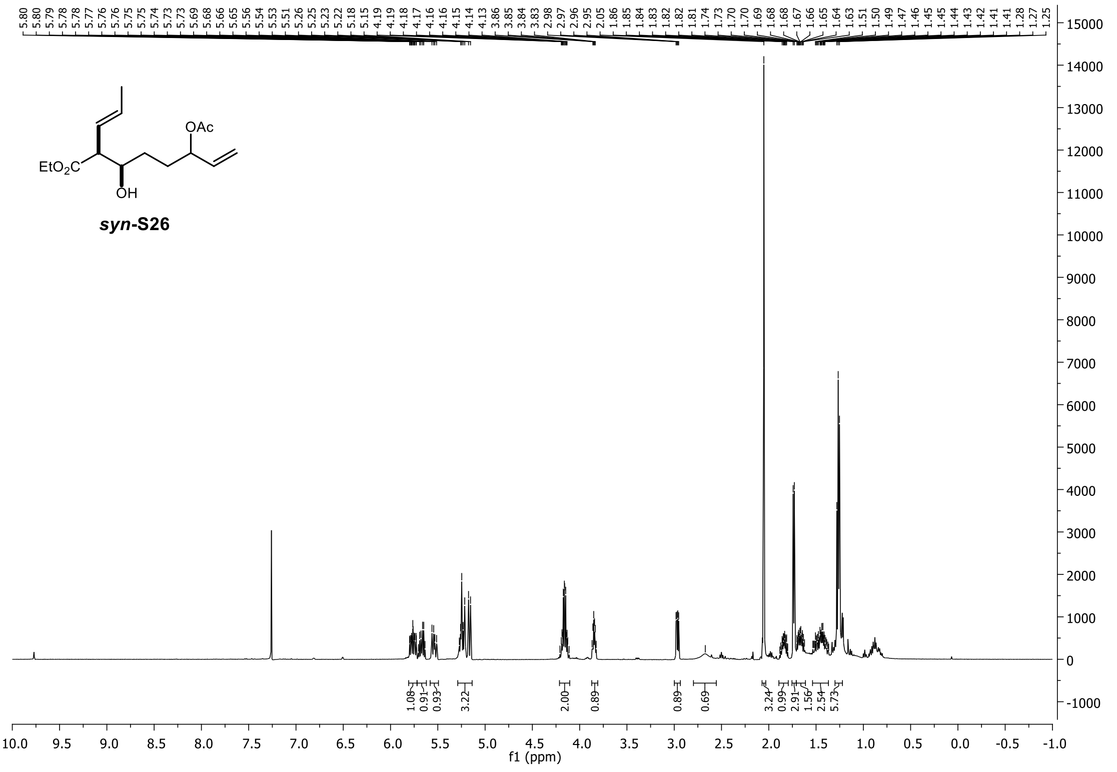

Supplementary Figure 55: ^{1}H NMR *syn*-S26 showing alkene isomerization.

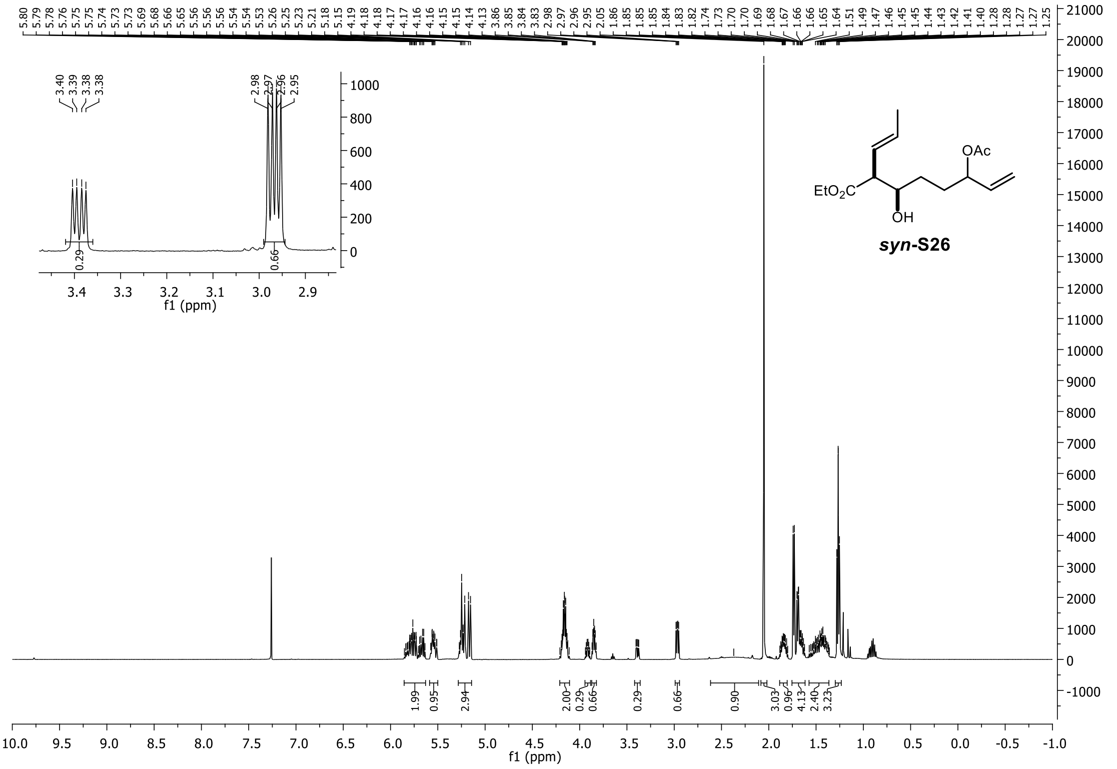

Supplementary Figure 56: ^{13}NMR *syn*-S26.

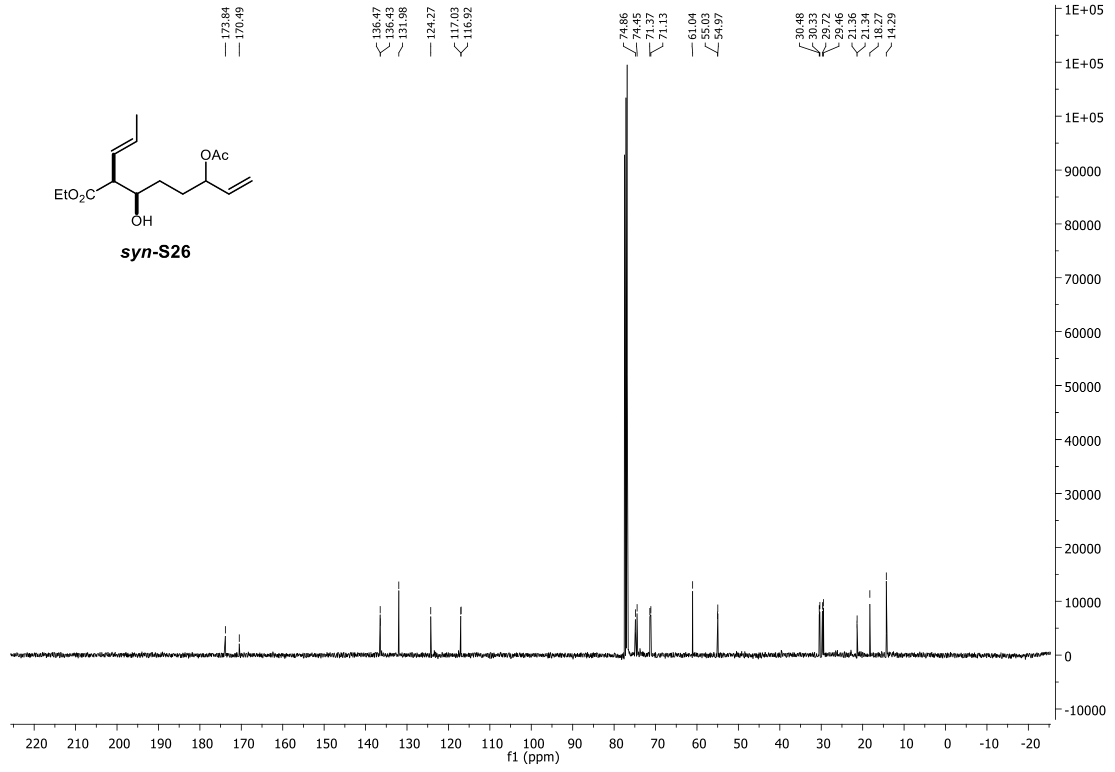

Supplementary Figure 57: ^{1}H NMR S29.

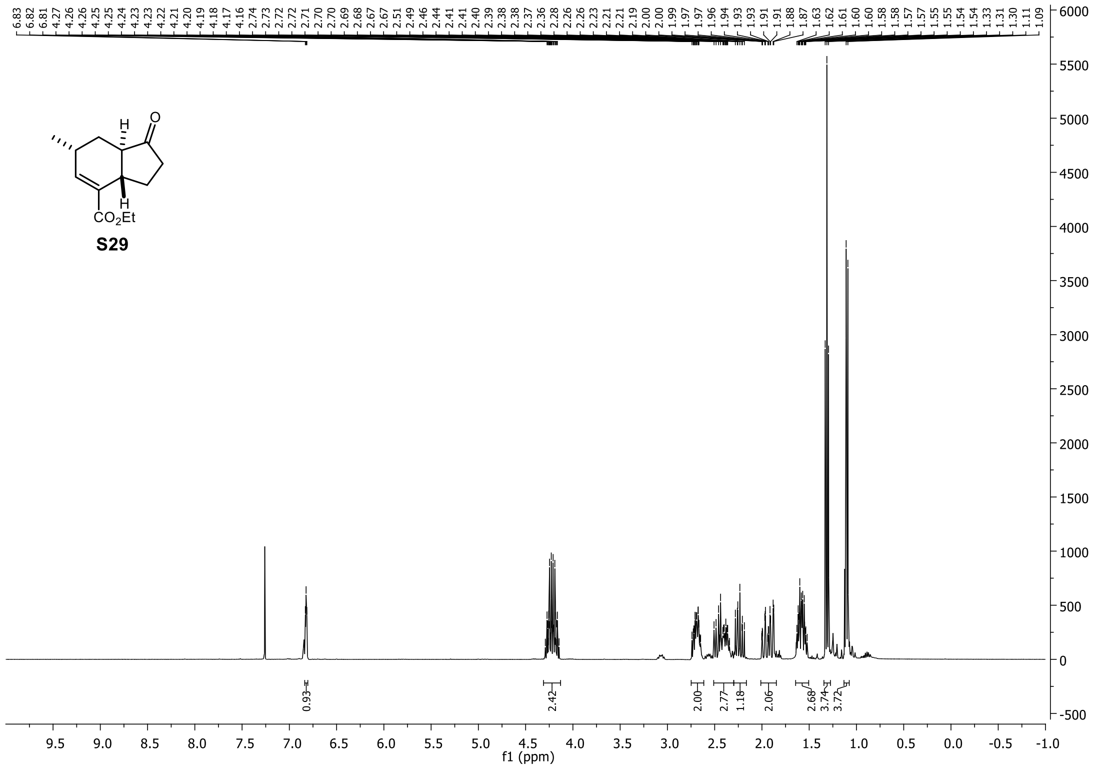

Supplementary Figure 58: ^{13}C NMR S29.

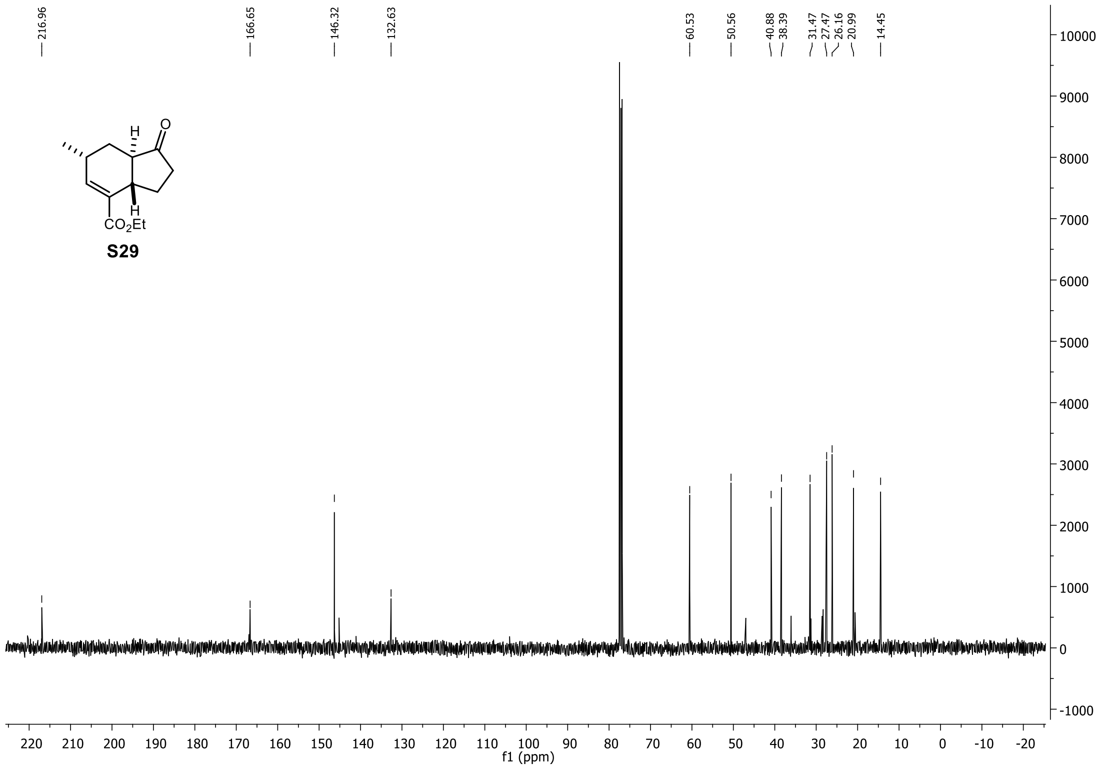

Supplementary Figure 59: ^{1}H NMR S30.

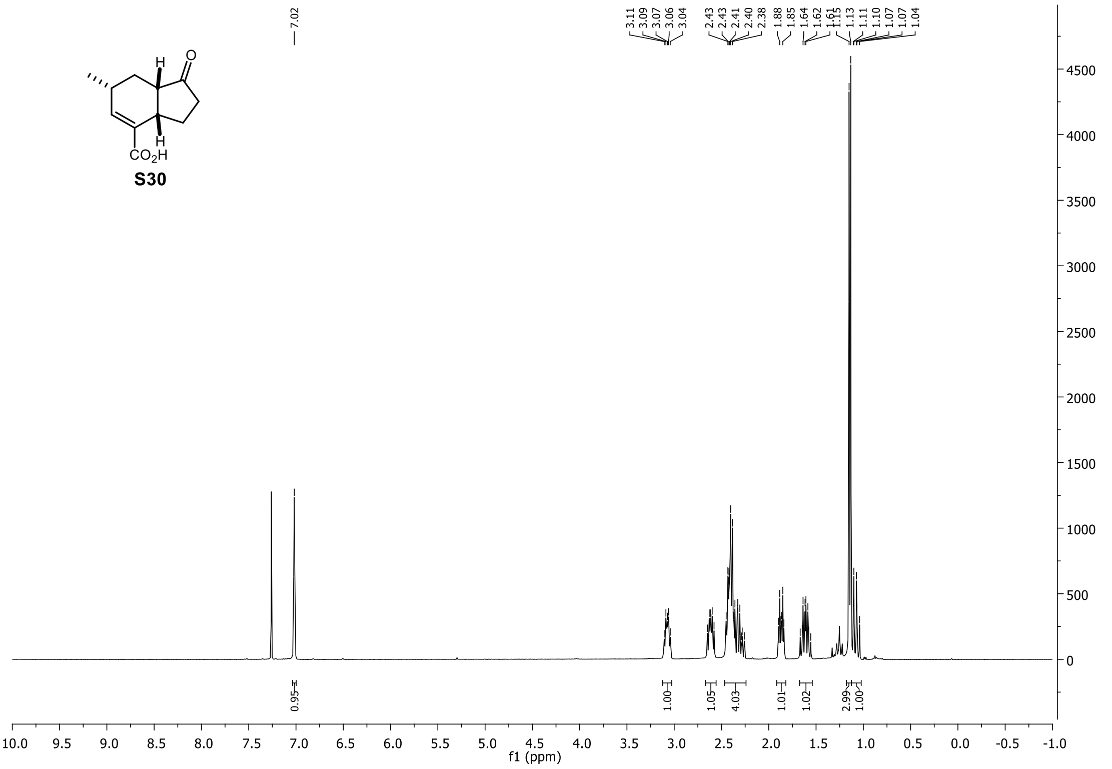

Supplementary Figure 60: ^{13}C NMR S30.

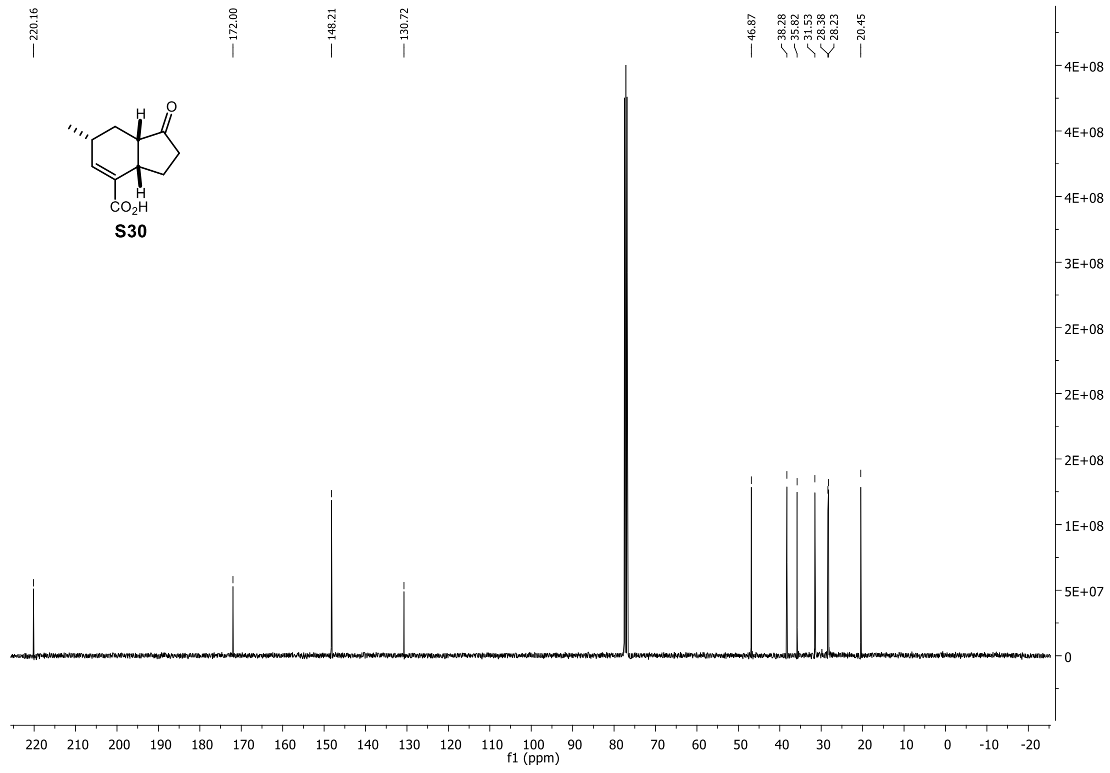

Supplementary Figure 61: ^{1}H NMR *syn*-S31.

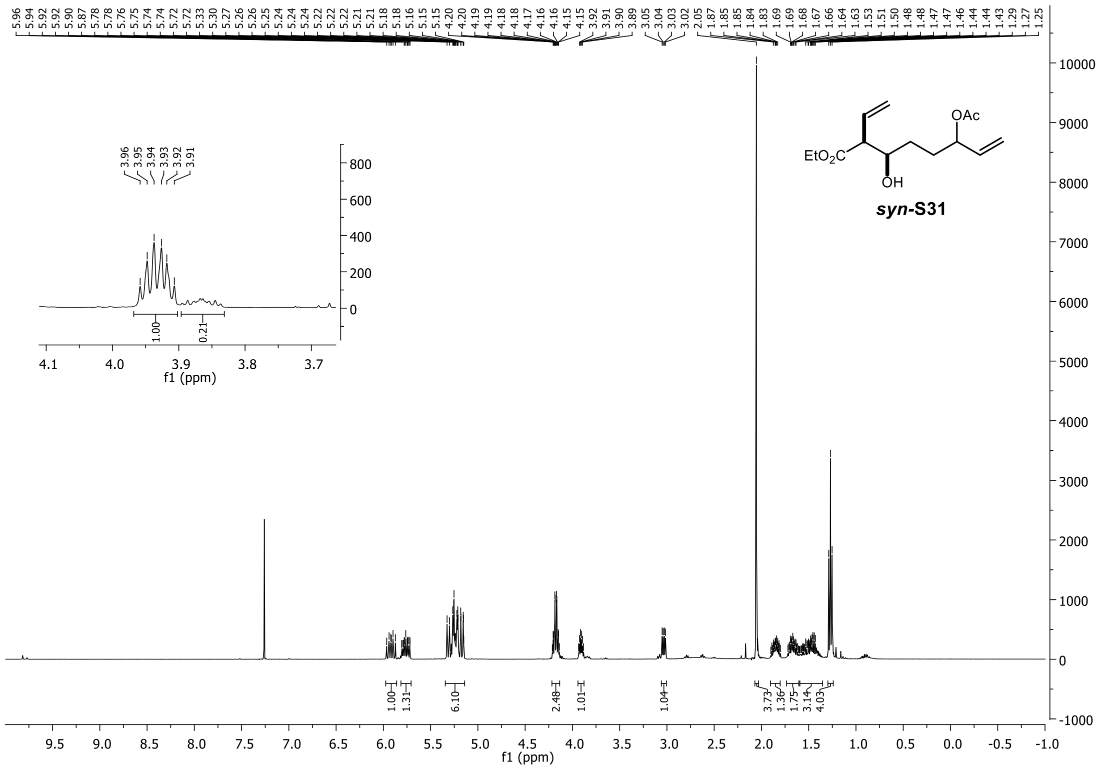

Supplementary Figure 62: ^{13}C NMR *syn*-S31.

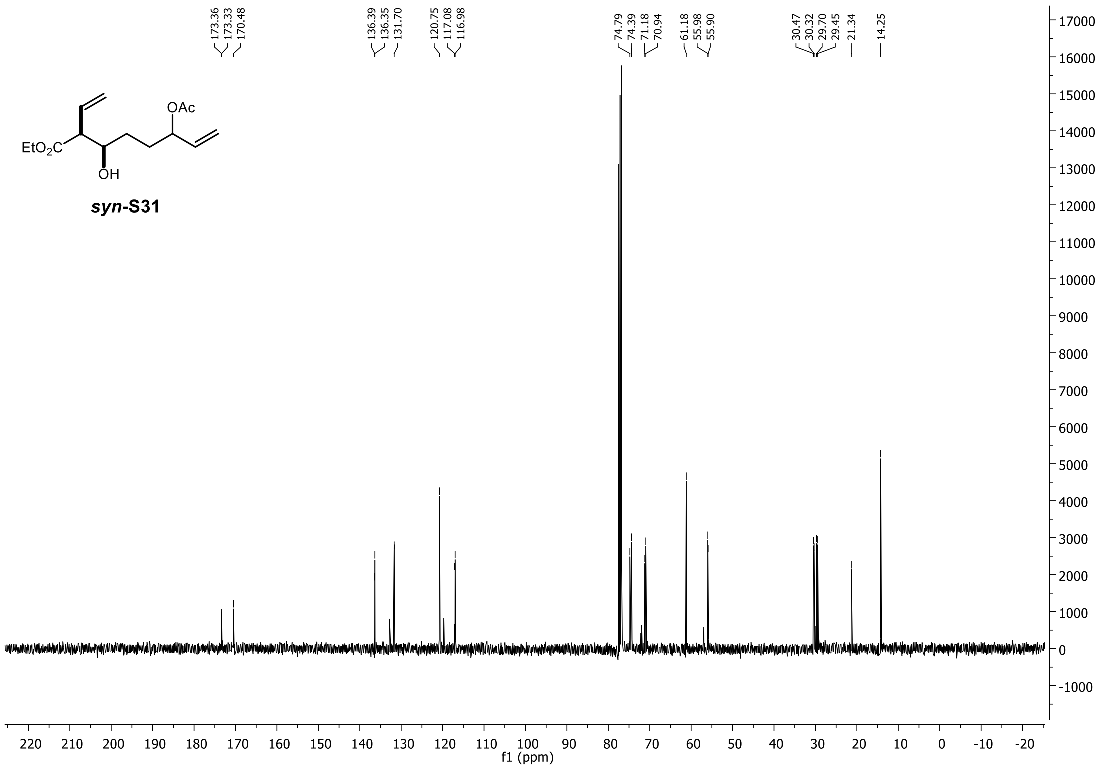

Supplementary Figure 63: ^{1}H NMR S34.

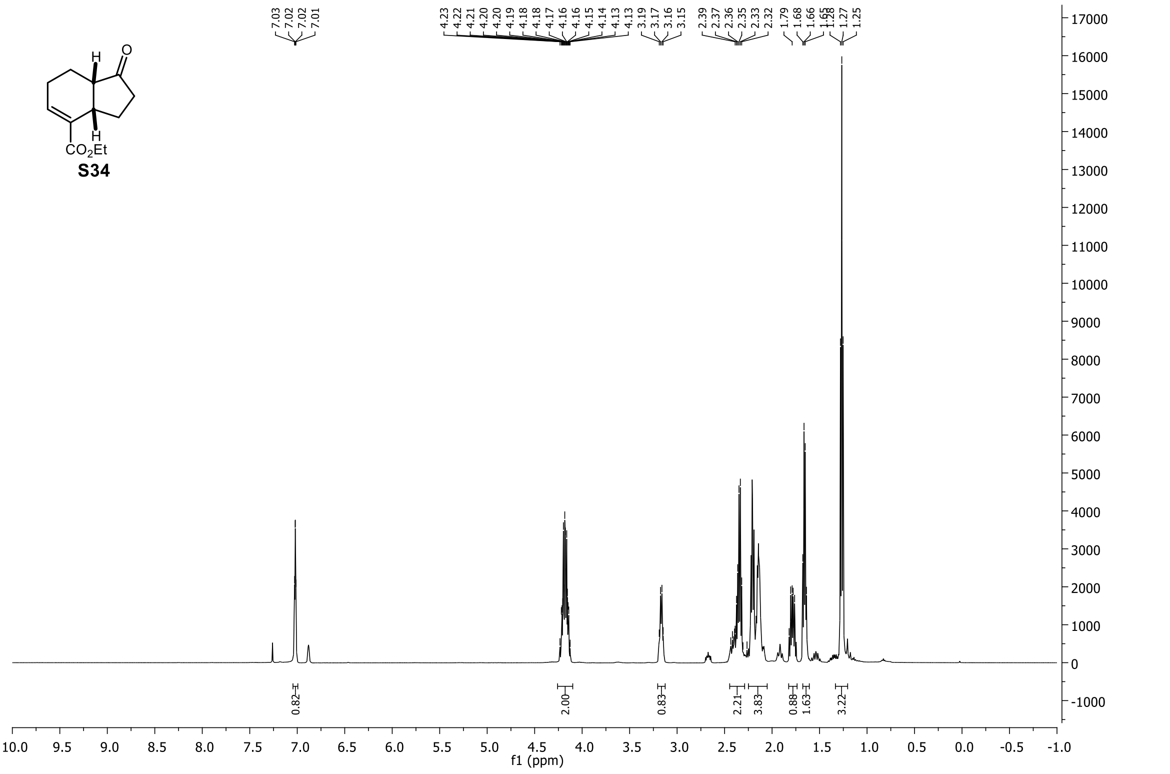

Supplementary Figure 64: ^{13}C NMR S34.

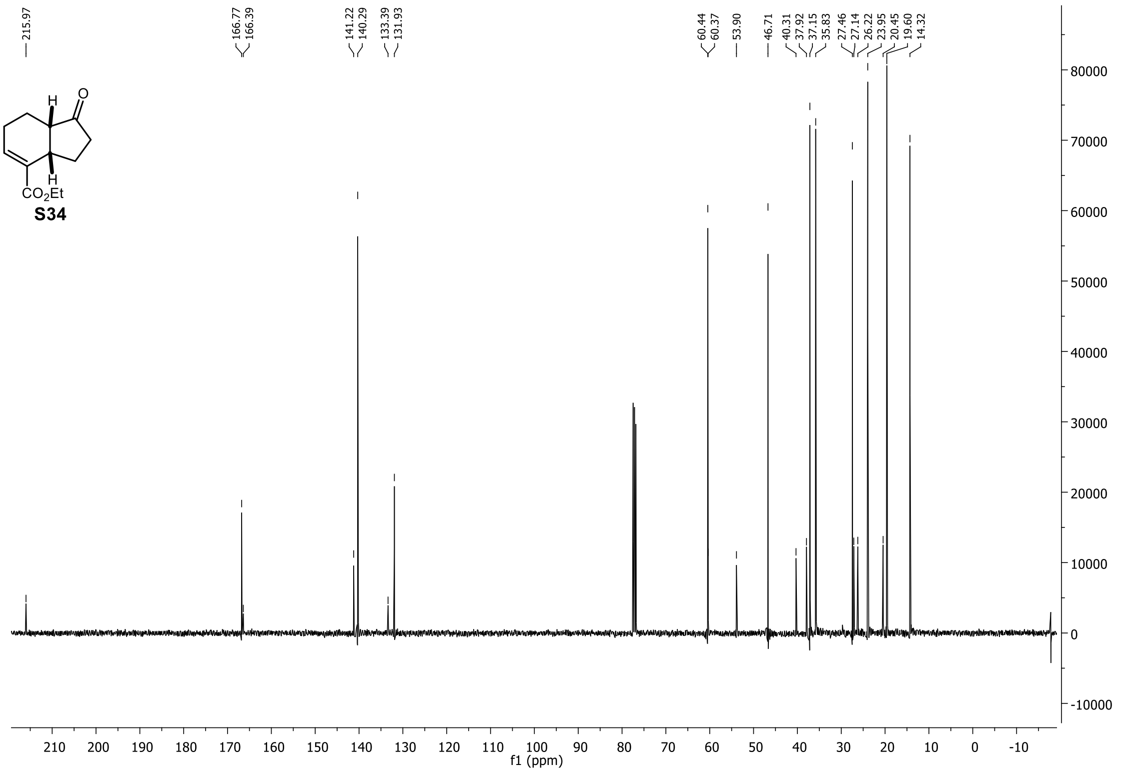

Supplementary Figure 65: ^{1}H NMR S35.

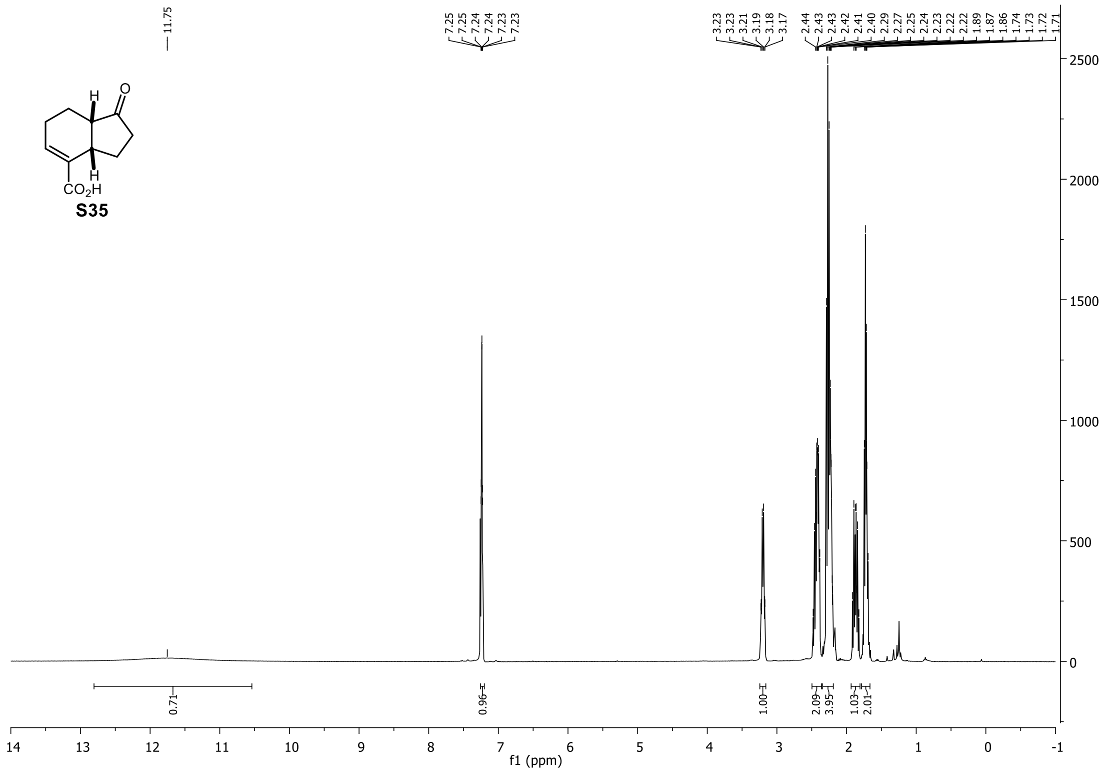

Supplementary Figure 66: ^{13}C NMR S35.

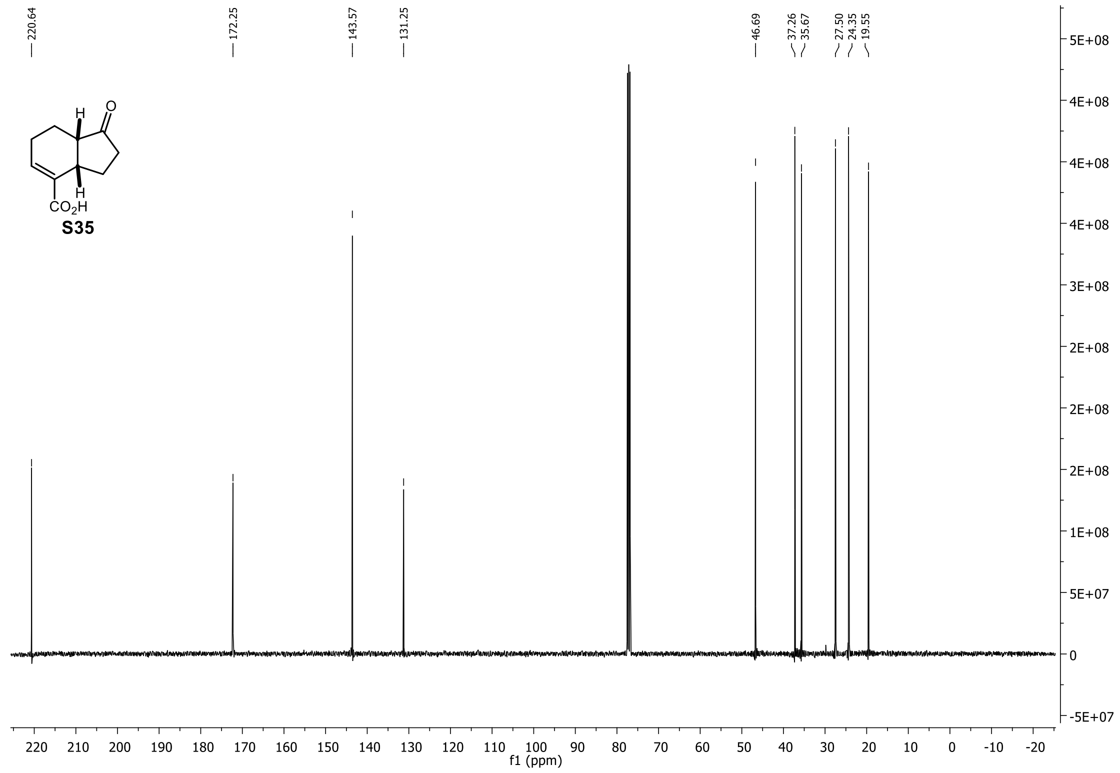

Supplementary Figure 67: ^{1}H NMR S4.

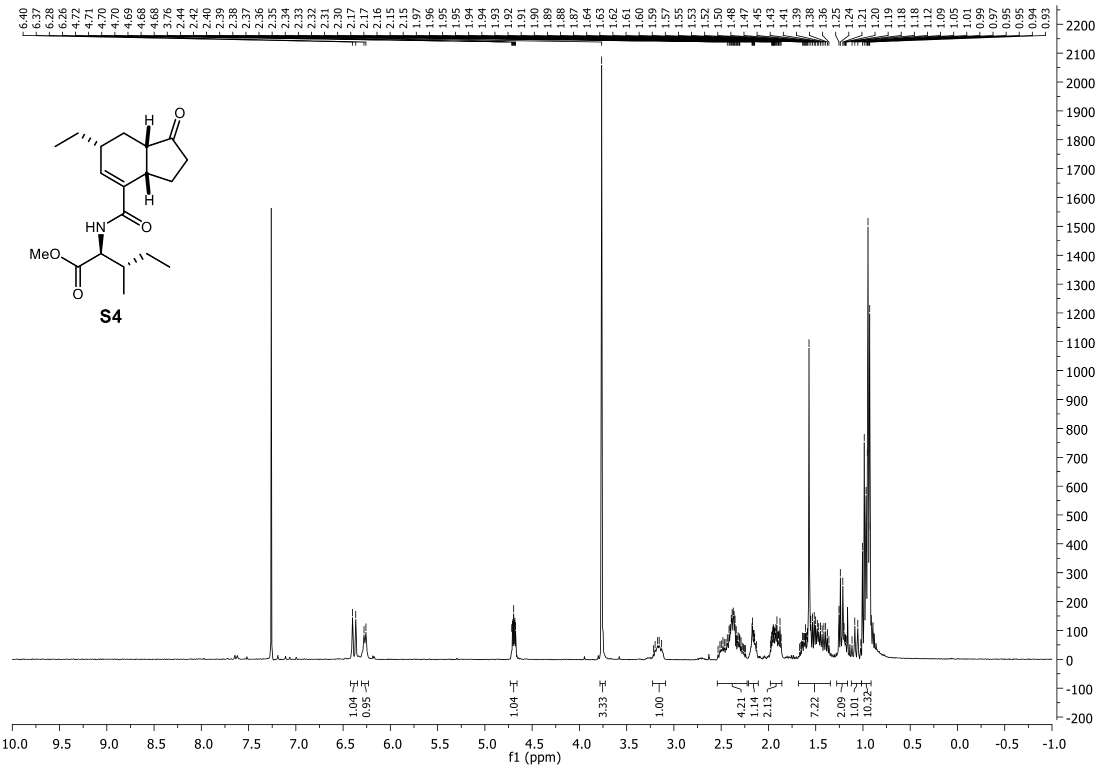

Supplementary Figure 68: ^{13}C NMR S4.

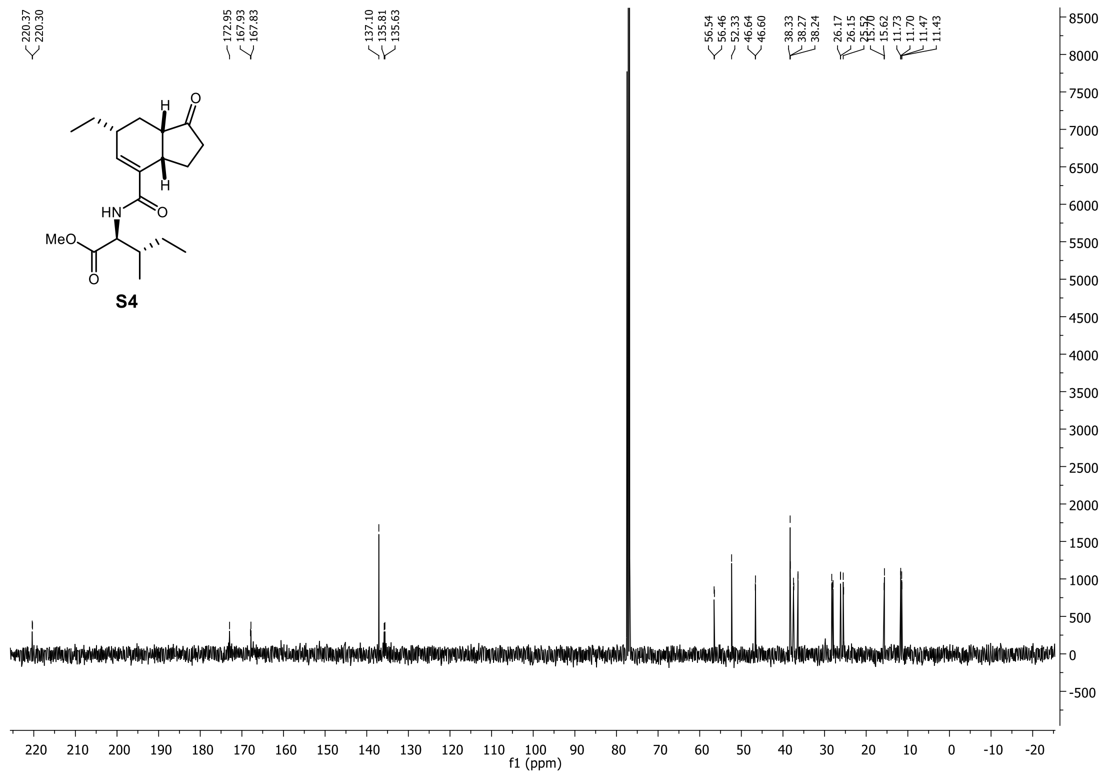

Supplementary Figure 69: ^{1}H NMR 16.

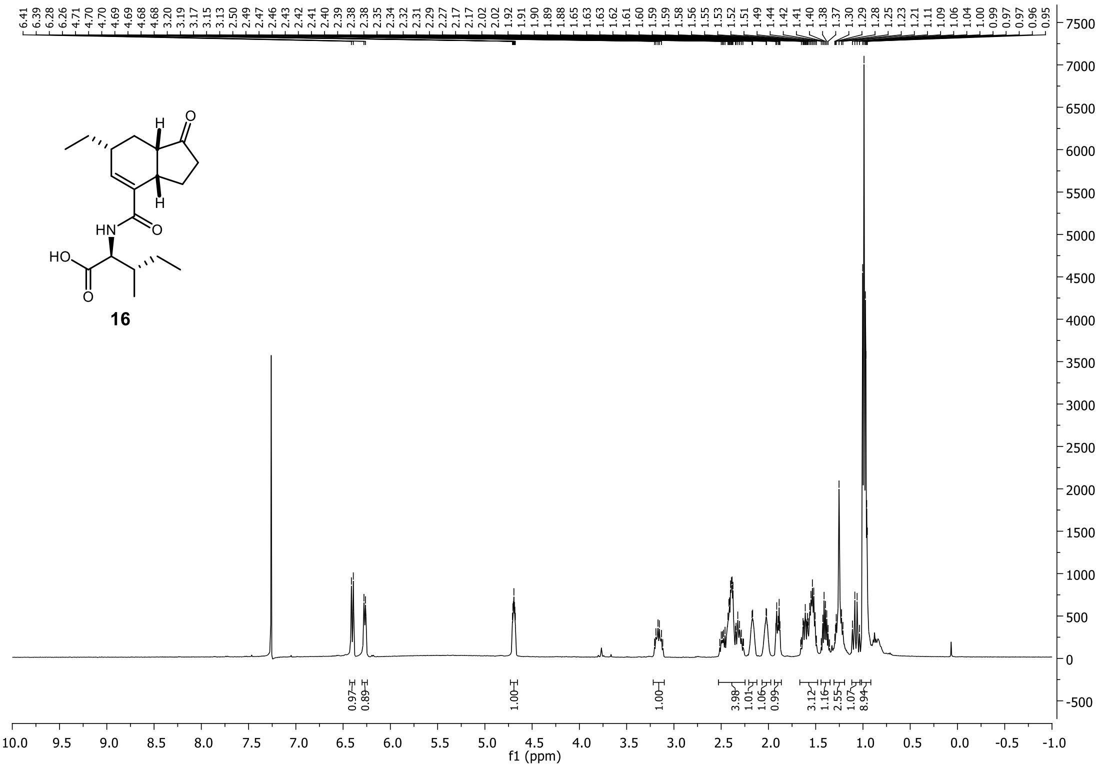

Supplementary Figure 70: ^{13}C NMR 16.

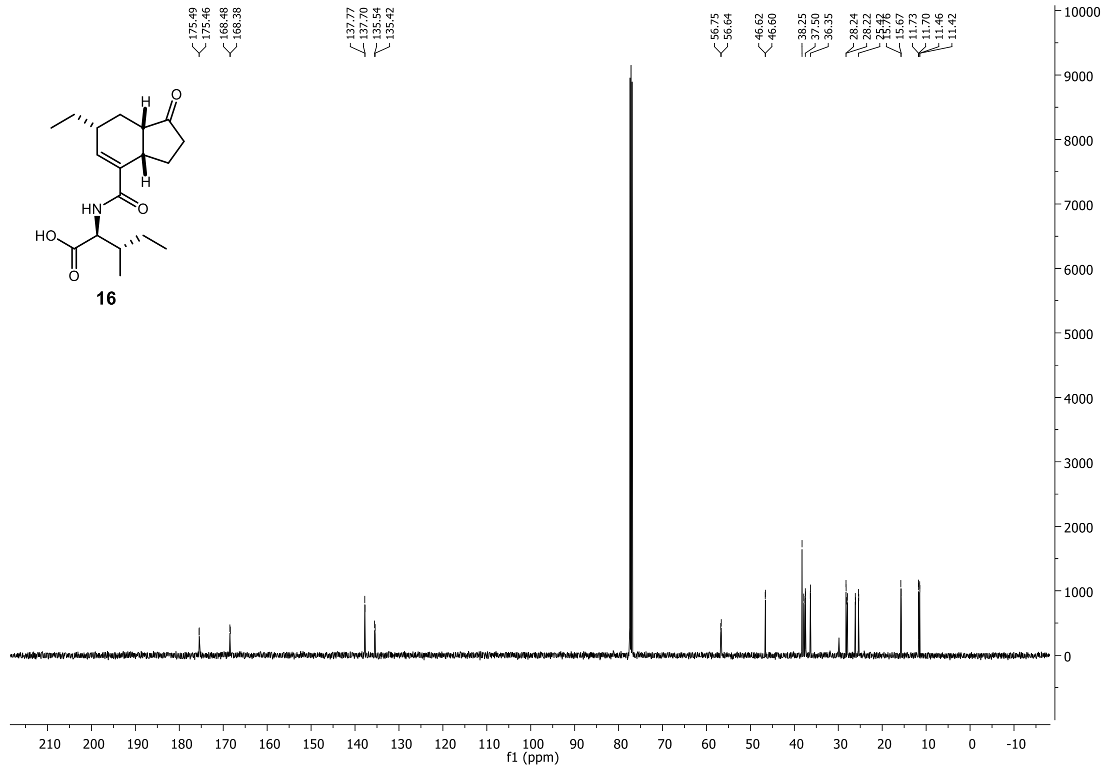

Supplementary Figure 71: ^{1}H NMR S36.

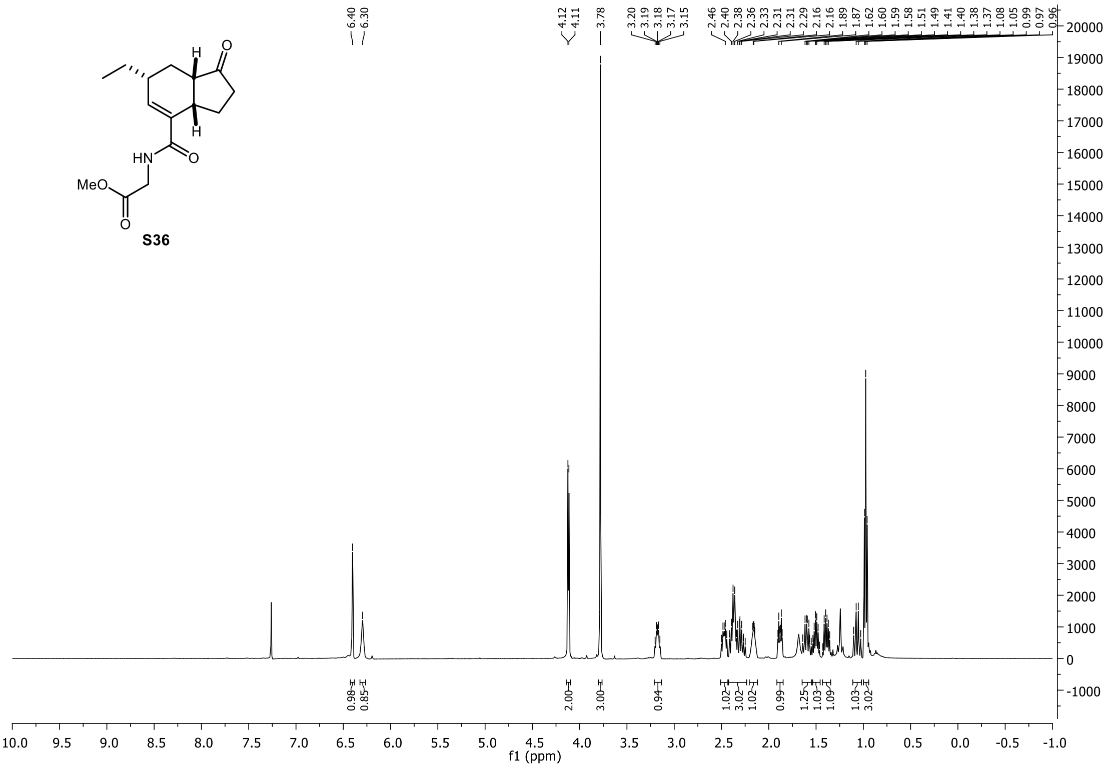

Supplementary Figure 72: ^{13}C NMR S36.

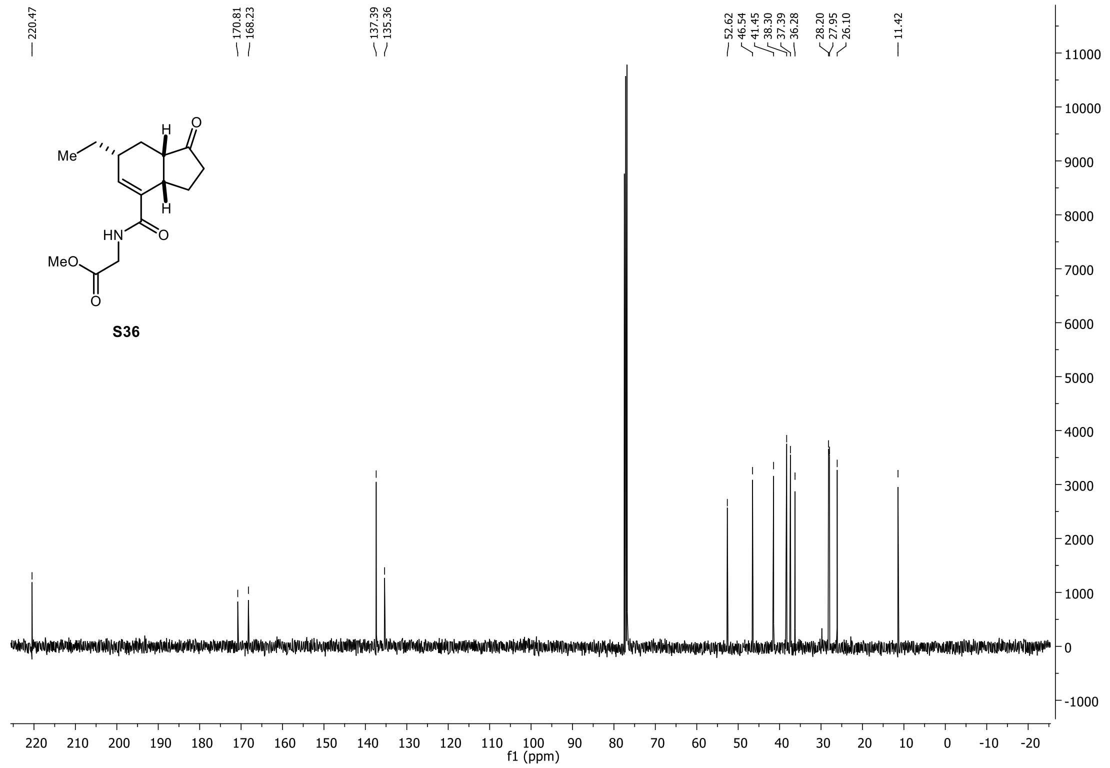

Supplementary Figure 73: ^{1}H NMR 17.

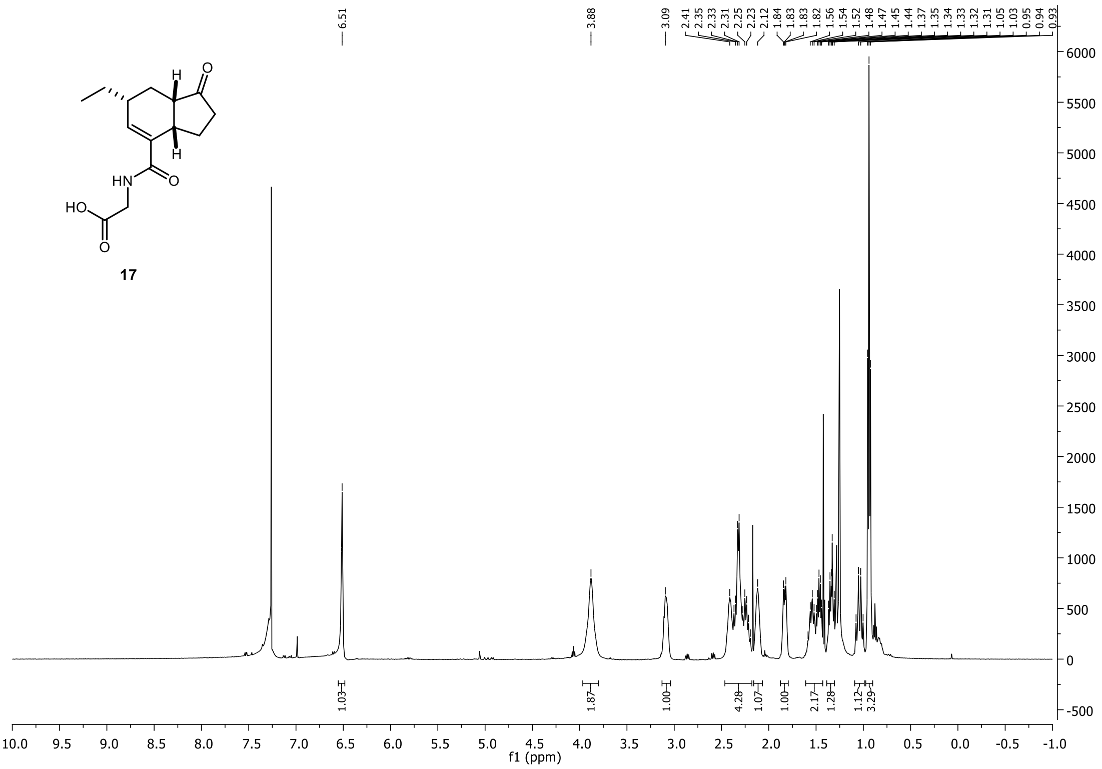

Supplementary Figure 74: ^{13}C NMR 17.

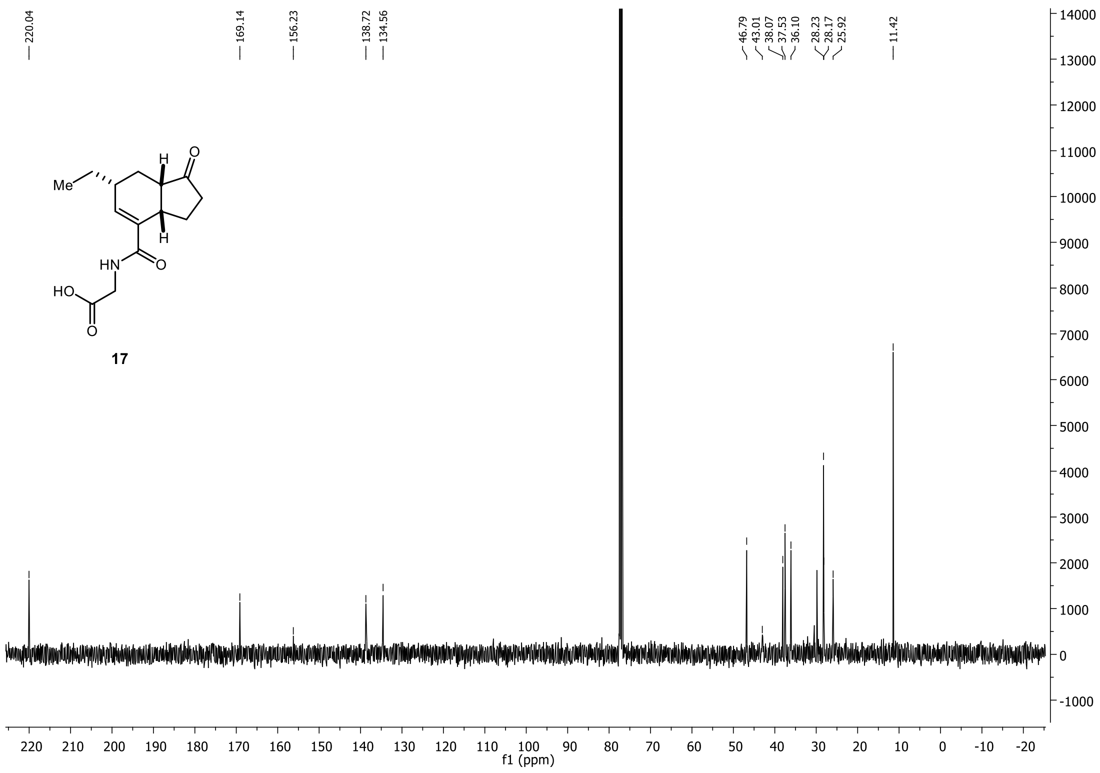

Supplementary Figure 75: ^{1}H NMR S37.

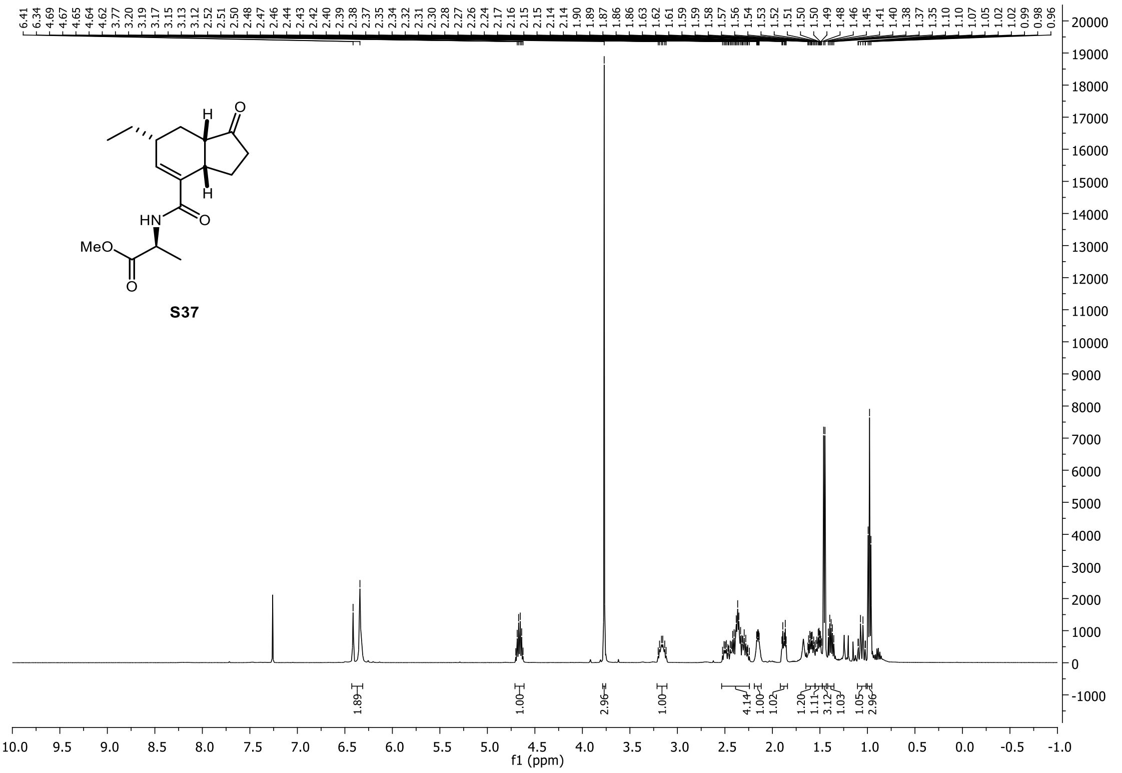

Supplementary Figure 76: ^{13}C NMR S37.

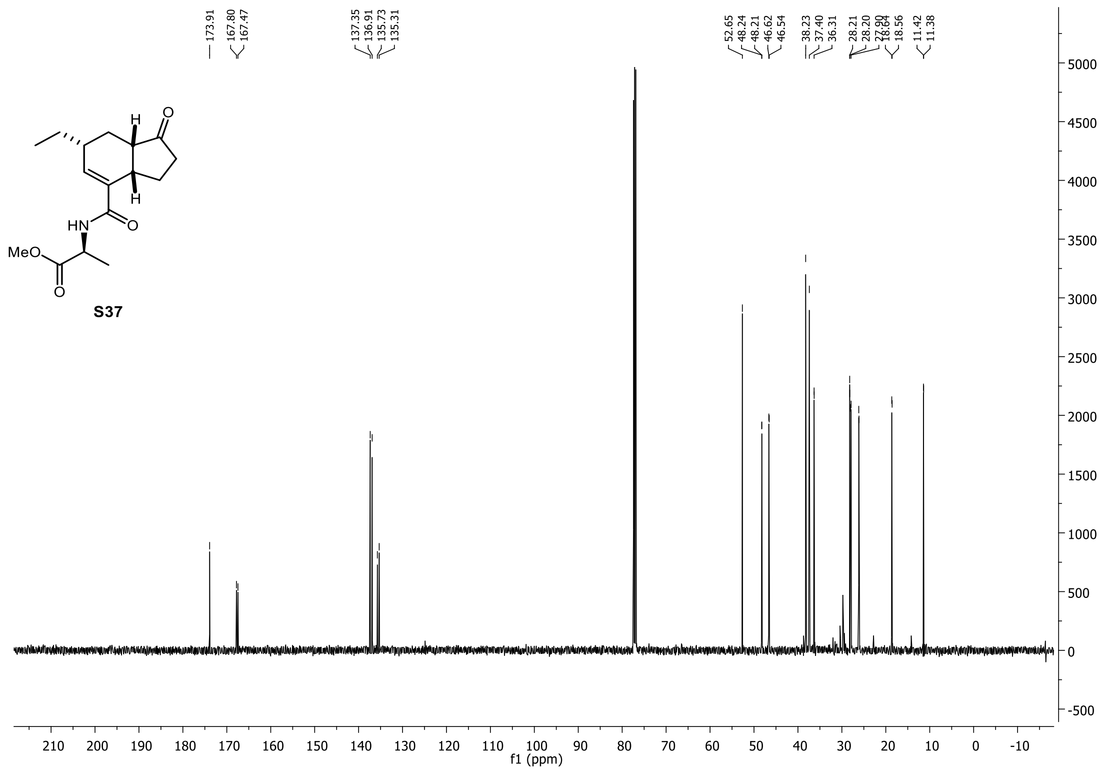

Supplementary Figure 77: ^{1}H NMR 18.

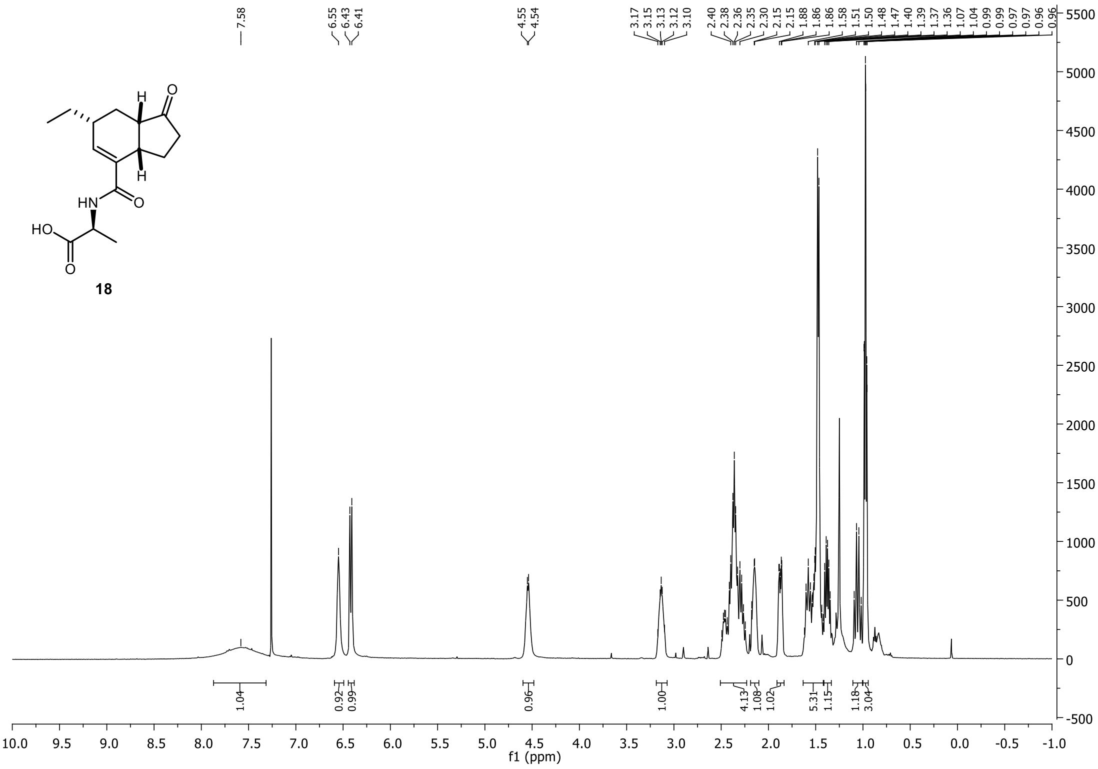

Supplementary Figure 78: ^{13}C NMR 18.

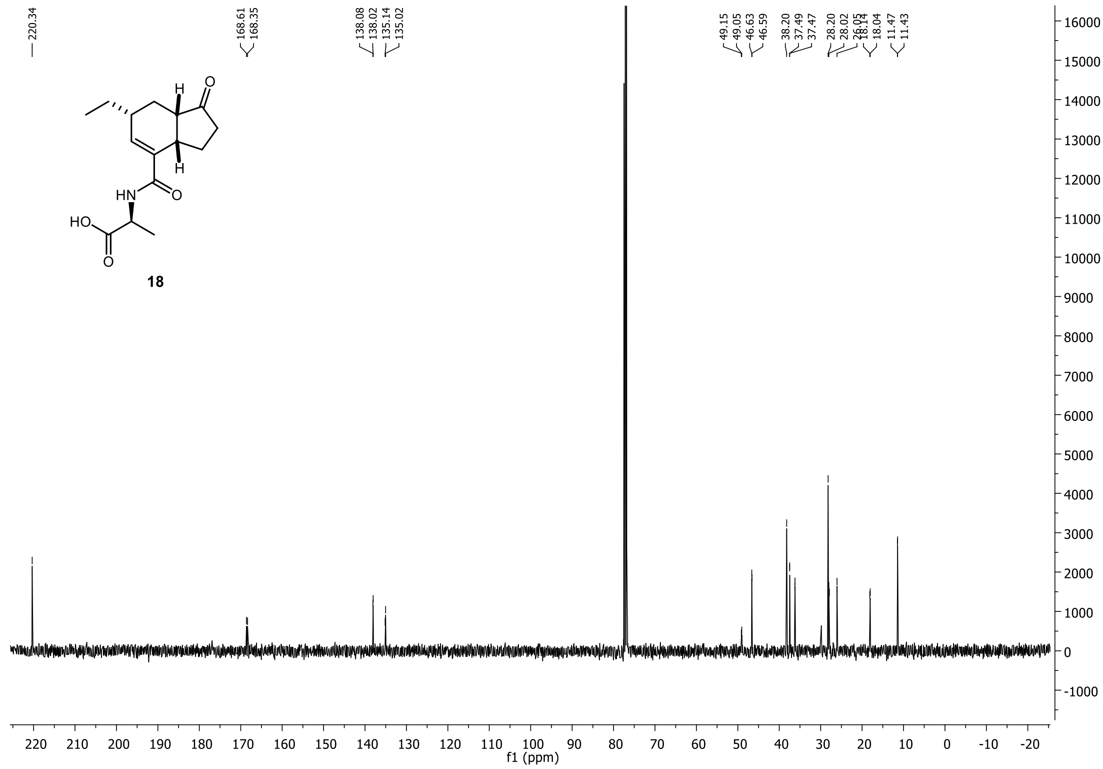

Supplementary Figure 79: ^{1}H NMR S38.

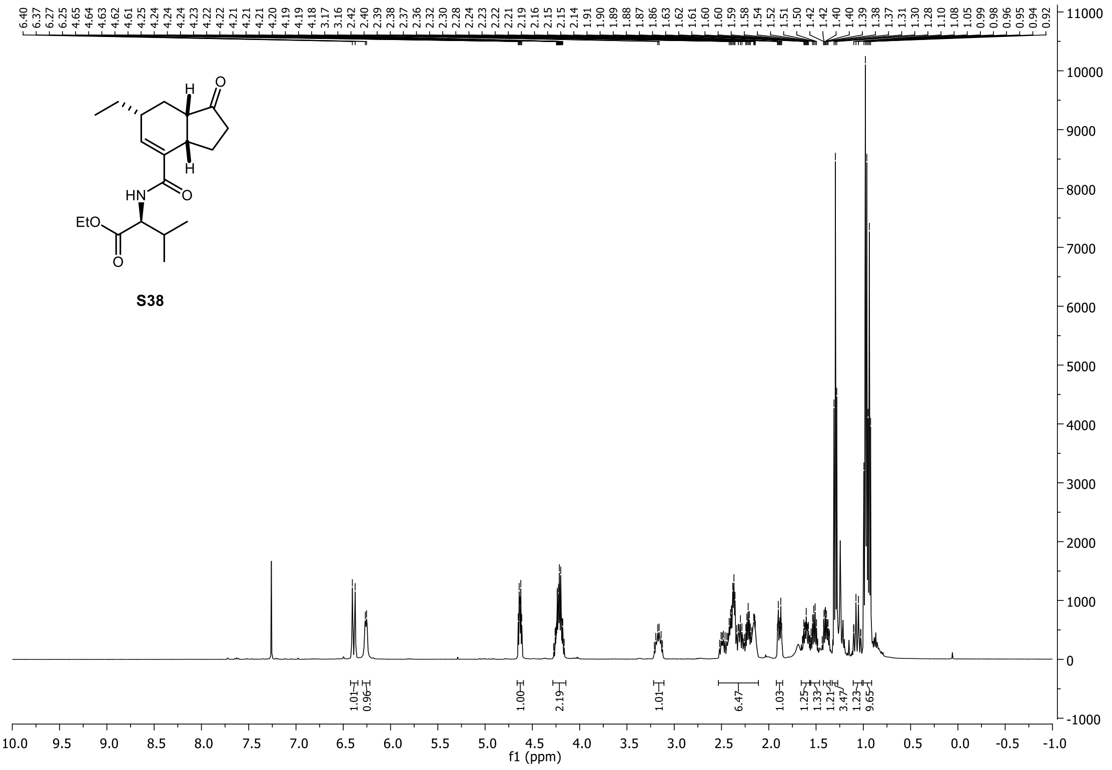

Supplementary Figure 80: ^{13}C NMR S38.

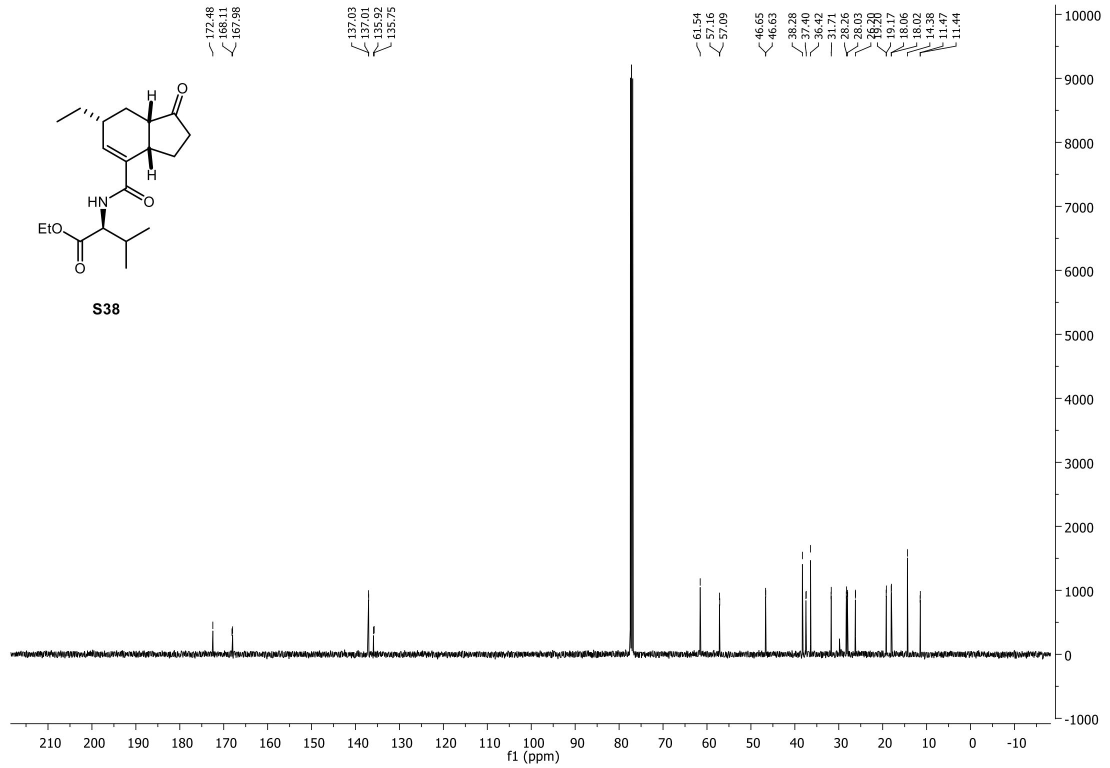

Supplementary Figure 81: ^{1}H NMR 19.

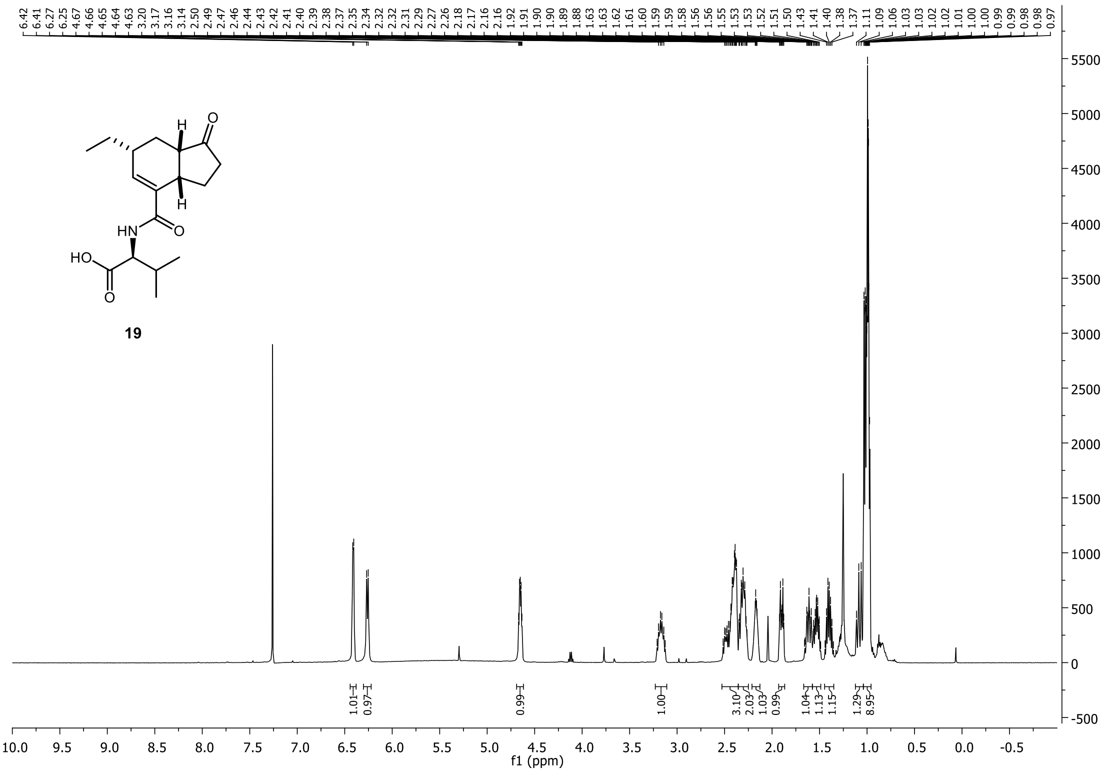

Supplementary Figure 82: ^{13}C NMR 19.

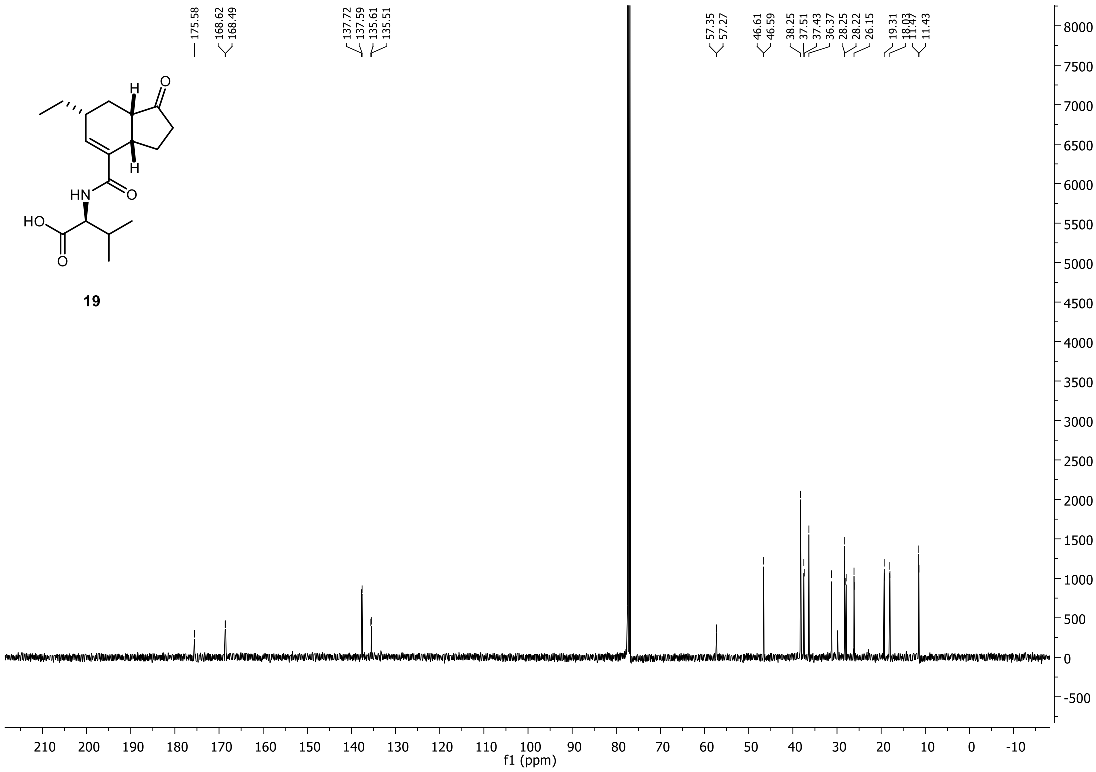

Supplementary Figure 83: ^{1}H NMR S39.

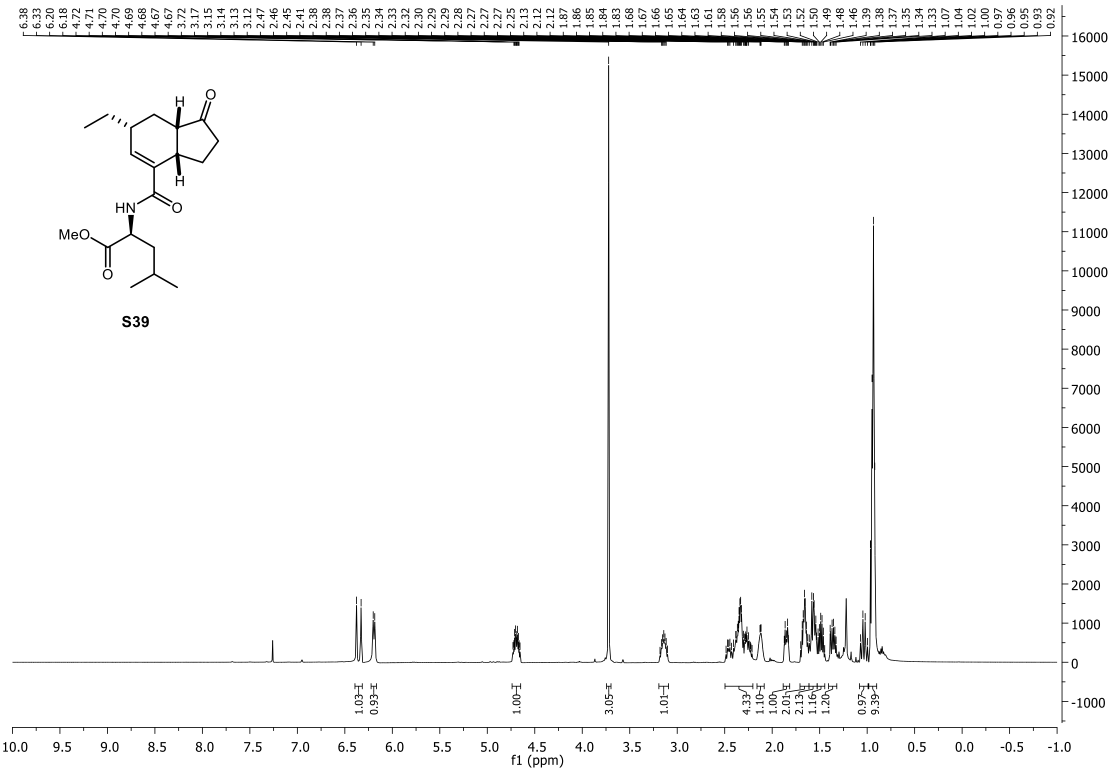

Supplementary Figure 84: ^{13}C NMR S39.

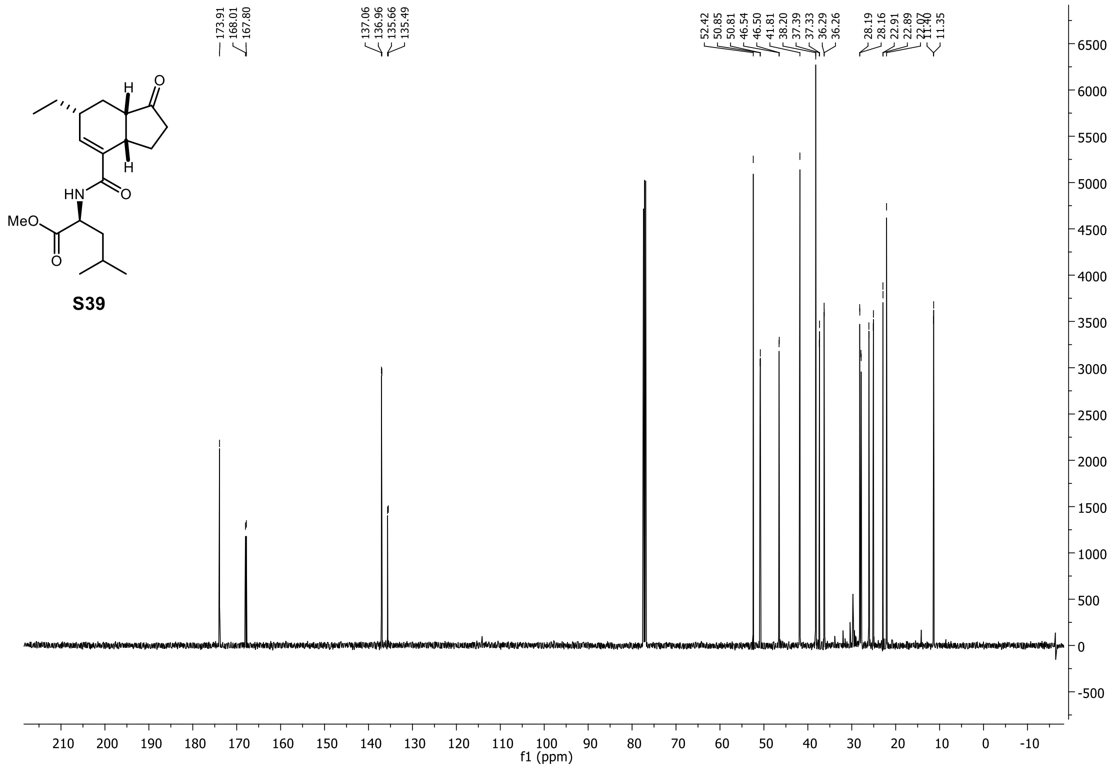

Supplementary Figure 85: ^{1}H NMR 20.

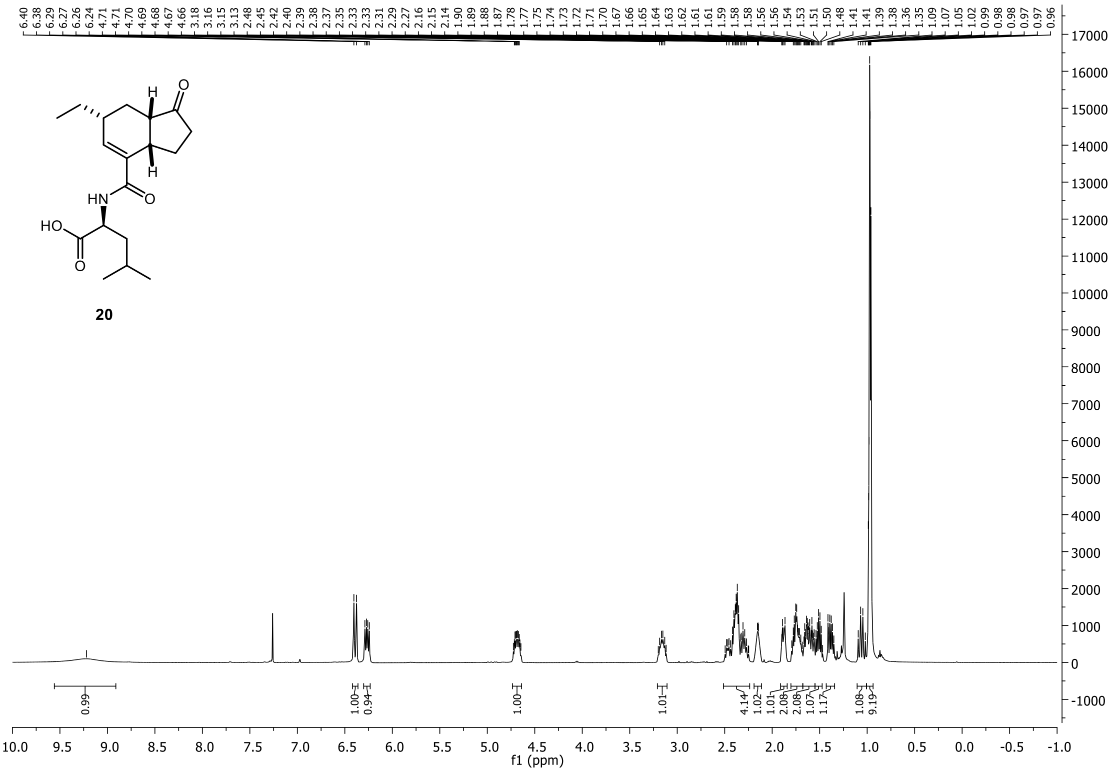

Supplementary Figure 86: ^{13}C NMR 20.

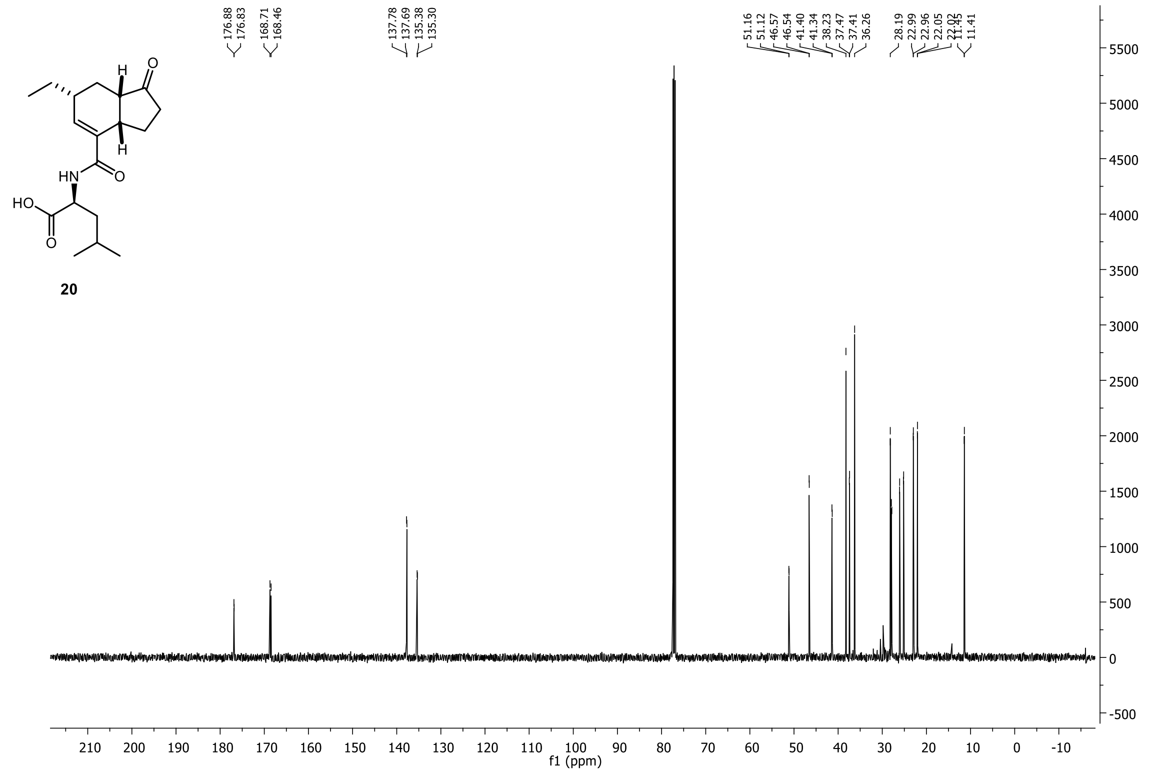

Supplementary Figure 87: ¹H NMR S40.

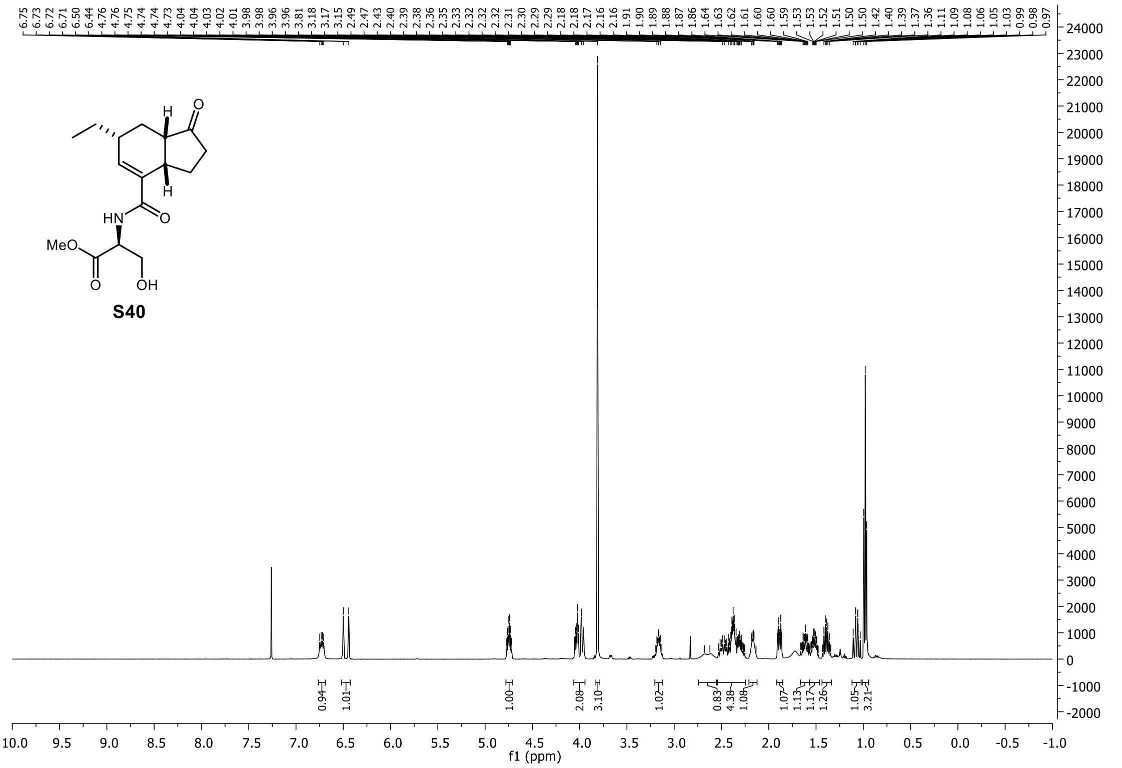

Supplementary Figure 88: ¹³C NMR S40.

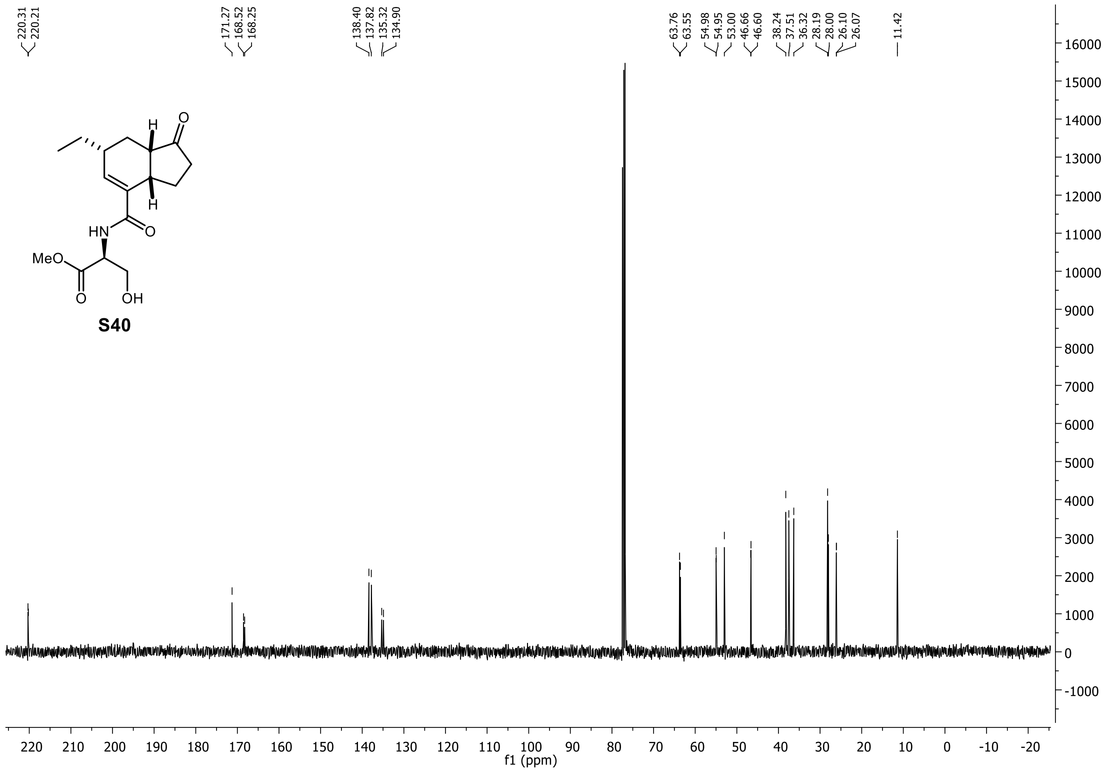

Supplementary Figure 89: ^{1}H NMR 21.

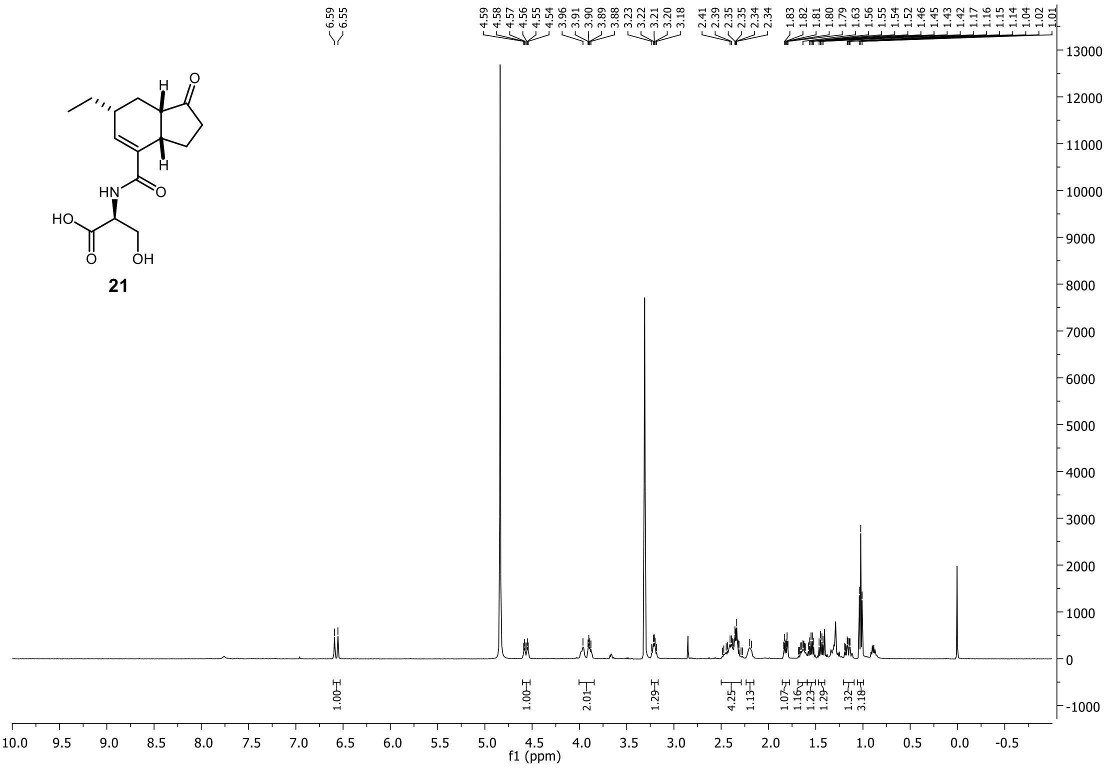

Supplementary Figure 90: ^{13}C NMR 21.

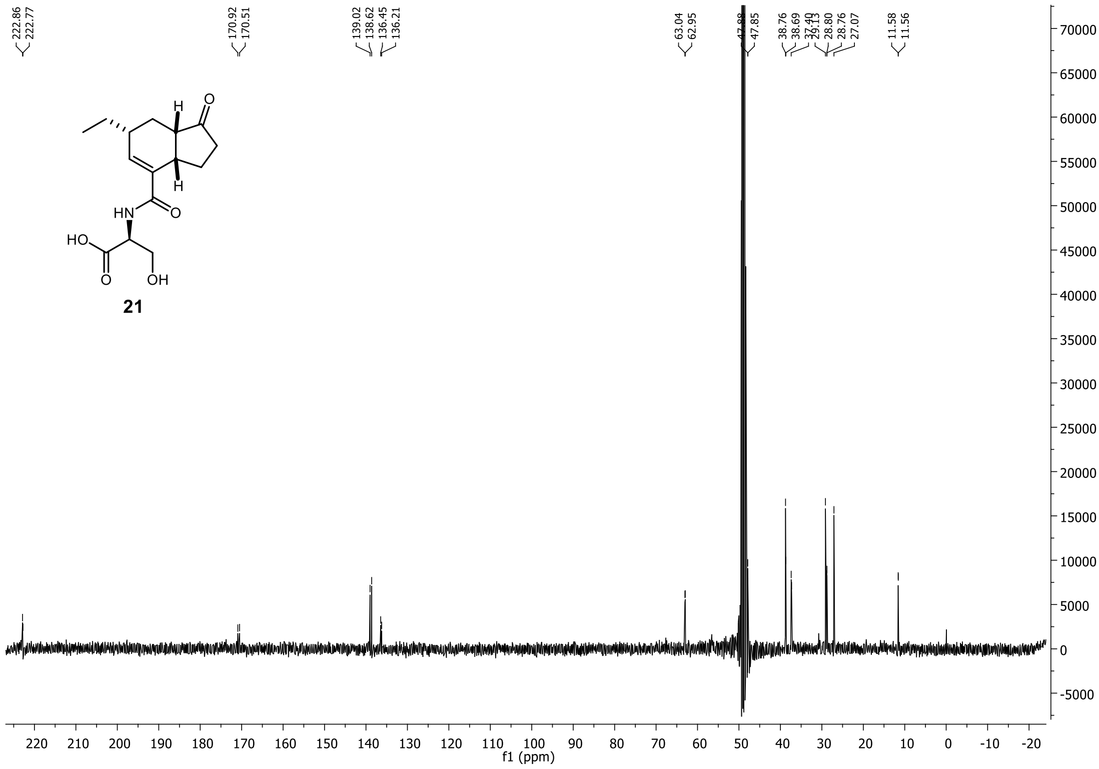

Supplementary Figure 91: ^{1}H NMR S41.

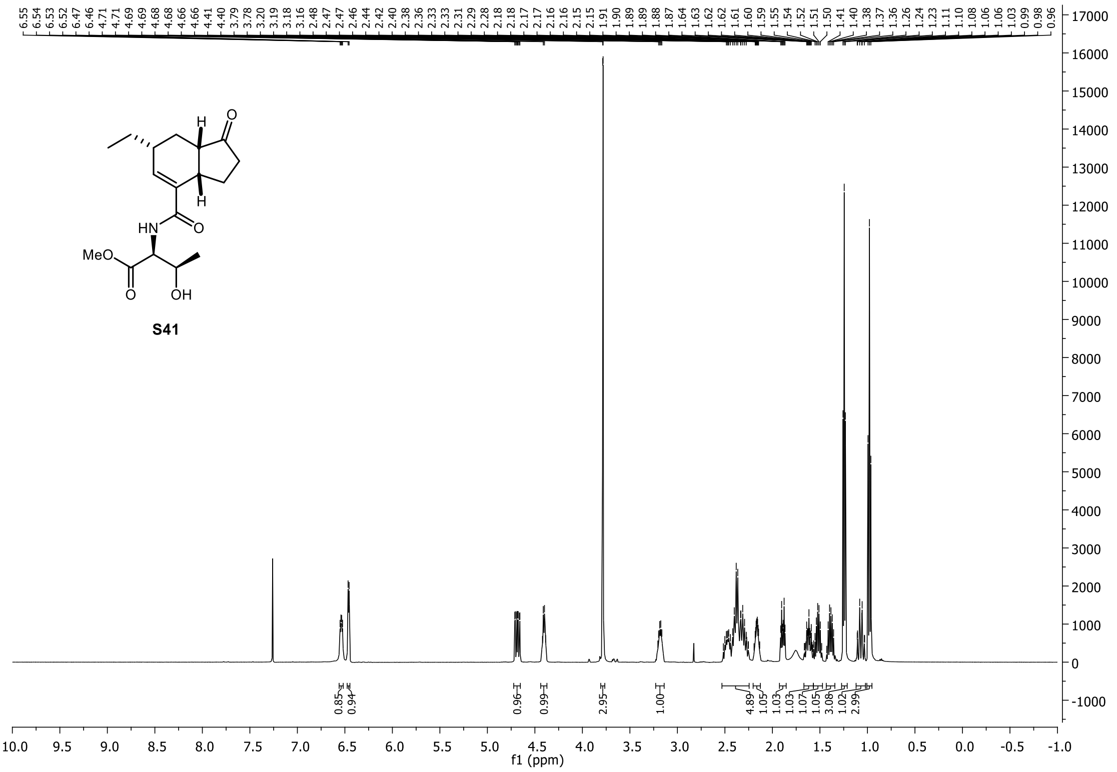

Supplementary Figure 92: ^{13}C NMR S41.

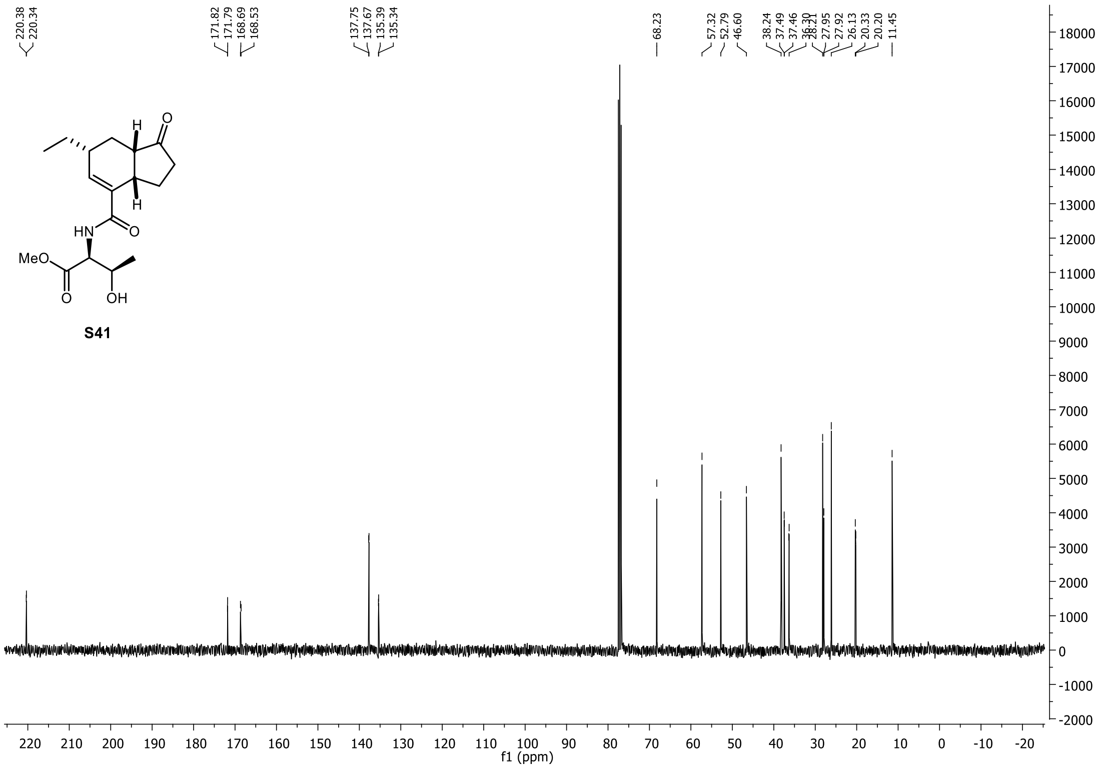

Supplementary Figure 93: ^{1}H NMR 22.

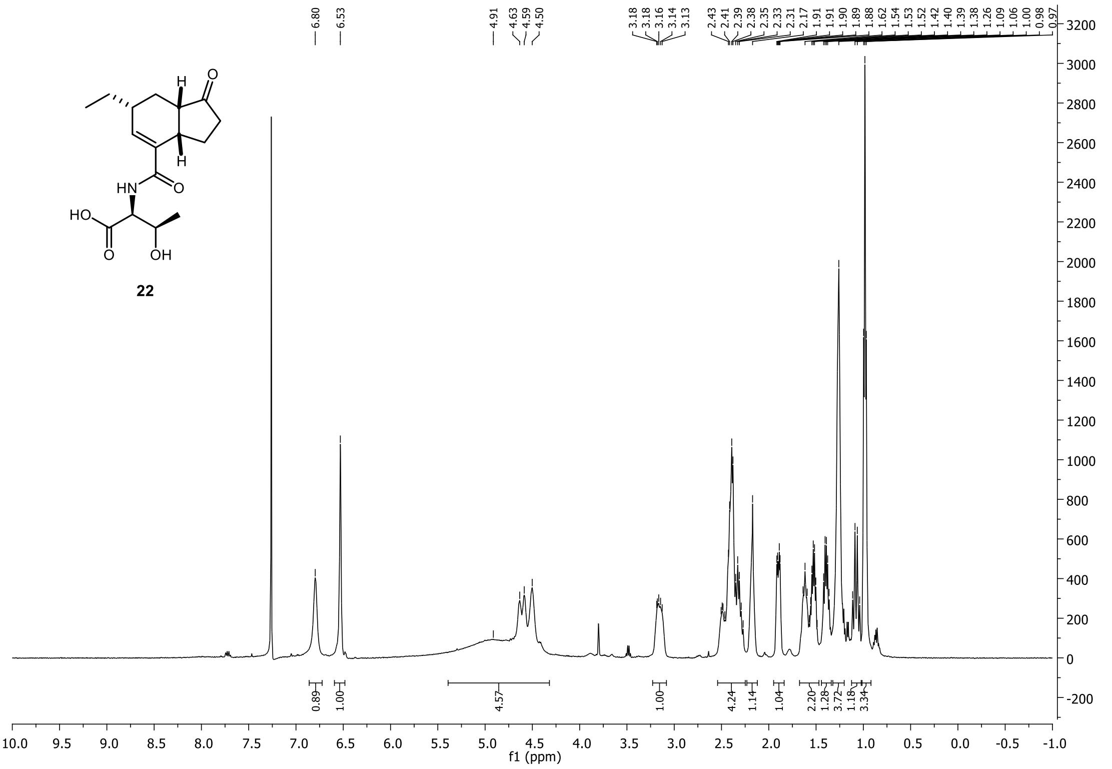

Supplementary Figure 94: ^{13}C NMR 22.

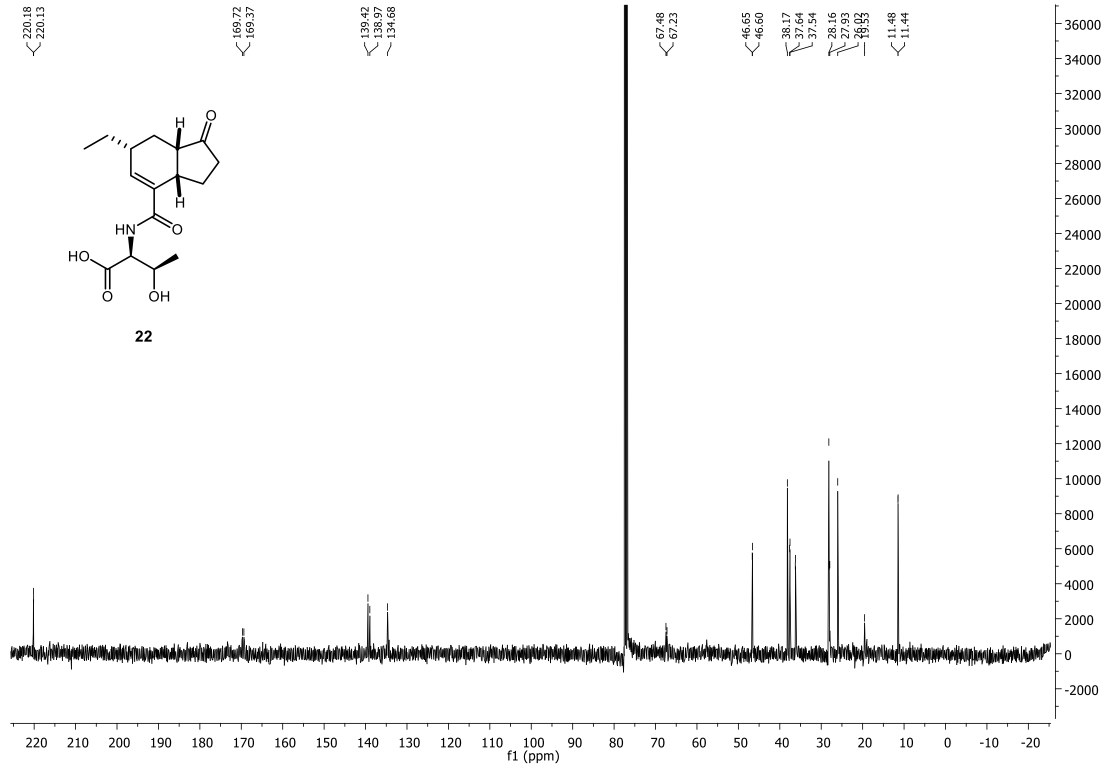

Supplementary Figure 95: ^{1}H NMR S42.

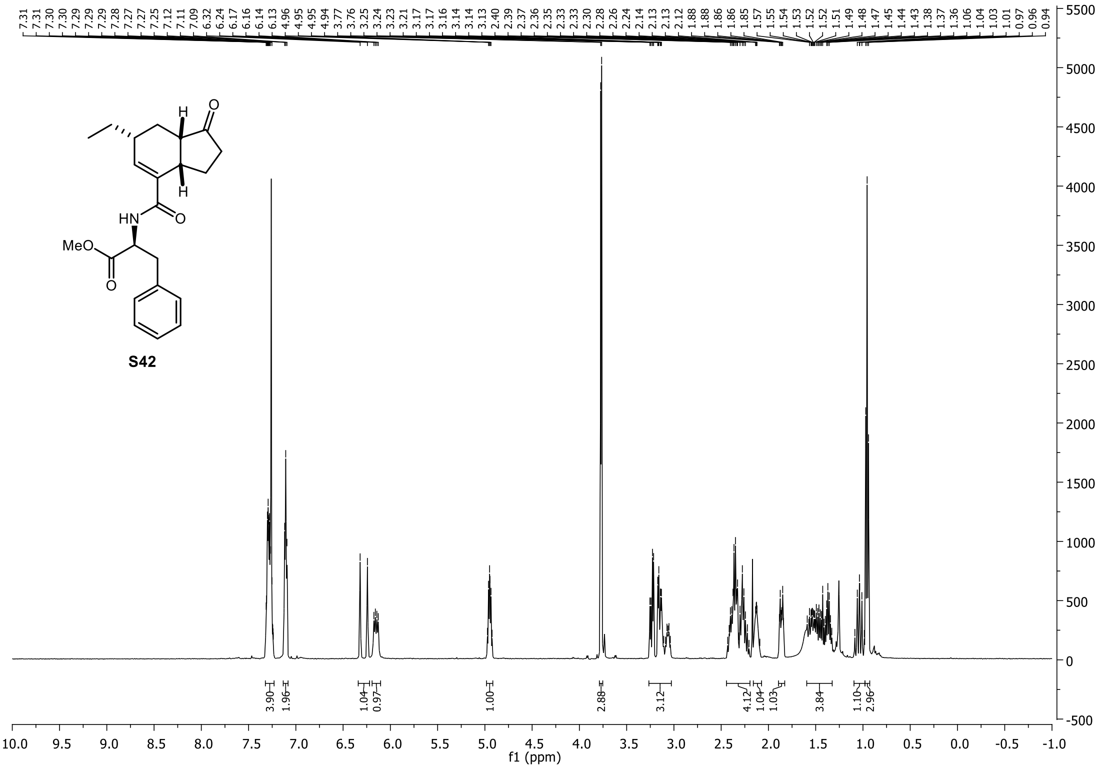

Supplementary Figure 96: ^{13}C NMR S42.

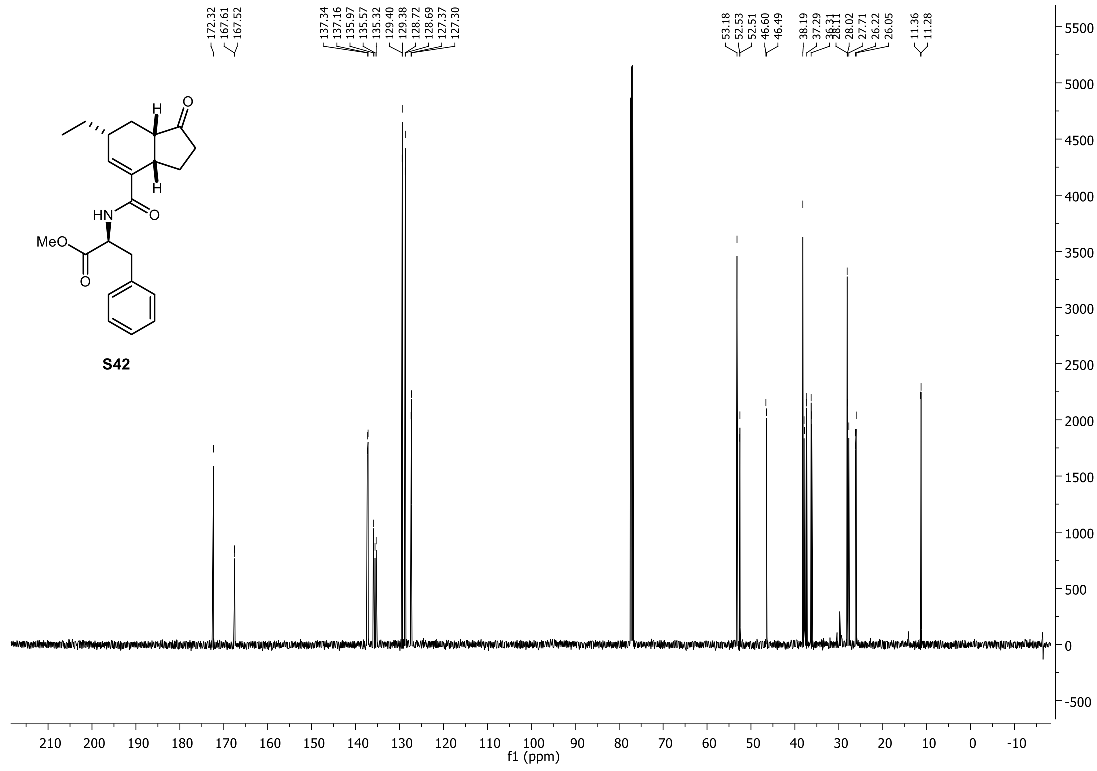

Supplementary Figure 97: ^{1}H NMR 23.

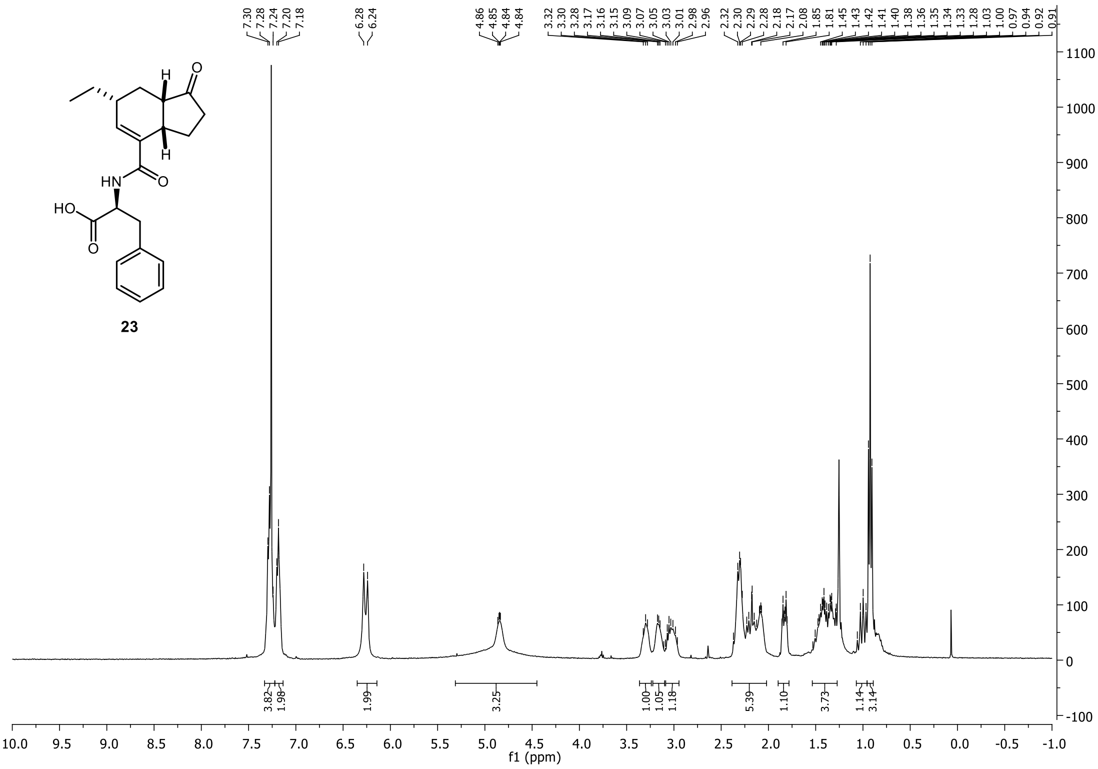

Supplementary Figure 98: ^{13}C NMR 23.

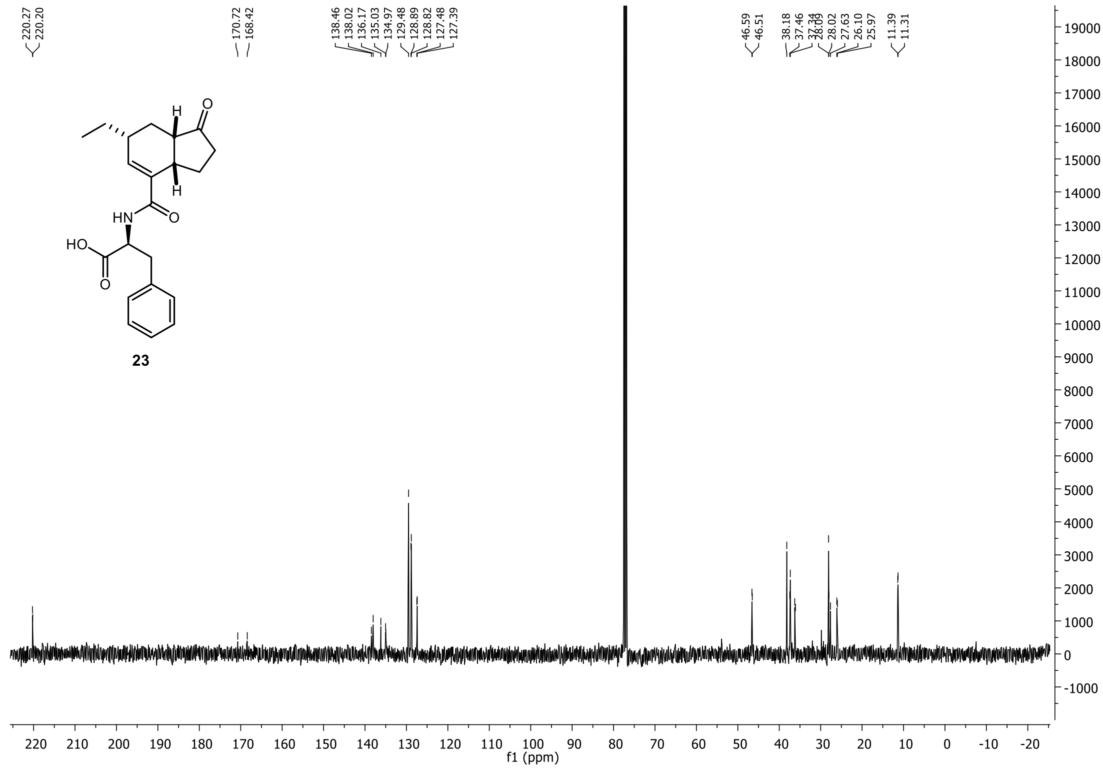

Supplementary Figure 99: ^{1}H NMR S43.

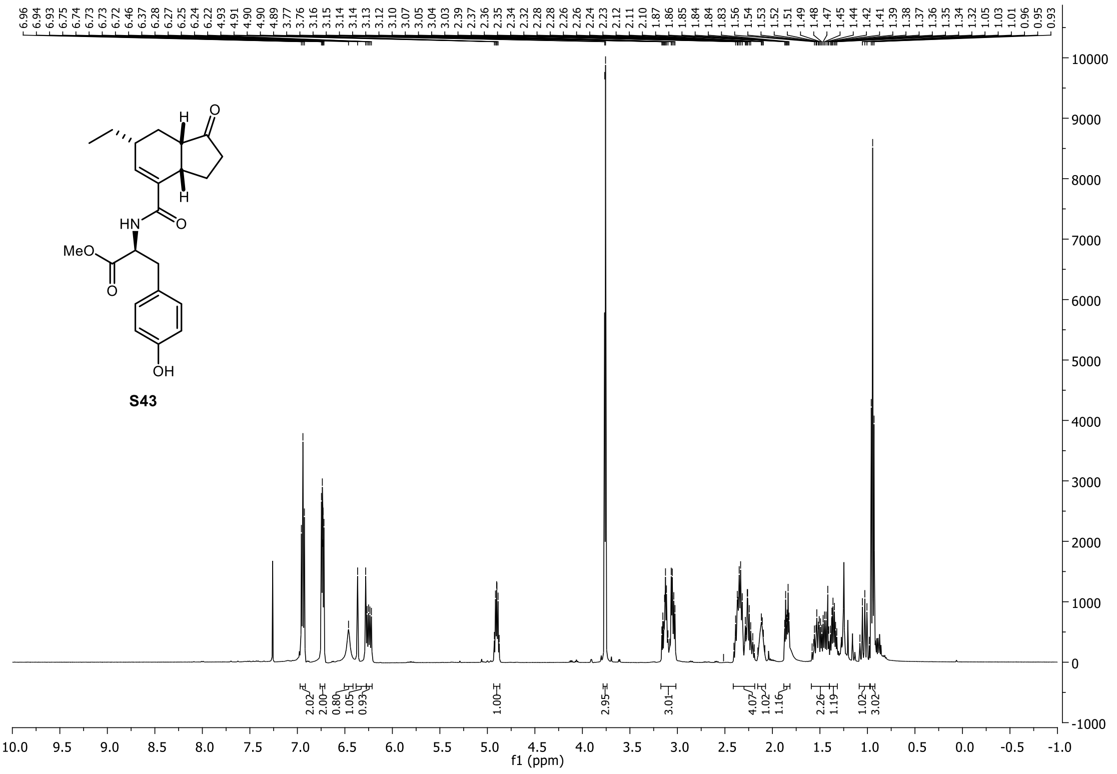

Supplementary Figure 100: ^{13}C NMR S43.

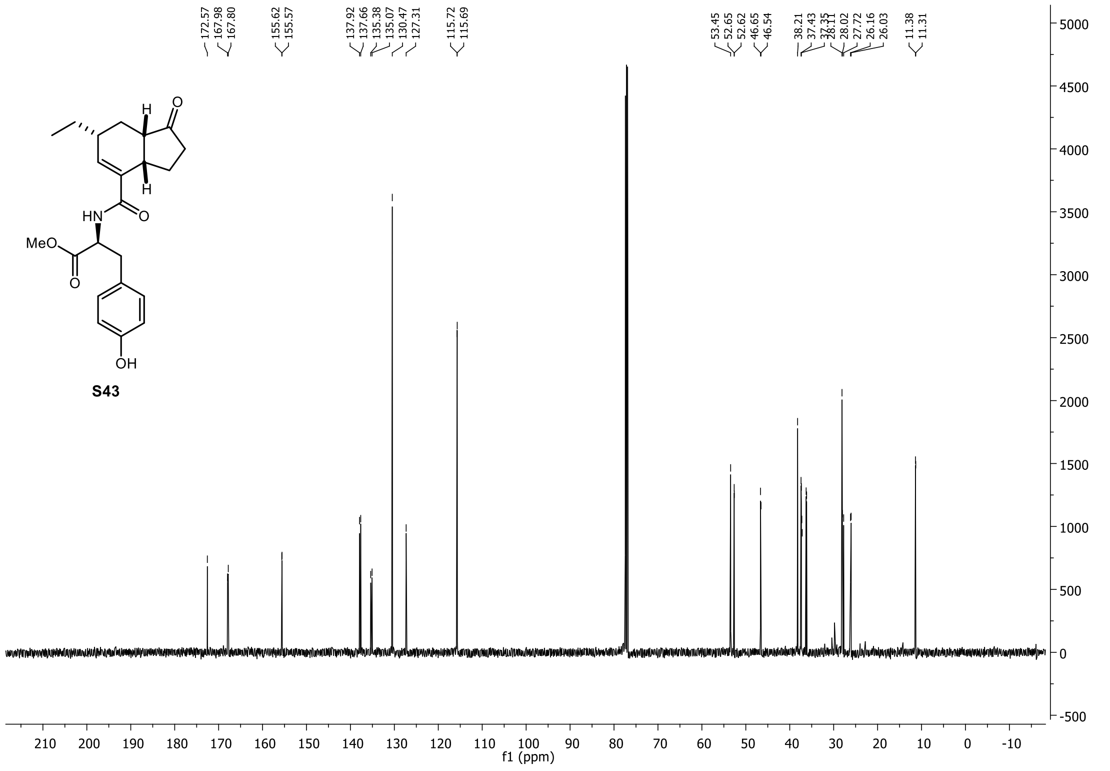

Supplementary Figure 101: ^{1}H NMR 24.

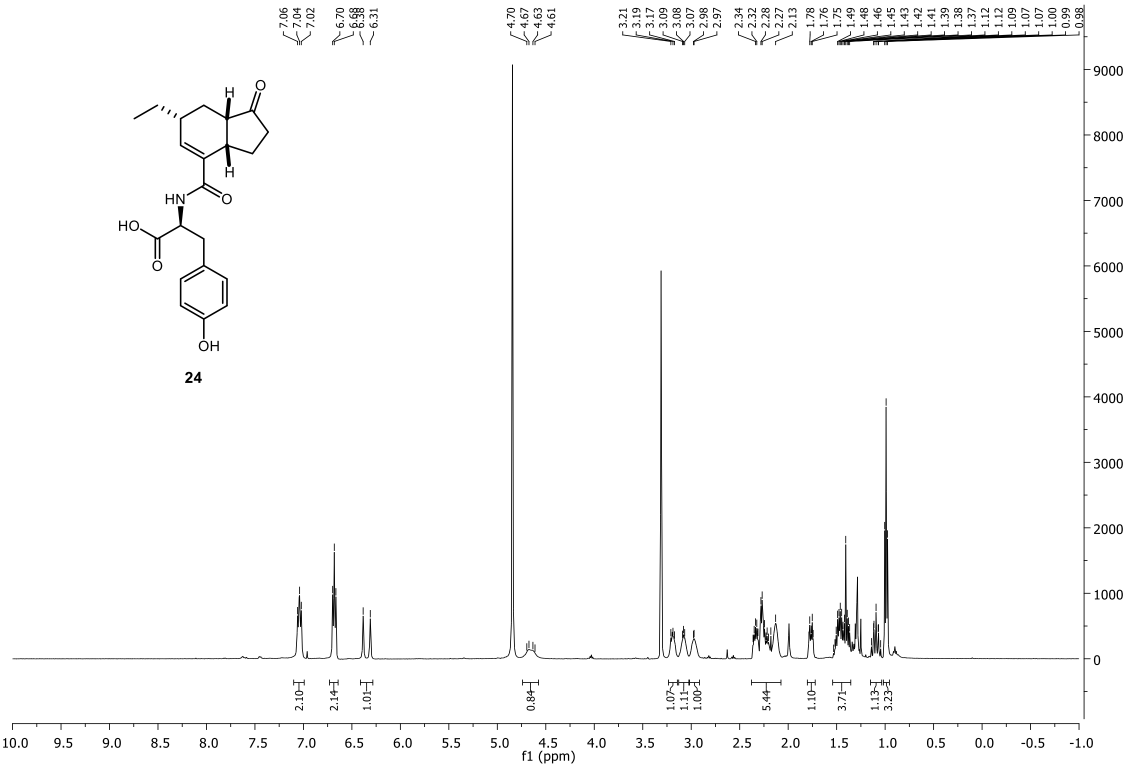

Supplementary Figure 102: ^{13}C NMR 24.

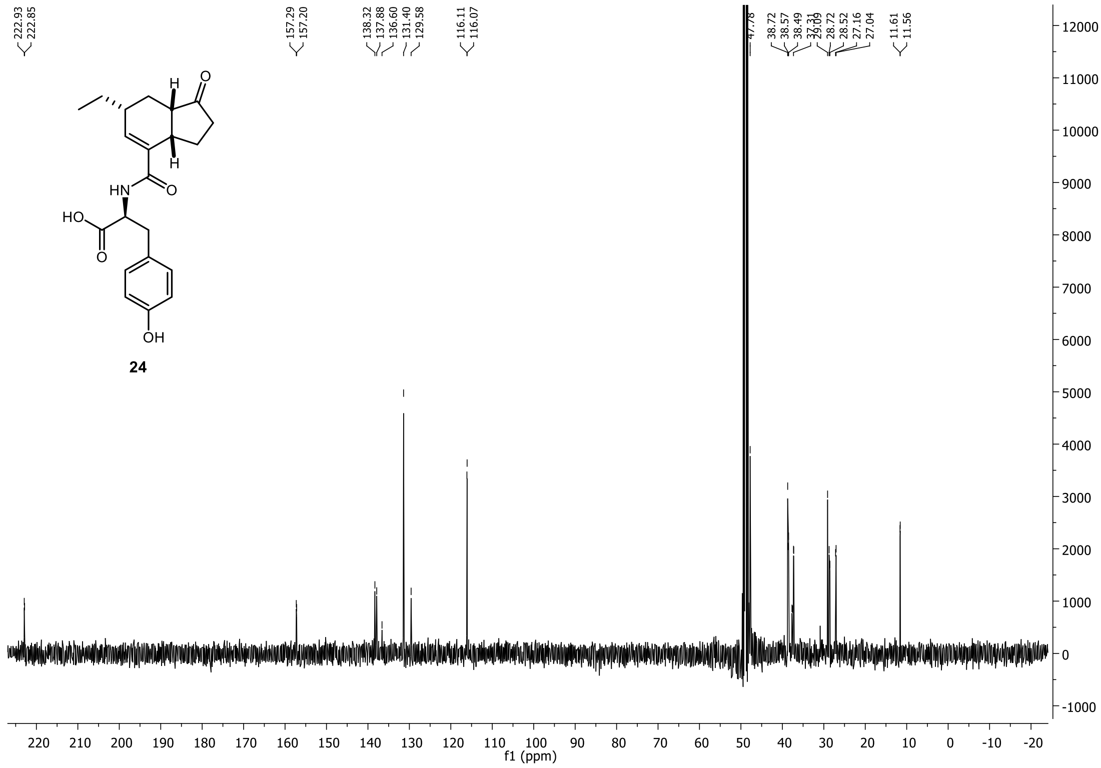

Supplementary Figure 103: ^{1}H NMR S44.

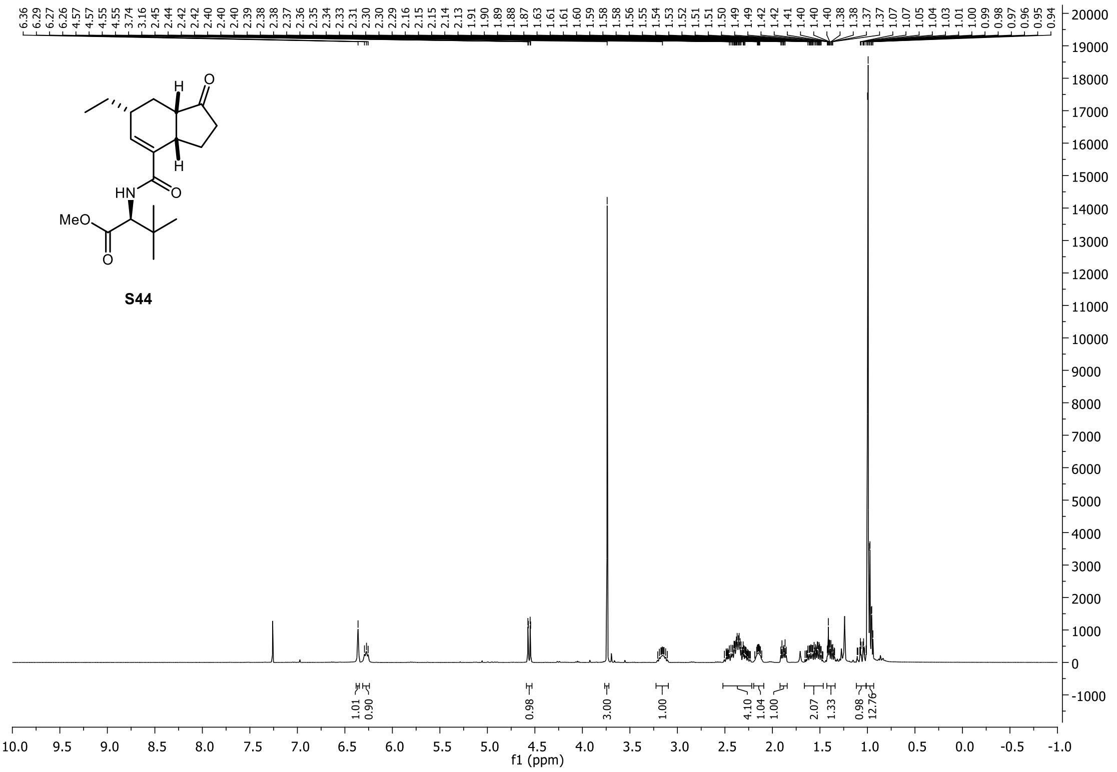

Supplementary Figure 104: ^{13}C NMR S44.

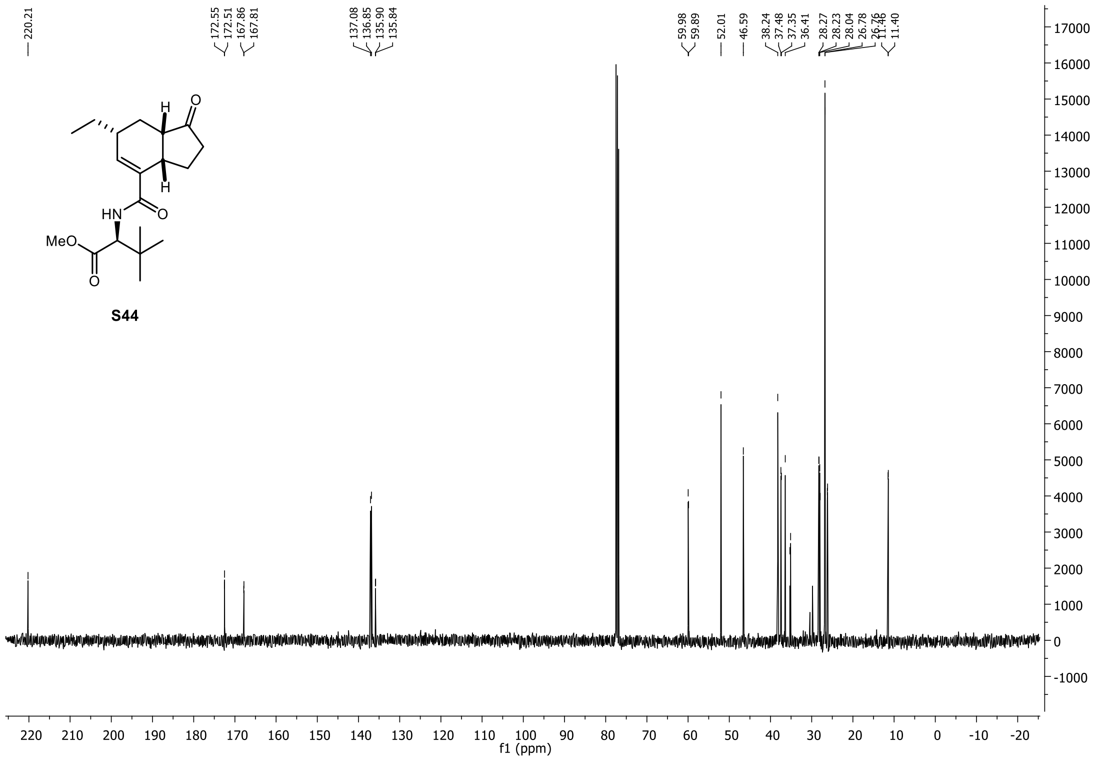

Supplementary Figure 105: ^{1}H NMR 25.

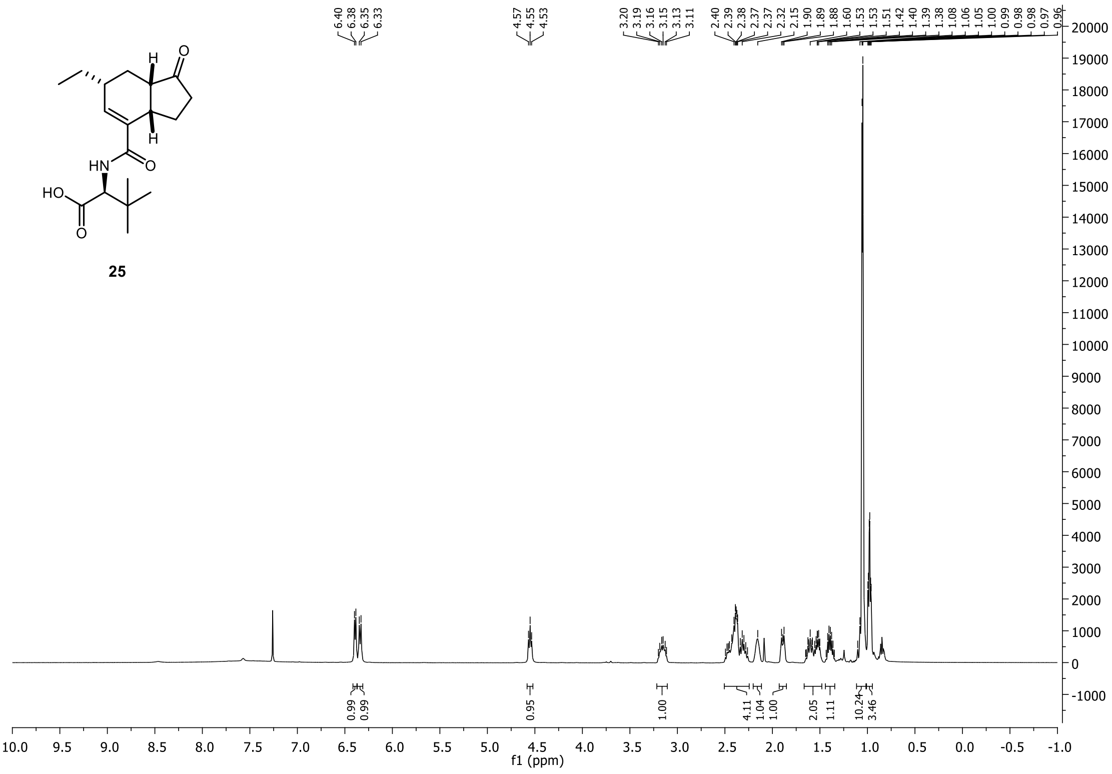

Supplementary Figure 106: ^{13}C NMR 25.

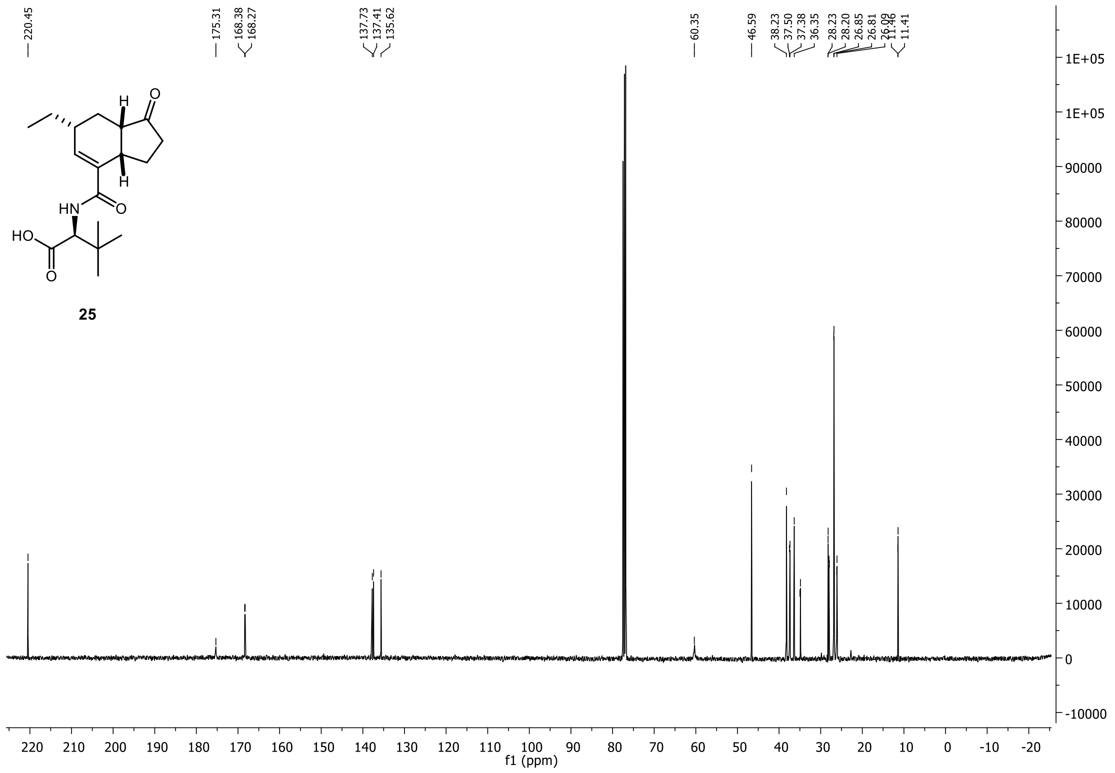

Supplementary Figure 107: ^{1}H NMR S45.

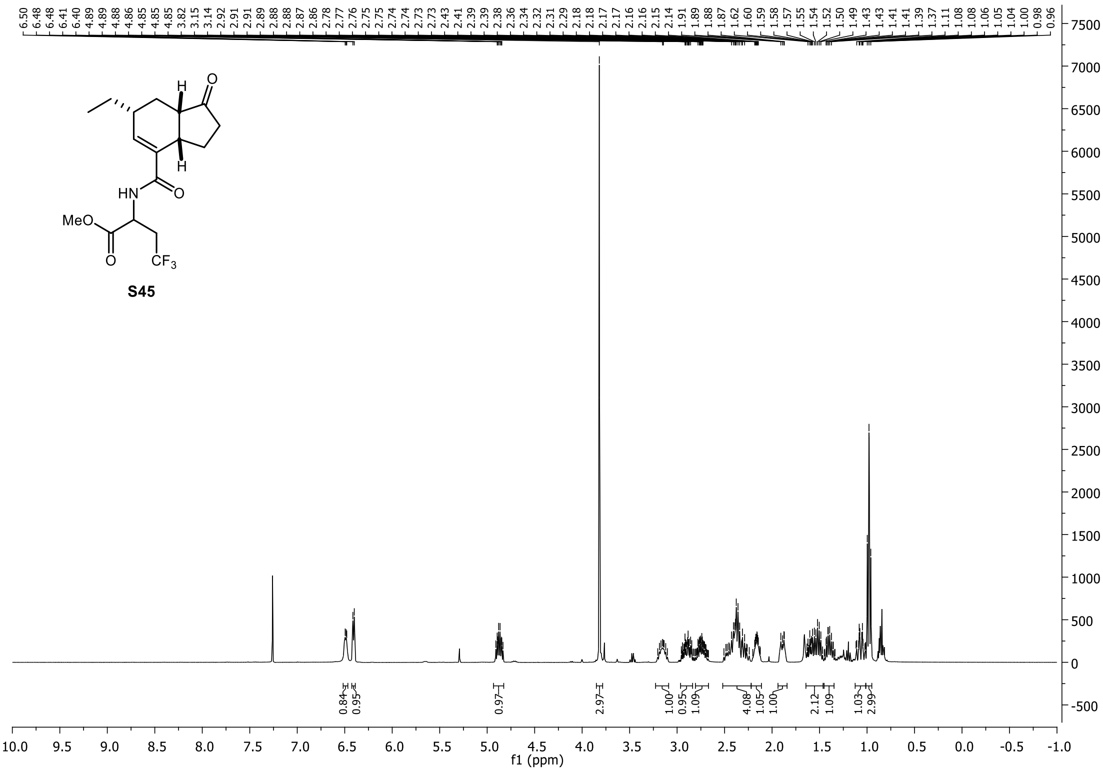

Supplementary Figure 108: ^{13}C NMR S45.

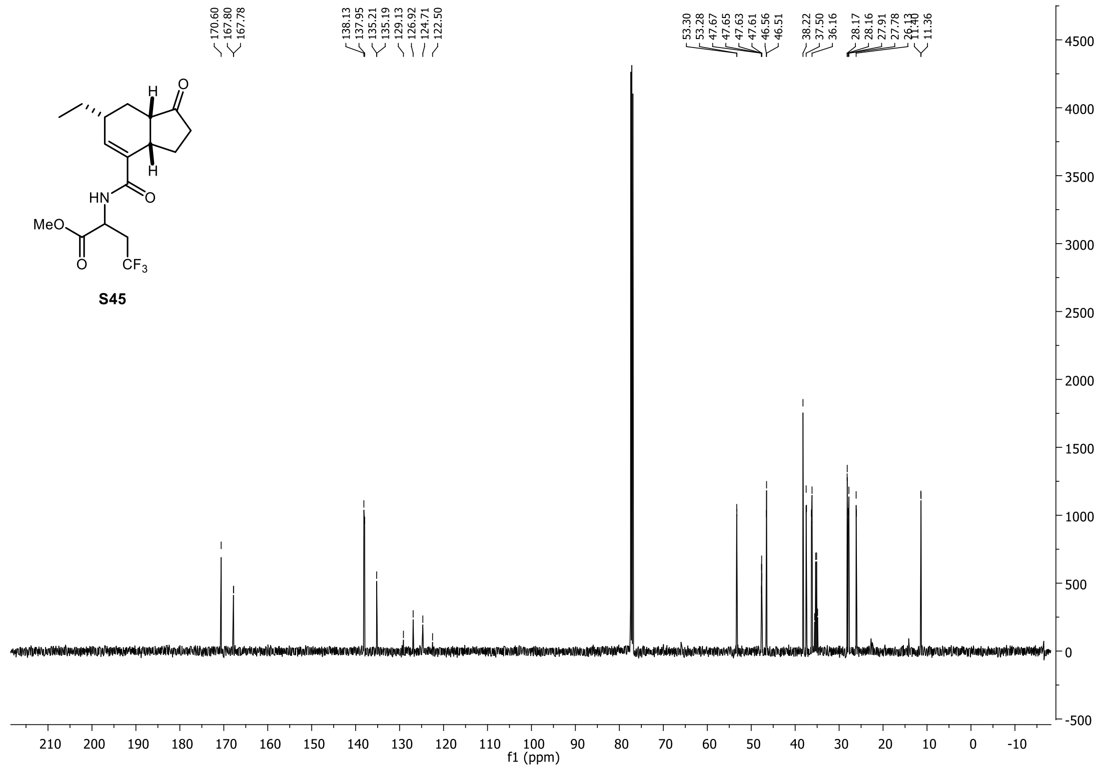

Supplementary Figure 109: ^{19}F NMR S45.

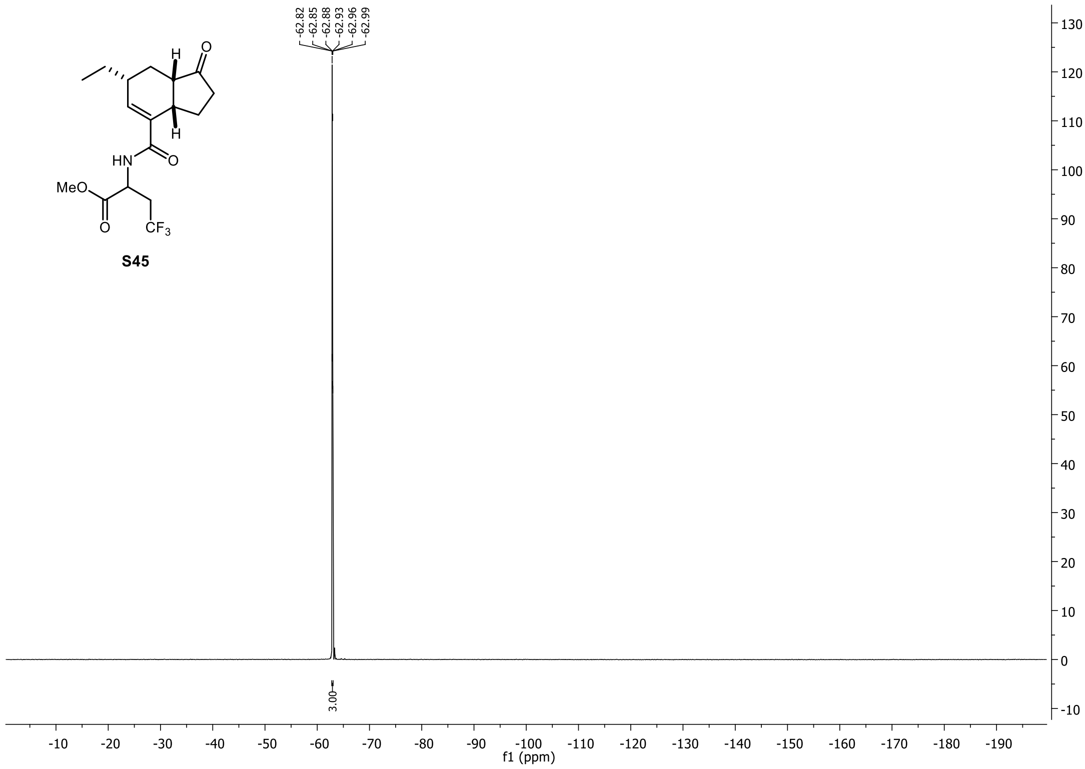

Supplementary Figure 110: ^{1}H NMR 26.

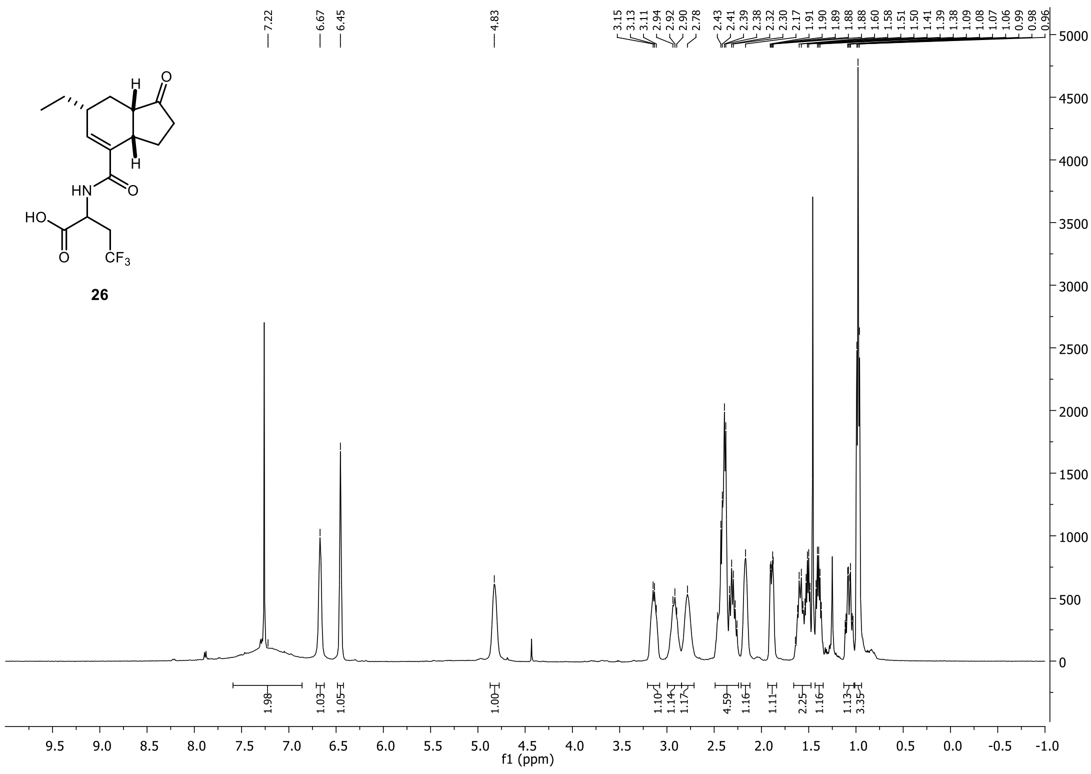

Supplementary Figure 111: ^{13}C NMR 26.

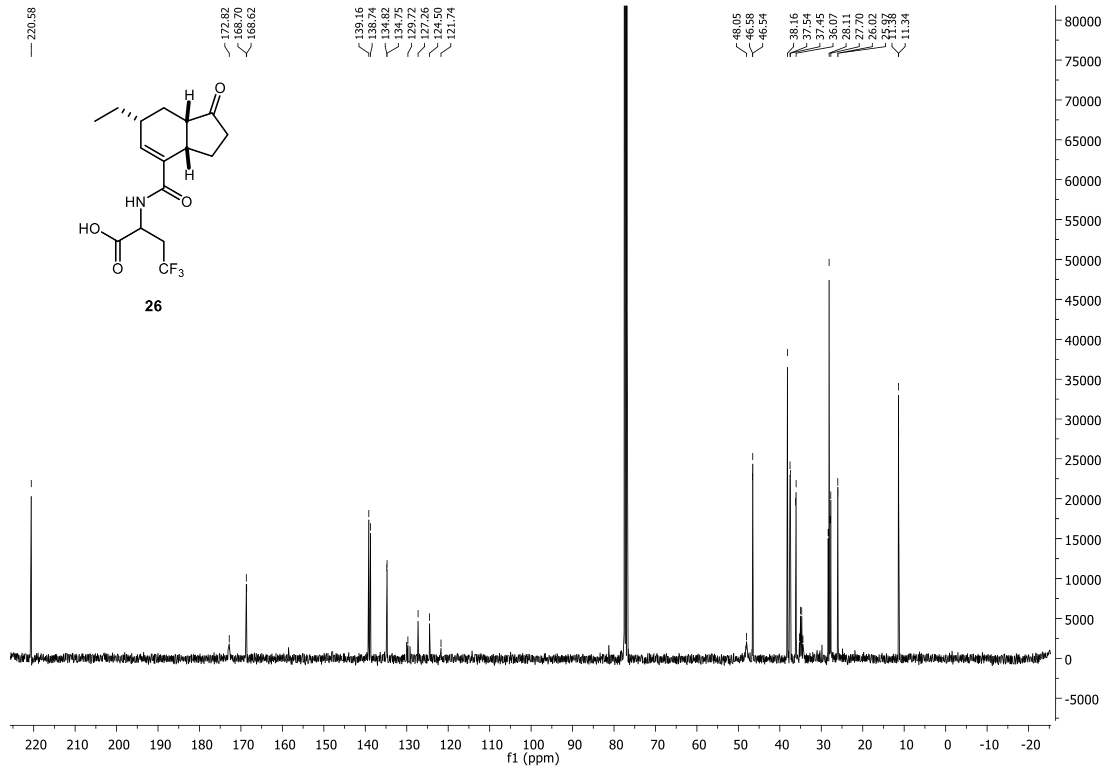

Supplementary Figure 112: ^{19}F NMR 26.

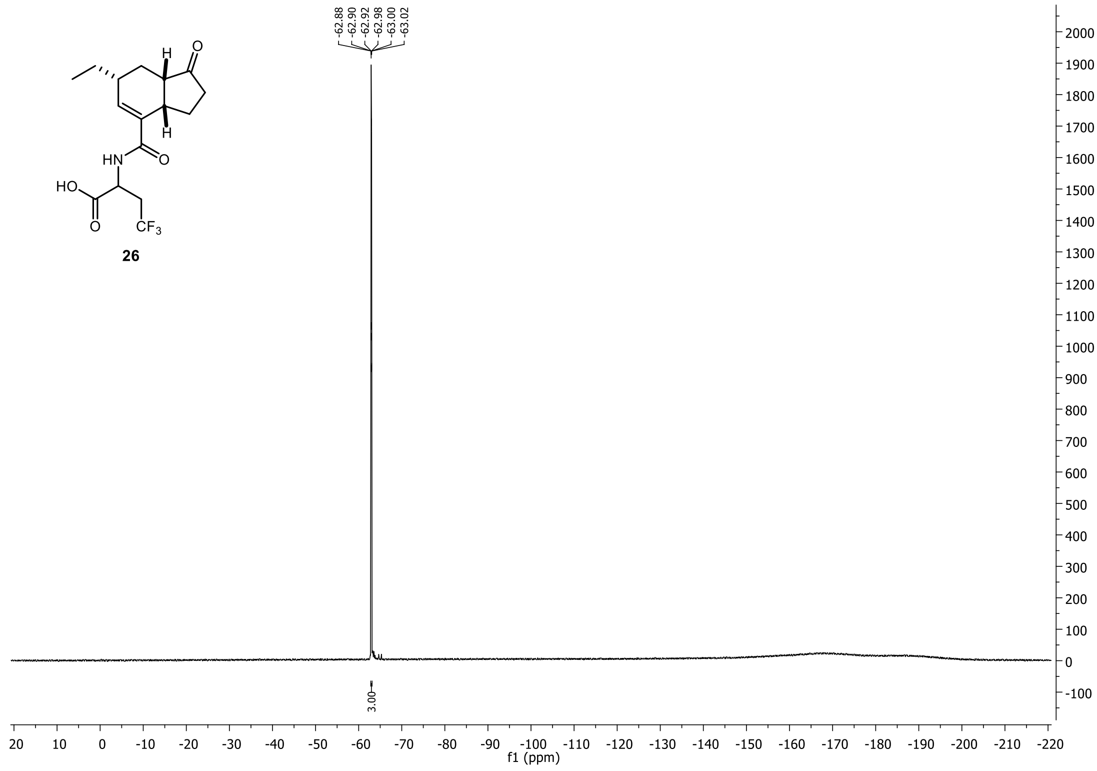

Supplementary Figure 113: ^{1}H NMR 27.

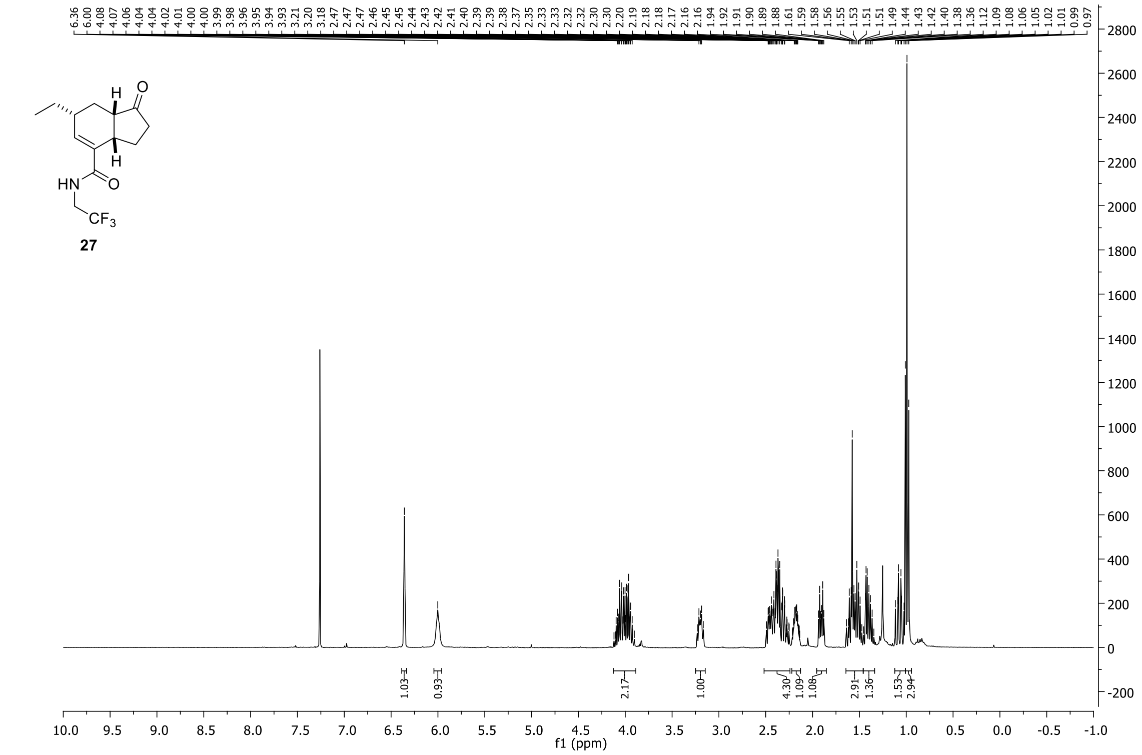

Supplementary Figure 114: ^{13}C NMR 27.

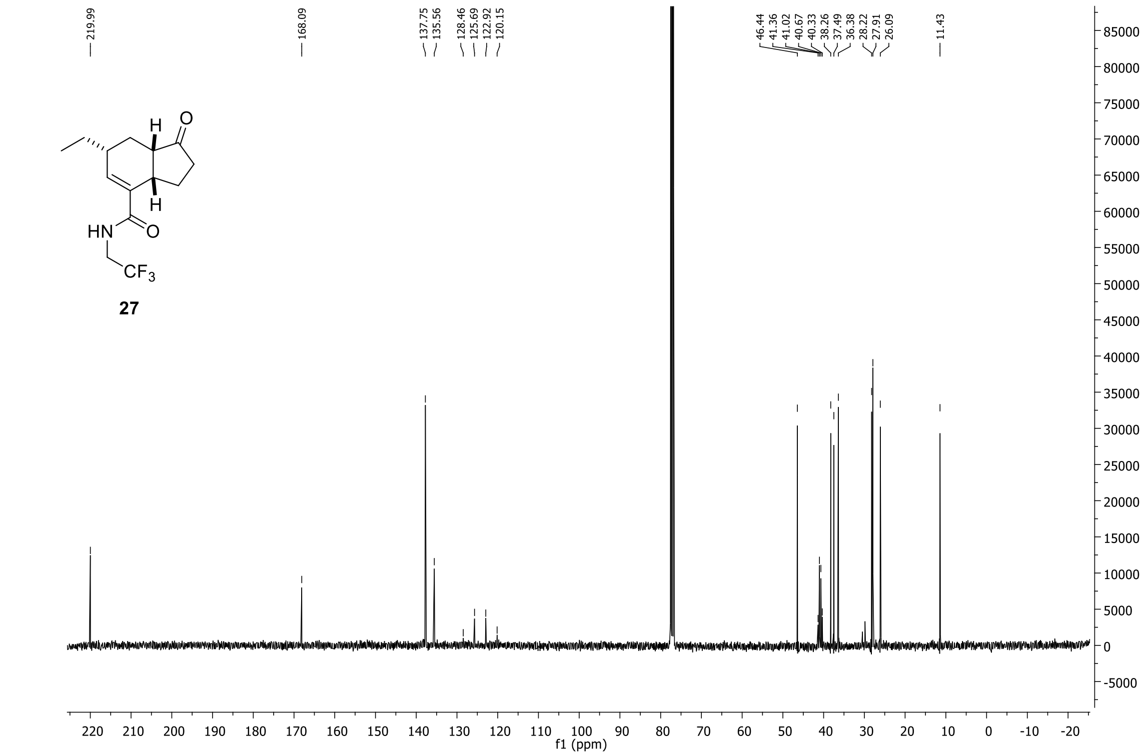

Supplementary Figure 115: ^{19}F NMR 27.

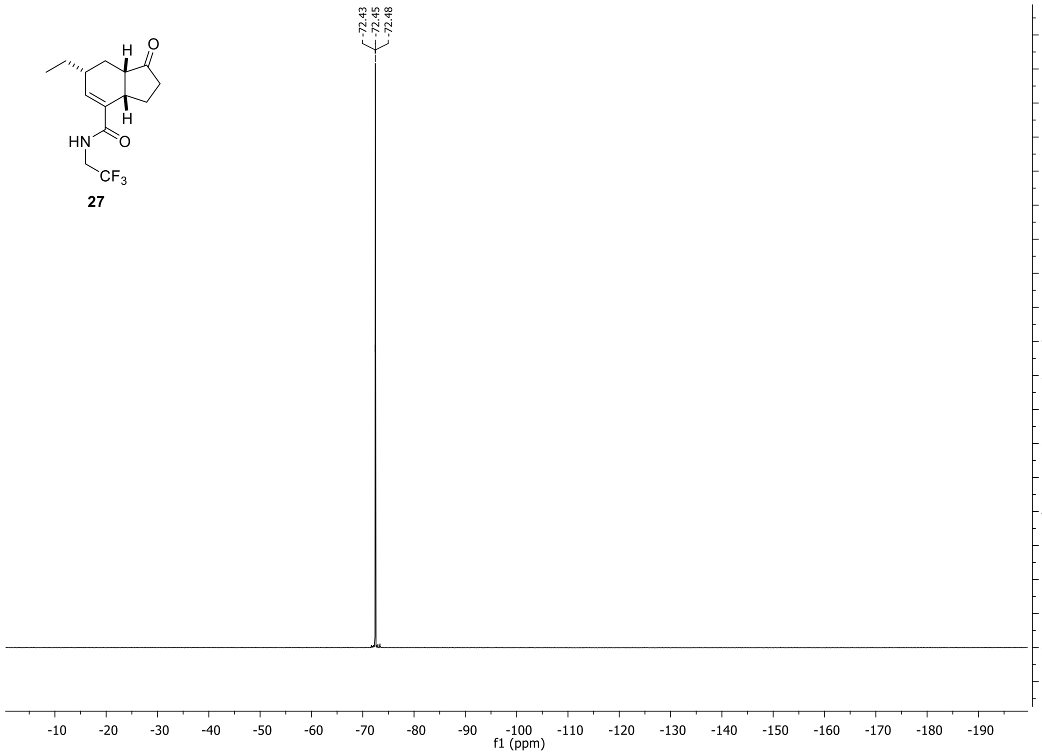

Supplementary Figure 116: ^{1}H NMR S46.

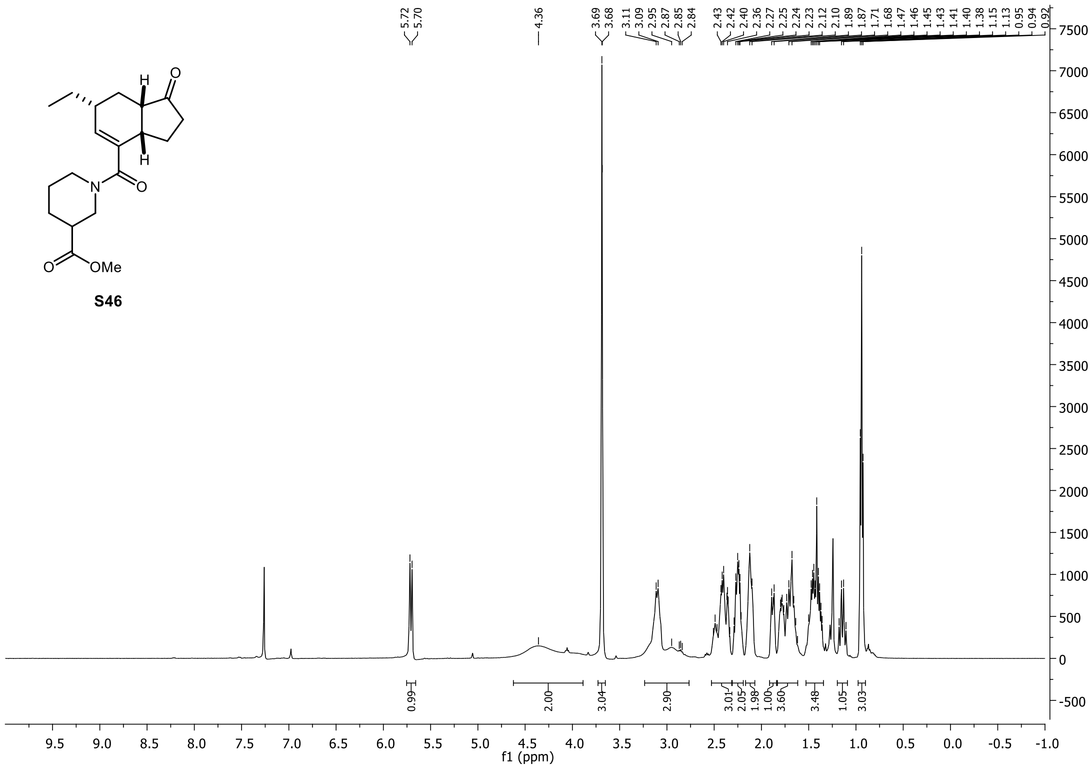

Supplementary Figure 117: ^{13}C NMR S46.

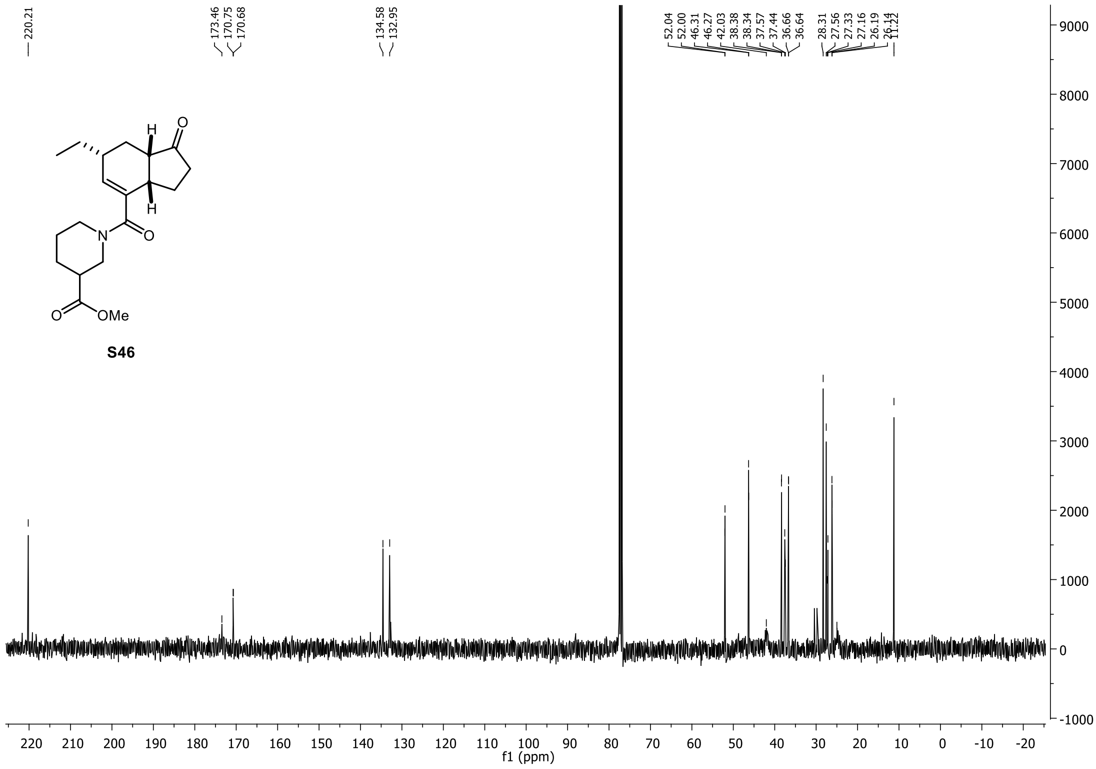

Supplementary Figure 118: ^{1}H NMR 28.

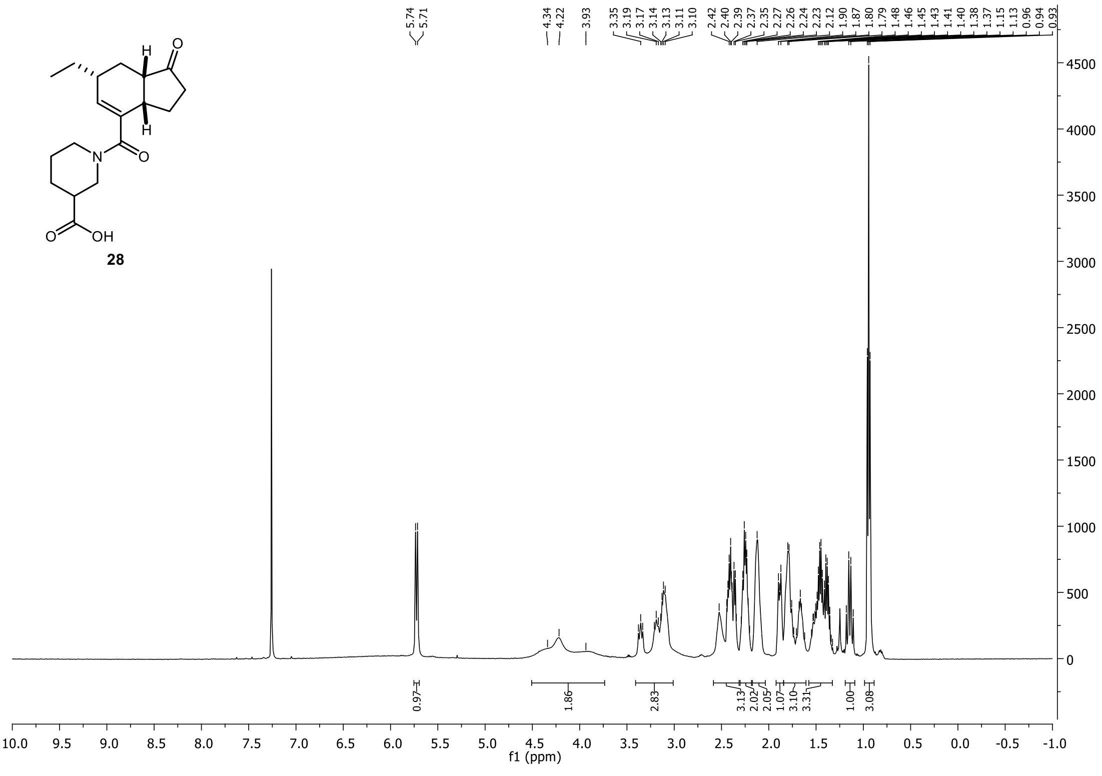

Supplementary Figure 119: ^{13}C NMR 28.

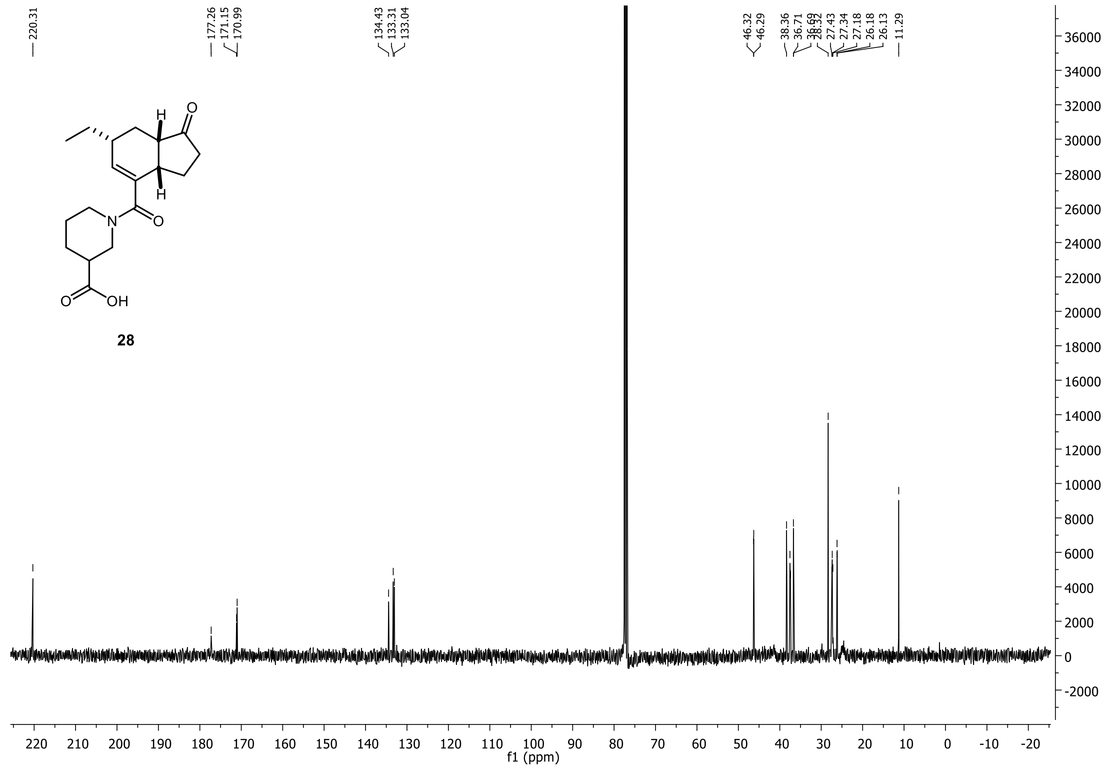

Supplementary Figure 120: ^{1}H NMR S47.

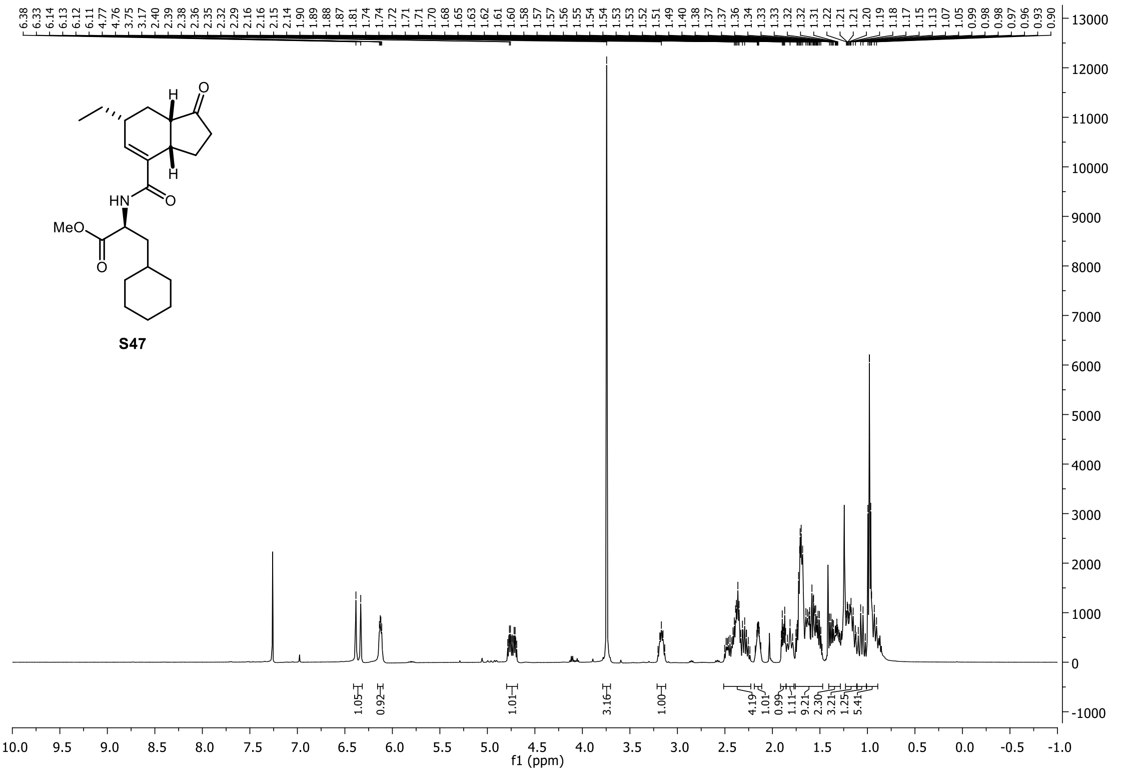

Supplementary Figure 121: ^{13}C NMR S47.

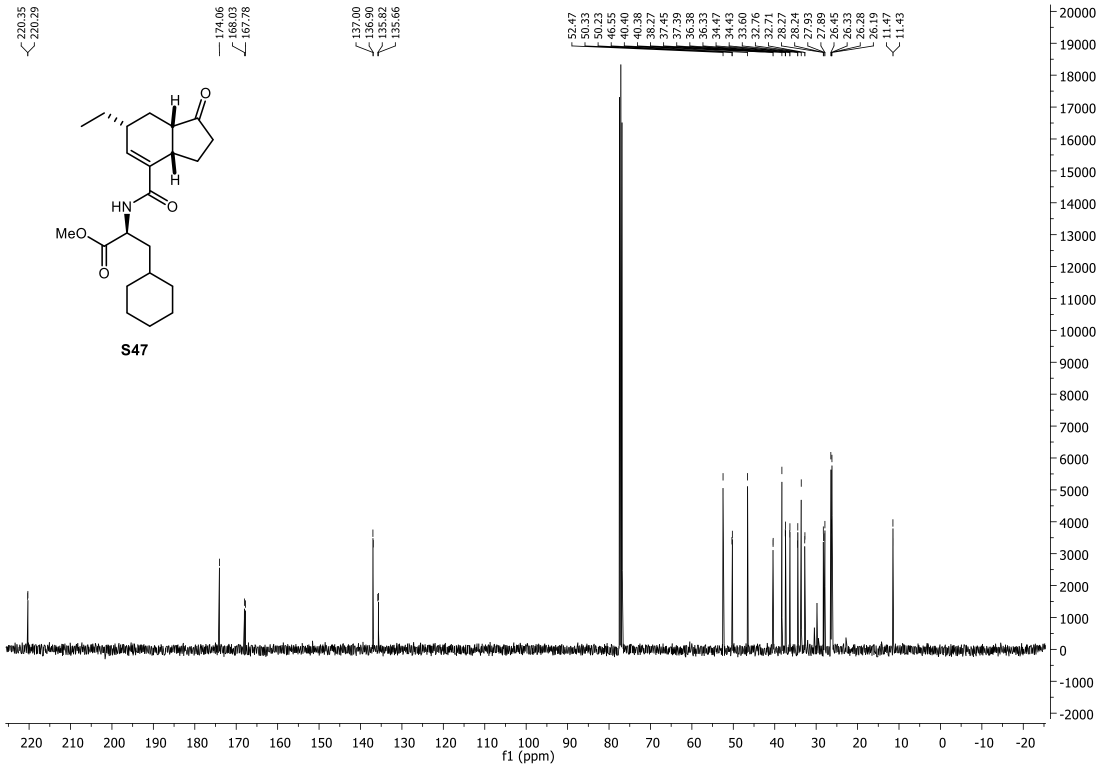

Supplementary Figure 122 ^{1}H NMR 29.

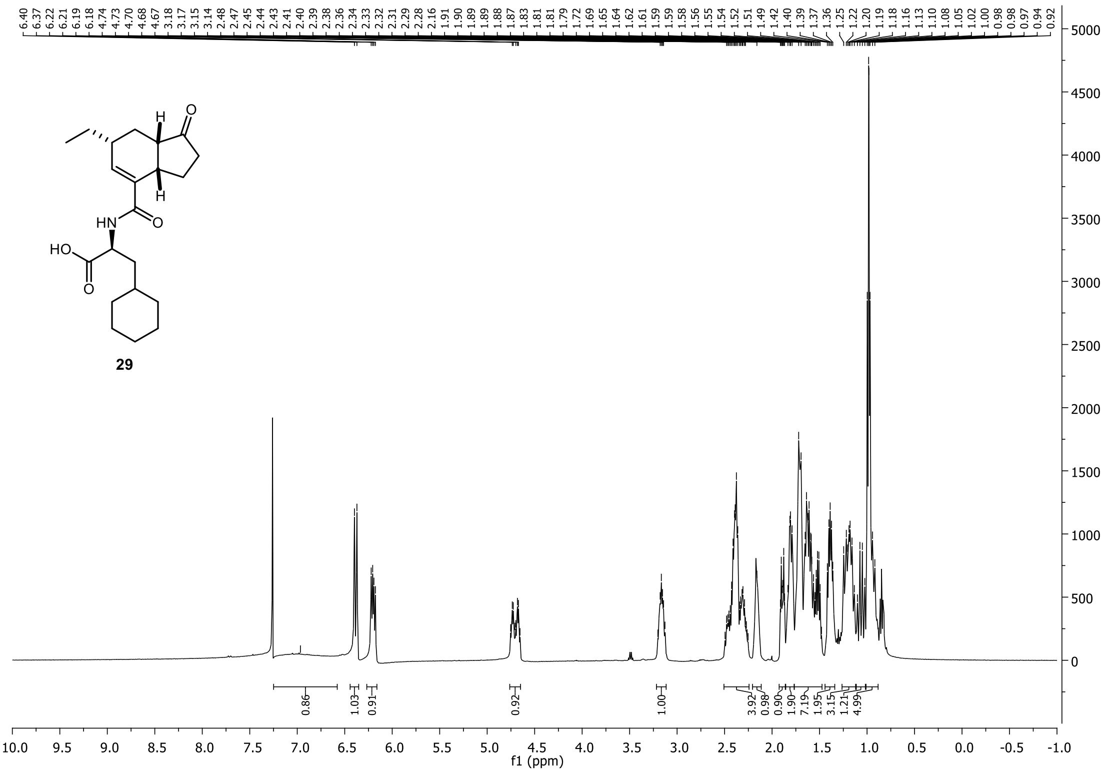

Supplementary Figure 123: ^{13}C NMR 29.

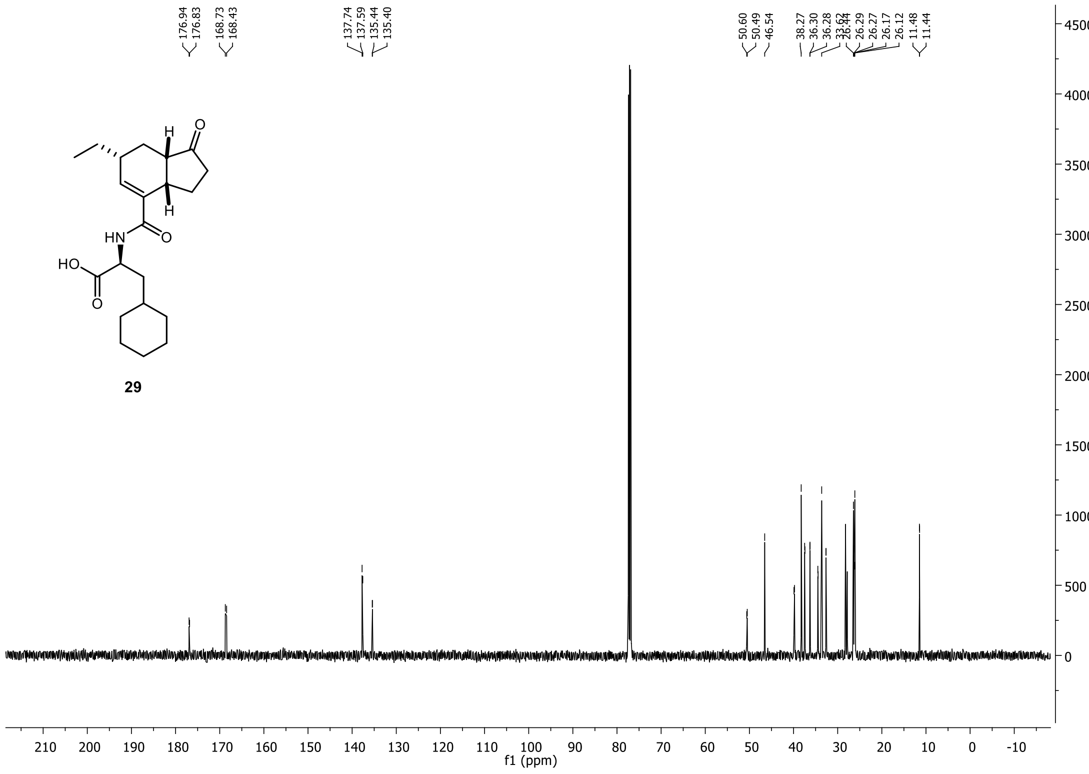

Supplementary Figure 124: ^{1}H NMR S48.

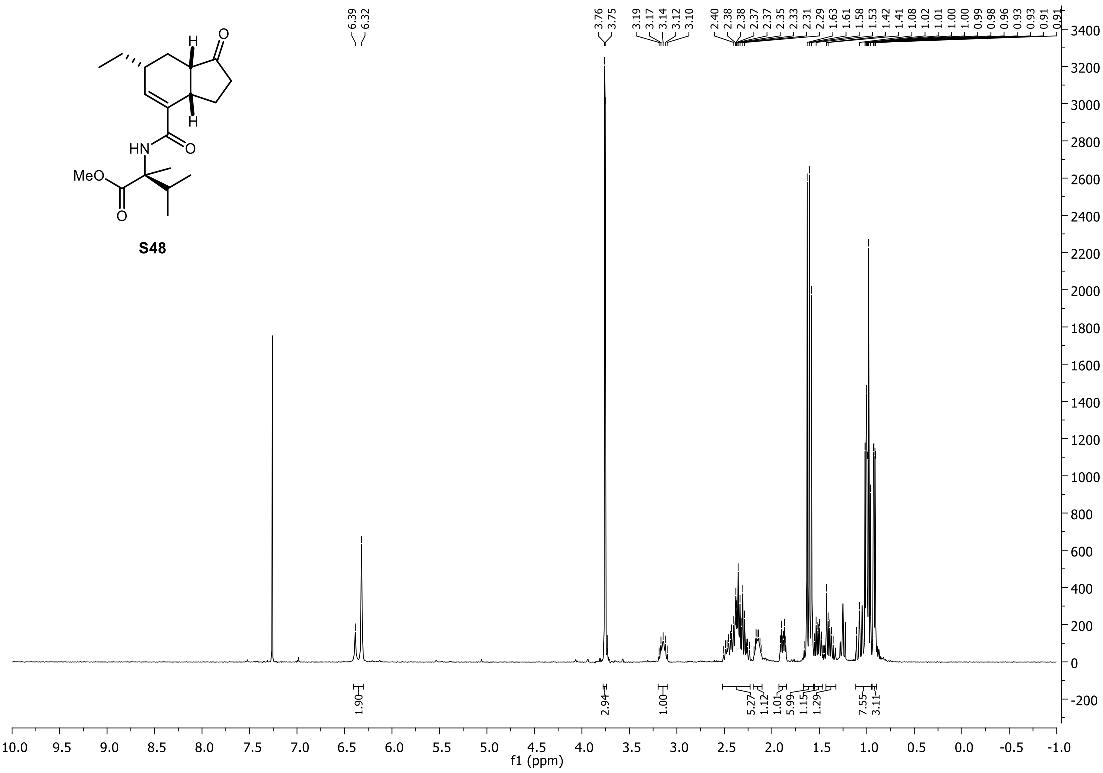

Supplementary Figure 125: ^{13}C NMR S48.

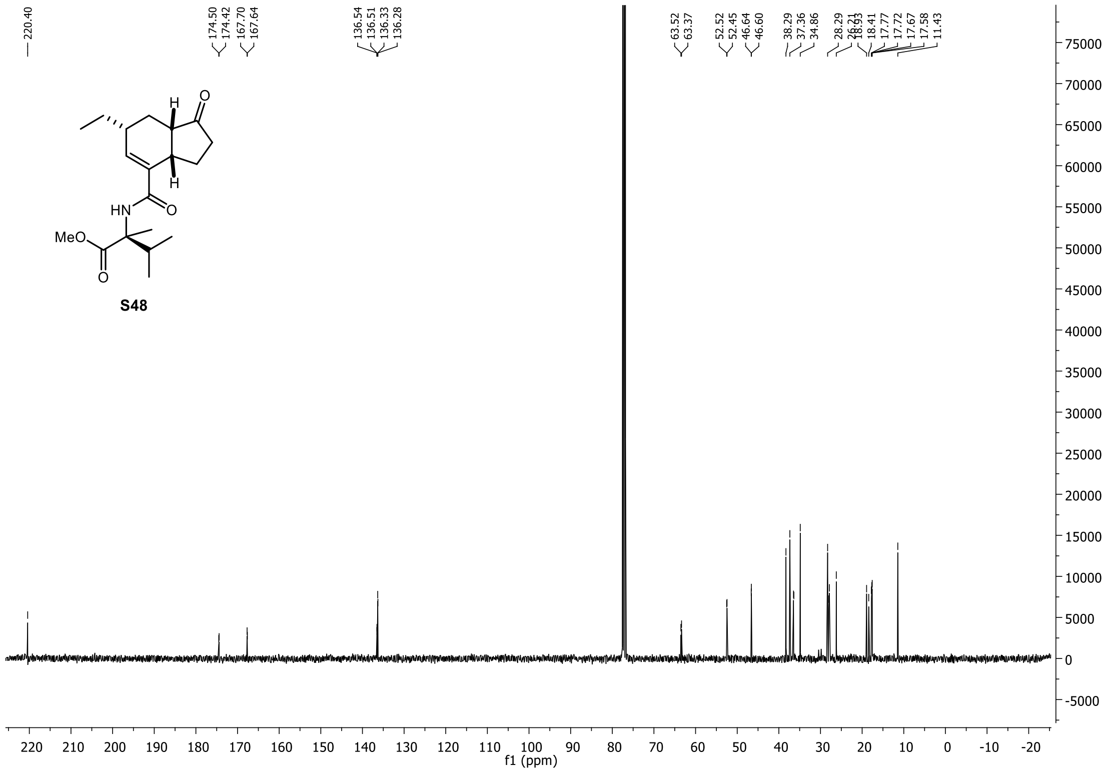

Supplementary Figure 126: ^{1}H NMR 30.

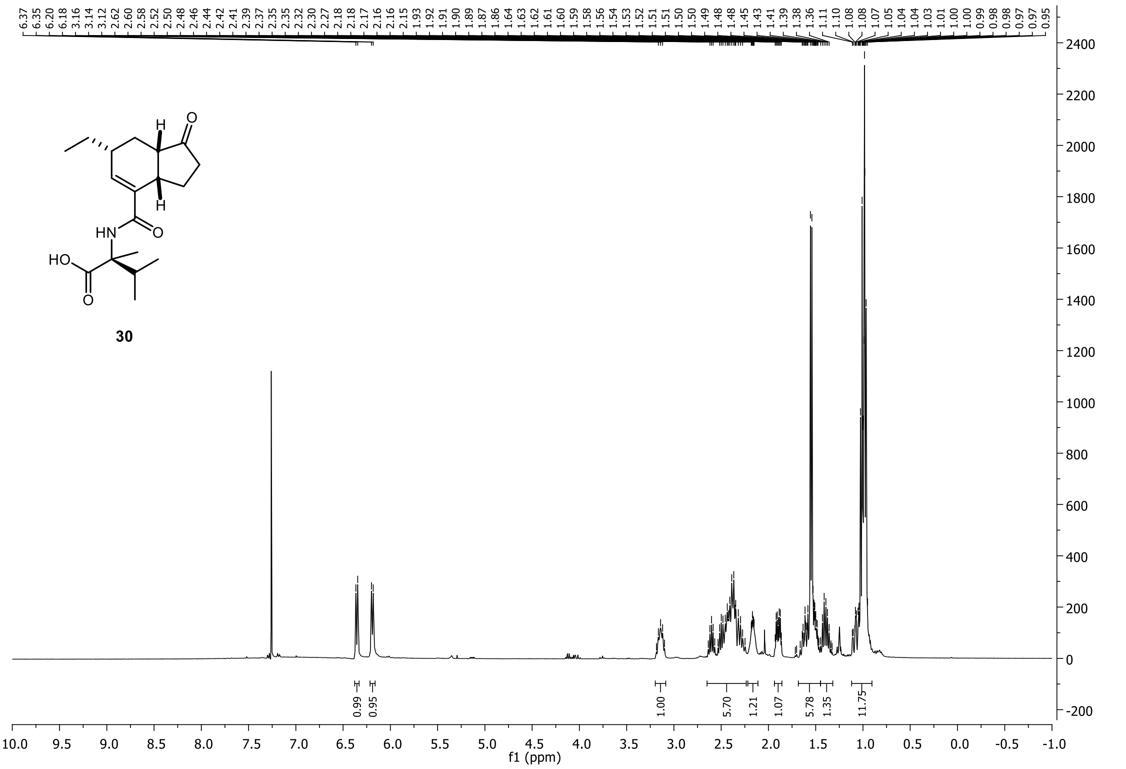

Supplementary Figure 127: ^{13}C NMR 30.

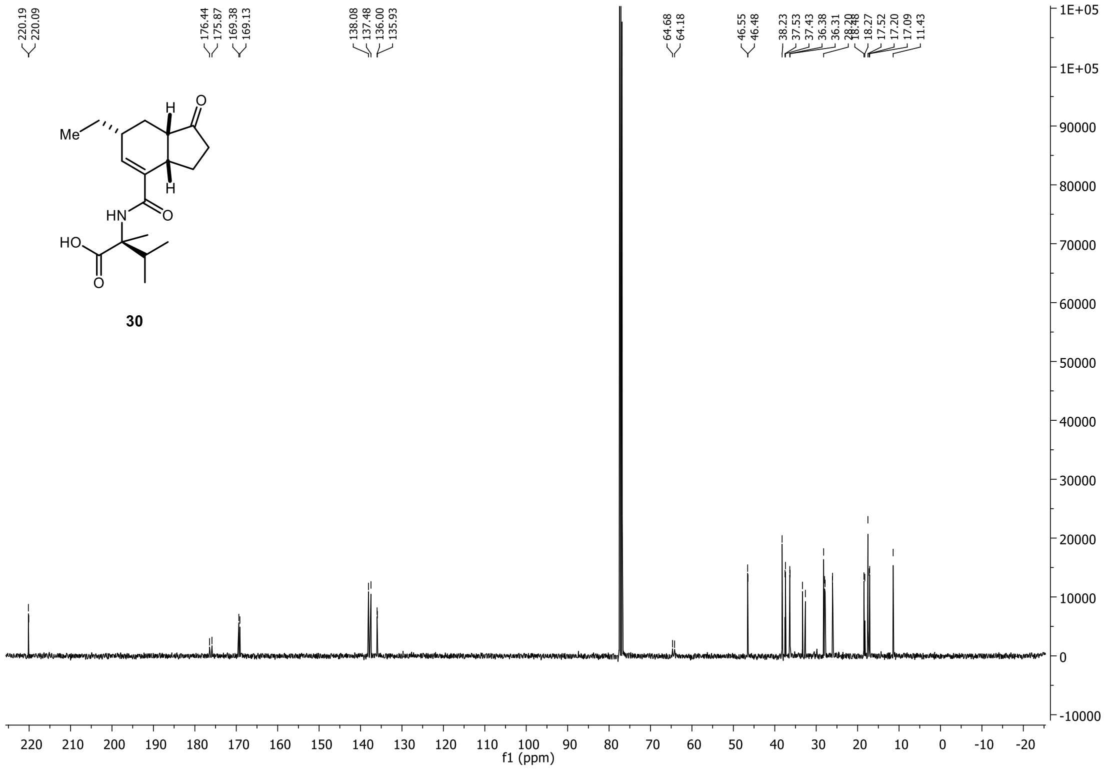

Supplementary Figure 128: ^{1}H NMR 31.

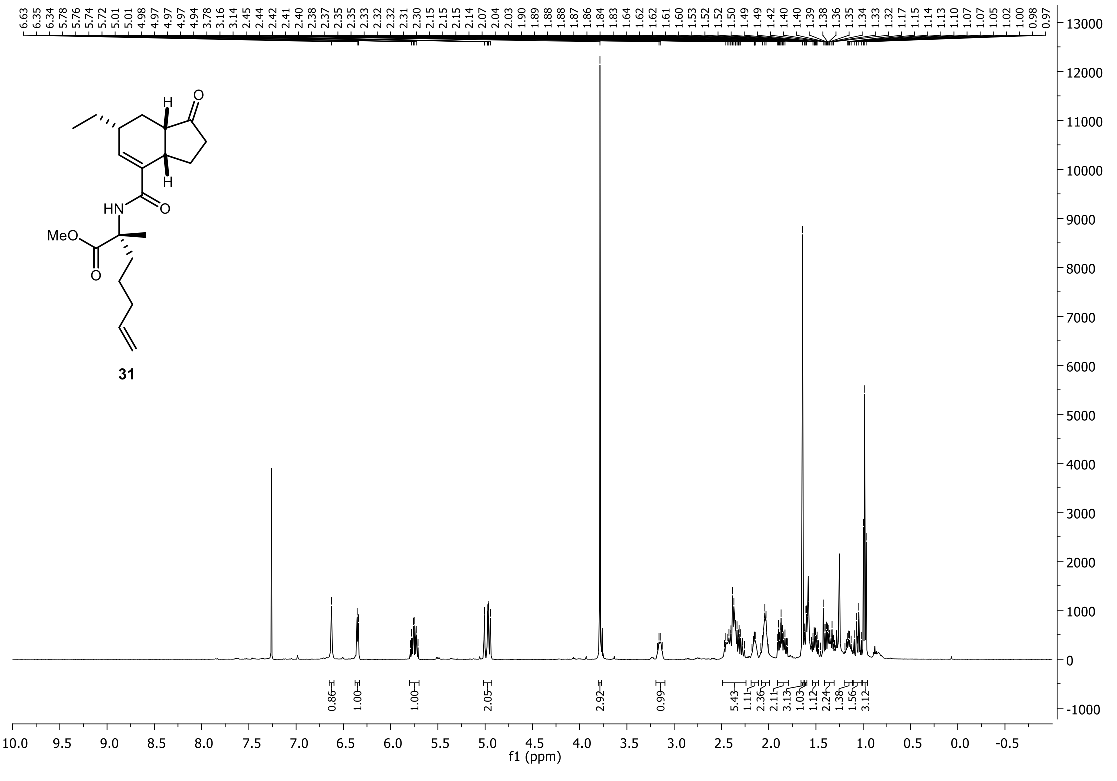

Supplementary Figure 129: ^{13}C NMR 31.

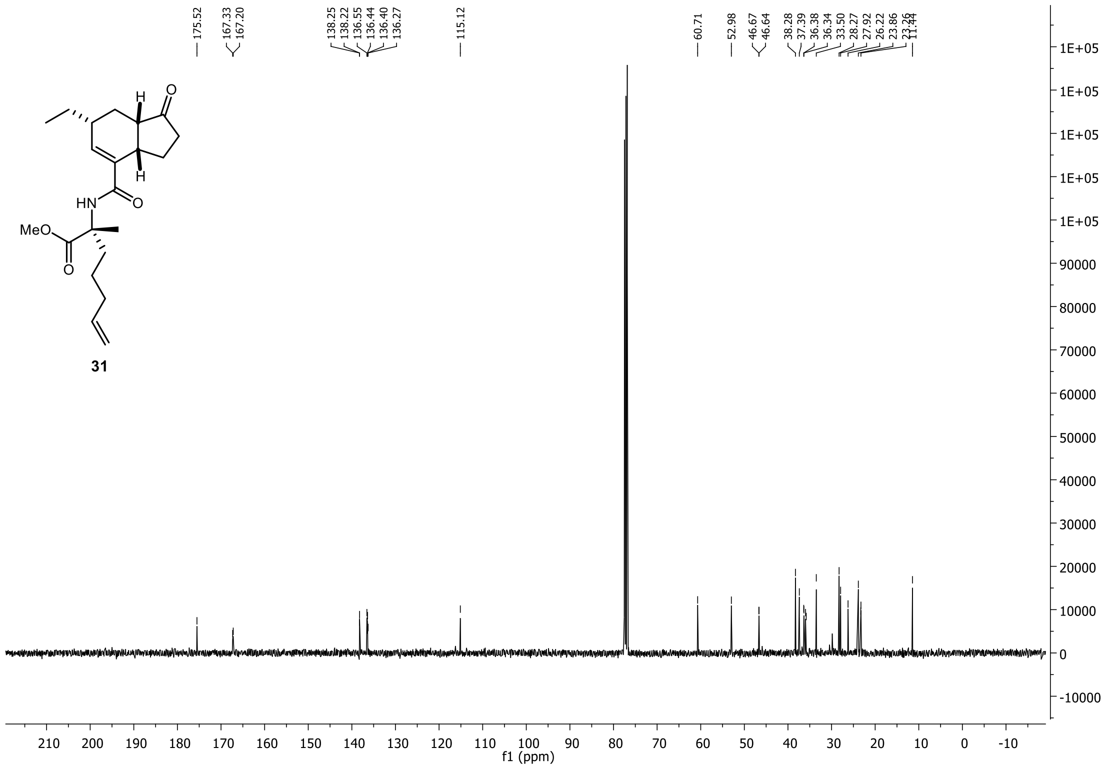

Supplementary Figure 130: ^{1}H NMR 32.

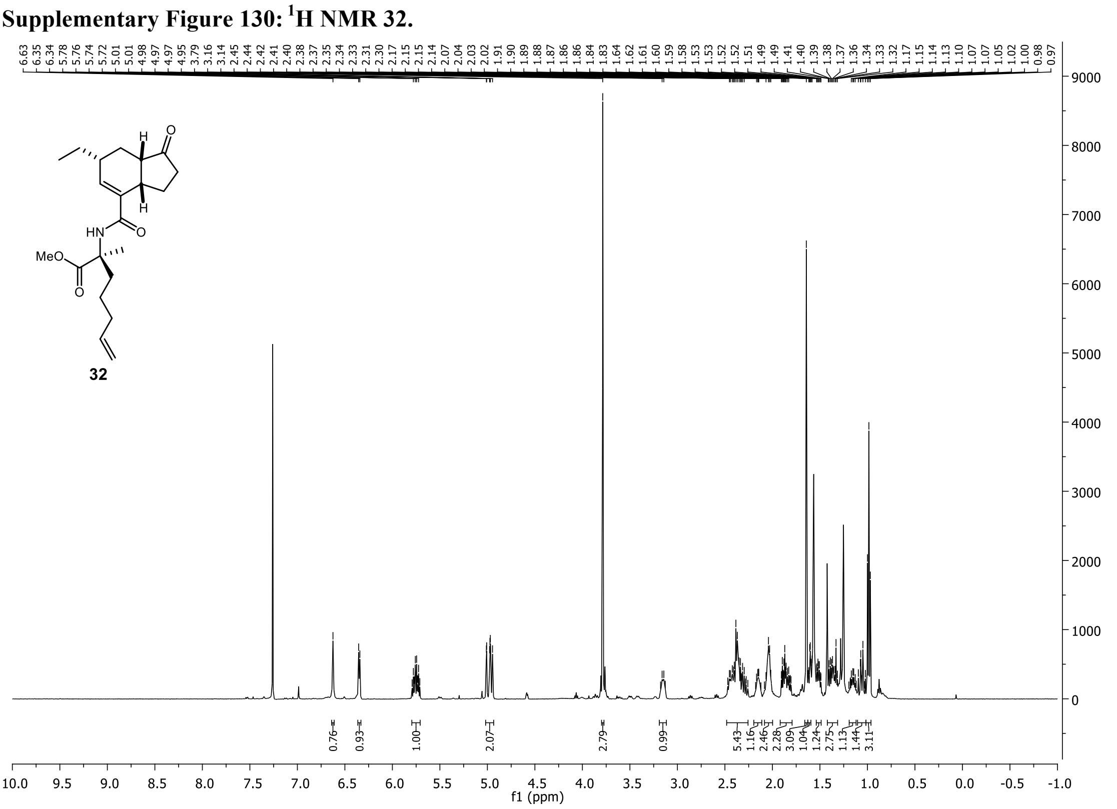

Supplementary Figure 131: ^{13}C NMR 32.

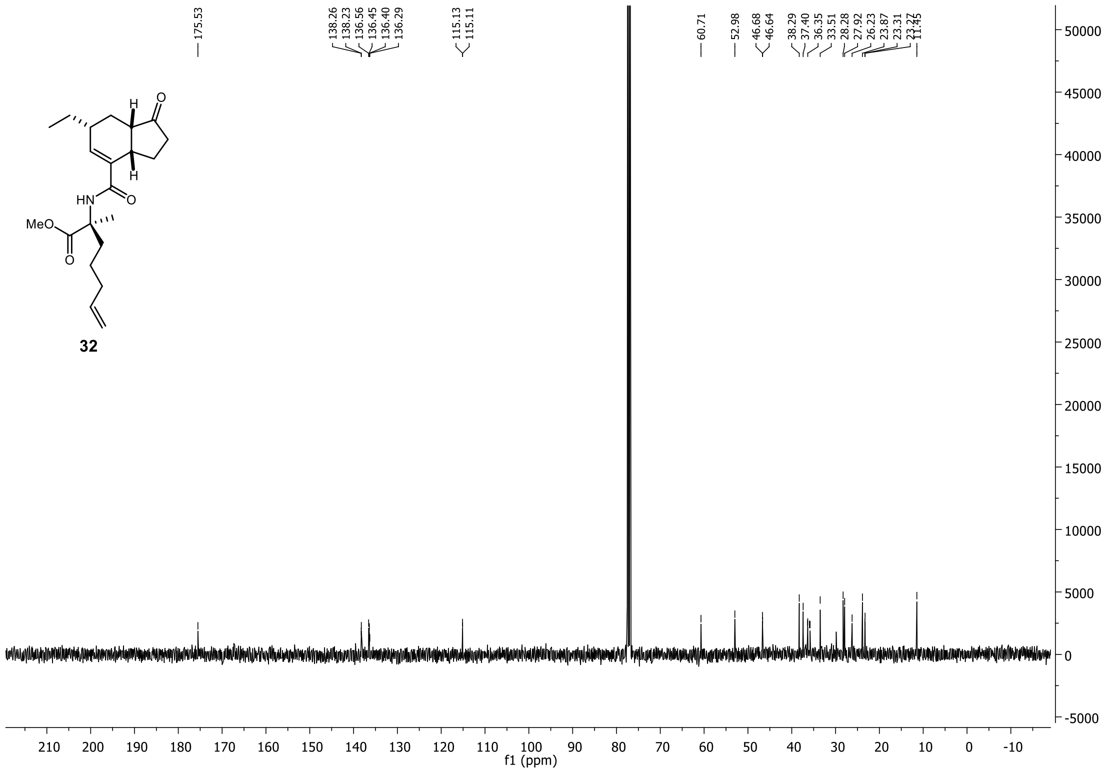

Supplementary Figure 132: ^{1}H NMR S49.

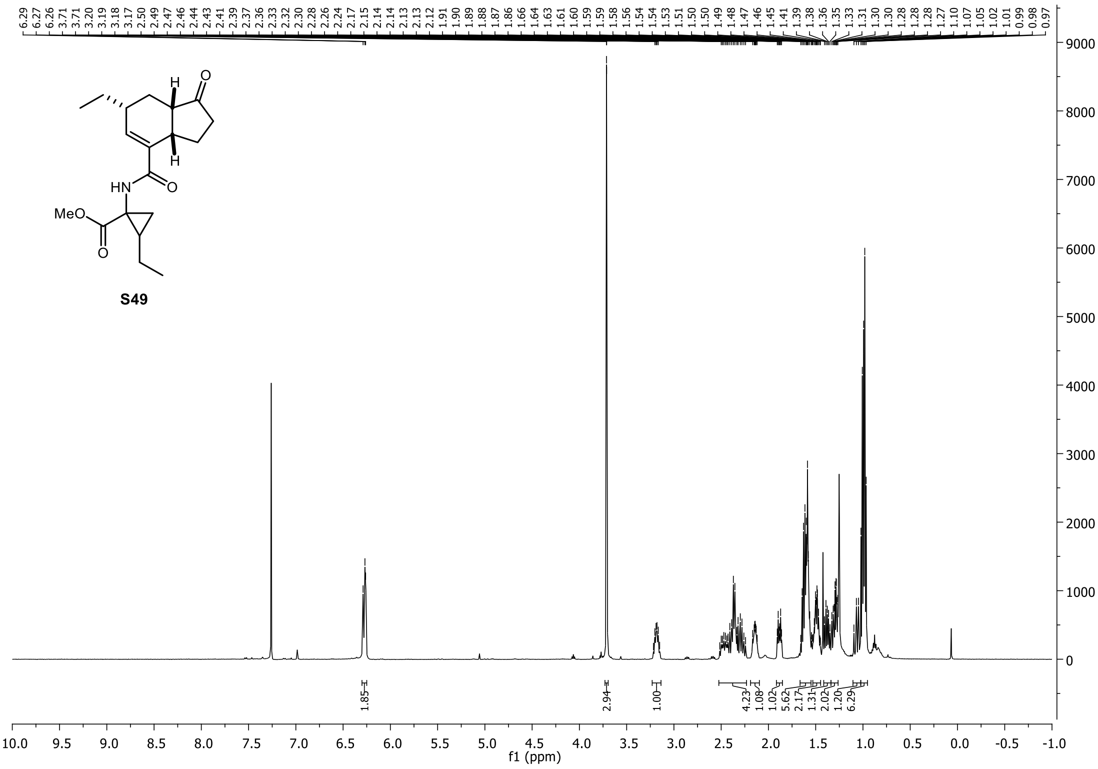

Supplementary Figure 133: ^{13}C NMR S49.

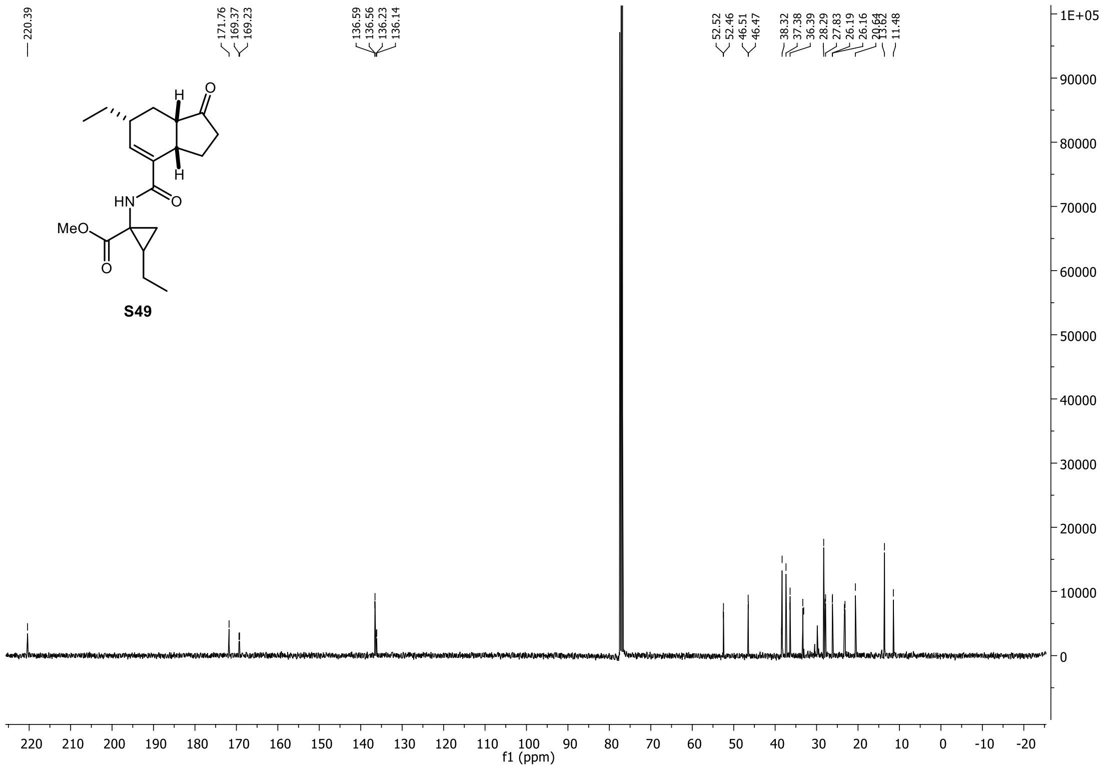

Supplementary Figure 134: ^{1}H NMR 33.

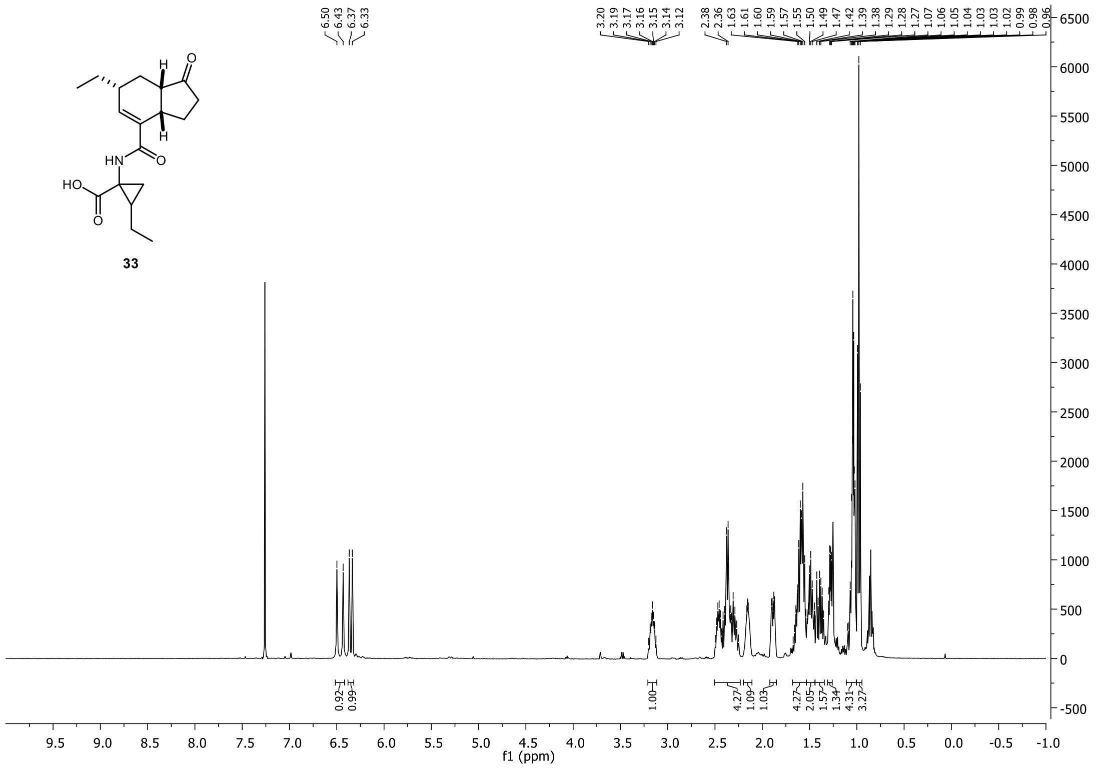

Supplementary Figure 135: ^{13}C NMR 33.

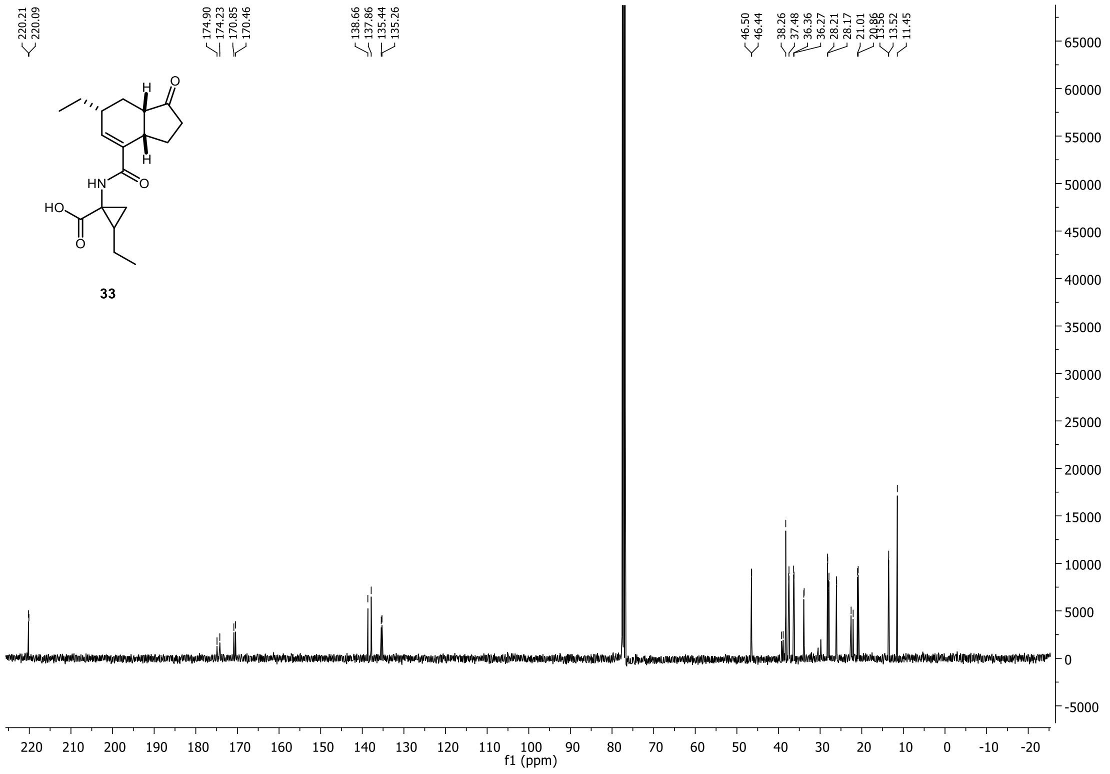

Supplementary Figure 136: ^{1}H NMR S50.

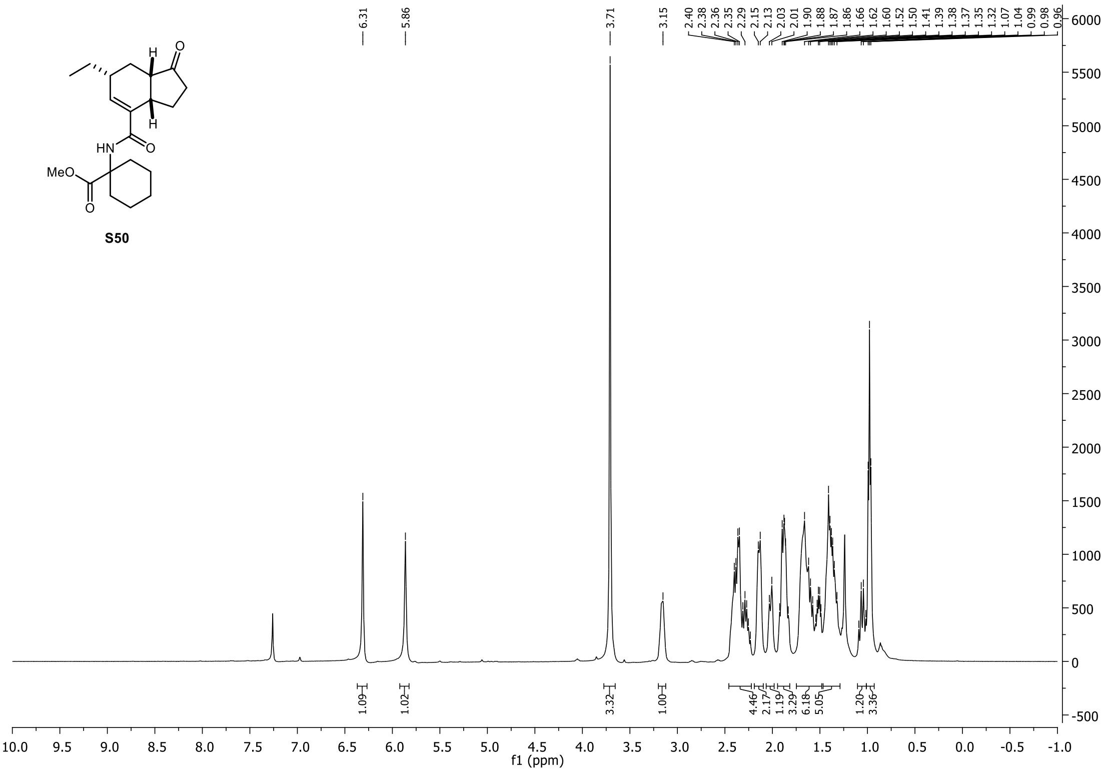

Supplementary Figure 137: ^{13}C NMR S50.

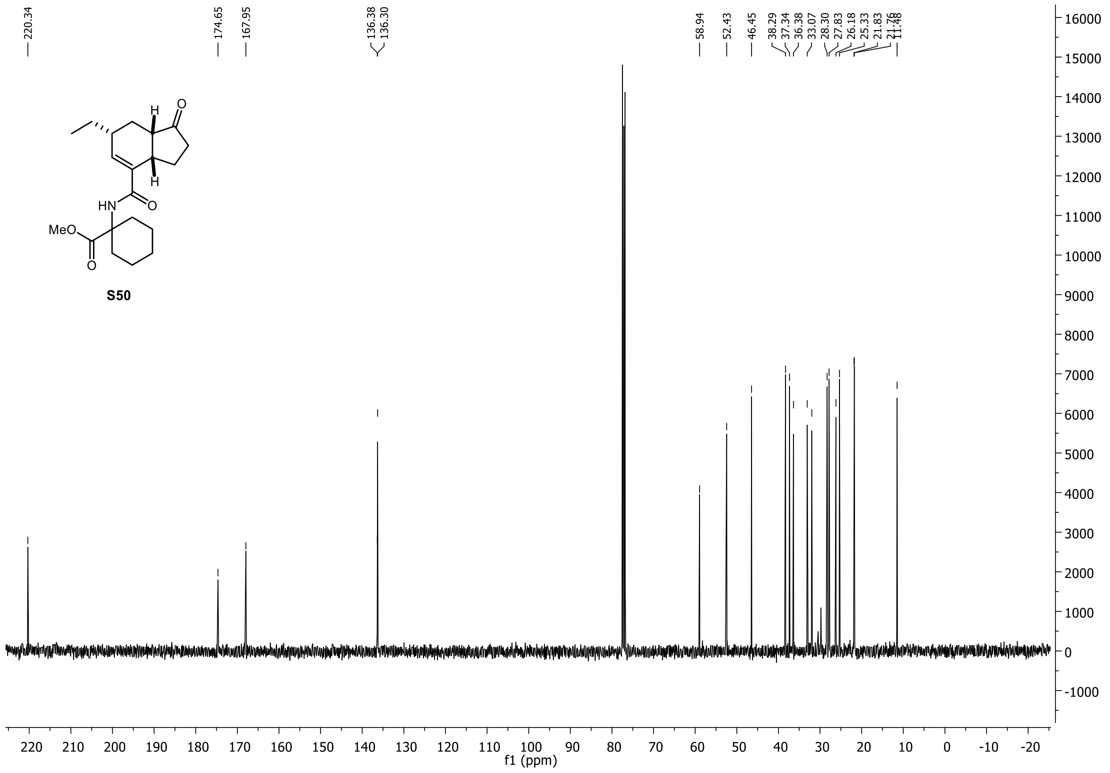

Supplementary Figure 138: ^{1}H NMR 34.

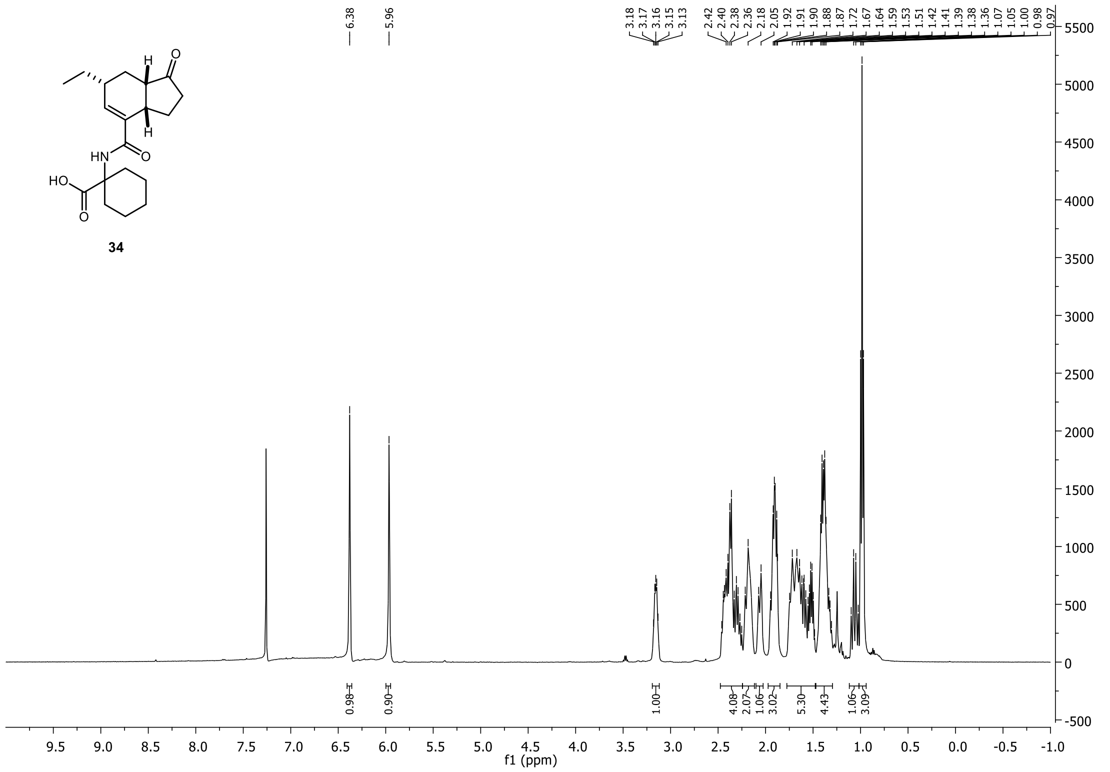

Supplementary Figure 139: ^{13}C NMR 34.

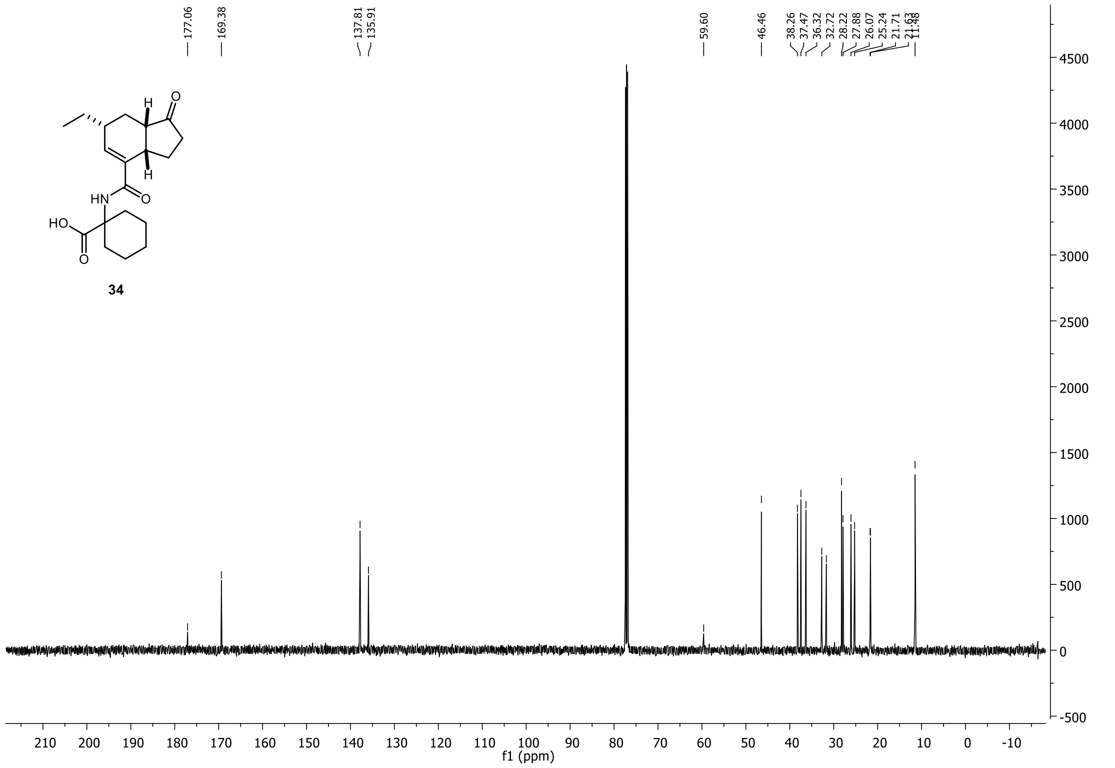

Supplementary Figure 140: ^{1}H NMR S51.

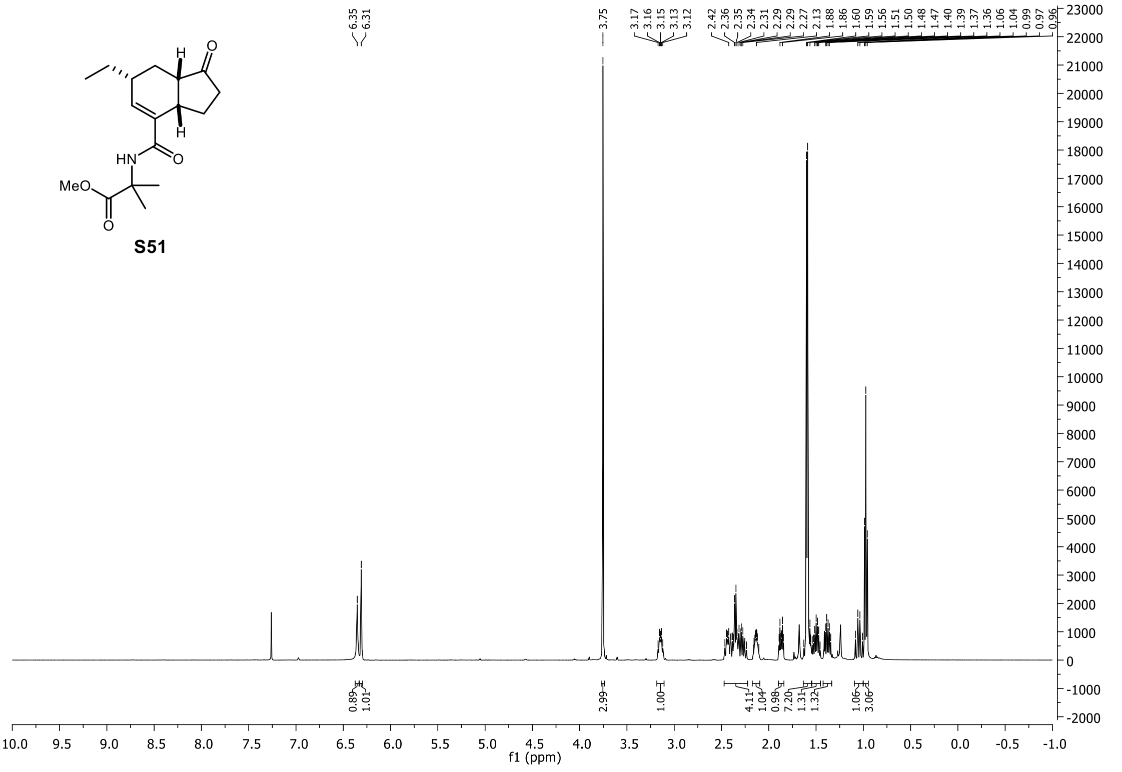

Supplementary Figure 141: ^{13}C NMR S51.

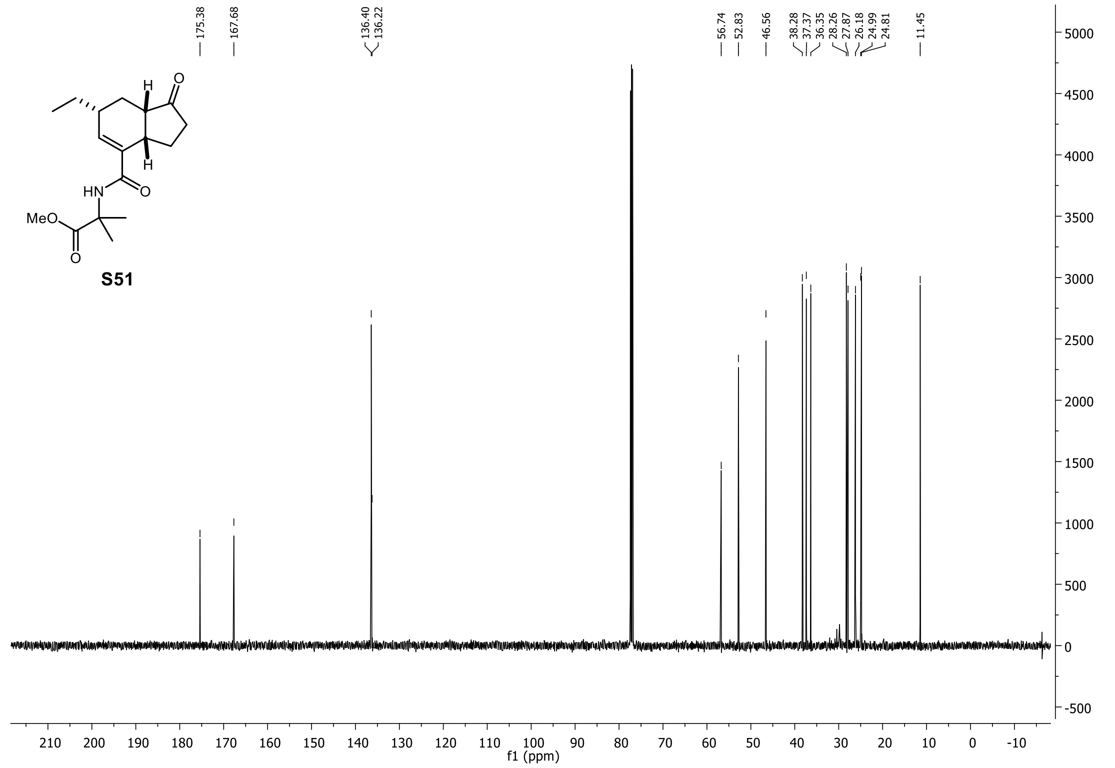

Supplementary Figure 142: ^{1}H NMR 35.

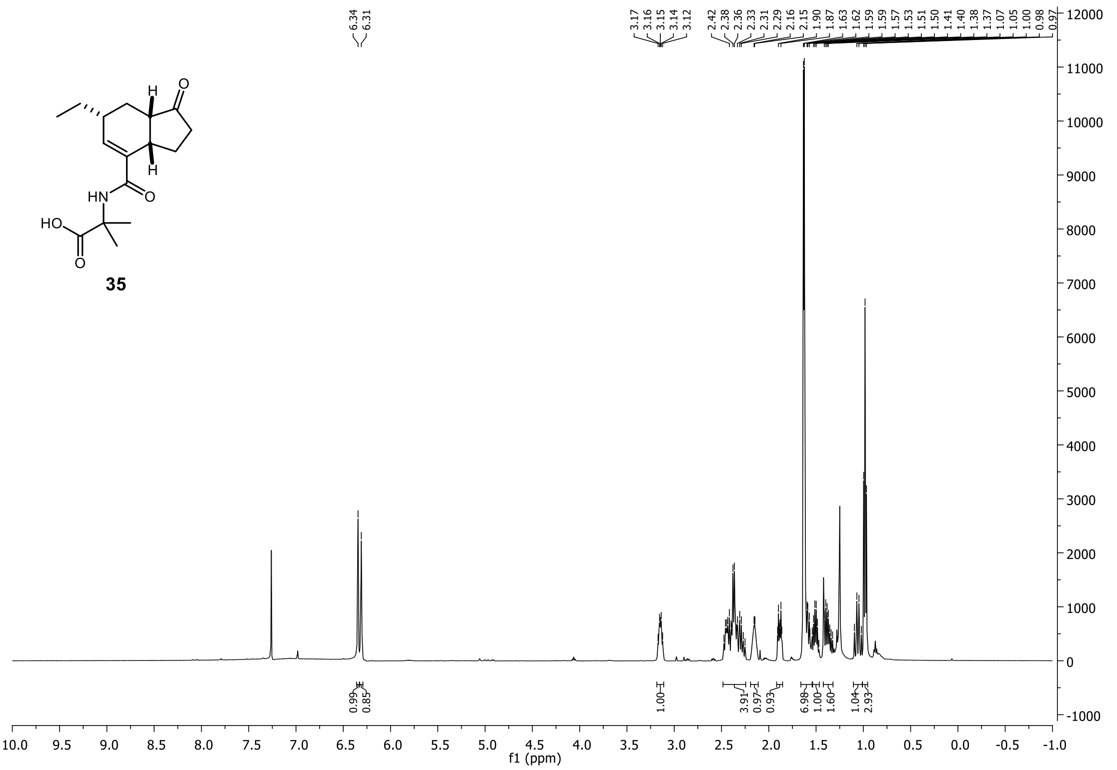

Supplementary Figure 143: ^{13}C NMR 35.

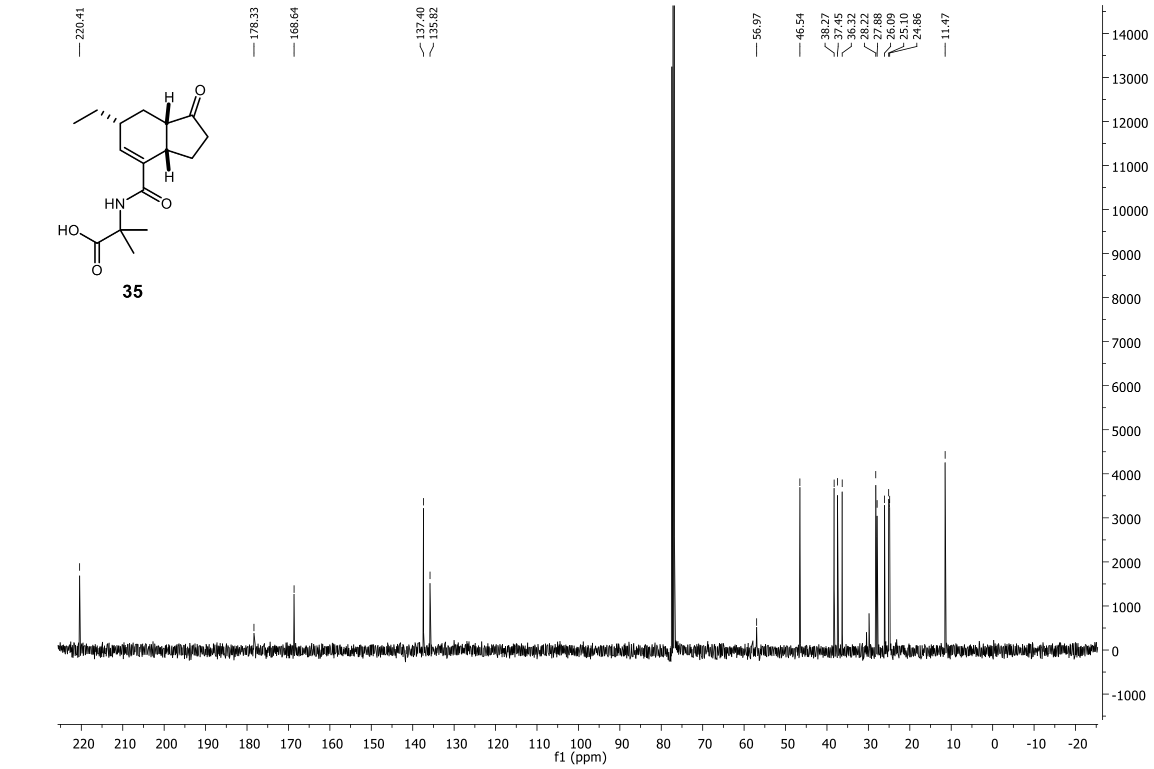

Supplementary Figure 144: ^{1}H NMR S52.

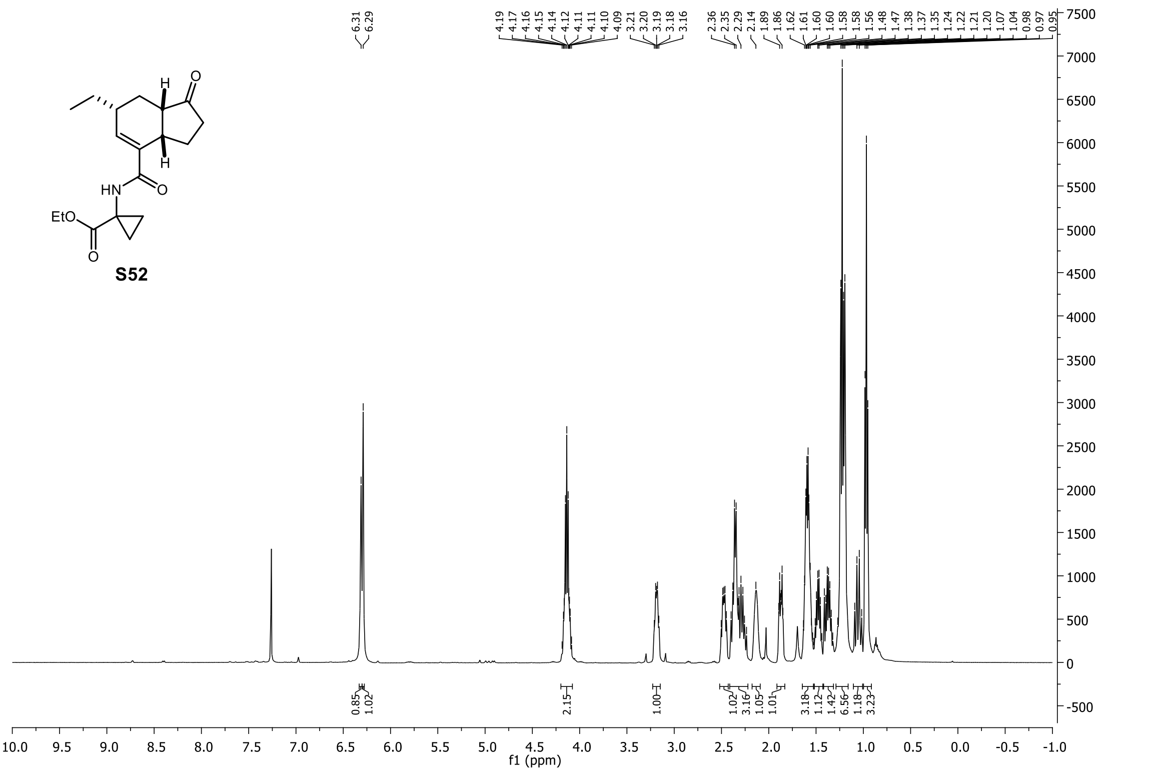

Supplementary Figure 145: ^{13}C NMR S52.

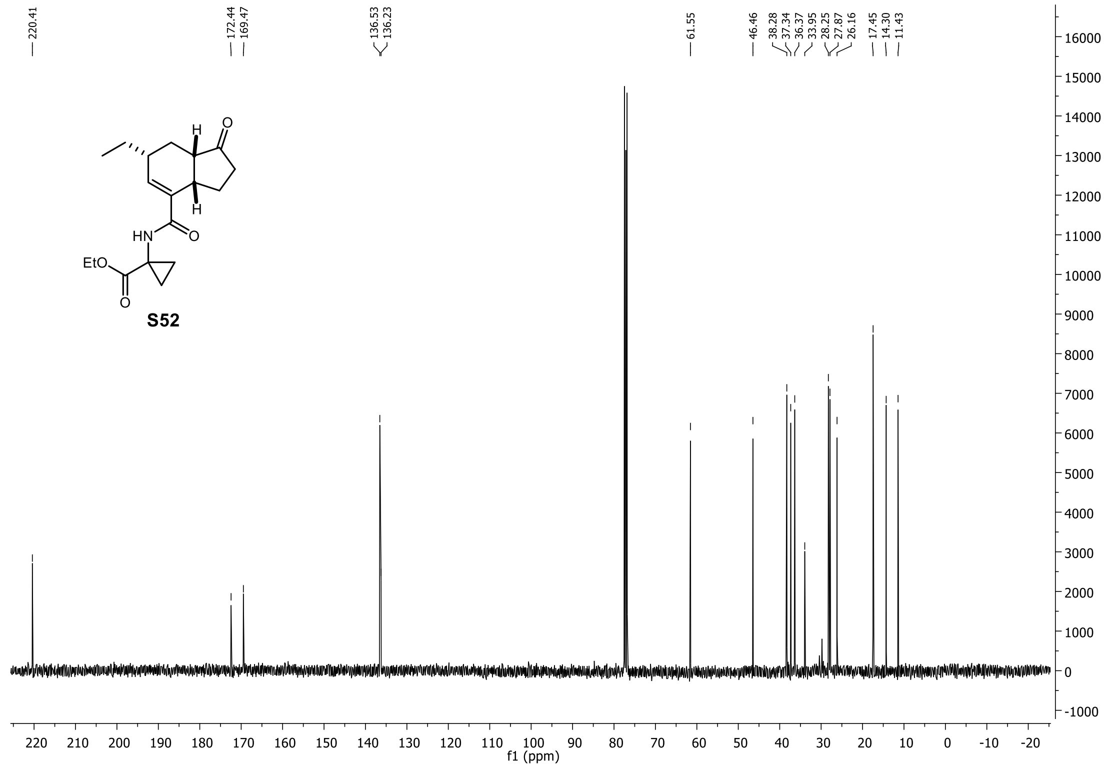

Supplementary Figure 146: ^{1}H NMR 36.

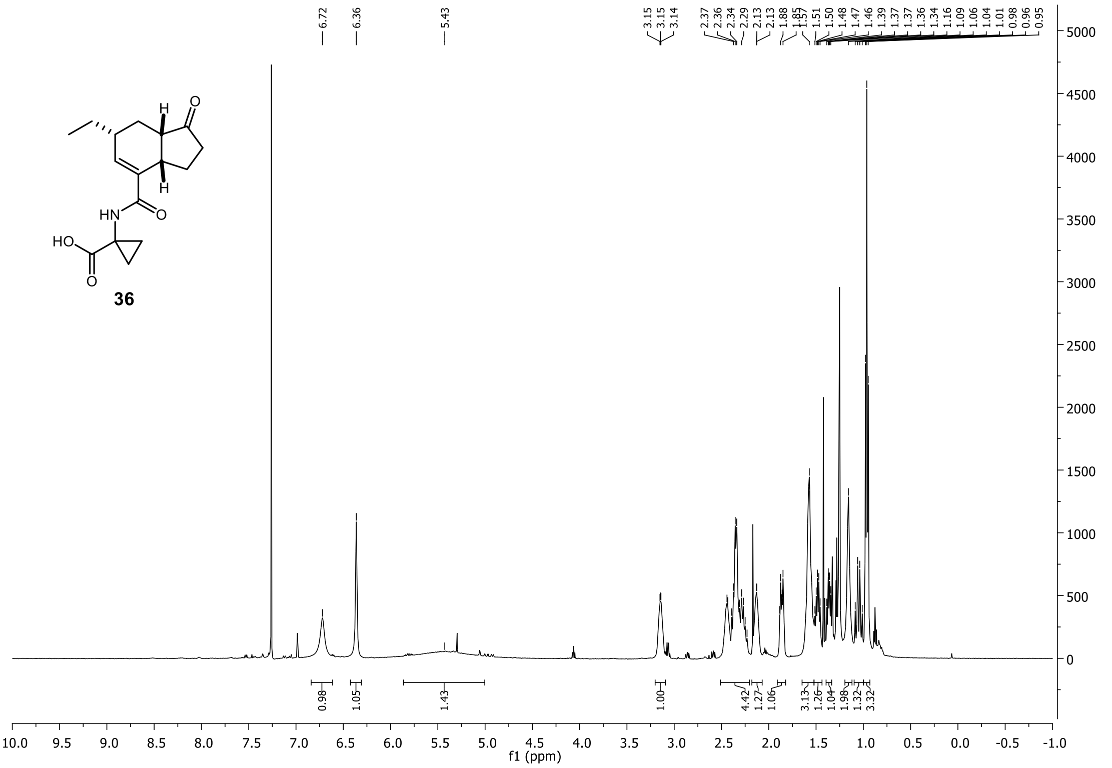

Supplementary Figure 147: ^{13}C NMR 36.

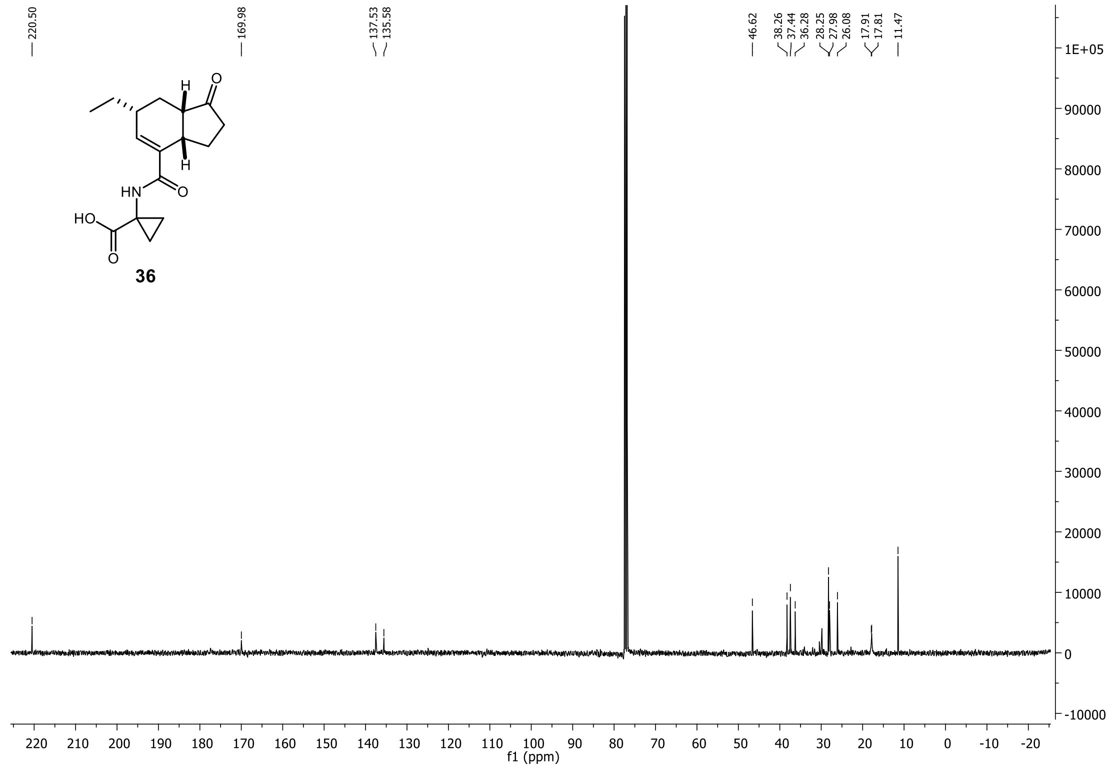

Supplementary Figure 148: ^{1}H NMR S53.

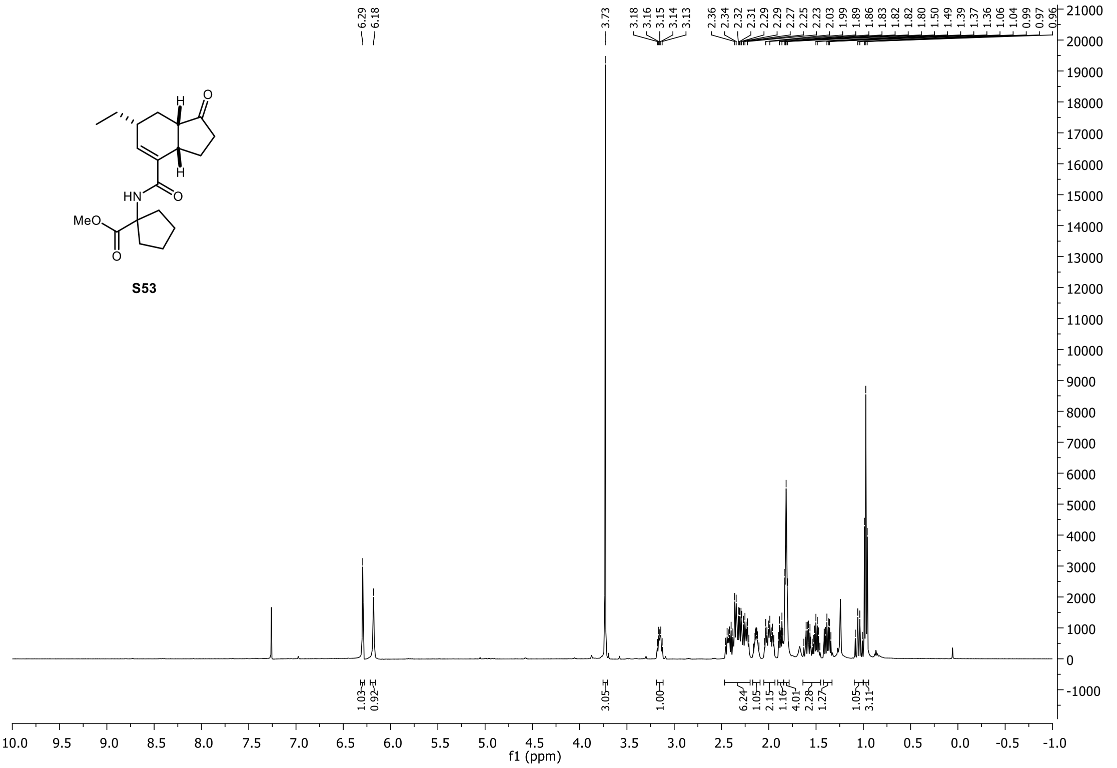

Supplementary Figure 149: ^{13}C NMR S53.

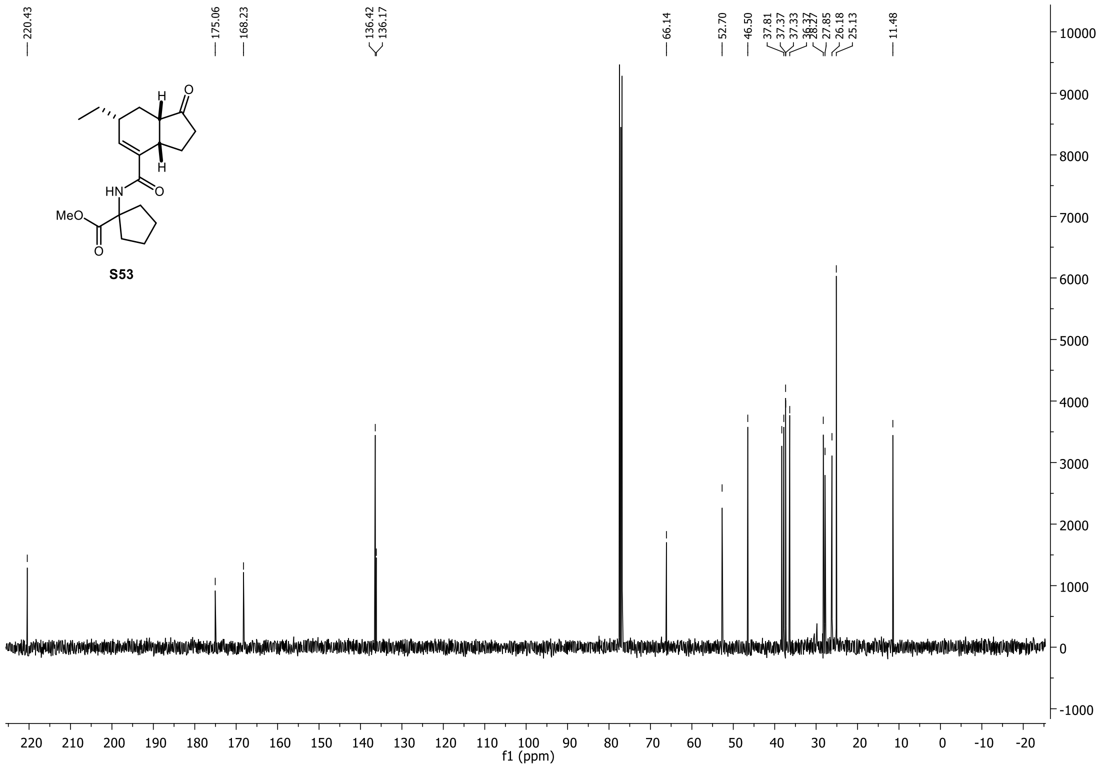

Supplementary Figure 150: ^{1}H NMR 37.

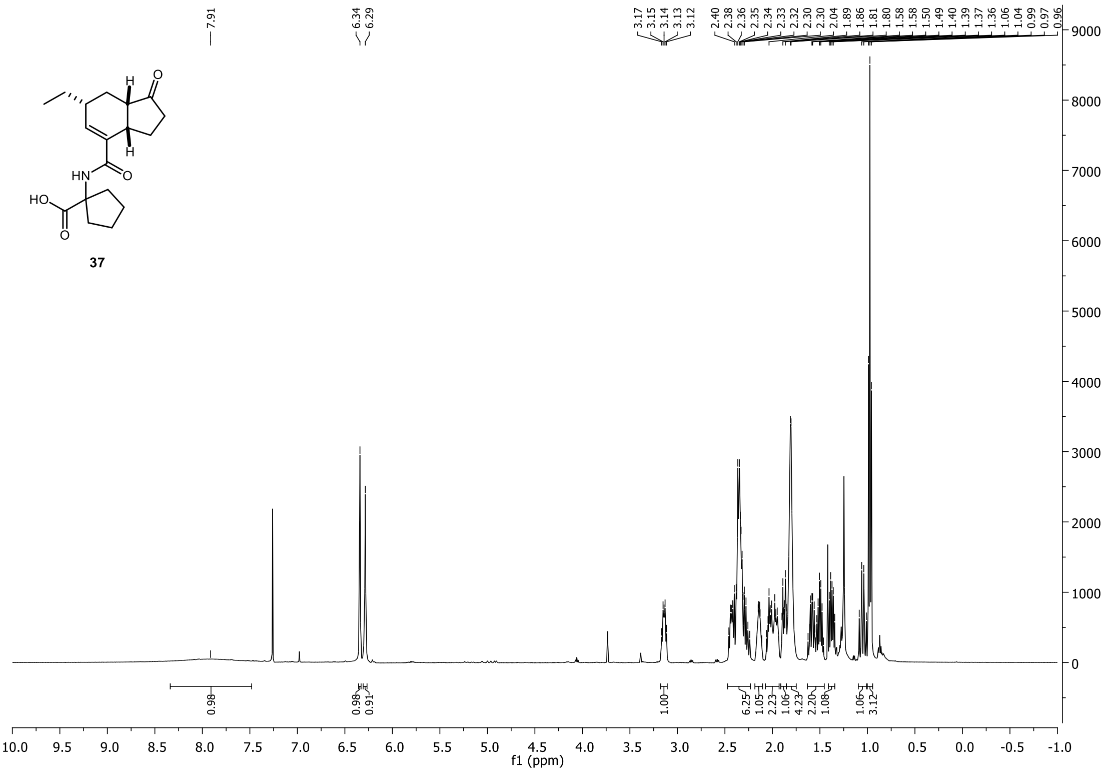

Supplementary Figure 151: ^{13}C NMR 37.

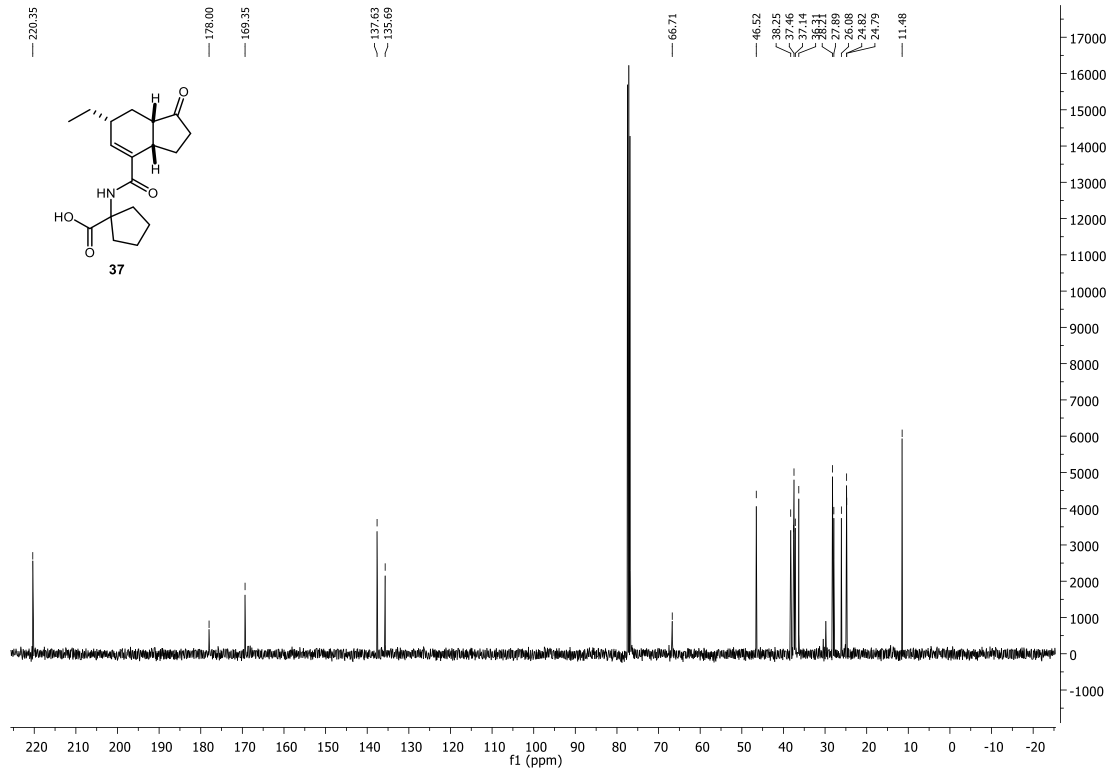

Supplementary Figure 152: ^{1}H NMR S56.

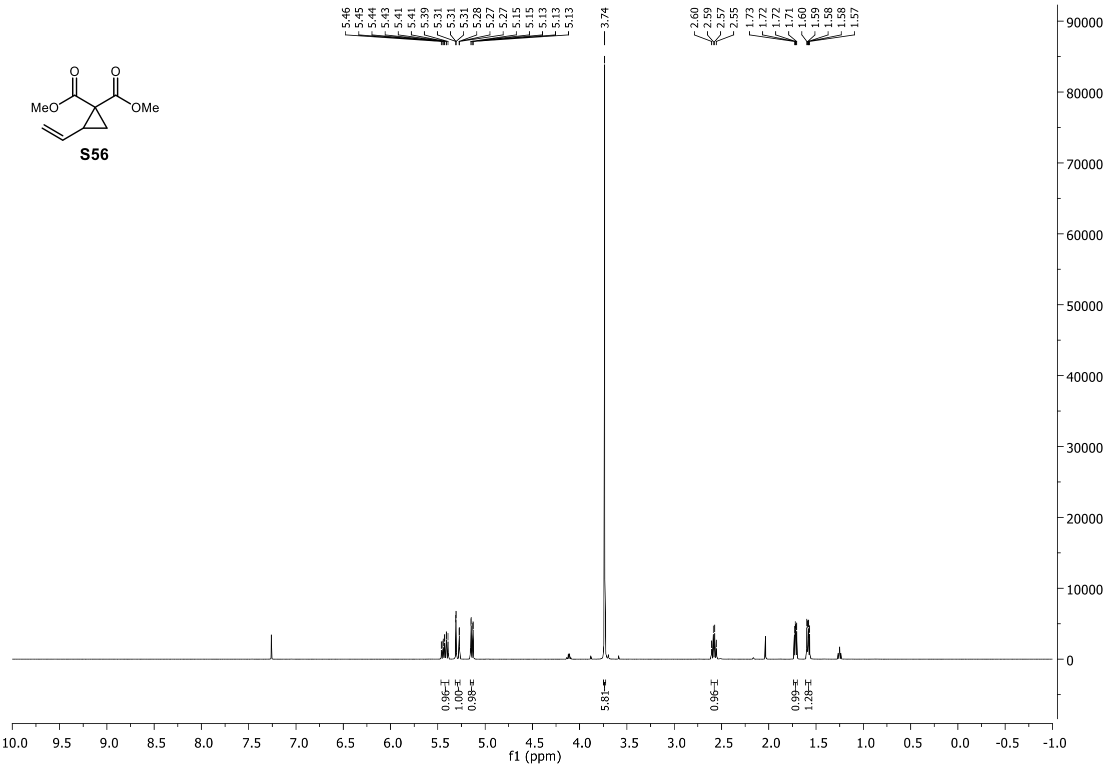

Supplementary Figure 153: ^{13}C NMR S56.

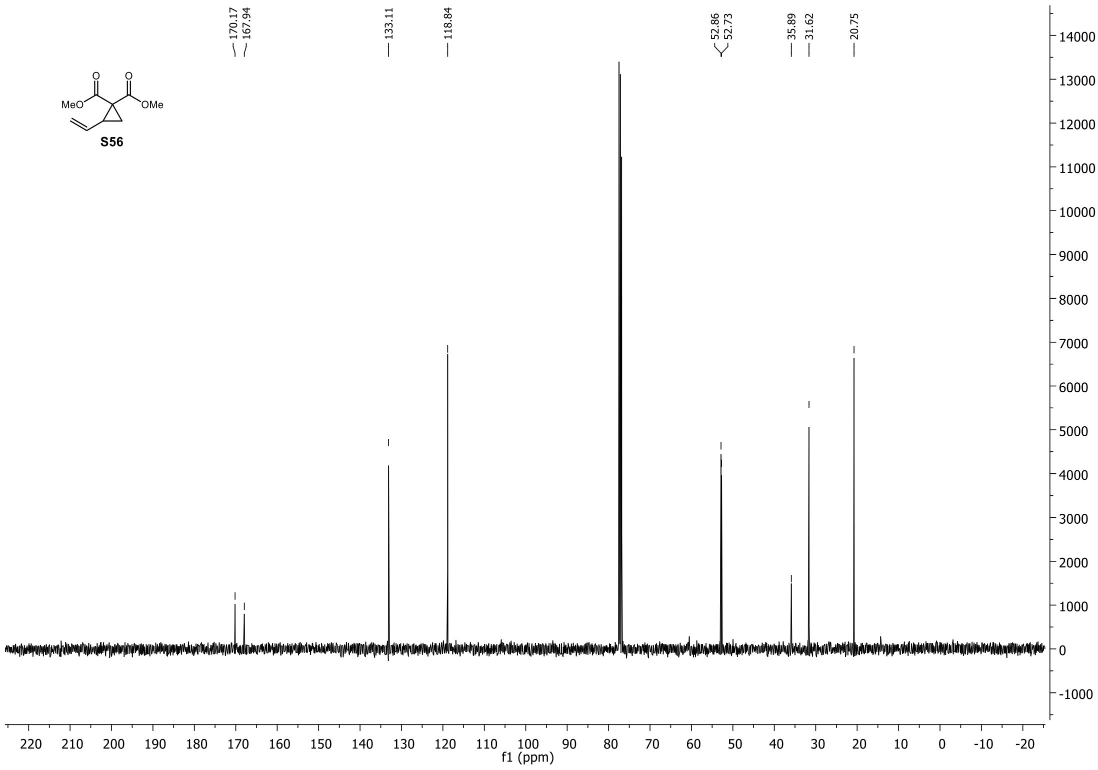

Supplementary Figure 154: ^{1}H NMR S57.

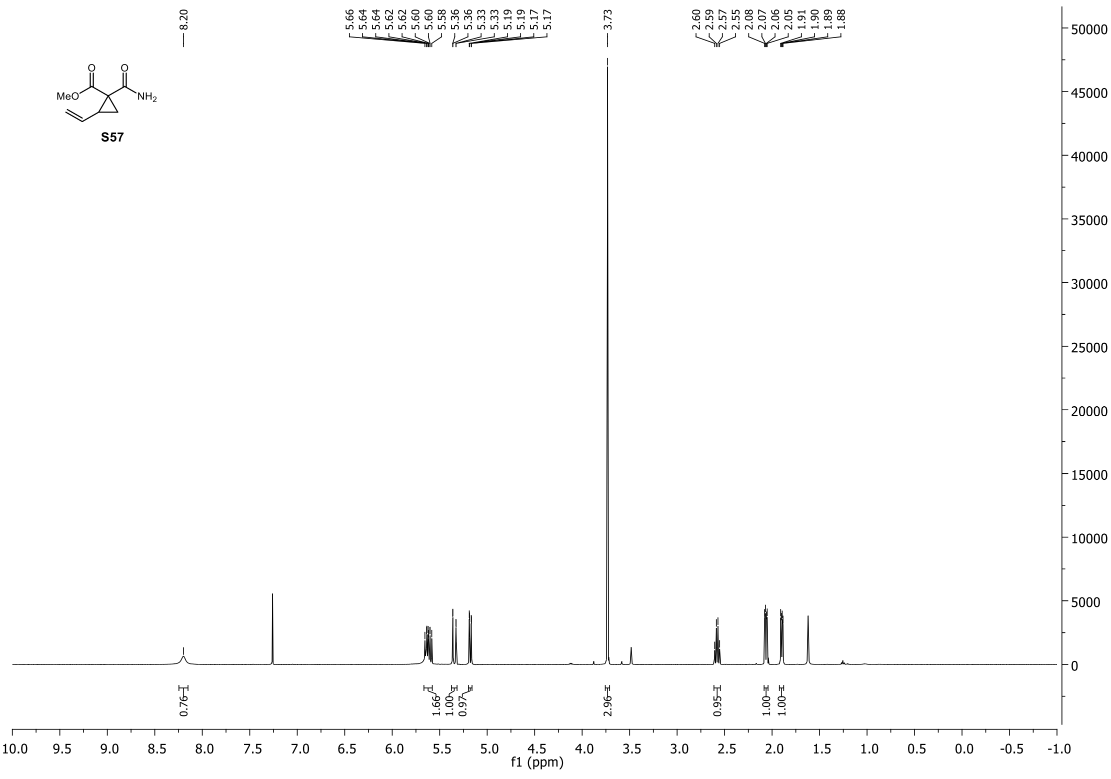

Supplementary Figure 155: ^{13}C NMR S57.

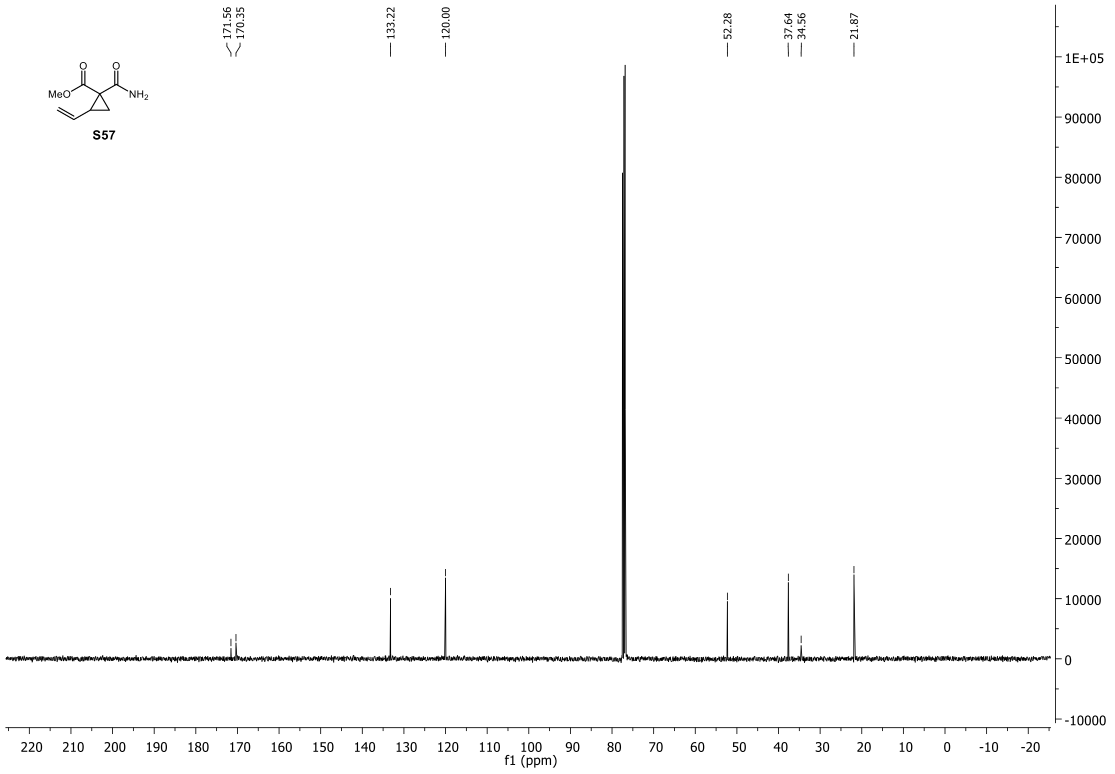

Supplementary Figure 156: ^{1}H NMR S58.

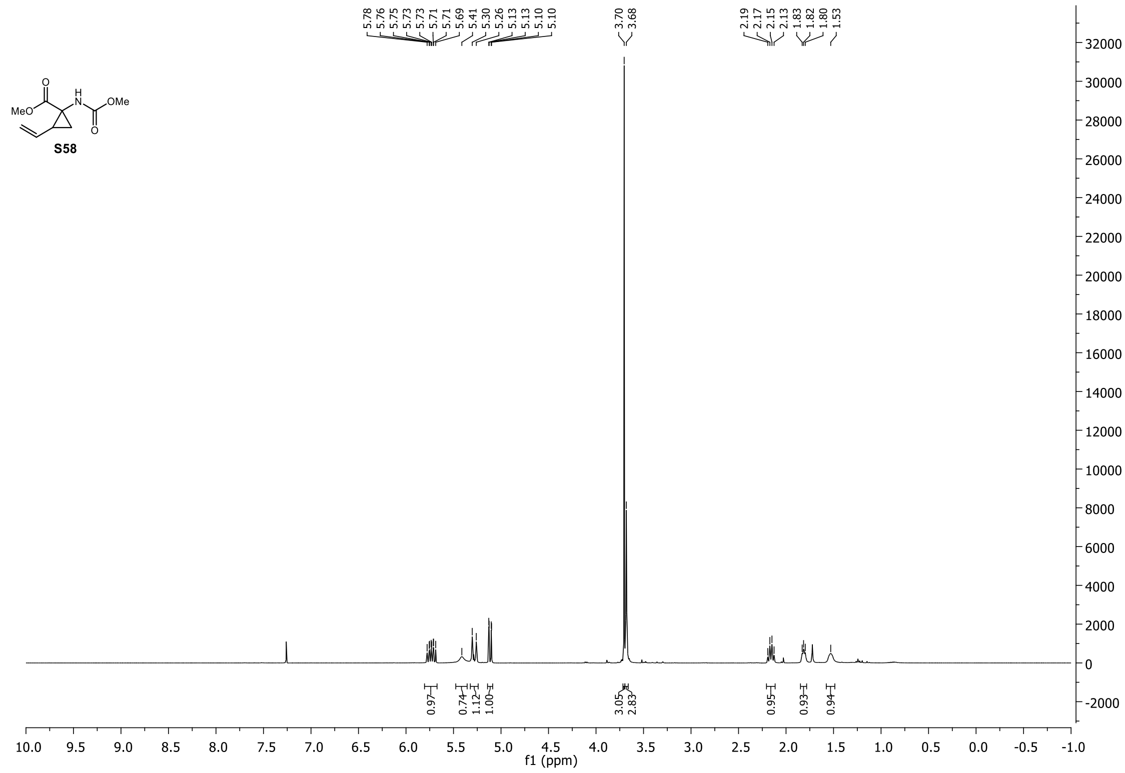

Supplementary Figure 157: ^{13}C NMR S58.

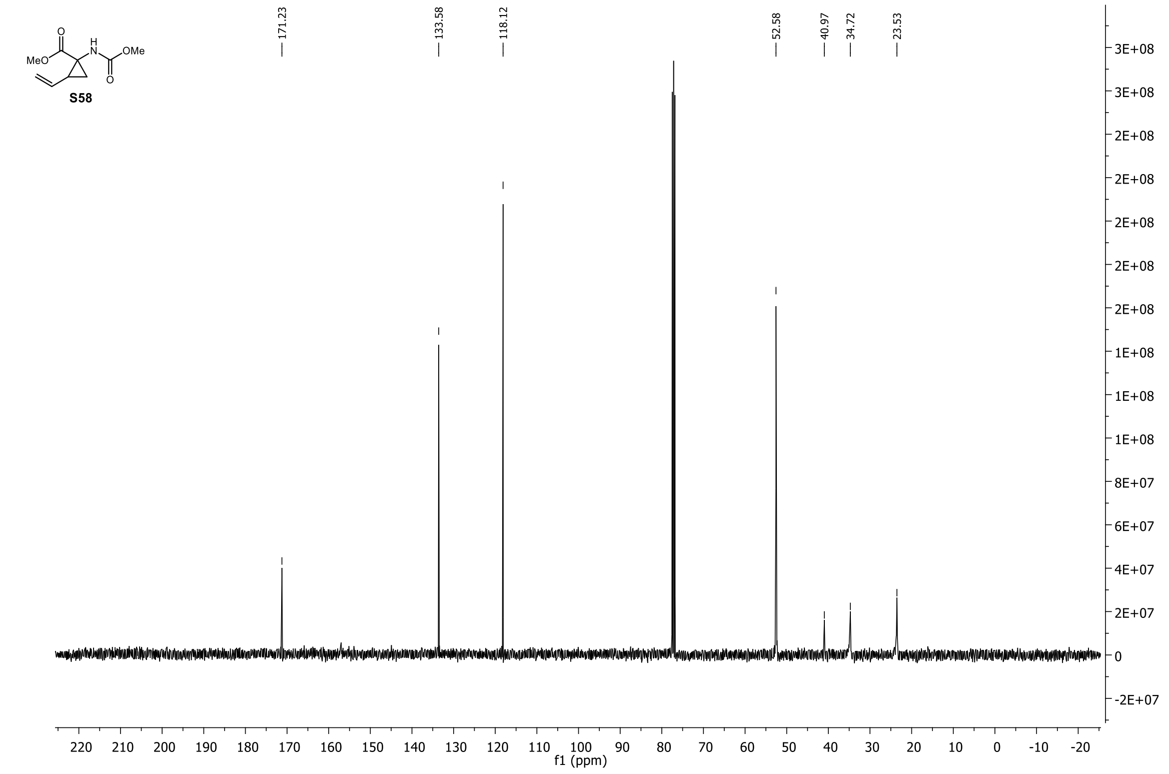

Supplementary Figure 158: ^{1}H NMR S60.

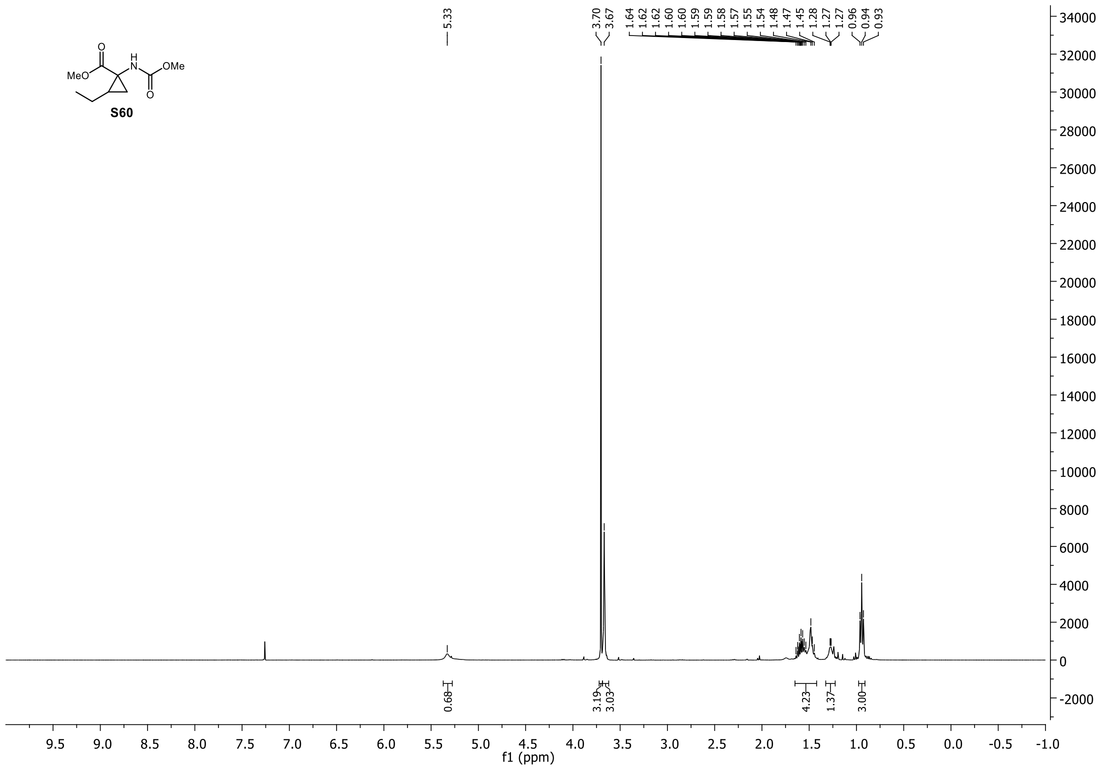

Supplementary Figure 159: ^{13}C NMR S60.

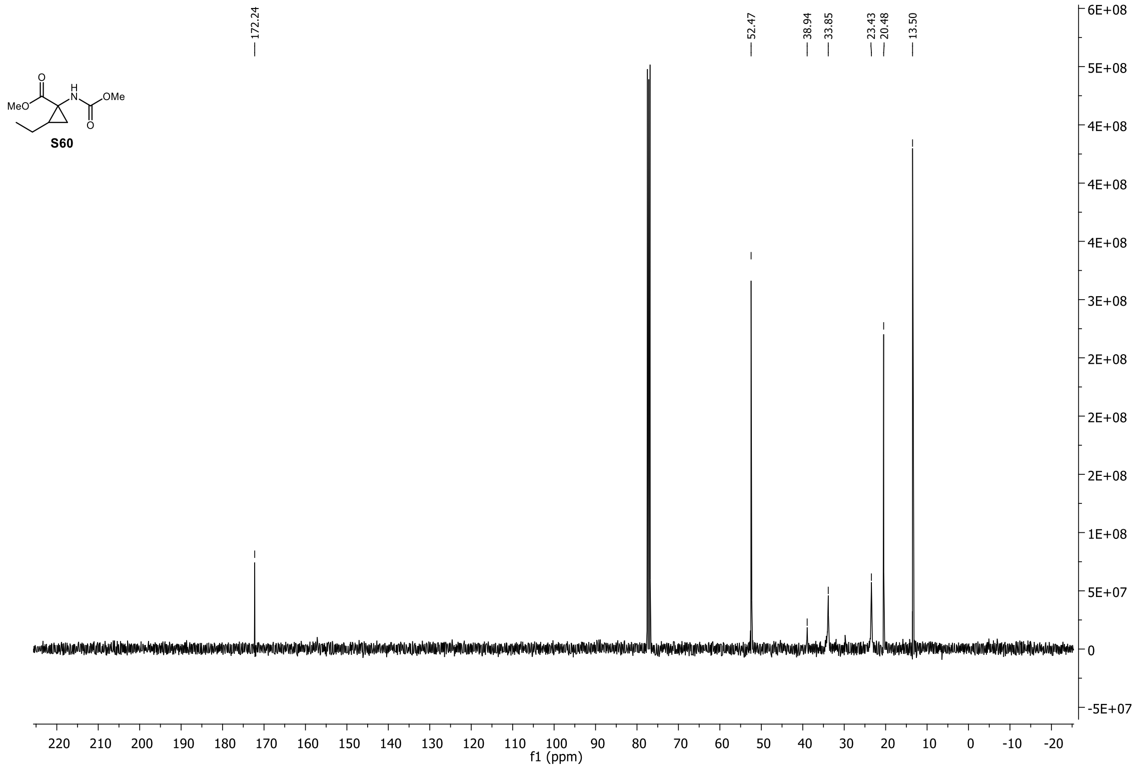

Supplementary Figure 160: ^{1}H NMR S61.

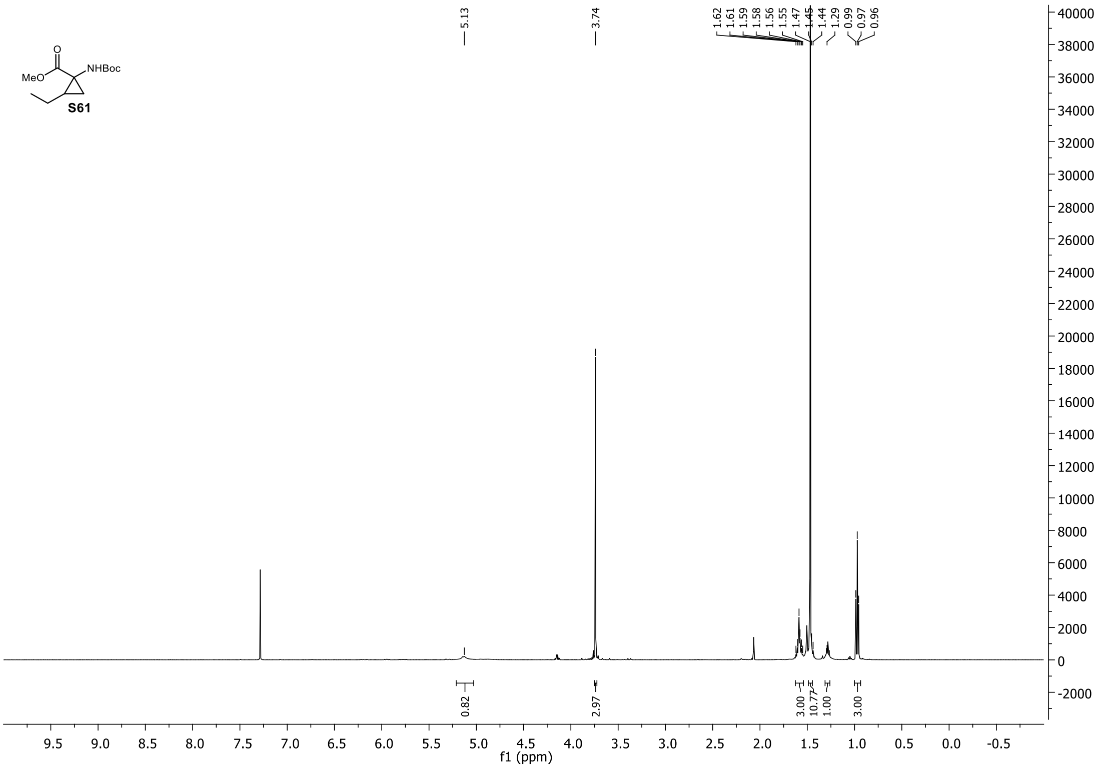

Supplementary Figure 161: ^{13}C NMR S61.

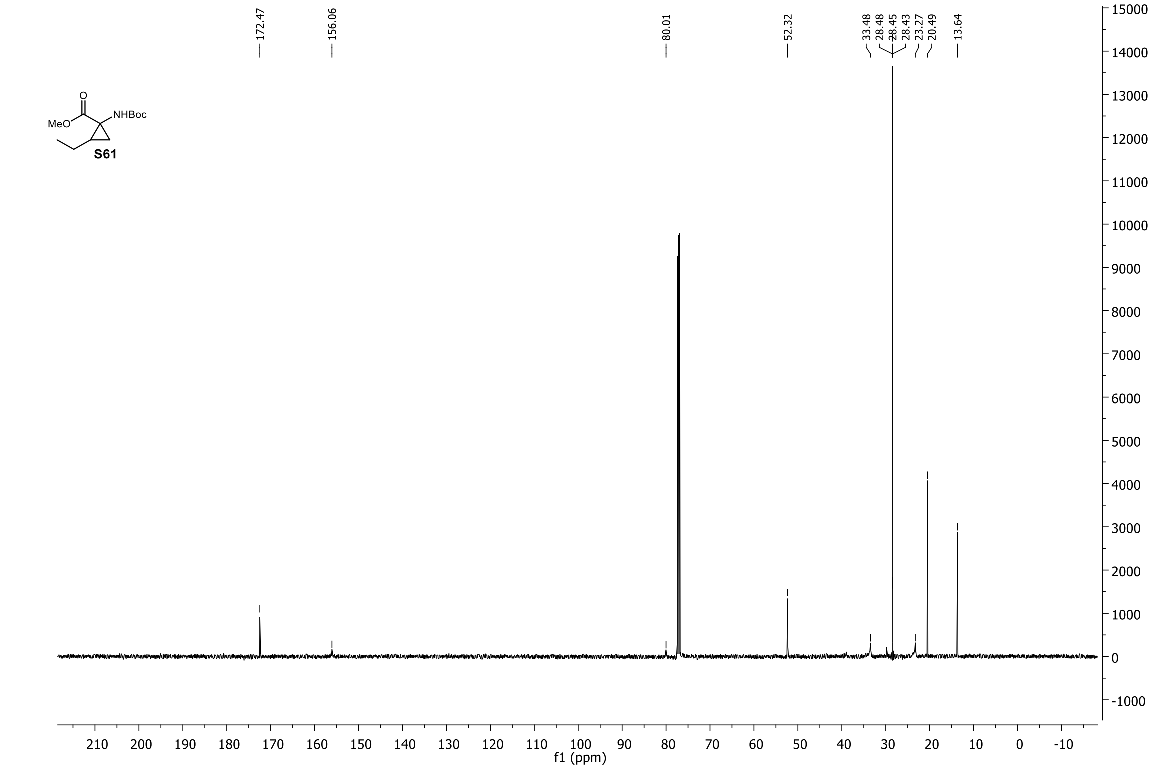

Supplementary Figure 162: ^{1}H NMR S62.

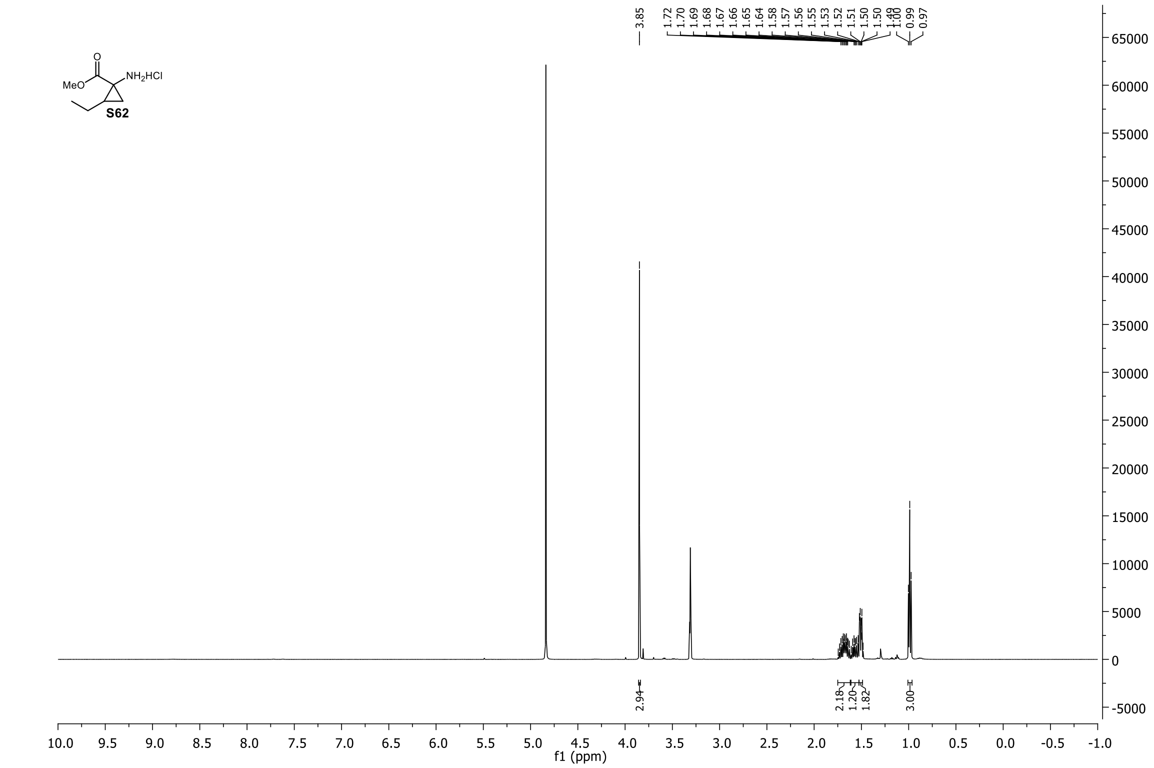

Supplementary Figure 163: ^{13}C NMR S62.

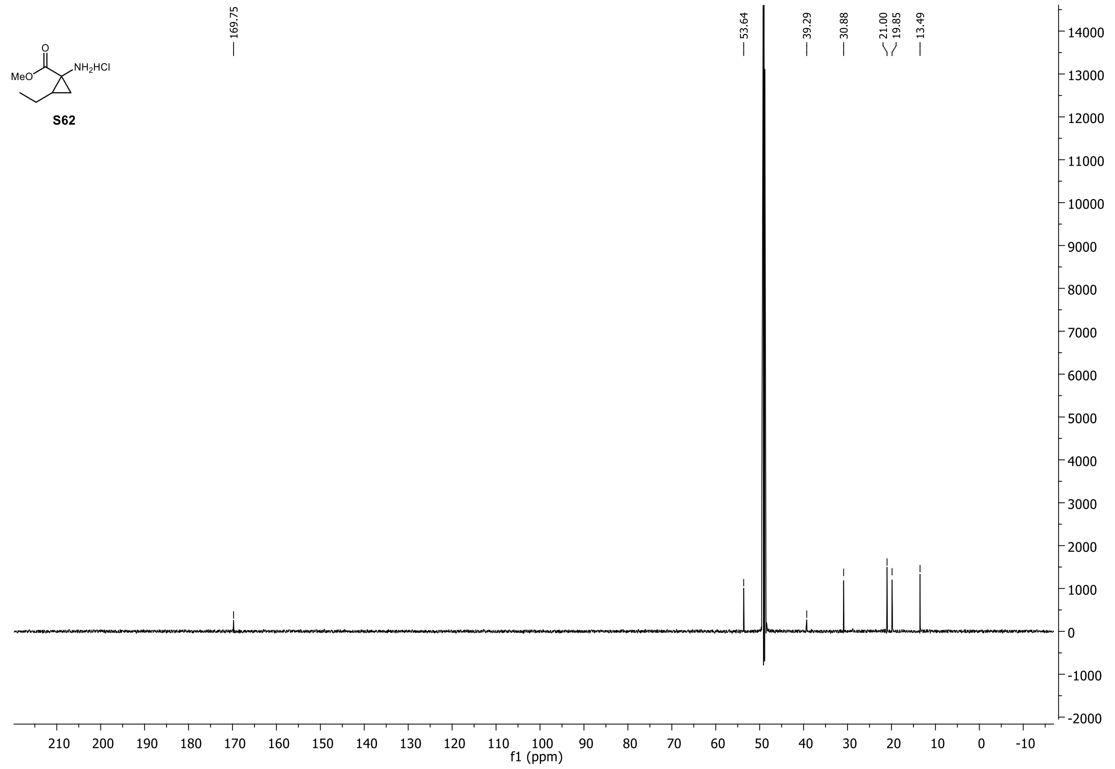

Supplementary Figure 164: ^{1}H NMR ((±)-CMA) (3).

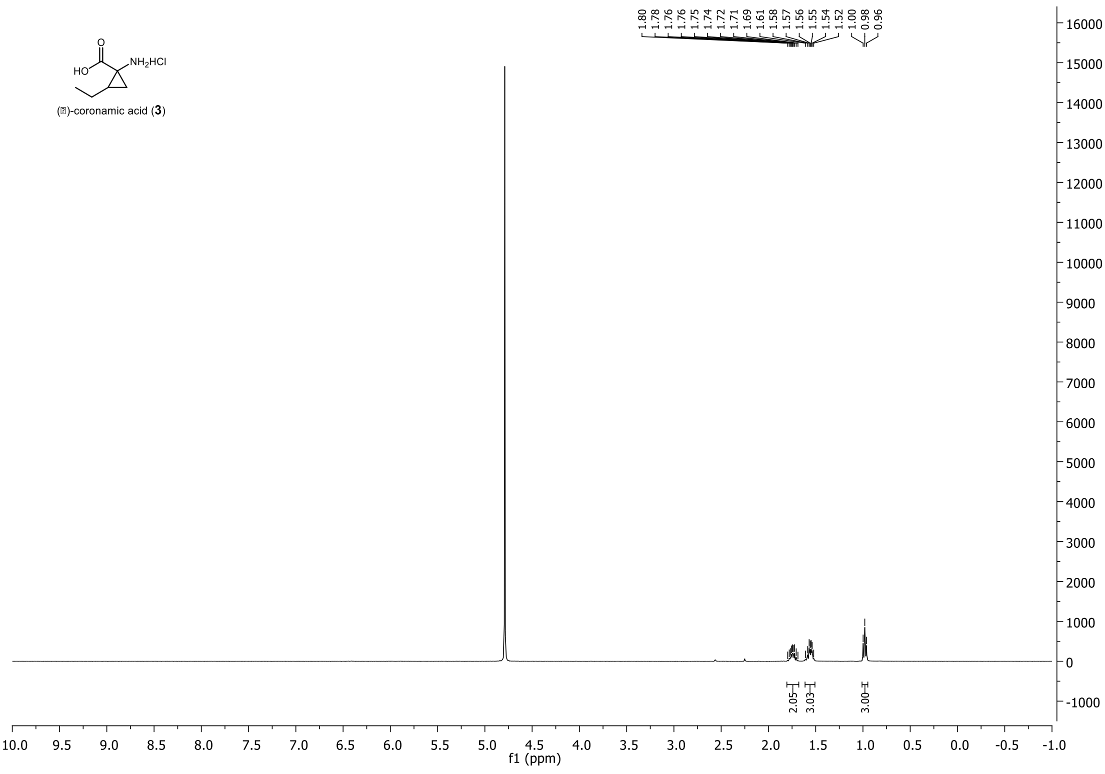

Supplementary Figure 165: ^{13}C NMR ((±)-CMA) (3).

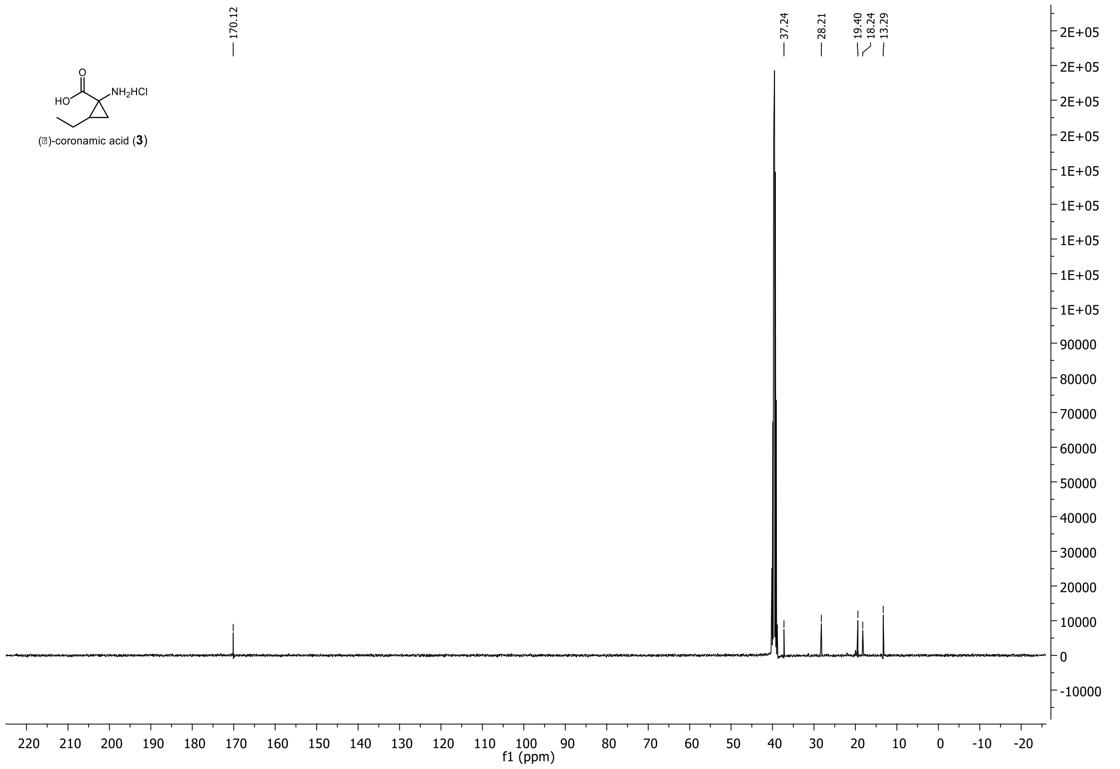

Supplementary Figure 166: ^{1}H NMR S64.

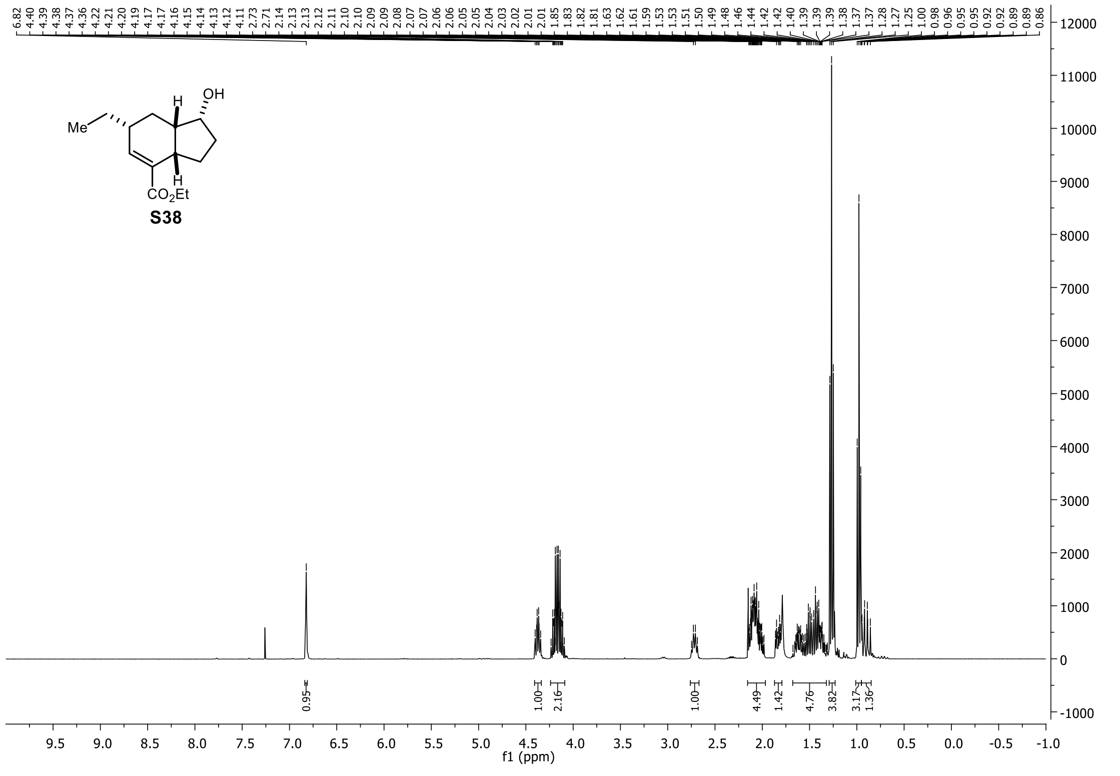

Supplementary Figure 167: ^{13}C NMR S38.

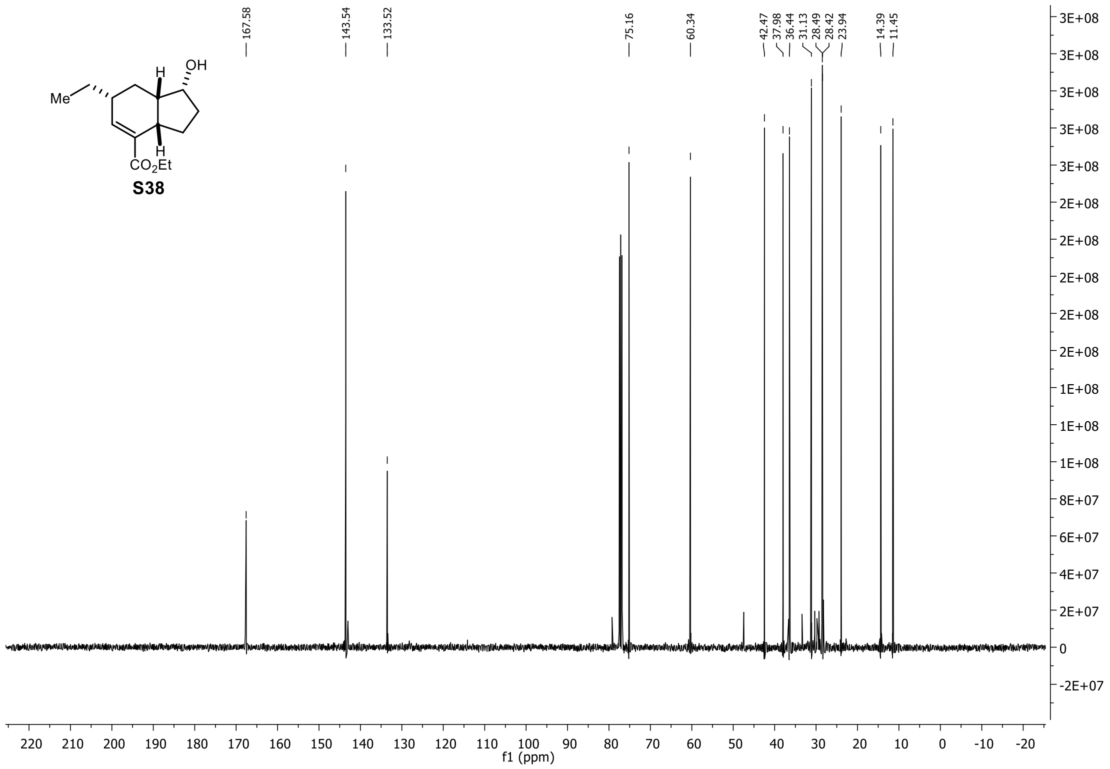

Supplementary Figure 168: ^{1}H NMR S66.

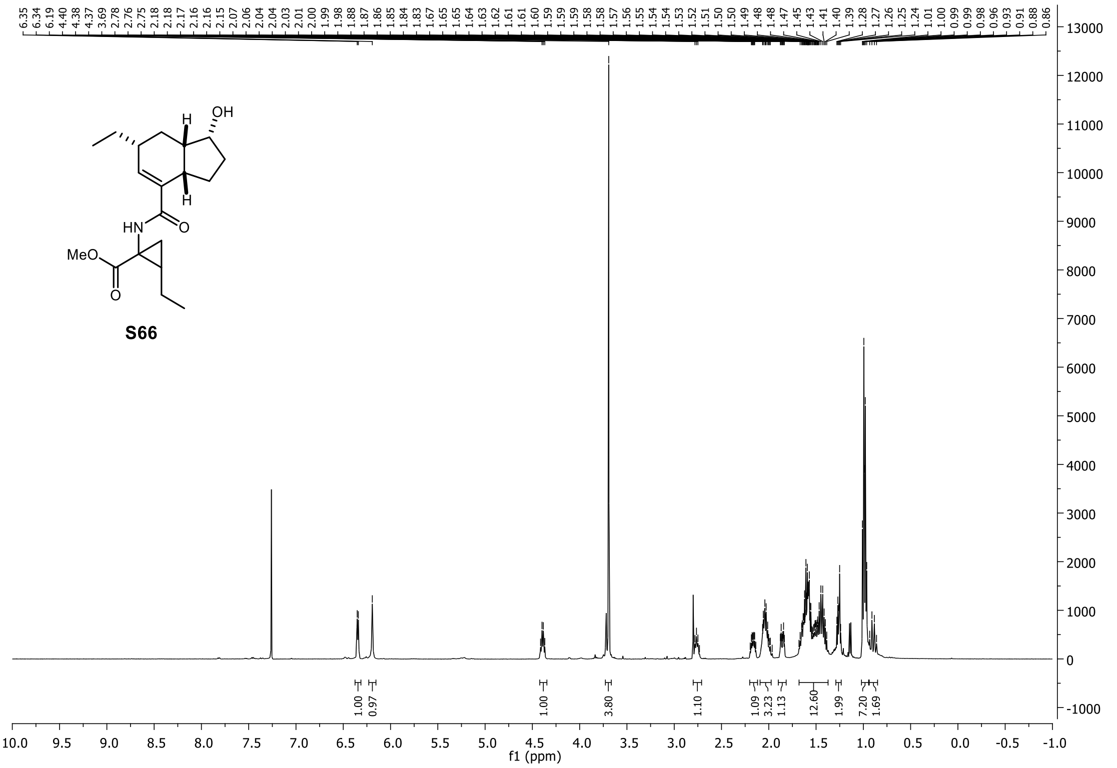

Supplementary Figure 169: ^{13}C NMR S66.

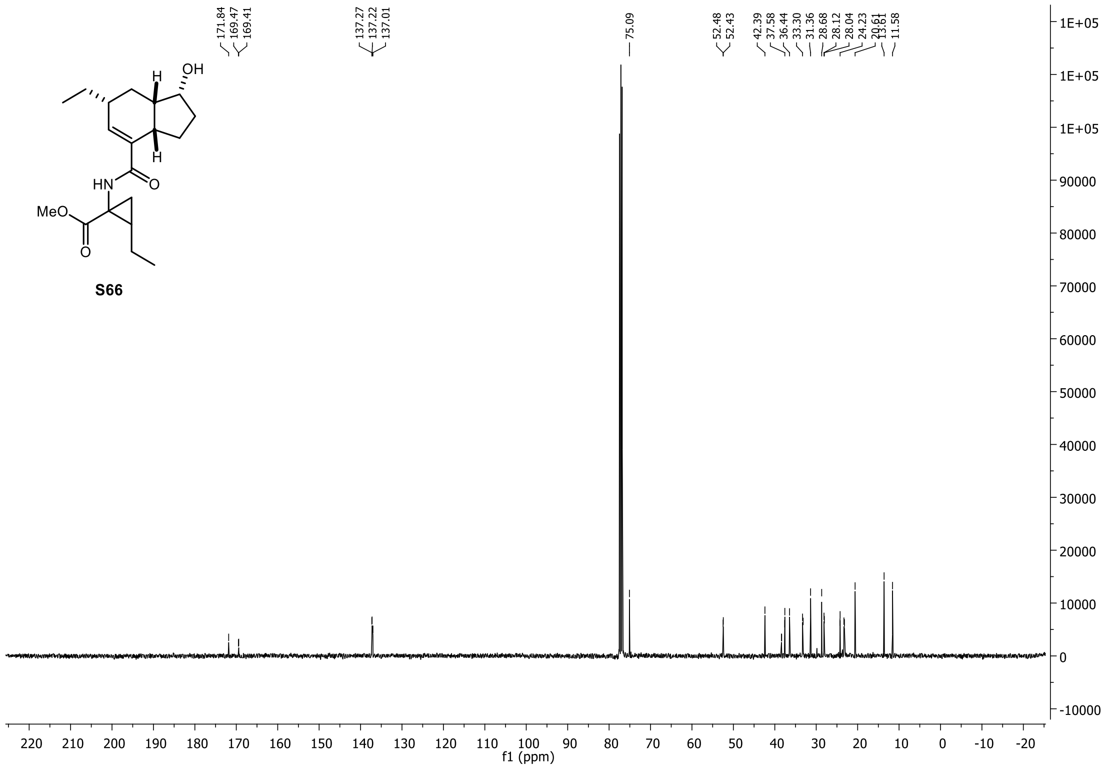

Supplementary Figure 170: ^{1}H NMR 38a.

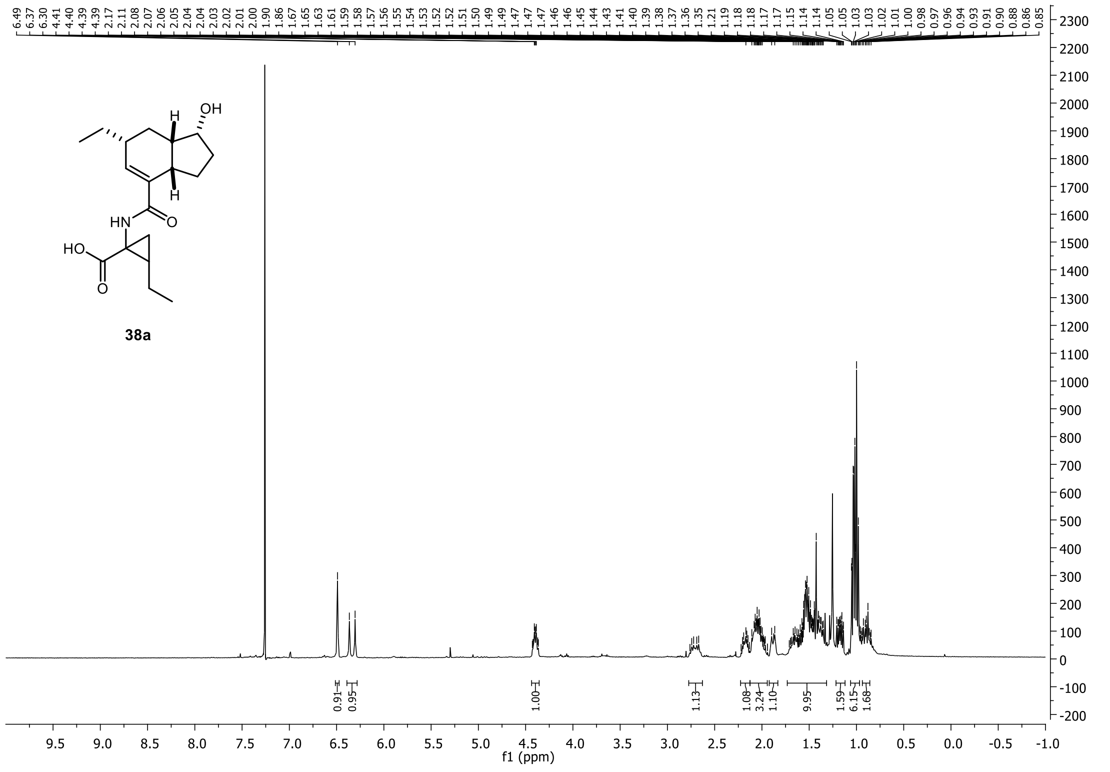

Supplementary Figure 171: ^{13}C NMR 38a.

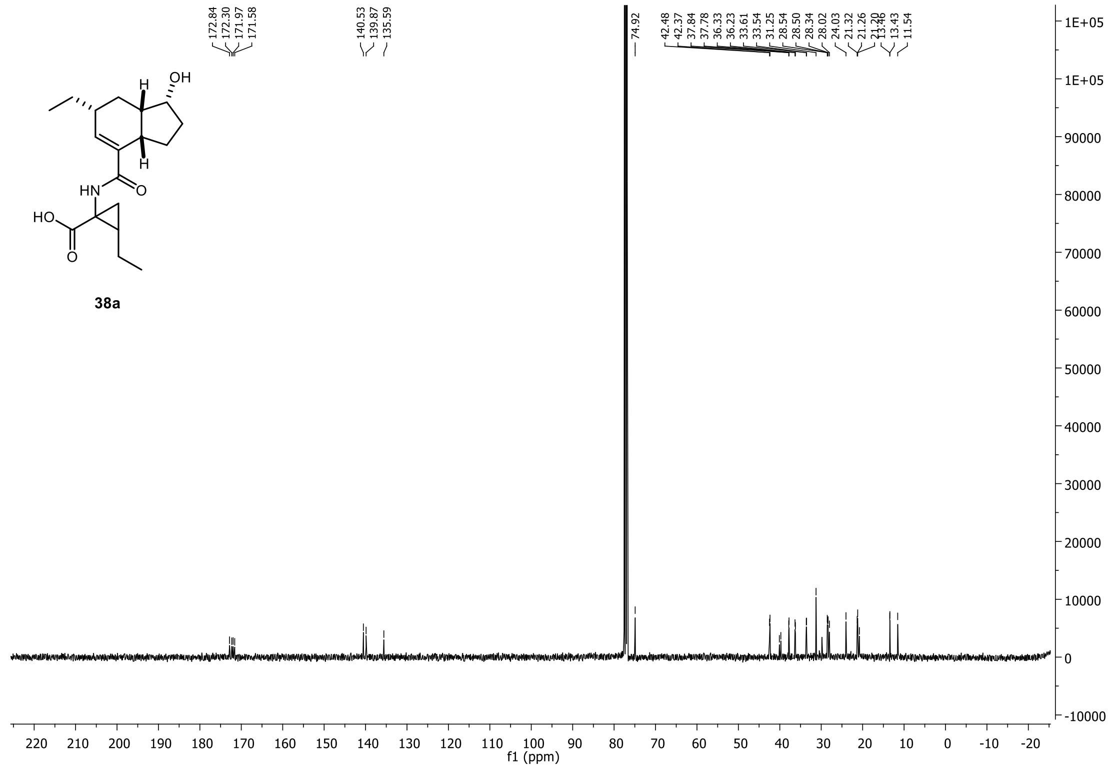

Supplementary Figure 172: ^{1}H NMR 38b.

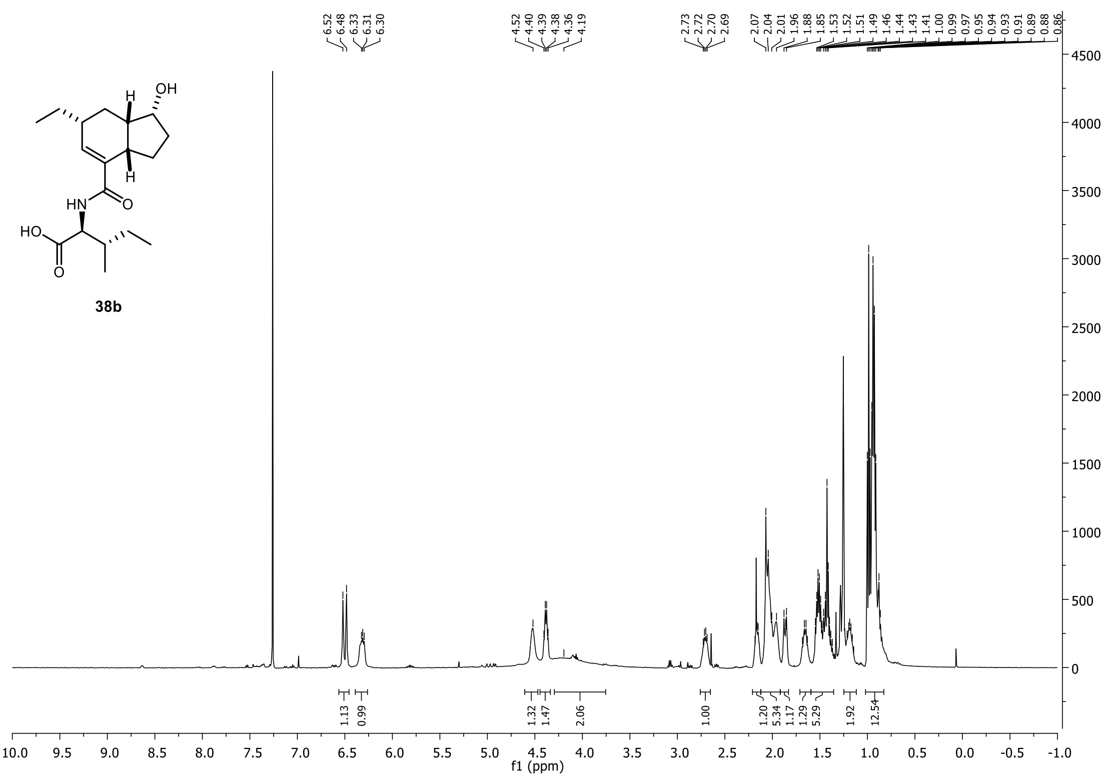

Supplementary Figure 173: ^{13}C NMR 38b.

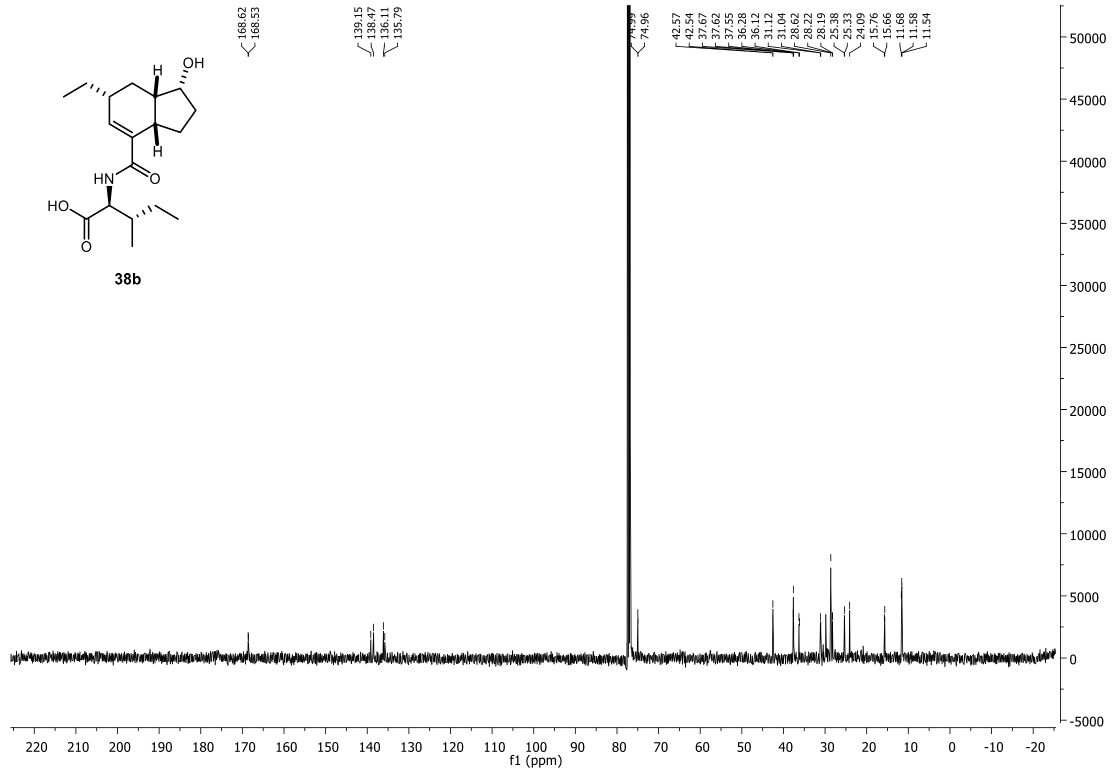

Supplementary Figure 174: ^{1}H NMR S67.

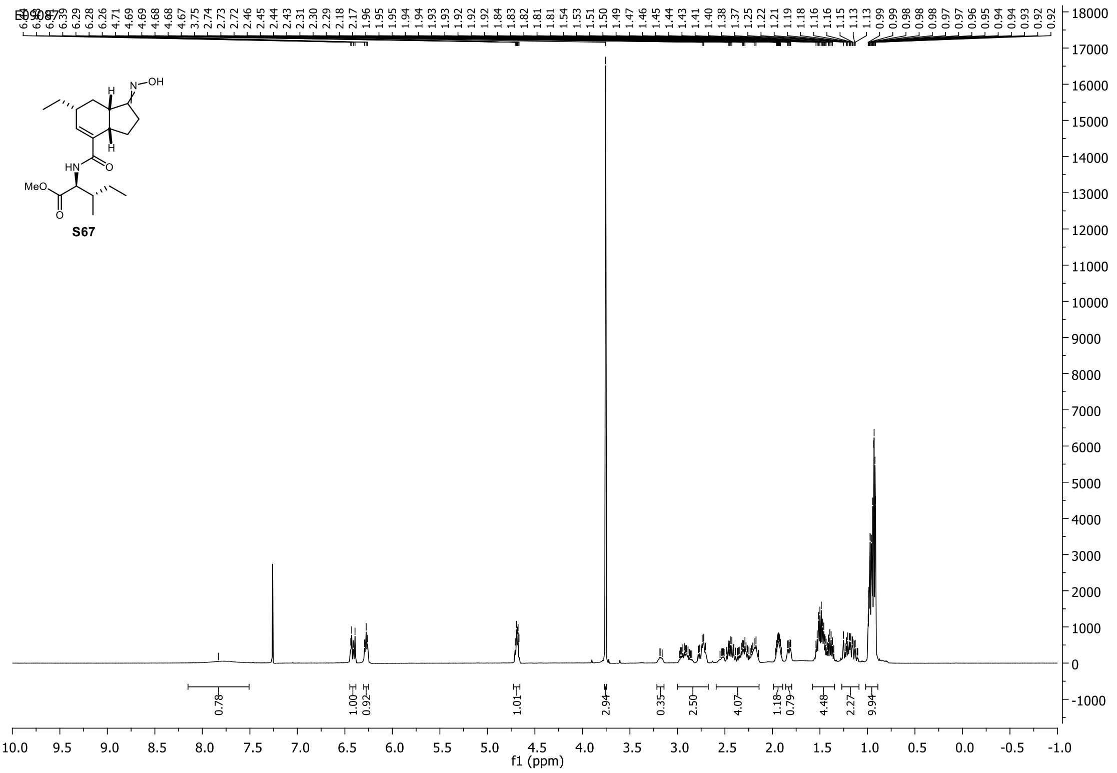

Supplementary Figure 175: ^{13}C NMR S67.

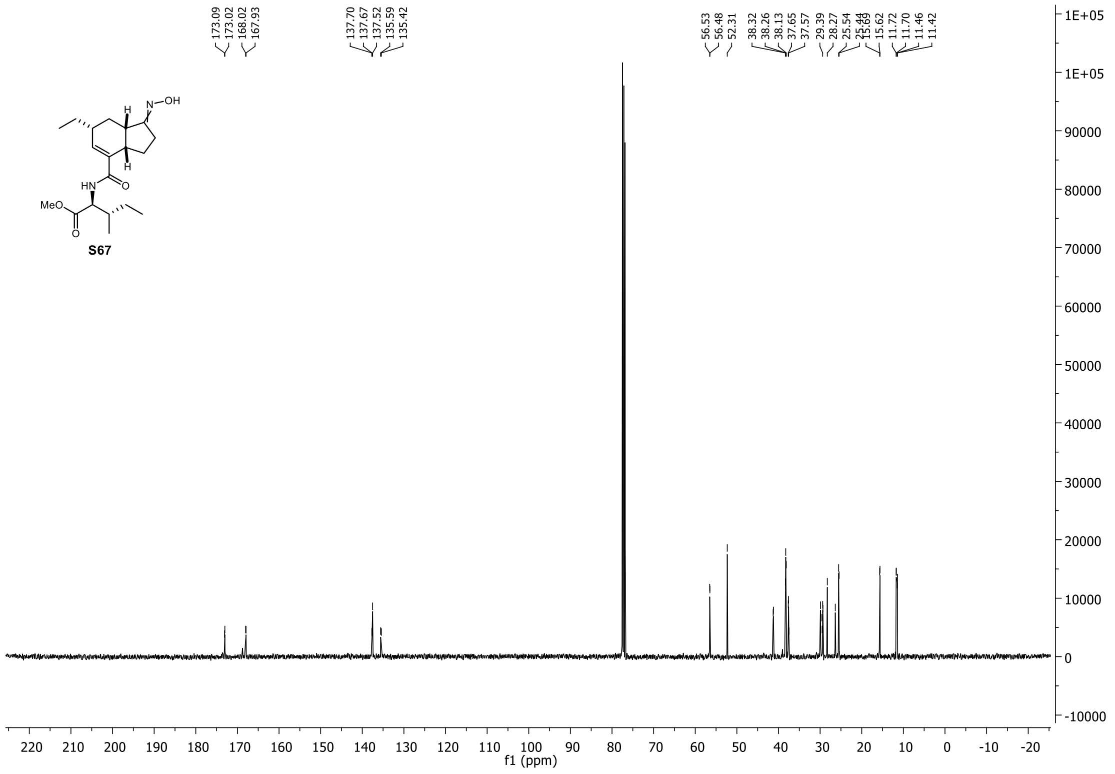

Supplementary Figure 176: ^{1}H NMR 39b.

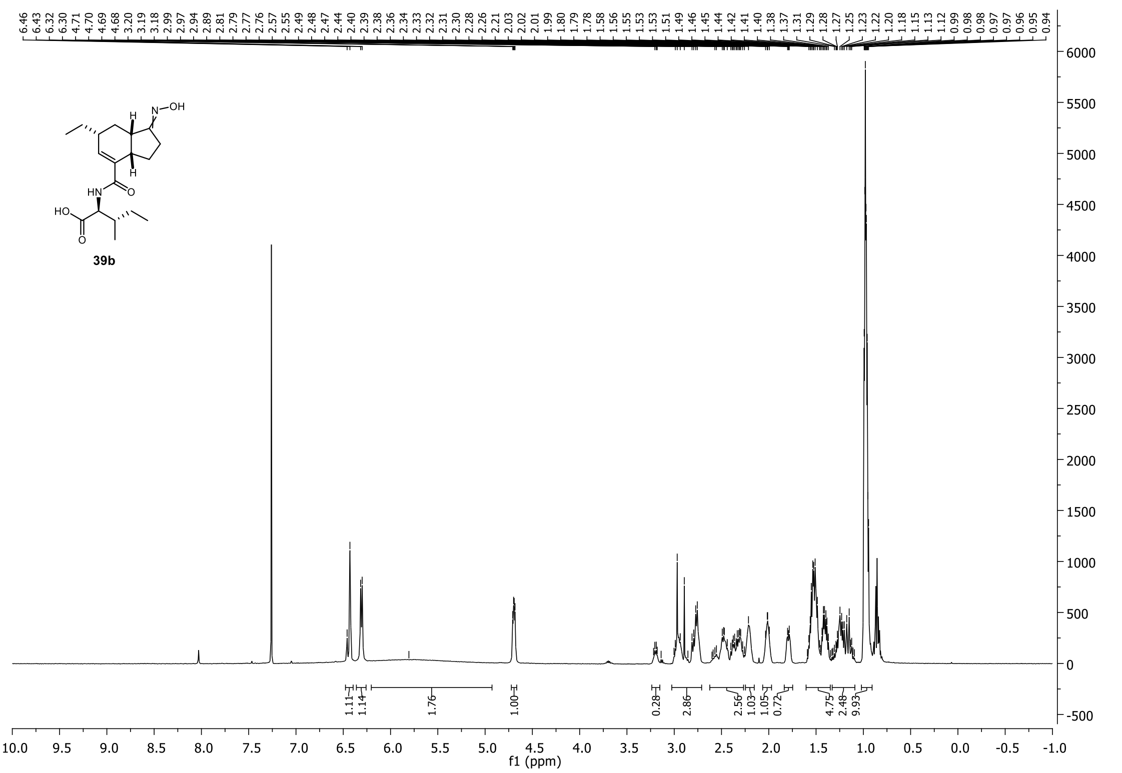

Supplementary Figure 177: ^{13}C NMR 39b.

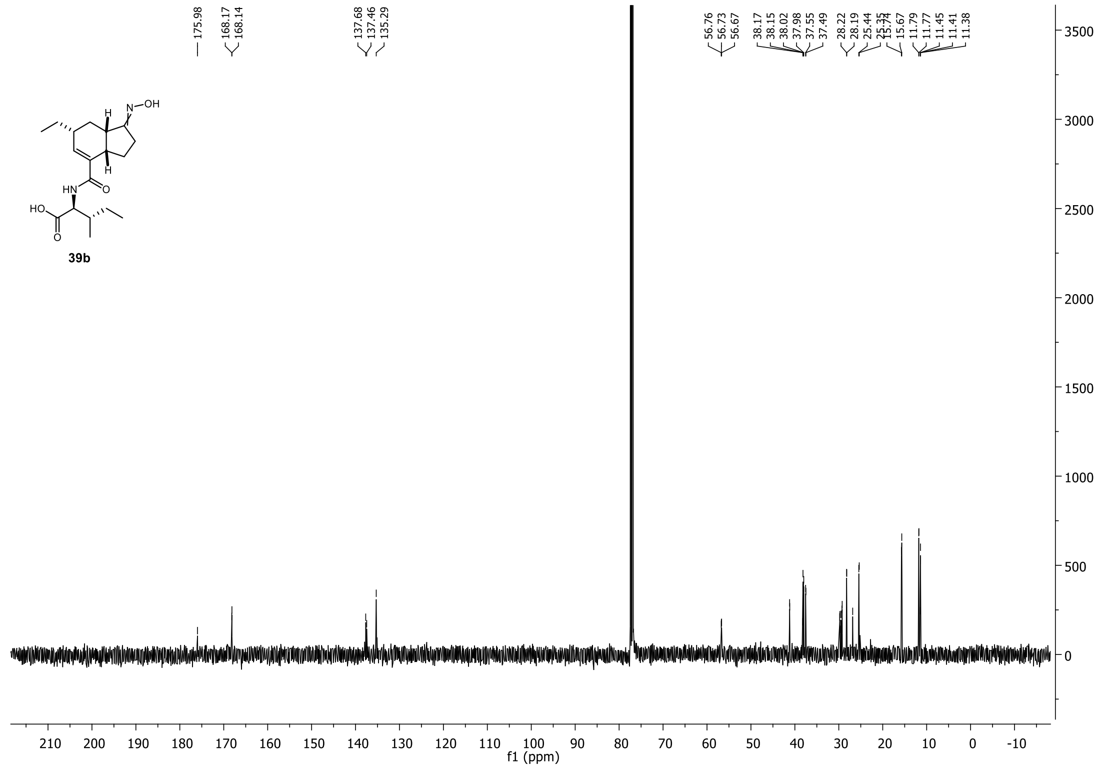

Supplementary Figure 178: ^{1}H NMR S68.

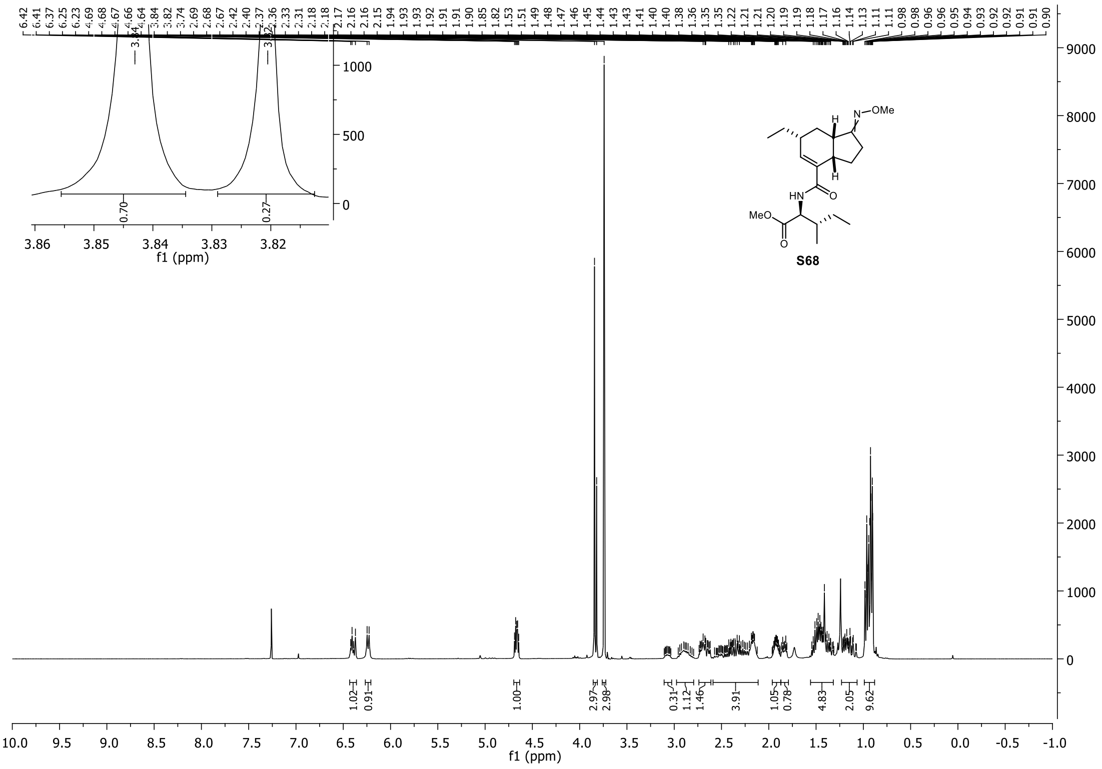

Supplementary Figure 179: ^{13}C NMR S68.

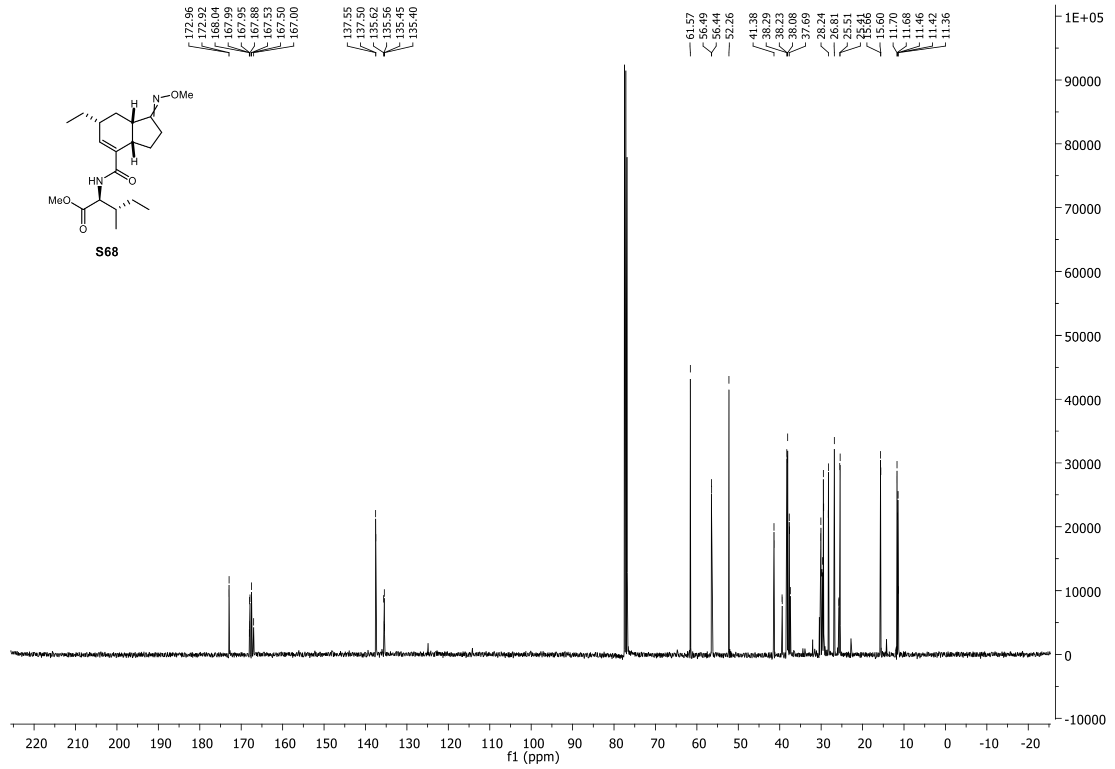

Supplementary Figure 180: ^{1}H NMR 40b.

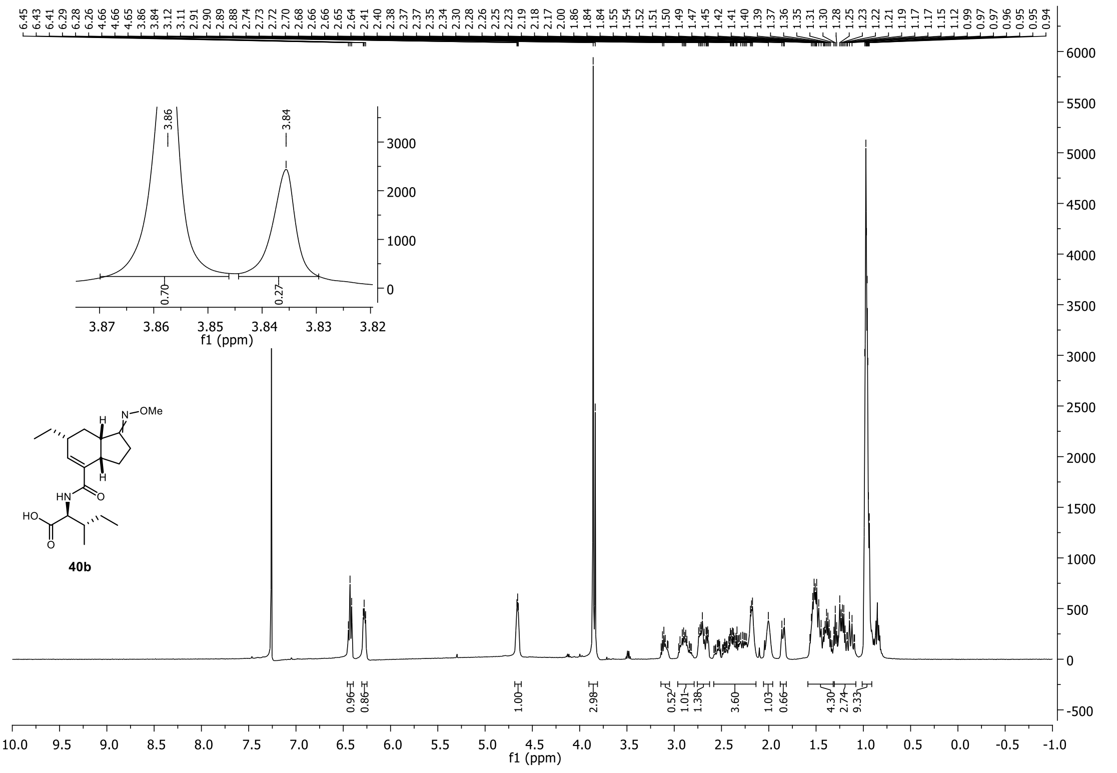

Supplementary Figure 181: ^{13}C NMR 40b.

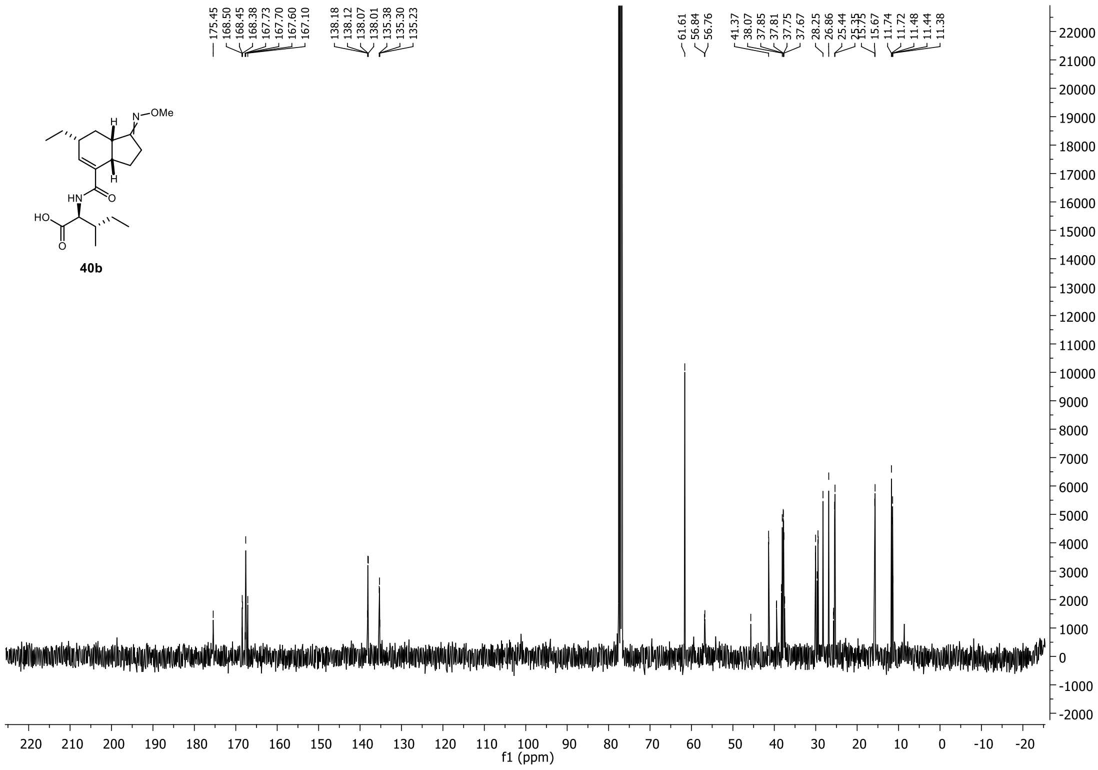

Supplementary Figure 182: ^{1}H NMR S69.

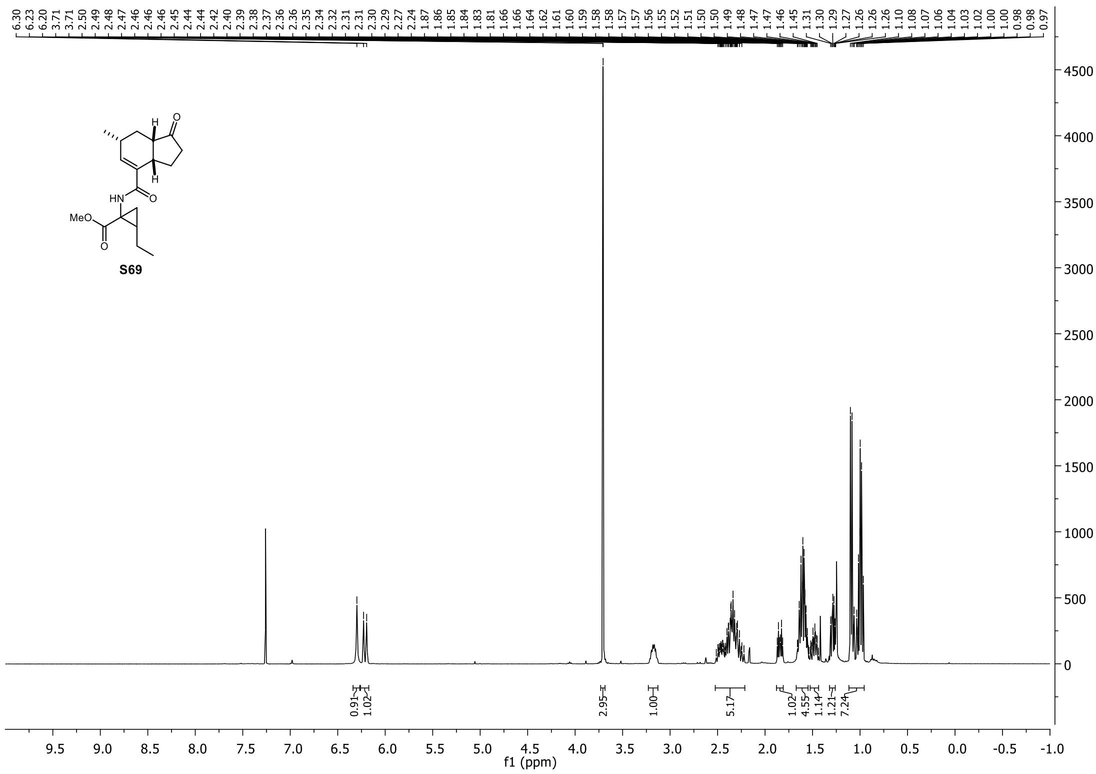

Supplementary Figure 183: ^{13}C NMR S69.

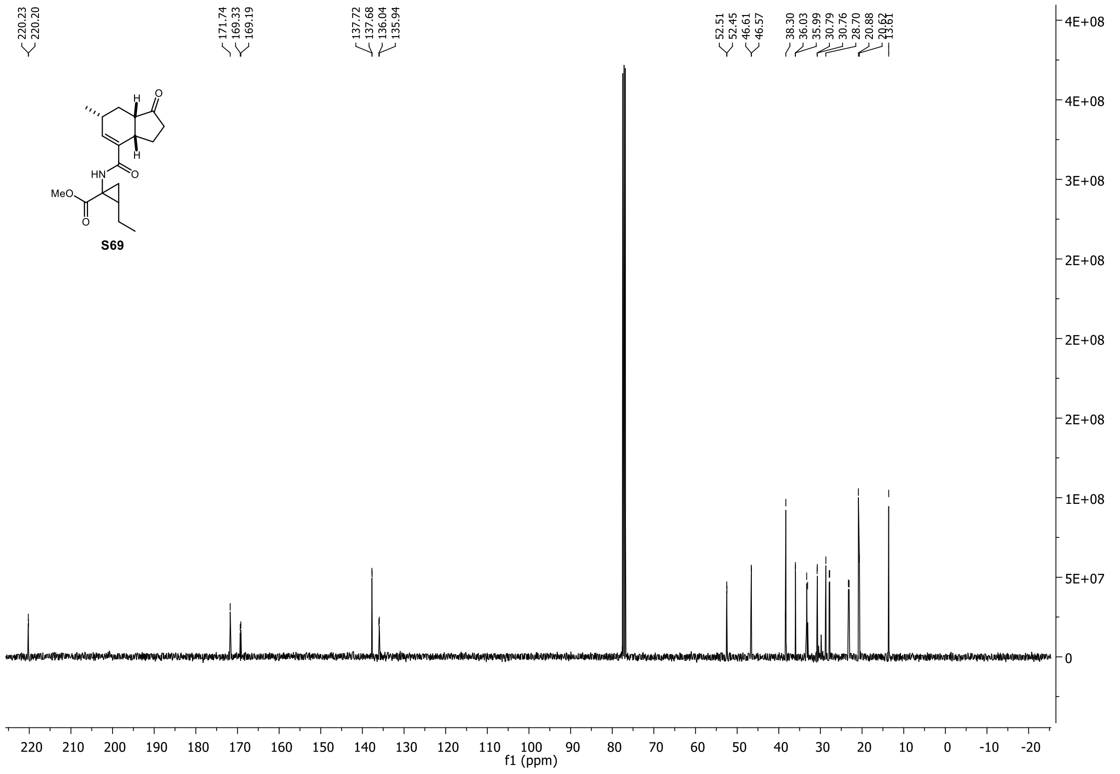

Supplementary Figure 184: ^{1}H NMR 41a.

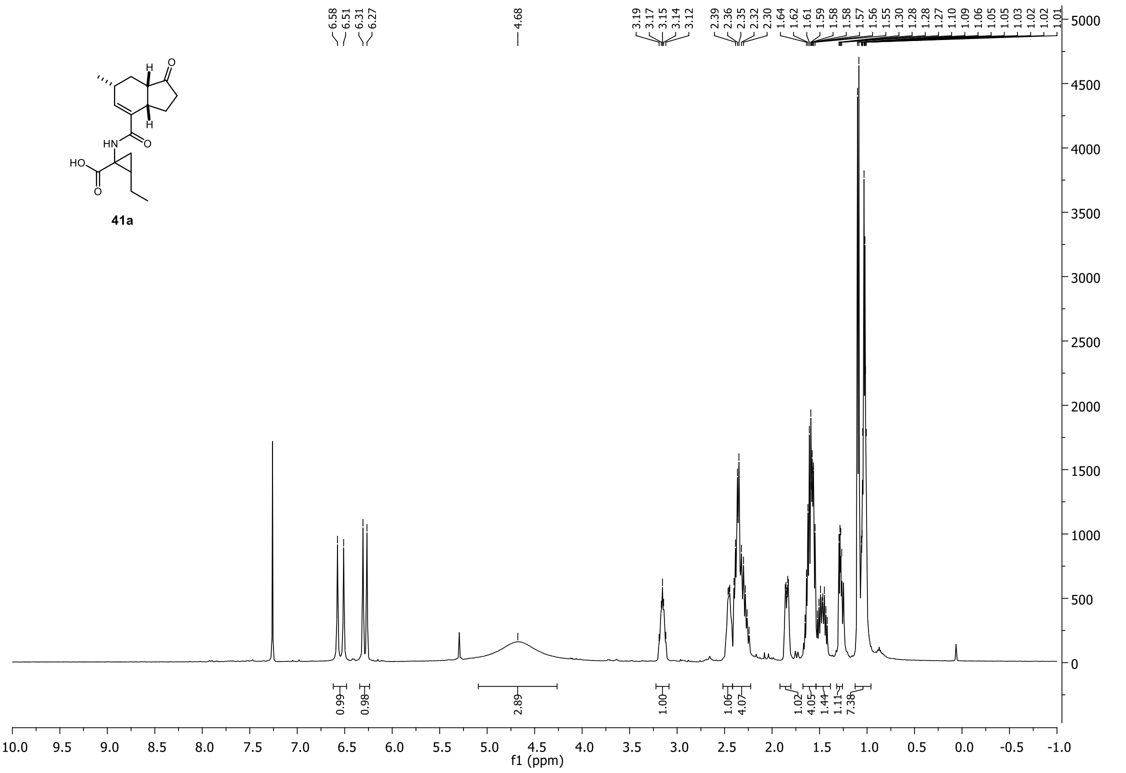

Supplementary Figure 185: ^{13}C NMR 41a.

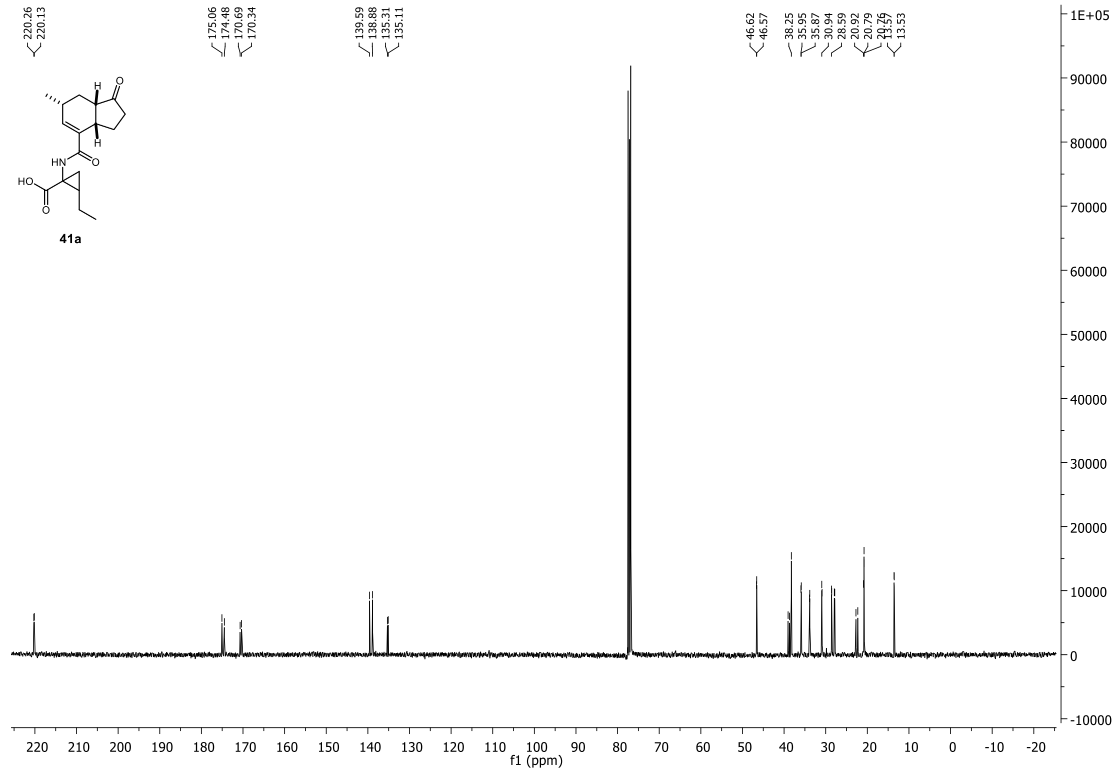

Supplementary Figure 186: ^{1}H NMR S70.

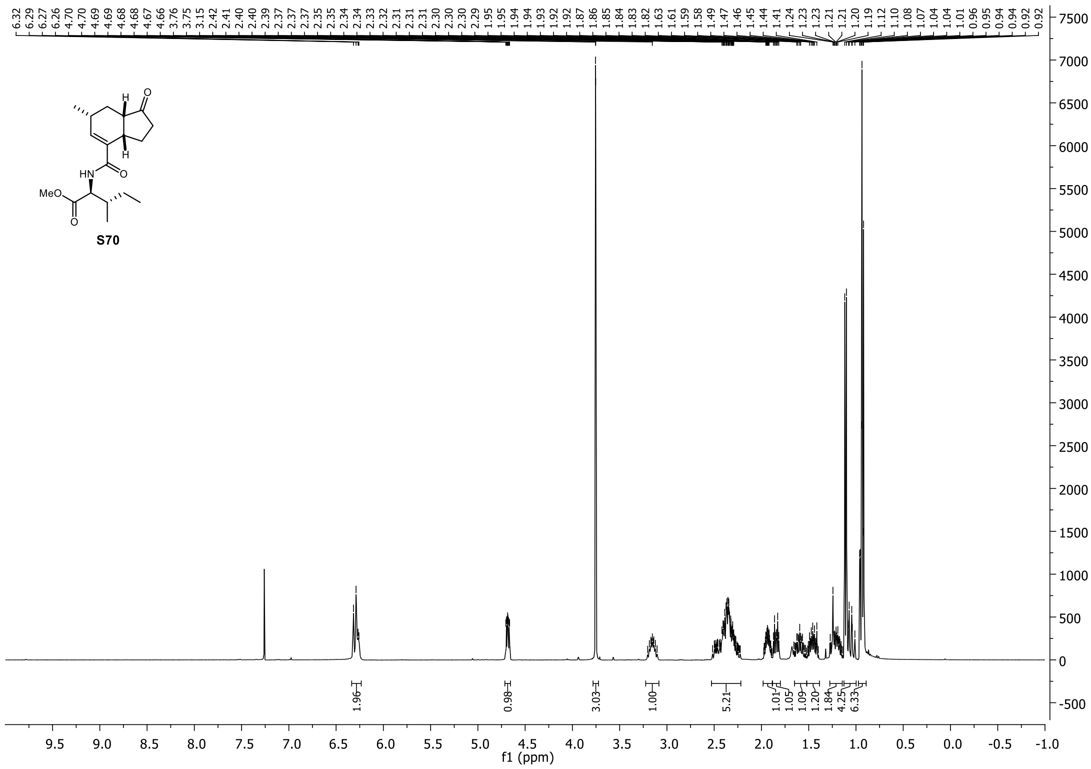

Supplementary Figure 187: ^{13}C NMR S70.

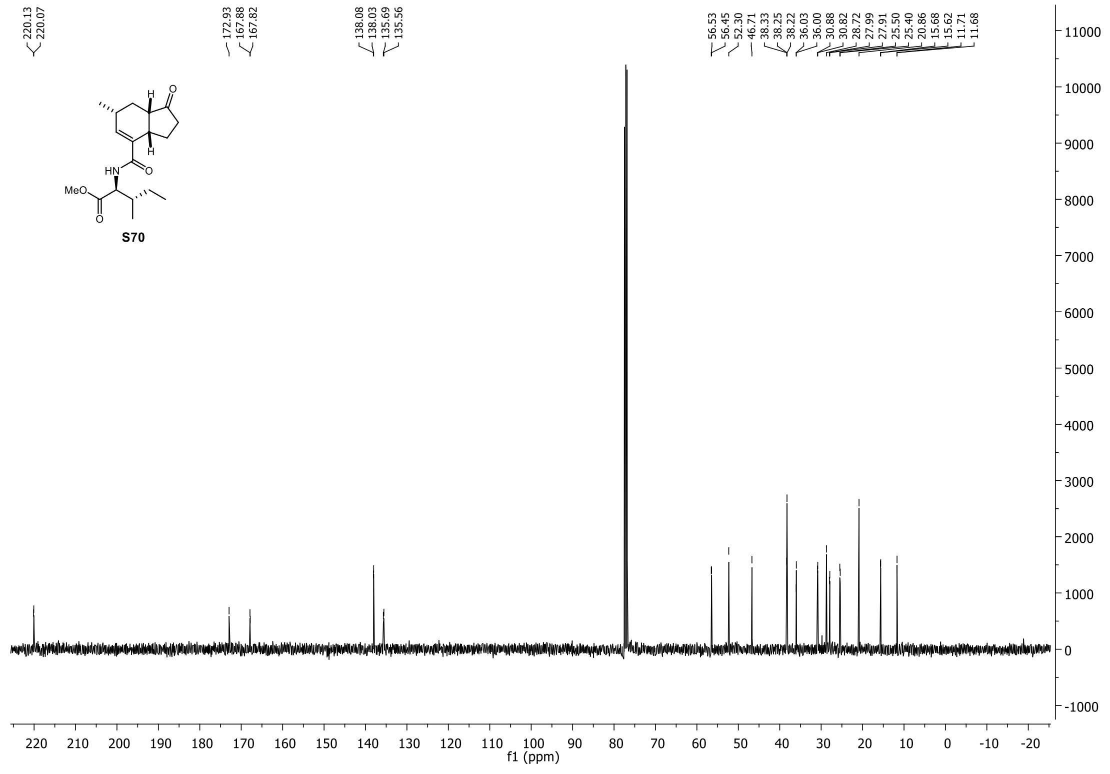

Supplementary Figure 188: ^{1}H NMR 41b.

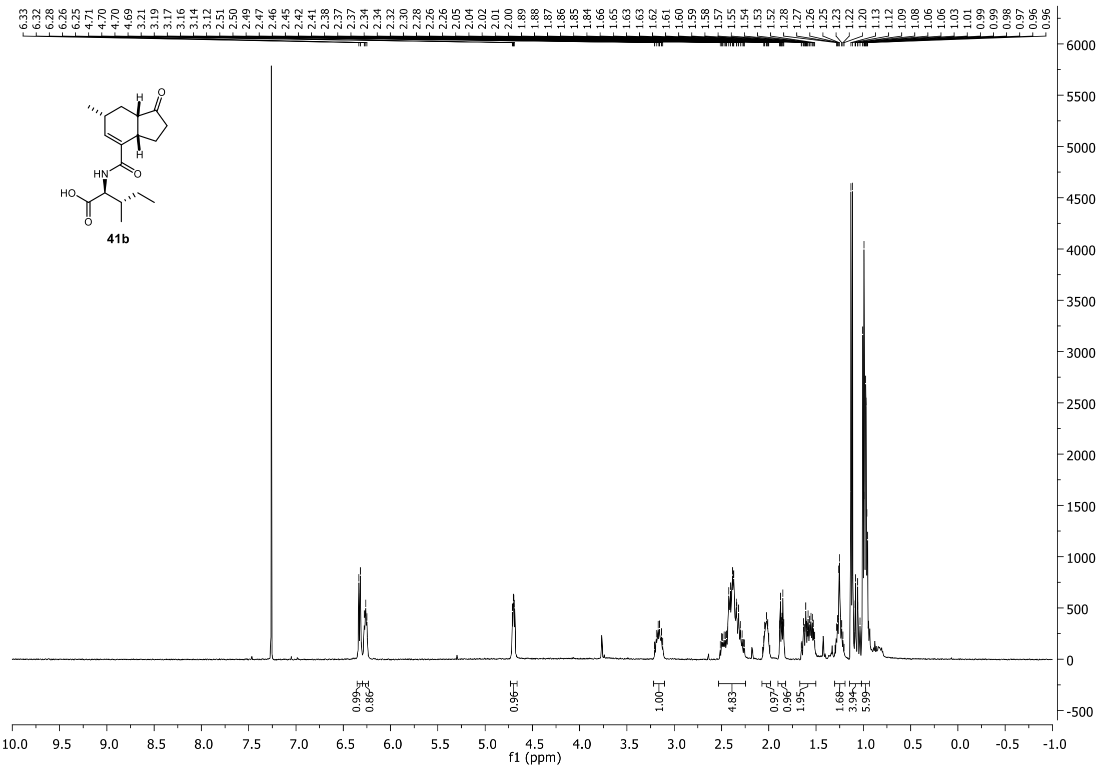

Supplementary Figure 189: ^{13}C NMR 41b.

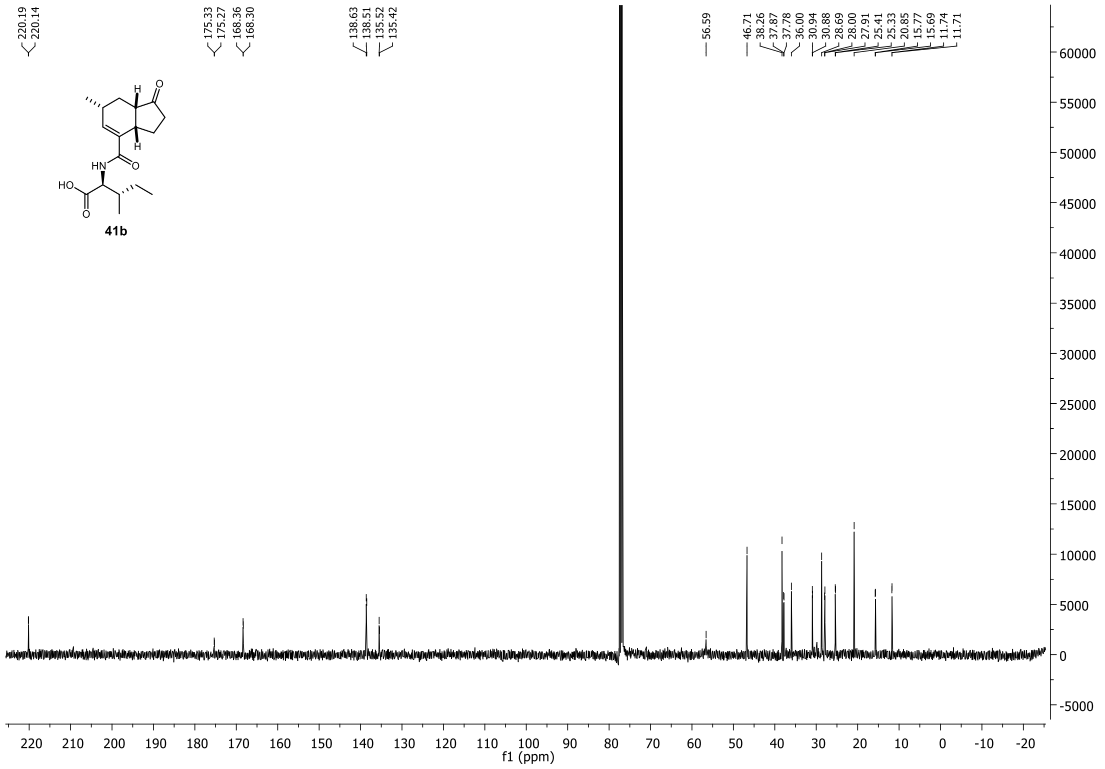

Supplementary Figure 190: ^{1}H NMR S71.

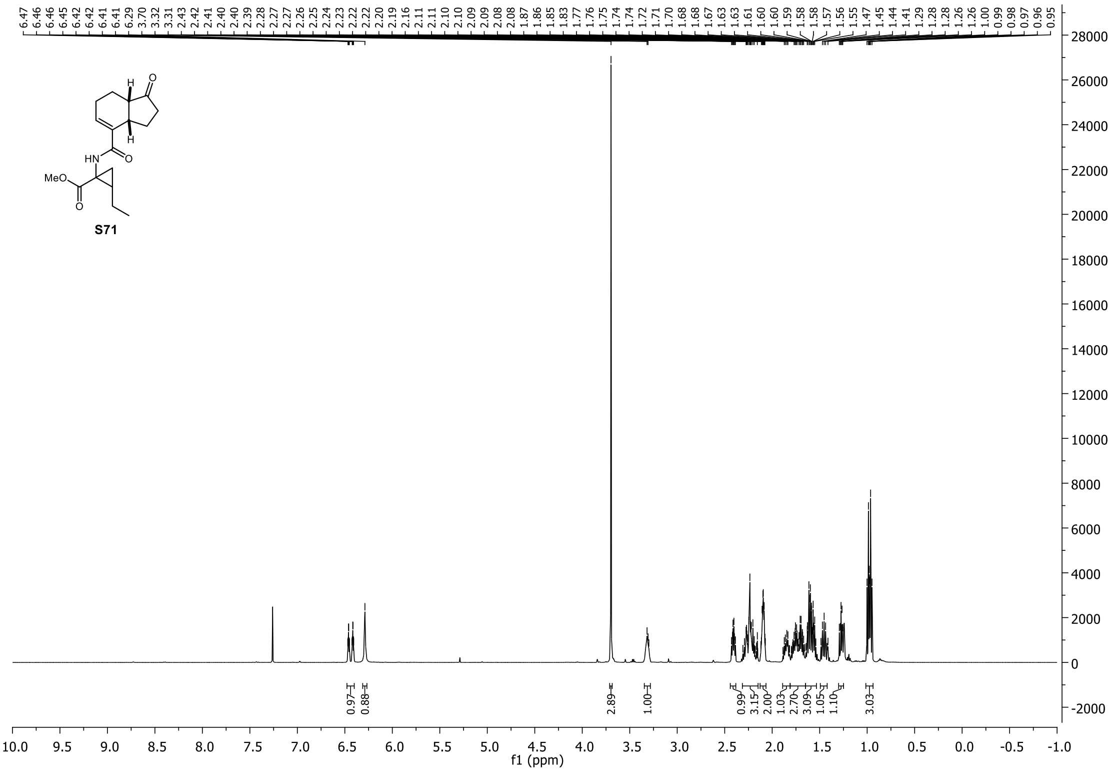

Supplementary Figure 191: ^{13}C NMR S71.

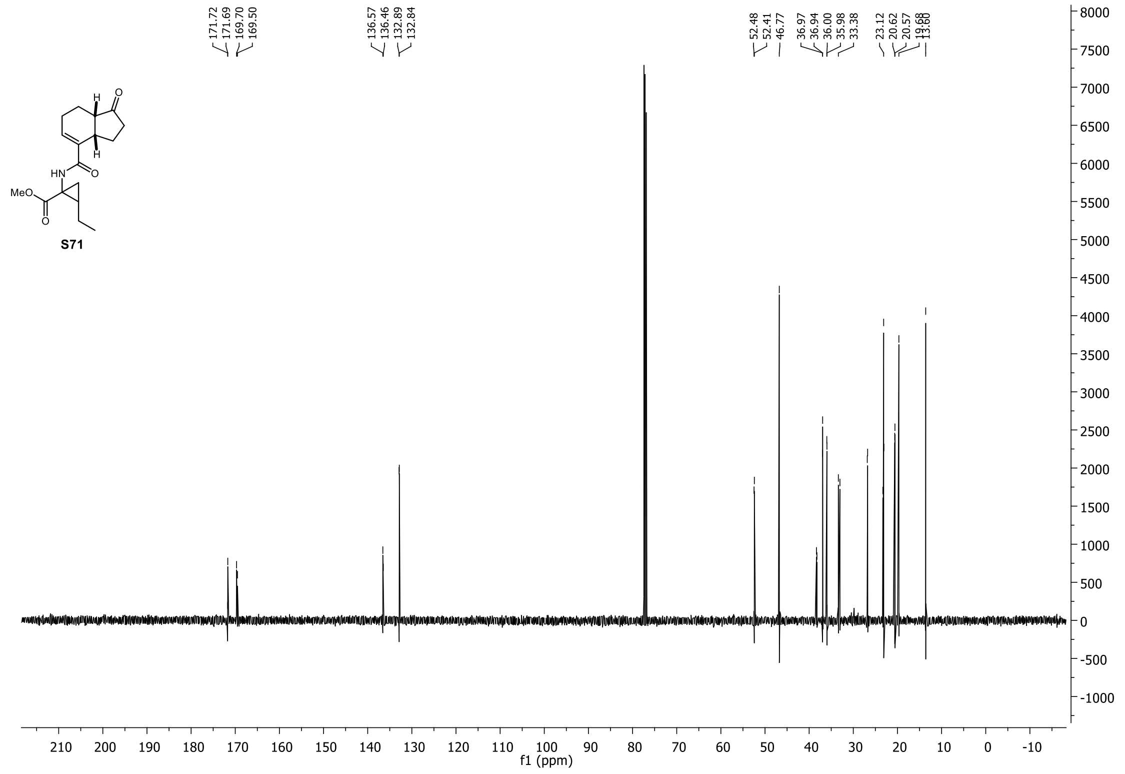

Supplementary Figure 192: ^{1}H NMR 42a.

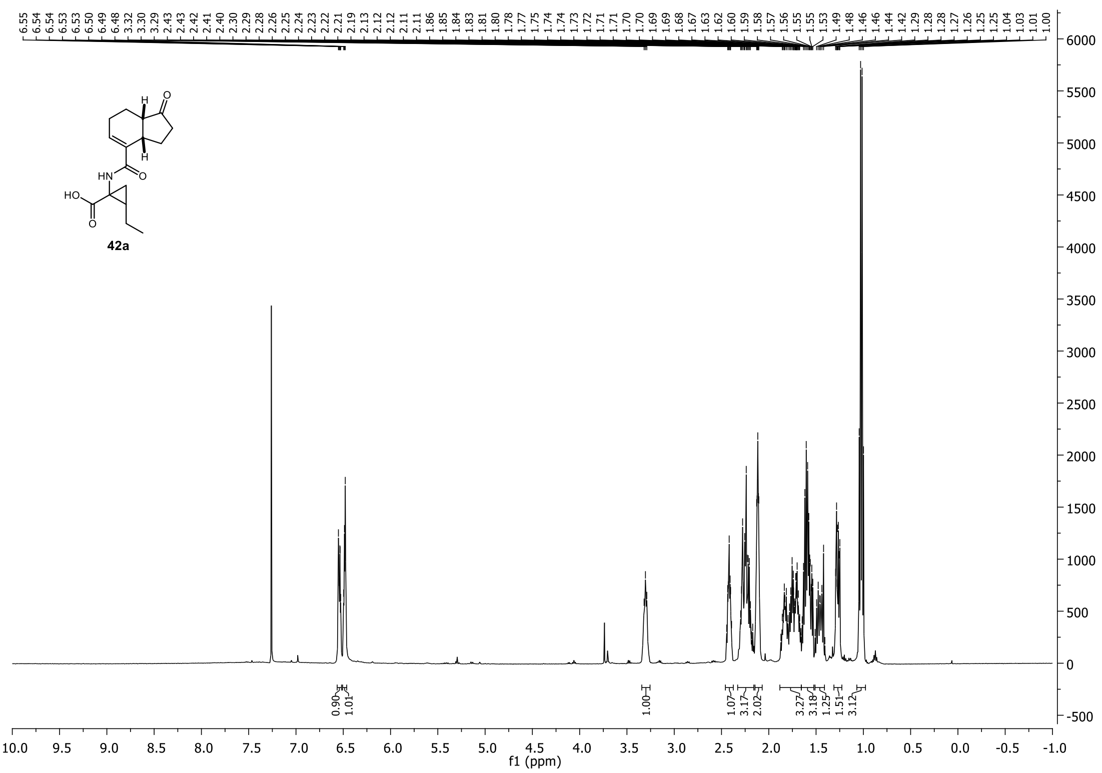

Supplementary Figure 193: ^{13}C NMR 42a.

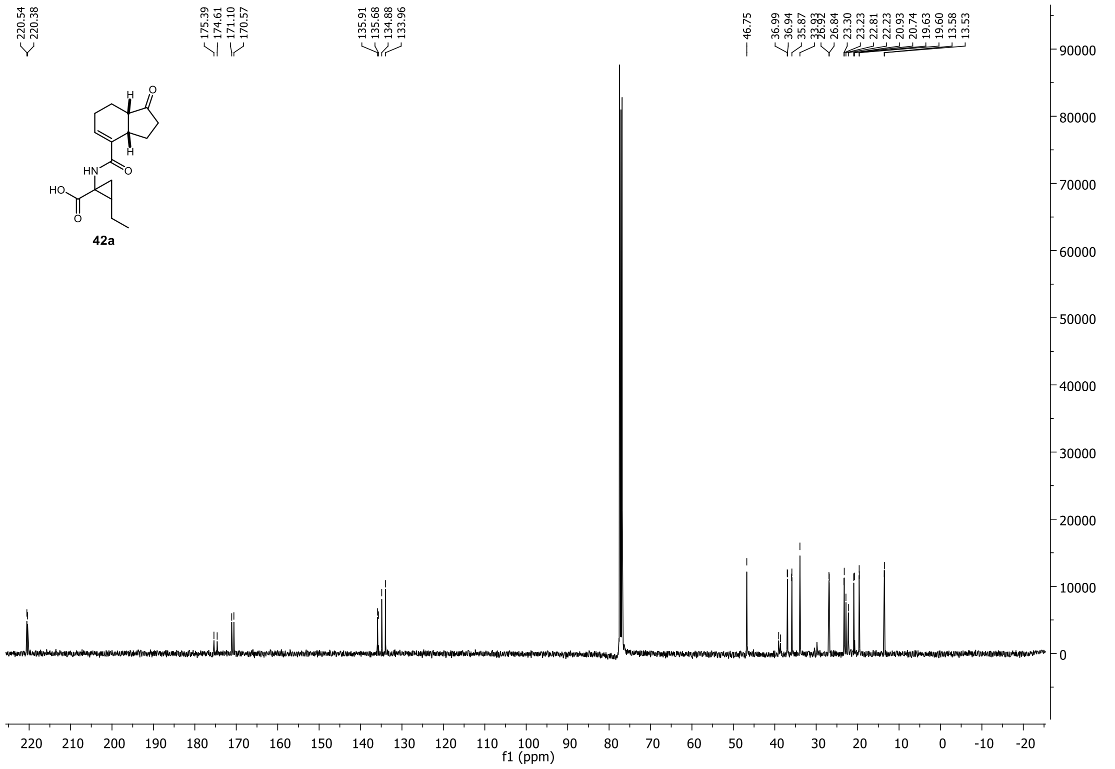

Supplementary Figure 194: ^{1}H NMR S72.

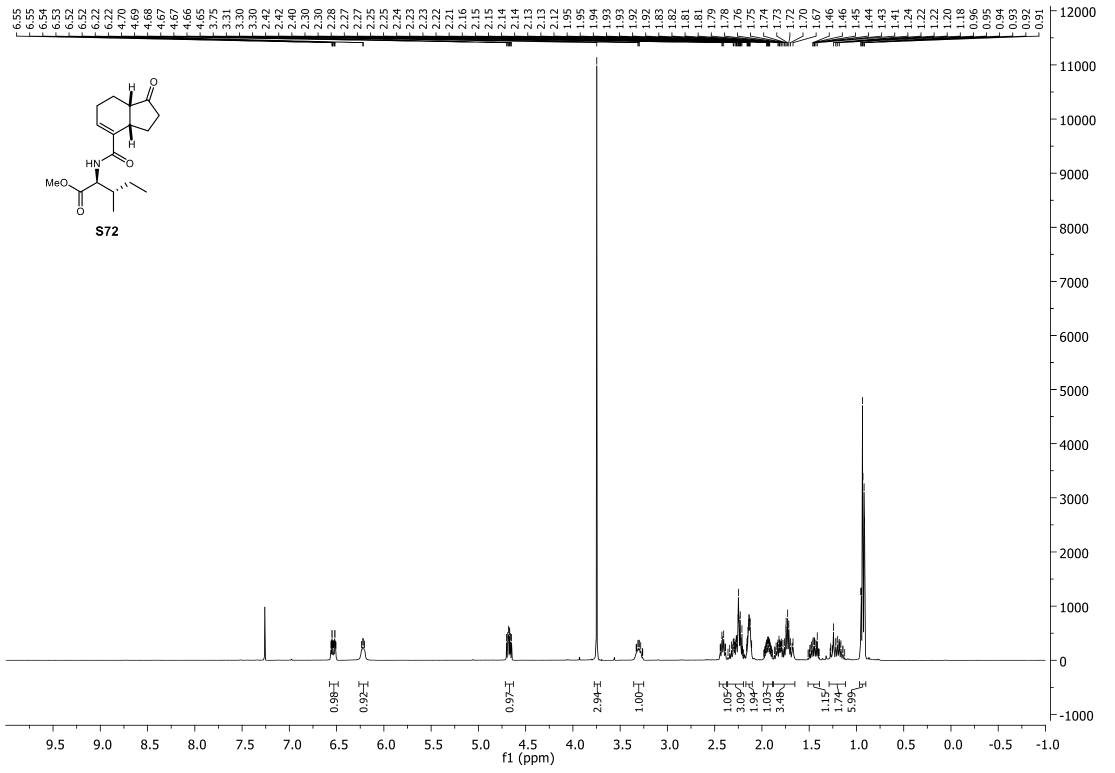

Supplementary Figure 195: ^{13}C NMR S72.

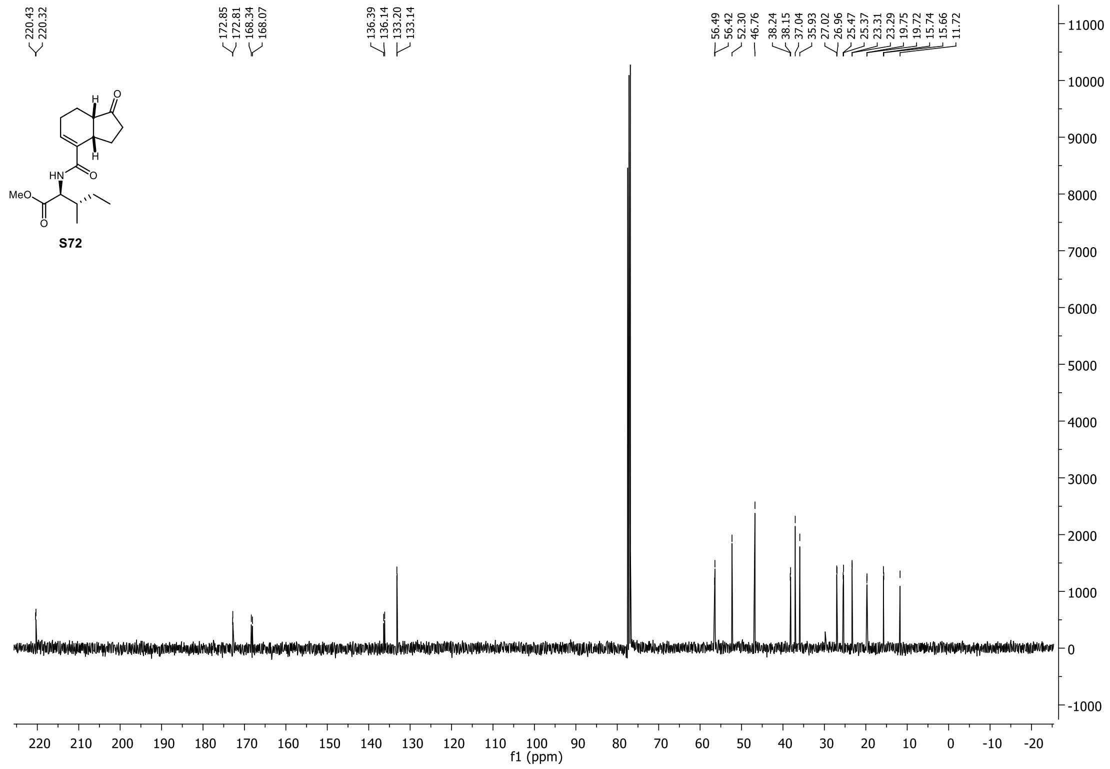

Supplementary Figure 196: ^{1}H NMR 42b.

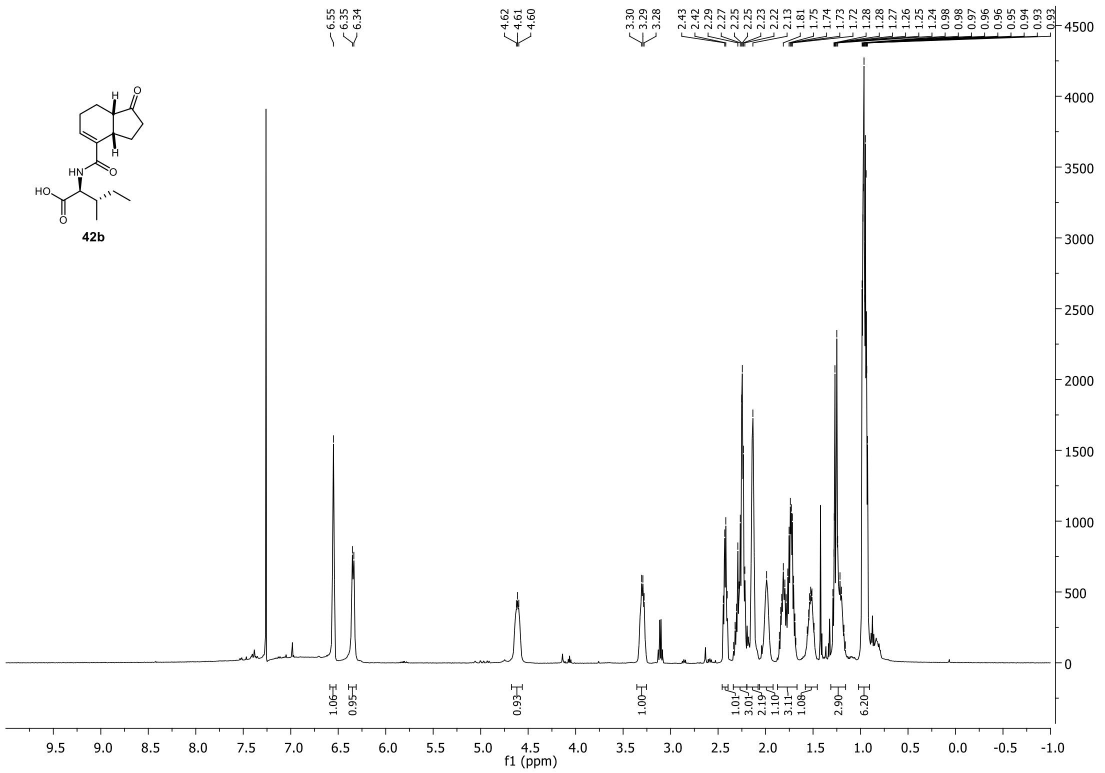

Supplementary Figure 197: ^{13}C NMR 42b.

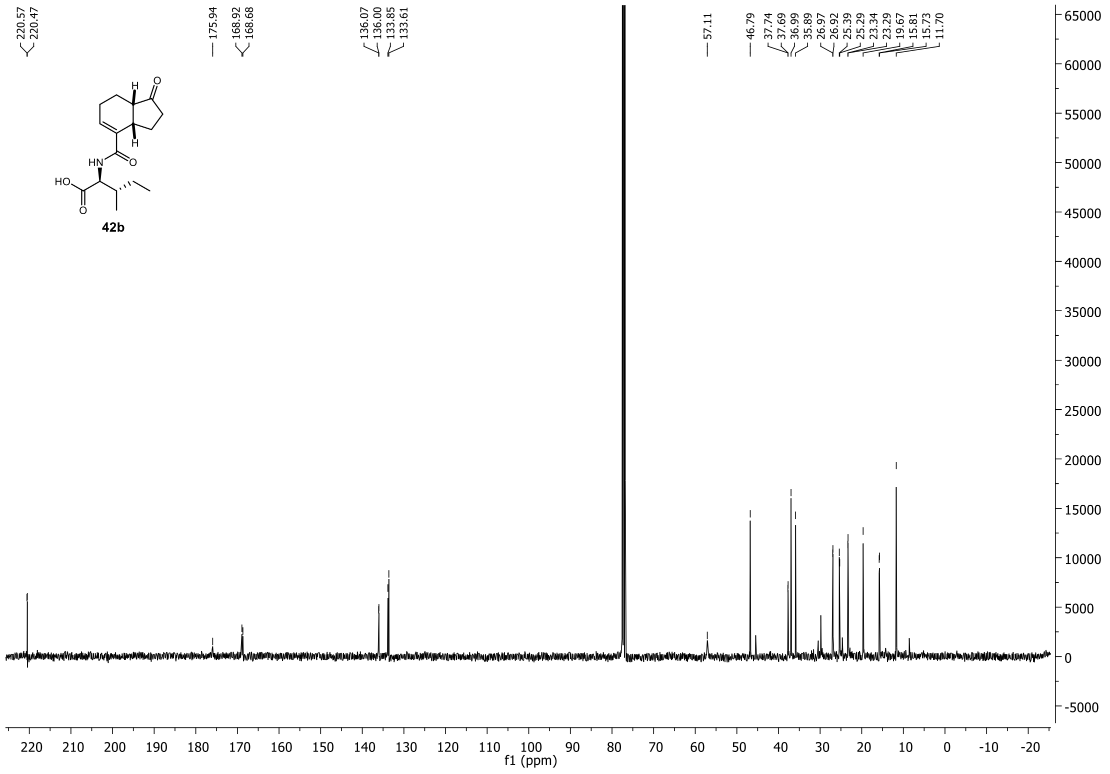

Supplementary Figure 198: ^{1}H NMR S71.

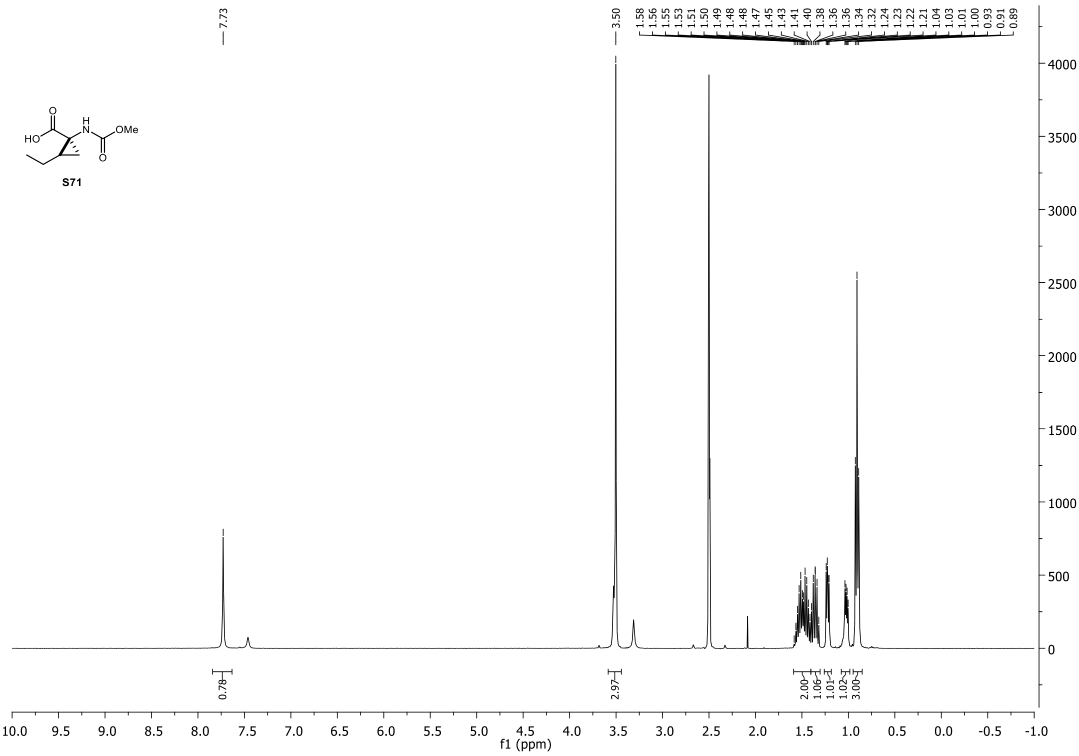

Supplementary Figure 199: ^{13}C NMR S71.

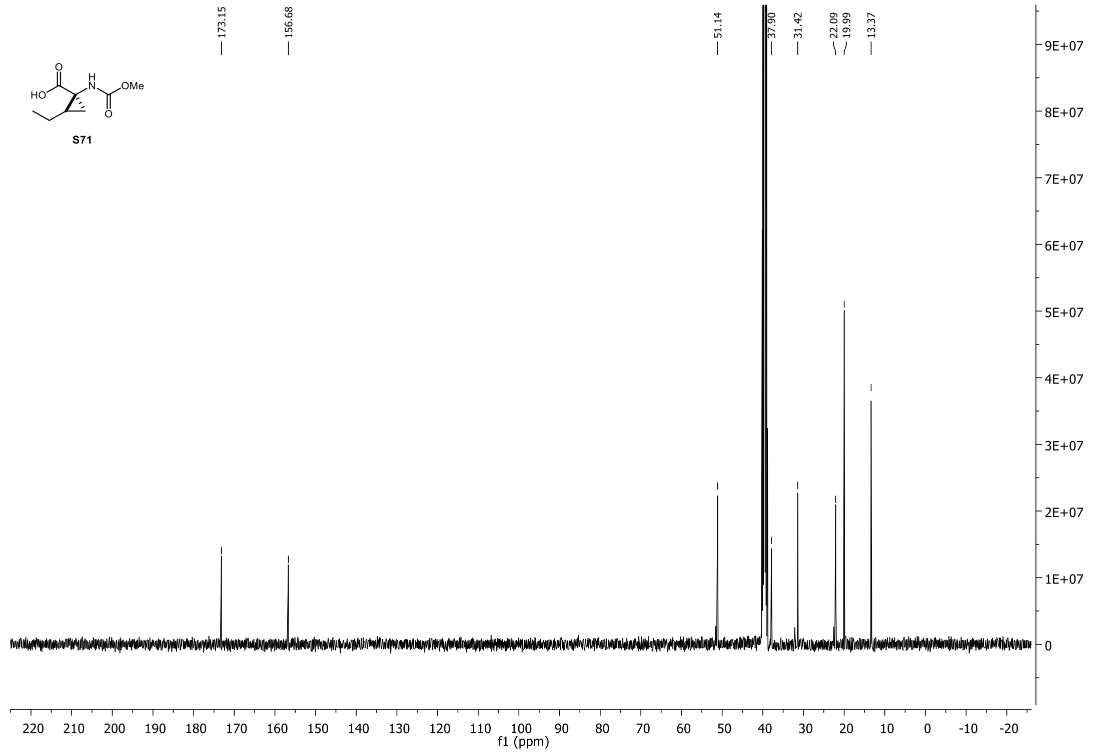

Supplementary Figure 200: ^{1}H NMR S72.

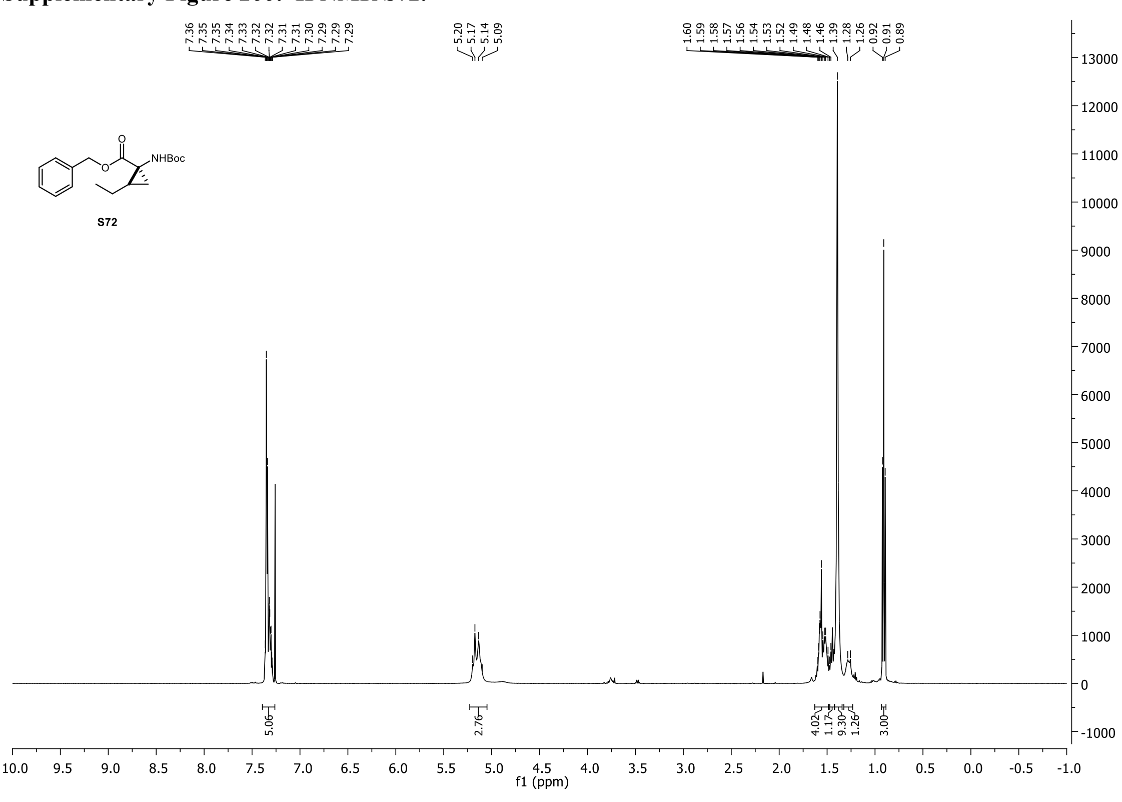

Supplementary Figure 201: ^{13}C NMR S72.

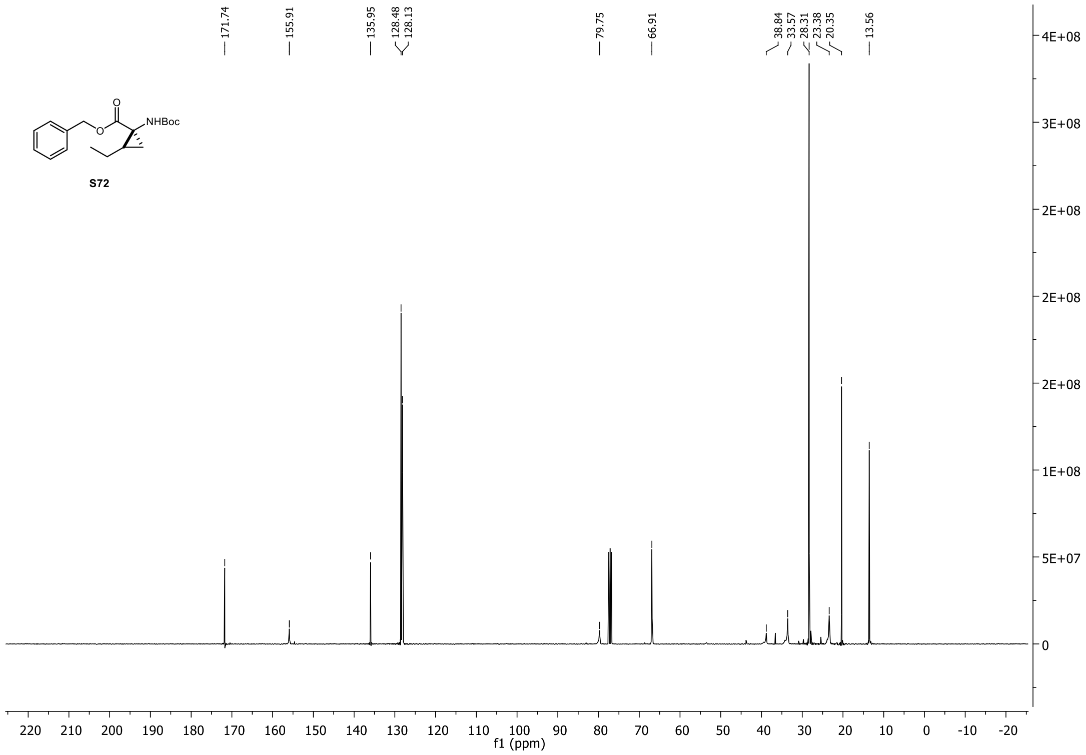

Supplementary Figure 202: ^{1}H NMR S73.

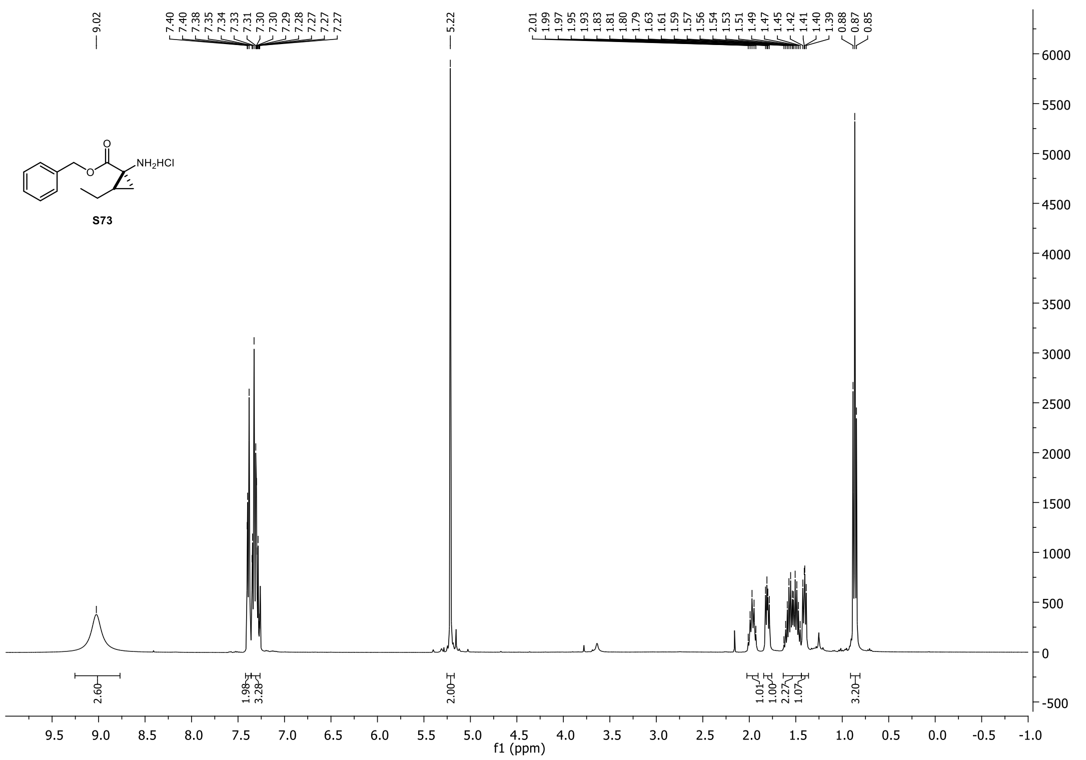

Supplementary Figure 203: ^{13}C NMR S73.

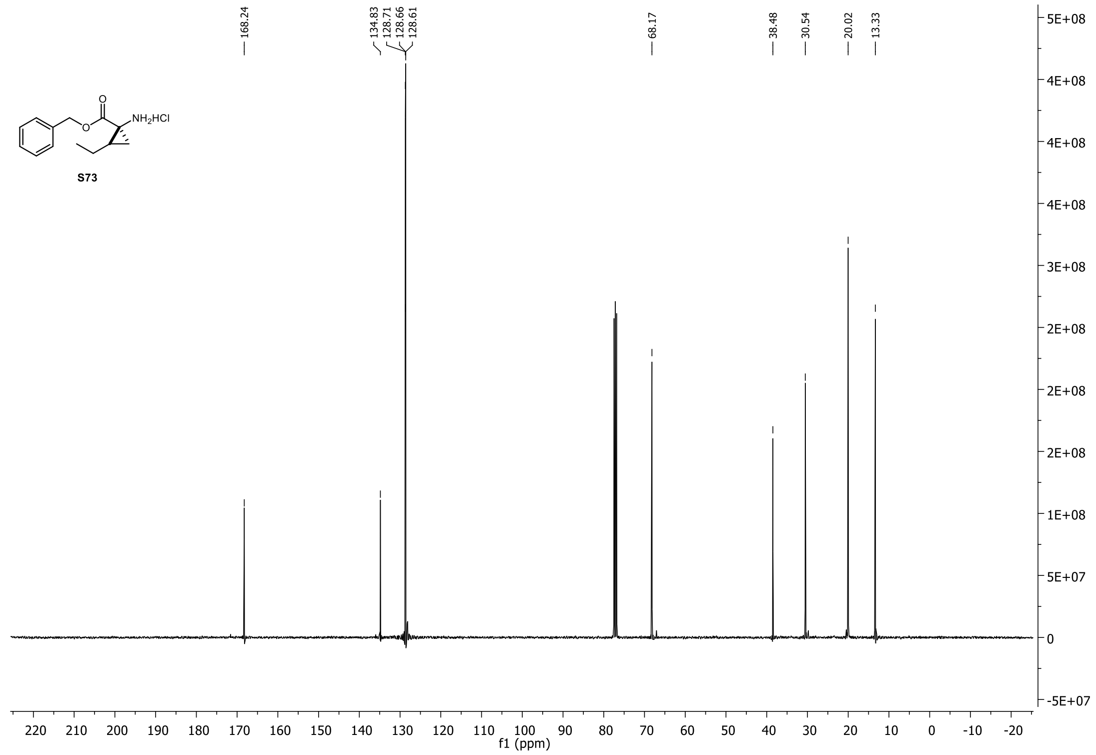

Supplementary Figure 204: ^{1}H NMR S74.

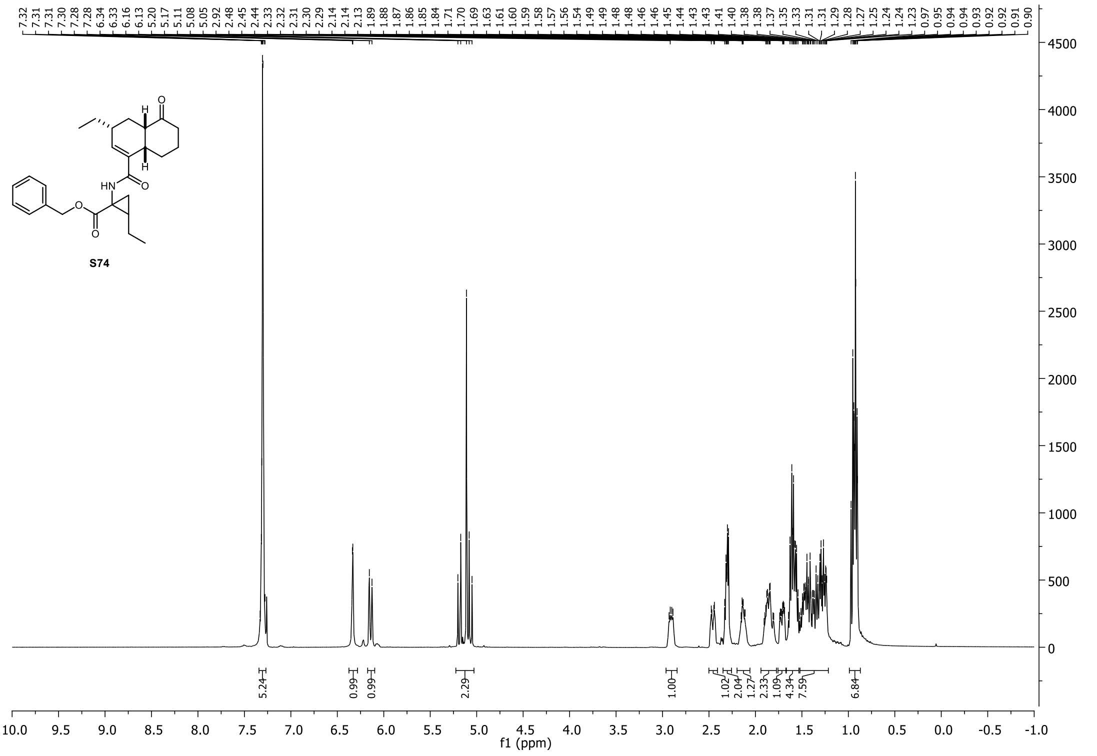

Supplementary Figure 205: ^{13}C NMR S74.

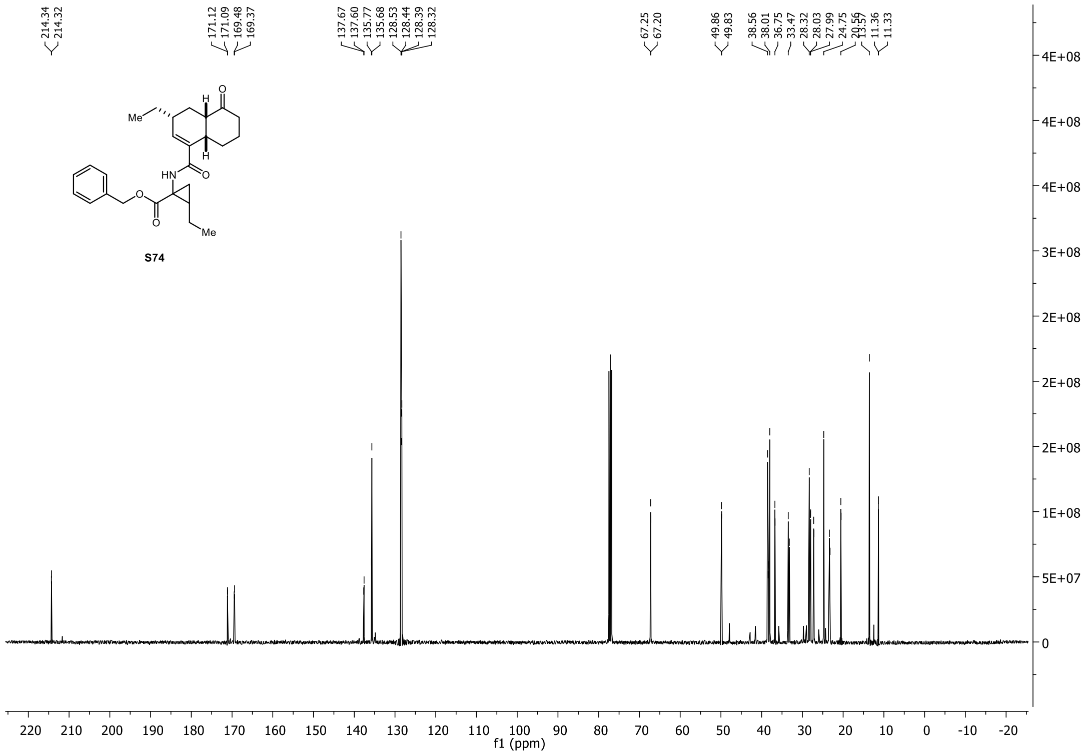

Supplementary Figure 206: ^{1}H NMR 43a.

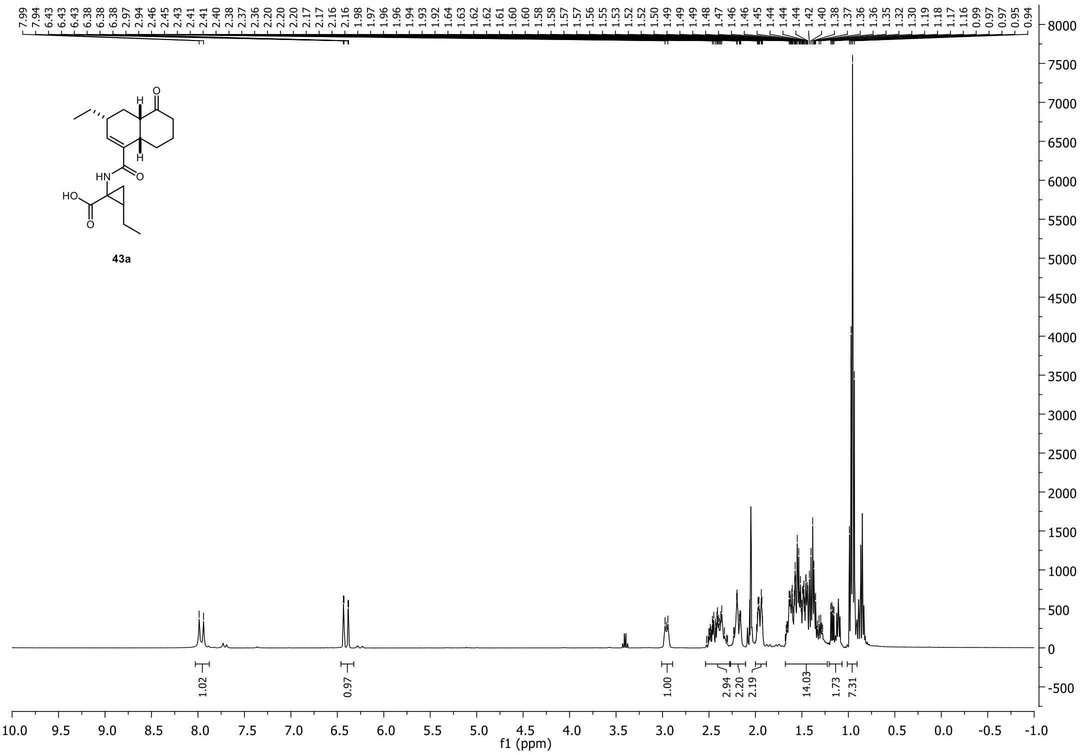

Supplementary Figure 207: ^{13}C NMR 43a.

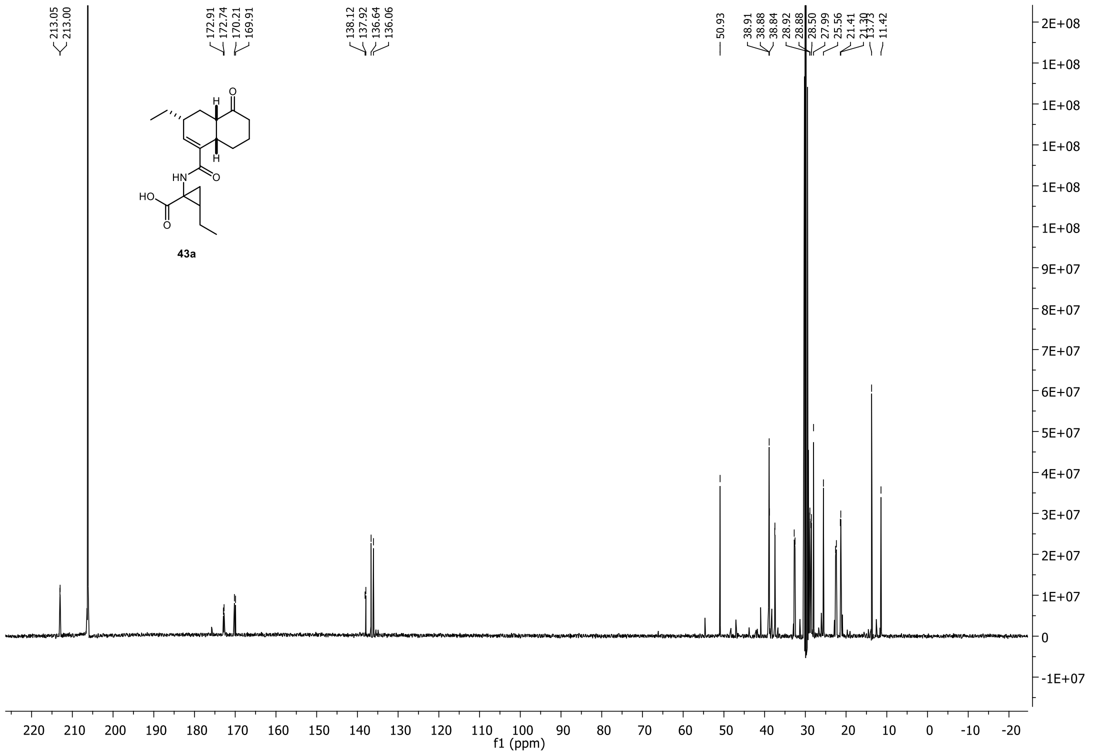

Supplementary Figure 208: ^{1}H NMR S75.

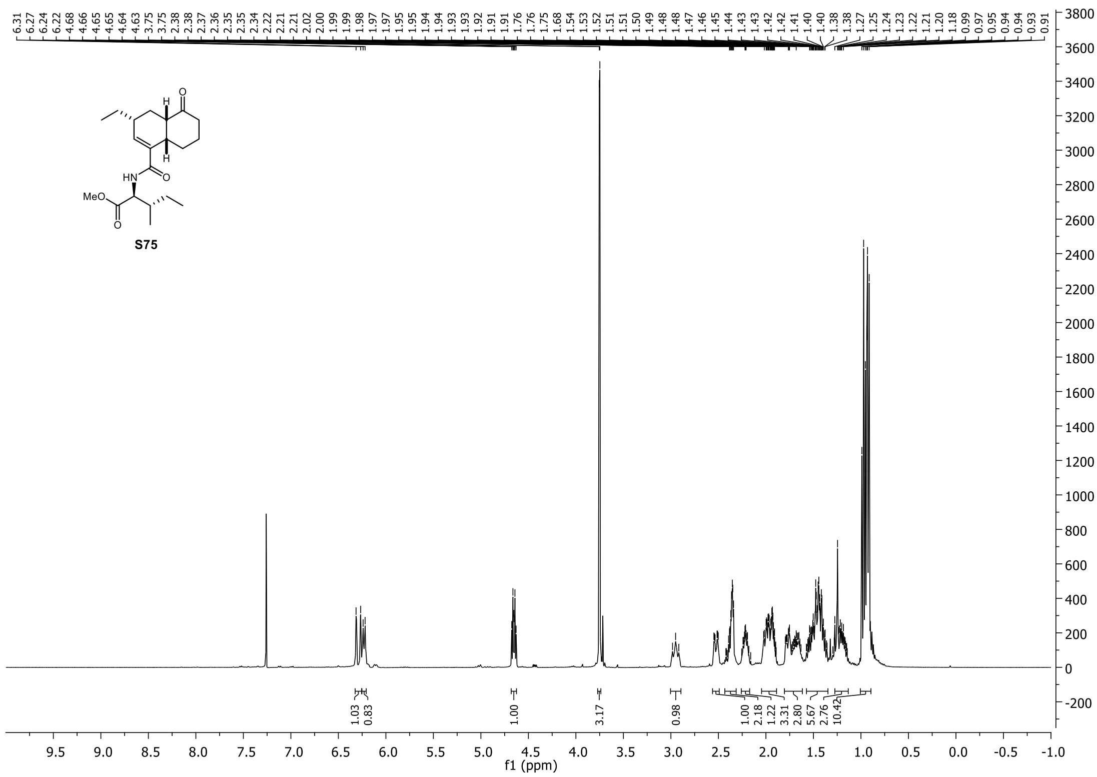

Supplementary Figure 209: ^{13}C NMR S75.

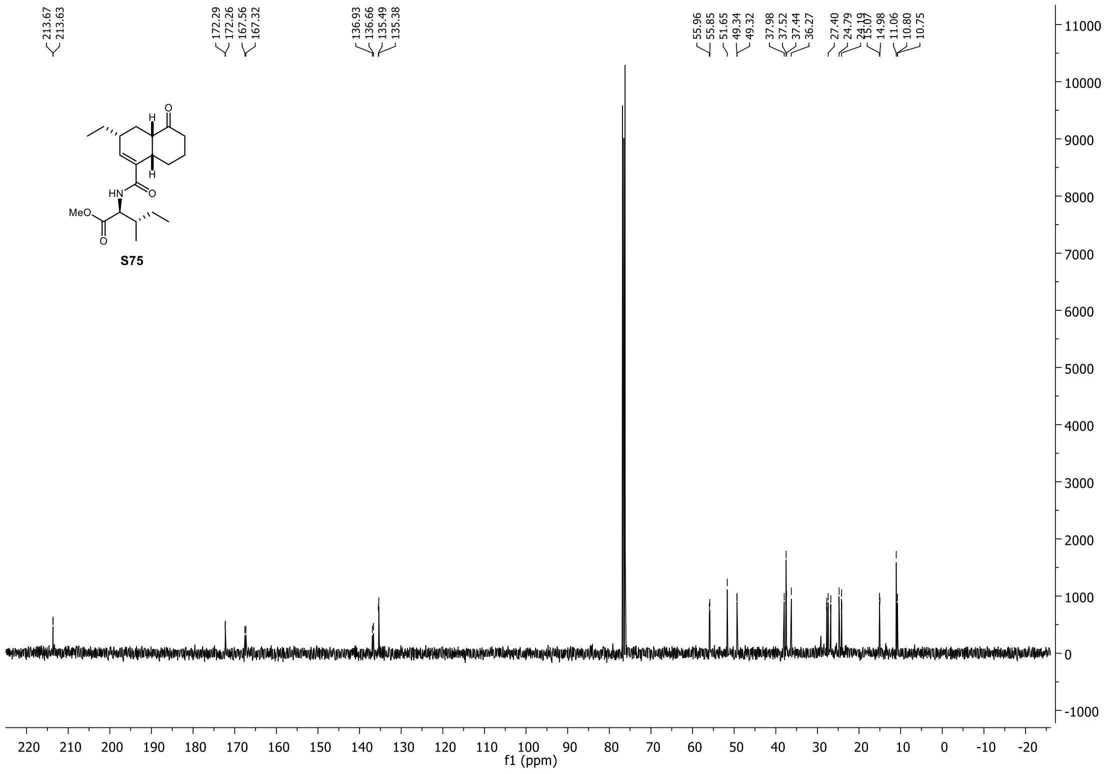

Supplementary Figure 210: ^{1}H NMR 43b.

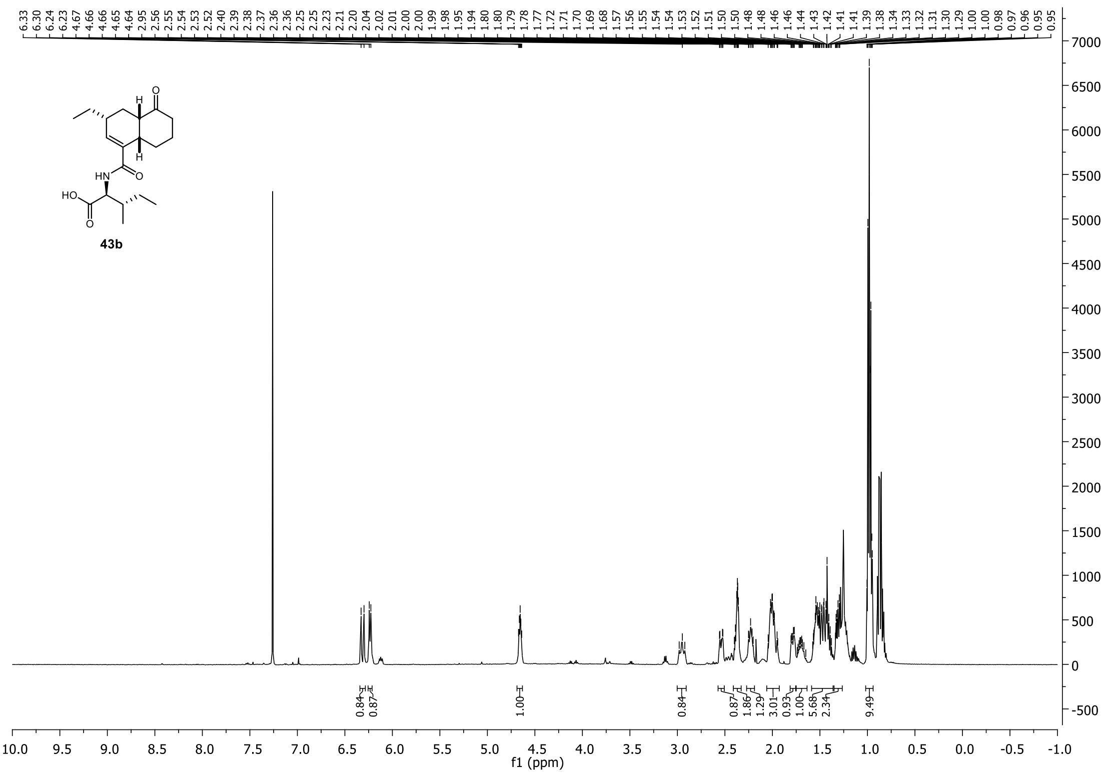

Supplementary Figure 211: ^{13}C NMR 43b.

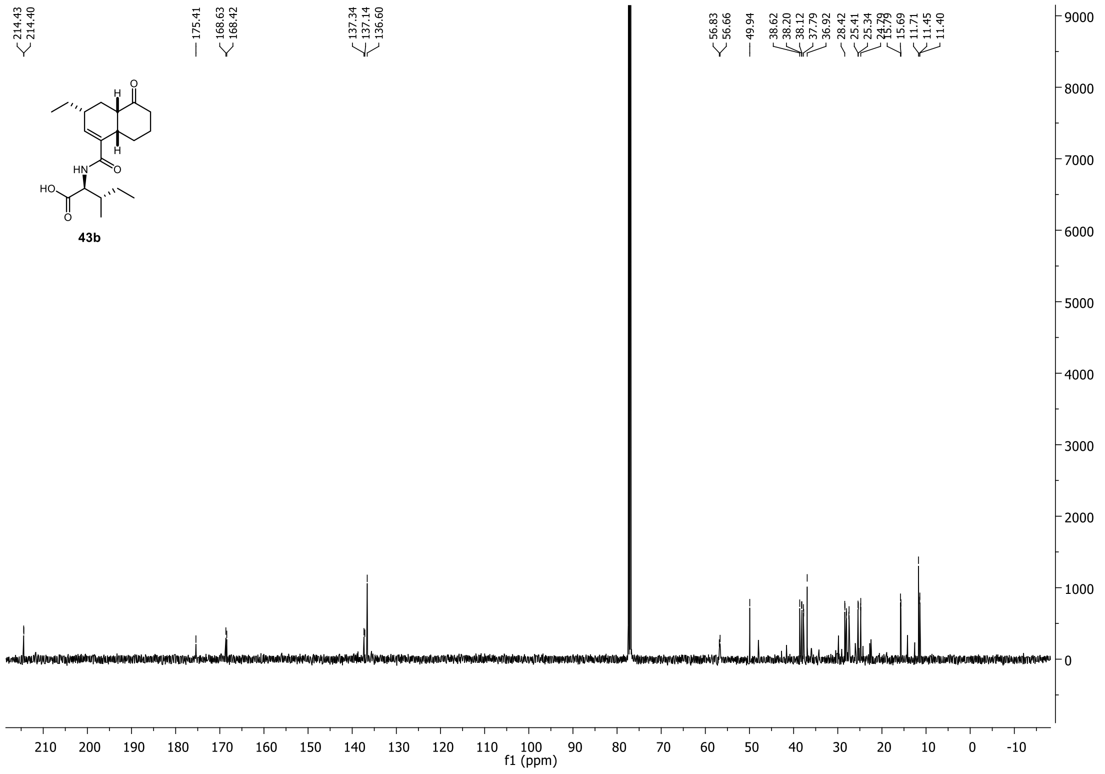

Supplementary Figure 212: ^{1}H NMR 44a.

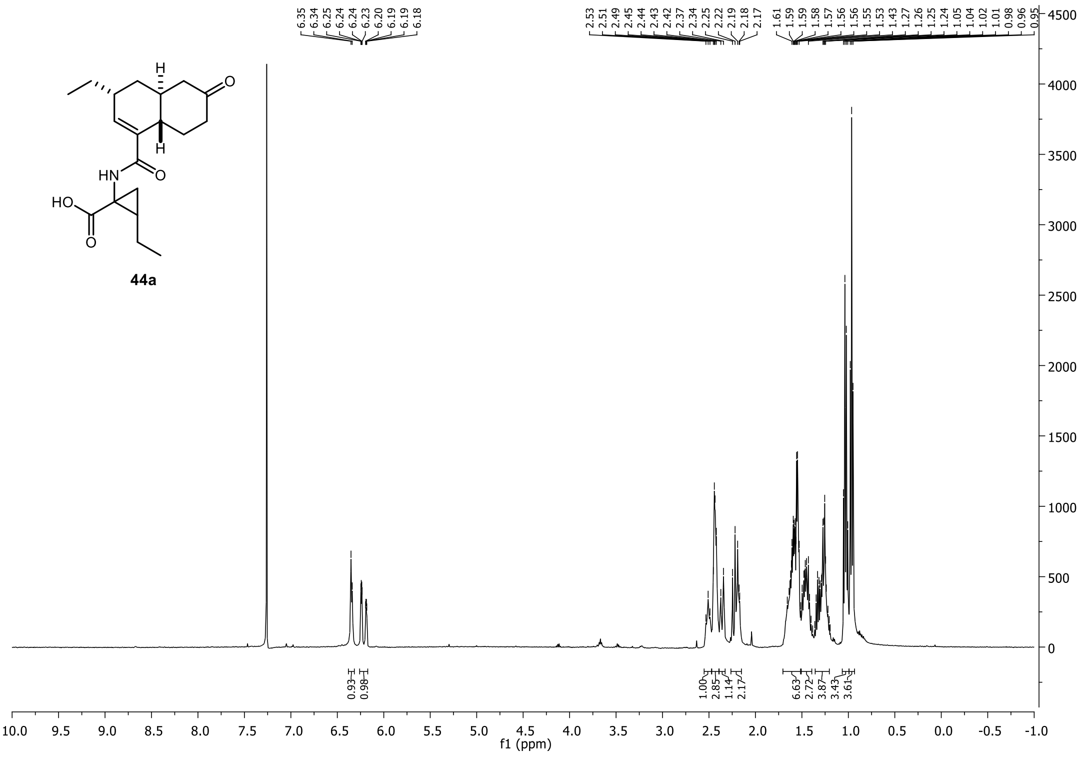

Supplementary Figure 213: ^{13}C NMR 44a.

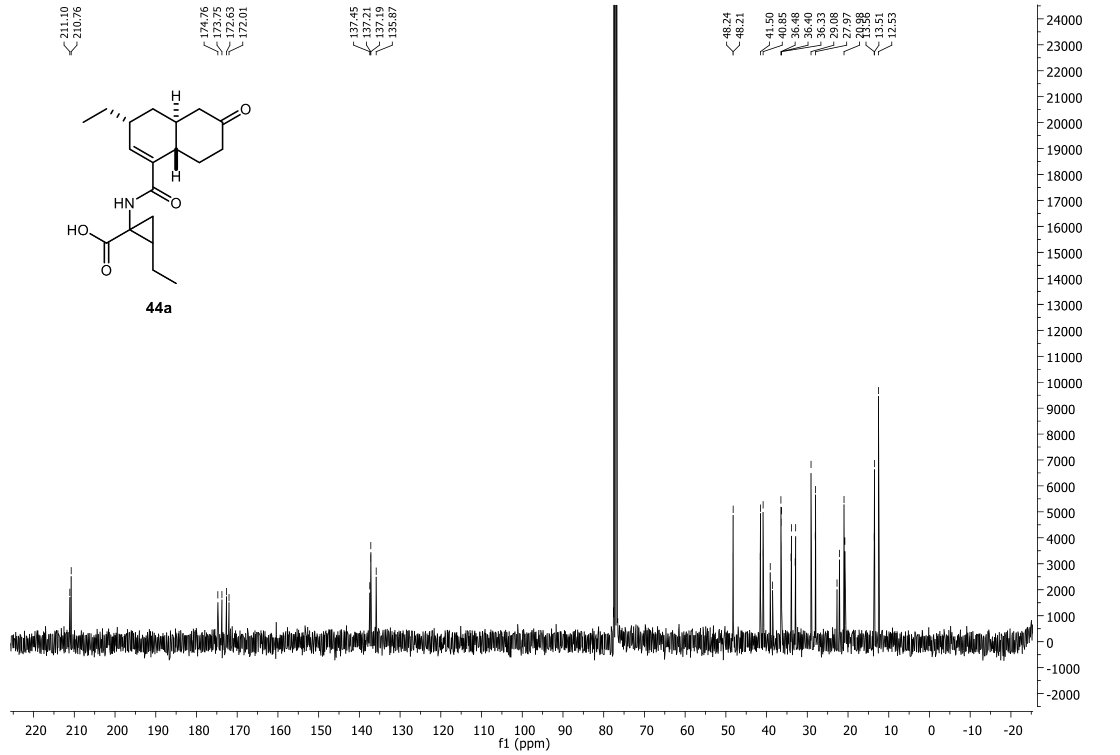

Supplementary Figure 214: ^{1}H NMR S76.

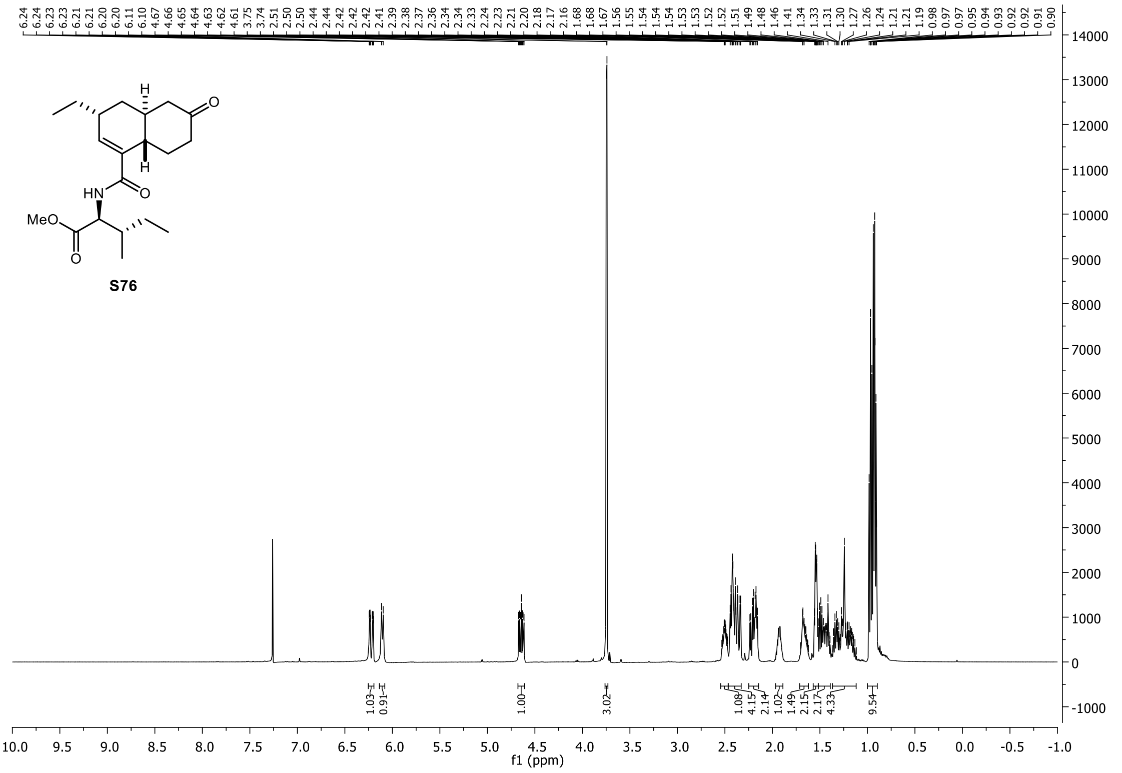

Supplementary Figure 215: ^{13}C NMR S76.

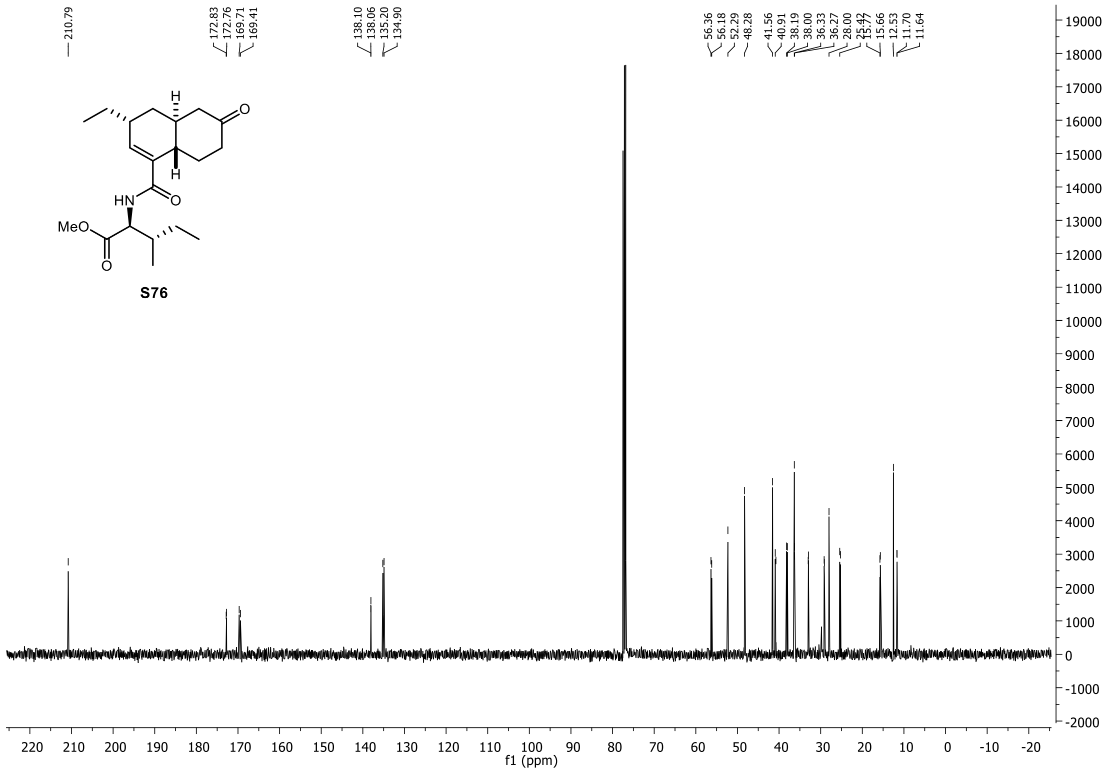

Supplementary Figure 216: ^{1}H NMR 44b.

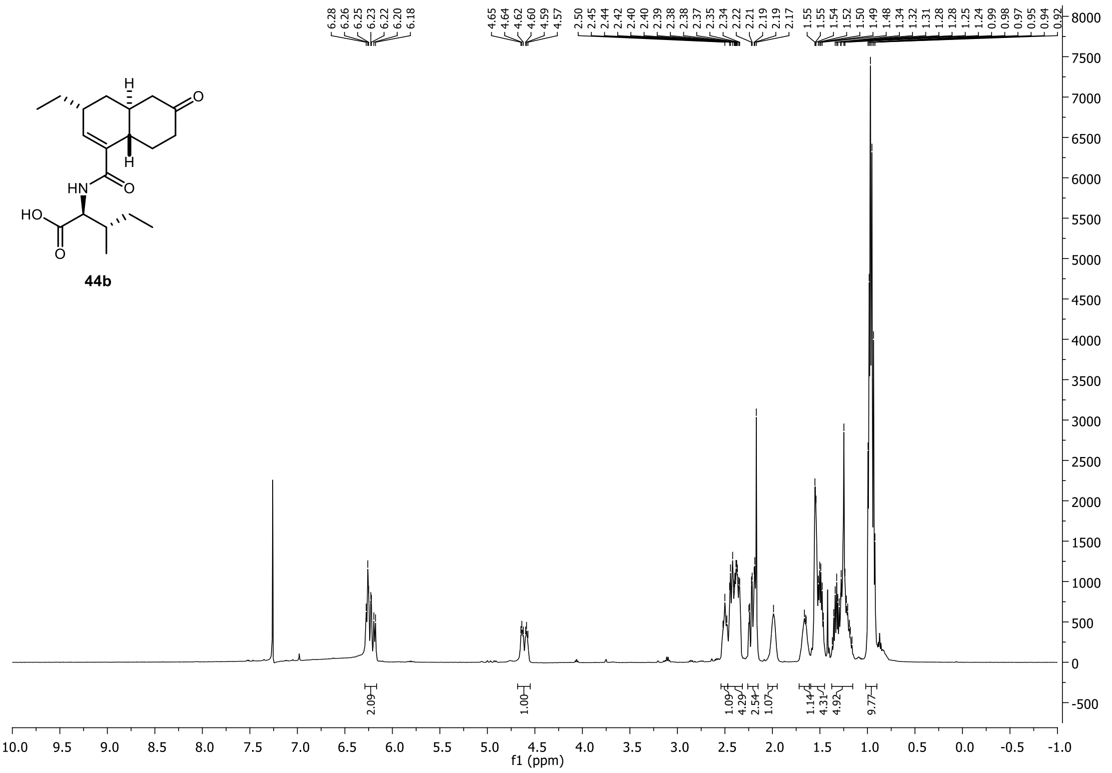

Supplementary Figure 217: ^{13}C NMR 44b.

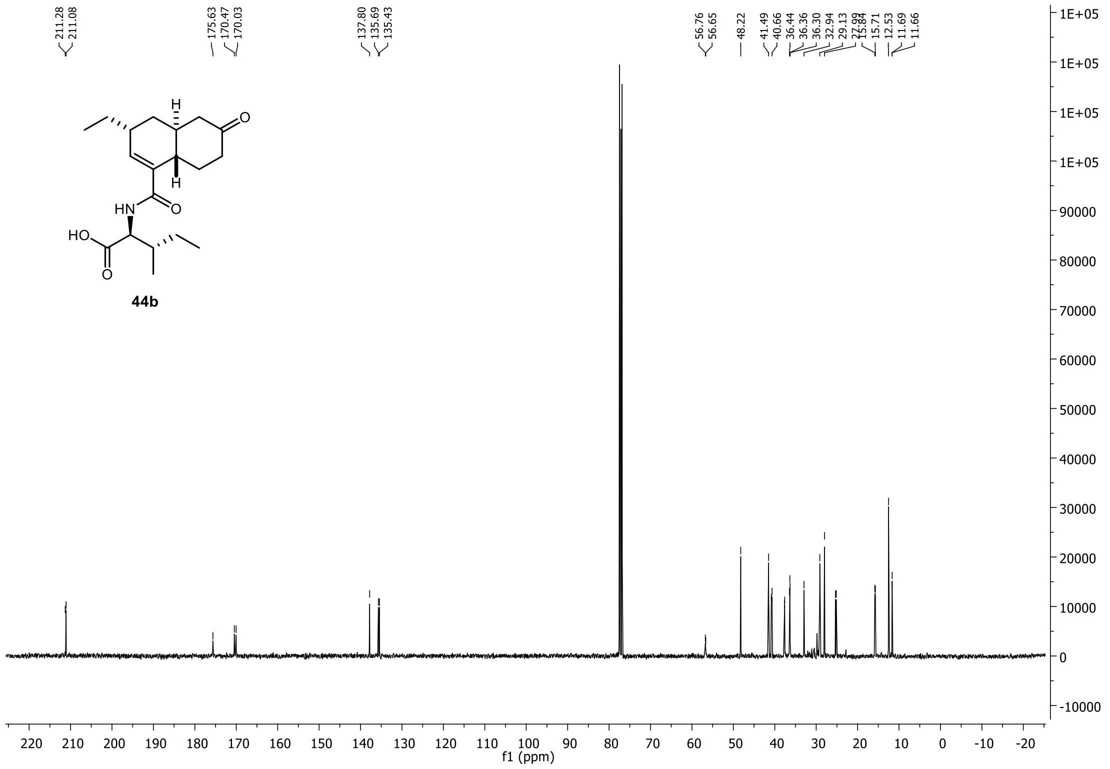

Supplementary Figure 218: ^{1}H NMR S78.

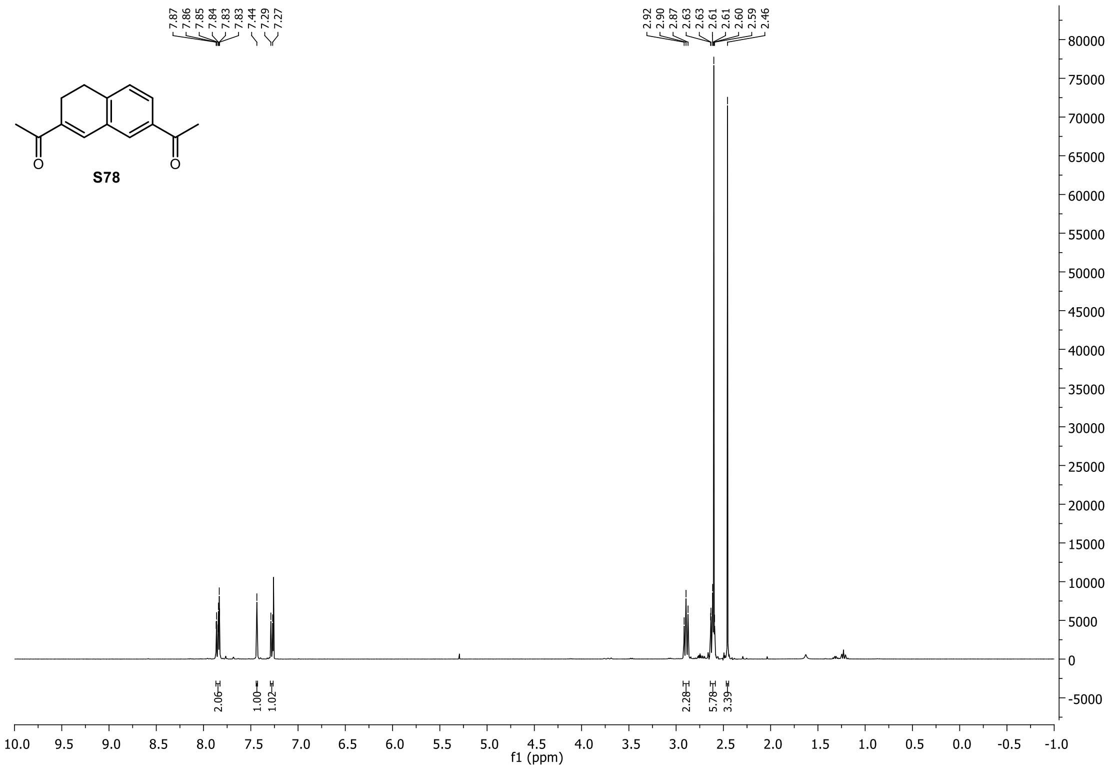

Supplementary Figure 219: ^{13}C NMR S78.

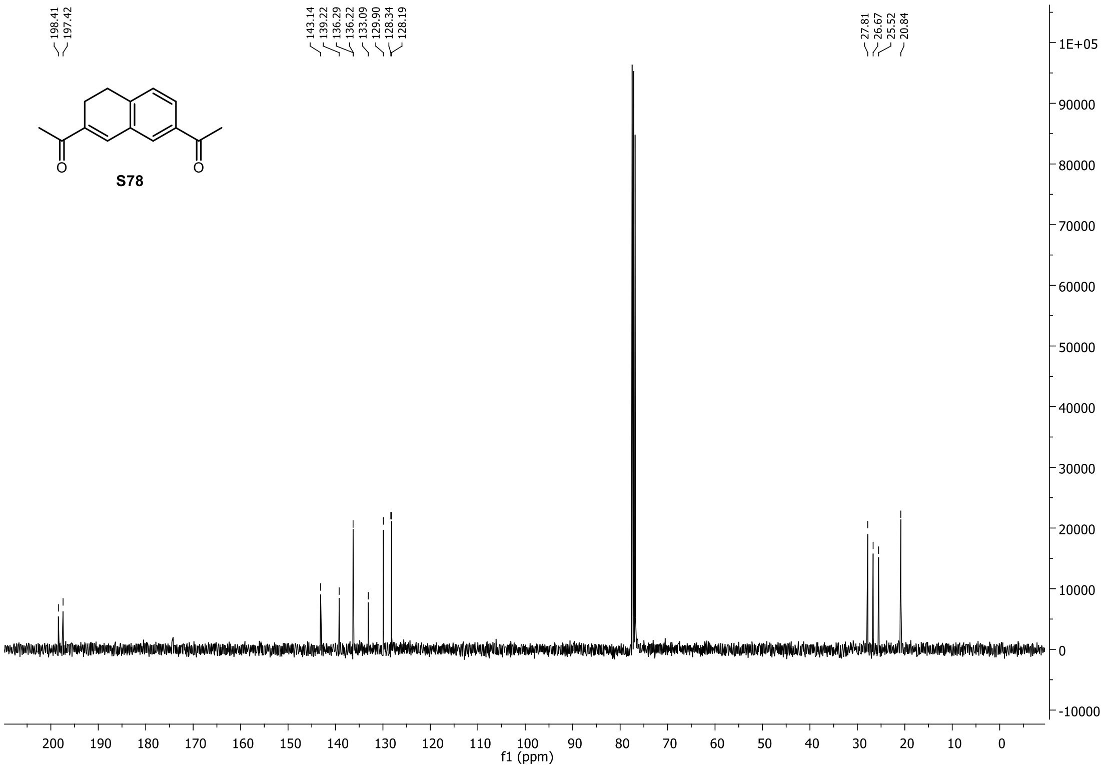

Supplementary Figure 220: ^{1}H NMR S79.

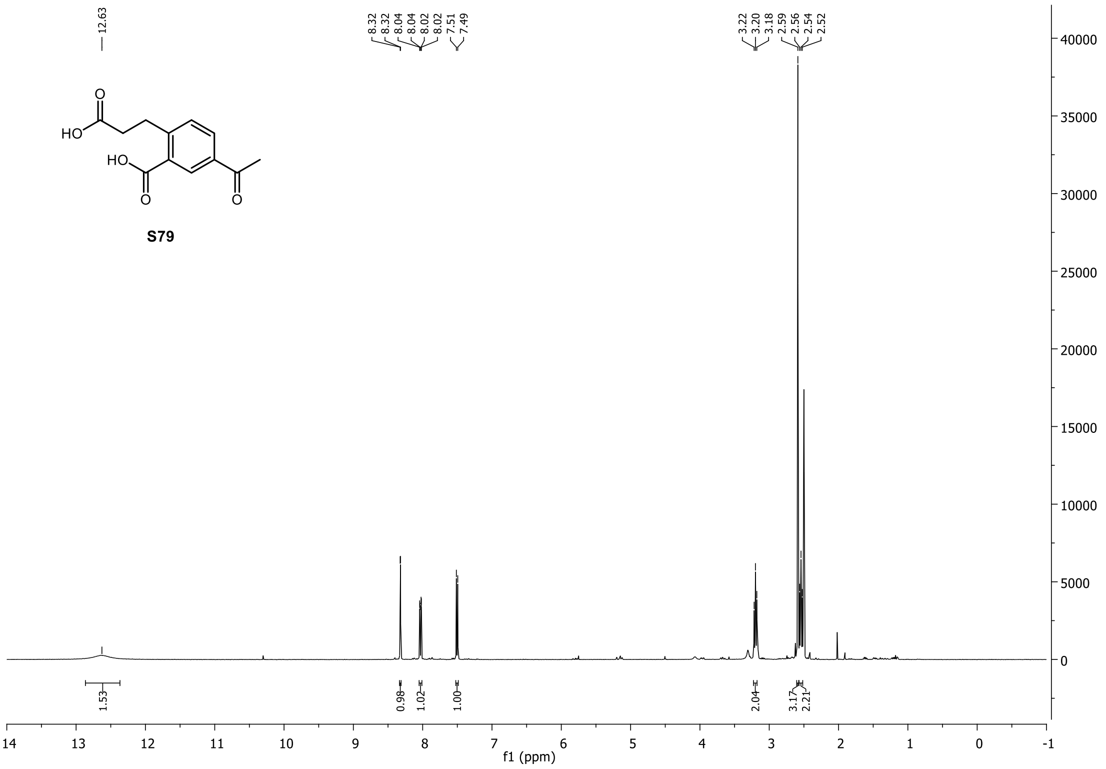

Supplementary Figure 221: ^{13}C NMR S79.

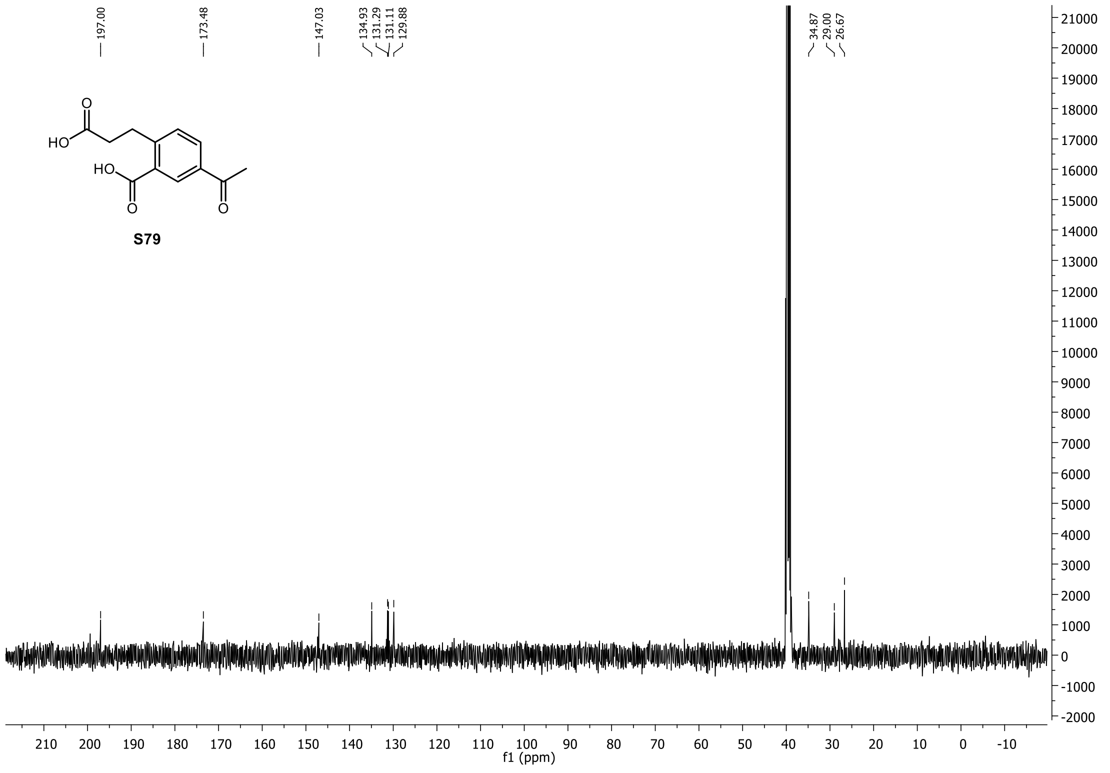

Supplementary Figure 222: ^{1}H NMR S80.

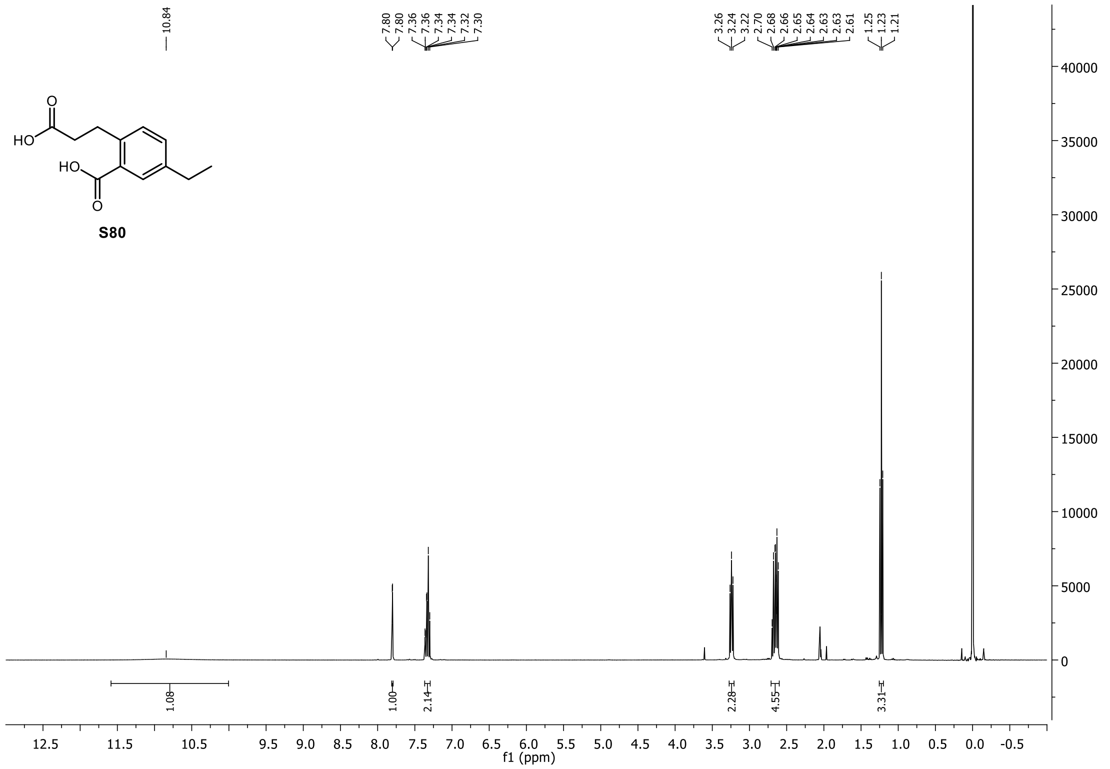

Supplementary Figure 223: ^{13}C NMR S80.

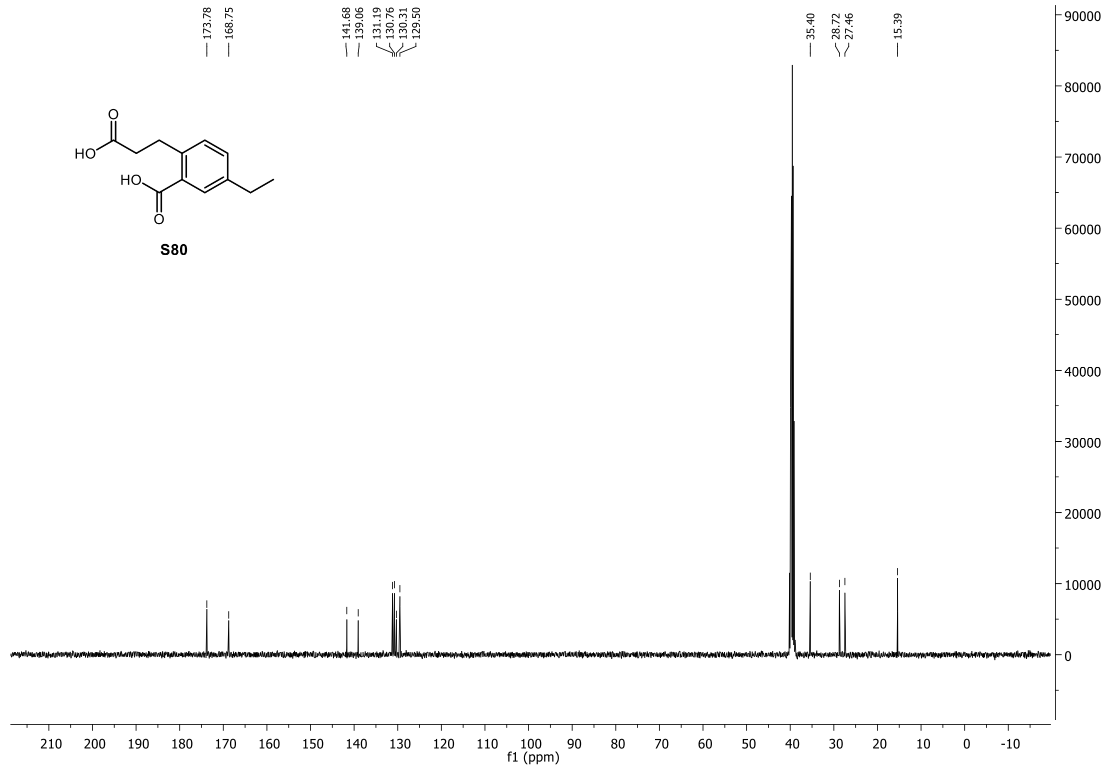

Supplementary Figure 224: ^{1}H NMR S81.

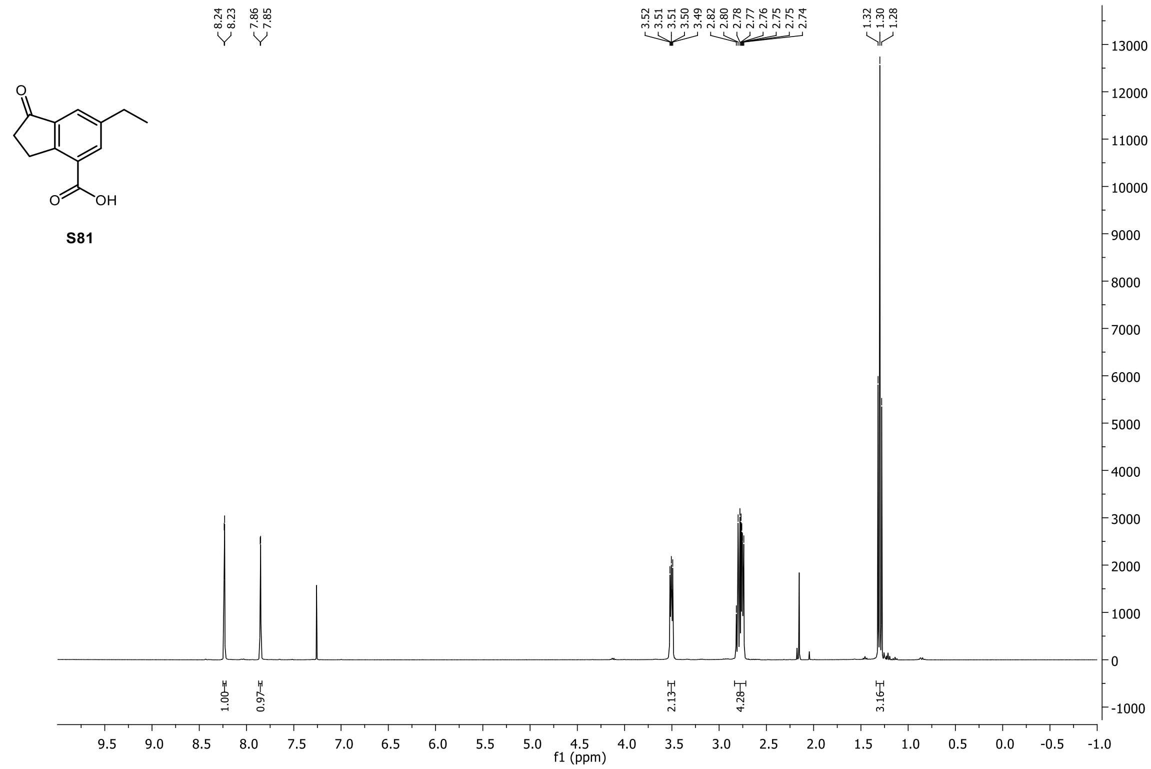

Supplementary Figure 225: ^{13}C NMR S81.

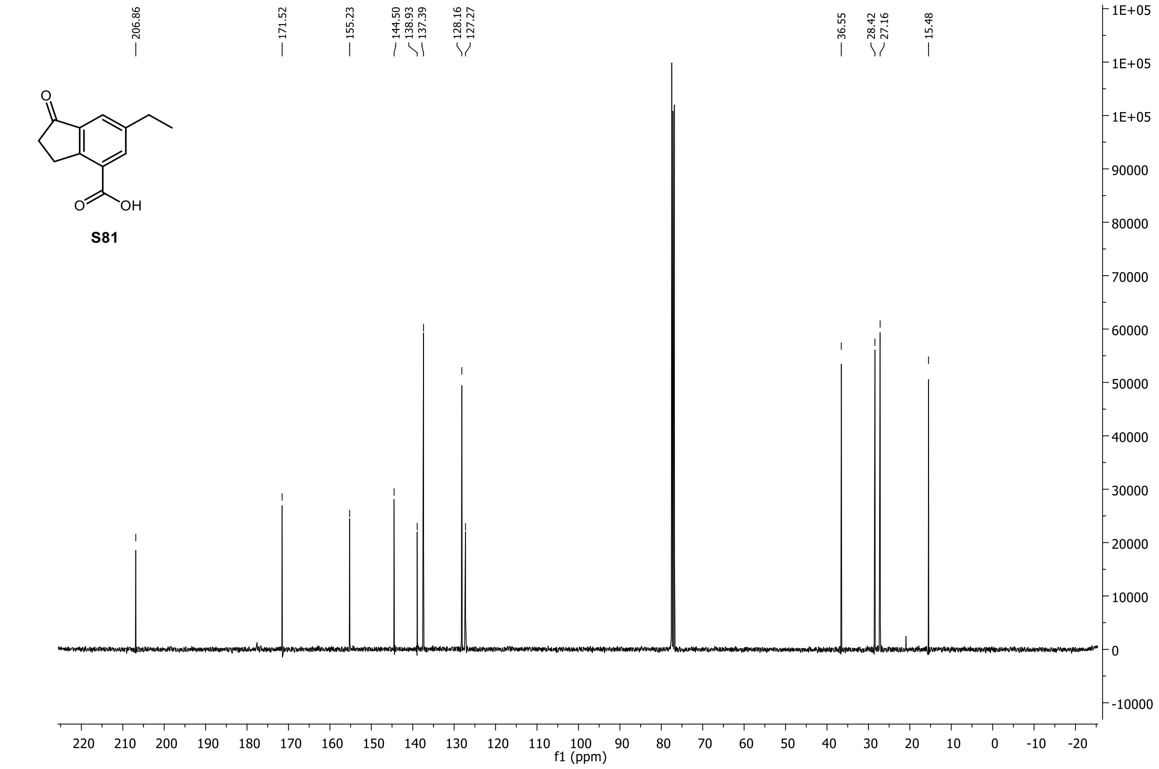

Supplementary Figure 226: ^{1}H NMR S82.

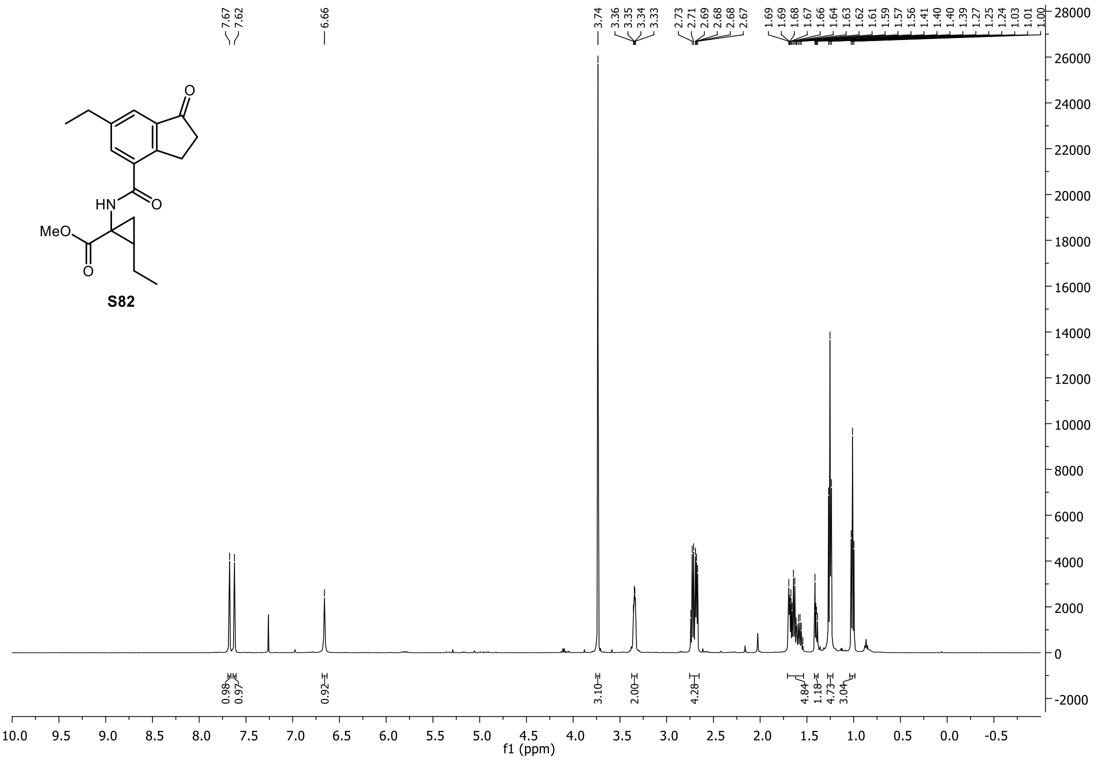

Supplementary Figure 227: ^{13}C NMR S82.

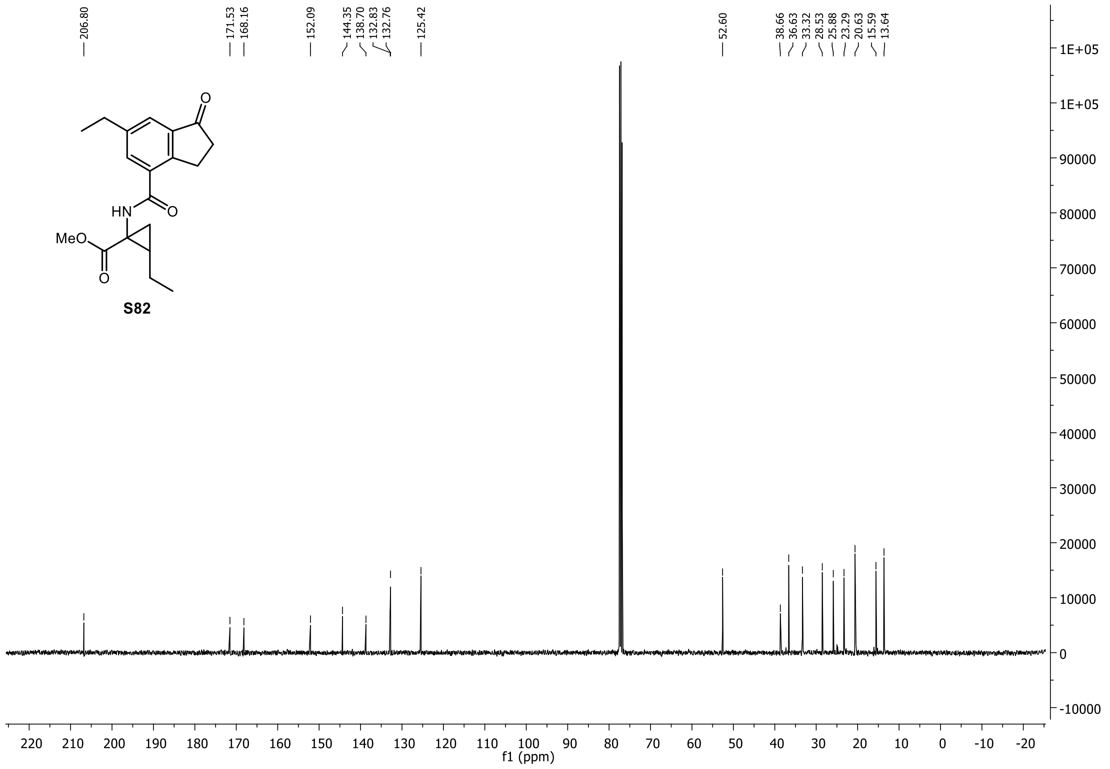

Supplementary Figure 228: ^{1}H NMR 45a.

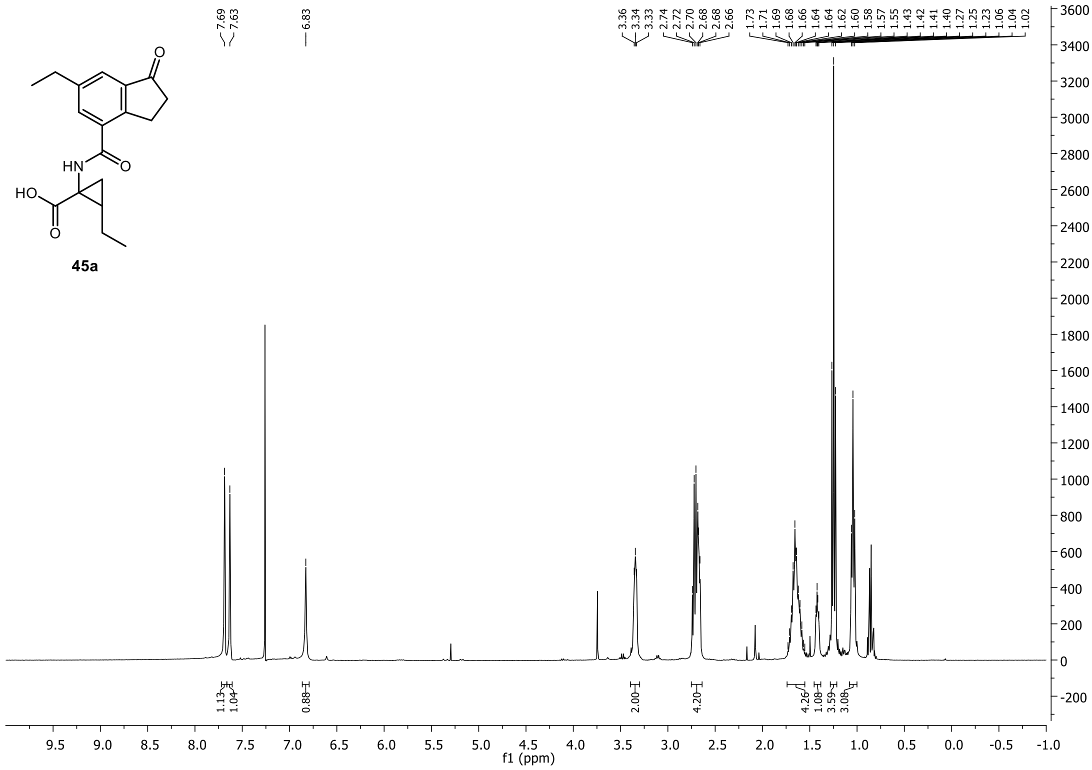

Supplementary Figure 229: ^{13}C NMR 45a.

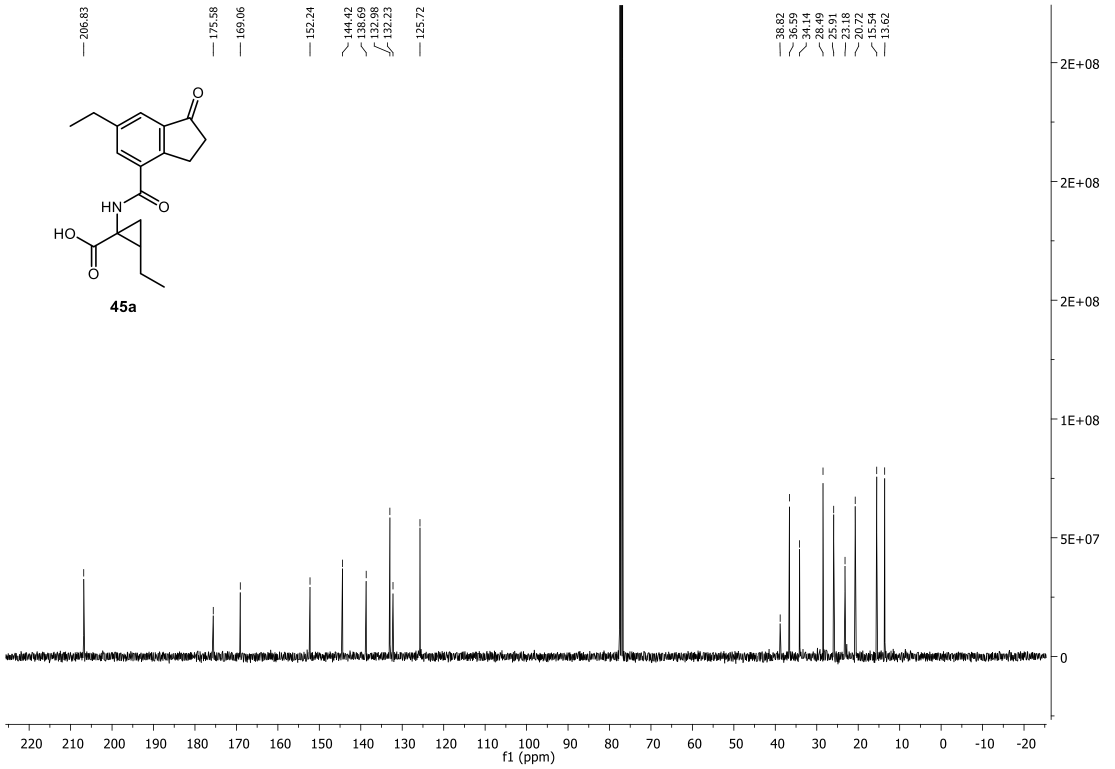

Supplementary Figure 230: ^{1}H NMR S83.

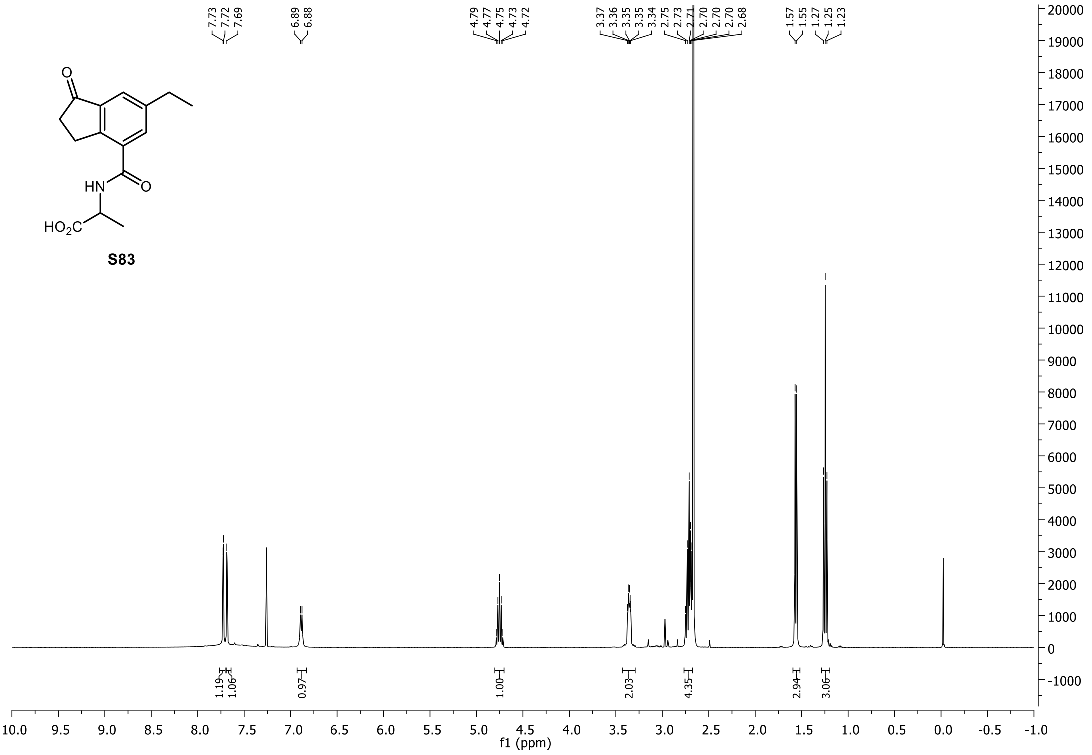

Supplementary Figure 231: ^{13}C NMR S83.

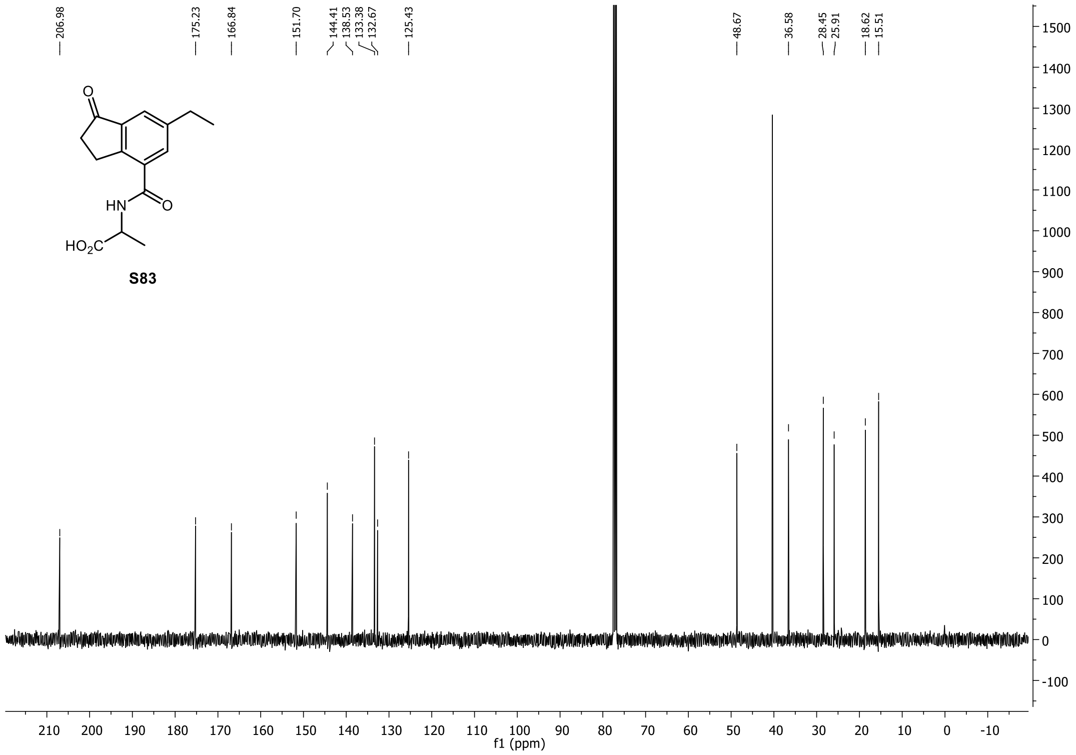

Supplementary Figure 232: ^{1}H NMR S84.

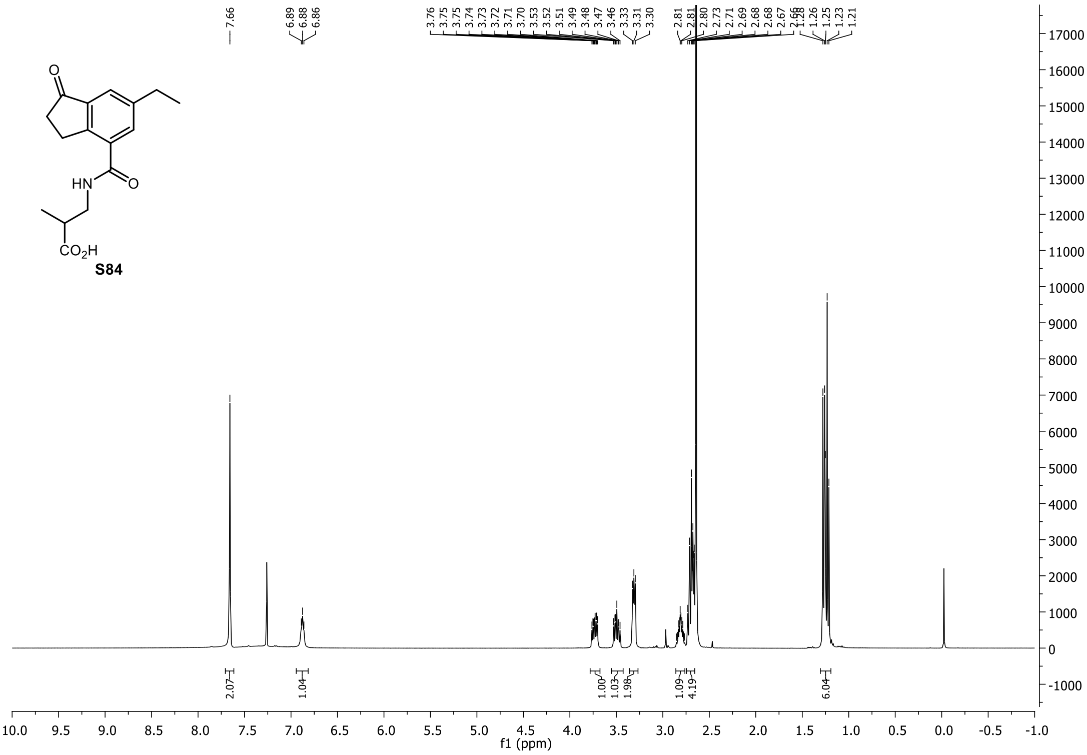

Supplementary Figure 233: ^{3}C NMR S84.

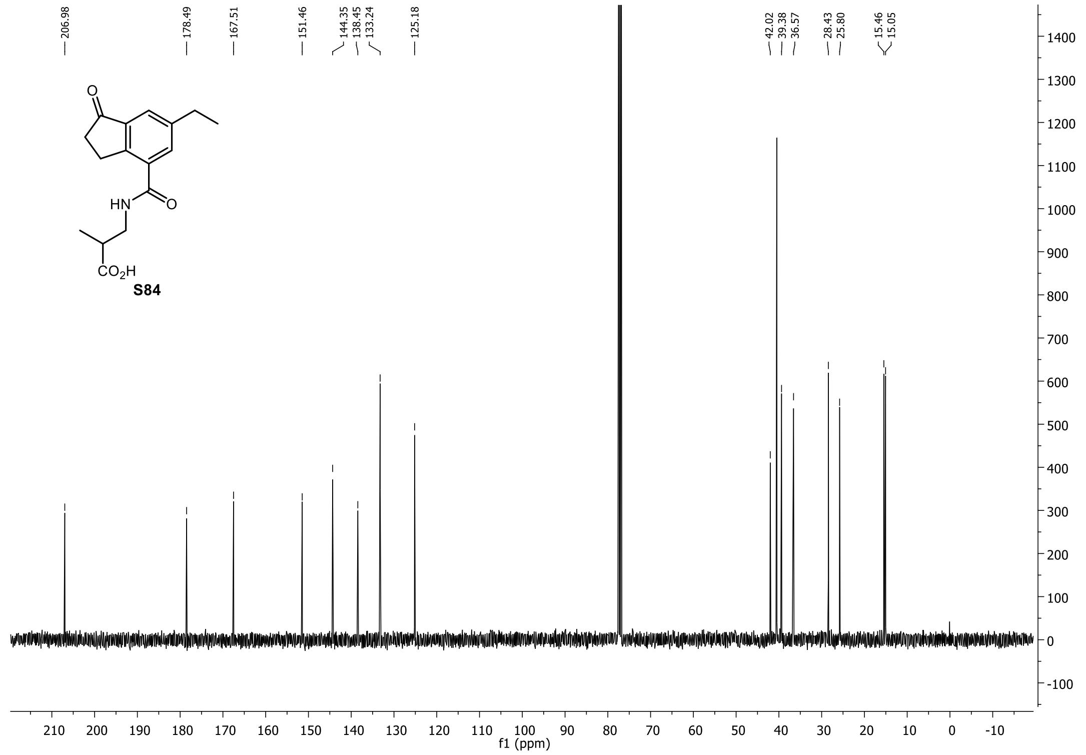

Supplementary Figure 234: ^{1}H NMR S85.

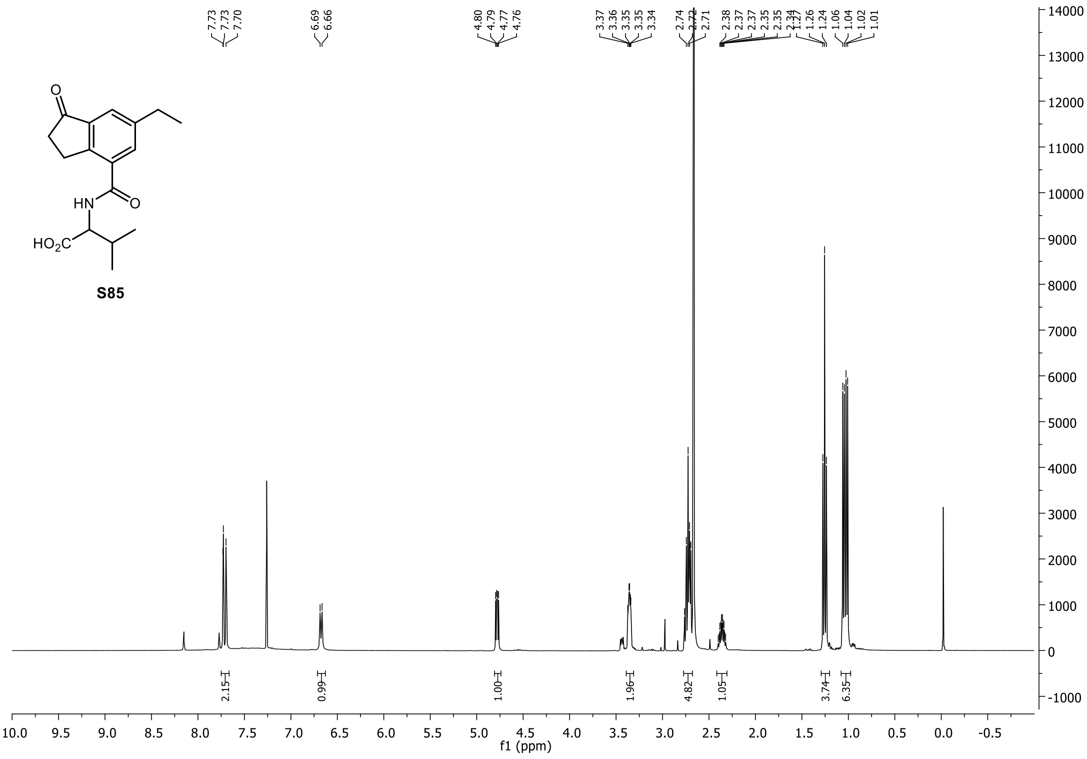

Supplementary Figure 235: ^{13}C NMR S85.

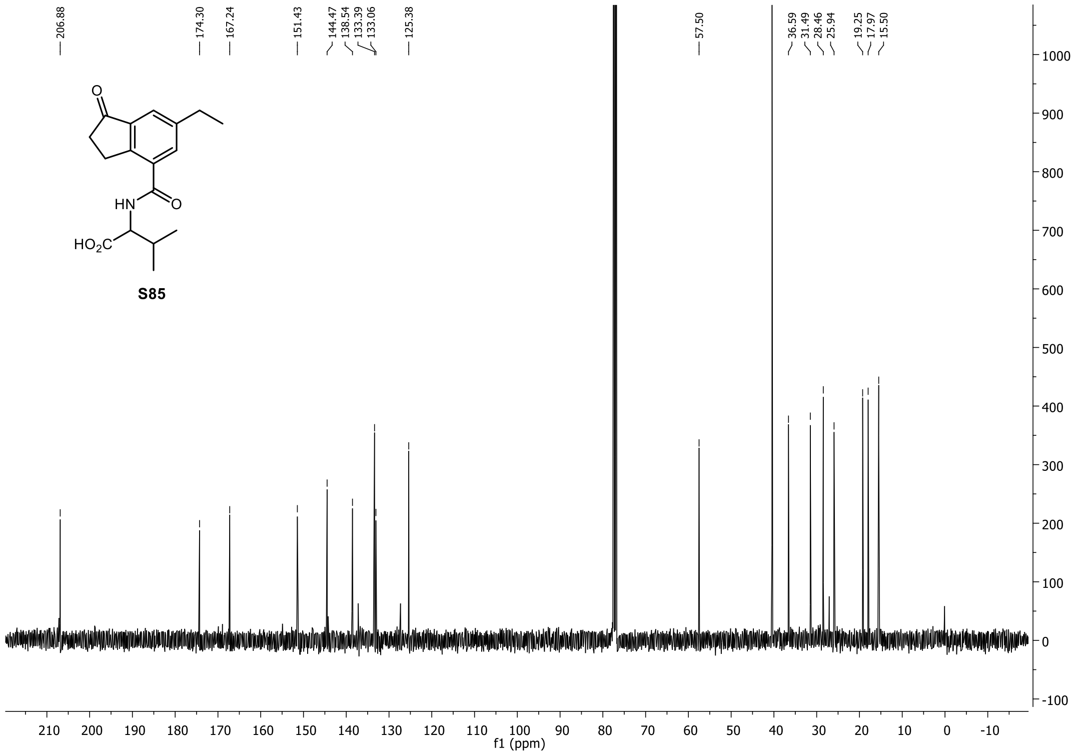

Supplementary Figure 236: ^{1}H NMR 45b.

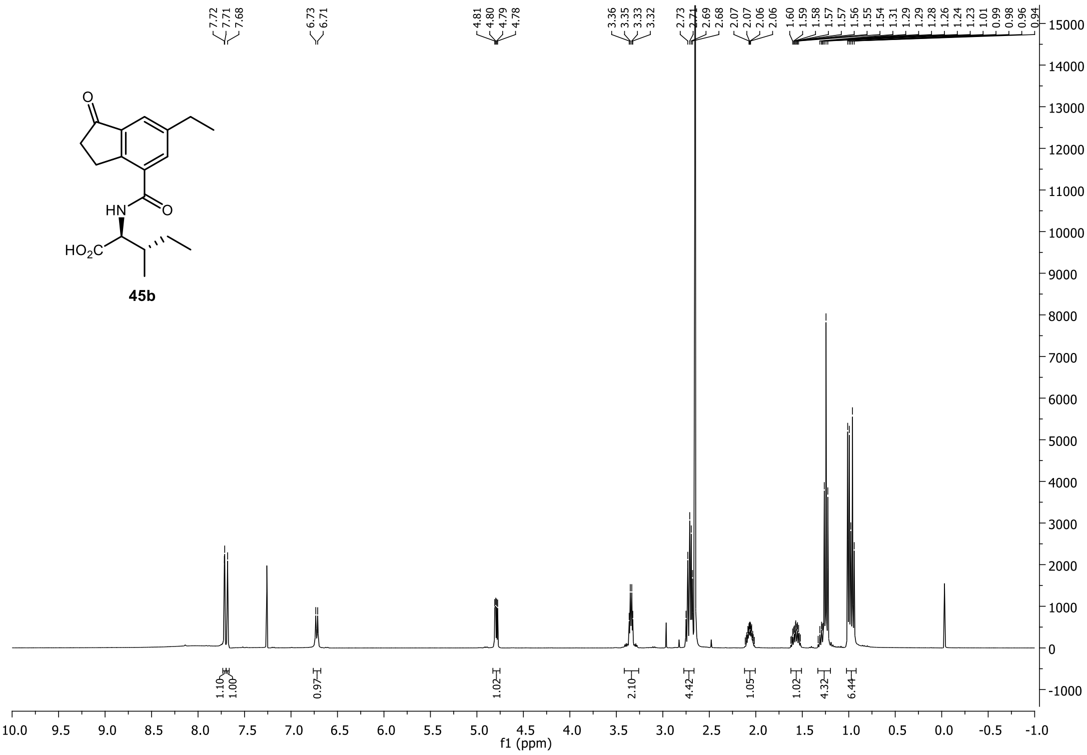

Supplementary Figure 237: ^{13}C NMR 45b.

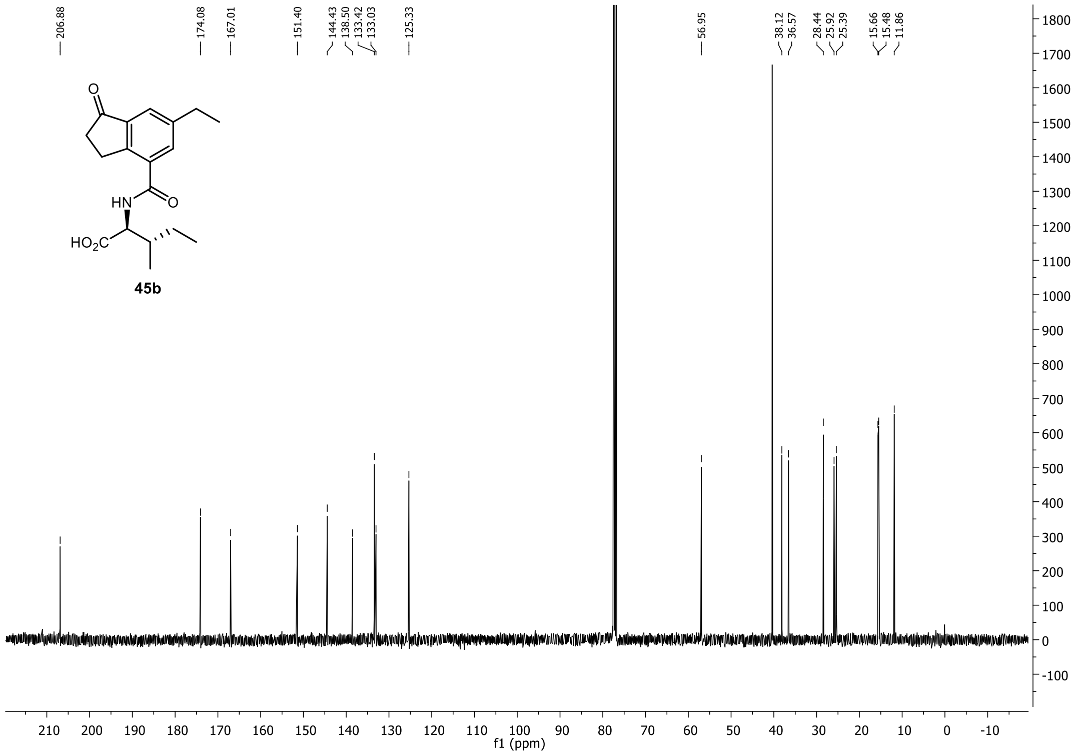

Supplementary Figure 238: ^{1}H NMR S86.

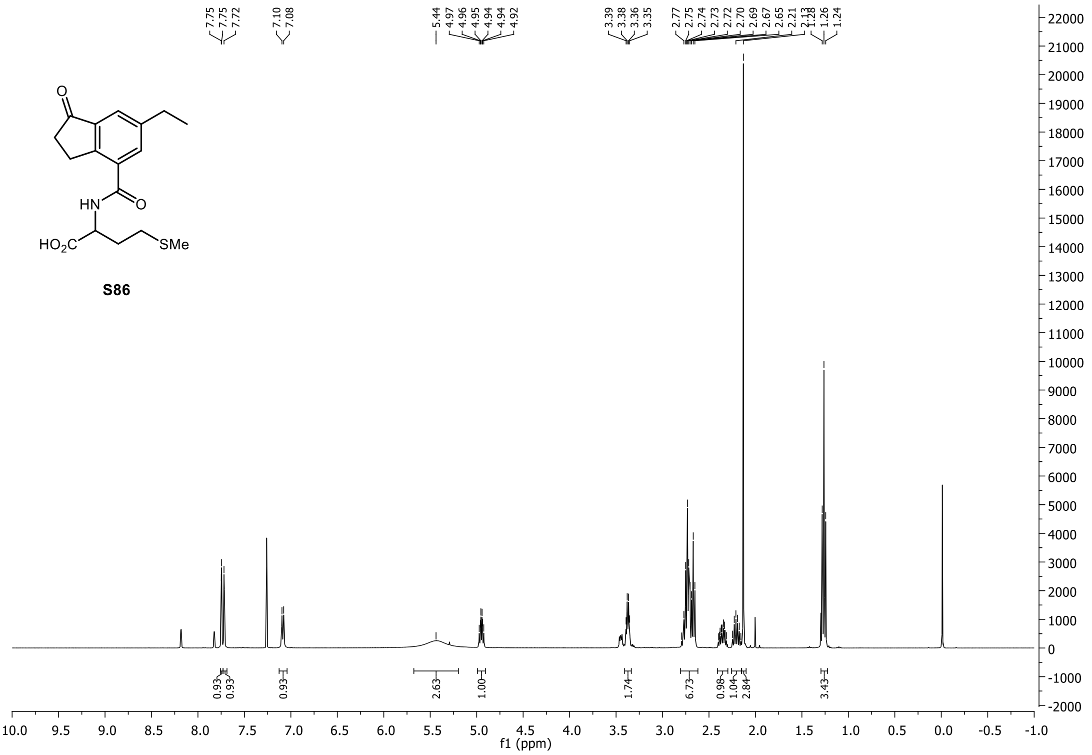

Supplementary Figure 239: ^{13}C NMR S86.

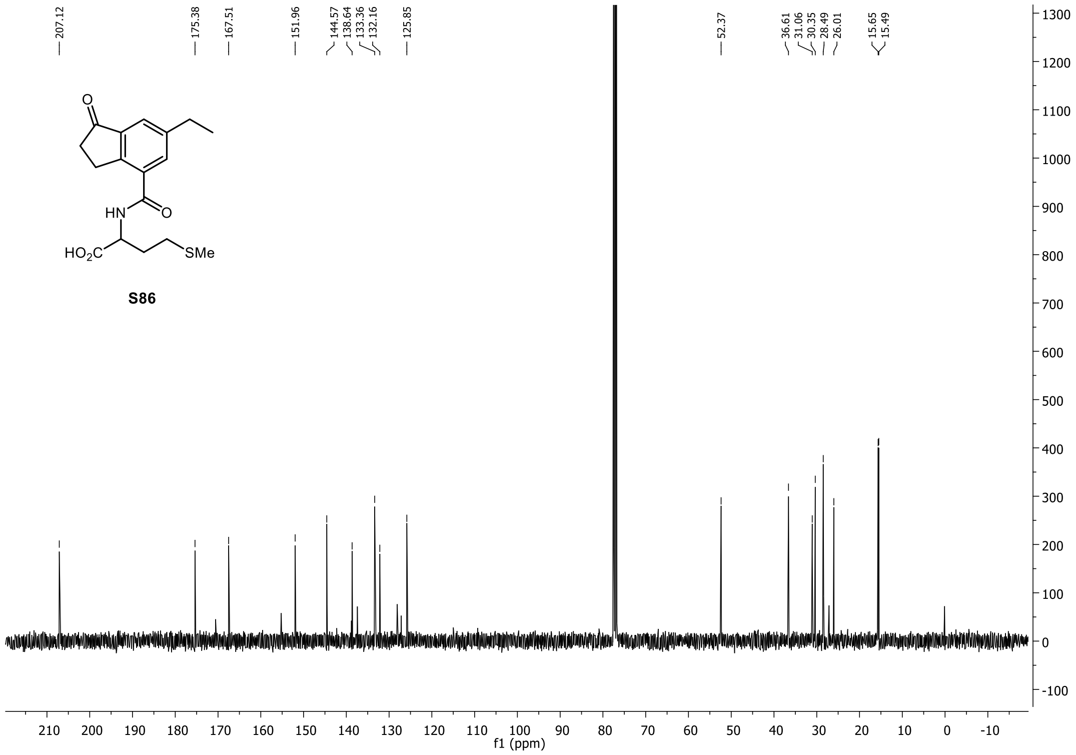

Supplementary Figure 240: ^{1}H NMR S87.

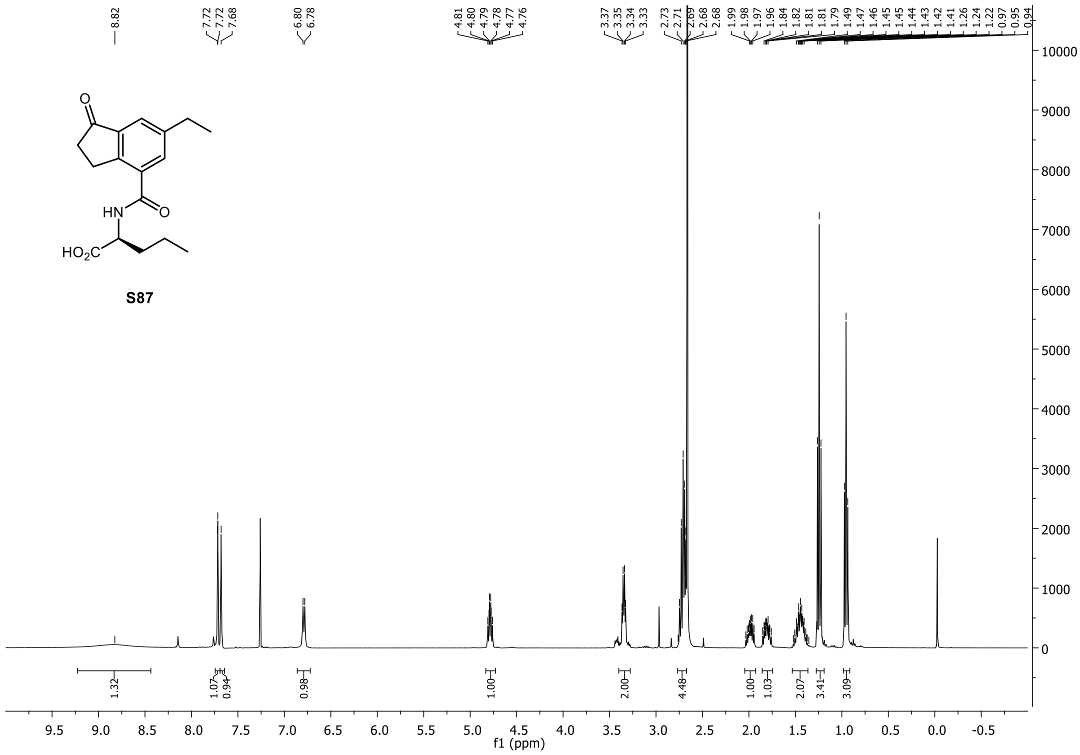

Supplementary Figure 241: ^{13}C NMR S87.

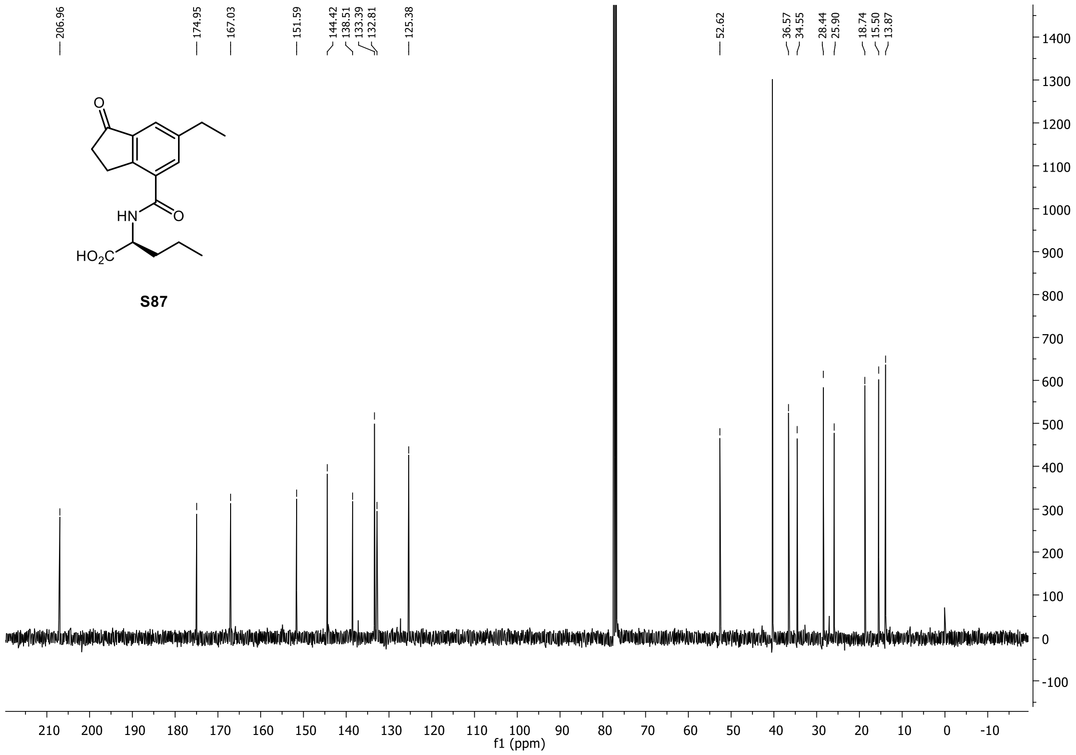

Supplementary Figure 242: ^{1}H NMR S88.

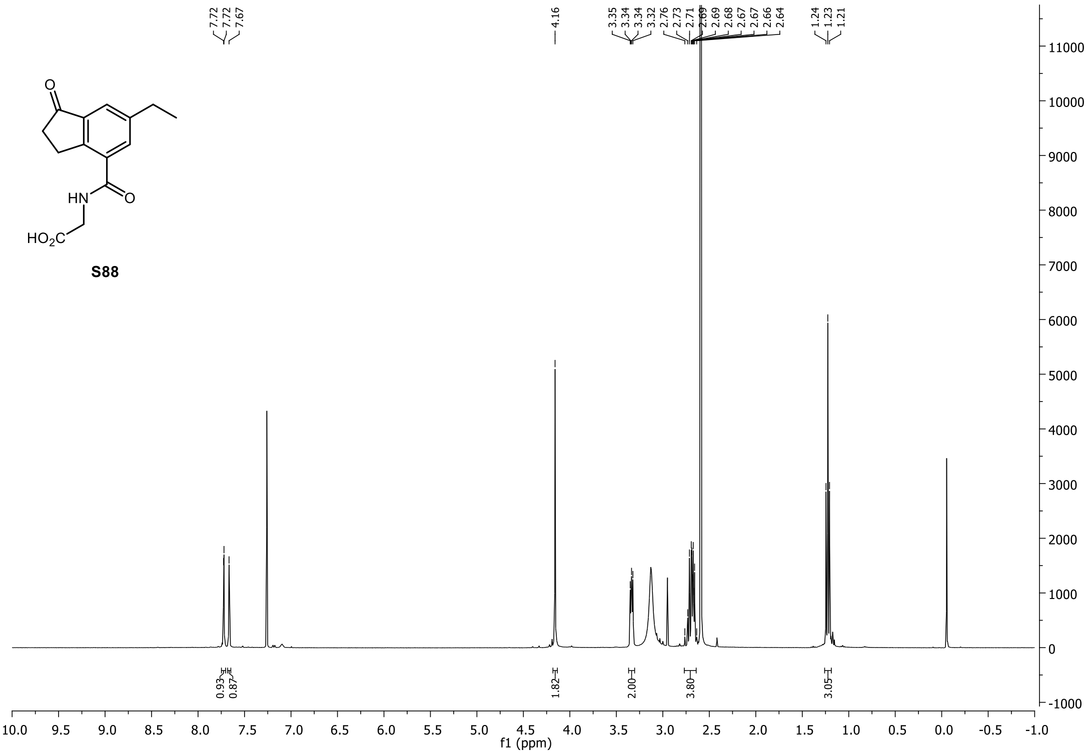

Supplementary Figure 243: ^{13}C NMR S88.

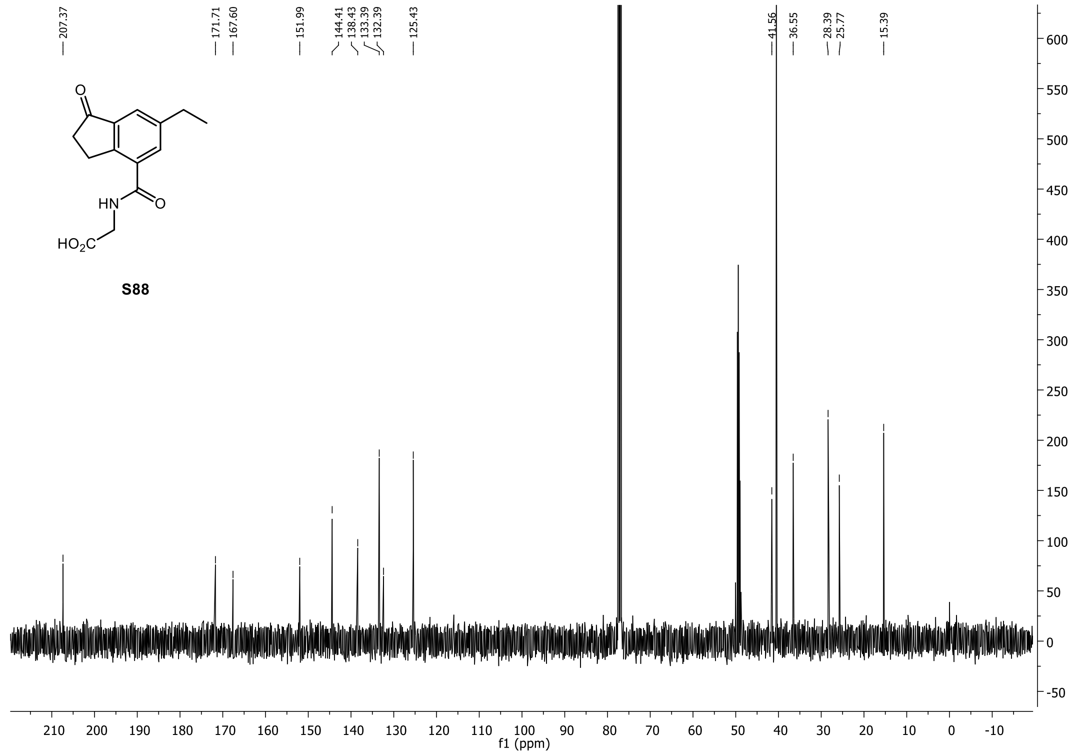

Supplementary Figure 244: ^{1}H NMR S89.

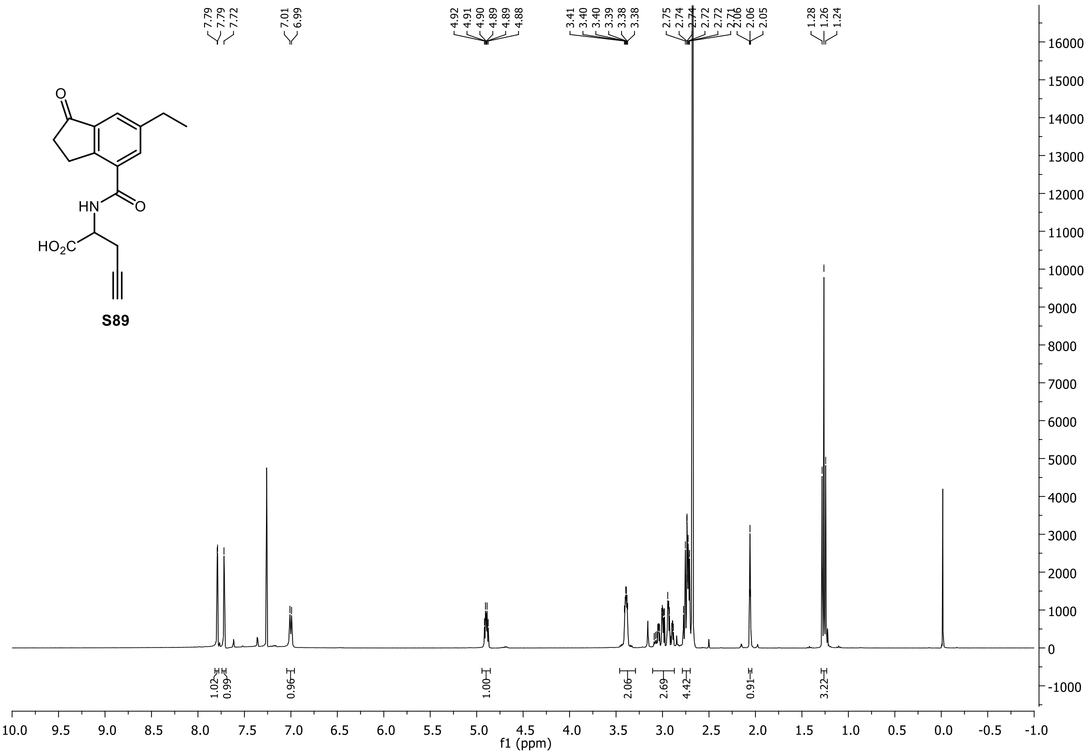

Supplementary Figure 245: ^{13}C NMR S89.

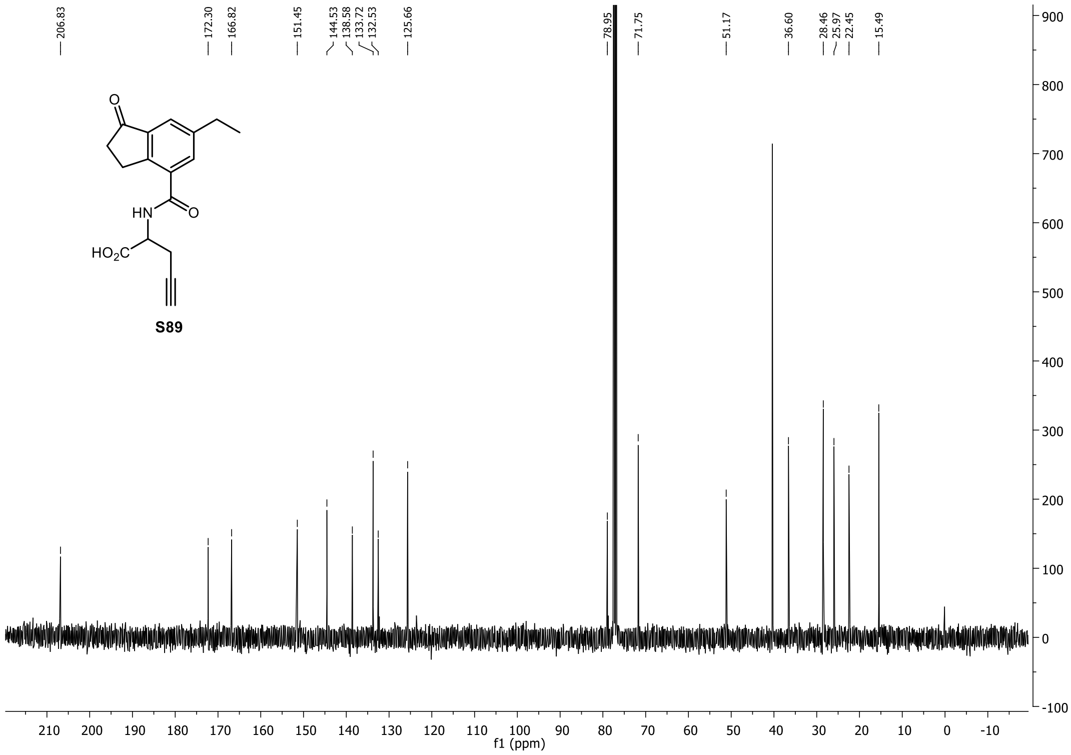

Supplementary Figure 246: ^{1}H NMR S90.

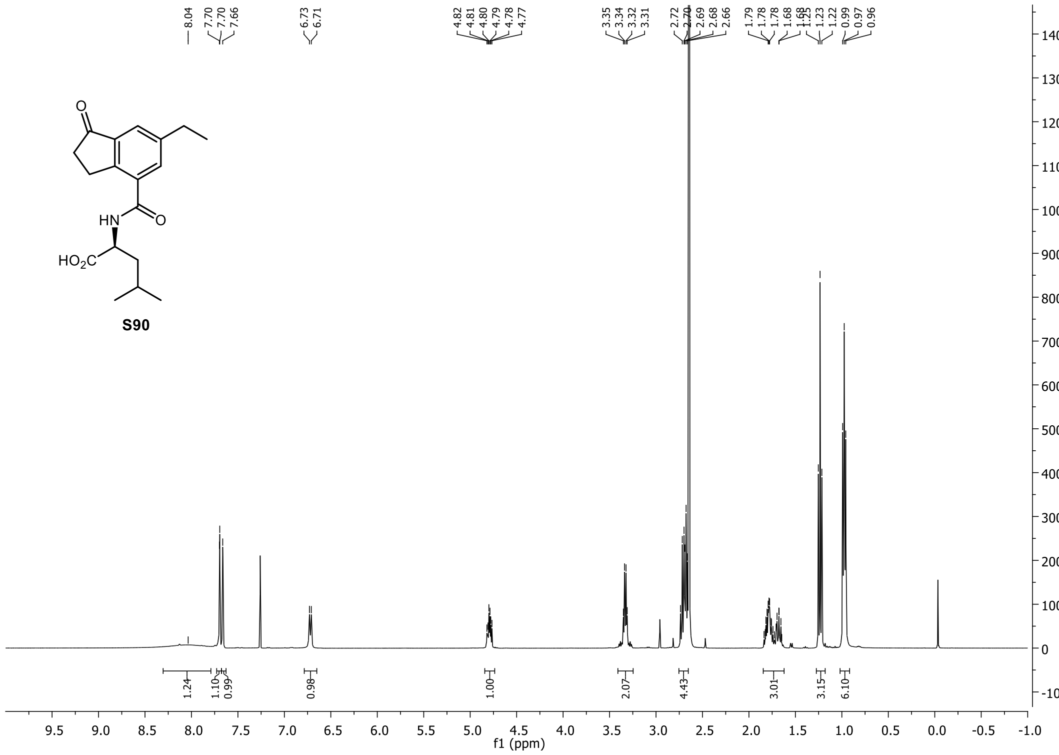

Supplementary Figure 247: ^{13}C NMR S90.

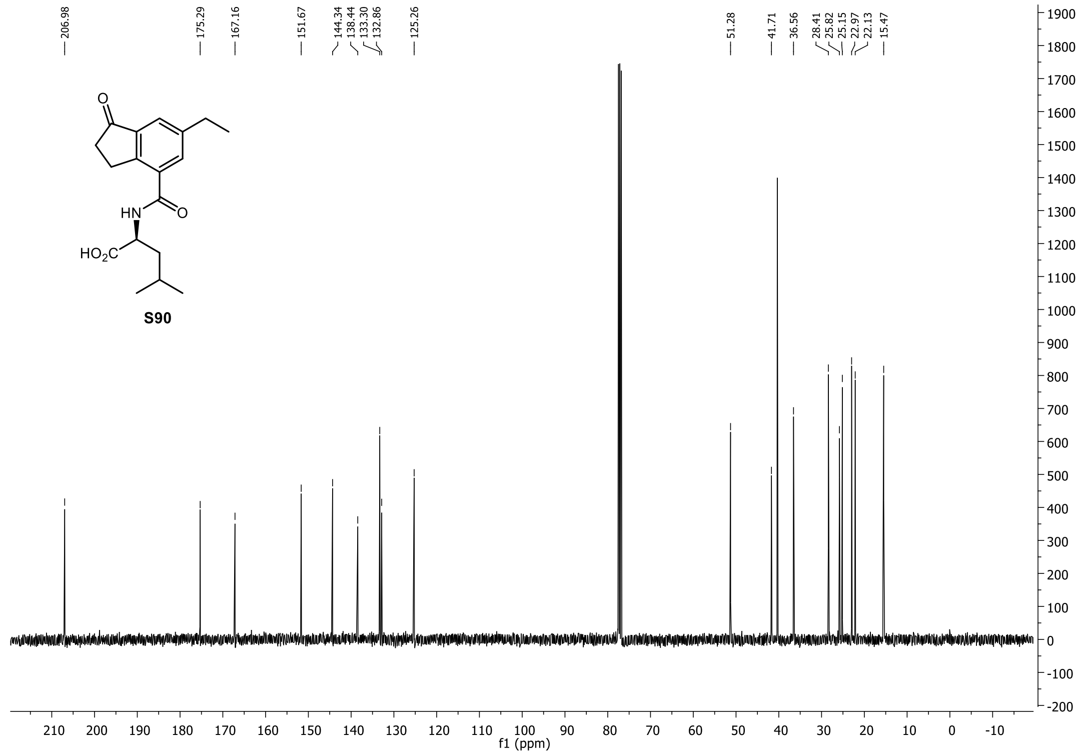

Supplementary Figure 248: ^{1}H NMR S91.

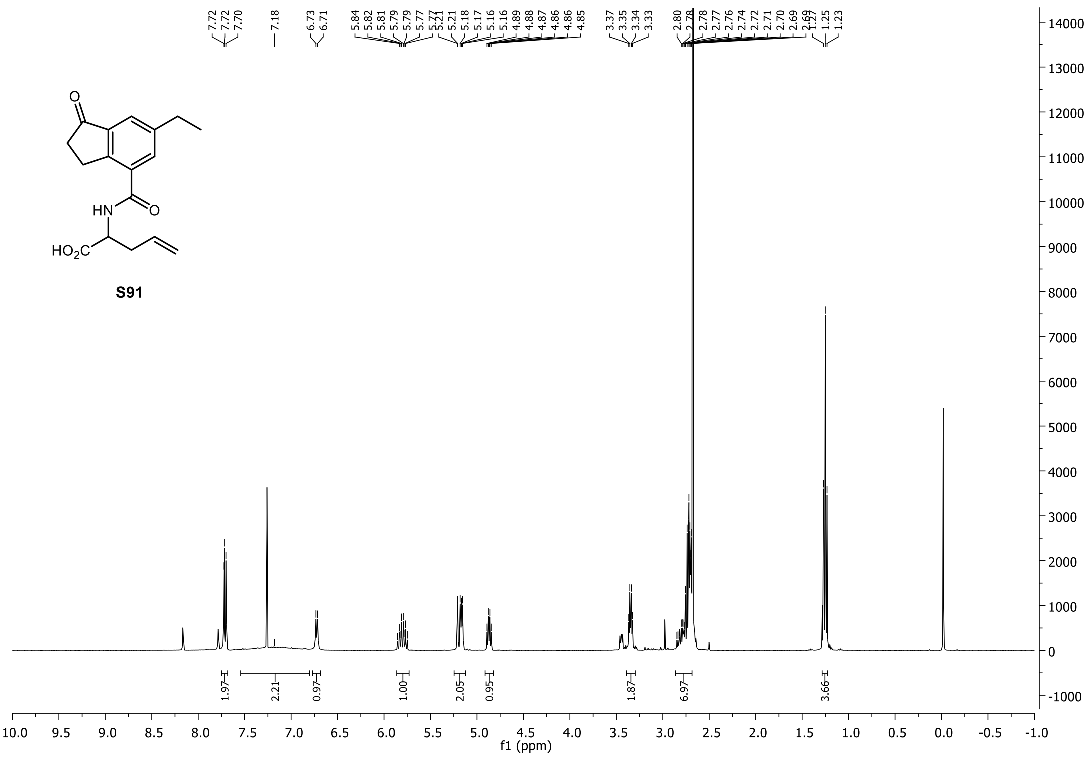

Supplementary Figure 249: ^{13}C NMR S91.

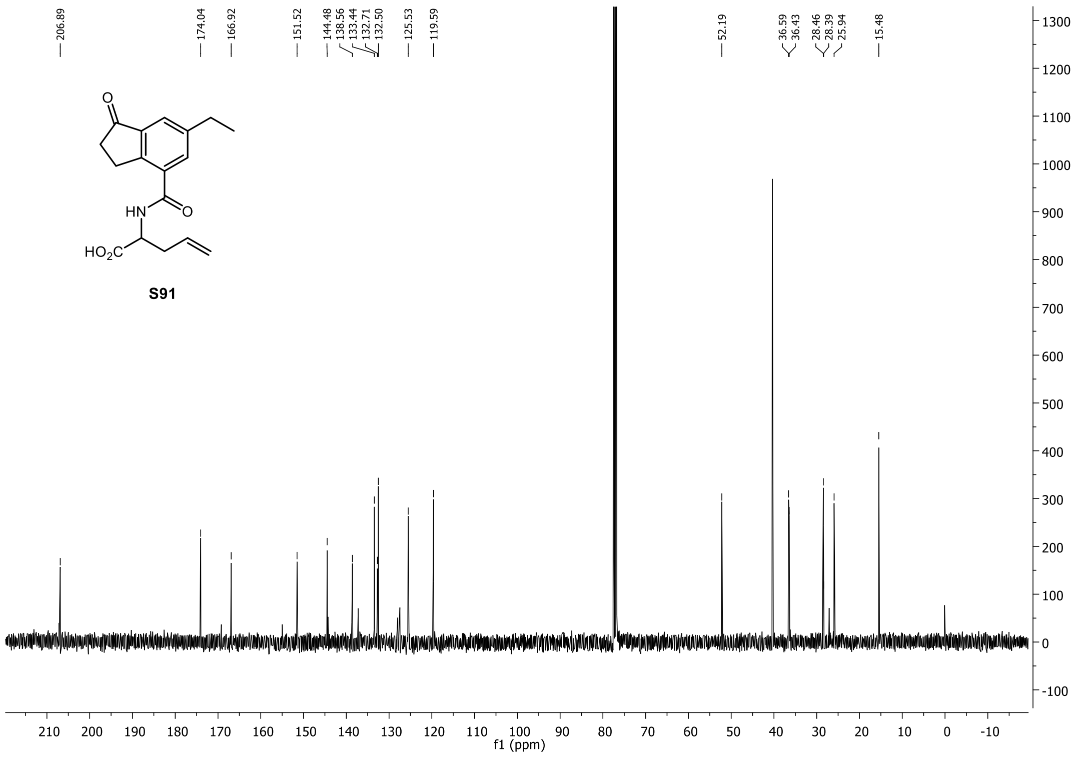

Supplementary Figure 250: ^{1}H NMR S92.

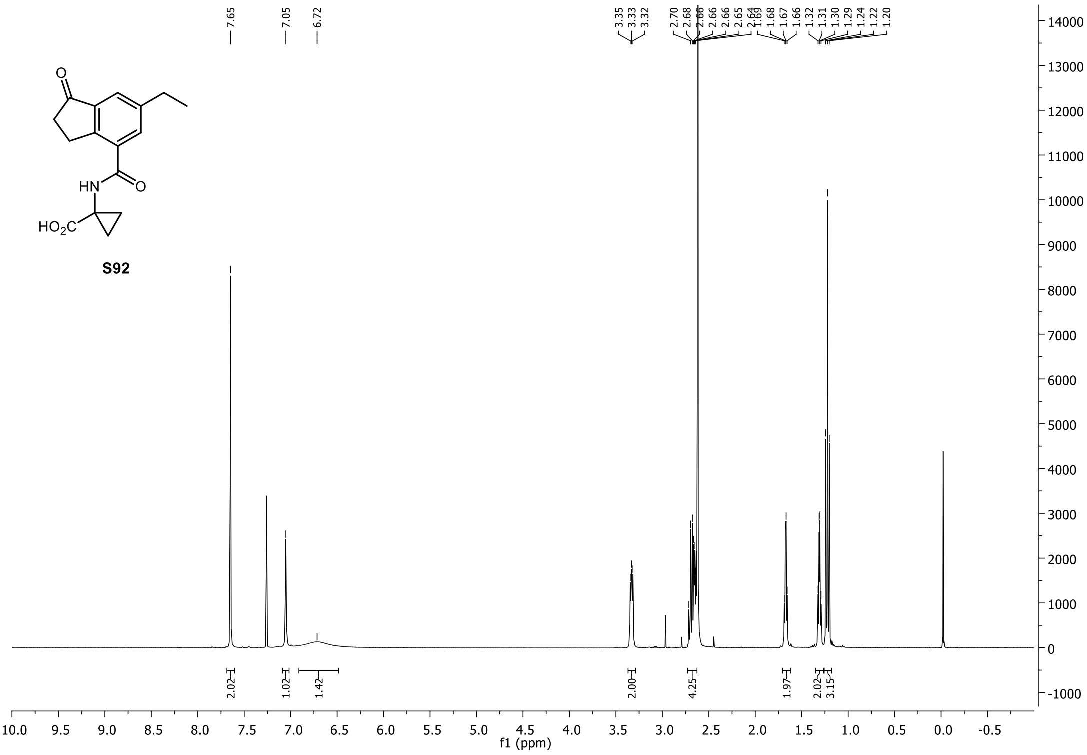

Supplementary Figure 251: ^{13}C NMR S92.

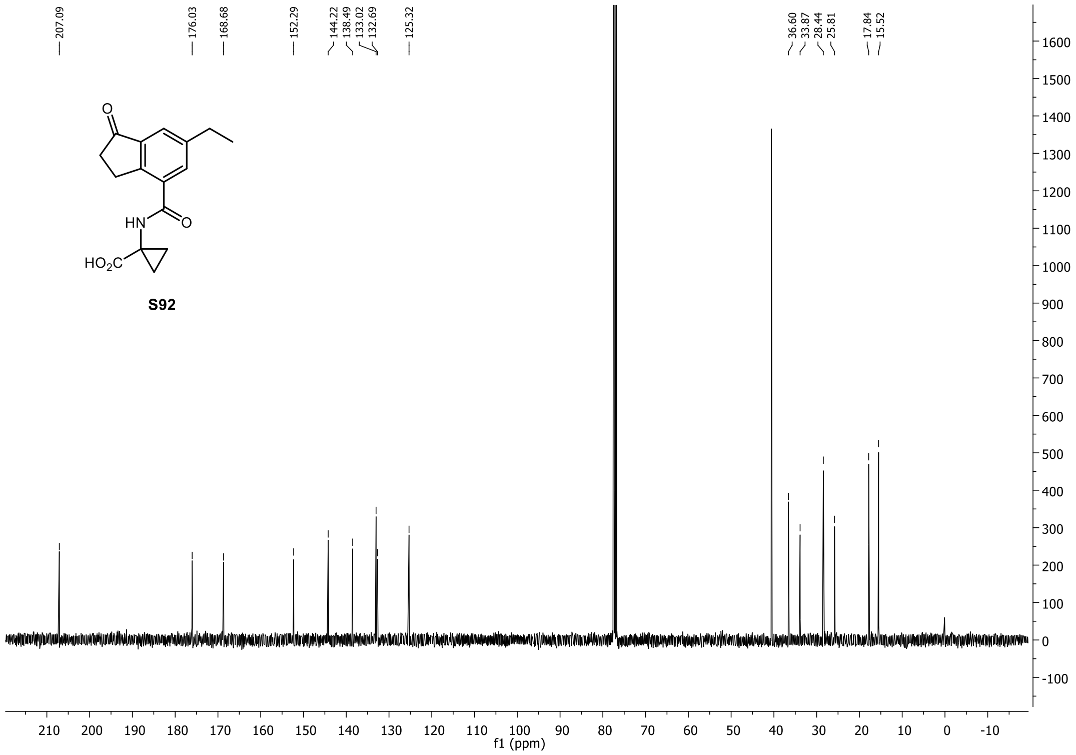

Supplementary Figure 252: ^{1}H NMR S93.

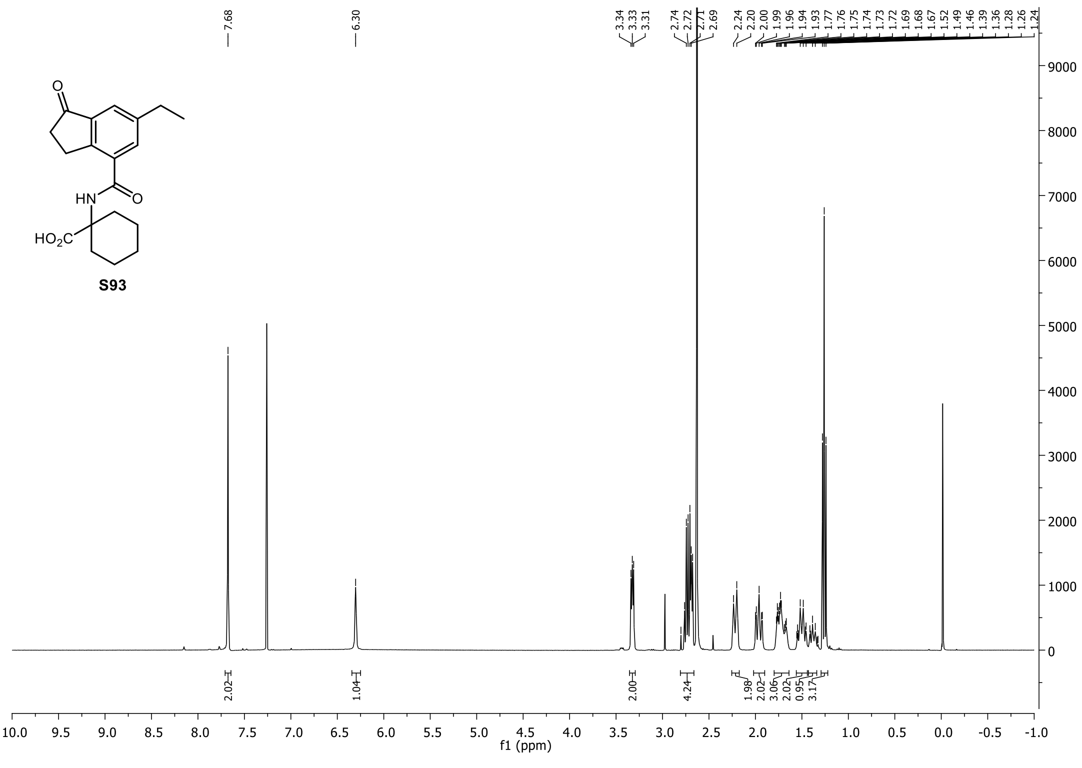

Supplementary Figure 253: ^{13}C NMR S93.

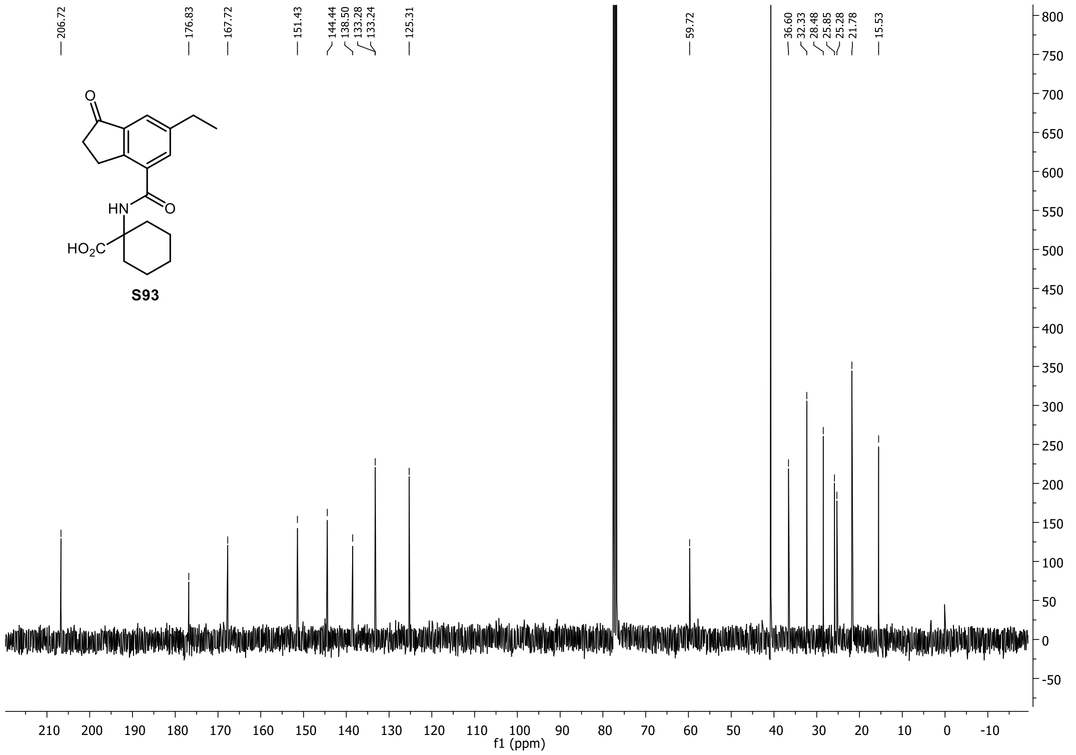

Supplementary Figure 254 ^{1}H NMR S94.

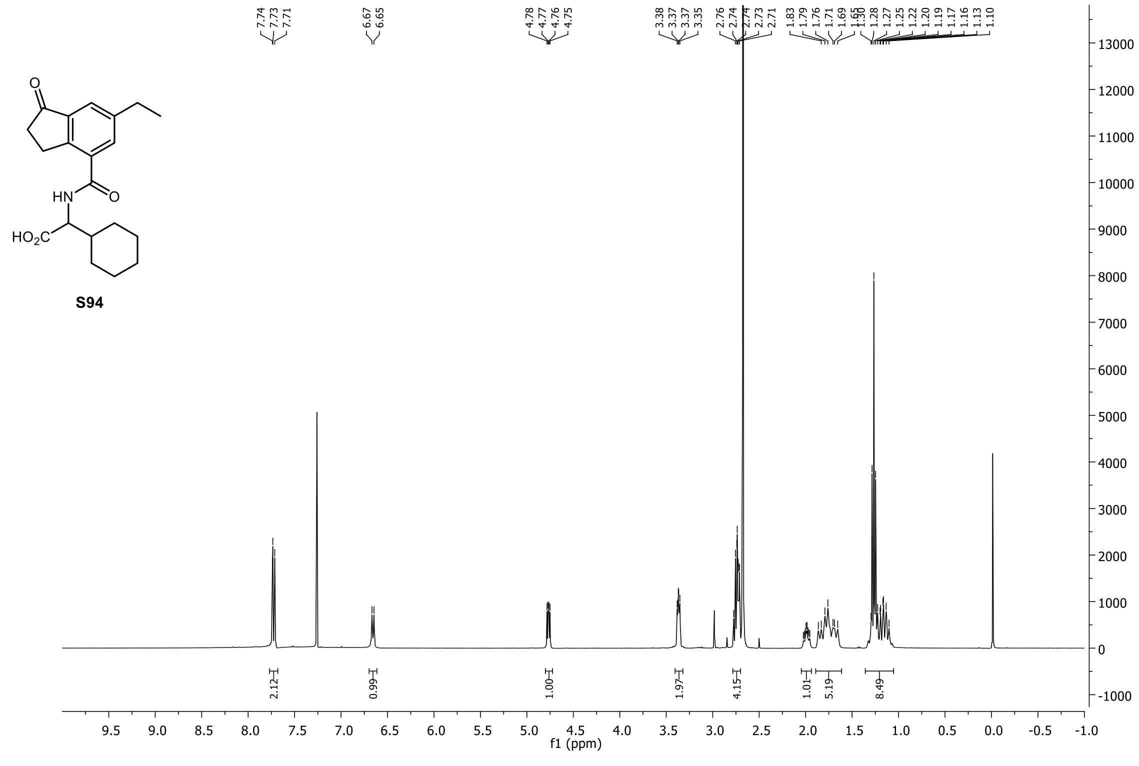

Supplementary Figure 255: ^{13}C NMR S94.

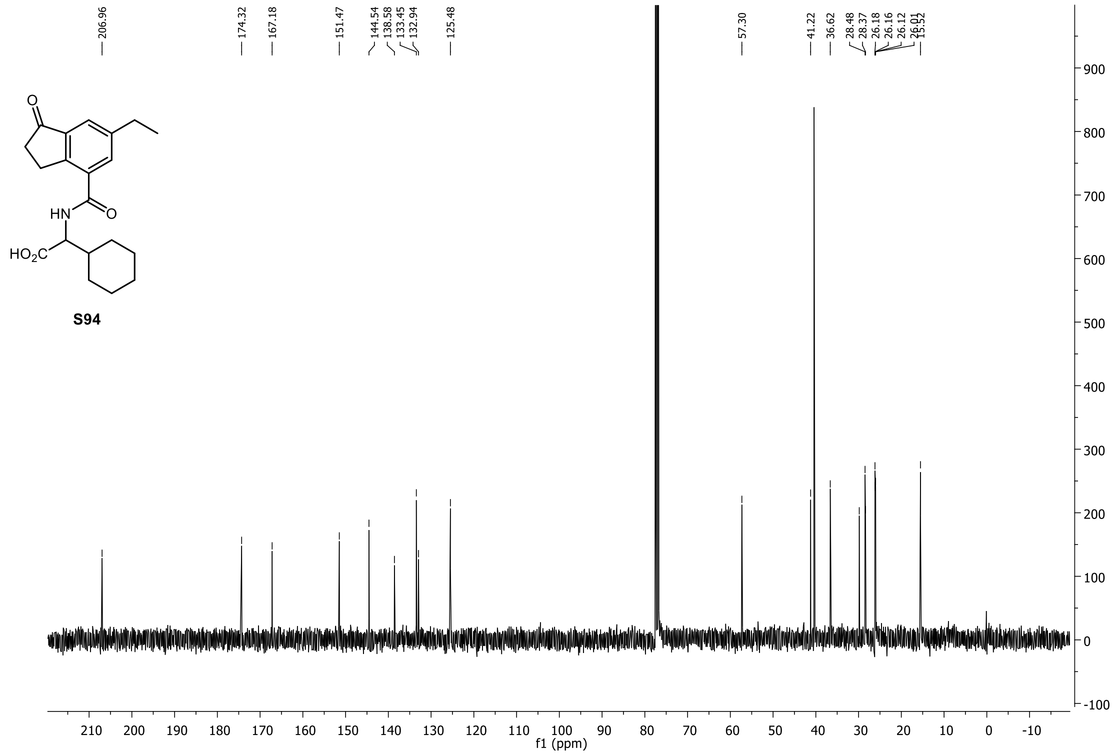

Supplementary Figure 256: ^{1}H NMR S95.

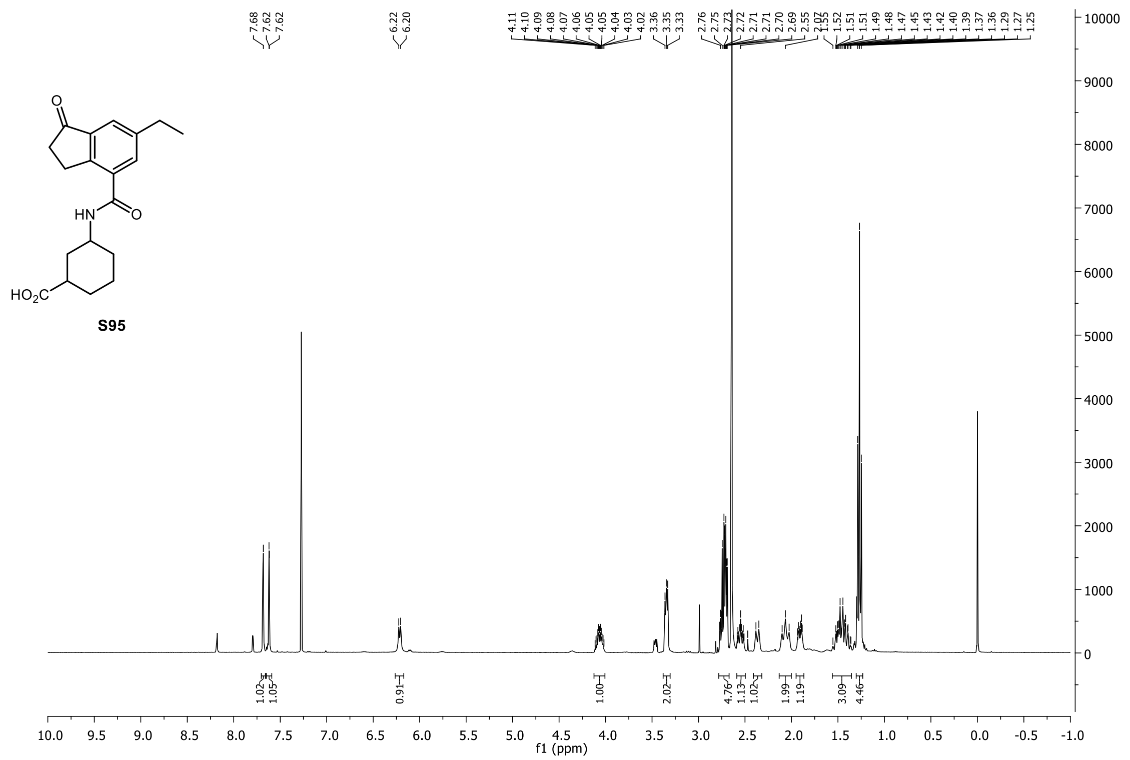

Supplementary Figure 257: ^{13}C NMR S95.

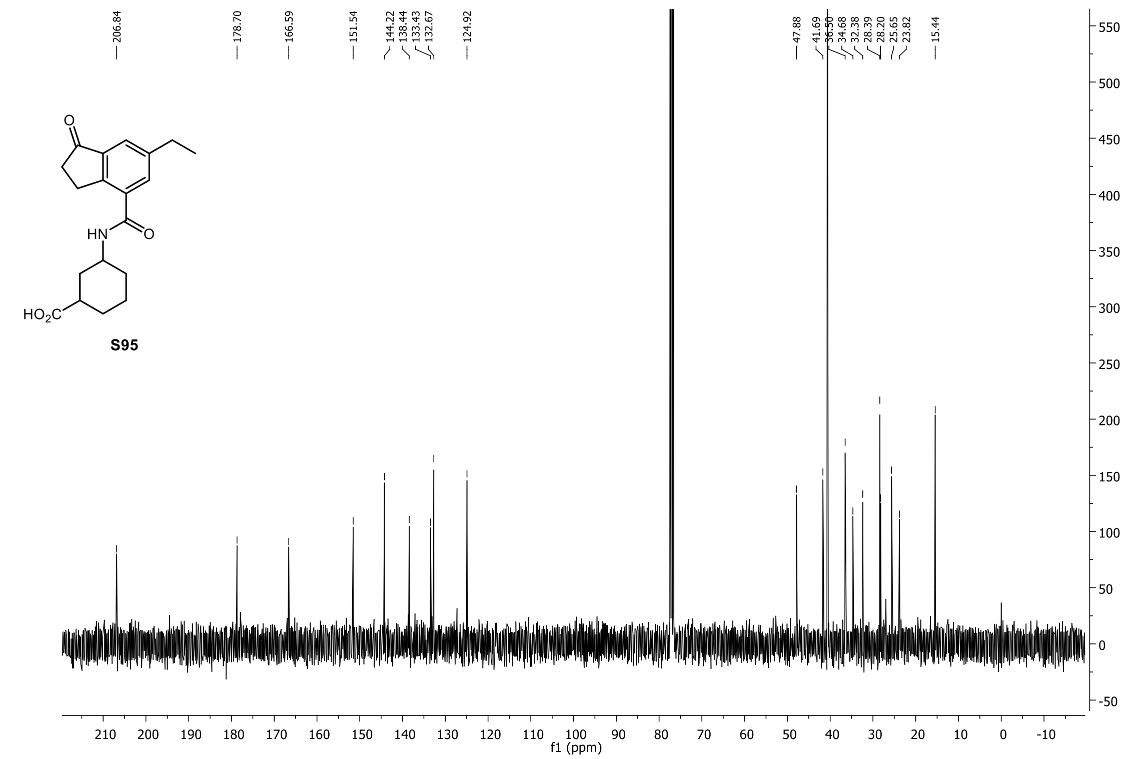

Supplementary Figure 258: ^{1}H NMR S96.

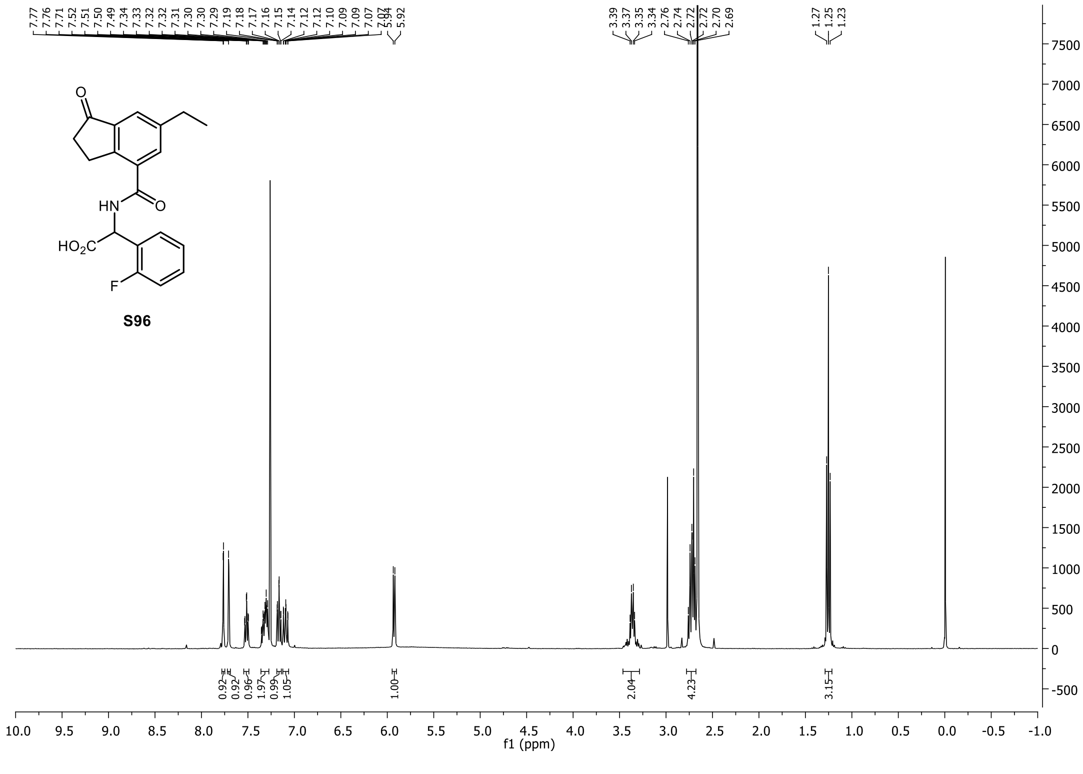

Supplementary Figure 259: ^{13}C NMR S96.

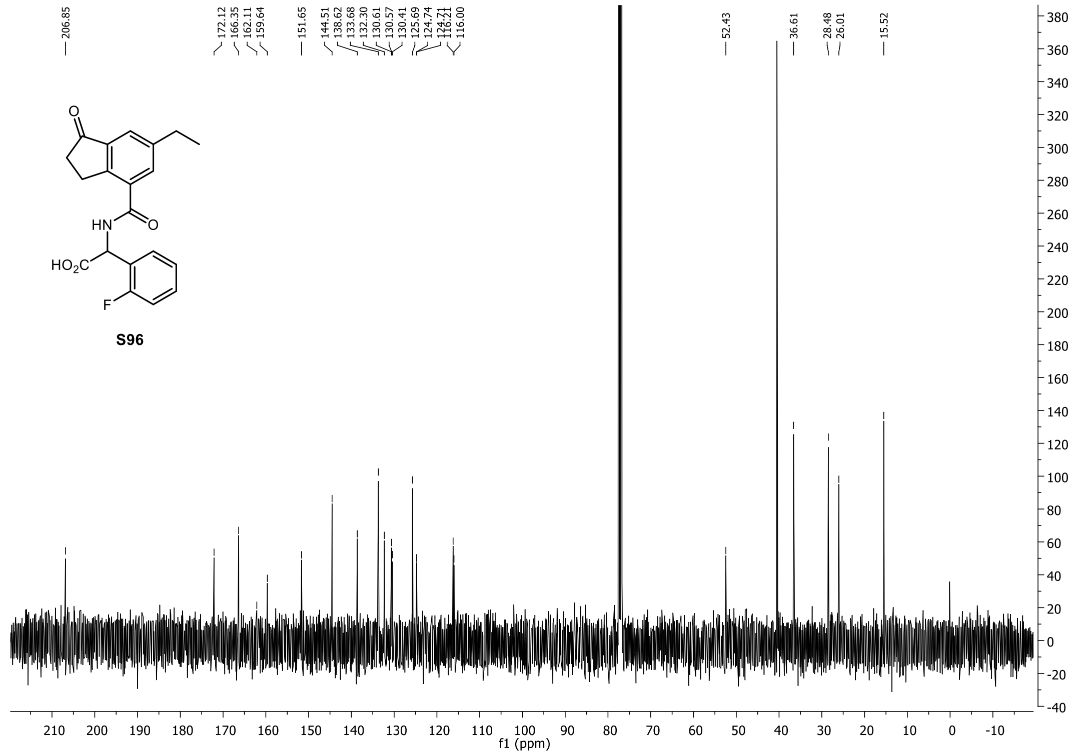

Supplementary Figure 260: ^{1}H NMR S97.

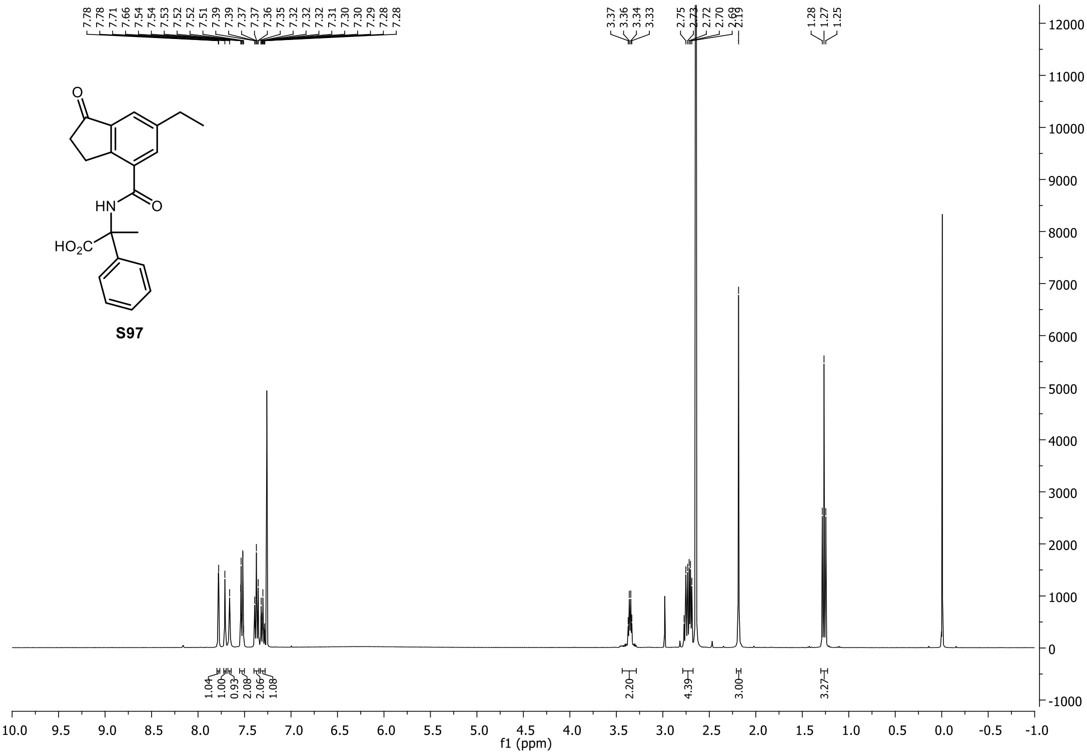

Supplementary Figure 261: ^{13}C NMR S97.

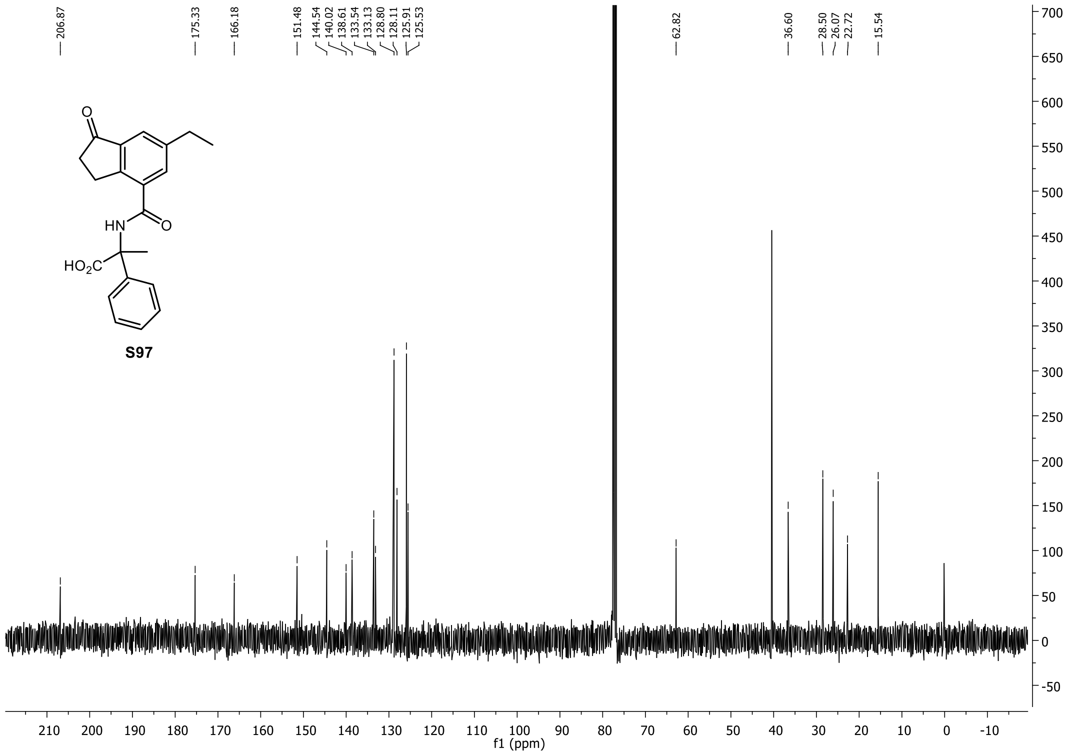

Supplementary Figure 262: ^{1}H NMR S98.

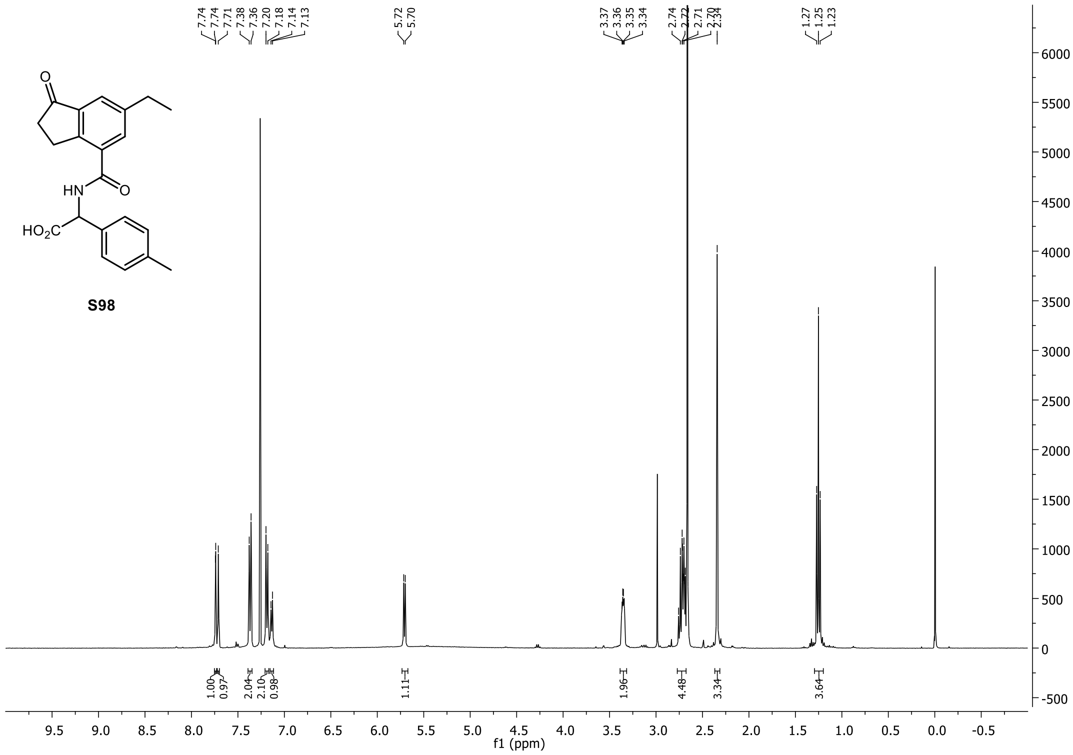

Supplementary Figure 263: ^{13}C NMR S98.

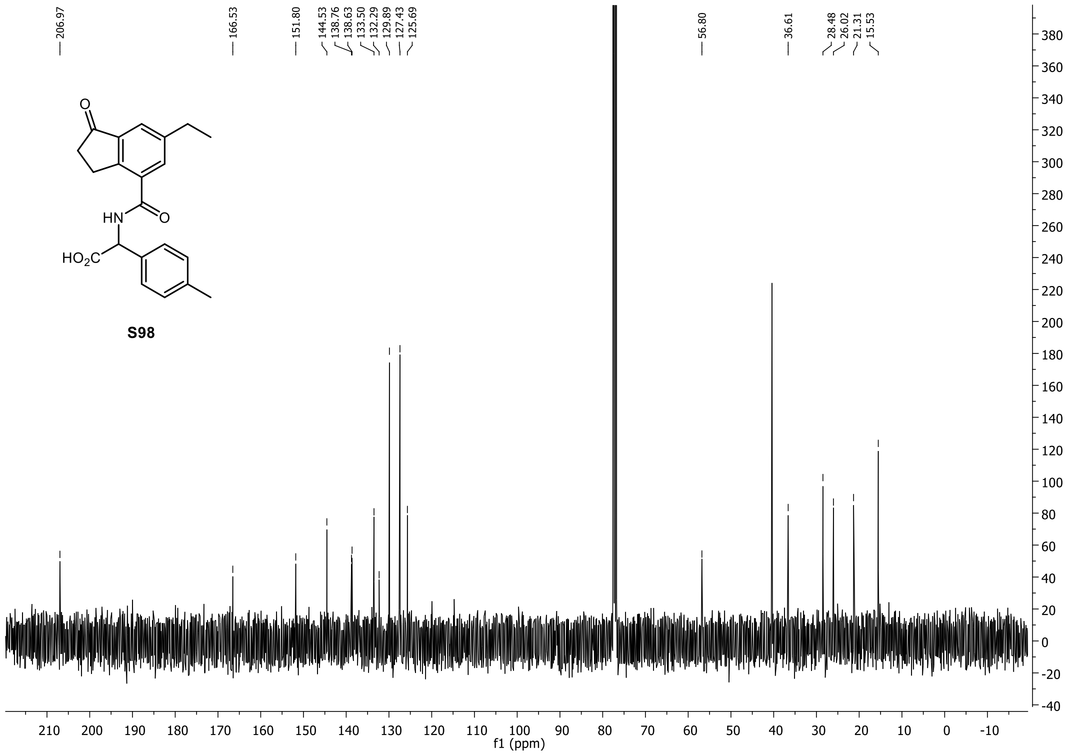

Supplementary Figure 264: ^{1}H NMR S99.

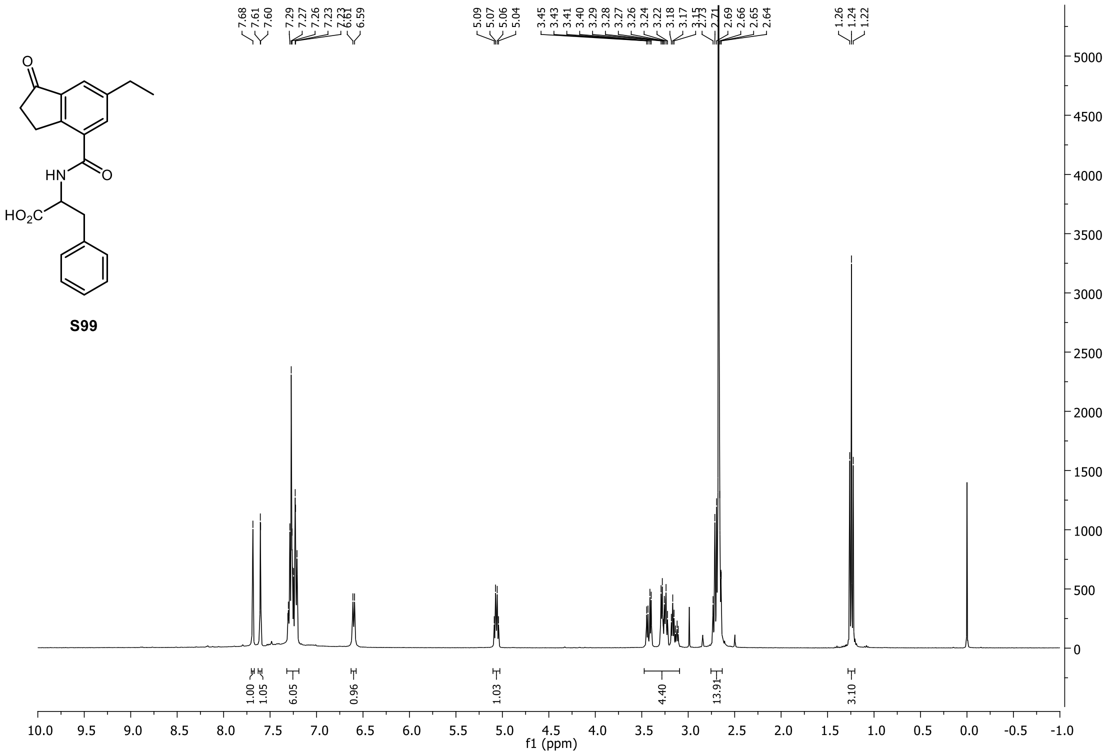

Supplementary Figure 265: ^{13}C NMR S99.

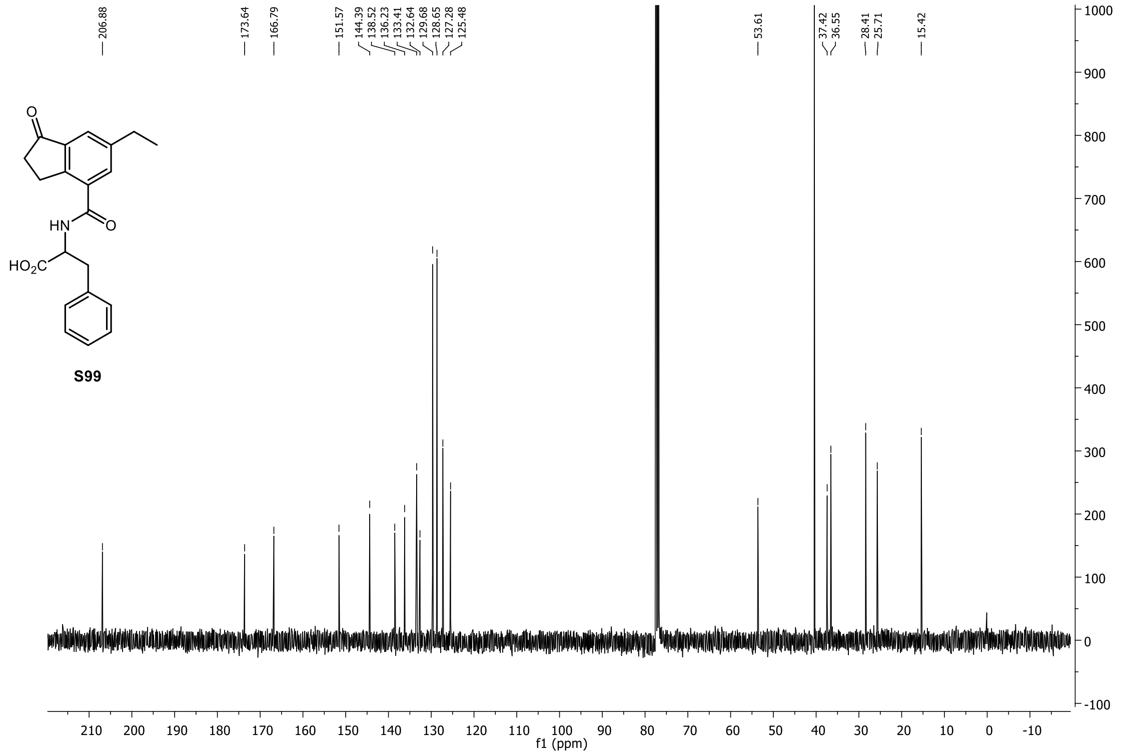

Supplementary Figure 266: ^{1}H NMR S100.

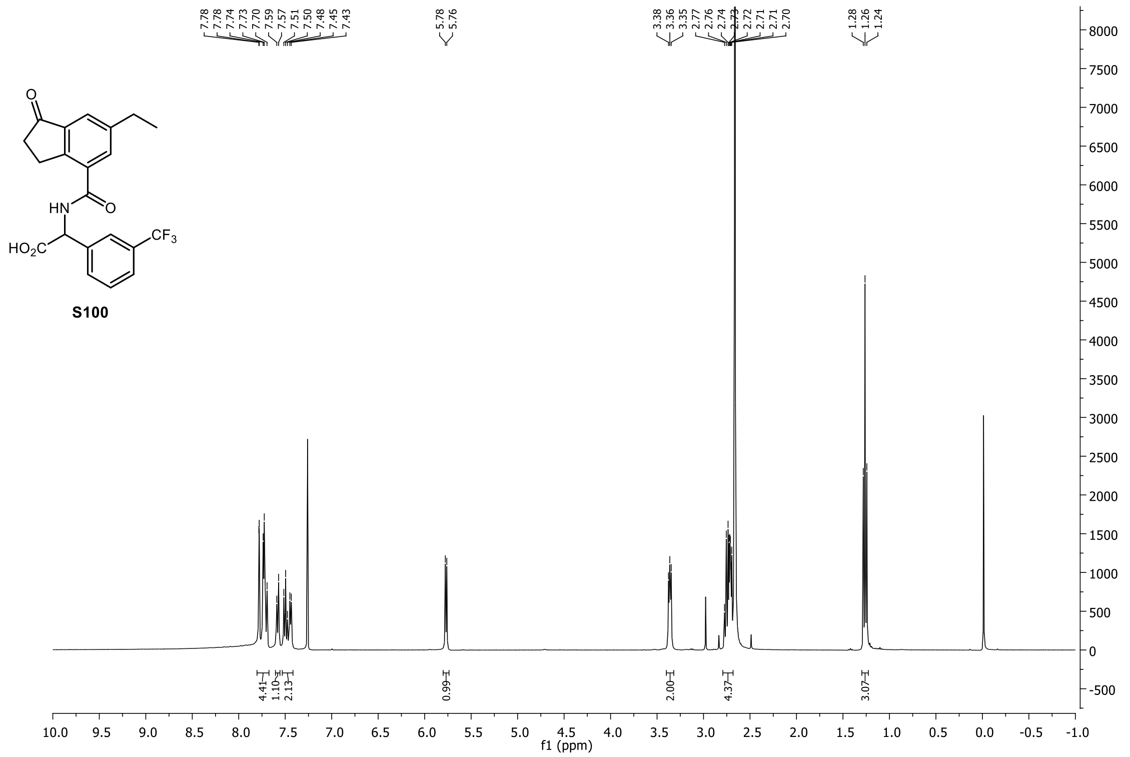

Supplementary Figure 267: ^{13}C NMR S100.

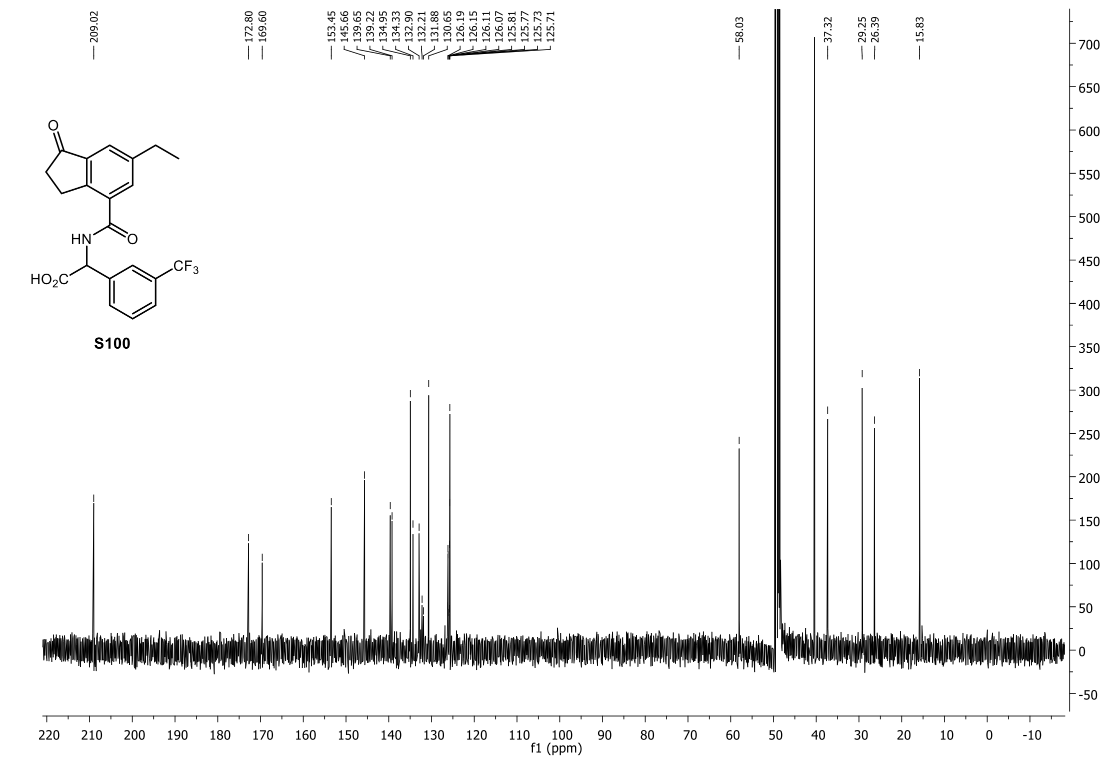

Supplementary Figure 268: ^{1}H NMR S101.

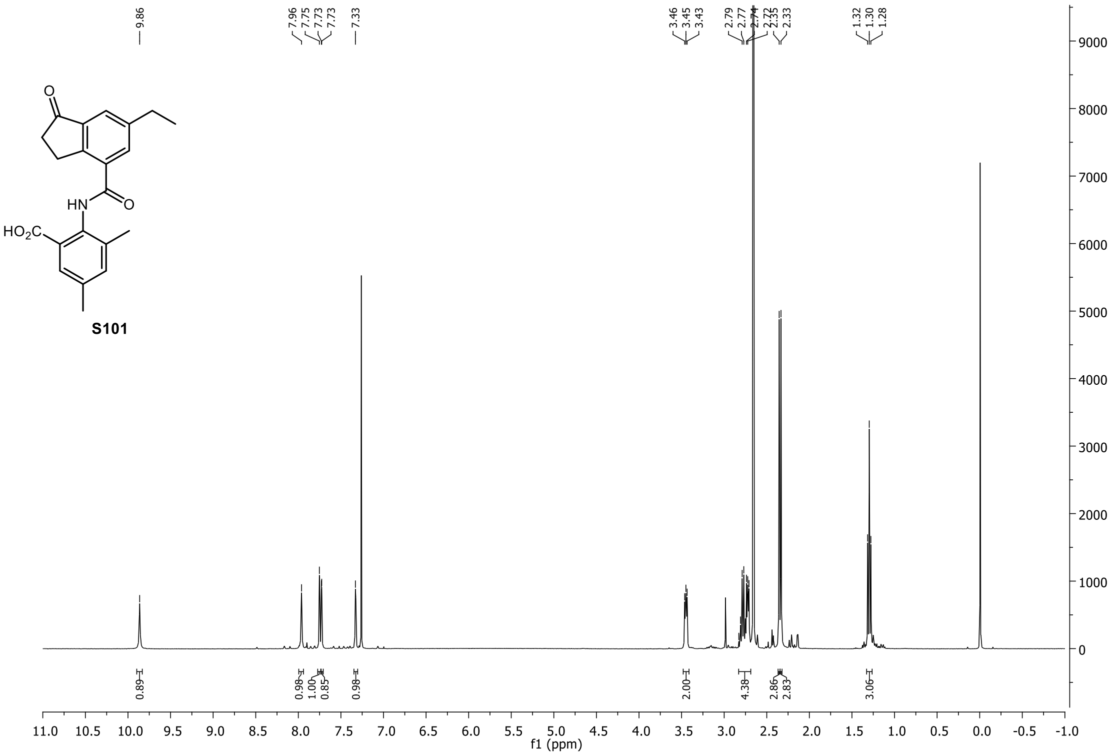

Supplementary Figure 269: ^{13}C NMR S101.

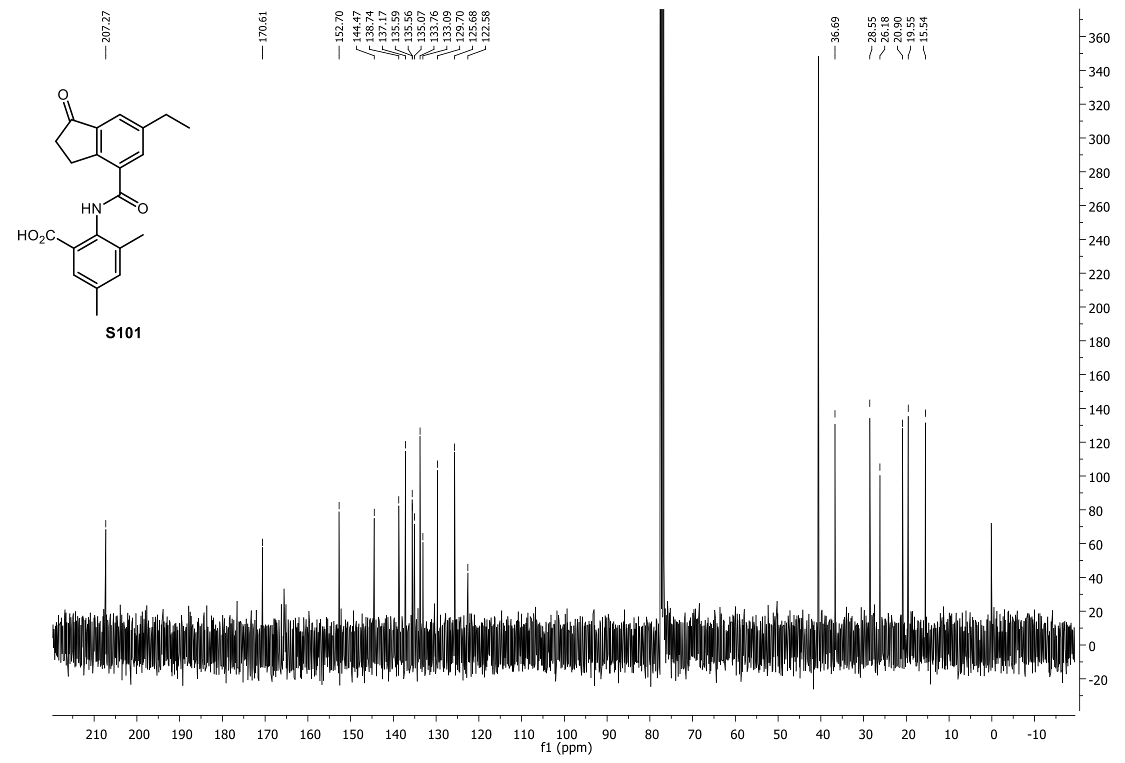

Supplementary Figure 270: ^{1}H NMR S102.

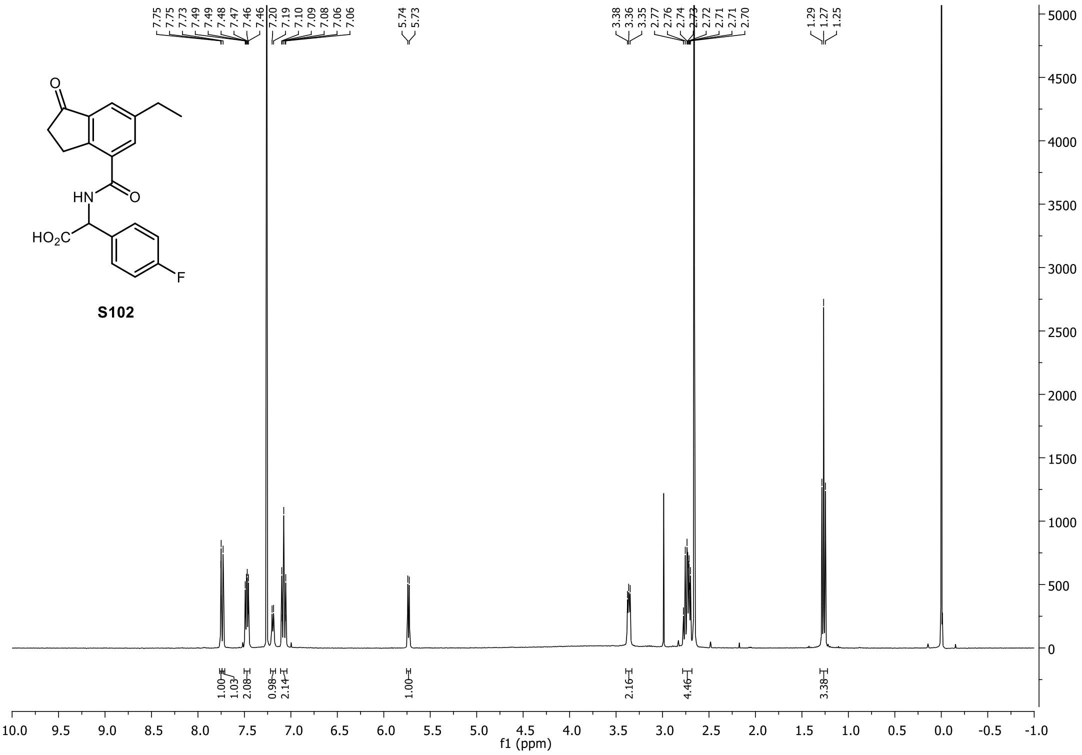

Supplementary Figure 271: ^{13}C NMR S102.

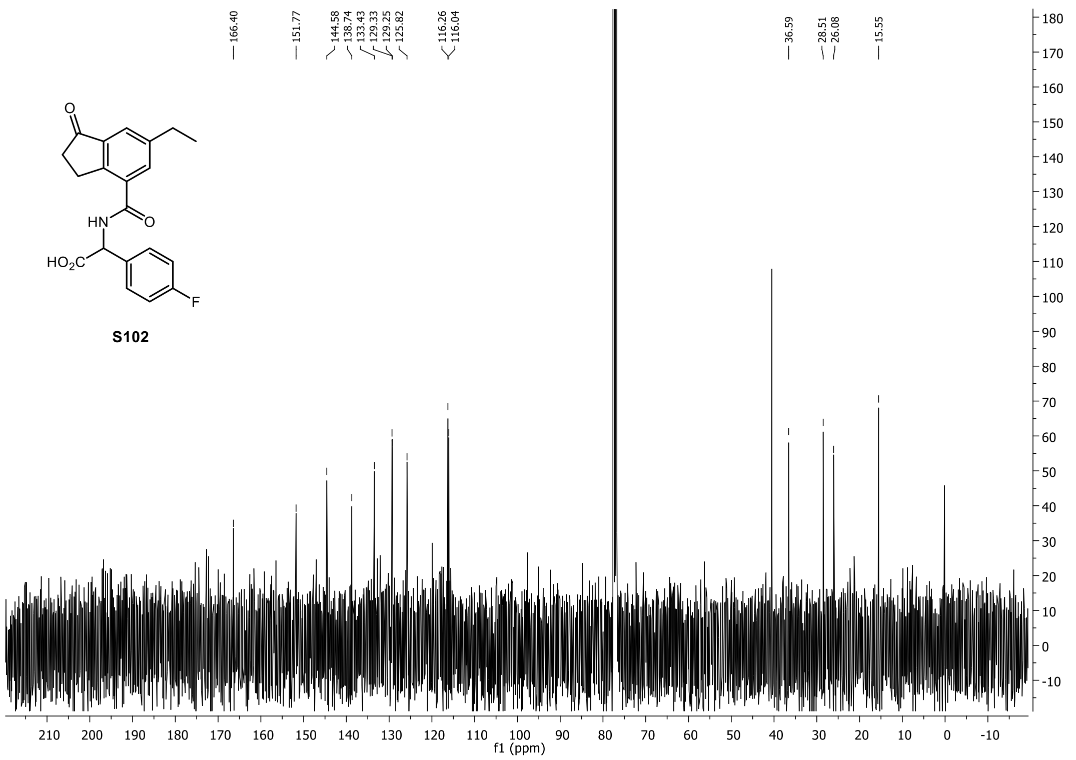

Supplementary Figure 272: ^{1}H NMR S103.

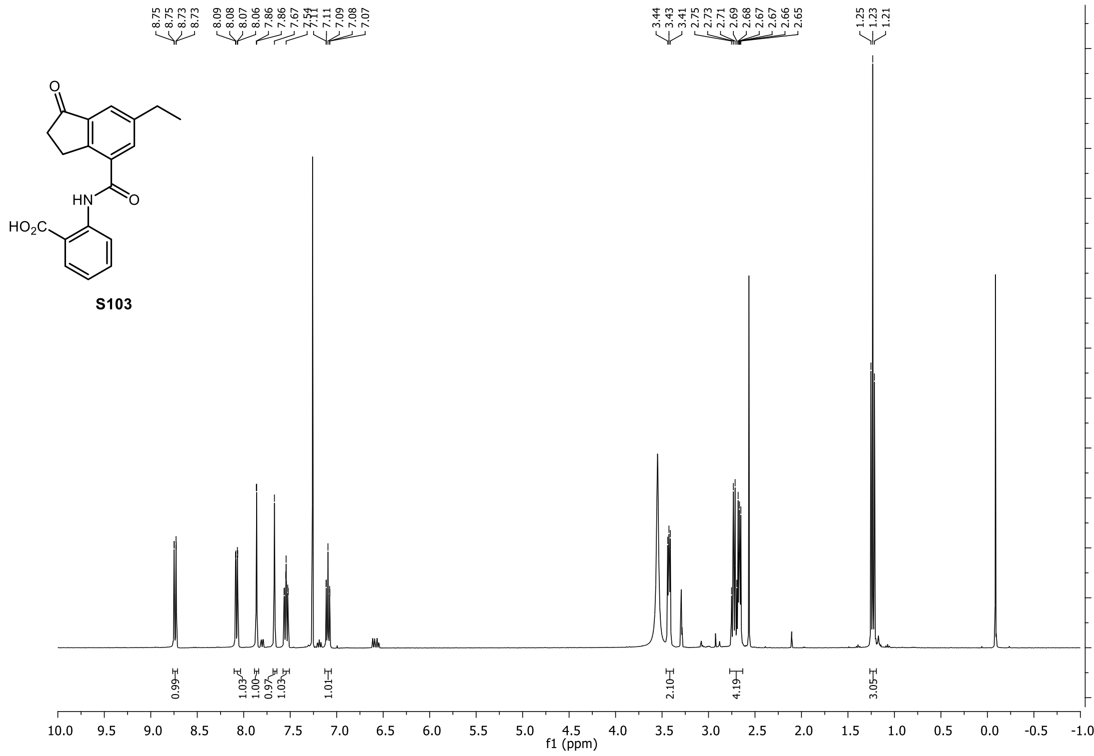

Supplementary Figure 273: ^{13}C NMR S103.

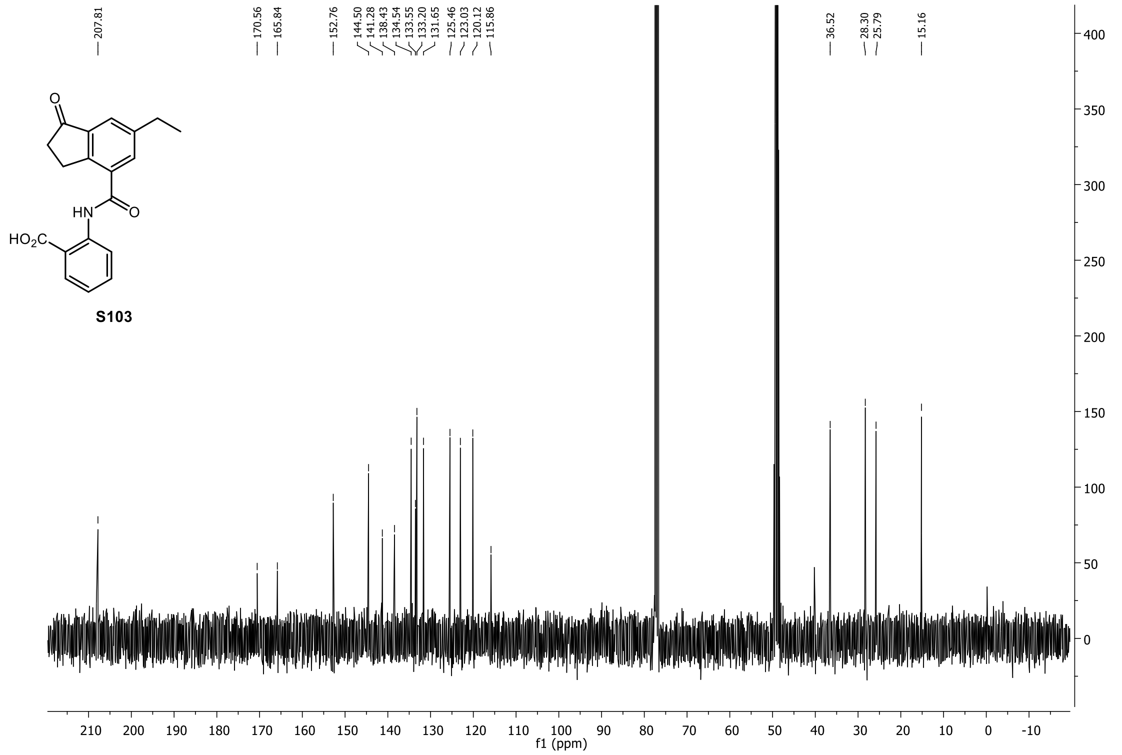

Supplementary Figure 274: ^{1}H NMR S104.

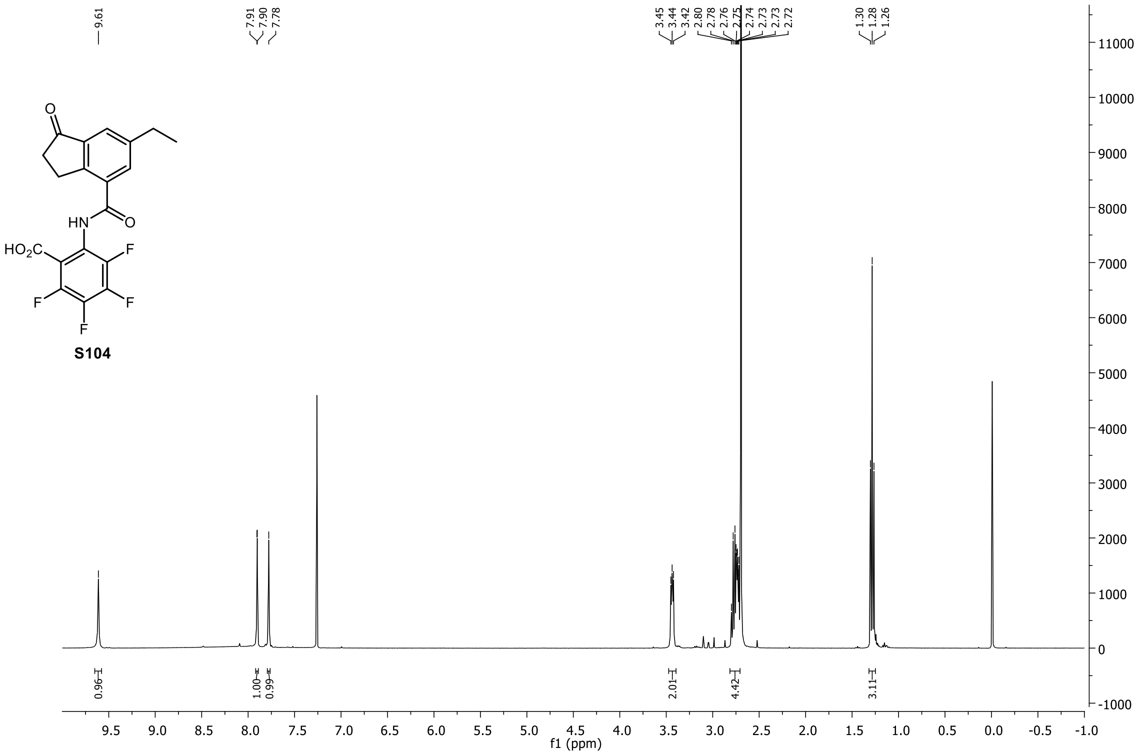

Supplementary Figure 275: ^{13}C NMR S104.

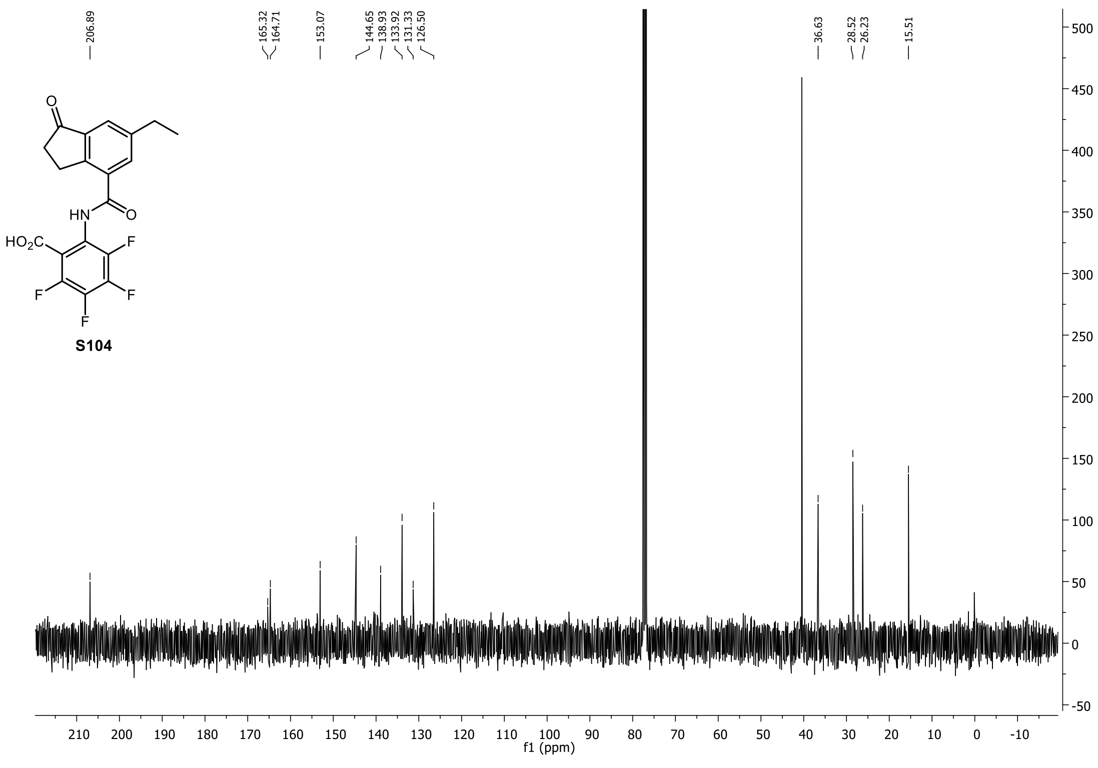

Supplementary Figure 276: ^{1}H NMR S105.

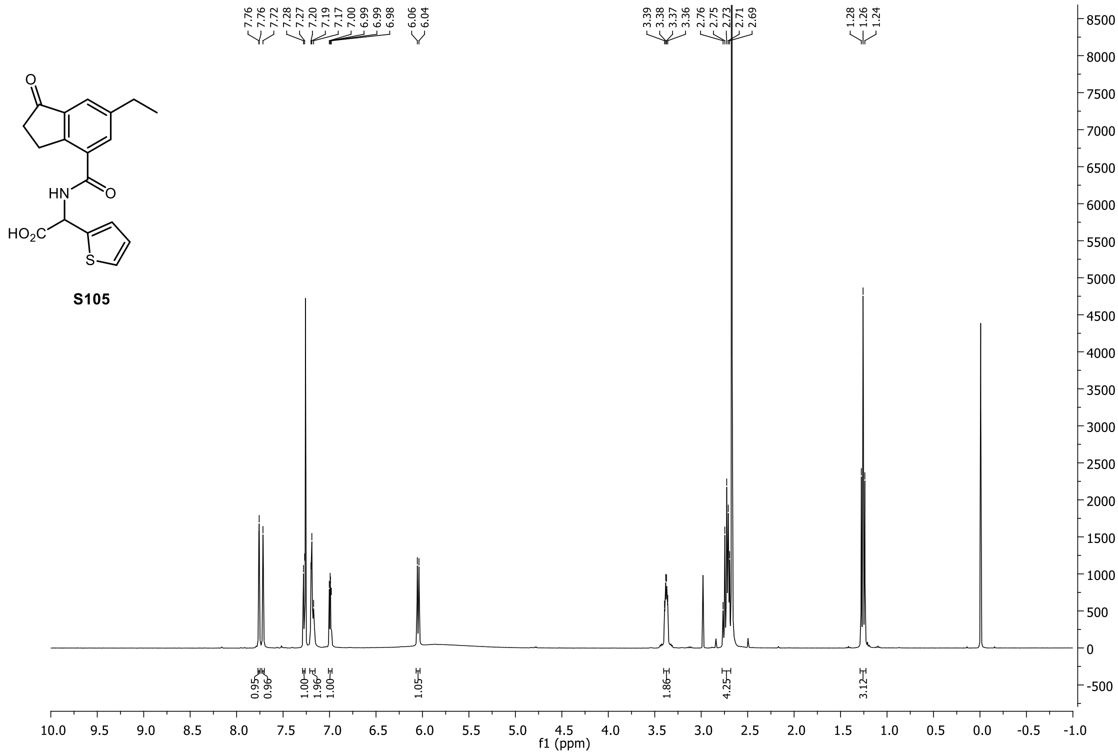

Supplementary Figure 277: ^{13}C NMR S105.

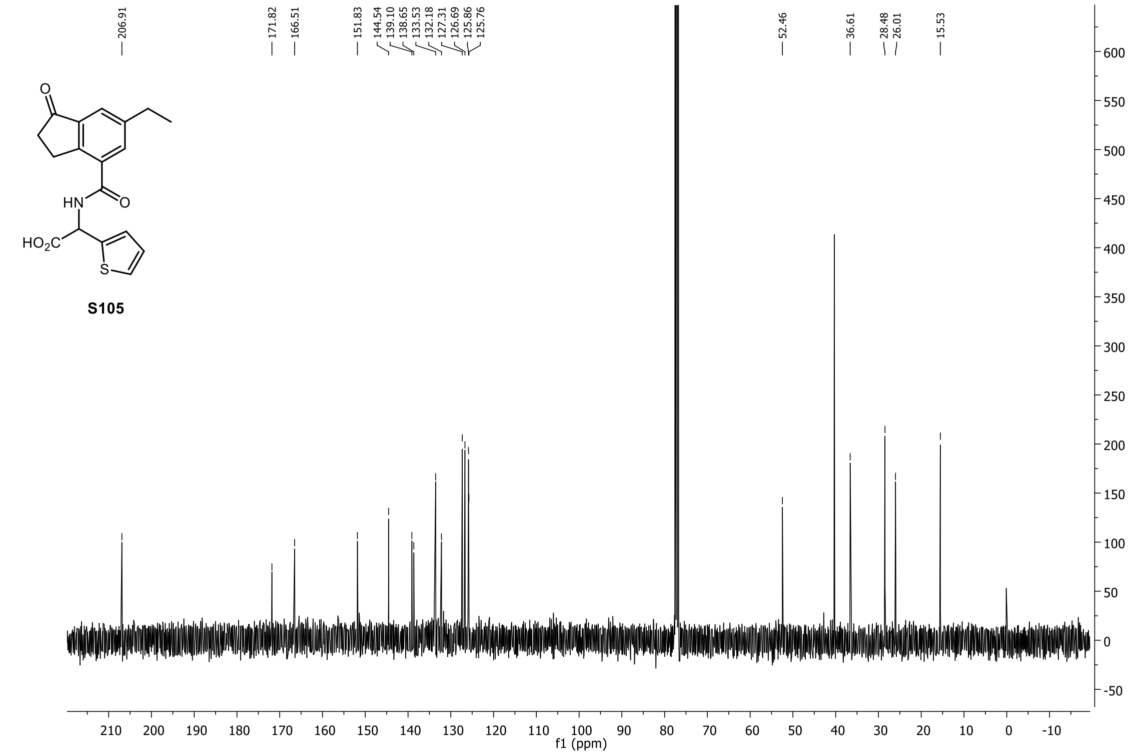

Supplementary Figure 278: ^{1}H NMR S106.

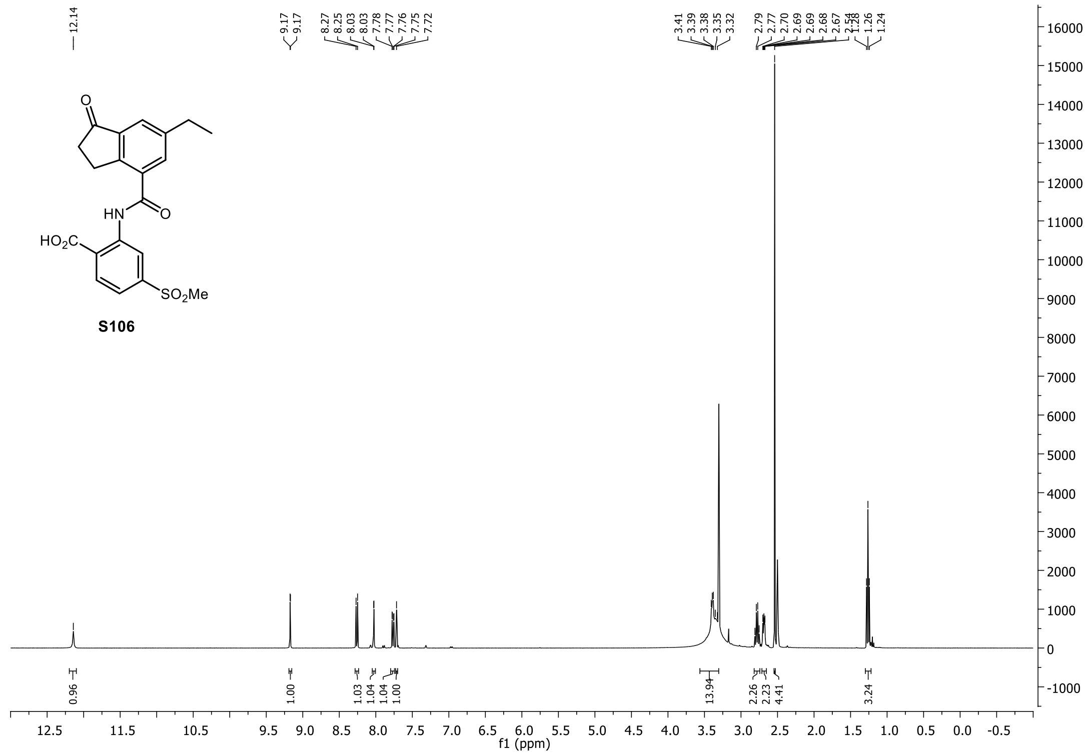

Supplementary Figure 279: ^{13}C NMR S106.

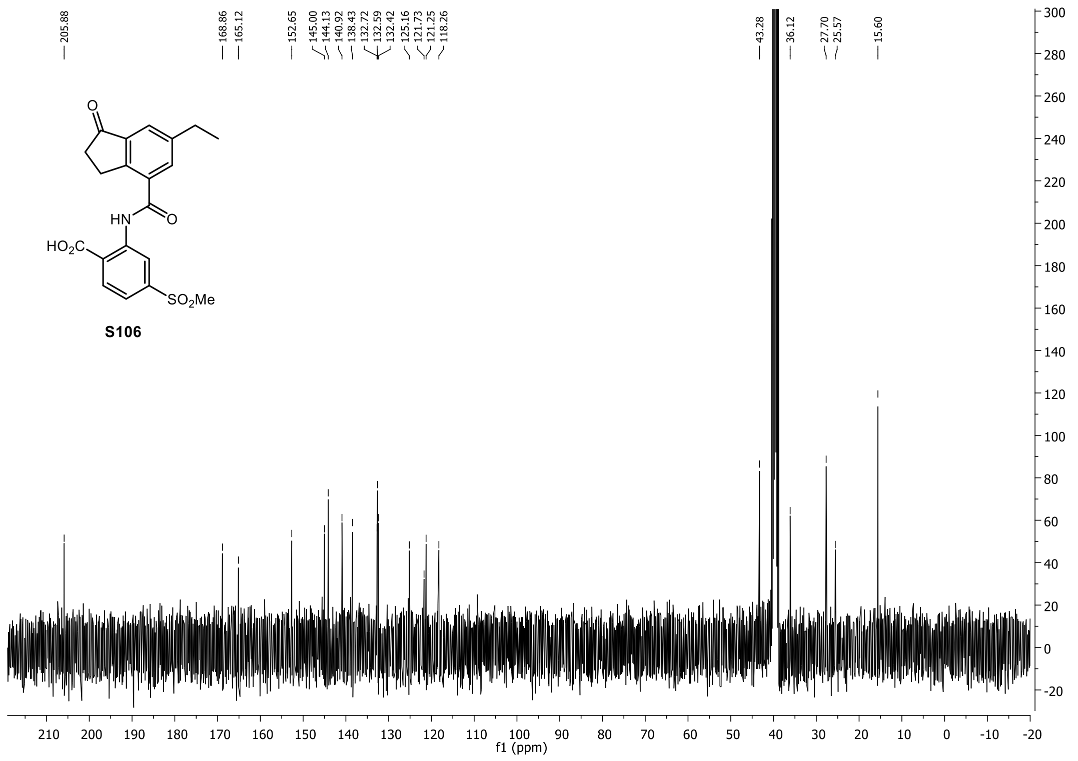

Supplementary Figure 280: ^{1}H NMR S107.

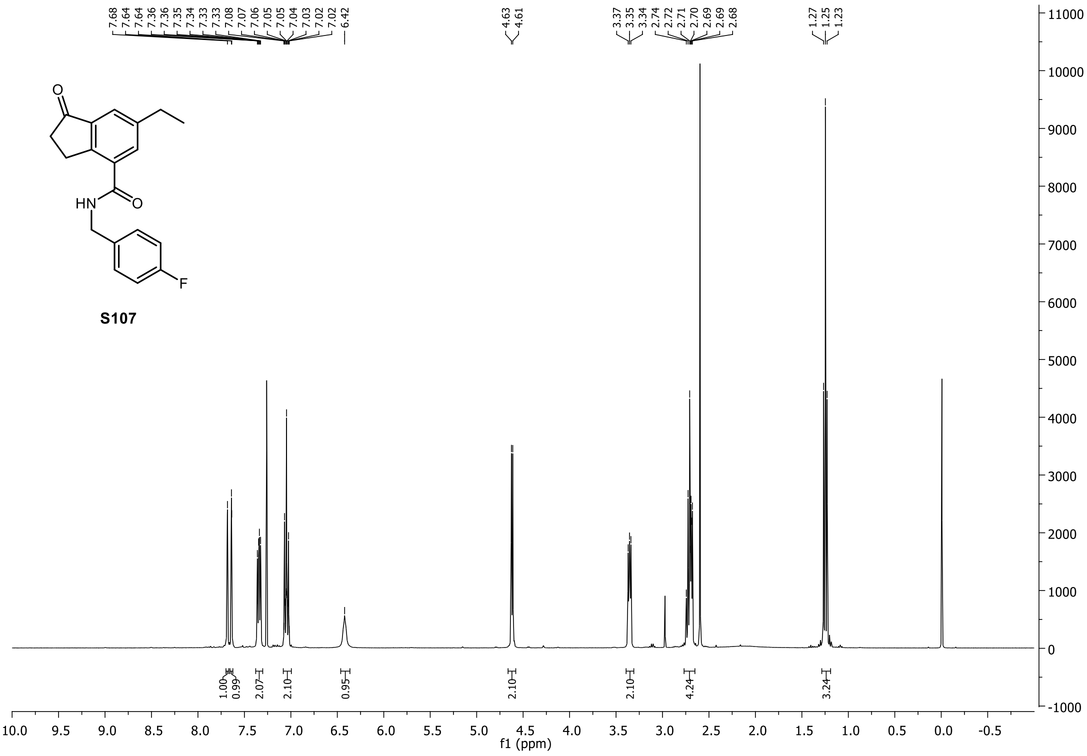

Supplementary Figure 281: ^{13}C NMR S107.

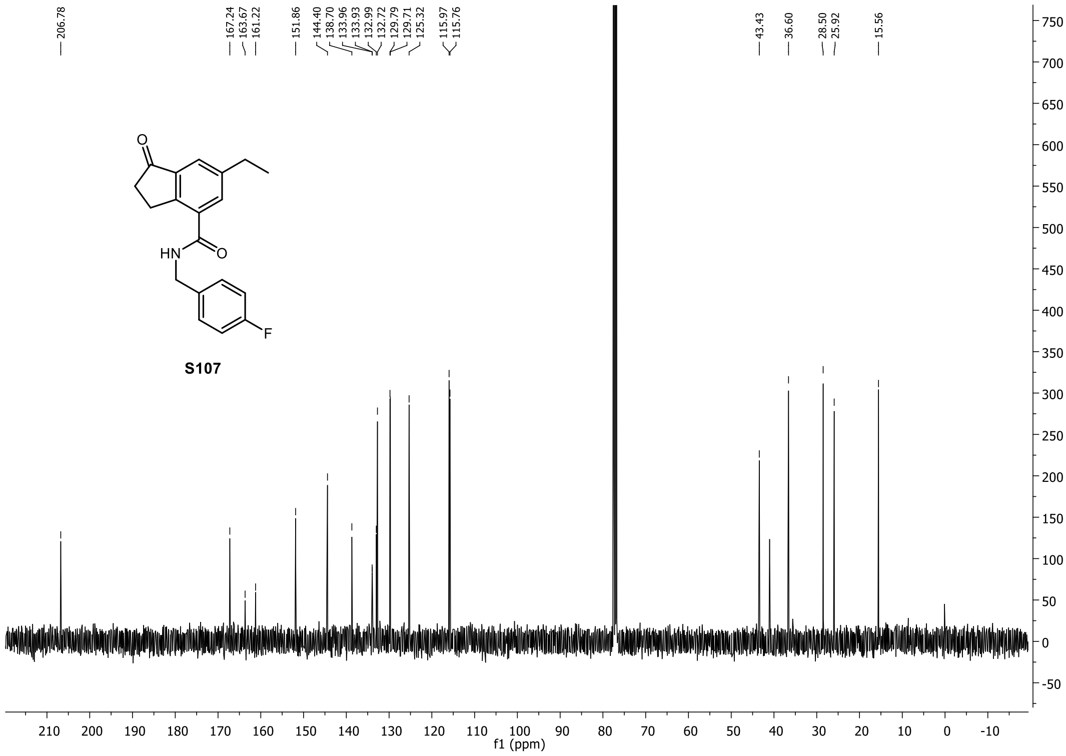

Supplementary Figure 282: ^{1}H NMR S108.

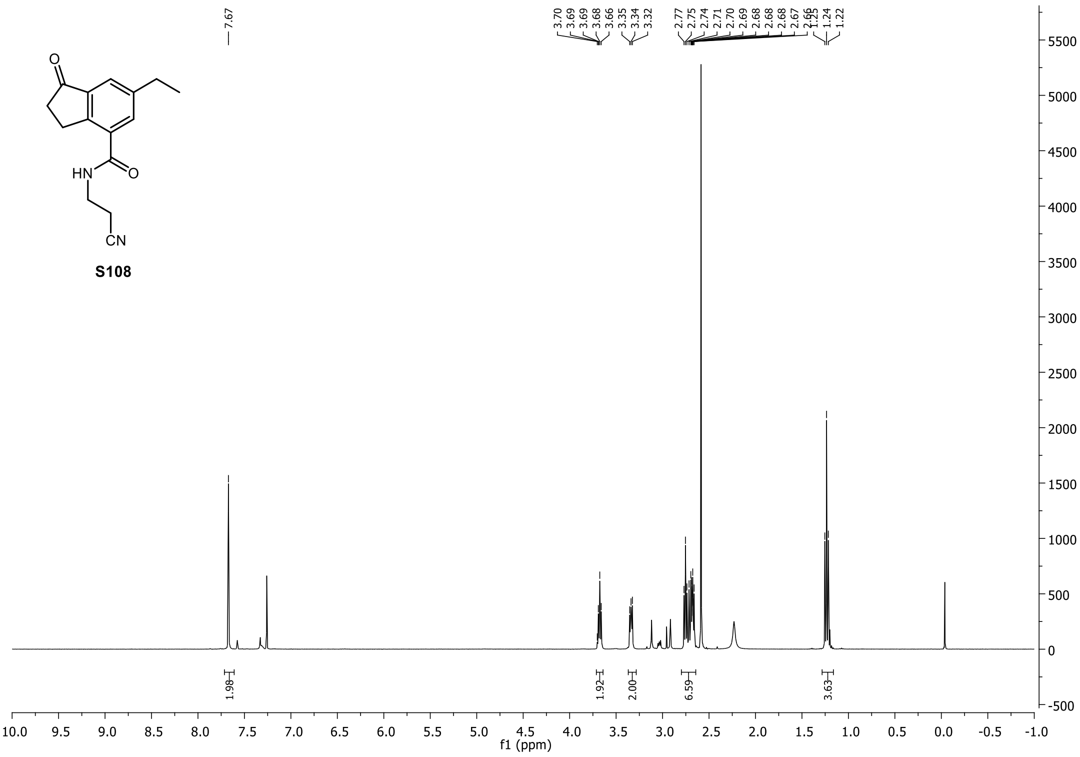

Supplementary Figure 283: ^{13}C NMR S108.

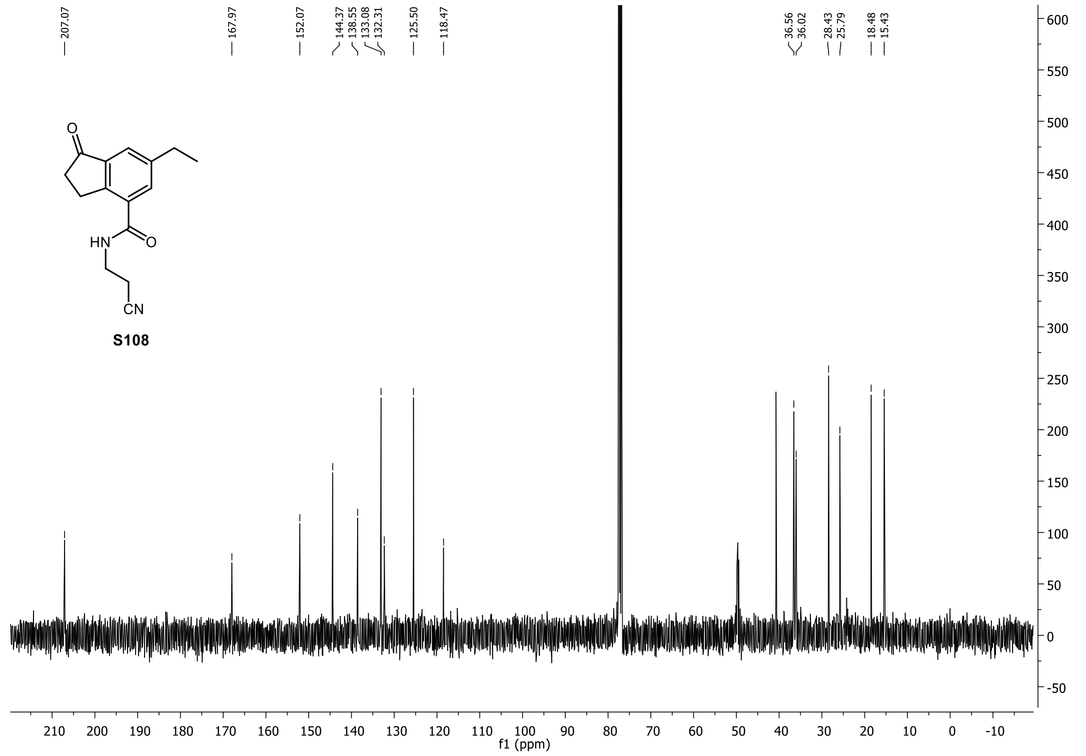

Supplementary Figure 284: ^{1}H NMR S109.

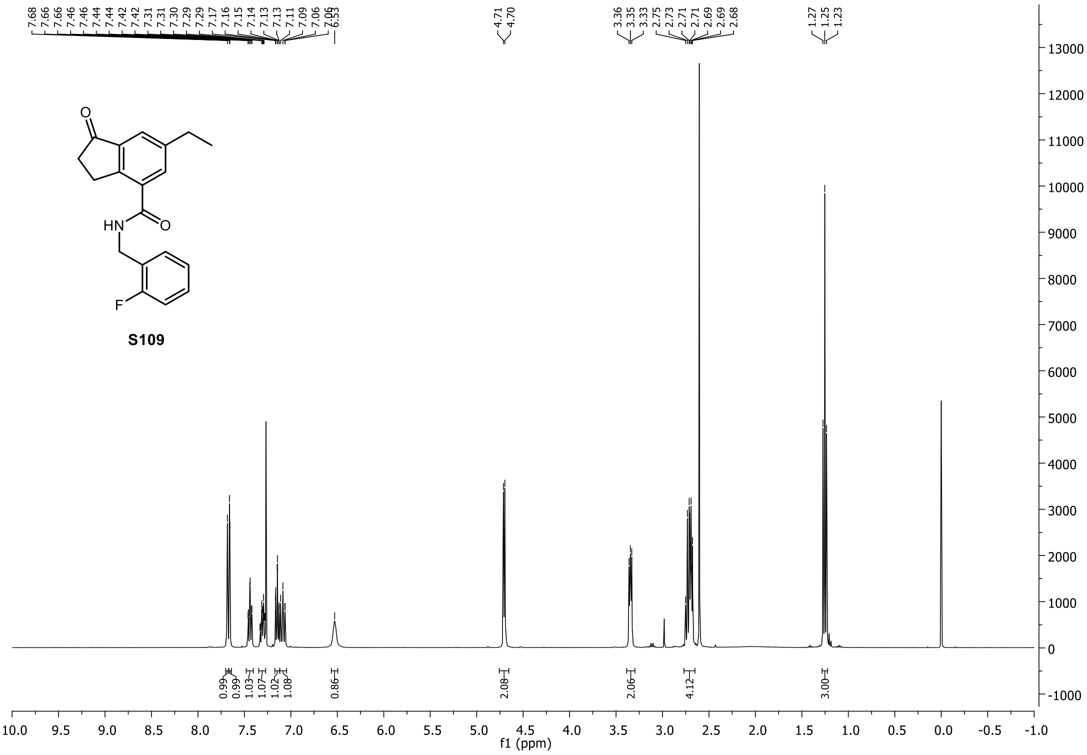

Supplementary Figure 285: ^{13}C NMR S109.

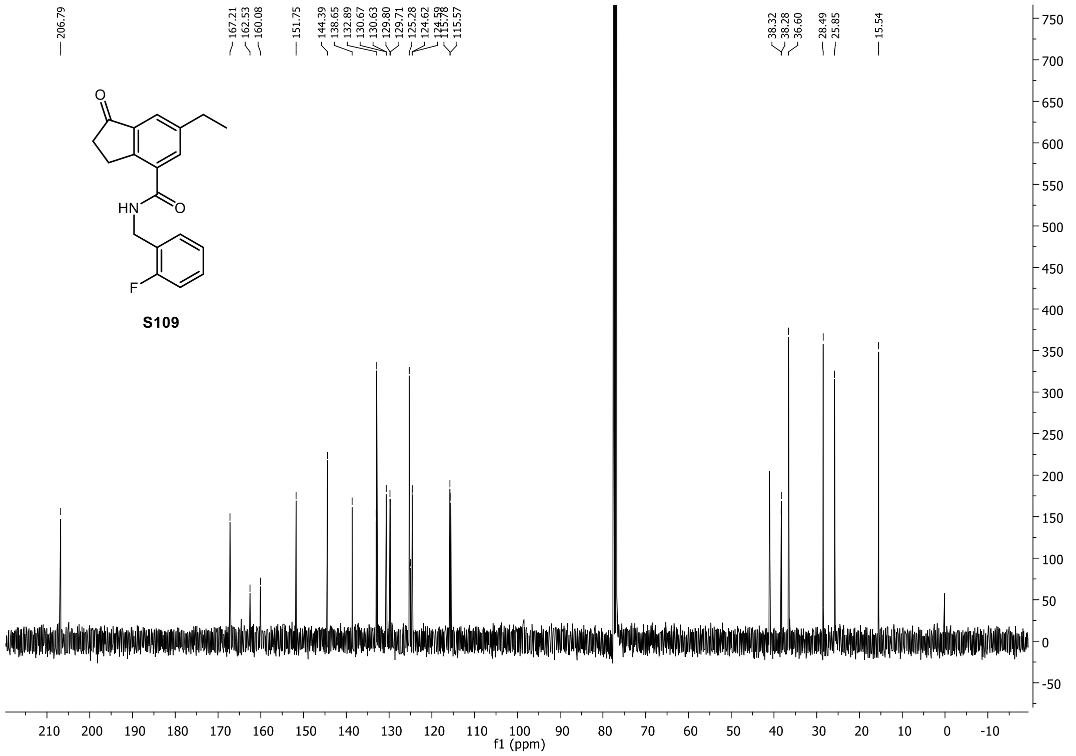

Supplementary Figure 286: ^{1}H NMR S110.

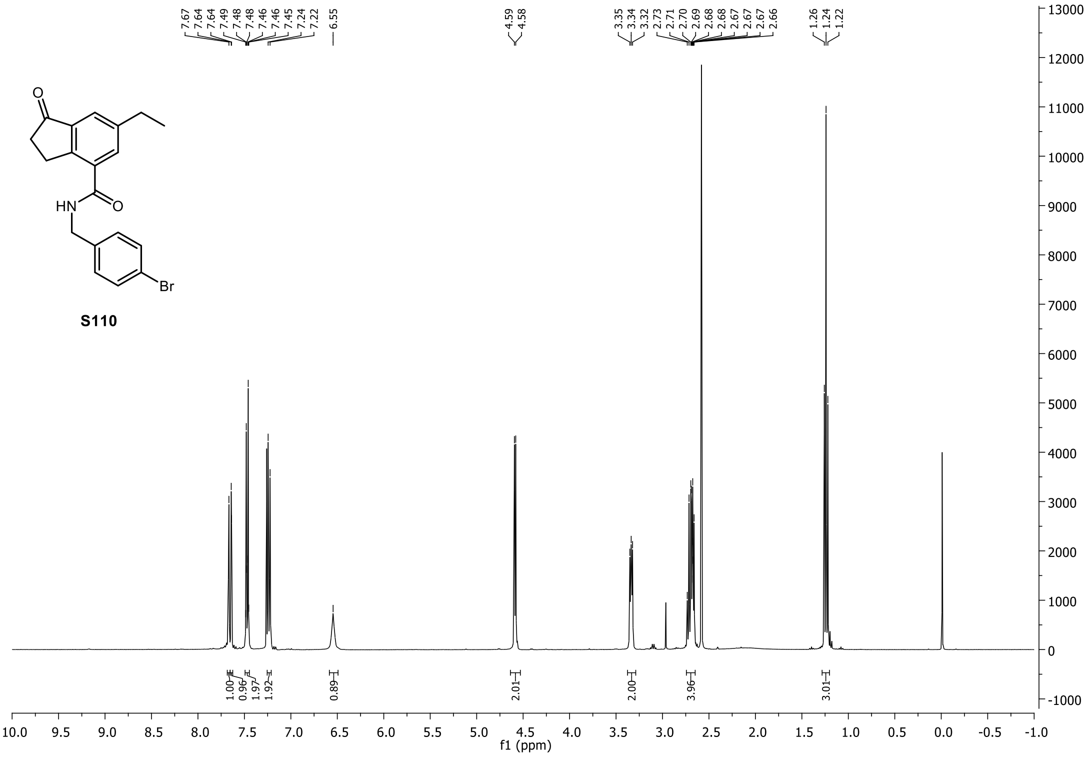

Supplementary Figure 287: ^{13}C NMR S110.

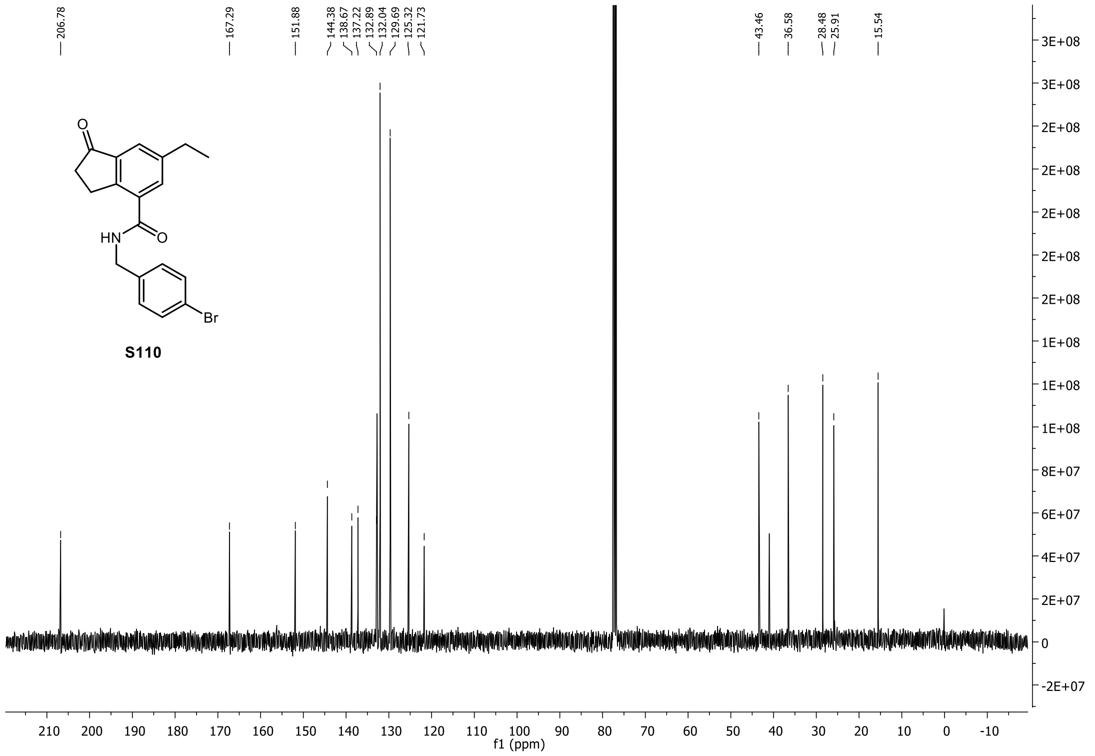

Supplementary Figure 288: ^{1}H NMR S111.

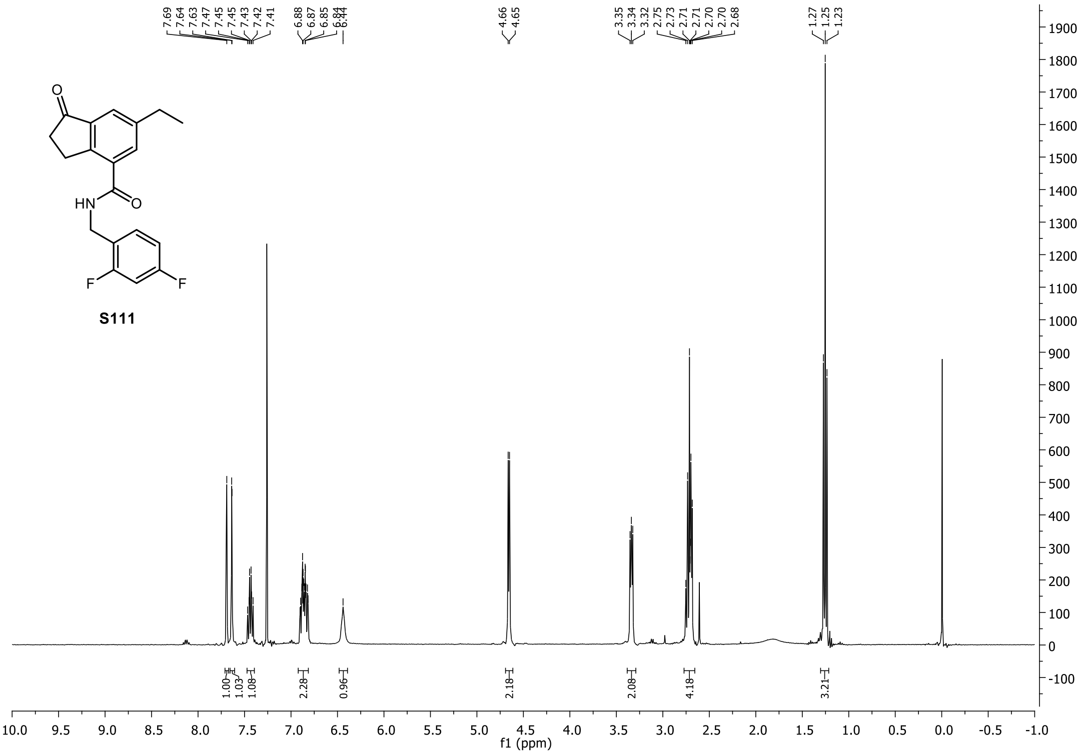

Supplementary Figure 289: ^{13}C NMR S111.

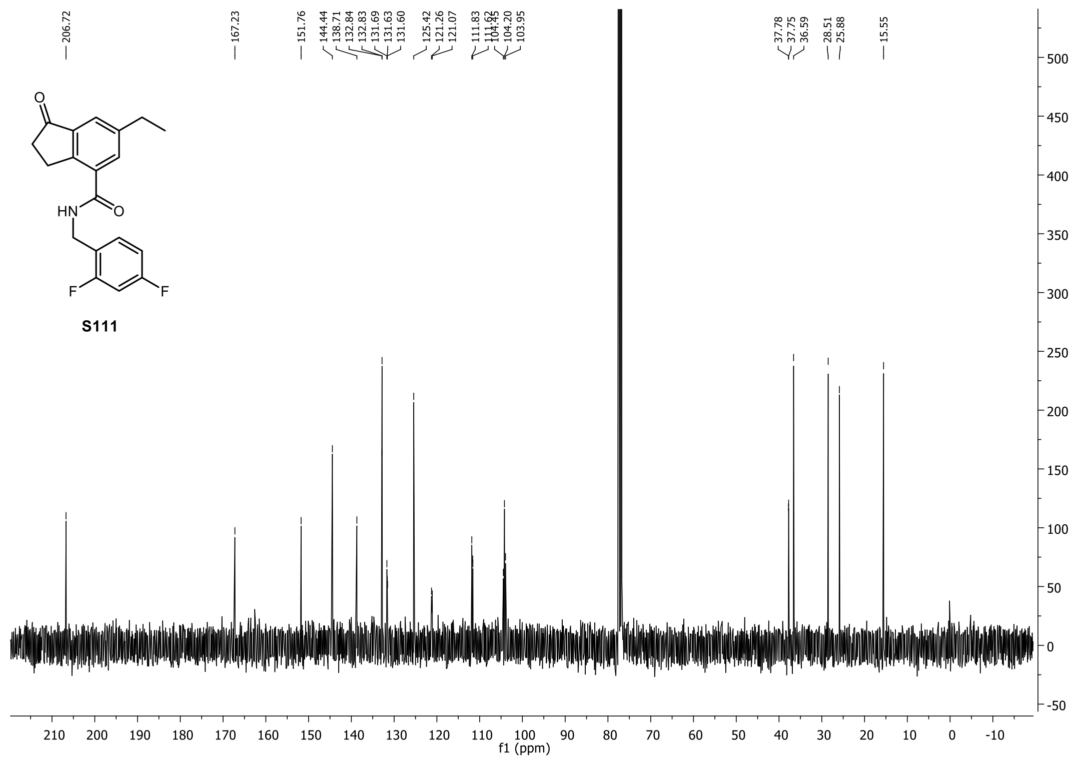

Supplementary Figure 290: ^{1}H NMR S112.

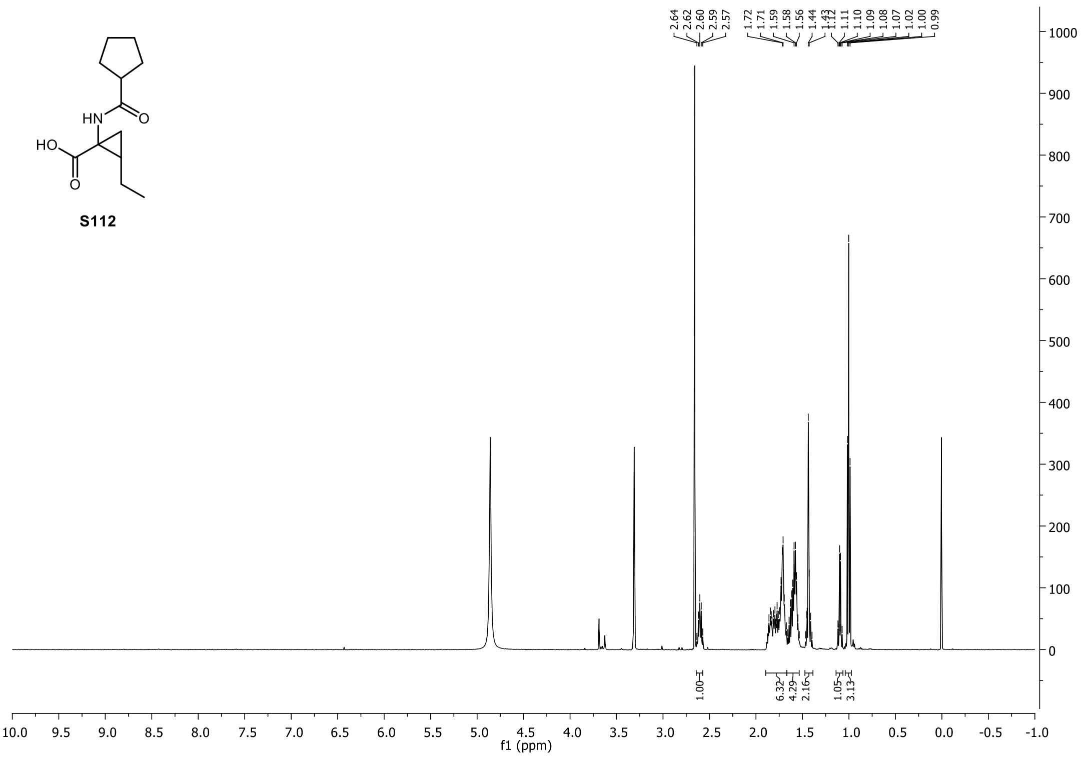

Supplementary Figure 291: ^{13}C NMR S112.

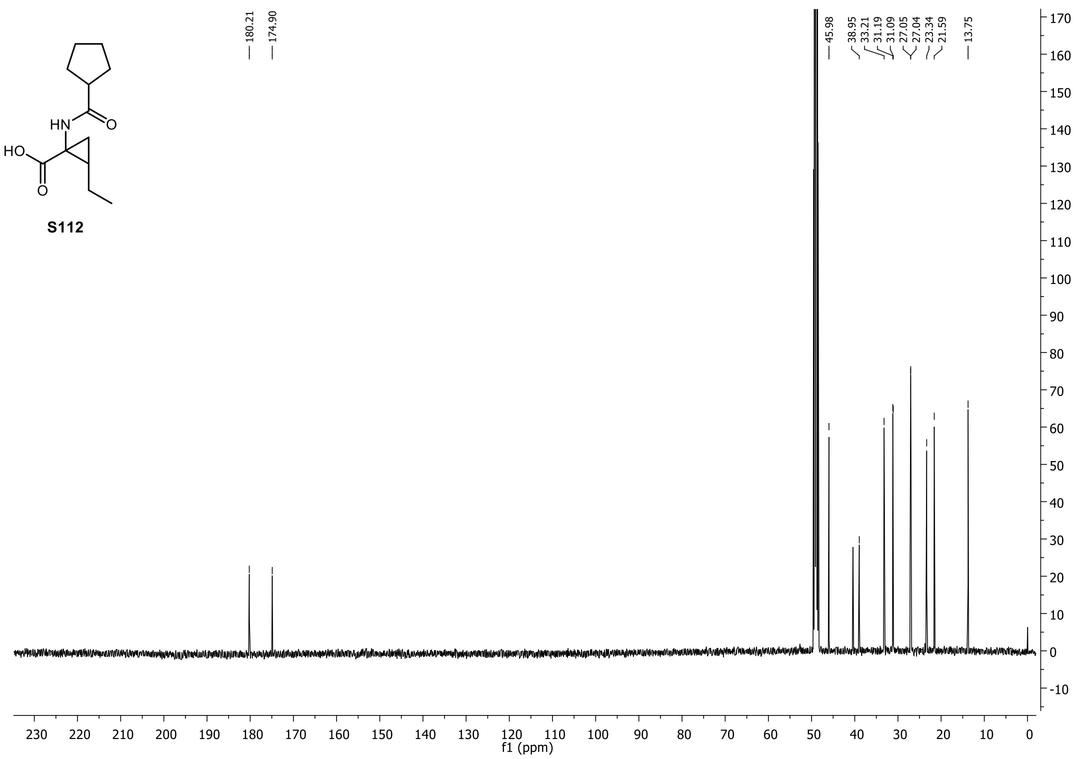

Supplementary Figure 292: ^{1}H NMR S113.

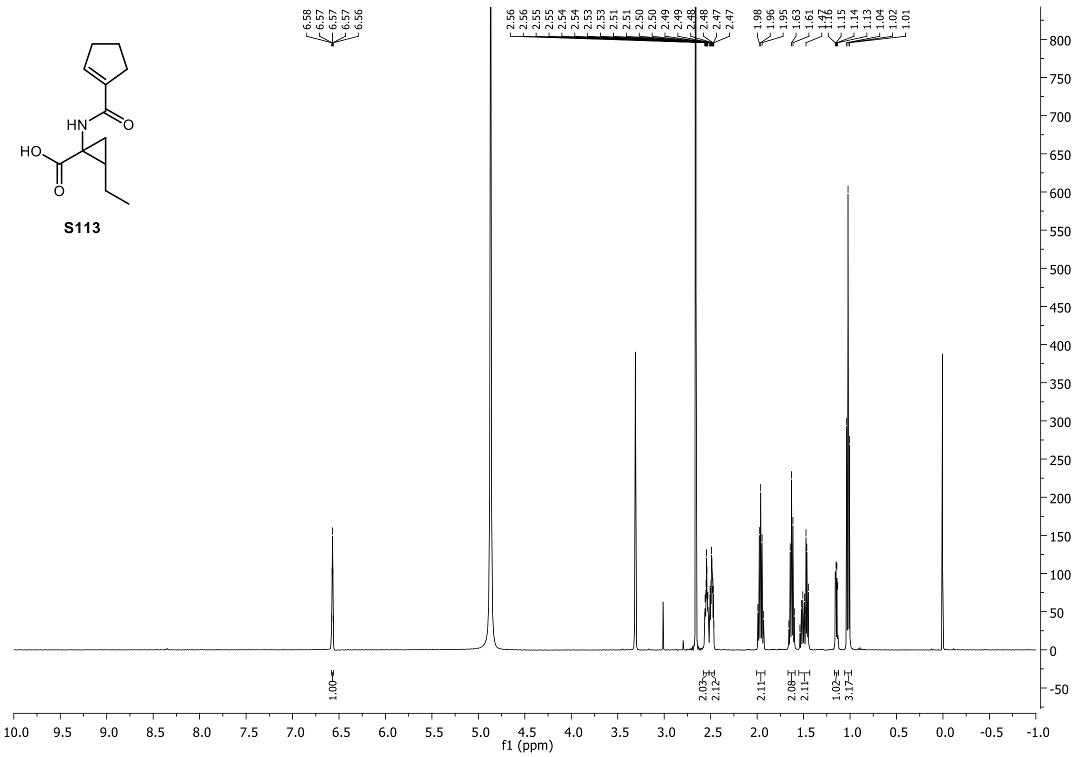

Supplementary Figure 293: ^{13}C NMR S113.

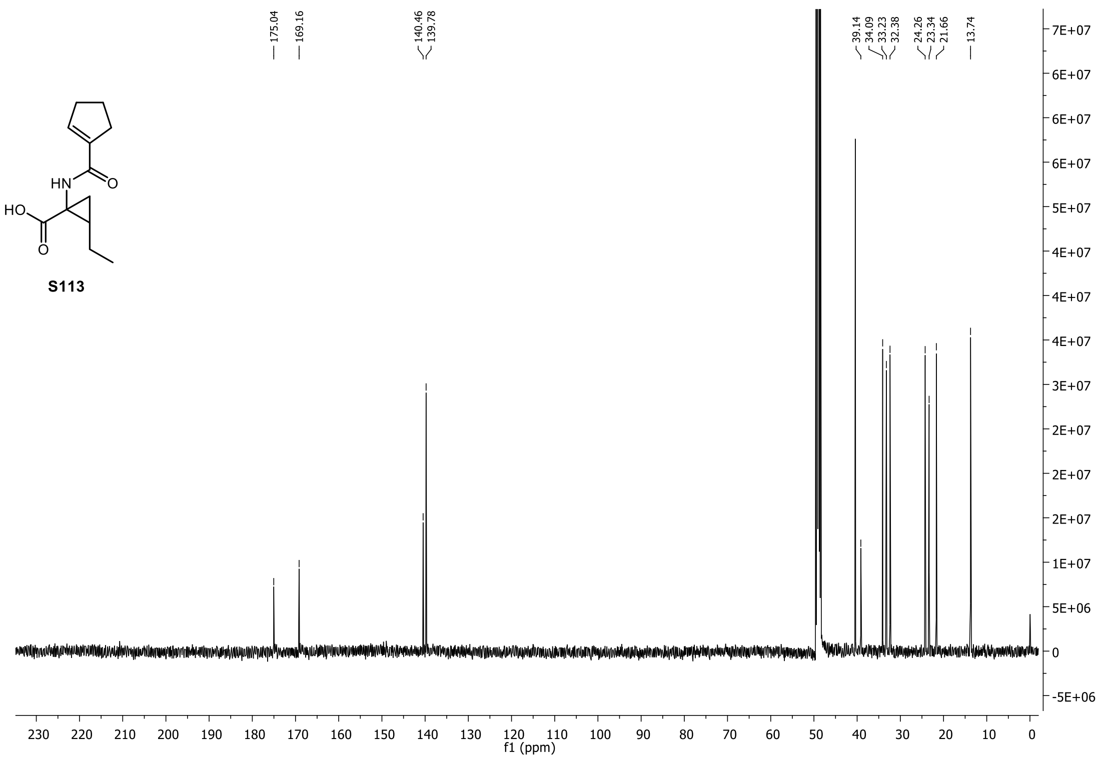

S113

Supplementary Figure 294: ^{1}H NMR S114.

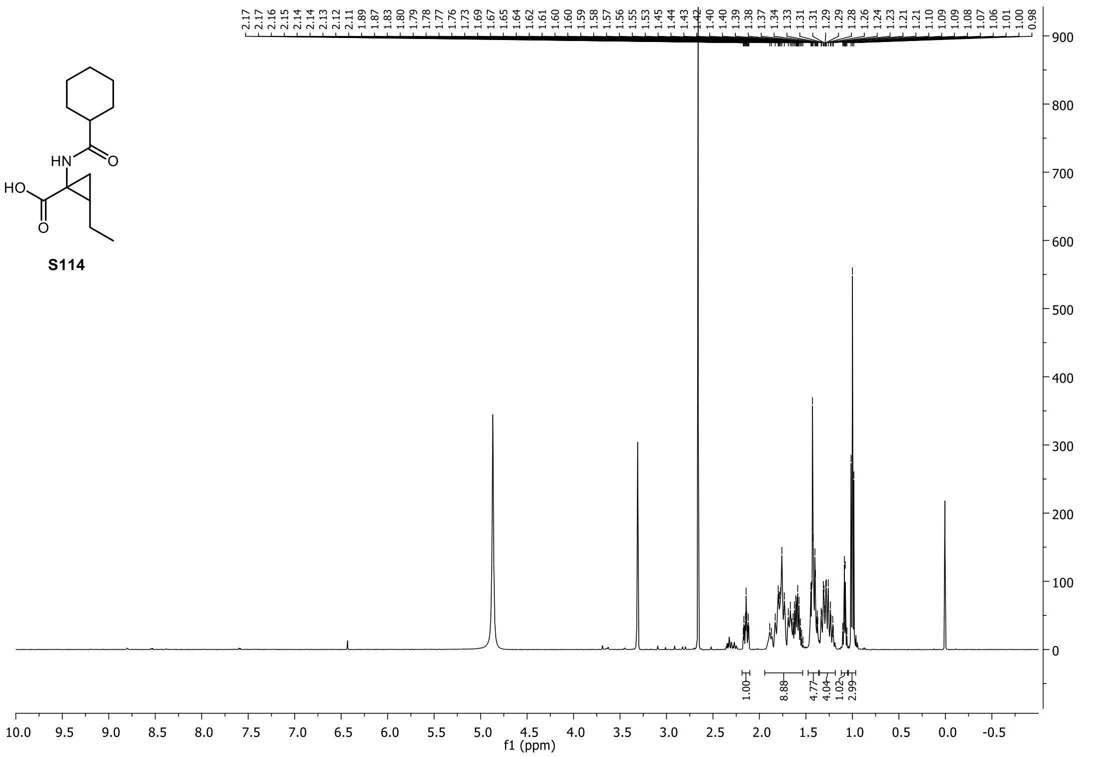

S114

Supplementary Figure 295: ^{13}C NMR S114.

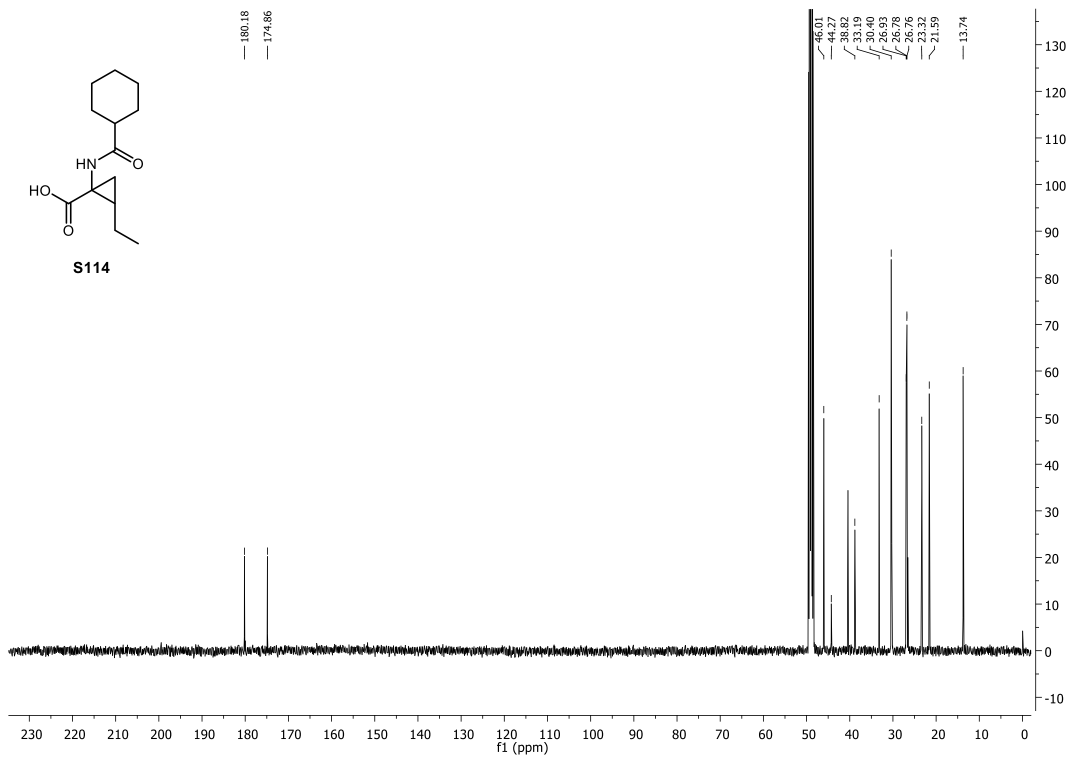

Supplementary Figure 296: ^{1}H NMR S115.

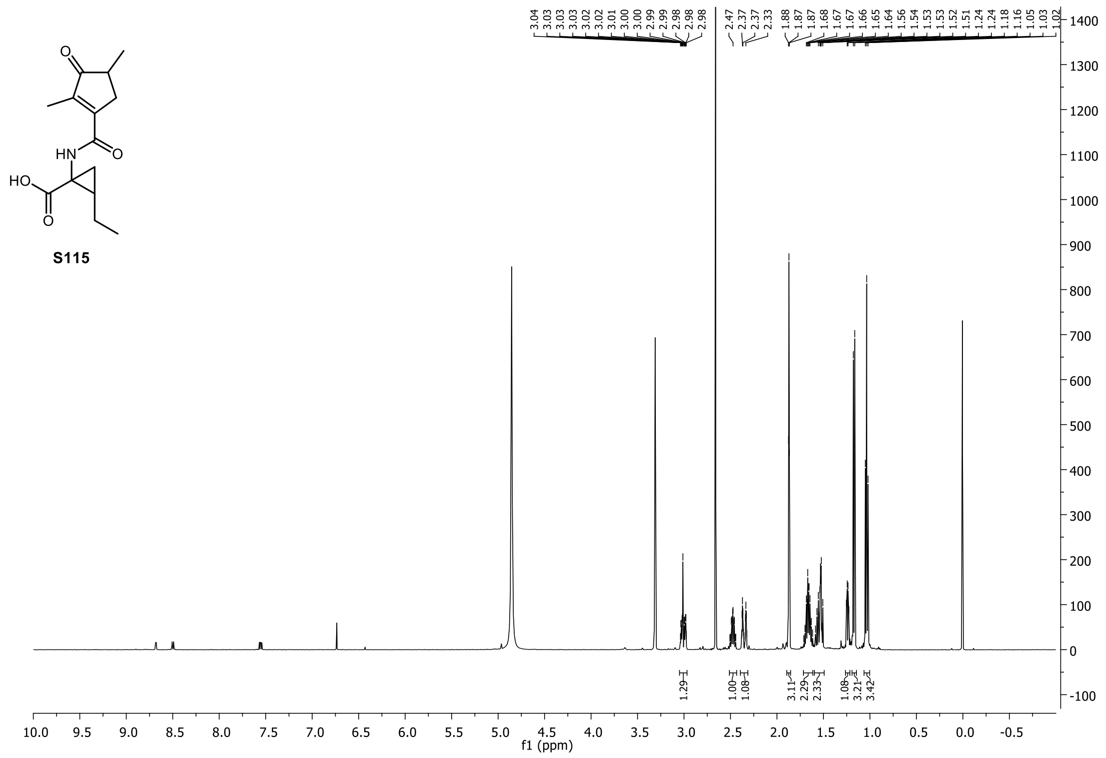

Supplementary Figure 297: ^{13}C NMR S115.

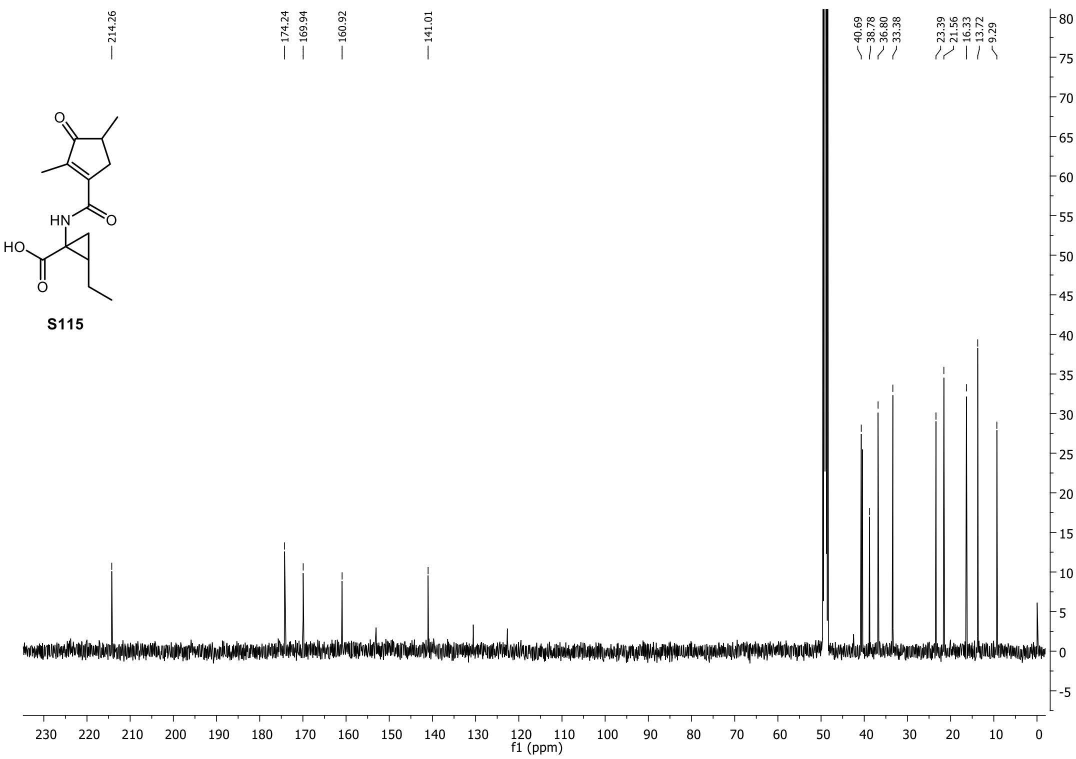

Supplementary Figure 298: ^{1}H NMR S116.

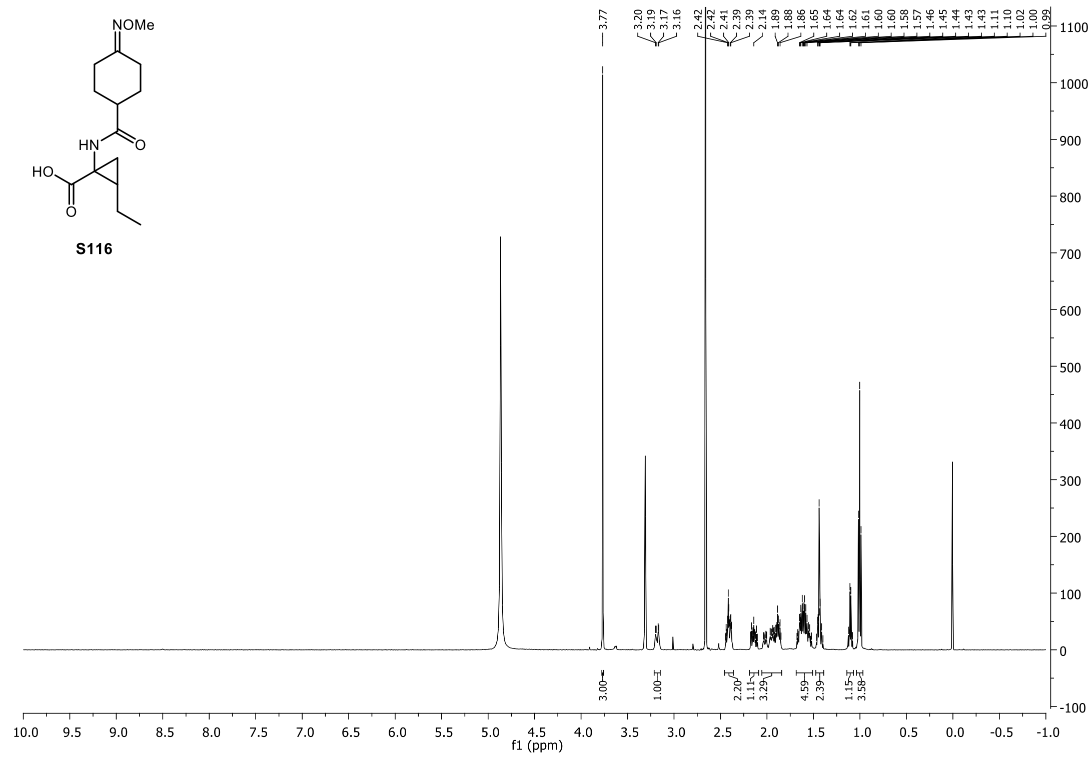

Supplementary Figure 299: ^{13}C NMR S116.

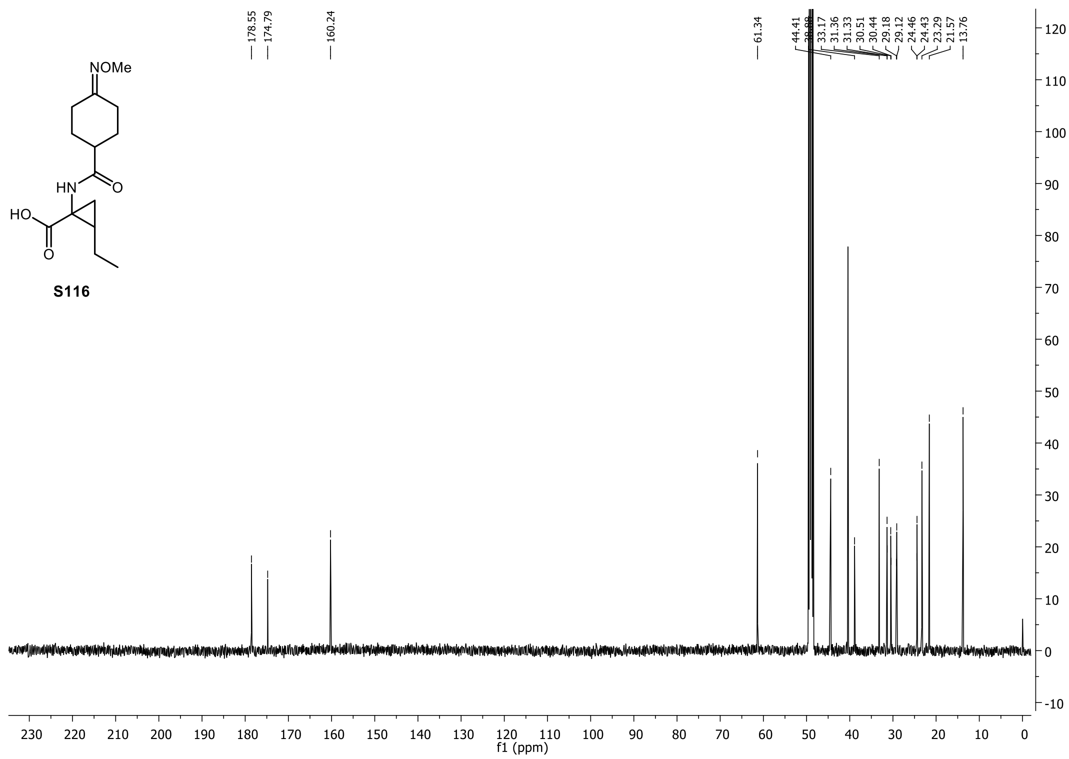

Supplementary Figure 300: ^{1}H NMR S117.

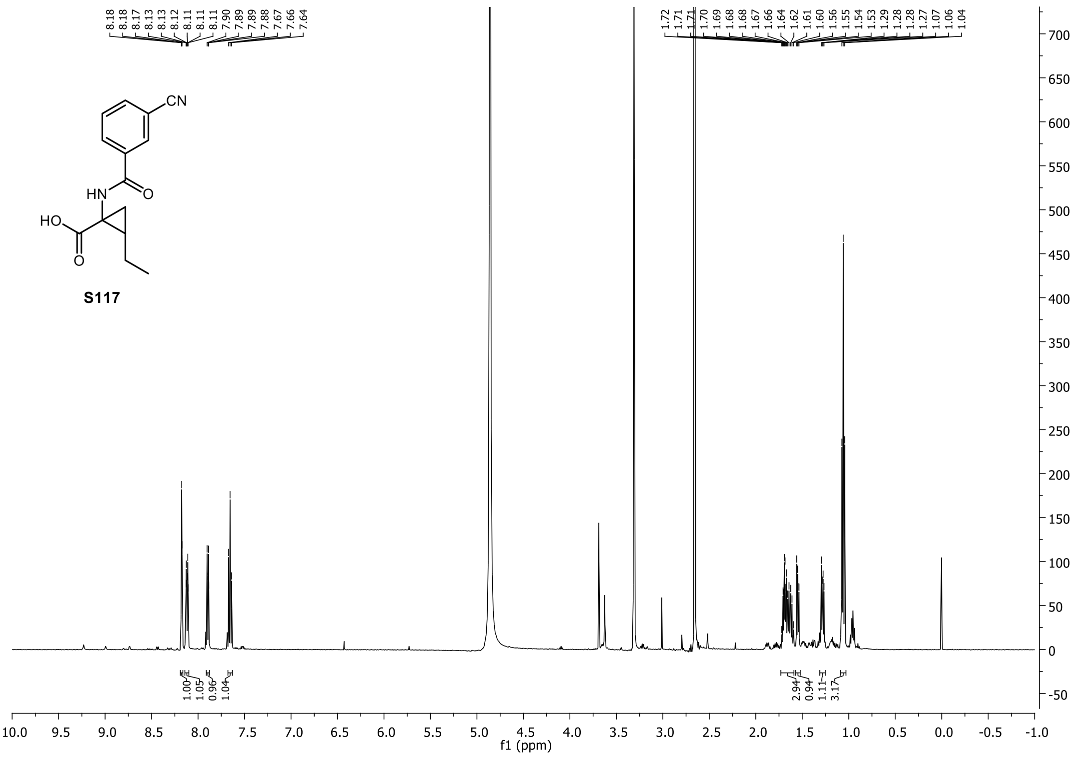

Supplementary Figure 301: ^{13}C NMR S117.

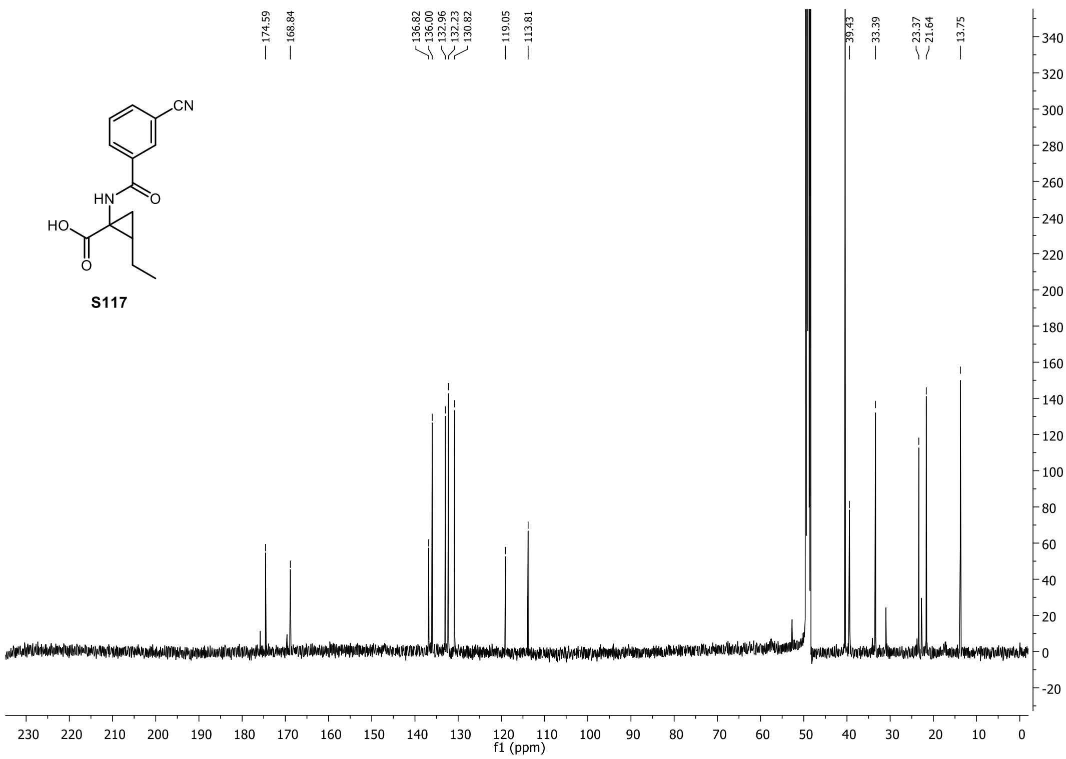

Supplementary Figure 302: ^{1}H NMR S118.

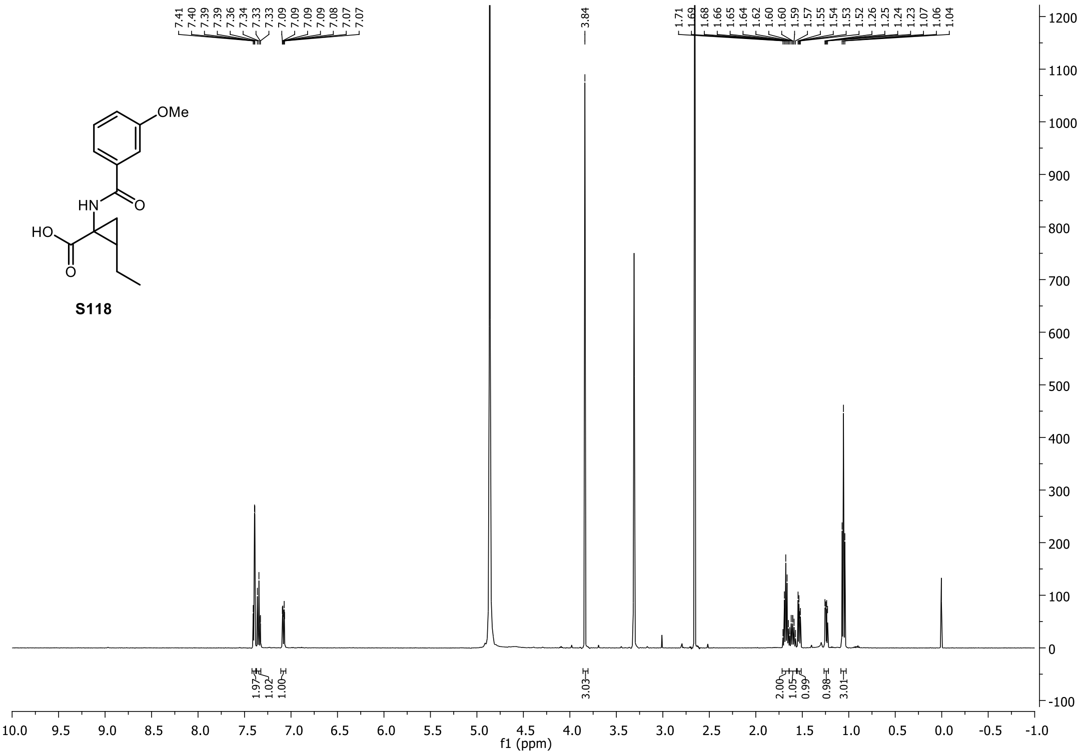

Supplementary Figure 303: ^{13}C NMR S118.

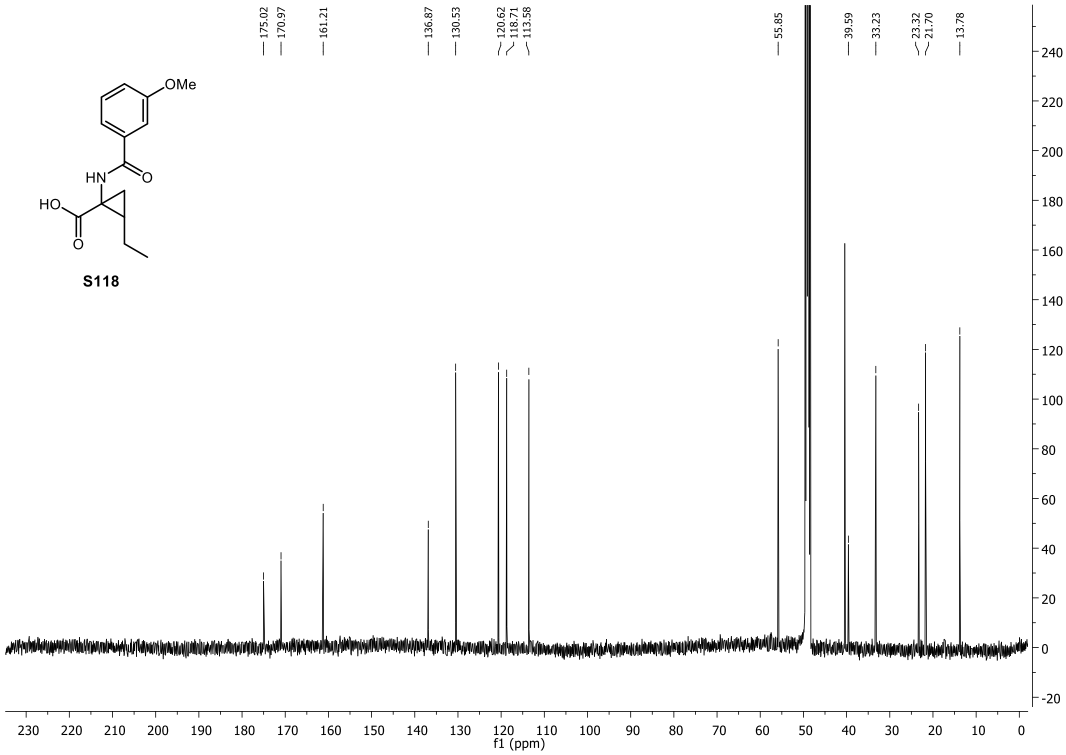

Supplementary Figure 304: ^{1}H NMR S119.

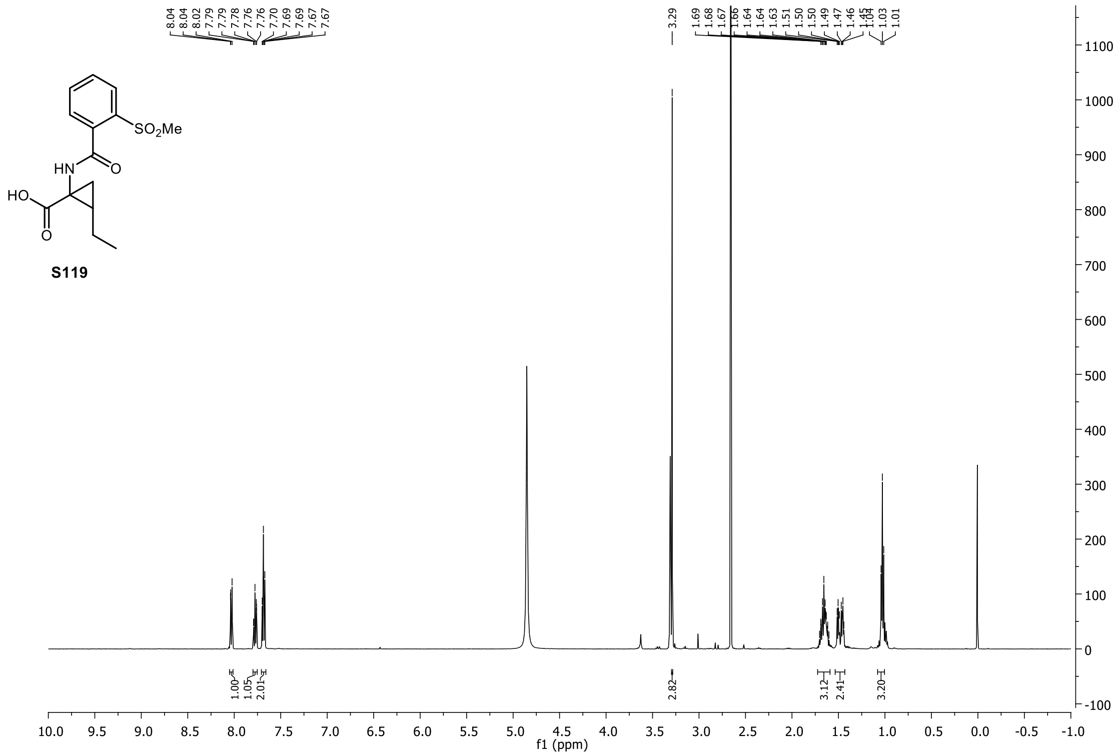

Supplementary Figure 305: ^{13}C NMR S119.

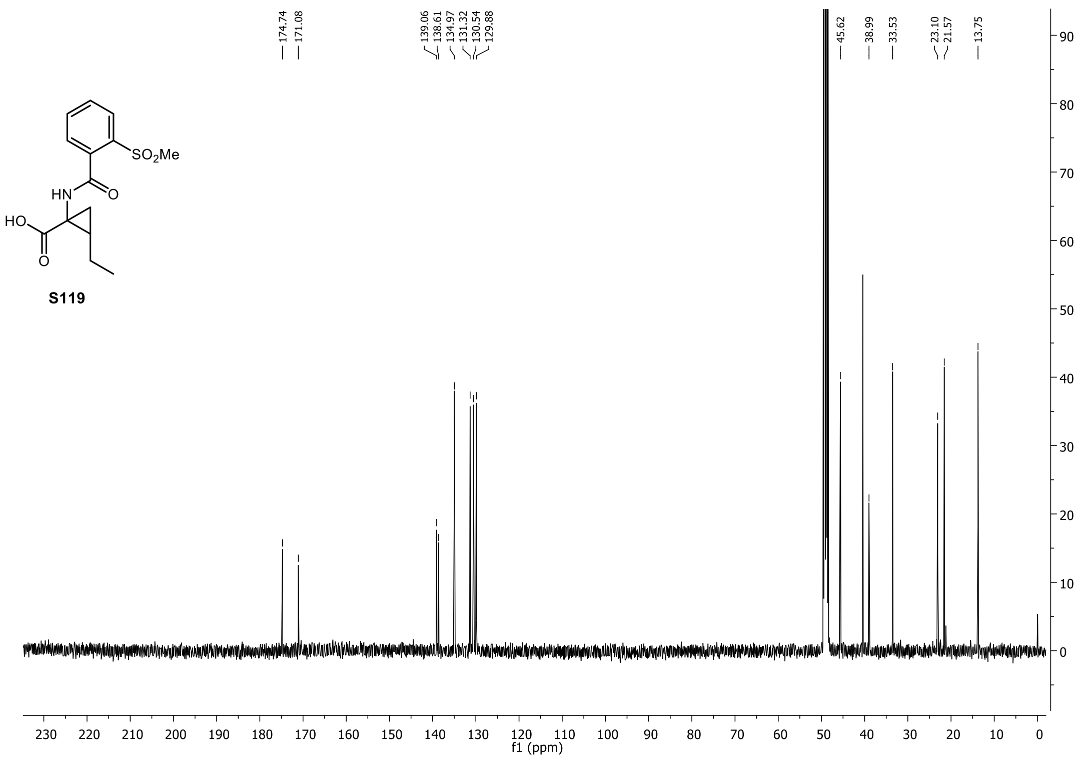

Supplementary Figure 306: ^{1}H NMR S120.

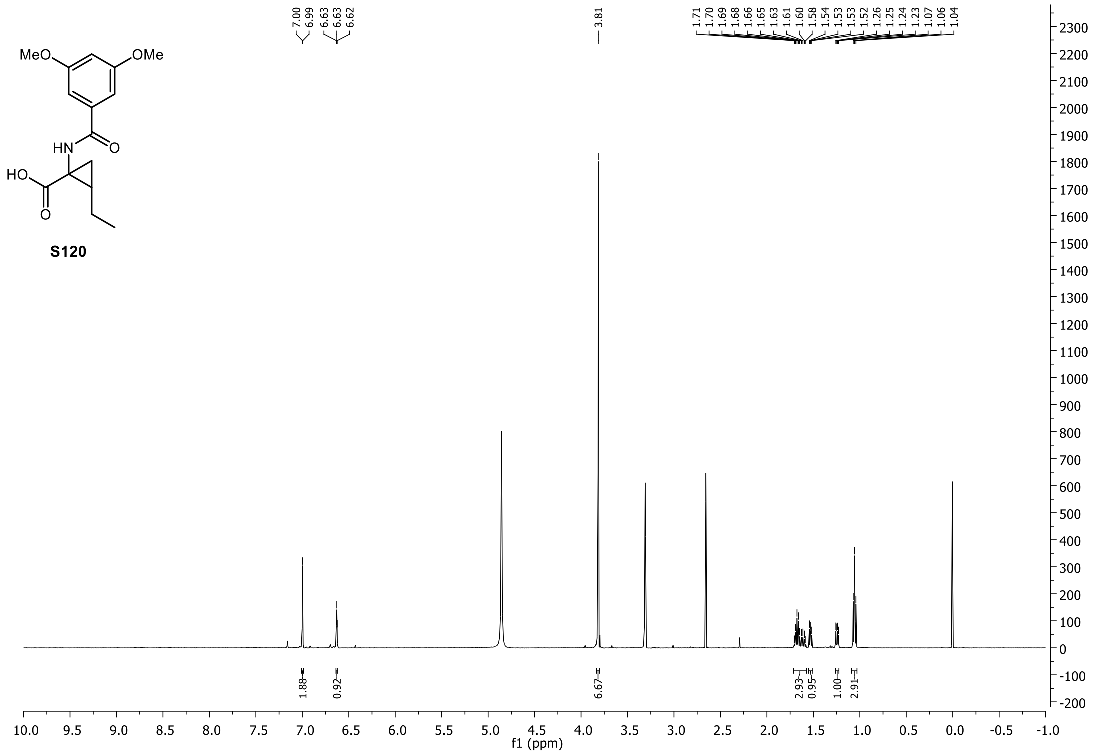

Supplementary Figure 307: ^{13}C NMR S120.

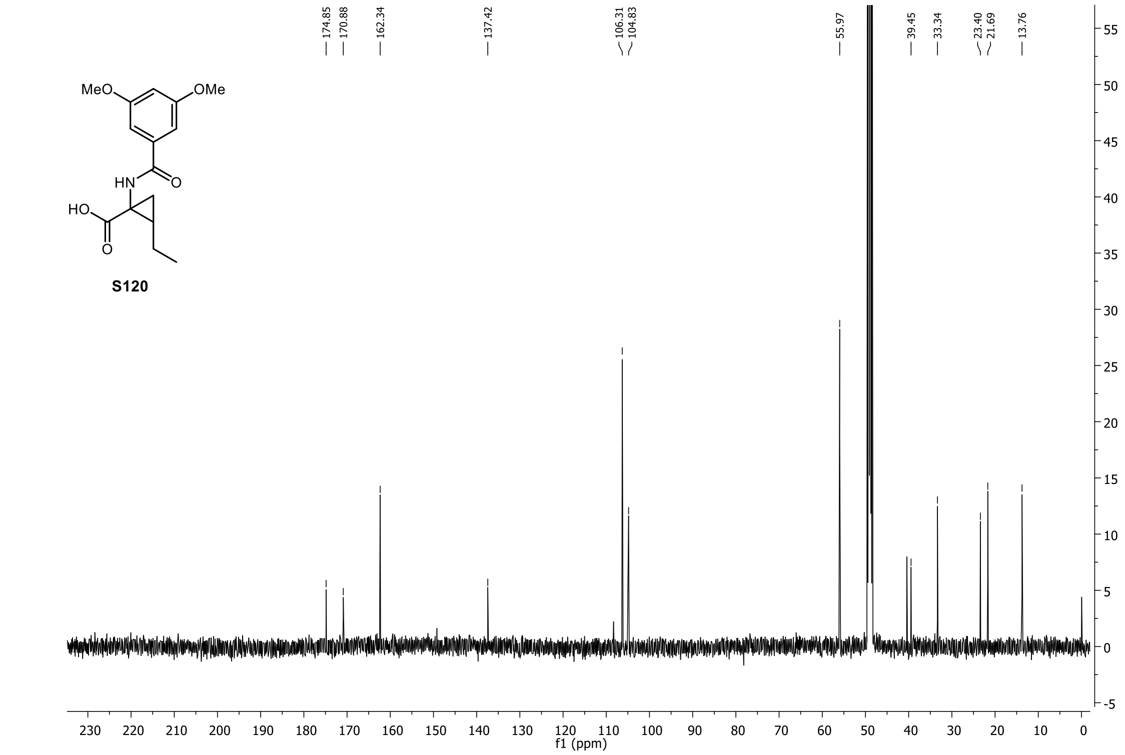

Supplementary Figure 308: ^{1}H NMR S121.

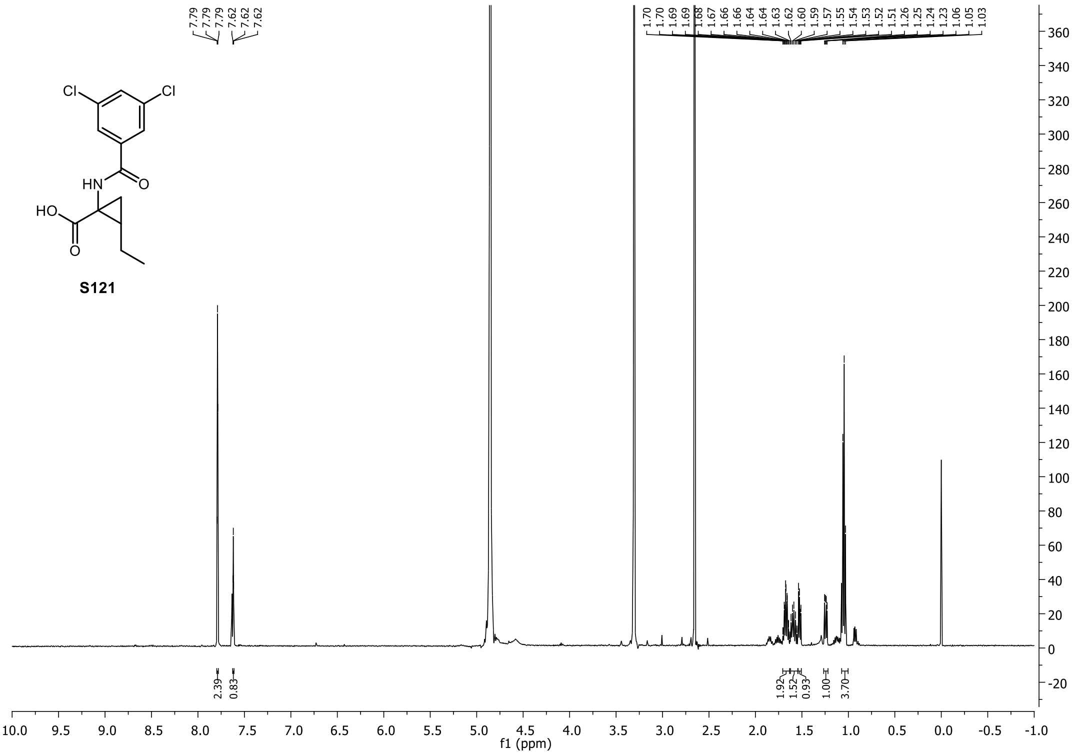

Supplementary Figure 309: ^{13}C NMR S121.

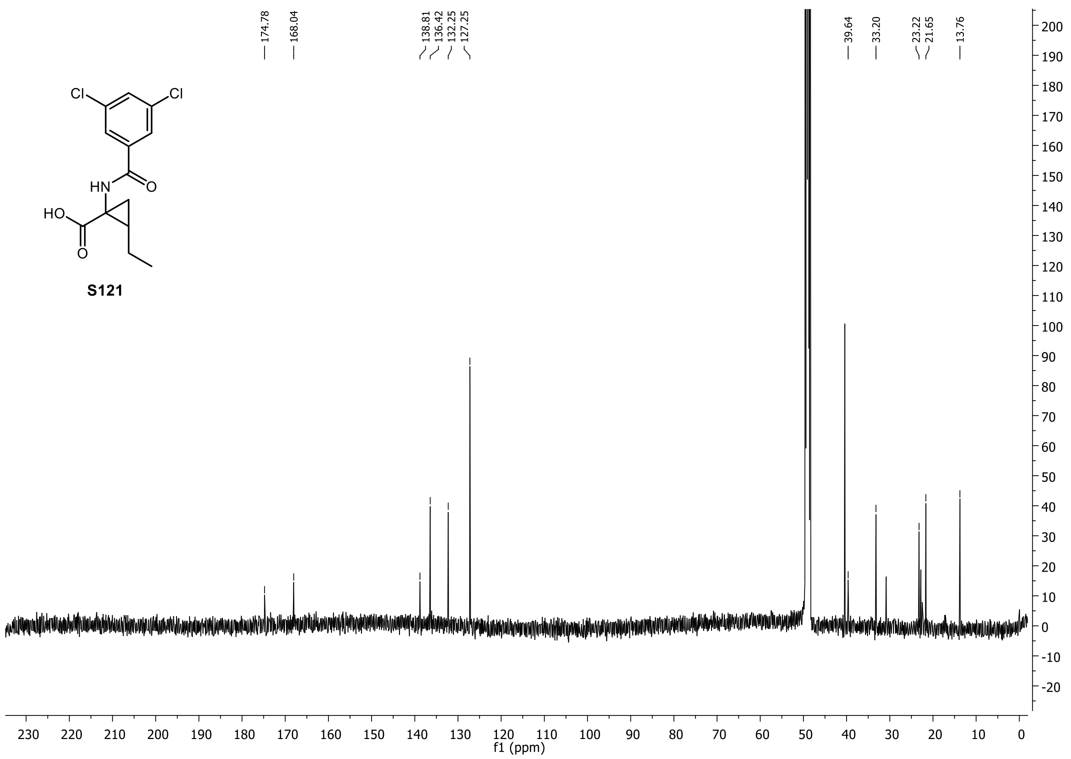

Supplementary Figure 310: ^{1}H NMR S122.

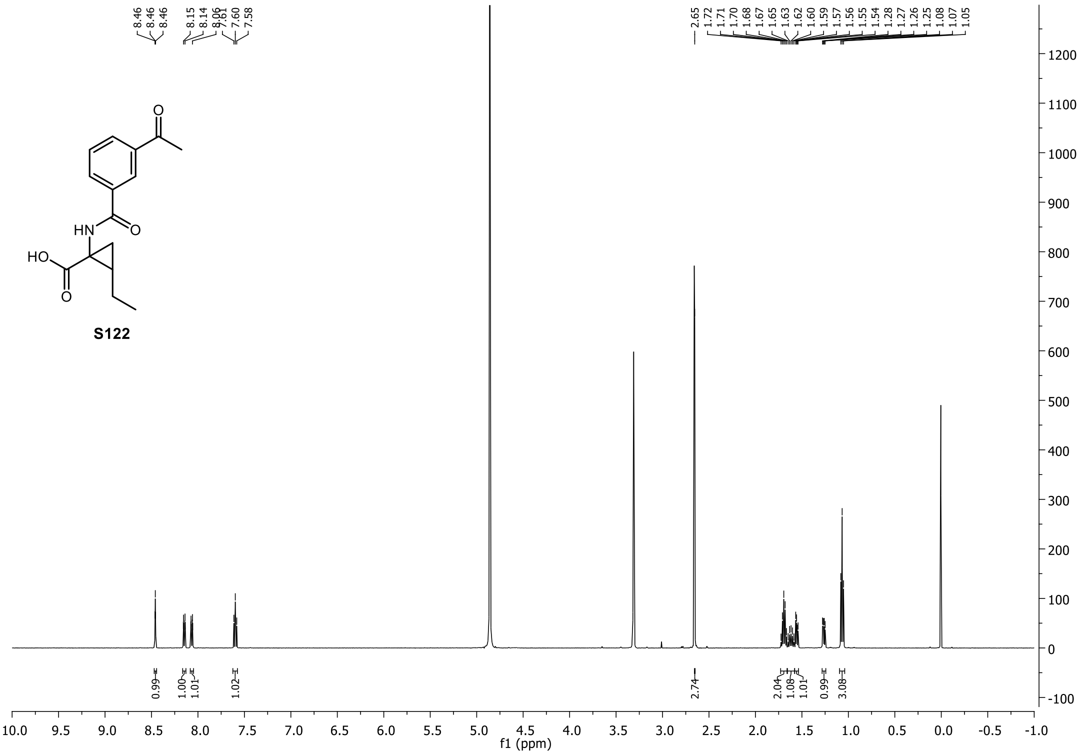

Supplementary Figure 311: ^{13}C NMR S122.

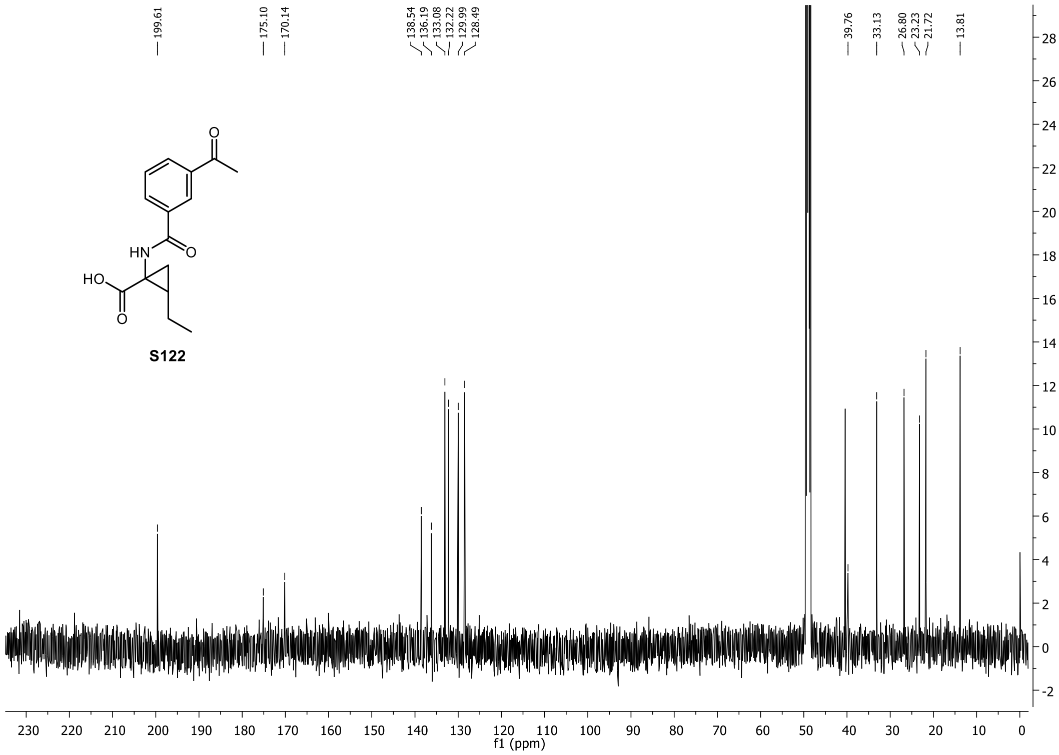

Supplementary Figure 312: ^1H NMR S123.

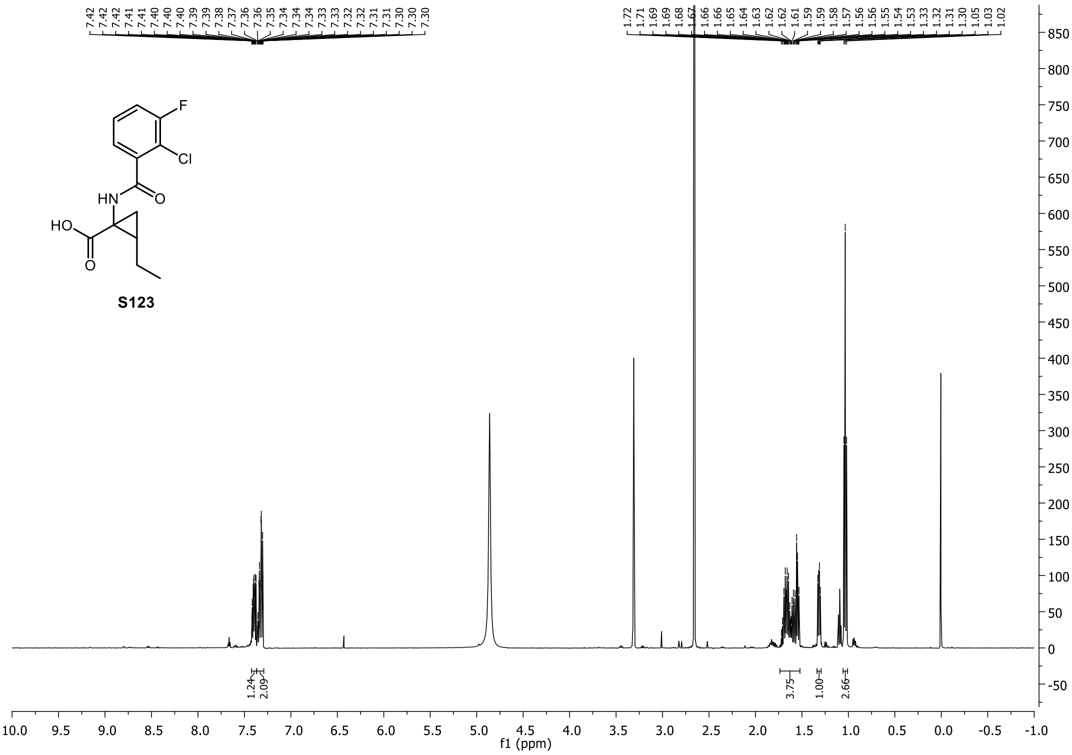

Supplementary Figure 313: ^{13}C NMR S123.

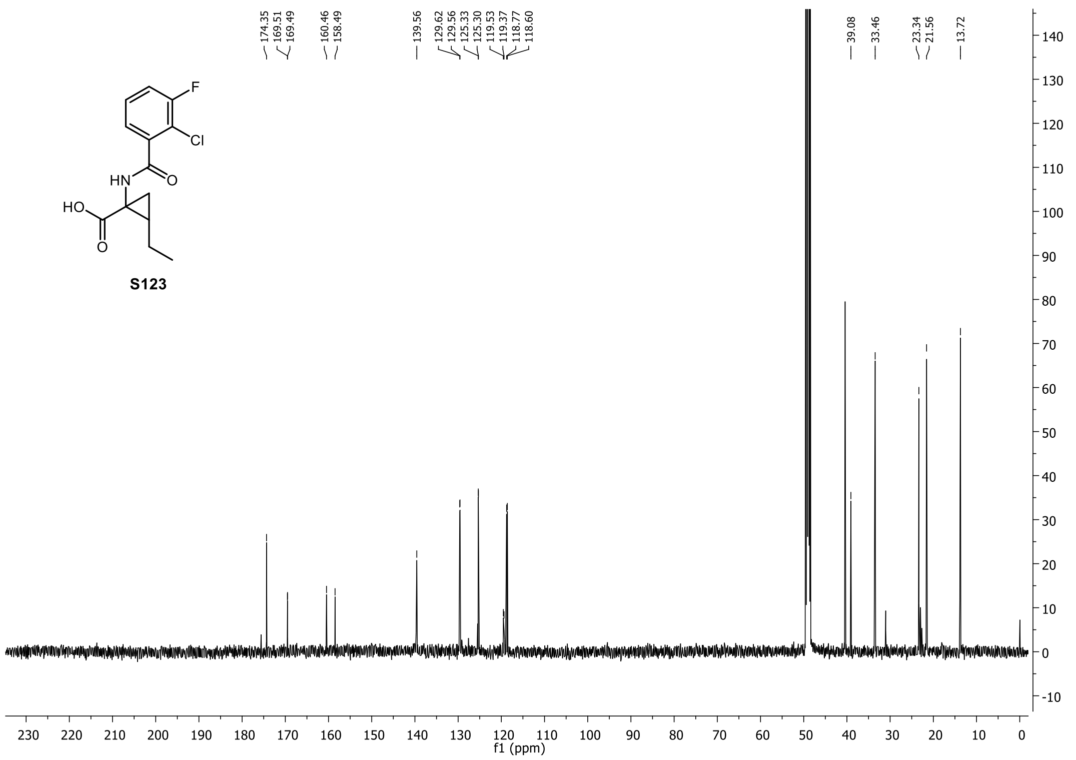

Supplementary Figure 314: ^{1}H NMR S124.

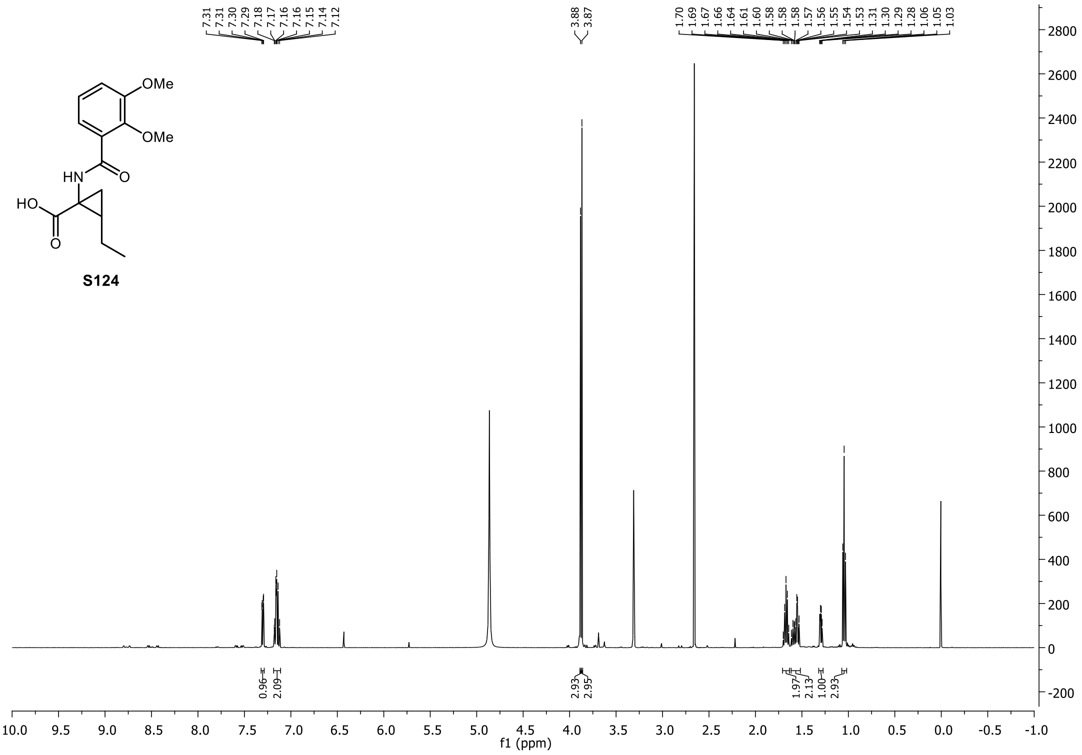

Supplementary Figure 315: ^{13}C NMR S124.

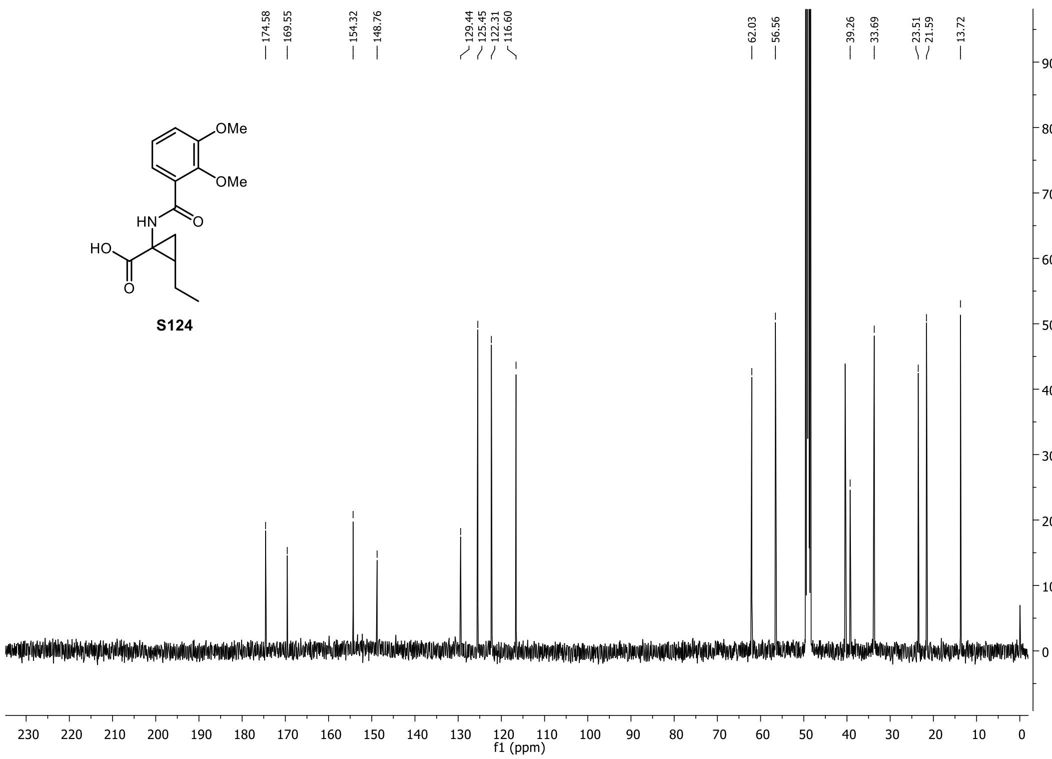

Supplementary Figure 316: ^{1}H NMR S125.

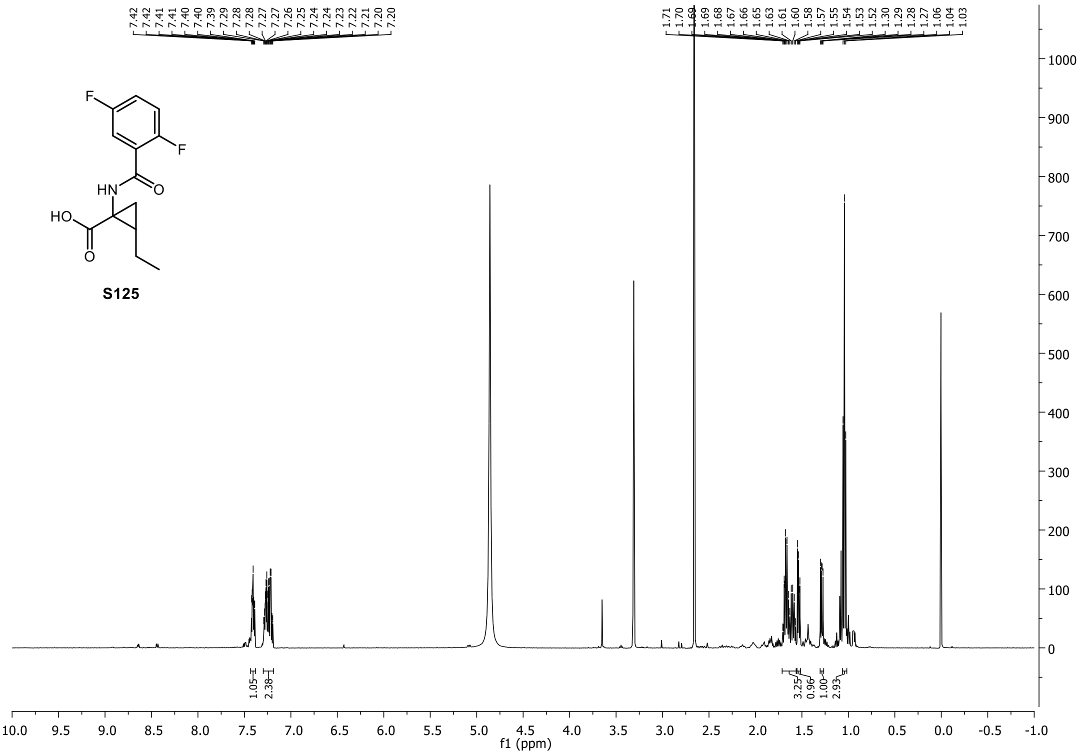

Supplementary Figure 317: ^{13}C NMR S125.

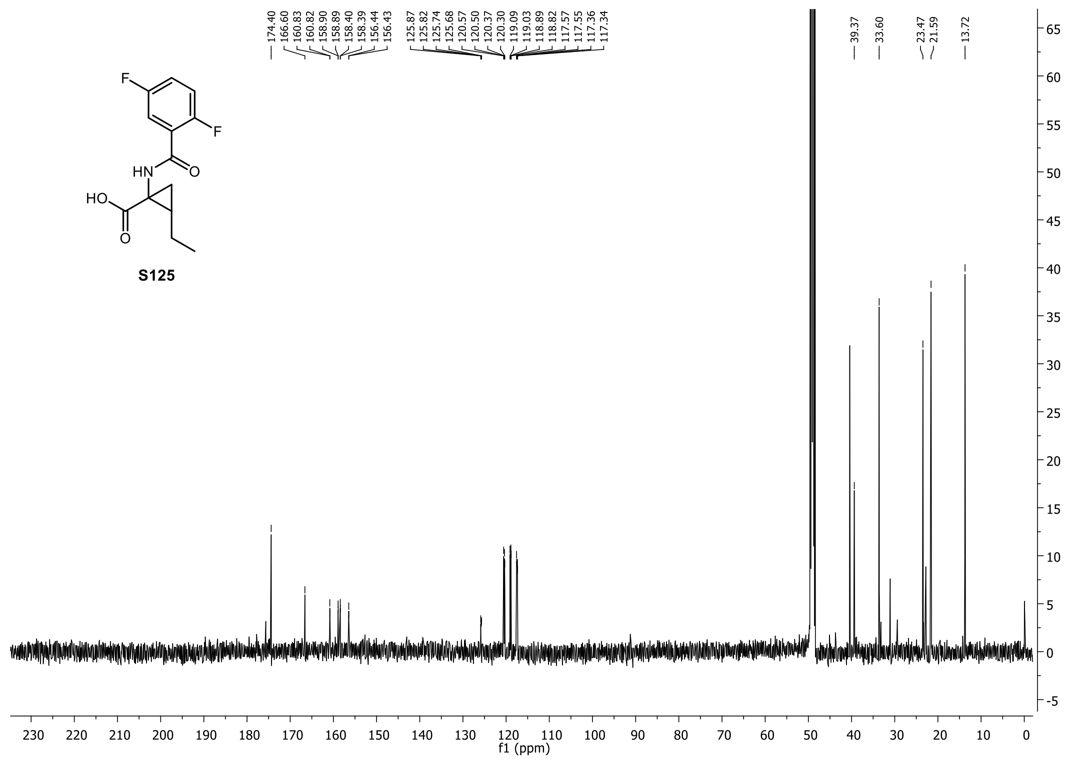

Supplementary Figure 318: ^{1}H NMR S126.

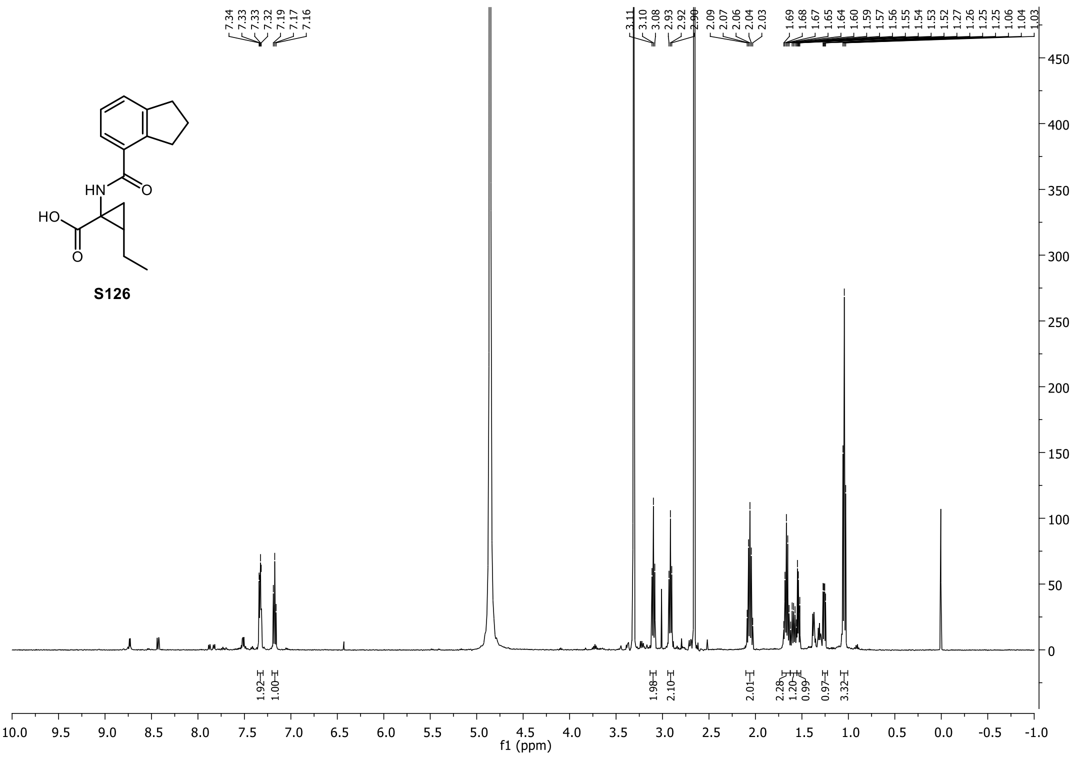

Supplementary Figure 319: ^{13}C NMR S126.

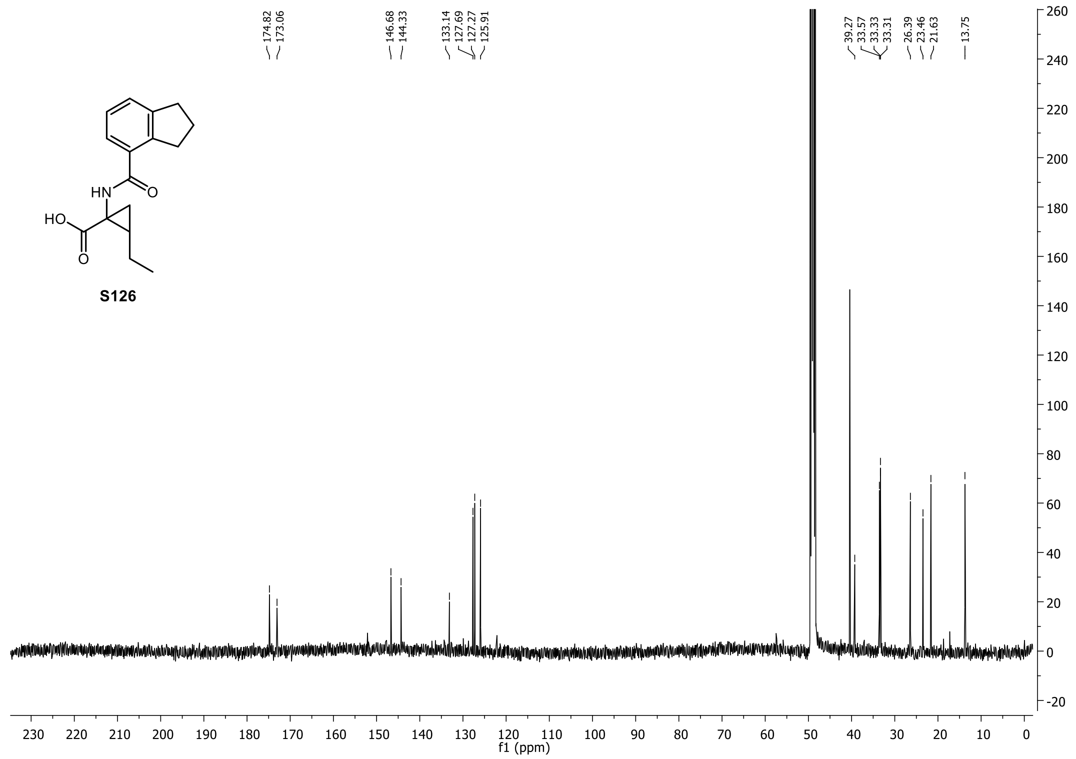

Supplementary Figure 320: ^{1}H NMR S127.

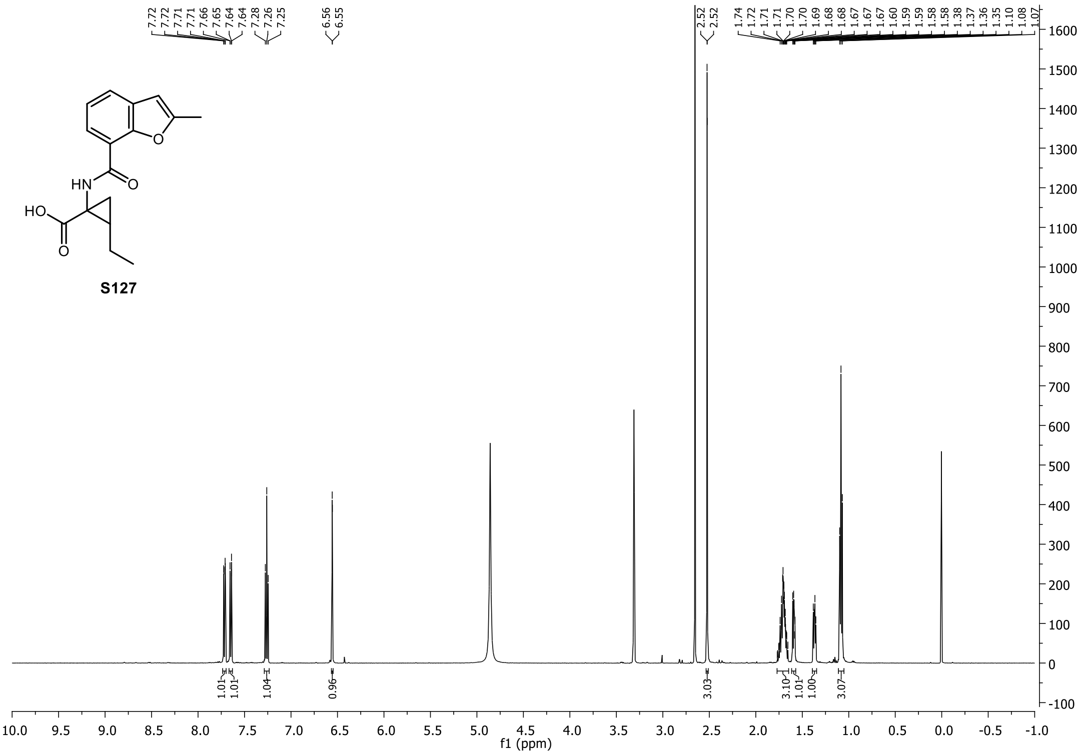

Supplementary Figure 321: ^{13}C NMR S127.

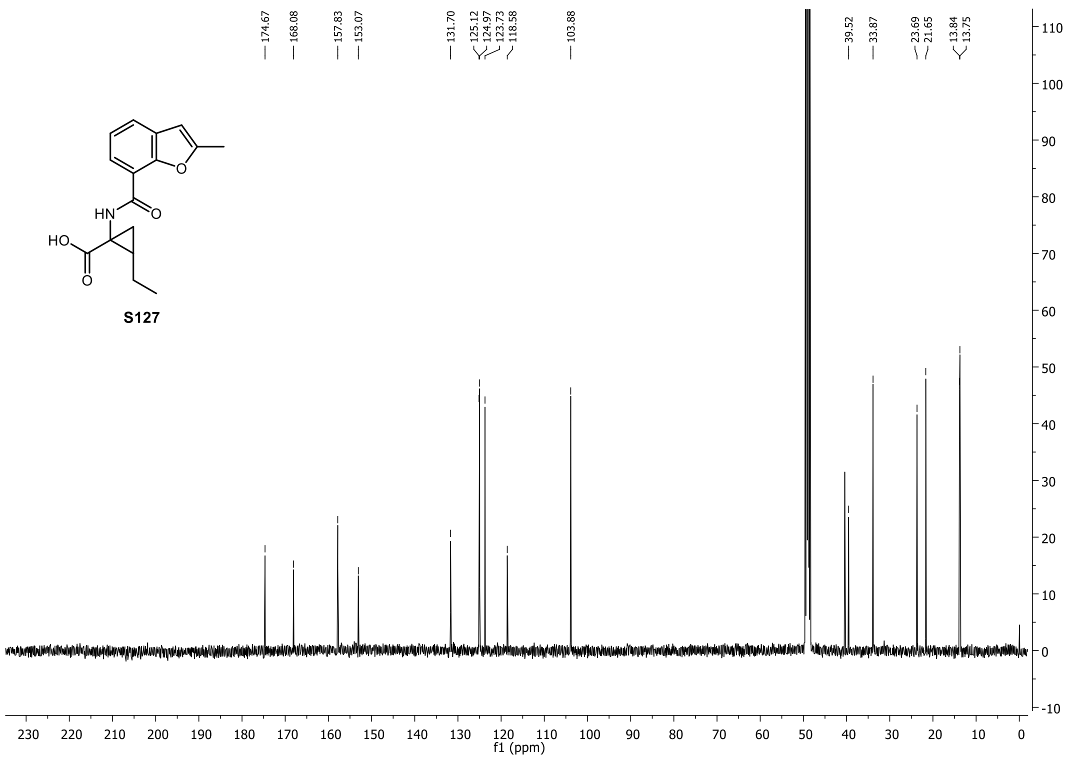

Supplementary Figure 322: ^{1}H NMR S128.

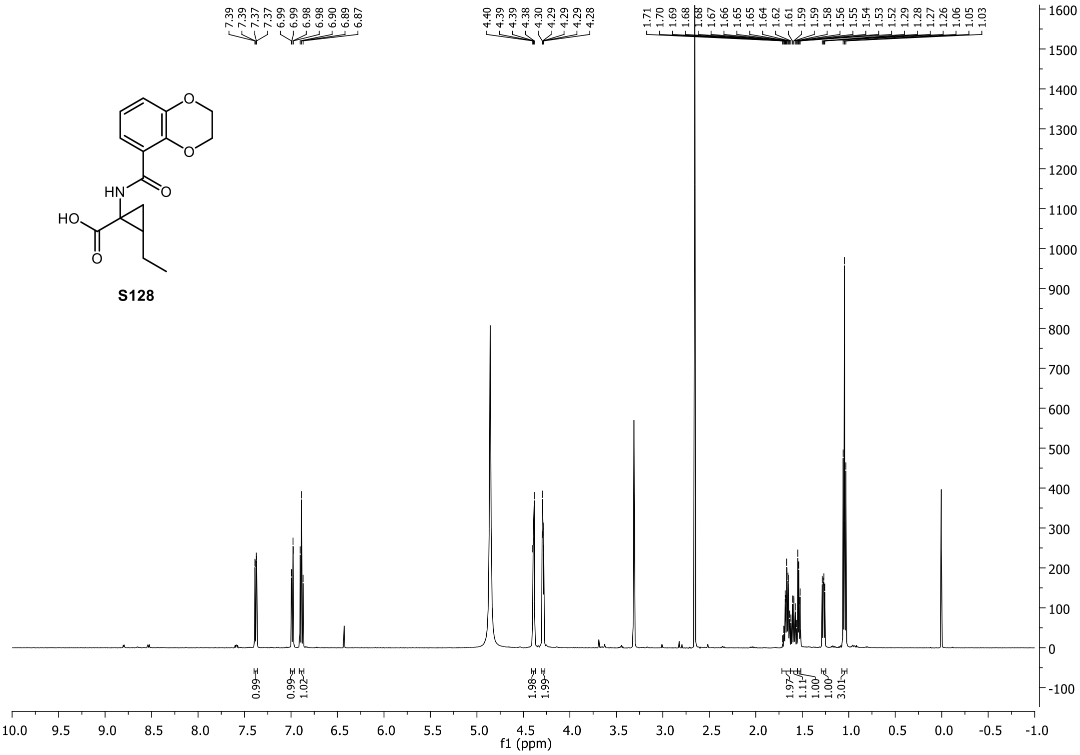

Supplementary Figure 323: ^{13}C NMR S128.

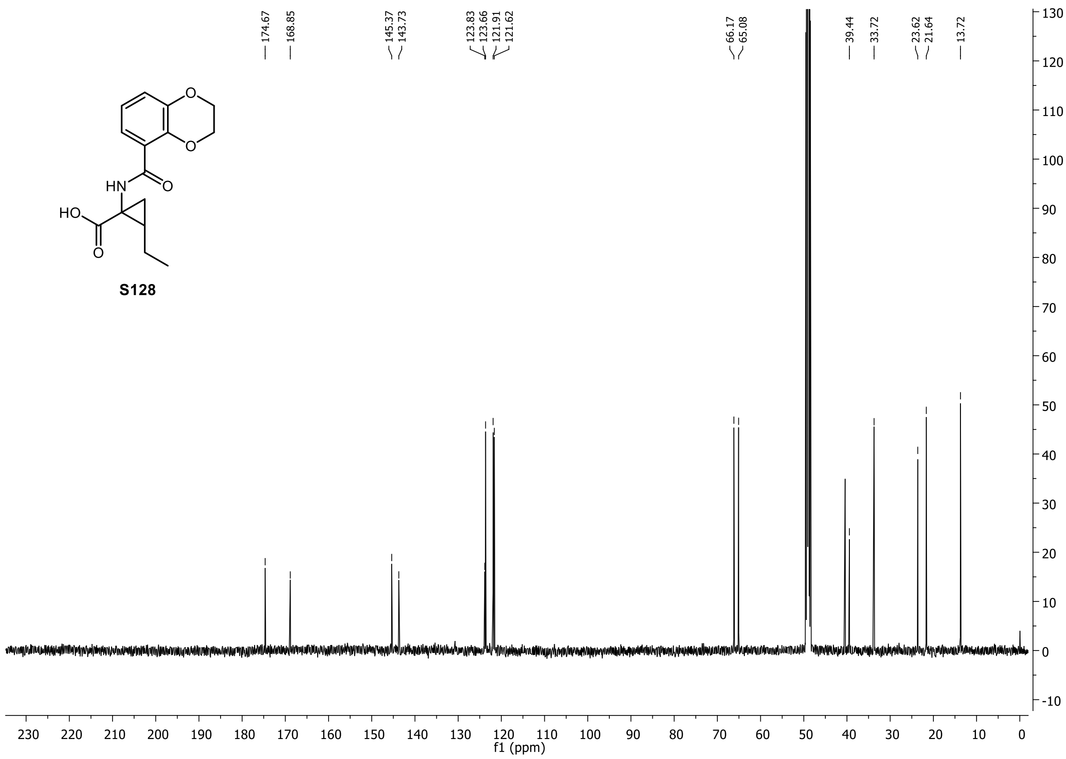

Supplementary Figure 324: ^{1}H NMR S129.

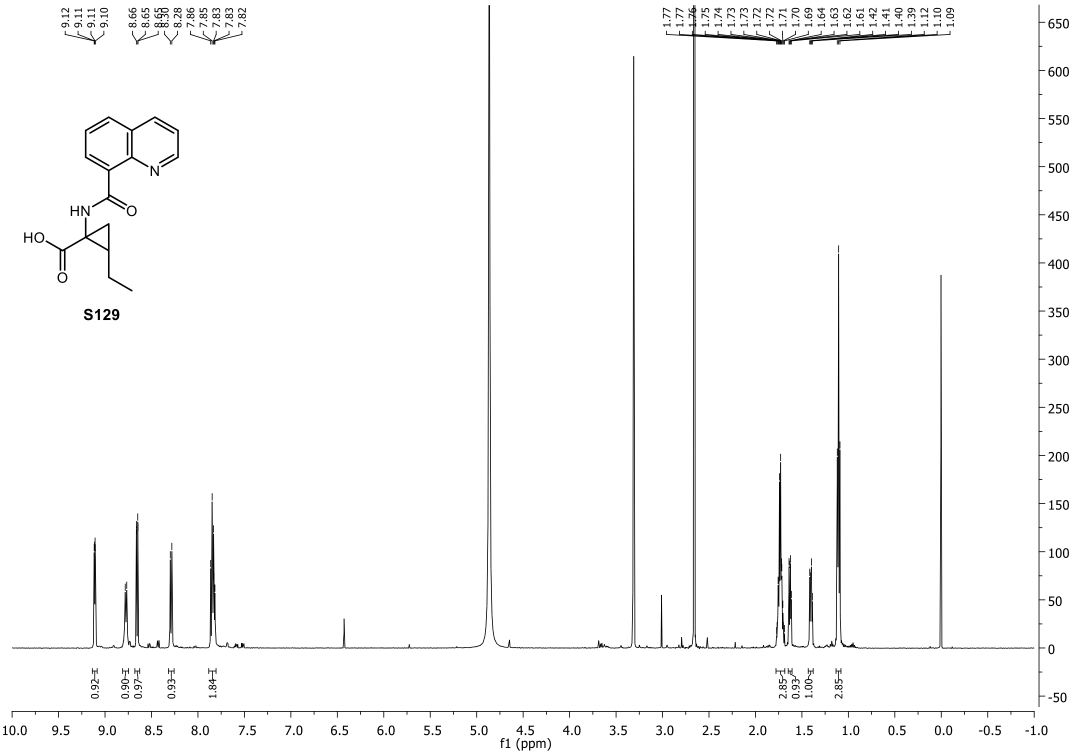

Supplementary Figure 325: ^{13}C NMR S129.

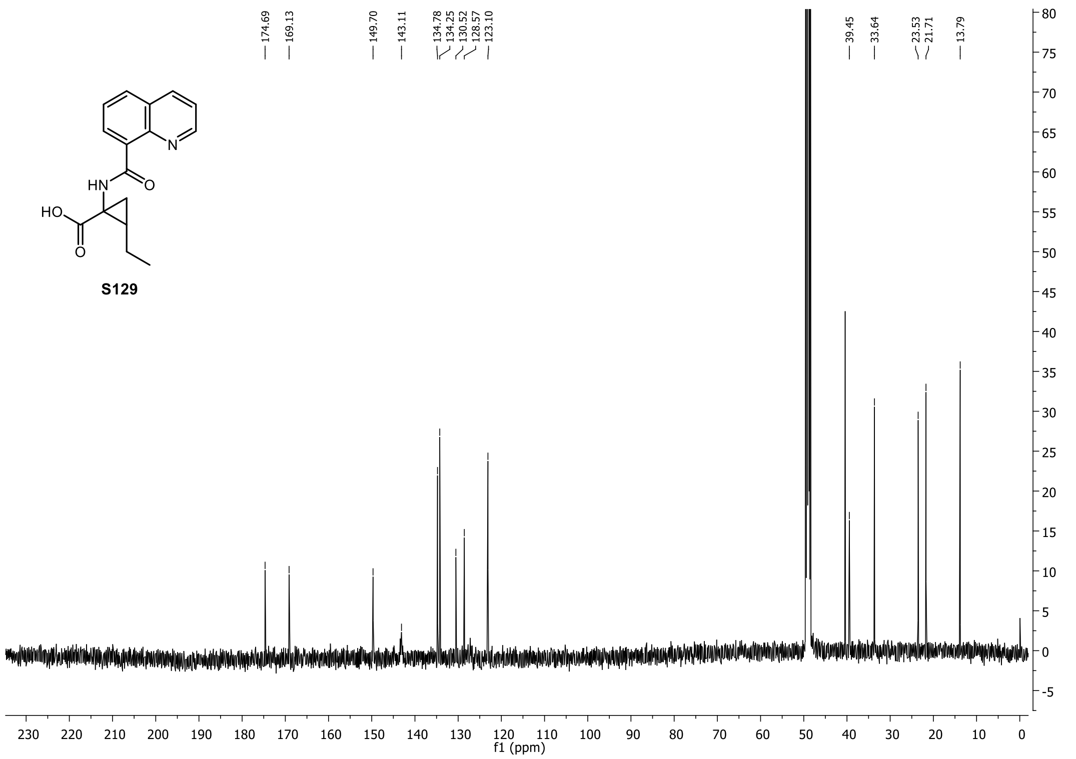

Supplementary Figure 326: ^{1}H NMR S130.

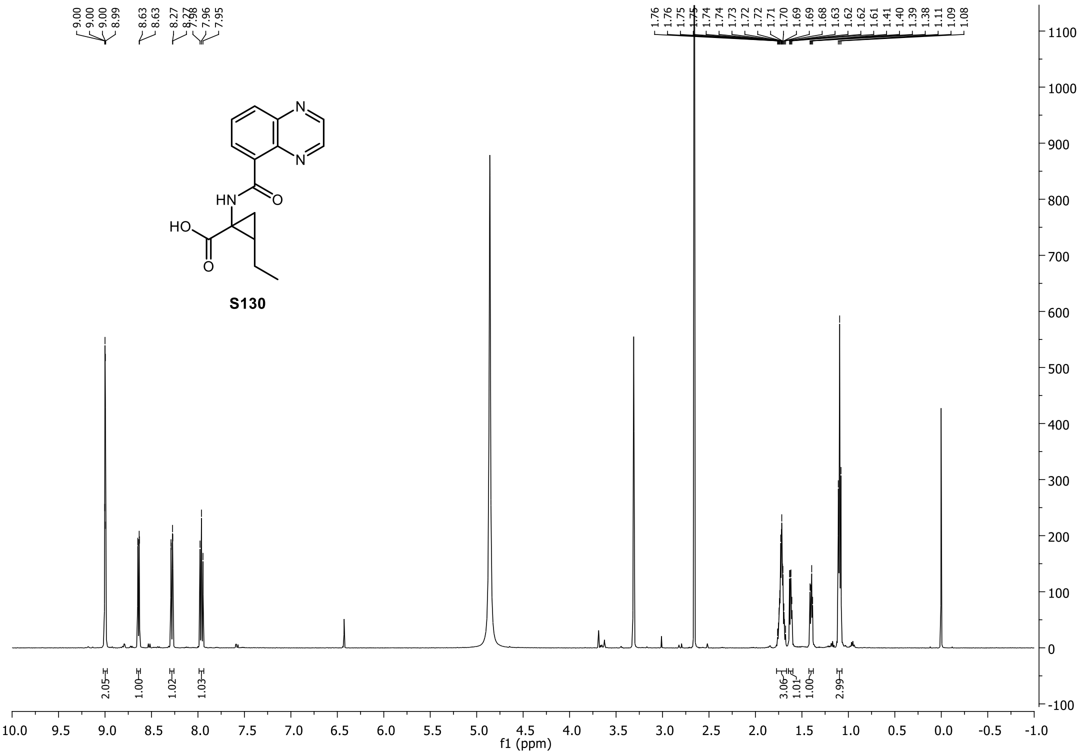

Supplementary Figure 327: ^{13}C NMR S130.

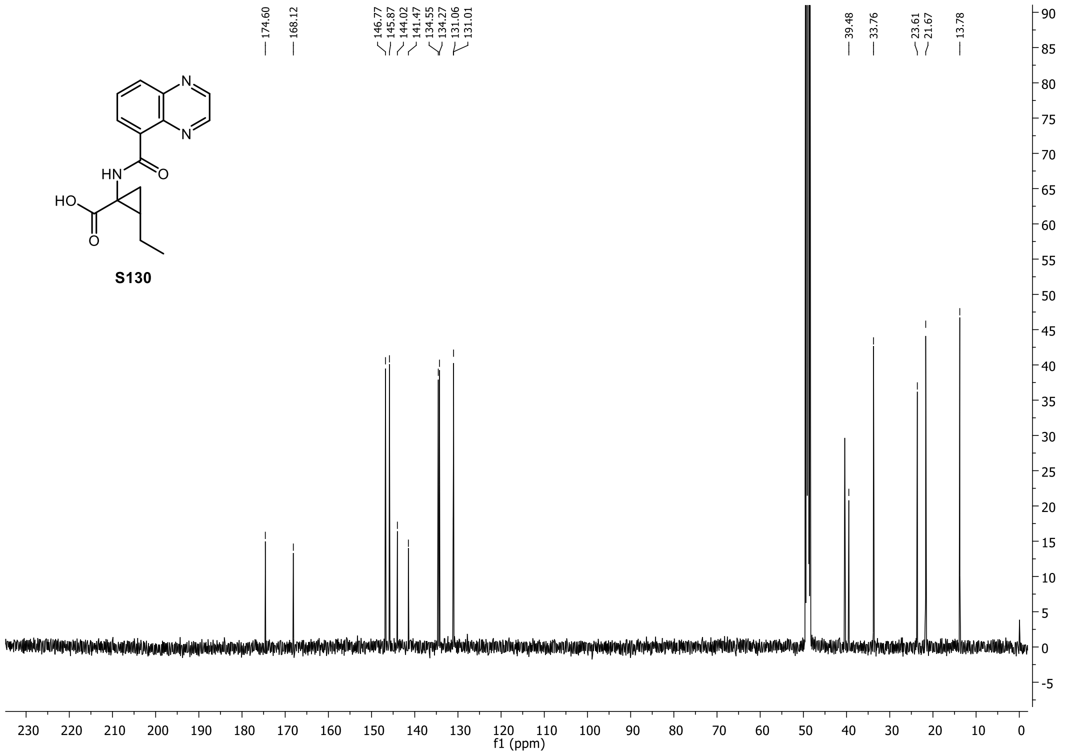

Supplementary Figure 328: ^{1}H NMR 45a, isomer 1.

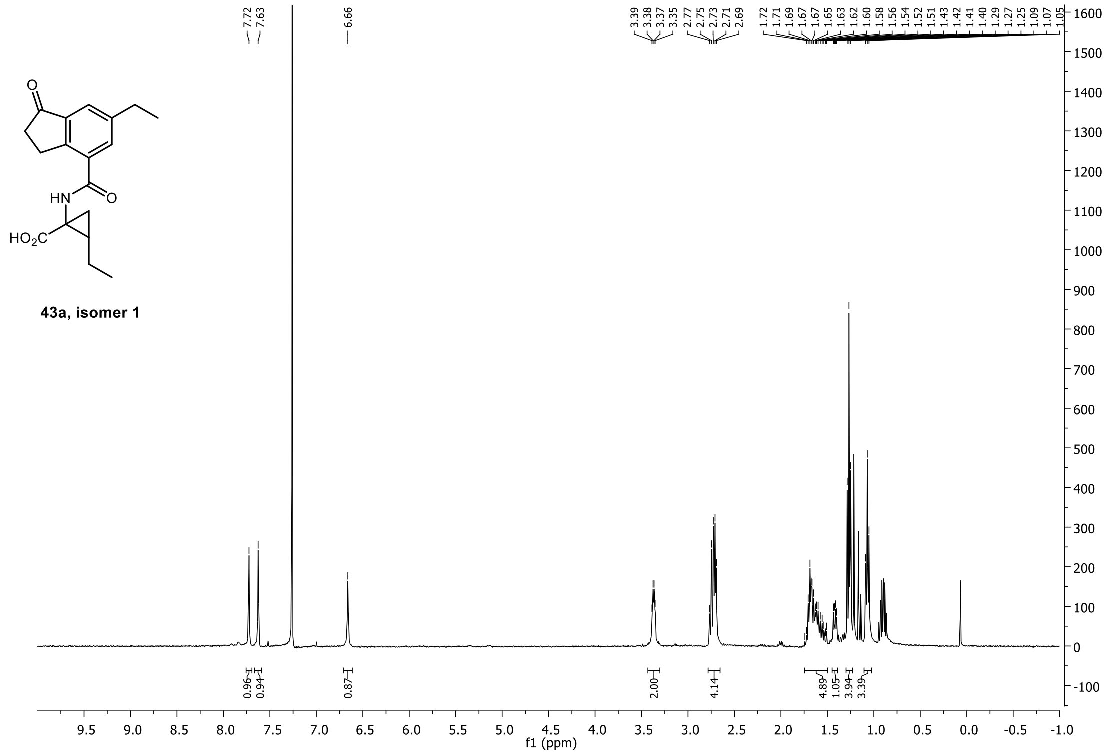

Supplementary Figure 329: ^{13}C NMR 45a, isomer 1.

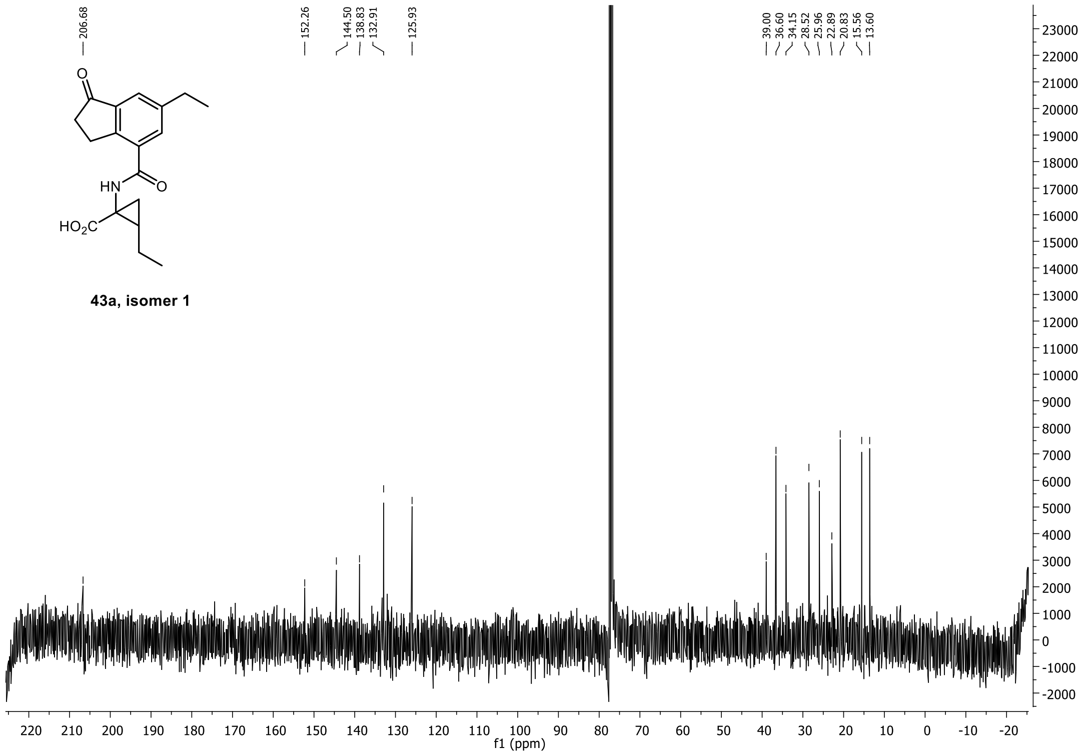

Supplementary Figure 330: ^{1}H NMR 45a, isomer 2.

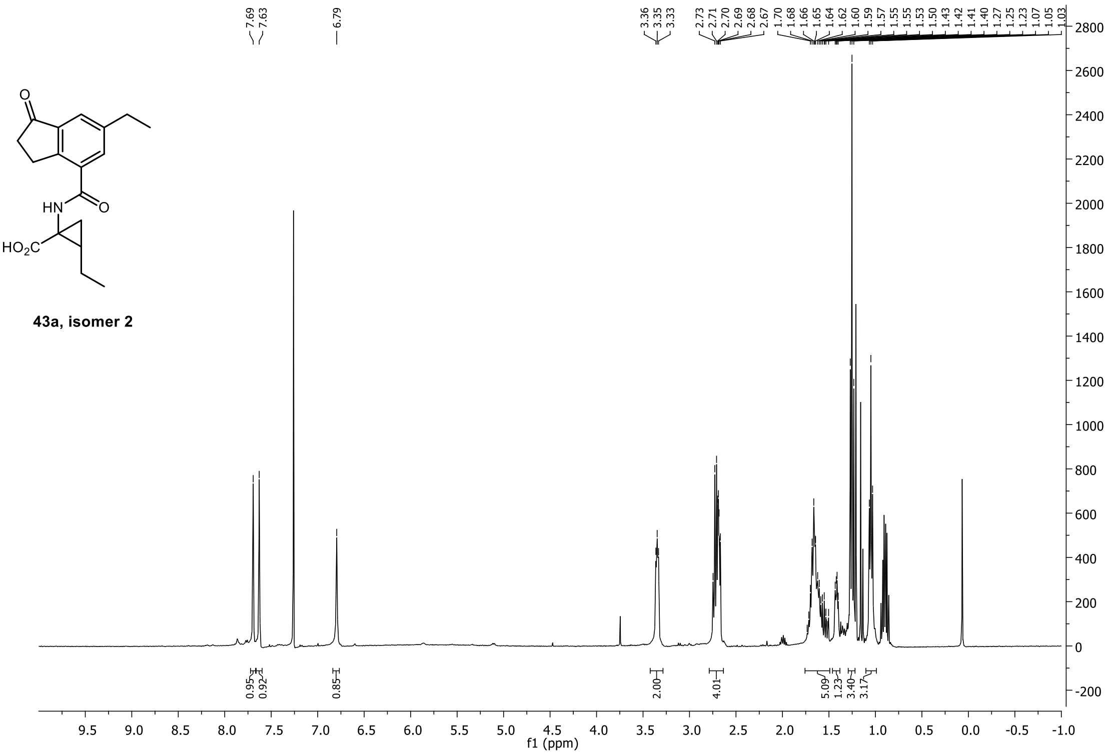

Supplementary Figure 331: ^{13}C NMR 45a, isomer 2.

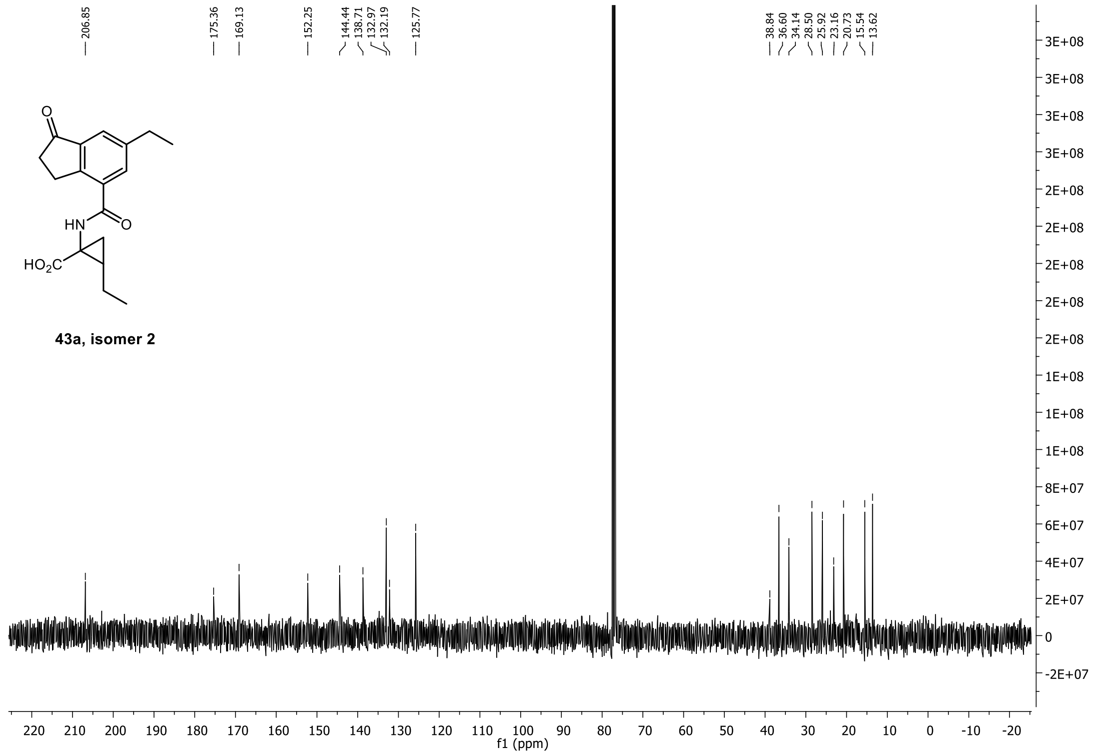

Supplementary Figure 332: ^{1}H NMR 43a, isomer 1.

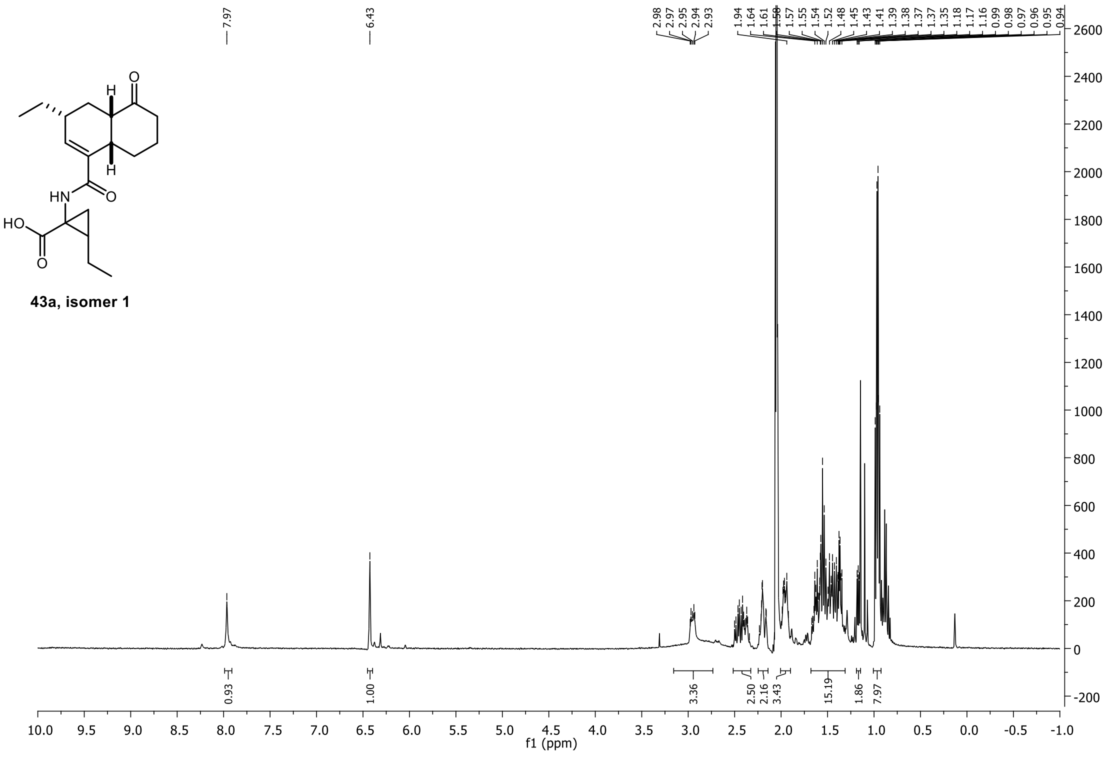

Supplementary Figure 333: ^{13}C NMR 43a, isomer 1.

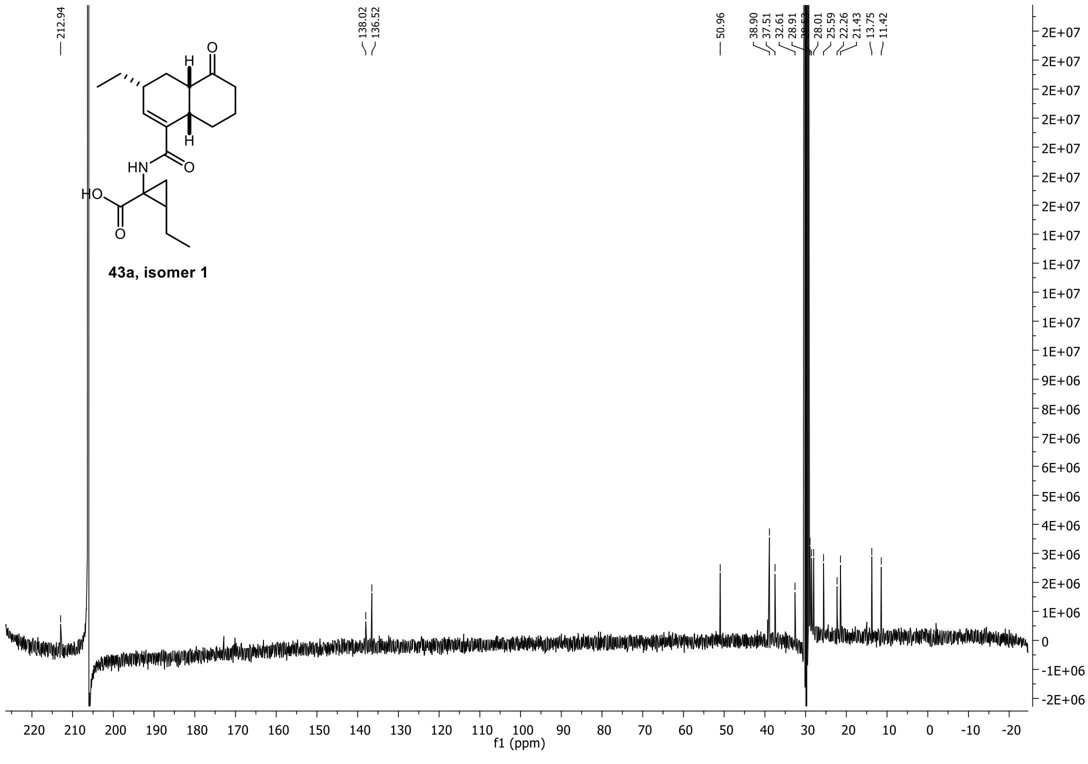

Supplementary Figure 334: ^{1}H NMR 43a, isomer 2.

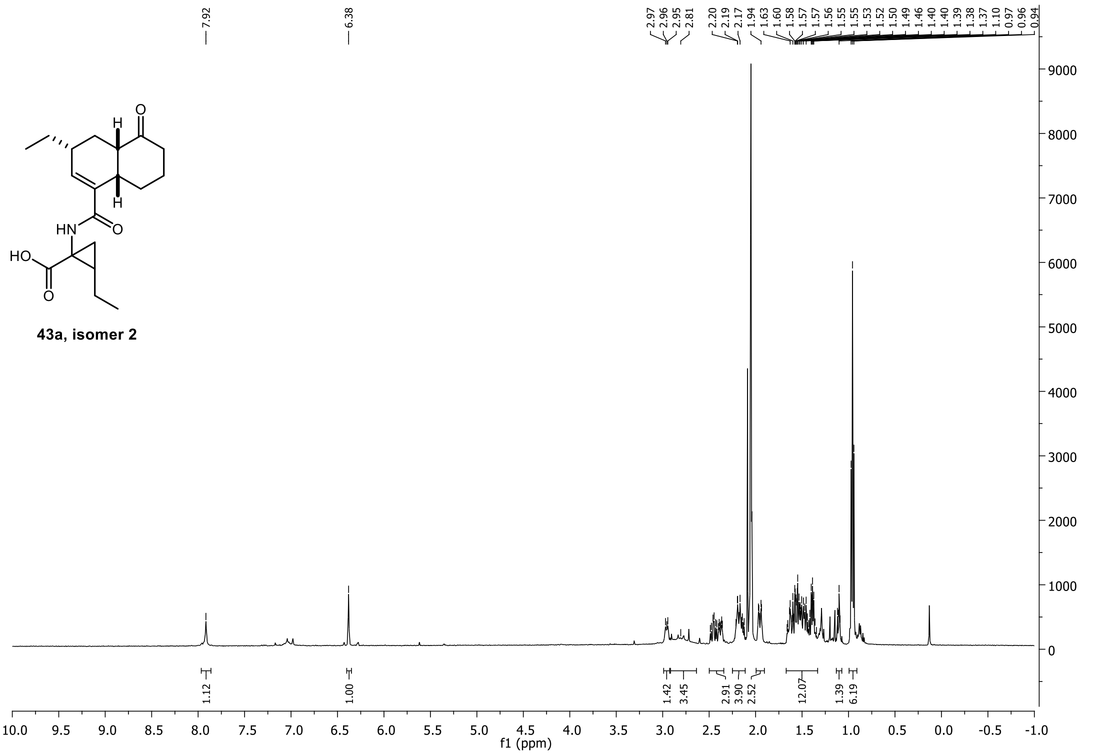

Supplementary Figure 335: ^{13}C NMR 43a, isomer 2.

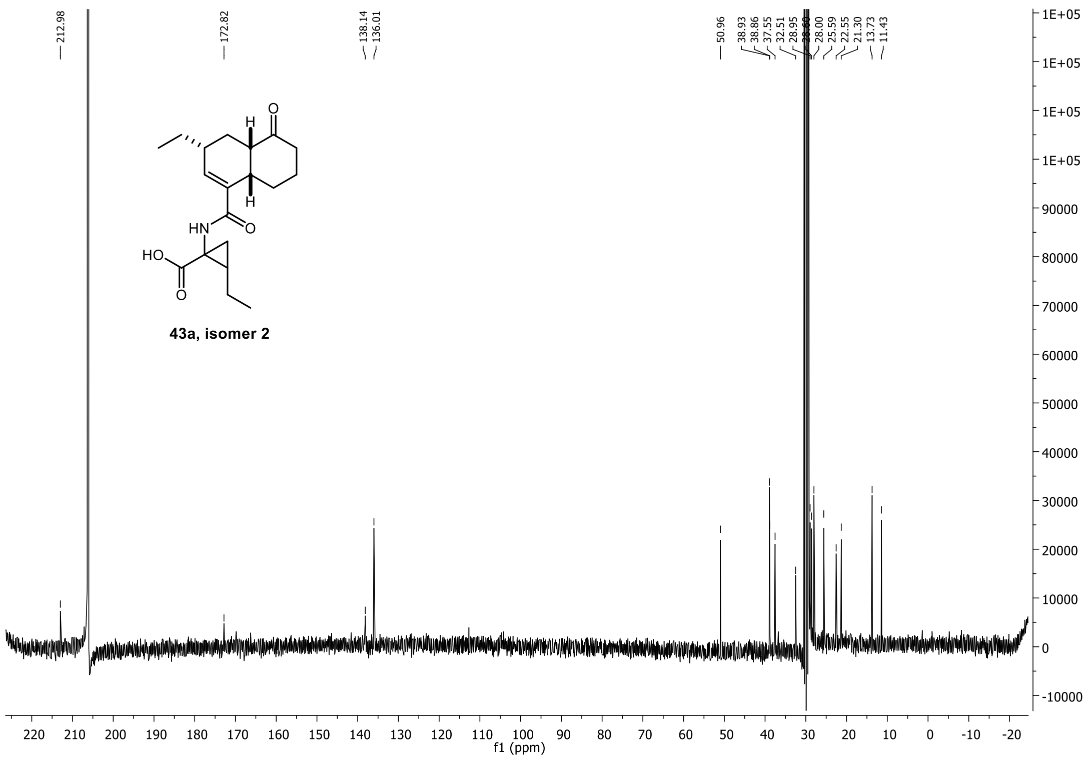

Supplementary Figure 336: ^{1}H NMR 43a, isomer 3.

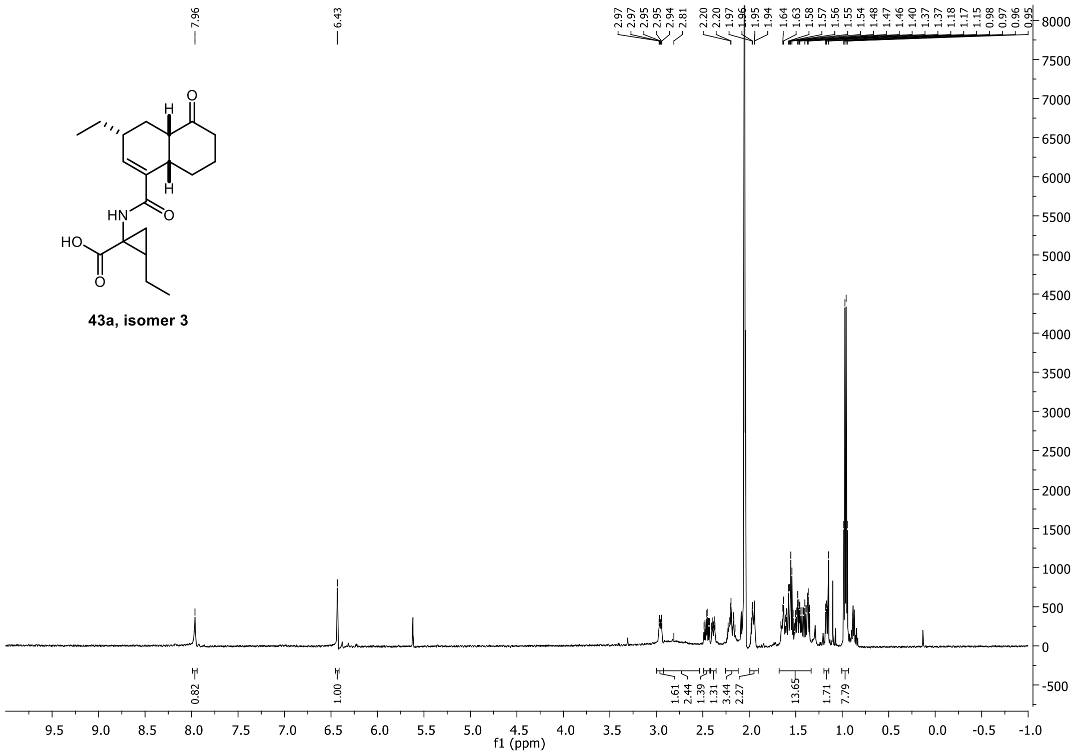

Supplementary Figure 337: ^{13}C NMR 43a, isomer 3.

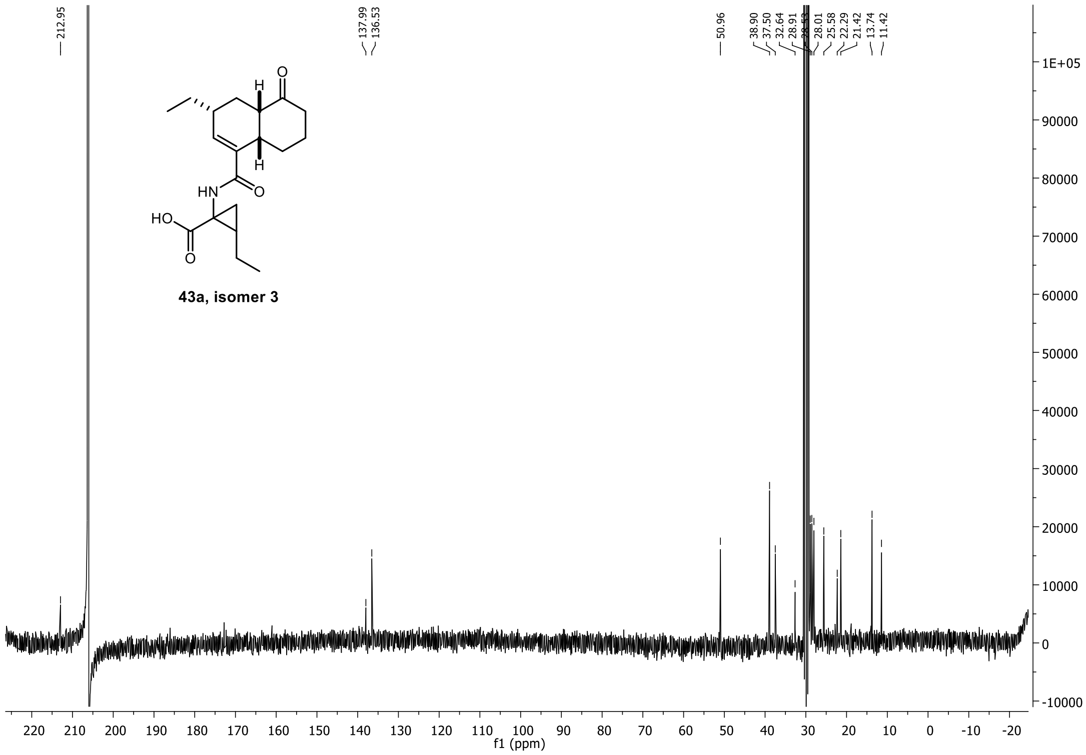

Supplementary Figure 338: ^{1}H NMR 43a, isomer 4.

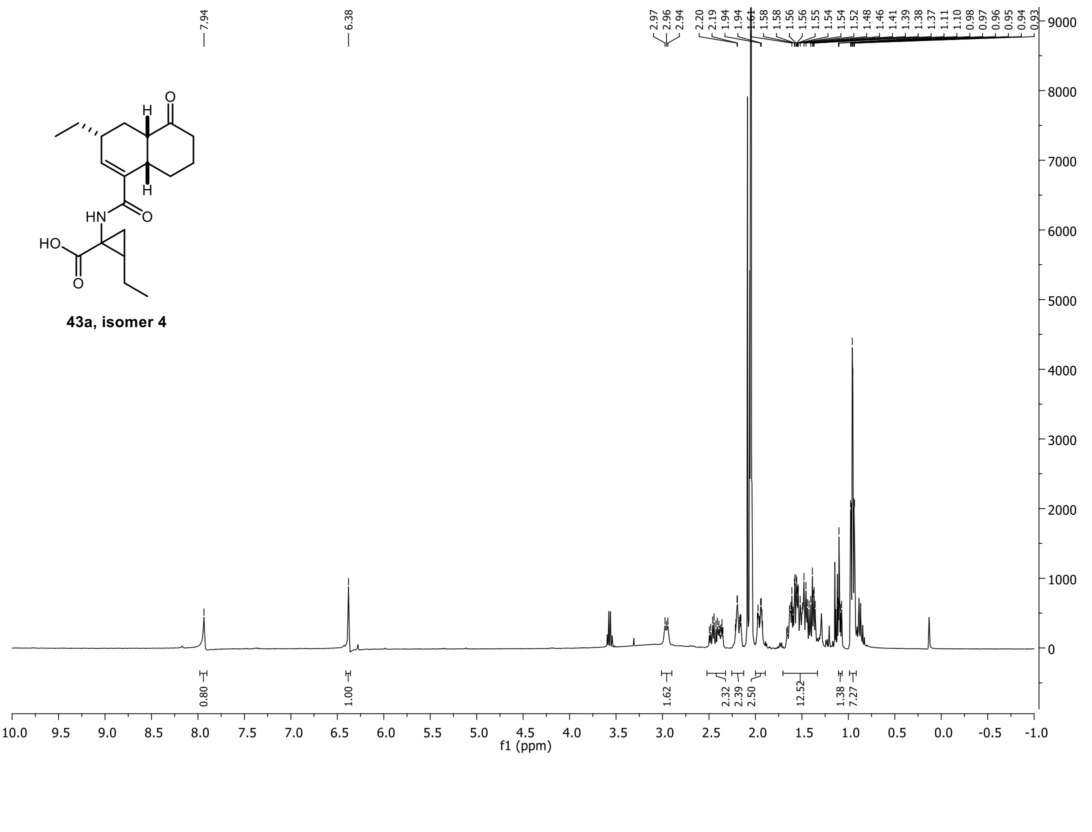

Supplementary Figure 339: ^{13}C NMR 43a, isomer 4.

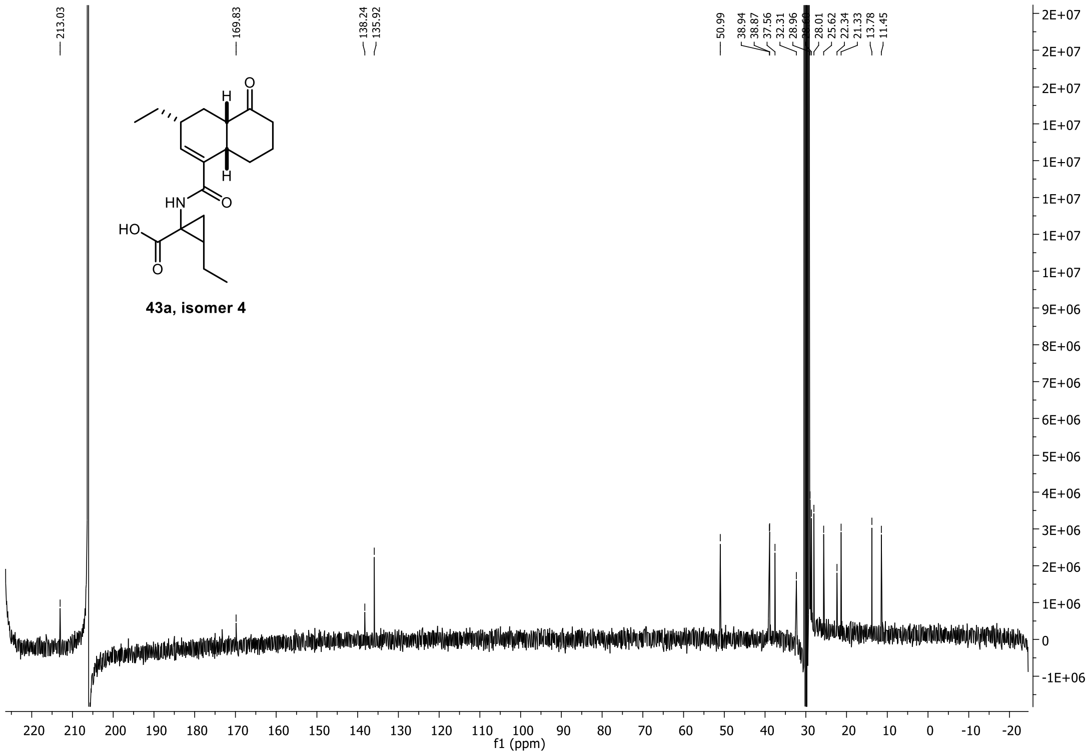

Supplementary References.

1. Armarego, W. L. F. & Chai, C. L. L. *Purification of Laboratory Chemicals*, Elsevier Inc. (Oxford, 2009).
2. Berman, H. M. *et al.* The Protein Data Bank. *Nucleic Acids Res.* ***28***, 235−242 (2000).
3. Sheard, L. B. *et al*. Jasmonate Perception by Inositol-Phosphate-potentiated COI1–JAZ Co-receptor. *Nature* **468,** 400–405 (2010).
4. G. M. Sastry, G. M., Adzhigirey, M., Day, T., Annabhimoju, R. & Sherman, W. Protein and Ligand Preparation: Parameters, Protocols, and Influence on Virtual Screening Enrichments. *J. Comput. Aided Mol. Des.* **27,** 221−234 (2013).
5. Friesner, R. A. *et al.* Glide: A New Approach for Rapid, Accurate Docking and Scoring. 1. Method and Assessment of Docking Accuracy. *J. Med. Chem.* **47,** 1739−1749 (2004).
6. T. A. Halgren, T. A. *et al.* Glide: A New Approach for Rapid, Accurate Docking and Scoring. 2. Enrichment Factors in Database Screening. *J. Med. Chem.* **47,** 1750−1759 (2004).
7. Schrödinger Release 2017-1: Maestro, Schrödinger, LLC, New York, NY (2017).
8. Reddy, G. V. *et al.* Novel Malyngamide Structural Analogs: Synthesis and Biological Evaluation. *Med. Chem. Res.* **22,** 4581–4591 (2013).
9. Crévisy, C., Couturier, M., Dugave, C., Dory, Y. L. & Deslongchamps, P. Studies on the Formation of 14-Membered Macrocycles by Intramolecular Michael Addition. *Bull. Soc. Chim. Fr.* **132,** 360−370 (1995).
10. Moreau, B., Ginisty, M., Alberico, D. & Charette, A. B. Expedient Stereoselective Synthesis of Coronafacic Acid Through Intramolecular Diels-Alder Cyclization*. J. Org. Chem.* **72,** 1235−1240 (2007).
11. Kishore Kumar, G. D. & Baskaran, S. A Facile, Catalytic, and Environmentally Benign Method for Selective Deprotection of *tert*-Butyldimethylsilyl Ether Mediated by Phosphomolybdic Acid Supported on Silica Gel. *J. Org. Chem.* **70,** 4520–4523 (2005).
12. Toshima, H., Nara, S., Ichihara, A., Koda, Y. & Kikuta, Y. Syntheses and Potato Tuber-Inducing Activity of Coronafacic Acid Analogues. *Biosci. Biotechnol. Biochem.* **62,** 681−688 (1998).
13. Kosaki, Y., Ogawa, N., Wang, Q. & Kobayashi, Y. Synthesis of Coronafacic Acid *via* TBAF-Assisted Elimination of the Mesylate and Its Conversion to the Isoleucine Conjugate. *Org. Lett.* **13,** 4232−4235 (2011).
14. Egoshi, S. *et al.* Dual Function of Coronatine as a Bacterial Virulence Factor Against Plants: Possible COI1-JAZ-Independent Role. *RSC Adv*. **6,** 19404−19412 (2016).
15. Okada, M., Ito, S., Matsubara, A., Iwakura, I., Egoshi, S. & Udea, M. Total Syntheses of Coronatines by *exo*-Selective Diels-Alder Reaction and their Biological Activities on Stomatal Opening. *Org. Biomol. Chem.* **7,** 3065−3073 (2009).
16. Crane, Z. D., Nichols, P. J., Sammakia, T. & Stengel, P. J. Synthesis of Methyl-1-(*tert*-butoxycarbonylamino)-2-vinylcyclopropanecarboxylate *via* a Hofmann Rearrangement Utilizing Trichloroisocyanuric Acid as an Oxidant. *J. Org. Chem.* **76,** 277–280 (2011).
17. Wullschleger, C. W., Gertsch, J. & Altmann, K.-H. Stereoselective Synthesis of a Monocyclic Peloruside A Analogue. *Org. Lett.* **12,** 1120–1123 (2010).
18. Cho, A., Kim, C. U. & Sheng, X. C. *Antiviral Compounds*, WO2007009109 A2 (2007).
19. Toshima, H. & Ichihara, A. Practical Stereoselective Syntheses of All Four Stereoisomers of Coronamic Acid (2-Ethyl-1-aminocyclopropane-1-carboxylic acid). *Biosci. Biotech. Biochem.* **59,** 497−500 (1995).
20. G. Schüler, W. Boland, R. Lauchli, *6-Substituted Indanoyl Amino Acid Conjugates as Mimics to the Biological Activity of Coronatine*, **2002**, WO2002055480 A3.
